# ChIP-Atlas 2025 update: 10-year anniversary of a data-mining platform for exploring epigenomic landscape

**DOI:** 10.1093/nar/gkag378

**Published:** 2026-04-29

**Authors:** Zhaonan Zou, Tazro Ohta, Takeya Kasukawa, Shinya Oki

**Affiliations:** Institute of Resource Development and Analysis, Kumamoto University, Gene Technology Center 6F, 2-2-1, Honjo, Chuo-ku, Kumamoto 860-0811, Japan; Department of Artificial Intelligence Medicine, Graduate School of Medicine, Chiba University, 1-8-1 Inohana, Chuo-ku, Chiba 260-8670, Japan; Institute for Advanced Academic Research, Chiba University, 1-33 Yayoicho, Inage-ku, Chiba 263-8522, Japan; Database Division for Life Science (DBCLS), BioData Science Initiative (BSI), National Institute of Genetics, Research Organization of Information and Systems, 1111 Yata, Mishima, Shizuoka 411-8540, Japan; RIKEN Center for Integrative Medical Sciences, 1-7-22 Suehiro-cho, Tsurumi-ku, Yokohama, Kanagawa 230-0045, Japan; Institute of Resource Development and Analysis, Kumamoto University, Gene Technology Center 6F, 2-2-1, Honjo, Chuo-ku, Kumamoto 860-0811, Japan

## Abstract

ChIP-Atlas (https://chip-atlas.org/) is a data-mining platform that systematically processes and integrates public epigenomic profiling data, including ChIP-seq, ATAC-seq, DNase-seq, and Bisulfite-seq, together with curated sample metadata, currently encompassing >460 000 experiments across multiple model organisms. In this 2025 update, an experiment-level quality control framework was embedded in every experiment page, enabling intuitive assessment of data reliability and representativeness. In addition, a parametric analysis of gene set enrichment-based module was implemented, allowing enrichment analysis directly from continuous RNA-seq count tables without relying on threshold-defined, discrete gene lists, thereby helping elucidate underlying gene regulatory mechanisms. Combined with sustained data expansion, these updates advance ChIP-Atlas into a more quantitative and flexible platform for exploring the epigenomic landscape in multicellular organisms, marking its 10th anniversary.

## Graphical Abstract

**Graphical Abstract:**
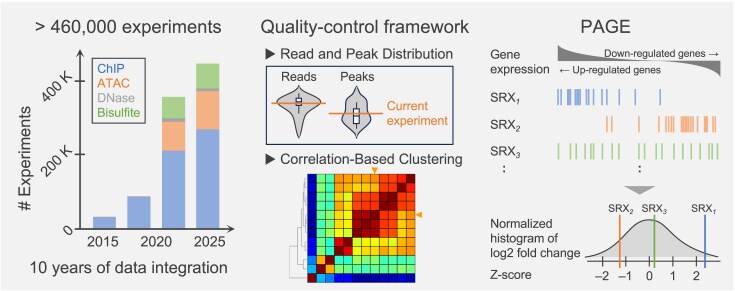

## Introduction

A central challenge in multicellular biology is deciphering the regulation of cell-type-specific gene expression. Although all somatic cells share the same genome, their distinct characteristics result from epigenomic states shaped by transcription factors (TFs), histone modifications, chromatin accessibility, and DNA methylation. Over the past decade, advances in high-throughput sequencing technologies, such as ChIP-seq, ATAC-seq, DNase-seq, and Bisulfite-seq, have generated vast data describing these epigenetic features. Most raw datasets have been archived in public repositories such as the NCBI Sequence Read Archive (SRA) [1], where each experiment is assigned a unique accession identifier (e.g. SRX IDs), representing an individual sequencing experiment. These datasets constitute a vast yet underutilized resource for understanding transcriptional regulation in a data-driven manner.

The ChIP-Atlas project (https://chip-atlas.org/) was launched in 2015 to enable full exploitation and reuse of public domain data. The project systematically processes and integrates epigenomic profiling datasets through a unified computational pipeline. In its early stages (ChIP-Atlas 1.0 and 2.0) [2, 3], ChIP-Atlas standardized the alignment and calling of peaks for ChIP-seq, DNase-seq, ATAC-seq, and whole-genome Bisulfite-seq data from six model organisms. Users can access these datasets through integrated online tools, such as the Peak Browser and Enrichment Analysis, to explore genome-wide TF binding, histone modifications, chromatin accessibility, and DNA methylation status at specified genomic regions or gene loci. Genome- and epigenome-level annotation tracks were integrated in a subsequent major update (ChIP-Atlas 3.0) [4], enabling users to visualize disease-associated single nucleotide polymorphisms alongside cell-type-specific enhancers and Hi-C-based chromatin conformations directly within the platform. The update also introduced the Diff Analysis module, allowing users to identify differential epigenomic states between experimental conditions. Since then, continuous monthly updates and systematic metadata curation have established ChIP-Atlas as one of the most comprehensive and actively maintained platforms for epigenomic data exploration and mining.

Despite this progress, two longstanding challenges remain. First, data quality varies. As ChIP-Atlas aims to provide comprehensive coverage by incorporating nearly all experiments registered in the SRA, no explicit quality filtering is performed during data collection. Although this inclusiveness supports an unbiased view of epigenomic features, datasets of widely varying reliability coexist within the platform, making it difficult for users to determine whether a given experiment is reliable enough for downstream analyses. Second, the Enrichment Analysis module, which is used in many studies to interpret user-supplied RNA-seq data and infer the underlying gene regulatory landscape, requires user-defined gene lists, typically differentially expressed genes (DEGs) obtained from RNA-seq experiments. In many practical situations, obtaining a sufficient number of robust DEGs is difficult due to limited effect or sample sizes. Even when possible, user-defined thresholding often discards genes that show statistically modest yet biologically meaningful changes, leading to information loss and potential bias in downstream enrichment analyses.

In celebration of its 10th anniversary and to address the above challenges, we introduce the ChIP-Atlas 2025 update. This version implements an experiment-level quality-control framework that makes data reliability explicitly assessable for each experiment and introduces a parametric analysis of gene set enrichment (PAGE)-based enrichment module [5] that extends RNA-seq-driven regulatory analysis beyond conventional DEG-centric workflows. Together, these developments advance ChIP-Atlas into a more quantitative data-mining platform for exploring the epigenomic landscape.

## Materials and methods

### Transcribed enhancers and promoters dataset

Cell type-specific cis-regulatory element (CRE) annotations for human (hg38) and mouse (mm10) genomes were obtained from fanta.bio (https://fanta.bio/) [6], which provides cap analysis of gene expression (CAGE)-derived enhancer and promoter catalogs [7]. These CRE tracks were downloaded in BED format and integrated into the Peak Browser “Annotation Tracks” section to allow the visualization of transcribed enhancers and promoters alongside ChIP-Atlas experimental tracks in Integrative Genomics Viewer (IGV) [8]. For each CRE, RNA 5′-ends captured by CAGE within the element were counted, normalized to counts per million (CPM) to account for sequencing depth, and subsequently scaled across samples using the relative log expression method [9] to enable meaningful sample-wise comparison. To display CRE activity in IGV in a quantitative manner, normalized CPM values were converted into BED scores (0–1000) to define the IGV color scale as follows: BED score = 0 (if CPM = 0); BED score = 1 (if CPM > 0 and log_2_CPM ≤ −0.5); BED score = ⌊((log_2_CPM + 0.5)/10.5) × 999⌋ + 1 (if CPM > 0 and − 0.5 < log_2_CPM < 10); and BED score = 1000 (if CPM > 0 and log_2_CPM ≥ 10). CRE tracks for legacy assemblies (hg19 and mm9) were additionally generated using liftOver (downloaded from the UCSC Genome Browser [10] on 31 May 2022) from the hg38/mm10 coordinates.

### Read and peak distribution

For each experiment type, which was further subcategorized for ChIP-seq into antigen classes (histone marks, TFs and others, RNA polymerase, and input controls), the distributions of sequencing read counts and called peak numbers were summarized across all experiments. For ChIP-seq, ATAC-seq, and DNase-seq, peak counts were based on peaks with MACS2 scores <50 [11], whereas for Bisulfite-seq experiments, “peaks” represented hypermethylated regions identified using MethPipe [12]. These distributions were represented visually through violin plots overlaid with box plots. The position of each individual experiment within its corresponding distribution was indicated by an orange horizontal line, thereby providing a cohort-level context for the depth and signal yield of the data. All plots were generated in Python (v3.12.3) using matplotlib (v3.6.3).

### Correlation-based clustering

For experiments sharing the same biological context, defined by the same genome assembly and cell type, as well as the same antigen for ChIP-seq, pairwise Pearson correlations of bigWig signal profiles were computed and visualized using deepTools (v3.5.5) [13]. Briefly, bigWig files were segmented into 10-kb genomic windows, and signal intensities were summarized across bins using the multiBigwigSummary subcommand (e.g. multiBigwigSummary bins -b srx1.bw srx2.bw srx3.bw -o results.npz). The resulting matrix was subsequently used as input for plotCorrelation to calculate correlation coefficients, generate heatmaps, and perform hierarchical clustering (e.g. plotCorrelation -in results.npz -c pearson -p heatmap -o plot.png --outFileCorMatrix). All analyses were conducted using default parameters.

### PAGE module

Upon receiving a two-group integer-valued gene count table (TSV or CSV), log_2_ fold changes (log_2_FC) for all genes between the two experimental groups are first estimated using the DESeq2 package (v1.40.2) [14] in R (v4.3.1). The input table contains gene names or IDs in the first column and a header row in which each subsequent column represents a single sample; column headers indicate the condition and replicate number separated by an underscore (e.g. treated_1, untreated_2). Gene identifiers provided as Ensembl, UniProt, or RefSeq IDs are automatically mapped to official gene symbols, and duplicate symbols are merged by summing their counts. Subsequently, for each ChIP-seq, ATAC-seq, or Bisulfite-seq experiment, the overlap between its peak regions and gene loci within a user-defined transcription start site (TSS) window is assessed, thereby constructing an experiment-specific “target gene set.” In accordance with the original PAGE framework [5], *Z*-scores are calculated by standardizing the mean log_2_FC of each target gene set against the genome-wide distribution of log_2_FC values. Specifically, *μ* and *δ* represent the mean and standard deviation of log_2_FC across all genes, respectively, whereas S*_m_* denotes the mean log_2_FC of a target gene set of size *m*. The *Z*-score is calculated as follows: *Z* = (S*_m_* – *μ*) × *m*^1/2^ / *δ*. Two-tailed *P*-values are then derived from the *Z*-scores, followed by multiple-testing correction using the Benjamini–Hochberg procedure to obtain *Q*-values. Scripts for the PAGE-based analysis are available in the ChIP-Atlas GitHub repository (https://github.com/inutano/chip-atlas/tree/v4.0.1/script/update_2025/PAGE) and Zenodo [15].

### RNA-seq dataset from tamoxifen-treated and -untreated MCF-7 cells

FASTQ files were downloaded from the NCBI Gene Expression Omnibus [16] under accession GSE178303. Reads were aligned to the hg38 reference genome using HISAT2 (v2.2.1) [17], and gene-level counts were subsequently obtained using featureCounts (v2.0.6) [18]. DEGs were identified using iDEP (v0.96) [19]. All analyses were performed using default parameters.

## Results

### Overview of the ChIP-Atlas 2025 update and a decade of progress

The 2025 update of ChIP-Atlas addresses two major challenges: the lack of explicit data quality information and the reliance of enrichment analysis on predefined gene lists. First, we implemented an experiment-level quality-control framework on the detail page of each experiment, enabling users to easily assess whether a selected dataset exhibits sufficient read and peak quality, shows consistency with comparable experiments, and is thus suitable for downstream analyses. Second, we introduced a PAGE-based module that performs enrichment analysis directly from continuous RNA-seq count tables, allowing users to detect coordinated gene set–level changes without relying exclusively on discrete DEG lists. The DEG-based enrichment analysis tool and PAGE function as complementary options within a unified interface, thereby extending the flexibility of ChIP-Atlas for interpreting RNA-seq data in the context of large-scale gene regulatory profiles (Table 1).

**Table 1.** Ten-year progress of ChIP-Atlas

Year	2015	2016	2017	2018	2019	2020	2021	2022	2023	2024	2025
Track type	ChIP-seq, DNase-seq						+ATAC-seq, Bisulfite-seq		+Annotation tracks		
Genome assembly	hg19, mm9		+rn6, dm3, ce10, sacCer3			+hg38, mm10, dm6, ce11					
Integrative analysis tool	Peak browser, target genes, colocalization	+Incremental dataset search	+Enrichment analysis						+Diff Analysis		+PAGE-based enrichment module
Cumulative number of experiments	37 720	52 249	72 199	90 322	131 903	158 863	196 136	361 856	408 697	459 231	464 655
Metadata	Manual curated metadata, original metadata, process log			CNN-assisted curation				SVM-assisted curation			+Data quality information (experiment comparative profile)
Data hosting server	NBDC					DBCLS					
Computing server	NIG										+Standby server on AWS
Publication				[2]				[3]		[3]	
Citations per year	0	1	15	29	69	112	178	197	219	254	361

PAGE, parametric analysis of gene set enrichment; CNN, convolutional neural network; SVM, support vector machine; NBDC, National Bioscience Database Center, Japan; DBCLS, Database Center for Life Science, Japan; NIG, National Institute of Genetics, Japan; AWS, Amazon Web Services.

Several additional refinements were implemented to improve user experience and system stability. First, we integrated the CAGE dataset for cell type-specific bidirectionally transcribed enhancers and promoters from the fanta.bio database (447 315 and 288 877 CREs for human and mouse, respectively) into the annotation tracks of Peak Browser, enabling users to examine regulatory elements alongside other epigenomic data within a unified interface (Supplementary Fig. S1 and  Supplementary Materials S1). Second, in previous versions, enrichment analysis was computationally demanding because overlaps between all called ChIP-seq peaks and TSS windows were calculated on demand for each user-submitted gene list. In the 2025 update, a precomputed strategy was adopted, in which peak–gene overlaps are calculated in advance for commonly used TSS windows (±1, ±5, and ±10 kb), substantially reducing computation time for both the DEG-based enrichment analysis tool and the newly implemented PAGE module. Third, we established a standby computation server to ensure uninterrupted service during scheduled maintenance events, such as annual power shutdowns, which previously required full suspension of the ChIP-Atlas web service. Collectively, these upgrades improved the efficiency and reliability of the ChIP-Atlas platform.

Since its initial release in 2015, ChIP-Atlas has grown into a comprehensive platform integrating diverse epigenomic profiling datasets. Initially focused on human and mouse ChIP-seq and DNase-seq data, it has expanded to include multiple model organisms with updated genome assemblies, additional assay types (ATAC-seq and Bisulfite-seq), and web-based tools supporting comparative epigenomic studies. Manual metadata curation has progressively been augmented via convolutional neural network- and support vector machine-based methods, improving the efficiency and scalability of large-scale annotation. By 2025, ChIP-Atlas had processed over 460 000 experiments and had been cited in >1400 publications worldwide, reflecting a decade of continuous growth and refinement as an epigenomic data infrastructure.

### Example of use: quality-control framework

**Figure 1.**
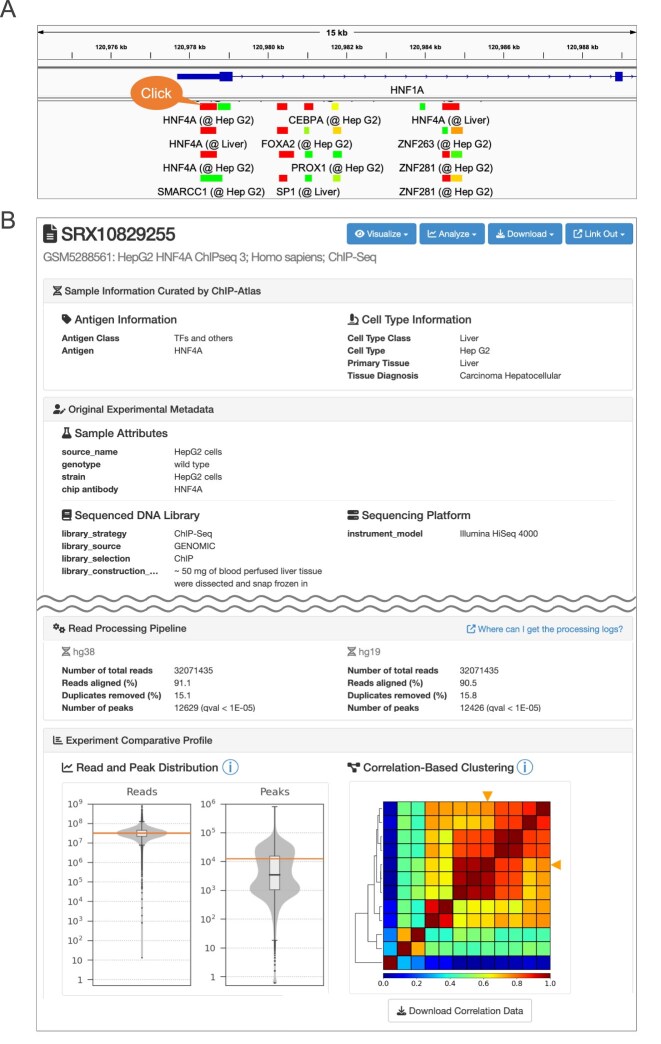
Example of a detailed experiment page. (**A**) Visualization of TF bindings around the *HNF1A* locus in human liver using IGV. (**B**) Detailed page of SRX10829255 (https://chip-atlas.org/view?id=SRX10829255). Curated and original metadata, processing information, and the newly implemented quality-control panel (Experiment Comparative Profile) are presented. The read and peak distribution plot shows the position of the selected experiment (orange horizontal line) relative to comparable datasets. The correlation heatmap shows pairwise Pearson correlations between experiments, with colors ranging from blue (low correlation) to red (high correlation). Hierarchical clustering groups experiments based on the similarity of genome-wide signal profiles. Arrowheads indicate the selected experiment, and the color reflects the median correlation of the selected experiment with other experiments within the same biological context.

As summarized in Table 1, ChIP-Atlas 2025 introduced the Experiment Comparative Profile, a new experiment-level quality-control panel integrated into each experiment page. The basic workflow for browsing and accessing data remains consistent with previous versions. For instance, users can examine TF binding around a gene of interest using the Peak Browser (Fig. 1A) and then click on a peak to open the corresponding experiment page (Fig. 1B). In this example, the *HNF1A* locus is displayed with multiple colored tracks representing ChIP-seq peaks for different TFs in liver-derived tissues or cell lines (see Supplementary Materials S2 for the IGV session). Each peak corresponds to an experiment archived in the NCBI SRA and processed through the unified ChIP-Atlas pipeline. Clicking on the indicated peak (HNF4A @ Hep G2) directs the user to the detailed experiment page for SRX10829255 (https://chip-atlas.org/view?id=SRX10829255), where all associated curated and original metadata and processing information are compiled.

The newly introduced Experiment Comparative Profile panel, located at the bottom of each detailed experiment page, provides quantitative quality metrics for individual experiments. The panel contains two plots that contextualize each dataset relative to all other experiments of the same assay type. The first plot, “Read and Peak Distribution,” shows where the selected experiment falls within the overall distribution of sequencing read counts and peak numbers across comparable experiments (i.e. ATAC-seq, DNase-seq, Bisulfite-seq, or ChIP-seq, with ChIP-seq further subdivided by antigen class, including TFs, histones, and RNA polymerase). In this plot, an orange horizontal line indicates the position of the selected experiment, allowing users to immediately assess whether it lies within the typical range. In Fig. 1B, the ChIP-seq experiment SRX10829255 (genome: hg38; antigen: HNF4A; cell type: Hep G2) lies near the mode of both read and peak distributions among ChIP-seq datasets targeting TF binding, indicating that its sequencing depth and peak yield are consistent with those of comparable experiments. The second plot, “Correlation-Based Clustering,” provides an orthogonal measure of data reproducibility. It calculates pairwise correlations between read alignment profiles for experiments sharing the same biological context, defined by the genome assembly, experiment type (e.g. ATAC-seq, DNase-seq, or Bisulfite-seq) or ChIP-seq antigen, and cell type. Hierarchical clustering is then applied to group experiments according to their overall signal similarity. Arrowheads indicate the selected experiment, with the color reflecting the median correlation coefficient with other experiments in the same cluster. In Fig. 1B, SRX10829255 shows a median correlation value of approximately 0.7–0.8 (orange), indicating a signal pattern highly comparable to other Hep G2 ChIP-seq experiments targeting HNF4A. Conversely, experiments with low median correlation values or isolated cluster positions may reflect distinct experimental conditions or potential technical variability (Supplementary Fig. S2). In addition to visualization, the underlying correlation data are available for download in TSV format, enabling users to identify related experiments for downstream comparative analyses.

These two quantitative indicators provide complementary perspectives, enabling users to assess the quality and representativeness of individual experiments within their broader assay context. In previous versions, reliability could only be inferred indirectly from metadata, processing logs (e.g. total read counts, alignment rate, duplicate rate, and number of peaks; see the Read Processing Pipeline panel in Fig. 1B), or visual intuition. In contrast, this update provides numerical and comparative metrics, allowing users to assess experiments across studies without leaving the interface or downloading additional data, thereby facilitating the selection of reliable datasets for downstream analyses.

### Example of use: PAGE-based enrichment analysis

**Figure 2.**
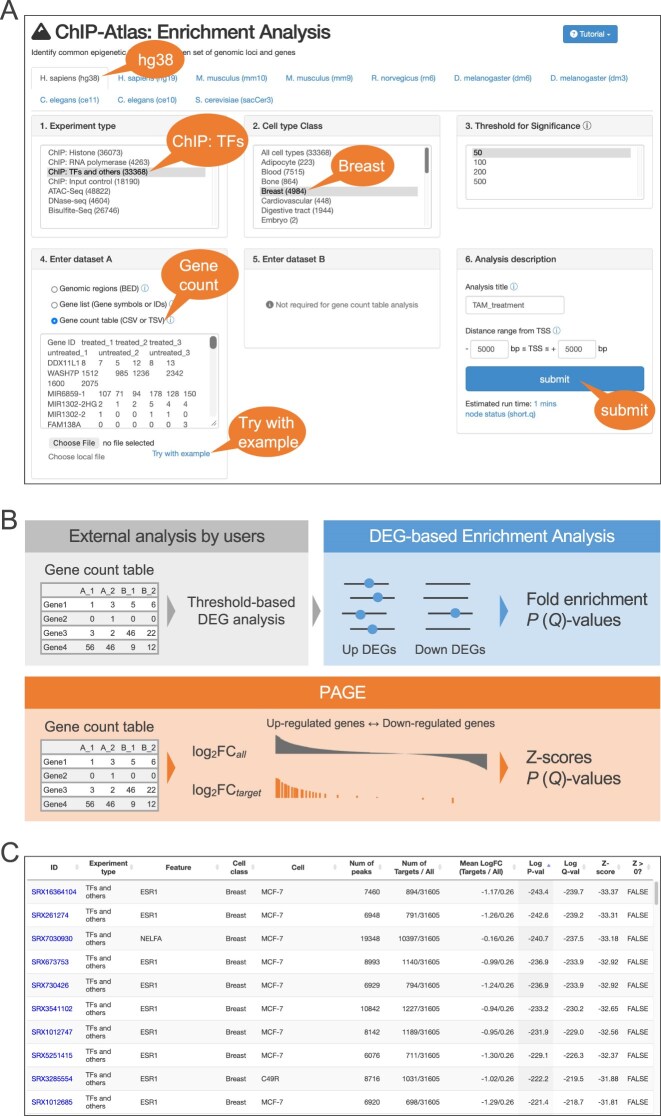
Use case of the PAGE module. (**A**) Parameter setting and dataset loading. (**B**) Schema of the DEG-based Enrichment Analysis and the PAGE-based method. (**C**) Result of PAGE analysis of an RNA-seq dataset from tamoxifen-treated and untreated MCF-7 cells. DEG, differentially expressed gene; FC, fold change.

In contrast to the DEG-based Enrichment Analysis tool, the newly implemented PAGE module in ChIP-Atlas 2025 allows analysis directly from continuous RNA-seq count tables, thereby eliminating the need for discrete DEG lists. Figure 2 illustrates the usage of PAGE for analyzing an RNA-seq dataset from tamoxifen-treated and untreated MCF-7 cells (derived from a reanalysis of GSE178303) to infer alterations in underlying gene regulatory mechanisms upon drug treatment.

To use PAGE, users first configure analysis parameters in the Enrichment Analysis interface (Fig. 2A). In this example, the genome assembly was set to hg38. To examine TF binding profiles, the track type was set to ChIP-seq: TFs and others. The cell type was designated as “Breast,” including MCF-7 cells, whereas the default “Threshold for Significance” was left unchanged. Selecting “Gene count table” in the “Enter dataset A” panel activates PAGE mode. After specifying these parameters, users can load their RNA-seq count table in CSV or TSV format. Here, a sample count table is provided for demonstration. Clicking “Try with example” automatically loads a TSV table obtained from RNA-seq experiments comparing tamoxifen-treated and untreated MCF-7 cells. The first column contains gene symbols, and each subsequent column represents a sample. Column headers indicate the condition and replicate number, separated by an underscore (e.g. treated_1, untreated_2). By default, overlaps between ChIP-seq peaks and gene loci within ±5 kb of TSSs are evaluated, though this distance can be adjusted using the “Distance range from TSS” option in the “Analysis description” panel. After clicking Submit, the server begins computing using the selected parameters and dataset.

Unlike the DEG-based enrichment analysis tool, which requires DEGs as input and evaluates the overlap of gene lists (e.g. tamoxifen-treated- and untreated-specific genes) with all available ChIP-seq peaks, PAGE uses continuous read counts from all genes (Fig. 2B). The algorithm compares the mean log₂FC of the genes associated with each ChIP-seq dataset (target gene set) with the genome-wide mean log₂FC of all genes, yielding *Z*-scores that quantify transcriptional enrichment at the gene set level. Positive *Z*-scores indicate enrichment under conditions of tamoxifen treatment, whereas negative scores indicate enrichment under no treatment. This approach detects subtle, coordinated transcriptional changes that threshold-dependent DEG analysis might miss while producing results compatible with conventional downstream workflows.

After several minutes of computation, results are returned in HTML and TSV formats (Fig. 2C and Supplementary Materials S3 and S4). The HTML output contains the same information as the DEG-based enrichment analysis results: SRX identifiers, epigenomic features (e.g. ChIP antigens), and cell types. In addition, it displays PAGE-specific metrics, including the number of target genes overlapping ChIP-seq peaks within the TSS ±5 kb window, the ratio of the mean log₂FC of target genes to that of all genes, *Z*-scores, and corresponding log_10_*P*- and *Q*-values. By default, the table is sorted by ascending log_10_*P*, facilitating identification of the most significantly enriched features. In the tamoxifen example, multiple experiments targeting the estrogen receptor (ESR1) in MCF-7 cells dominated the top ranks, showing consistently negative *Z*-scores and small log_10_*P*-values (e.g. SRX16364104: log_10_*P* = –243.4; *Z* = –33.37). These results suggest that ESR1 target genes were preferentially downregulated in tamoxifen-treated samples, consistent with the function of tamoxifen as an ESR1 antagonist, which inhibits estrogen-dependent transcriptional activation and suppresses tumor cell proliferation [20]. This example demonstrates that PAGE can accurately capture biologically significant transcriptional changes reflecting drug action. The SRX identifiers in the results table are hyperlinked to their corresponding experiment pages, enabling users to navigate directly to the Experiment Comparative Profile, in addition to the Peak Browser route shown in Fig. 1. This linkage supports immediate reliability checks and confident interpretation of enrichment results. To validate PAGE results, we analyzed the same RNA-seq dataset using conventional DEG-based enrichment analysis. DEGs identified using iDEP [19] produced comparable enrichment of ESR1-related experiments in MCF-7 cells (Supplementary Fig. S3 and Supplementary Materials S5 and S6). For programmatic batch processing, an application programming interface (API) is also available for PAGE, similar to that provided for the DEG-based module. Comprehensive API documentation is available at https://github.com/inutano/chip-atlas/wiki/Perform-Enrichment-Analysis-programmatically.

## Discussion

Herein, we present the 2025 update to ChIP-Atlas, featuring two significant advancements: an experiment-level quality control framework and a PAGE-based enrichment analysis module. The quality control framework enables users to efficiently evaluate the reliability and representativeness of individual experiments before using them in downstream analyses. The PAGE module allows gene set-oriented enrichment to be performed directly from continuous RNA-seq data, capturing coordinated expression shifts while avoiding the arbitrary cutoffs and information loss inherent to DEG-based workflows. Together, these developments substantially enhance both the analytical reliability and flexibility of the ChIP-Atlas platform.

The quality-control framework introduced in this update is designed to provide complementary perspectives rather than a single measure of data quality. Metrics such as read counts and peak numbers depend strongly on the profiled factor and experimental context and therefore cannot be interpreted solely. Similarly, correlation-based clustering reflects similarity in genome-wide signal patterns but does not directly indicate data quality, as technically biased datasets may show high correlation if they share similar artifacts (e.g. low antibody specificity). Taken together, these considerations indicate that no single metric is sufficient, and data quality is more reliably assessed by integrating processing-level metrics with relative, context-dependent comparisons.

Although PAGE is advantageous for detecting subtle yet coordinated changes, the DEG-based enrichment analysis remains a robust approach for evaluating sharply defined expression differences based on predefined DEGs (Table 2). PAGE is sensitive to modest but coherent regulatory shifts that DEG thresholds may overlook. However, very small expression changes may reflect noise, and thus careful interpretation is required. In contrast, the DEG-based method focuses on statistically significant DEGs, which are robust to noise despite potential thresholding biases. For these reasons, both analyses are provided in parallel on ChIP-Atlas, sharing a unified interface and output format to facilitate comparison and integration.

**Table 2.** Comparison between DEG-based and PAGE-enrichment analyses

Feature	DEG-based enrichment analysis	PAGE-based enrichment analysis
Input	List of DEGs	RNA-seq count table (all genes)
Data usage	Subset of genes (threshold-dependent)	All genes (continuous values)
Strength	Robust to noise; easy to interpret	Detects subtle, coordinated expression changes
Limitation	Sensitive to threshold choice; may miss modest effects	Sensitive to noise; requires careful interpretation
Best use case	Clear differential expression with sufficient DEGs	Weak or diffuse transcriptional changes; small effect sizes
Typical scenario	Strong perturbation (e.g. knockout)	Subtle regulation (e.g. drug response and early time points)

Unlike other public epigenomic resources such as GTRD [21] and ReMap [22], in which limited recent updates are available, ChIP-Atlas continues to be actively maintained through routine data integration, incremental expansion of metadata, and iterative refinement of its analytical framework. This sustained maintenance is essential for adapting to the rapid accumulation of epigenomic profiling data and for preserving the utility of the resource as an entry point to public epigenomic archives.

As ChIP-Atlas enters its second decade, several future directions emerge from current limitations. First, the coverage of the platform remains biased toward a subset of model organisms and assay types. Extending to additional species, including plants and non-model organisms, as well as emerging experimental modalities such as CUT&Tag [23], ChIL-seq [24], and spatial epigenomics technologies, will broaden the range of addressable biological questions. Second, although the current metadata curation framework incorporates machine learning-based classification, much experimental context, such as gene knockout or overexpression, drug treatment, and disease status, remains incompletely captured. Refinement of metadata annotation using large language model-based extraction and classification systems currently under evaluation will address this gap [25]. Third, despite the rapidly increasing number of experiments, many modality-tissue/cell type combinations remain underrepresented or absent, limiting comprehensive reconstruction of the gene-regulatory landscape. Recent advancements in cross-cell-type modeling leveraging shared representations of regulatory states suggest that imputing these missing contexts is now feasible [26]. Consequently, future efforts will focus on implementing artificial intelligence algorithms for predicting and completing sparse modality–cell type matrices, enabling a more systematic investigation of regulatory patterns in undersampled contexts.

## Supplementary Material

gkag378_Supplemental_Files

## Data Availability

ChIP-Atlas (https://chip-atlas.org) is an open-source web server. All codes and libraries are available in the ChIP-Atlas GitHub repository (https://github.com/inutano/chip-atlas/tree/v4.0.1) and Zenodo [15]. This website is free and open to all users and there is no login requirement.
