# Supplementary material for: ChIP-Atlas 2025 update: 10-year anniversary of a data-mining platform for exploring epigenomic landscape
Source: Nucleic Acids Res. 2026 Apr 29;54(W1):W77–84. doi: 10.1093/nar/gkag378 (PMC13355075; doi:10.1093/nar/gkag378)
Supplement: gkag378_Supplemental_Files [file gkag378_supplemental_files.zip › Supplementary_Figure_with_Legends.pdf]

Supplementary Figure 1. Visualization of cis-regulatory elements (CAGE-derived bidirectionally transcribed enhancers) from fanta.bio using the Peak Browser. (A) Parameter settings for loading the CAGE: Enhancer dataset in the Peak Browser query page prior to visualization in IGV. (B) The CAGE: Enhancer track displayed in IGV alongside histone modification and transcription factor binding tracks in human macrophages. Bars in the "CAGE: Enhancer" track represent cell–type–specific cis-regulatory regions, with colors indicating BED scores (blue, green, and red correspond to 50, 500, and ≥1,000, respectively). Details of the BED score calculation are described in the Materials and Methods (Transcribed enhancers and promoters dataset). The IGV session file for panel (B) is provided as Supplementary Material 1.

Supplementary Figure 2. Example of an experiment (SRX15717769: https://chip-atlas.org/view?id=SRX15717769) from the same biological context as Figure 1 but showing lower consistency in the quality-control framework. (A) Detailed page of SRX15717769. The read and peak distribution plots show that the experiment deviates from the typical range of comparable datasets. The correlation-based clustering (see Figure 1 for details) indicates that the selected experiment (arrowheads) exhibits lower similarity to other experiments and is positioned apart from the main cluster, suggesting reduced consistency in genome-wide signal patterns. (B) Correlation matrix. The underlying correlation data (TSV) can be downloaded using the "Download" button below the heatmap and explored in spreadsheet software with color scaling to identify related experiments within the same biological context.

Supplementary Figure 3. Settings and results of the DEG-based Enrichment Analysis using differentially expressed genes specific to tamoxifen-treated versus untreated MCF-7 cells. (A) Parameter setting and dataset loading. (B) DEG-based enrichment analysis result.

Supplementary Material 1. IGV session file for Supplementary Figure 1B.

Supplementary Material 2. IGV session file for Figure 1A.

Supplementary Material 3. Full PAGE results in HTML format corresponding to Figure 2C.

Supplementary Material 4. Full PAGE results in TSV format corresponding to Figure 2C.

Supplementary Material 5. Full DEG-based Enrichment Analysis results in HTML format corresponding to Supplementary Figure 3B.

Supplementary Material 6. Full DEG-based Enrichment Analysis results in TSV format corresponding to Supplementary Figure 3B.

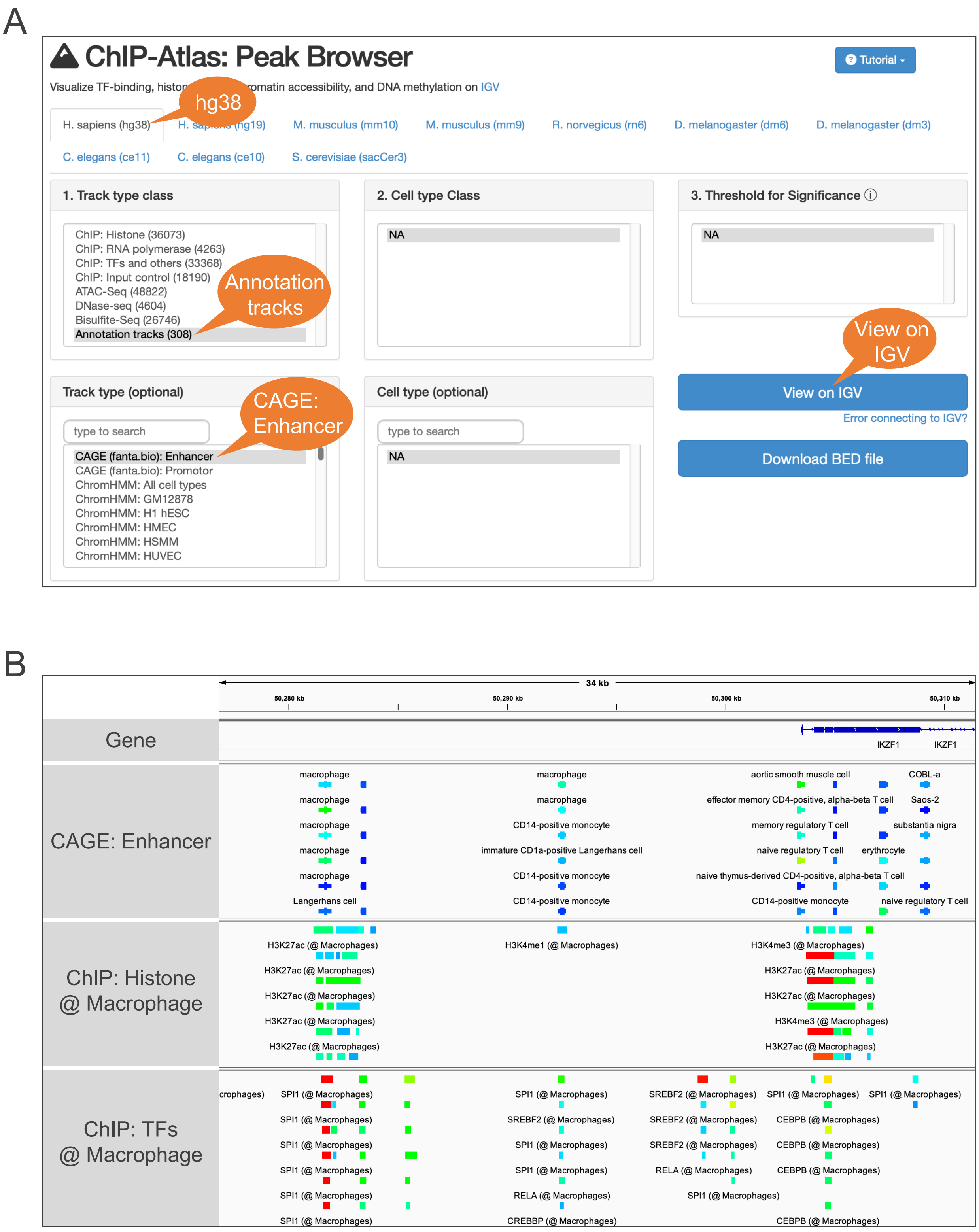

Supplementary Figure 1

A

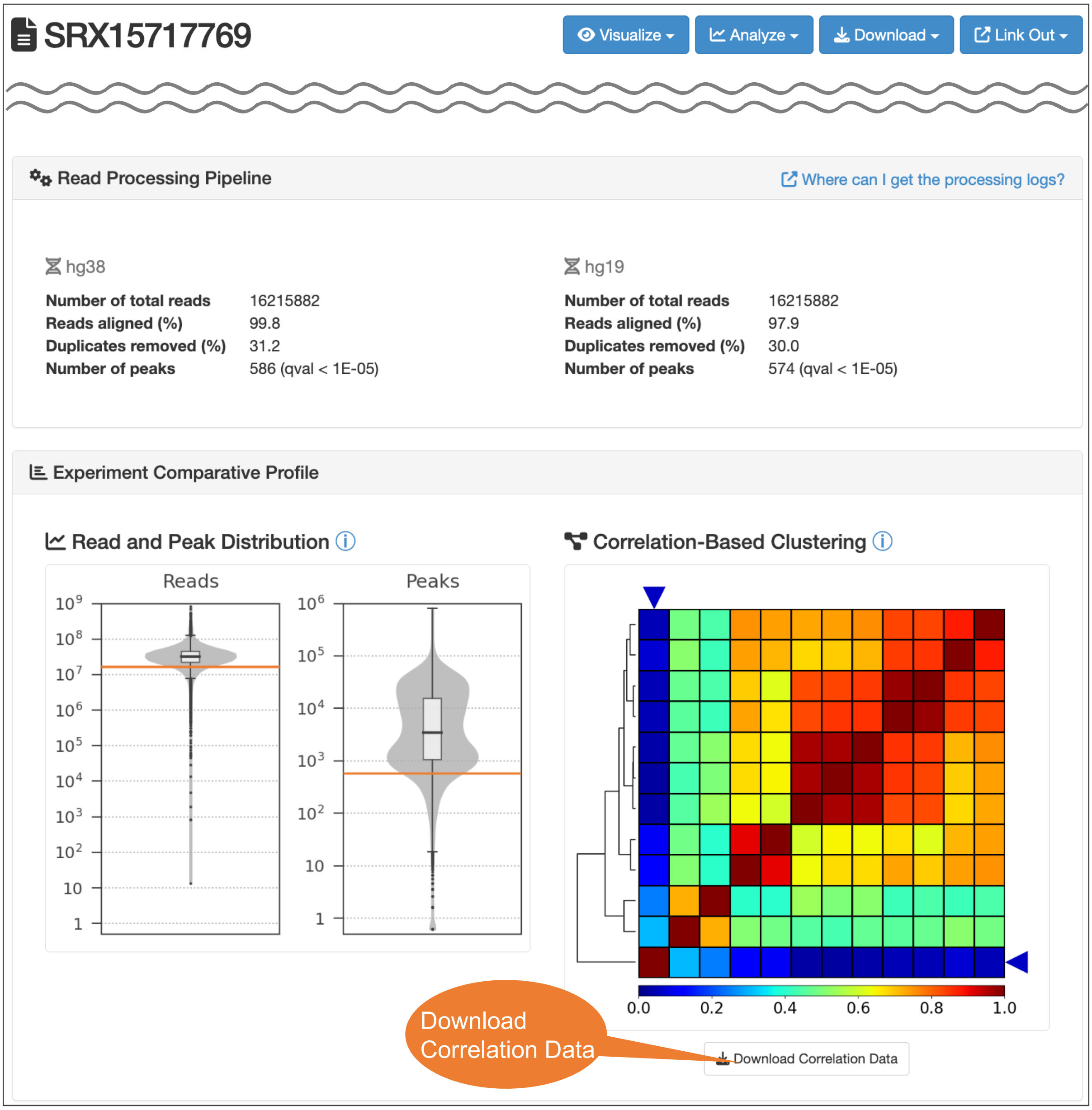

B

	'SRX15717769'	'SRX15717766'	'SRX15717767'	'SRX018625'	'SRX018626'	'SRX10829253'	'SRX10829254'	'SRX10829255'	'SRX10475577'	'SRX10475578'	'SRX100505'	'SRX150698'
'SRX150698'	0.0493	0.4917	0.4444	0.7545	0.741	0.7309	0.7386	0.7598	0.839	0.837	0.8797	1
'SRX100505'	0.0741	0.516	0.4233	0.7407	0.7151	0.6808	0.6916	0.717	0.8451	0.8543	1	0.8797
'SRX10475578'	0.0475	0.4752	0.4431	0.6938	0.6208	0.8314	0.839	0.8503	0.977	1	0.8543	0.837
'SRX10475577'	0.0477	0.4574	0.4253	0.737	0.6698	0.8351	0.8536	0.8577	1	0.977	0.8451	0.839
'SRX10829255'	0.0387	0.4694	0.5171	0.6788	0.6439	0.9618	0.9647	1	0.8577	0.8503	0.717	0.7598
'SRX10829254'	0.03	0.4361	0.5014	0.6788	0.6494	0.9629	1	0.9647	0.8536	0.839	0.6916	0.7386
'SRX10829253'	0.0313	0.4535	0.5396	0.6411	0.6036	1	0.9629	0.9618	0.8351	0.8314	0.6808	0.7309
'SRX018626'	0.1021	0.4859	0.4022	0.9123	1	0.6036	0.6494	0.6439	0.6698	0.6208	0.7151	0.741
'SRX018625'	0.1164	0.5084	0.4052	1	0.9123	0.6411	0.6788	0.6788	0.737	0.6938	0.7407	0.7545
'SRX15717767'	0.2494	0.7253	1	0.4052	0.4022	0.5396	0.5014	0.5171	0.4253	0.4431	0.4233	0.4444
'SRX15717766'	0.3053	1	0.7253	0.5084	0.4859	0.4535	0.4361	0.4694	0.4574	0.4752	0.516	0.4917
SRX15717769'	1	0.3053	0.2494	0.1164	0.1021	0.0313	0.03	0.0387	0.0477	0.0475	0.0741	0.0493

Supplementary Figure 2

A

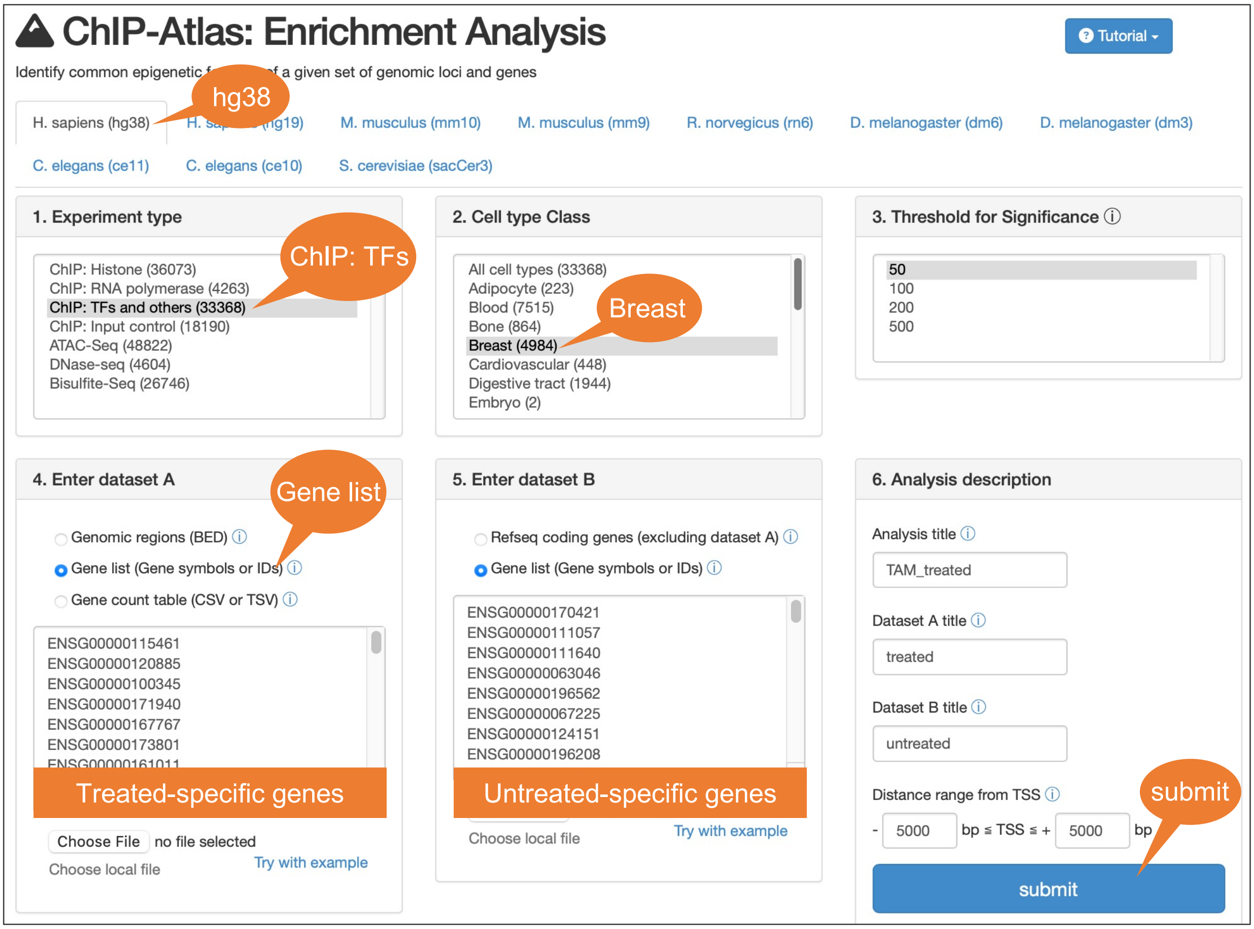

B

ID	Experiment type	Feature	Cell class	Cell	Num of peaks	Overlaps / treated	Overlaps / untreated	Log P-val	Log Q-val	Fold Enrichment	FE > 1?
SRX10971491	TFs and others	ESR1	Breast	MCF-7	10391	60/1008	634/1457	-105.2	-101.6	0.14	FALSE
SRX10971500	TFs and others	ESR1	Breast	MCF-7	9336	57/1008	621/1457	-103.8	-100.4	0.13	FALSE
SRX673753	TFs and others	ESR1	Breast	MCF-7	8993	34/1008	547/1457	-102.3	-99.1	0.09	FALSE
SRX7030930	TFs and others	NELFA	Breast	MCF-7	19348	331/1008	1099/1457	-99.5	-96.4	0.44	FALSE
SRX4417022	TFs and others	ESR1	Breast	MCF-7	14573	89/1008	684/1457	-98.8	-95.9	0.19	FALSE
SRX1012747	TFs and others	ESR1	Breast	MCF-7	8142	48/1008	570/1457	-96.6	-93.7	0.12	FALSE
SRX2922231	TFs and others	ESR1	Breast	SUM 44PE	29836	222/1008	919/1457	-92.8	-89.9	0.35	FALSE
SRX3541102	TFs and others	ESR1	Breast	MCF-7	10842	69/1008	610/1457	-91.7	-88.9	0.16	FALSE
SRX16364104	TFs and others	ESR1	Breast	MCF-7	7460	33/1008	506/1457	-91.4	-88.7	0.09	FALSE
SRX2871338	TFs and others	ESR1	Breast	T-47D	10650	74/1008	613/1457	-89.1	-86.4	0.17	FALSE

Supplementary Figure 3
