# Supplementary material for: ChIP-Atlas 2025 update: 10-year anniversary of a data-mining platform for exploring epigenomic landscape
Source: Nucleic Acids Res. 2026 Apr 29;54(W1):W77–84. doi: 10.1093/nar/gkag378 (PMC13355075; doi:10.1093/nar/gkag378)
Supplement: gkag378_Supplemental_Files [file gkag378_supplemental_files.zip › supplementary_materials.zip!/supplementary_materials/Supplementary_Material_S3_Fig2C.html]

TAM\_treatment


# ChIP-Atlas / Enrichment Analysis

Search for epigenomic features significantly bound to your data.

## TAM\_treatment

| ID | Experiment type | Feature | Cell class | Cell | Num of peaks | Num of Targets / All | Mean LogFC (Targets / All) | Log P-val | Log Q-val | Z-score | Z > 0? |
| --- | --- | --- | --- | --- | --- | --- | --- | --- | --- | --- | --- |
| SRX16364104 | TFs and others | ESR1 | Breast | MCF-7 | 7460 | 894/31605 | -1.17/0.26 | -243.4 | -239.7 | -33.37 | FALSE |
| SRX261274 | TFs and others | ESR1 | Breast | MCF-7 | 6948 | 791/31605 | -1.26/0.26 | -242.6 | -239.2 | -33.31 | FALSE |
| SRX7030930 | TFs and others | NELFA | Breast | MCF-7 | 19348 | 10397/31605 | -0.16/0.26 | -240.7 | -237.5 | -33.18 | FALSE |
| SRX673753 | TFs and others | ESR1 | Breast | MCF-7 | 8993 | 1140/31605 | -0.99/0.26 | -236.9 | -233.9 | -32.92 | FALSE |
| SRX730426 | TFs and others | ESR1 | Breast | MCF-7 | 6929 | 794/31605 | -1.24/0.26 | -236.9 | -233.9 | -32.92 | FALSE |
| SRX3541102 | TFs and others | ESR1 | Breast | MCF-7 | 10842 | 1227/31605 | -0.94/0.26 | -233.2 | -230.2 | -32.65 | FALSE |
| SRX1012747 | TFs and others | ESR1 | Breast | MCF-7 | 8142 | 1189/31605 | -0.95/0.26 | -231.9 | -229.0 | -32.56 | FALSE |
| SRX5251415 | TFs and others | ESR1 | Breast | MCF-7 | 6076 | 711/31605 | -1.30/0.26 | -229.1 | -226.3 | -32.37 | FALSE |
| SRX3285554 | TFs and others | ESR1 | Breast | C49R | 8716 | 1031/31605 | -1.02/0.26 | -222.2 | -219.5 | -31.88 | FALSE |
| SRX1012685 | TFs and others | ESR1 | Breast | MCF-7 | 6920 | 698/31605 | -1.29/0.26 | -221.4 | -218.7 | -31.81 | FALSE |
| SRX3541101 | TFs and others | ESR1 | Breast | MCF-7 | 4980 | 553/31605 | -1.47/0.26 | -219.4 | -216.8 | -31.67 | FALSE |
| SRX10971500 | TFs and others | ESR1 | Breast | MCF-7 | 9336 | 1606/31605 | -0.75/0.26 | -218.4 | -215.8 | -31.60 | FALSE |
| SRX7030912 | TFs and others | HEXIM1 | Breast | MCF-7 | 20058 | 9955/31605 | -0.15/0.26 | -217.8 | -215.3 | -31.56 | FALSE |
| SRX6757650 | TFs and others | ESR1 | Breast | MCF-7 | 6719 | 828/31605 | -1.15/0.26 | -217.3 | -214.8 | -31.52 | FALSE |
| SRX5511291 | TFs and others | ESR1 | Breast | MCF-7 | 4494 | 447/31605 | -1.65/0.26 | -215.4 | -212.9 | -31.38 | FALSE |
| SRX1012679 | TFs and others | ESR1 | Breast | MCF-7 | 6755 | 647/31605 | -1.32/0.26 | -214.7 | -212.2 | -31.33 | FALSE |
| SRX1012750 | TFs and others | ESR1 | Breast | MCF-7 | 6049 | 878/31605 | -1.10/0.26 | -214.6 | -212.2 | -31.32 | FALSE |
| SRX673741 | TFs and others | ESR1 | Breast | MCF-7 | 3446 | 439/31605 | -1.66/0.26 | -214.4 | -211.9 | -31.30 | FALSE |
| SRX3583179 | TFs and others | ESR1 | Breast | MCF-7 | 7775 | 726/31605 | -1.23/0.26 | -213.0 | -210.6 | -31.20 | FALSE |
| SRX6712618 | TFs and others | RAD21 | Breast | MCF-7 | 14029 | 10620/31605 | -0.13/0.26 | -212.1 | -209.7 | -31.14 | FALSE |
| SRX11120886 | TFs and others | TAF1 | Breast | MCF-7 | 19681 | 11271/31605 | -0.12/0.26 | -211.9 | -209.6 | -31.12 | FALSE |
| SRX5511293 | TFs and others | ESR1 | Breast | MCF-7 | 5203 | 588/31605 | -1.38/0.26 | -209.5 | -207.2 | -30.95 | FALSE |
| SRX10971491 | TFs and others | ESR1 | Breast | MCF-7 | 10391 | 1697/31605 | -0.70/0.26 | -208.5 | -206.2 | -30.87 | FALSE |
| SRX5511292 | TFs and others | ESR1 | Breast | MCF-7 | 4020 | 442/31605 | -1.62/0.26 | -208.3 | -206.0 | -30.85 | FALSE |
| SRX730451 | TFs and others | ESR1 | Breast | MCF-7 | 6804 | 952/31605 | -1.02/0.26 | -207.8 | -205.5 | -30.82 | FALSE |
| SRX5089557 | TFs and others | BRD4 | Breast | MCF-7 | 22814 | 10702/31605 | -0.13/0.26 | -206.9 | -204.7 | -30.75 | FALSE |
| SRX3629604 | TFs and others | ESR1 | Breast | MCF-7 | 6924 | 710/31605 | -1.22/0.26 | -206.5 | -204.2 | -30.72 | FALSE |
| SRX3123818 | TFs and others | ESR1 | Breast | MCF-7 | 9203 | 835/31605 | -1.11/0.26 | -206.3 | -204.1 | -30.70 | FALSE |
| SRX3583187 | TFs and others | ESR1 | Breast | MCF-7 | 7938 | 639/31605 | -1.30/0.26 | -206.2 | -203.9 | -30.69 | FALSE |
| SRX7030928 | TFs and others | NELFA | Breast | MCF-7 | 17038 | 11278/31605 | -0.11/0.26 | -205.3 | -203.1 | -30.63 | FALSE |
| SRX107370 | TFs and others | ESR1 | Breast | H3396 | 6940 | 695/31605 | -1.23/0.26 | -205.0 | -202.8 | -30.60 | FALSE |
| SRX11120885 | TFs and others | TAF1 | Breast | MCF-7 | 18036 | 11163/31605 | -0.12/0.26 | -204.2 | -202.0 | -30.54 | FALSE |
| SRX4417022 | TFs and others | ESR1 | Breast | MCF-7 | 14573 | 2063/31605 | -0.61/0.26 | -203.7 | -201.5 | -30.51 | FALSE |
| SRX1733713 | TFs and others | ESR1 | Breast | MCF-7 | 3861 | 765/31605 | -1.16/0.26 | -203.6 | -201.4 | -30.50 | FALSE |
| SRX1336216 | TFs and others | ESR1 | Breast | T-47D | 8061 | 803/31605 | -1.12/0.26 | -203.5 | -201.3 | -30.49 | FALSE |
| SRX3285559 | TFs and others | ESR1 | Breast | MCF-7 | 10867 | 1145/31605 | -0.90/0.26 | -203.3 | -201.1 | -30.48 | FALSE |
| SRX6757657 | TFs and others | ESR1 | Breast | MCF-7 | 4531 | 472/31605 | -1.54/0.26 | -203.2 | -201.1 | -30.47 | FALSE |
| SRX5511297 | TFs and others | ESR1 | Breast | MCF-7 | 3982 | 435/31605 | -1.62/0.26 | -203.2 | -201.1 | -30.47 | FALSE |
| SRX968971 | TFs and others | RELA | Breast | MCF-7 | 11903 | 1167/31605 | -0.89/0.26 | -202.5 | -200.4 | -30.42 | FALSE |
| SRX2424161 | TFs and others | SIN3A | Breast | MCF-7 | 17916 | 9628/31605 | -0.14/0.26 | -202.3 | -200.2 | -30.40 | FALSE |
| SRX8617021 | TFs and others | ESR1 | Breast | MCF-7 | 9202 | 1268/31605 | -0.84/0.26 | -201.5 | -199.4 | -30.34 | FALSE |
| SRX7692443 | TFs and others | POLR2C | Breast | MCF-7 | 16630 | 10248/31605 | -0.13/0.26 | -200.5 | -198.5 | -30.27 | FALSE |
| SRX5511298 | TFs and others | ESR1 | Breast | MCF-7 | 3817 | 400/31605 | -1.68/0.26 | -200.5 | -198.4 | -30.27 | FALSE |
| SRX4786539 | TFs and others | ESR1 | Breast | MCF-7 | 8870 | 1489/31605 | -0.75/0.26 | -200.4 | -198.4 | -30.26 | FALSE |
| SRX1012687 | TFs and others | ESR1 | Breast | MCF-7 | 6872 | 620/31605 | -1.30/0.26 | -198.9 | -196.9 | -30.15 | FALSE |
| SRX10365547 | TFs and others | NCOA3 | Breast | MCF-7 | 7807 | 752/31605 | -1.15/0.26 | -198.9 | -196.8 | -30.14 | FALSE |
| SRX2922225 | TFs and others | ESR1 | Breast | MCF-7 | 14045 | 1526/31605 | -0.73/0.26 | -198.6 | -196.5 | -30.12 | FALSE |
| SRX1012749 | TFs and others | ESR1 | Breast | MCF-7 | 4229 | 570/31605 | -1.36/0.26 | -198.6 | -196.5 | -30.12 | FALSE |
| SRX4786543 | TFs and others | ESR1 | Breast | MCF-7 | 6339 | 1068/31605 | -0.92/0.26 | -198.1 | -196.1 | -30.08 | FALSE |
| SRX7692444 | TFs and others | POLR2C | Breast | MCF-7 | 16551 | 10184/31605 | -0.13/0.26 | -197.9 | -195.9 | -30.07 | FALSE |
| SRX5511296 | TFs and others | ESR1 | Breast | MCF-7 | 3721 | 387/31605 | -1.70/0.26 | -197.7 | -195.8 | -30.06 | FALSE |
| SRX3541110 | TFs and others | ESR1 | Breast | MCF-7 | 5578 | 551/31605 | -1.38/0.26 | -197.2 | -195.2 | -30.02 | FALSE |
| SRX7540325 | TFs and others | BRD4 | Breast | MCF-7 | 19707 | 10455/31605 | -0.12/0.26 | -196.5 | -194.5 | -29.96 | FALSE |
| SRX3541097 | TFs and others | ESR1 | Breast | MCF-7 | 6120 | 697/31605 | -1.20/0.26 | -196.5 | -194.5 | -29.96 | FALSE |
| SRX3123811 | TFs and others | ESR1 | Breast | MCF-7 | 9099 | 784/31605 | -1.11/0.26 | -196.4 | -194.5 | -29.95 | FALSE |
| SRX3123812 | TFs and others | ESR1 | Breast | MCF-7 | 10503 | 929/31605 | -1.00/0.26 | -196.1 | -194.2 | -29.93 | FALSE |
| SRX9424463 | TFs and others | RUVBL2 | Breast | MCF-7 | 20111 | 10435/31605 | -0.12/0.26 | -196.0 | -194.1 | -29.93 | FALSE |
| SRX730436 | TFs and others | NCAPG2 | Breast | MCF-7 | 5105 | 569/31605 | -1.35/0.26 | -196.0 | -194.1 | -29.92 | FALSE |
| SRX673742 | TFs and others | ESR1 | Breast | MCF-7 | 4126 | 449/31605 | -1.55/0.26 | -195.7 | -193.8 | -29.90 | FALSE |
| SRX1585244 | TFs and others | ESR1 | Breast | LTED cells | 9845 | 1270/31605 | -0.82/0.26 | -195.6 | -193.7 | -29.89 | FALSE |
| SRX3629597 | TFs and others | ESR1 | Breast | MCF-7 | 5539 | 599/31605 | -1.31/0.26 | -195.6 | -193.7 | -29.89 | FALSE |
| SRX1012743 | TFs and others | ESR1 | Breast | MCF-7 | 3169 | 412/31605 | -1.63/0.26 | -195.1 | -193.2 | -29.85 | FALSE |
| SRX7030932 | TFs and others | NELFA | Breast | MCF-7 | 10685 | 8000/31605 | -0.17/0.26 | -195.0 | -193.1 | -29.84 | FALSE |
| SRX4115993 | TFs and others | ESR1 | Breast | MCF-7 | 8060 | 1333/31605 | -0.79/0.26 | -194.5 | -192.6 | -29.81 | FALSE |
| SRX4451201 | TFs and others | ESR1 | Breast | MCF-7 | 6003 | 533/31605 | -1.40/0.26 | -194.5 | -192.6 | -29.81 | FALSE |
| SRX8617018 | TFs and others | TET2 | Breast | MCF-7 | 5763 | 660/31605 | -1.23/0.26 | -194.3 | -192.5 | -29.79 | FALSE |
| SRX5534851 | TFs and others | EP300 | Breast | MCF-7 | 6577 | 1026/31605 | -0.94/0.26 | -194.2 | -192.3 | -29.78 | FALSE |
| SRX4193150 | TFs and others | ESR1 | Breast | MCF-7 | 10581 | 3219/31605 | -0.42/0.26 | -193.5 | -191.6 | -29.73 | FALSE |
| SRX9424462 | TFs and others | RUVBL2 | Breast | MCF-7 | 18208 | 10017/31605 | -0.12/0.26 | -193.2 | -191.3 | -29.70 | FALSE |
| SRX2145563 | TFs and others | ESR1 | Breast | MCF-7 | 11743 | 1279/31605 | -0.81/0.26 | -192.8 | -190.9 | -29.67 | FALSE |
| SRX16364105 | TFs and others | ESR1 | Breast | MCF-7 | 5380 | 814/31605 | -1.08/0.26 | -192.7 | -190.9 | -29.67 | FALSE |
| SRX11169864 | TFs and others | ESR1 | Breast | MCF-7 | 10533 | 3225/31605 | -0.41/0.26 | -192.5 | -190.7 | -29.65 | FALSE |
| SRX7540322 | TFs and others | ESR1 | Breast | MCF-7 | 5741 | 670/31605 | -1.21/0.26 | -192.4 | -190.6 | -29.65 | FALSE |
| SRX2424162 | TFs and others | SIN3A | Breast | MCF-7 | 14215 | 9362/31605 | -0.14/0.26 | -192.3 | -190.5 | -29.64 | FALSE |
| SRX19662626 | TFs and others | JUN | Breast | T-47D | 20658 | 10792/31605 | -0.11/0.26 | -191.5 | -189.7 | -29.58 | FALSE |
| SRX6777396 | TFs and others | ESR1 | Breast | MCF-7 | 3522 | 347/31605 | -1.77/0.26 | -190.9 | -189.1 | -29.53 | FALSE |
| SRX3629600 | TFs and others | ESR1 | Breast | MCF-7 | 4363 | 472/31605 | -1.49/0.26 | -190.9 | -189.1 | -29.53 | FALSE |
| SRX2922233 | TFs and others | ESR1 | Breast | SUM 44PE | 44750 | 9712/31605 | -0.13/0.26 | -189.7 | -187.9 | -29.43 | FALSE |
| SRX5287696 | TFs and others | ESR1 | Breast | MCF-7 | 14784 | 1786/31605 | -0.64/0.26 | -189.6 | -187.8 | -29.42 | FALSE |
| SRX2922231 | TFs and others | ESR1 | Breast | SUM 44PE | 29836 | 5486/31605 | -0.25/0.26 | -188.5 | -186.7 | -29.34 | FALSE |
| SRX1012681 | TFs and others | ESR1 | Breast | MCF-7 | 9832 | 881/31605 | -1.01/0.26 | -187.8 | -186.0 | -29.28 | FALSE |
| SRX3285555 | TFs and others | ESR1 | Breast | C70R | 6667 | 878/31605 | -1.01/0.26 | -187.5 | -185.8 | -29.26 | FALSE |
| SRX1012739 | TFs and others | ESR1 | Breast | MCF-7 | 3400 | 530/31605 | -1.37/0.26 | -187.0 | -185.2 | -29.22 | FALSE |
| SRX396818 | TFs and others | ESR1 | Breast | MCF-7 | 4768 | 390/31605 | -1.64/0.26 | -186.9 | -185.1 | -29.21 | FALSE |
| SRX20282965 | TFs and others | ESR1 | Breast | MCF-7 | 6051 | 600/31605 | -1.27/0.26 | -186.6 | -184.8 | -29.19 | FALSE |
| SRX14101925 | TFs and others | NELFE | Breast | MCF-7 | 13411 | 9080/31605 | -0.14/0.26 | -186.6 | -184.8 | -29.19 | FALSE |
| SRX3629599 | TFs and others | ESR1 | Breast | MCF-7 | 8761 | 914/31605 | -0.98/0.26 | -186.0 | -184.2 | -29.14 | FALSE |
| SRX3110724 | TFs and others | ESR1 | Breast | MCF-7 | 15627 | 1986/31605 | -0.58/0.26 | -185.6 | -183.9 | -29.11 | FALSE |
| SRX8080561 | TFs and others | Epitope tags | Breast | Mammary gland adenocarcinoma | 12909 | 1295/31605 | -0.78/0.26 | -185.2 | -183.5 | -29.08 | FALSE |
| SRX1012745 | TFs and others | ESR1 | Breast | MCF-7 | 3381 | 402/31605 | -1.60/0.26 | -185.2 | -183.4 | -29.08 | FALSE |
| SRX2880999 | TFs and others | ESR1 | Breast | MCF-7 | 12375 | 2026/31605 | -0.57/0.26 | -185.1 | -183.3 | -29.07 | FALSE |
| SRX099866 | TFs and others | MYC | Breast | MCF-7 | 27213 | 9300/31605 | -0.13/0.26 | -184.9 | -183.2 | -29.06 | FALSE |
| SRX8616981 | TFs and others | ESR1 | Breast | ZR-75-1 | 5322 | 656/31605 | -1.20/0.26 | -184.8 | -183.1 | -29.05 | FALSE |
| SRX5824589 | TFs and others | BRD2 | Breast | SUM 159PT | 11697 | 8460/31605 | -0.15/0.26 | -184.7 | -183.0 | -29.04 | FALSE |
| SRX3123816 | TFs and others | ESR1 | Breast | MCF-7 | 12003 | 1174/31605 | -0.83/0.26 | -184.7 | -182.9 | -29.04 | FALSE |
| SRX3110716 | TFs and others | ESR1 | Breast | MCF-7 | 7200 | 900/31605 | -0.98/0.26 | -184.6 | -182.9 | -29.03 | FALSE |
| SRX9932053 | TFs and others | STAT5A | Breast | MCF-7 | 21483 | 9140/31605 | -0.13/0.26 | -184.4 | -182.7 | -29.02 | FALSE |
| SRX3110710 | TFs and others | ESR1 | Breast | MCF-7 | 5522 | 766/31605 | -1.09/0.26 | -184.3 | -182.6 | -29.01 | FALSE |
| SRX5089460 | TFs and others | ESR1 | Breast | MCF-7 | 7611 | 1392/31605 | -0.74/0.26 | -184.1 | -182.4 | -28.99 | FALSE |
| SRX190294 | TFs and others | PML | Breast | MCF-7 | 13834 | 8239/31605 | -0.15/0.26 | -183.9 | -182.2 | -28.97 | FALSE |
| SRX3629602 | TFs and others | ESR1 | Breast | MCF-7 | 9438 | 996/31605 | -0.92/0.26 | -183.7 | -182.0 | -28.96 | FALSE |
| SRX5824561 | TFs and others | BRD2 | Breast | SUM 149PT | 20761 | 10458/31605 | -0.11/0.26 | -183.5 | -181.8 | -28.95 | FALSE |
| SRX8616962 | TFs and others | ESR1 | Breast | MCF-7 | 6794 | 796/31605 | -1.06/0.26 | -183.4 | -181.7 | -28.94 | FALSE |
| SRX3322264 | TFs and others | ELF1 | Breast | MCF-7 | 17636 | 8660/31605 | -0.14/0.26 | -183.0 | -181.4 | -28.91 | FALSE |
| SRX2871338 | TFs and others | ESR1 | Breast | T-47D | 10650 | 2047/31605 | -0.56/0.26 | -182.6 | -180.9 | -28.88 | FALSE |
| SRX190318 | TFs and others | SIN3A | Breast | MCF-7 | 43761 | 10937/31605 | -0.10/0.26 | -182.5 | -180.9 | -28.87 | FALSE |
| SRX054572 | TFs and others | Epitope tags | Breast | MCF-7 | 11165 | 6754/31605 | -0.19/0.26 | -182.3 | -180.7 | -28.85 | FALSE |
| SRX734309 | TFs and others | E2F1 | Breast | HMEC | 9623 | 7599/31605 | -0.17/0.26 | -181.7 | -180.0 | -28.80 | FALSE |
| SRX1961156 | TFs and others | ESR1 | Breast | T-47D | 8345 | 1491/31605 | -0.70/0.26 | -181.5 | -179.8 | -28.78 | FALSE |
| SRX190220 | TFs and others | ELF1 | Breast | MCF-7 | 26101 | 9835/31605 | -0.12/0.26 | -181.4 | -179.8 | -28.78 | FALSE |
| SRX7692225 | TFs and others | ARID1B | Breast | MCF-7 | 3916 | 546/31605 | -1.32/0.26 | -181.3 | -179.7 | -28.77 | FALSE |
| SRX2881007 | TFs and others | ESR1 | Breast | MCF-7 | 5006 | 543/31605 | -1.33/0.26 | -181.2 | -179.6 | -28.76 | FALSE |
| SRX5089453 | TFs and others | ESR1 | Breast | MCF-7 | 4706 | 795/31605 | -1.05/0.26 | -180.5 | -178.8 | -28.71 | FALSE |
| SRX8537714 | TFs and others | ESR1 | Breast | MCF-7 | 14283 | 1604/31605 | -0.66/0.26 | -180.4 | -178.7 | -28.70 | FALSE |
| SRX3285556 | TFs and others | ESR1 | Breast | C70R | 11848 | 1686/31605 | -0.64/0.26 | -180.3 | -178.7 | -28.69 | FALSE |
| SRX9932063 | TFs and others | HDAC6 | Breast | MCF-7 | 6281 | 628/31605 | -1.21/0.26 | -180.3 | -178.7 | -28.69 | FALSE |
| SRX3110712 | TFs and others | ESR1 | Breast | MCF-7 | 8939 | 1314/31605 | -0.76/0.26 | -180.2 | -178.6 | -28.68 | FALSE |
| SRX4193145 | TFs and others | ESR1 | Breast | MCF-7 | 10338 | 2730/31605 | -0.45/0.26 | -179.9 | -178.3 | -28.66 | FALSE |
| SRX3541100 | TFs and others | ESR1 | Breast | MCF-7 | 11999 | 1285/31605 | -0.77/0.26 | -179.9 | -178.3 | -28.66 | FALSE |
| SRX2513516 | TFs and others | ESR1 | Breast | MCF-7 | 6358 | 677/31605 | -1.15/0.26 | -179.8 | -178.2 | -28.65 | FALSE |
| SRX5089551 | TFs and others | BRD4 | Breast | MCF-7 | 18077 | 8790/31605 | -0.14/0.26 | -179.7 | -178.0 | -28.64 | FALSE |
| SRX3110714 | TFs and others | ESR1 | Breast | MCF-7 | 5758 | 864/31605 | -0.99/0.26 | -179.5 | -177.9 | -28.63 | FALSE |
| SRX3629596 | TFs and others | ESR1 | Breast | MCF-7 | 8493 | 910/31605 | -0.96/0.26 | -179.5 | -177.9 | -28.62 | FALSE |
| SRX8616990 | TFs and others | ESR1 | Breast | MCF-7 | 9362 | 1277/31605 | -0.77/0.26 | -178.9 | -177.3 | -28.58 | FALSE |
| SRX8616992 | TFs and others | ESR1 | Breast | MCF-7 | 7612 | 979/31605 | -0.91/0.26 | -178.7 | -177.1 | -28.56 | FALSE |
| SRX8616991 | TFs and others | ESR1 | Breast | MCF-7 | 9908 | 1199/31605 | -0.80/0.26 | -178.7 | -177.1 | -28.56 | FALSE |
| SRX24151139 | TFs and others | ESR1 | Breast | MCF-7 | 7791 | 769/31605 | -1.06/0.26 | -178.6 | -177.0 | -28.55 | FALSE |
| SRX6757651 | TFs and others | ESR1 | Breast | MCF-7 | 3917 | 368/31605 | -1.65/0.26 | -178.4 | -176.8 | -28.54 | FALSE |
| SRX8616979 | TFs and others | ESR1 | Breast | ZR-75-1 | 8034 | 1042/31605 | -0.88/0.26 | -178.4 | -176.8 | -28.54 | FALSE |
| SRX2897197 | TFs and others | ESR1 | Breast | Breast cancer cells | 30788 | 10796/31605 | -0.10/0.26 | -178.3 | -176.7 | -28.53 | FALSE |
| SRX1758493 | TFs and others | NELFE | Breast | MCF-7 | 8618 | 7055/31605 | -0.18/0.26 | -178.1 | -176.5 | -28.51 | FALSE |
| SRX1012626 | TFs and others | ESR1 | Breast | MCF-7 | 12532 | 1120/31605 | -0.84/0.26 | -177.8 | -176.3 | -28.49 | FALSE |
| SRX2871339 | TFs and others | ESR1 | Breast | T-47D | 10561 | 1894/31605 | -0.58/0.26 | -177.7 | -176.1 | -28.48 | FALSE |
| SRX730450 | TFs and others | ESR1 | Breast | MCF-7 | 12774 | 1652/31605 | -0.64/0.26 | -177.6 | -176.0 | -28.47 | FALSE |
| SRX8080544 | TFs and others | Epitope tags | Breast | Mammary gland ductal carcinoma | 31699 | 9857/31605 | -0.11/0.26 | -177.4 | -175.8 | -28.45 | FALSE |
| SRX1758496 | TFs and others | NELFE | Breast | MCF-7 | 7846 | 6711/31605 | -0.19/0.26 | -177.3 | -175.8 | -28.45 | FALSE |
| SRX19662627 | TFs and others | JUN | Breast | T-47D | 17476 | 11120/31605 | -0.09/0.26 | -177.3 | -175.7 | -28.45 | FALSE |
| SRX003938 | TFs and others | ESR1 | Breast | MCF-7 | 10395 | 1184/31605 | -0.80/0.26 | -177.3 | -175.7 | -28.45 | FALSE |
| SRX2424317 | TFs and others | MNT | Breast | MCF-7 | 16929 | 7970/31605 | -0.15/0.26 | -177.2 | -175.6 | -28.44 | FALSE |
| SRX2424318 | TFs and others | MNT | Breast | MCF-7 | 18065 | 7995/31605 | -0.15/0.26 | -177.1 | -175.6 | -28.43 | FALSE |
| SRX2922232 | TFs and others | ESR1 | Breast | SUM 44PE | 25223 | 4028/31605 | -0.32/0.26 | -177.0 | -175.4 | -28.42 | FALSE |
| SRX2513522 | TFs and others | ESR1 | Breast | MCF-7 | 10707 | 1141/31605 | -0.82/0.26 | -176.9 | -175.3 | -28.41 | FALSE |
| SRX4786549 | TFs and others | ESR1 | Breast | MCF-7 | 20378 | 5074/31605 | -0.26/0.26 | -176.7 | -175.2 | -28.40 | FALSE |
| SRX7692446 | TFs and others | POLR2C | Breast | MCF-7 | 16379 | 10008/31605 | -0.11/0.26 | -176.7 | -175.2 | -28.40 | FALSE |
| SRX3583203 | TFs and others | ESR1 | Breast | MCF-7 | 7903 | 625/31605 | -1.20/0.26 | -176.6 | -175.1 | -28.39 | FALSE |
| SRX9932051 | TFs and others | STAT5A | Breast | MCF-7 | 14935 | 7659/31605 | -0.16/0.26 | -176.3 | -174.7 | -28.37 | FALSE |
| SRX1961161 | TFs and others | ESR1 | Breast | T-47D | 6771 | 1236/31605 | -0.78/0.26 | -176.2 | -174.7 | -28.36 | FALSE |
| SRX9719175 | TFs and others | TET2 | Breast | MCF-7 | 6061 | 730/31605 | -1.09/0.26 | -176.2 | -174.6 | -28.36 | FALSE |
| SRX1961155 | TFs and others | ESR1 | Breast | T-47D | 8332 | 1468/31605 | -0.69/0.26 | -176.1 | -174.6 | -28.35 | FALSE |
| SRX3322532 | TFs and others | ZNF217 | Breast | MCF-7 | 17464 | 7316/31605 | -0.17/0.26 | -176.0 | -174.5 | -28.35 | FALSE |
| SRX8246294 | TFs and others | FGFR1 | Breast | MCF-7 | 13022 | 9121/31605 | -0.12/0.26 | -175.8 | -174.3 | -28.33 | FALSE |
| SRX5089457 | TFs and others | ESR1 | Breast | MCF-7 | 10049 | 1647/31605 | -0.64/0.26 | -175.8 | -174.3 | -28.33 | FALSE |
| SRX19024874 | TFs and others | ESR1 | Breast | MCF-7 | 10820 | 887/31605 | -0.96/0.26 | -175.6 | -174.1 | -28.31 | FALSE |
| SRX3629593 | TFs and others | ESR1 | Breast | MCF-7 | 9489 | 1064/31605 | -0.86/0.26 | -175.5 | -174.0 | -28.30 | FALSE |
| SRX3229145 | TFs and others | ESR1 | Breast | Breast cancer | 37694 | 10234/31605 | -0.10/0.26 | -175.5 | -174.0 | -28.30 | FALSE |
| SRX9932069 | TFs and others | HDAC6 | Breast | MCF-7 | 4249 | 374/31605 | -1.62/0.26 | -175.4 | -173.9 | -28.29 | FALSE |
| SRX3123824 | TFs and others | ESR1 | Breast | MCF-7 | 13743 | 1386/31605 | -0.72/0.26 | -175.3 | -173.8 | -28.28 | FALSE |
| SRX3629594 | TFs and others | ESR1 | Breast | MCF-7 | 11408 | 1256/31605 | -0.77/0.26 | -175.2 | -173.7 | -28.28 | FALSE |
| SRX5824595 | TFs and others | BRD2 | Breast | SUM 159PT | 14362 | 9554/31605 | -0.12/0.26 | -175.0 | -173.5 | -28.26 | FALSE |
| SRX1161170 | TFs and others | ESR1 | Breast | MCF-7 | 9247 | 834/31605 | -1.00/0.26 | -174.9 | -173.4 | -28.26 | FALSE |
| SRX1116525 | TFs and others | ESR1 | Breast | MCF-7 | 12733 | 2180/31605 | -0.52/0.26 | -174.9 | -173.4 | -28.25 | FALSE |
| SRX4451202 | TFs and others | ESR1 | Breast | MCF-7 | 3327 | 308/31605 | -1.81/0.26 | -174.8 | -173.3 | -28.24 | FALSE |
| SRX190320 | TFs and others | GABPA | Breast | MCF-7 | 16614 | 8346/31605 | -0.14/0.26 | -174.5 | -173.0 | -28.22 | FALSE |
| SRX21459789 | TFs and others | MYC | Breast | MCF-7 | 17706 | 9858/31605 | -0.11/0.26 | -174.4 | -172.9 | -28.21 | FALSE |
| SRX11169861 | TFs and others | ESR1 | Breast | MCF-7 | 9772 | 2706/31605 | -0.44/0.26 | -174.2 | -172.7 | -28.20 | FALSE |
| SRX4417023 | TFs and others | ESR1 | Breast | MCF-7 | 5220 | 867/31605 | -0.97/0.26 | -174.2 | -172.7 | -28.20 | FALSE |
| SRX8537728 | TFs and others | ESR1 | Breast | MDA-MB-134-VI | 4977 | 1058/31605 | -0.85/0.26 | -174.1 | -172.7 | -28.19 | FALSE |
| SRX4193142 | TFs and others | ESR1 | Breast | MCF-7 | 11185 | 3153/31605 | -0.39/0.26 | -174.1 | -172.7 | -28.19 | FALSE |
| SRX1116524 | TFs and others | ESR1 | Breast | MCF-7 | 10286 | 1966/31605 | -0.56/0.26 | -174.1 | -172.6 | -28.19 | FALSE |
| SRX5824560 | TFs and others | BRD2 | Breast | SUM 149PT | 24681 | 11059/31605 | -0.09/0.26 | -173.8 | -172.4 | -28.17 | FALSE |
| SRX5089500 | TFs and others | HDAC1 | Breast | MCF-7 | 16599 | 9569/31605 | -0.11/0.26 | -173.8 | -172.4 | -28.17 | FALSE |
| SRX4193143 | TFs and others | ESR1 | Breast | MCF-7 | 9767 | 2705/31605 | -0.44/0.26 | -173.6 | -172.2 | -28.15 | FALSE |
| SRX11169860 | TFs and others | ESR1 | Breast | MCF-7 | 11217 | 3175/31605 | -0.38/0.26 | -173.6 | -172.1 | -28.15 | FALSE |
| SRX2513525 | TFs and others | ESR1 | Breast | MCF-7 | 11236 | 1610/31605 | -0.64/0.26 | -173.6 | -172.1 | -28.15 | FALSE |
| SRX7692447 | TFs and others | POLR2C | Breast | MCF-7 | 14148 | 9456/31605 | -0.12/0.26 | -173.5 | -172.1 | -28.14 | FALSE |
| SRX10475558 | TFs and others | ESR1 | Breast | MCF-7 | 12853 | 1986/31605 | -0.55/0.26 | -173.5 | -172.1 | -28.14 | FALSE |
| SRX4213897 | TFs and others | LIN9 | Breast | MCF 10A | 7309 | 5934/31605 | -0.21/0.26 | -173.5 | -172.0 | -28.14 | FALSE |
| SRX5824588 | TFs and others | BRD2 | Breast | SUM 159PT | 9745 | 7325/31605 | -0.17/0.26 | -173.5 | -172.0 | -28.14 | FALSE |
| SRX6712572 | TFs and others | CBX3 | Breast | MCF-7 | 13247 | 8718/31605 | -0.13/0.26 | -173.0 | -171.6 | -28.10 | FALSE |
| SRX8617016 | TFs and others | TET2 | Breast | MCF-7 | 7332 | 791/31605 | -1.02/0.26 | -172.8 | -171.3 | -28.08 | FALSE |
| SRX1012746 | TFs and others | ESR1 | Breast | MCF-7 | 3052 | 477/31605 | -1.39/0.26 | -172.7 | -171.3 | -28.08 | FALSE |
| SRX190267 | TFs and others | TAF1 | Breast | MCF-7 | 10520 | 7366/31605 | -0.16/0.26 | -172.7 | -171.3 | -28.08 | FALSE |
| SRX5824566 | TFs and others | BRD2 | Breast | SUM 149PT | 24718 | 10856/31605 | -0.09/0.26 | -172.7 | -171.3 | -28.07 | FALSE |
| SRX5287691 | TFs and others | ESR1 | Breast | T-47D | 5665 | 850/31605 | -0.98/0.26 | -172.7 | -171.2 | -28.07 | FALSE |
| SRX8080562 | TFs and others | Epitope tags | Breast | Mammary gland adenocarcinoma | 25684 | 7157/31605 | -0.17/0.26 | -172.3 | -170.9 | -28.04 | FALSE |
| SRX24151140 | TFs and others | ESR1 | Breast | MCF-7 | 11443 | 1185/31605 | -0.79/0.26 | -172.3 | -170.9 | -28.04 | FALSE |
| SRX11120902 | TFs and others | ESR1 | Breast | MCF-7 | 10937 | 1251/31605 | -0.76/0.26 | -172.2 | -170.8 | -28.03 | FALSE |
| SRX4377550 | TFs and others | BRD2 | Breast | HCC1806 | 17726 | 10216/31605 | -0.10/0.26 | -172.2 | -170.7 | -28.03 | FALSE |
| SRX968967 | TFs and others | ESR1 | Breast | MCF-7 | 10511 | 1229/31605 | -0.77/0.26 | -172.0 | -170.6 | -28.02 | FALSE |
| SRX054576 | TFs and others | Epitope tags | Breast | MCF-7 | 23518 | 10725/31605 | -0.09/0.26 | -172.0 | -170.6 | -28.02 | FALSE |
| SRX8616961 | TFs and others | ESR1 | Breast | MCF-7 | 5839 | 762/31605 | -1.04/0.26 | -171.9 | -170.5 | -28.01 | FALSE |
| SRX3123817 | TFs and others | ESR1 | Breast | MCF-7 | 14184 | 1240/31605 | -0.76/0.26 | -171.9 | -170.5 | -28.01 | FALSE |
| SRX9932043 | TFs and others | STAT5A | Breast | MCF-7 | 14291 | 7014/31605 | -0.17/0.26 | -171.8 | -170.4 | -28.00 | FALSE |
| SRX4451208 | TFs and others | ESR1 | Breast | MCF-7 | 17604 | 2160/31605 | -0.52/0.26 | -171.8 | -170.4 | -28.00 | FALSE |
| SRX5089554 | TFs and others | BRD4 | Breast | MCF-7 | 19353 | 9321/31605 | -0.12/0.26 | -171.8 | -170.4 | -28.00 | FALSE |
| SRX2881003 | TFs and others | ESR1 | Breast | MCF-7 | 13305 | 1765/31605 | -0.60/0.26 | -171.7 | -170.3 | -27.99 | FALSE |
| SRX16364106 | TFs and others | ESR1 | Breast | MCF-7 | 16929 | 2396/31605 | -0.48/0.26 | -171.6 | -170.2 | -27.98 | FALSE |
| SRX9932065 | TFs and others | HDAC6 | Breast | MCF-7 | 4506 | 417/31605 | -1.50/0.26 | -171.4 | -170.0 | -27.97 | FALSE |
| SRX3583195 | TFs and others | ESR1 | Breast | MCF-7 | 5378 | 458/31605 | -1.42/0.26 | -171.2 | -169.8 | -27.95 | FALSE |
| SRX5824574 | TFs and others | BRD2 | Breast | SUM 149PT | 19856 | 9435/31605 | -0.11/0.26 | -171.2 | -169.8 | -27.95 | FALSE |
| SRX4213896 | TFs and others | LIN9 | Breast | MCF 10A | 6232 | 5305/31605 | -0.24/0.26 | -171.2 | -169.8 | -27.95 | FALSE |
| SRX150480 | TFs and others | E2F4 | Breast | MCF 10A | 19055 | 9235/31605 | -0.12/0.26 | -171.1 | -169.7 | -27.94 | FALSE |
| SRX107369 | TFs and others | ESR1 | Breast | H3396 | 13719 | 1432/31605 | -0.69/0.26 | -170.9 | -169.6 | -27.93 | FALSE |
| SRX5824584 | TFs and others | BRD2 | Breast | SUM 159PT | 13958 | 8982/31605 | -0.12/0.26 | -170.7 | -169.3 | -27.91 | FALSE |
| SRX5702624 | TFs and others | ESR1 | Breast | Breast cancer cells | 18087 | 4528/31605 | -0.28/0.26 | -170.7 | -169.3 | -27.91 | FALSE |
| SRX8617019 | TFs and others | ESR1 | Breast | MCF-7 | 13951 | 1838/31605 | -0.58/0.26 | -170.6 | -169.2 | -27.90 | FALSE |
| SRX4377549 | TFs and others | BRD2 | Breast | HCC1806 | 17115 | 10313/31605 | -0.10/0.26 | -170.6 | -169.2 | -27.90 | FALSE |
| SRX8616989 | TFs and others | ESR1 | Breast | MCF-7 | 12614 | 1596/31605 | -0.64/0.26 | -170.6 | -169.2 | -27.90 | FALSE |
| SRX8617015 | TFs and others | ESR1 | Breast | MCF-7 | 12163 | 1577/31605 | -0.64/0.26 | -170.6 | -169.2 | -27.90 | FALSE |
| SRX19024871 | TFs and others | ESR1 | Breast | MCF-7 | 10435 | 843/31605 | -0.98/0.26 | -170.5 | -169.1 | -27.89 | FALSE |
| SRX8537729 | TFs and others | ESR1 | Breast | MDA-MB-134-VI | 5795 | 1230/31605 | -0.76/0.26 | -170.2 | -168.8 | -27.87 | FALSE |
| SRX194566 | TFs and others | E2F4 | Breast | MCF-7 | 3851 | 2927/31605 | -0.40/0.26 | -170.0 | -168.7 | -27.85 | FALSE |
| SRX10475557 | TFs and others | ESR1 | Breast | MCF-7 | 13501 | 1890/31605 | -0.57/0.26 | -170.0 | -168.6 | -27.85 | FALSE |
| SRX3541111 | TFs and others | ESR1 | Breast | MCF-7 | 4537 | 336/31605 | -1.69/0.26 | -169.9 | -168.5 | -27.84 | FALSE |
| SRX1012627 | TFs and others | ESR1 | Breast | MCF-7 | 11822 | 1035/31605 | -0.85/0.26 | -169.8 | -168.4 | -27.83 | FALSE |
| SRX1012744 | TFs and others | ESR1 | Breast | MCF-7 | 4061 | 673/31605 | -1.12/0.26 | -169.7 | -168.4 | -27.83 | FALSE |
| SRX4193144 | TFs and others | ESR1 | Breast | MCF-7 | 10366 | 2529/31605 | -0.45/0.26 | -169.6 | -168.3 | -27.82 | FALSE |
| SRX5089497 | TFs and others | HDAC1 | Breast | MCF-7 | 14920 | 9343/31605 | -0.11/0.26 | -169.6 | -168.2 | -27.82 | FALSE |
| SRX1961162 | TFs and others | ESR1 | Breast | T-47D | 5833 | 1013/31605 | -0.86/0.26 | -169.5 | -168.1 | -27.81 | FALSE |
| SRX2922227 | TFs and others | ESR1 | Breast | MCF-7 | 11022 | 4797/31605 | -0.26/0.26 | -169.4 | -168.1 | -27.80 | FALSE |
| SRX5534847 | TFs and others | ESR1 | Breast | MCF-7 | 4257 | 470/31605 | -1.39/0.26 | -169.2 | -167.9 | -27.79 | FALSE |
| SRX4377551 | TFs and others | BRD2 | Breast | MDA-MB-231 | 23811 | 10968/31605 | -0.08/0.26 | -168.8 | -167.5 | -27.76 | FALSE |
| SRX5824575 | TFs and others | BRD2 | Breast | SUM 149PT | 17920 | 9254/31605 | -0.11/0.26 | -168.8 | -167.5 | -27.75 | FALSE |
| SRX5824565 | TFs and others | BRD2 | Breast | SUM 149PT | 30686 | 11381/31605 | -0.08/0.26 | -168.4 | -167.1 | -27.72 | FALSE |
| SRX9719174 | TFs and others | TET2 | Breast | MCF-7 | 5707 | 623/31605 | -1.17/0.26 | -168.3 | -166.9 | -27.71 | FALSE |
| SRX13991435 | TFs and others | MAFB | Breast | MCF 10A | 15693 | 10690/31605 | -0.09/0.26 | -168.2 | -166.9 | -27.70 | FALSE |
| SRX4451206 | TFs and others | ESR1 | Breast | MCF-7 | 11674 | 1464/31605 | -0.67/0.26 | -168.1 | -166.8 | -27.70 | FALSE |
| SRX21459786 | TFs and others | MYC | Breast | MCF-7 | 42458 | 11726/31605 | -0.07/0.26 | -168.0 | -166.7 | -27.69 | FALSE |
| SRX6413749 | TFs and others | ESR1 | Breast | MCF-7 | 15009 | 1861/31605 | -0.57/0.26 | -167.9 | -166.6 | -27.68 | FALSE |
| SRX4377528 | TFs and others | BRD2 | Breast | MDA-MB-231 | 23866 | 10969/31605 | -0.08/0.26 | -167.8 | -166.5 | -27.67 | FALSE |
| SRX5824594 | TFs and others | BRD2 | Breast | SUM 159PT | 19246 | 10368/31605 | -0.09/0.26 | -167.8 | -166.5 | -27.67 | FALSE |
| SRX9932045 | TFs and others | STAT5A | Breast | MCF-7 | 14311 | 7074/31605 | -0.17/0.26 | -167.8 | -166.4 | -27.67 | FALSE |
| SRX8537715 | TFs and others | ESR1 | Breast | MCF-7 | 14013 | 1635/31605 | -0.62/0.26 | -167.5 | -166.2 | -27.65 | FALSE |
| SRX5287698 | TFs and others | ESR1 | Breast | MCF-7 | 19611 | 2522/31605 | -0.45/0.26 | -167.4 | -166.1 | -27.64 | FALSE |
| SRX9322280 | TFs and others | ESR1 | Breast | MCF-7 | 5034 | 524/31605 | -1.29/0.26 | -167.2 | -165.9 | -27.62 | FALSE |
| SRX5702623 | TFs and others | ESR1 | Breast | Breast cancer cells | 27866 | 7145/31605 | -0.16/0.26 | -167.1 | -165.8 | -27.62 | FALSE |
| SRX21459793 | TFs and others | MYC | Breast | BT-549 | 11625 | 8388/31605 | -0.13/0.26 | -167.1 | -165.8 | -27.61 | FALSE |
| SRX4193146 | TFs and others | ESR1 | Breast | MCF-7 | 8167 | 3038/31605 | -0.39/0.26 | -166.7 | -165.4 | -27.58 | FALSE |
| SRX3229139 | TFs and others | ESR1 | Breast | Breast cancer | 9803 | 5026/31605 | -0.24/0.26 | -166.7 | -165.4 | -27.58 | FALSE |
| SRX10775028 | TFs and others | ESR1 | Breast | T-47D | 4023 | 390/31605 | -1.53/0.26 | -166.7 | -165.4 | -27.58 | FALSE |
| SRX5089452 | TFs and others | ESR1 | Breast | MCF-7 | 8051 | 1646/31605 | -0.62/0.26 | -166.6 | -165.3 | -27.57 | FALSE |
| SRX150556 | TFs and others | E2F1 | Breast | MCF-7 | 25785 | 11118/31605 | -0.08/0.26 | -166.3 | -165.0 | -27.55 | FALSE |
| SRX1758520 | TFs and others | NELFE | Breast | MCF-7 | 7355 | 6420/31605 | -0.19/0.26 | -166.2 | -164.9 | -27.54 | FALSE |
| SRX4451207 | TFs and others | ESR1 | Breast | MCF-7 | 20075 | 2622/31605 | -0.43/0.26 | -166.2 | -164.9 | -27.54 | FALSE |
| SRX1531796 | TFs and others | ESR1 | Breast | MCF-7 | 28858 | 4075/31605 | -0.30/0.26 | -166.2 | -164.9 | -27.54 | FALSE |
| SRX8080551 | TFs and others | Epitope tags | Breast | Mammary gland ductal carcinoma | 31084 | 8999/31605 | -0.12/0.26 | -166.1 | -164.8 | -27.53 | FALSE |
| SRX19024873 | TFs and others | ESR1 | Breast | MCF-7 | 10315 | 806/31605 | -0.99/0.26 | -166.1 | -164.8 | -27.53 | FALSE |
| SRX8272753 | TFs and others | ESR2 | Breast | MCF-7 | 10421 | 1566/31605 | -0.64/0.26 | -166.0 | -164.7 | -27.52 | FALSE |
| SRX054571 | TFs and others | Epitope tags | Breast | MCF-7 | 21821 | 10955/31605 | -0.08/0.26 | -165.9 | -164.6 | -27.51 | FALSE |
| SRX13991436 | TFs and others | MAFB | Breast | MCF 10A | 19084 | 11209/31605 | -0.08/0.26 | -165.8 | -164.5 | -27.51 | FALSE |
| SRX190295 | TFs and others | SRF | Breast | MCF-7 | 14996 | 6966/31605 | -0.17/0.26 | -165.4 | -164.2 | -27.47 | FALSE |
| SRX1758494 | TFs and others | NELFE | Breast | MCF-7 | 7808 | 6536/31605 | -0.18/0.26 | -165.3 | -164.0 | -27.46 | FALSE |
| SRX026530 | TFs and others | ESR1 | Breast | MCF-7 | 10023 | 1341/31605 | -0.71/0.26 | -165.3 | -164.0 | -27.46 | FALSE |
| SRX792932 | TFs and others | ESR1 | Breast | LY2 | 23971 | 4837/31605 | -0.25/0.26 | -165.0 | -163.8 | -27.44 | FALSE |
| SRX099865 | TFs and others | MYC | Breast | MCF-7 | 32285 | 11096/31605 | -0.08/0.26 | -165.0 | -163.7 | -27.44 | FALSE |
| SRX5089498 | TFs and others | HDAC1 | Breast | MCF-7 | 14699 | 9170/31605 | -0.11/0.26 | -164.7 | -163.4 | -27.41 | FALSE |
| SRX8617022 | TFs and others | TET2 | Breast | MCF-7 | 3239 | 356/31605 | -1.61/0.26 | -164.7 | -163.4 | -27.41 | FALSE |
| SRX10775050 | TFs and others | ESR1 | Breast | T-47D | 4100 | 428/31605 | -1.44/0.26 | -164.7 | -163.4 | -27.41 | FALSE |
| SRX2922234 | TFs and others | ESR1 | Breast | SUM 44PE | 18226 | 3093/31605 | -0.38/0.26 | -164.6 | -163.4 | -27.41 | FALSE |
| SRX5251431 | TFs and others | ESR1 | Breast | T-47D | 5255 | 932/31605 | -0.89/0.26 | -164.5 | -163.2 | -27.39 | FALSE |
| SRX5824579 | TFs and others | BRD2 | Breast | SUM 159PT | 24016 | 11072/31605 | -0.08/0.26 | -164.4 | -163.1 | -27.38 | FALSE |
| SRX4377552 | TFs and others | BRD2 | Breast | MDA-MB-231 | 24462 | 11066/31605 | -0.08/0.26 | -164.2 | -163.0 | -27.37 | FALSE |
| SRX3229149 | TFs and others | ESR1 | Breast | Breast cancer | 14297 | 6606/31605 | -0.18/0.26 | -164.0 | -162.7 | -27.35 | FALSE |
| SRX9322281 | TFs and others | ESR1 | Breast | MCF-7 | 5508 | 546/31605 | -1.24/0.26 | -164.0 | -162.7 | -27.35 | FALSE |
| SRX5089503 | TFs and others | HDAC1 | Breast | MCF-7 | 11793 | 8980/31605 | -0.11/0.26 | -163.7 | -162.4 | -27.33 | FALSE |
| SRX3123828 | TFs and others | ESR1 | Breast | MCF-7 | 14573 | 1348/31605 | -0.70/0.26 | -163.4 | -162.1 | -27.30 | FALSE |
| SRX5824578 | TFs and others | BRD2 | Breast | SUM 159PT | 22716 | 11185/31605 | -0.08/0.26 | -163.3 | -162.0 | -27.29 | FALSE |
| SRX2897205 | TFs and others | ESR1 | Breast | Breast cancer cells | 16757 | 8354/31605 | -0.13/0.26 | -162.9 | -161.7 | -27.26 | FALSE |
| SRX13991437 | TFs and others | WDR77 | Breast | MCF 10A | 10576 | 7231/31605 | -0.16/0.26 | -162.7 | -161.4 | -27.24 | FALSE |
| SRX054574 | TFs and others | Epitope tags | Breast | MCF-7 | 16817 | 10554/31605 | -0.08/0.26 | -162.5 | -161.2 | -27.23 | FALSE |
| SRX8080550 | TFs and others | Epitope tags | Breast | Mammary gland ductal carcinoma | 32112 | 7453/31605 | -0.15/0.26 | -162.5 | -161.2 | -27.22 | FALSE |
| SRX4377529 | TFs and others | BRD2 | Breast | MDA-MB-231 | 24519 | 11071/31605 | -0.08/0.26 | -162.4 | -161.2 | -27.22 | FALSE |
| SRX3583211 | TFs and others | ESR1 | Breast | MCF-7 | 4750 | 423/31605 | -1.44/0.26 | -162.4 | -161.1 | -27.22 | FALSE |
| SRX016327 | TFs and others | ESR1 | Breast | MCF-7 | 4501 | 520/31605 | -1.27/0.26 | -162.3 | -161.0 | -27.21 | FALSE |
| SRX16318946 | TFs and others | MYC | Breast | MDA-MB-231 | 15362 | 7269/31605 | -0.15/0.26 | -162.1 | -160.9 | -27.19 | FALSE |
| SRX20282944 | TFs and others | ESR1 | Breast | MCF-7 | 14090 | 1605/31605 | -0.61/0.26 | -162.1 | -160.8 | -27.19 | FALSE |
| SRX2871340 | TFs and others | ESR1 | Breast | T-47D | 8469 | 1682/31605 | -0.59/0.26 | -162.1 | -160.8 | -27.19 | FALSE |
| SRX1758479 | TFs and others | NELFE | Breast | MCF-7 | 7398 | 6625/31605 | -0.17/0.26 | -161.9 | -160.6 | -27.17 | FALSE |
| SRX9419931 | TFs and others | MED1 | Breast | SK-BR-3 | 33532 | 10062/31605 | -0.09/0.26 | -161.8 | -160.6 | -27.17 | FALSE |
| SRX5824570 | TFs and others | BRD2 | Breast | SUM 149PT | 25280 | 11263/31605 | -0.07/0.26 | -161.8 | -160.6 | -27.17 | FALSE |
| SRX19024875 | TFs and others | ESR1 | Breast | MCF-7 | 13011 | 1079/31605 | -0.80/0.26 | -161.7 | -160.5 | -27.16 | FALSE |
| SRX3229161 | TFs and others | ESR1 | Breast | Breast cancer | 43333 | 10584/31605 | -0.08/0.26 | -161.7 | -160.5 | -27.16 | FALSE |
| SRX8617017 | TFs and others | ESR1 | Breast | MCF-7 | 14470 | 2021/31605 | -0.52/0.26 | -161.4 | -160.1 | -27.13 | FALSE |
| SRX250095 | TFs and others | TCF7L2 | Breast | MDA-MB-453 | 35237 | 10681/31605 | -0.08/0.26 | -161.2 | -160.0 | -27.12 | FALSE |
| SRX10775026 | TFs and others | ESR1 | Breast | T-47D | 3923 | 397/31605 | -1.48/0.26 | -160.7 | -159.5 | -27.08 | FALSE |
| SRX8080553 | TFs and others | Epitope tags | Breast | Mammary gland ductal carcinoma | 31246 | 9410/31605 | -0.10/0.26 | -160.7 | -159.5 | -27.07 | FALSE |
| SRX8537725 | TFs and others | ESR1 | Breast | MCF-7 | 4502 | 866/31605 | -0.92/0.26 | -160.6 | -159.4 | -27.07 | FALSE |
| SRX1758508 | TFs and others | NELFE | Breast | MCF-7 | 7270 | 6372/31605 | -0.18/0.26 | -160.6 | -159.4 | -27.07 | FALSE |
| SRX968964 | TFs and others | ESR1 | Breast | MCF-7 | 14172 | 2562/31605 | -0.43/0.26 | -160.6 | -159.4 | -27.07 | FALSE |
| SRX747777 | TFs and others | ESR1 | Breast | MCF-7 | 12046 | 1068/31605 | -0.81/0.26 | -160.6 | -159.4 | -27.06 | FALSE |
| SRX3123822 | TFs and others | ESR1 | Breast | MCF-7 | 15587 | 1525/31605 | -0.63/0.26 | -160.5 | -159.3 | -27.06 | FALSE |
| SRX7624161 | TFs and others | ESR1 | Breast | T-47D | 33190 | 6366/31605 | -0.18/0.26 | -160.5 | -159.2 | -27.05 | FALSE |
| SRX5757566 | TFs and others | SMAD3 | Breast | SUM 159PT | 14532 | 10347/31605 | -0.09/0.26 | -160.4 | -159.2 | -27.05 | FALSE |
| SRX4377545 | TFs and others | BRD2 | Breast | SUM 159PT | 19200 | 10797/31605 | -0.08/0.26 | -160.4 | -159.1 | -27.05 | FALSE |
| SRX5702620 | TFs and others | ESR1 | Breast | T-47D | 18413 | 2672/31605 | -0.41/0.26 | -160.4 | -159.1 | -27.05 | FALSE |
| SRX2424331 | TFs and others | HCFC1 | Breast | MCF-7 | 9717 | 6146/31605 | -0.19/0.26 | -160.3 | -159.1 | -27.04 | FALSE |
| SRX13991439 | TFs and others | WDR77 | Breast | MCF 10A | 11481 | 7780/31605 | -0.14/0.26 | -160.2 | -159.0 | -27.03 | FALSE |
| SRX5128133 | TFs and others | ESR1 | Breast | ZR-75-1 | 10935 | 1445/31605 | -0.65/0.26 | -159.7 | -158.5 | -26.99 | FALSE |
| SRX5757568 | TFs and others | SMAD3 | Breast | WHIM12 | 17029 | 11125/31605 | -0.07/0.26 | -159.7 | -158.5 | -26.99 | FALSE |
| SRX7624162 | TFs and others | ESR1 | Breast | T-47D | 32867 | 5886/31605 | -0.20/0.26 | -159.6 | -158.4 | -26.98 | FALSE |
| SRX4193147 | TFs and others | ESR1 | Breast | MCF-7 | 9435 | 2570/31605 | -0.43/0.26 | -159.5 | -158.3 | -26.97 | FALSE |
| SRX8537724 | TFs and others | ESR1 | Breast | MCF-7 | 4542 | 866/31605 | -0.92/0.26 | -159.3 | -158.1 | -26.95 | FALSE |
| SRX9419956 | TFs and others | BRD4 | Breast | SK-BR-3 | 40771 | 11259/31605 | -0.07/0.26 | -159.3 | -158.1 | -26.95 | FALSE |
| SRX8080545 | TFs and others | Epitope tags | Breast | Mammary gland ductal carcinoma | 16265 | 5409/31605 | -0.21/0.26 | -159.2 | -158.0 | -26.95 | FALSE |
| SRX21459788 | TFs and others | MYC | Breast | MCF-7 | 29947 | 11807/31605 | -0.06/0.26 | -159.2 | -158.0 | -26.95 | FALSE |
| SRX4377537 | TFs and others | BRD2 | Breast | MDA-MB-231 | 25407 | 11179/31605 | -0.07/0.26 | -159.2 | -158.0 | -26.94 | FALSE |
| SRX19024872 | TFs and others | ESR1 | Breast | MCF-7 | 10222 | 851/31605 | -0.93/0.26 | -159.0 | -157.8 | -26.93 | FALSE |
| SRX20282945 | TFs and others | ESR1 | Breast | MCF-7 | 17864 | 2356/31605 | -0.46/0.26 | -158.9 | -157.7 | -26.92 | FALSE |
| SRX5128132 | TFs and others | ESR1 | Breast | ZR-75-1 | 11075 | 1416/31605 | -0.66/0.26 | -158.8 | -157.6 | -26.91 | FALSE |
| SRX4377541 | TFs and others | BRD2 | Breast | MDA-MB-231 | 18398 | 10593/31605 | -0.08/0.26 | -158.8 | -157.6 | -26.91 | FALSE |
| SRX323400 | TFs and others | ESR1 | Breast | MCF-7 | 16215 | 1893/31605 | -0.54/0.26 | -158.7 | -157.5 | -26.90 | FALSE |
| SRX6777399 | TFs and others | ESR1 | Breast | MCF-7 | 2831 | 293/31605 | -1.76/0.26 | -158.6 | -157.4 | -26.89 | FALSE |
| SRX3541098 | TFs and others | ESR1 | Breast | MCF-7 | 3424 | 464/31605 | -1.34/0.26 | -158.5 | -157.3 | -26.89 | FALSE |
| SRX5824569 | TFs and others | BRD2 | Breast | SUM 149PT | 25114 | 11285/31605 | -0.07/0.26 | -158.5 | -157.3 | -26.89 | FALSE |
| SRX21459787 | TFs and others | MYC | Breast | MCF-7 | 33572 | 11656/31605 | -0.06/0.26 | -158.4 | -157.2 | -26.88 | FALSE |
| SRX10579964 | TFs and others | BRD4 | Breast | MCF-7 | 15566 | 9646/31605 | -0.10/0.26 | -158.4 | -157.2 | -26.88 | FALSE |
| SRX1469954 | TFs and others | ESR1 | Breast | T-47D | 6341 | 1161/31605 | -0.75/0.26 | -158.2 | -157.1 | -26.86 | FALSE |
| SRX21459778 | TFs and others | MYC | Breast | BT-549 | 13492 | 9749/31605 | -0.09/0.26 | -158.2 | -157.0 | -26.86 | FALSE |
| SRX103003 | TFs and others | MYC | Breast | MCF-7 | 36652 | 8001/31605 | -0.13/0.26 | -158.1 | -156.9 | -26.85 | FALSE |
| SRX4377553 | TFs and others | BRD2 | Breast | MDA-MB-231 | 23161 | 10948/31605 | -0.07/0.26 | -158.1 | -156.9 | -26.85 | FALSE |
| SRX8616967 | TFs and others | TET2 | Breast | MCF-7 | 4115 | 430/31605 | -1.40/0.26 | -158.1 | -156.9 | -26.85 | FALSE |
| SRX2881004 | TFs and others | ESR1 | Breast | MCF-7 | 16703 | 2392/31605 | -0.45/0.26 | -158.0 | -156.9 | -26.85 | FALSE |
| SRX666558 | TFs and others | Epitope tags | Breast | T-47D | 29871 | 10201/31605 | -0.09/0.26 | -158.0 | -156.8 | -26.85 | FALSE |
| SRX9932077 | TFs and others | HDAC6 | Breast | MCF-7 | 3330 | 373/31605 | -1.52/0.26 | -157.9 | -156.8 | -26.84 | FALSE |
| SRX9419922 | TFs and others | BRD4 | Breast | SK-BR-3 | 44686 | 11373/31605 | -0.07/0.26 | -157.8 | -156.7 | -26.83 | FALSE |
| SRX2636530 | TFs and others | MBD2 | Breast | MCF-7 | 11236 | 5236/31605 | -0.22/0.26 | -157.7 | -156.6 | -26.82 | FALSE |
| SRX2145567 | TFs and others | ESR1 | Breast | MCF-7 | 14832 | 1699/31605 | -0.58/0.26 | -157.4 | -156.2 | -26.79 | FALSE |
| SRX4377530 | TFs and others | BRD2 | Breast | MDA-MB-231 | 23193 | 10950/31605 | -0.07/0.26 | -157.4 | -156.2 | -26.79 | FALSE |
| SRX23740458 | TFs and others | KDM5A | Breast | SUM 149PT | 12349 | 8945/31605 | -0.11/0.26 | -157.4 | -156.2 | -26.79 | FALSE |
| SRX2513523 | TFs and others | ESR1 | Breast | MCF-7 | 18726 | 2209/31605 | -0.47/0.26 | -157.3 | -156.2 | -26.79 | FALSE |
| SRX2871341 | TFs and others | ESR1 | Breast | T-47D | 9479 | 1913/31605 | -0.53/0.26 | -157.2 | -156.0 | -26.77 | FALSE |
| SRX8080566 | TFs and others | POU2F1 | Breast | Mammary gland ductal carcinoma | 20790 | 7820/31605 | -0.13/0.26 | -156.9 | -155.7 | -26.75 | FALSE |
| SRX9419954 | TFs and others | BRD4 | Breast | SK-BR-3 | 35610 | 11028/31605 | -0.07/0.26 | -156.5 | -155.3 | -26.72 | FALSE |
| SRX9419932 | TFs and others | MED1 | Breast | SK-BR-3 | 35317 | 10196/31605 | -0.08/0.26 | -156.3 | -155.1 | -26.69 | FALSE |
| SRX4786545 | TFs and others | TET2 | Breast | MCF-7 | 11454 | 7147/31605 | -0.15/0.26 | -156.0 | -154.9 | -26.68 | FALSE |
| SRX1510971 | TFs and others | ZNF143 | Breast | MCF-7 | 28878 | 10865/31605 | -0.07/0.26 | -156.0 | -154.8 | -26.67 | FALSE |
| SRX4377546 | TFs and others | BRD2 | Breast | SUM 159PT | 21036 | 10838/31605 | -0.07/0.26 | -156.0 | -154.8 | -26.67 | FALSE |
| SRX4377538 | TFs and others | BRD2 | Breast | MDA-MB-231 | 24831 | 11229/31605 | -0.07/0.26 | -155.9 | -154.8 | -26.67 | FALSE |
| SRX4377539 | TFs and others | BRD2 | Breast | MDA-MB-231 | 29040 | 11481/31605 | -0.06/0.26 | -155.9 | -154.7 | -26.66 | FALSE |
| SRX730428 | TFs and others | NCAPG | Breast | MCF-7 | 2846 | 382/31605 | -1.49/0.26 | -155.9 | -154.7 | -26.66 | FALSE |
| SRX8616963 | TFs and others | TET2 | Breast | MCF-7 | 4351 | 494/31605 | -1.28/0.26 | -155.8 | -154.6 | -26.65 | FALSE |
| SRX11867602 | TFs and others | KMT2C | Breast | CAL-51 | 13302 | 7825/31605 | -0.13/0.26 | -155.7 | -154.6 | -26.65 | FALSE |
| SRX9932061 | TFs and others | STAT5A | Breast | MCF-7 | 11621 | 6808/31605 | -0.16/0.26 | -155.5 | -154.4 | -26.63 | FALSE |
| SRX5287697 | TFs and others | ESR1 | Breast | MCF-7 | 25655 | 2795/31605 | -0.39/0.26 | -155.5 | -154.4 | -26.63 | FALSE |
| SRX4451217 | TFs and others | ESR1 | Breast | MCF-7 | 19136 | 2339/31605 | -0.45/0.26 | -155.4 | -154.3 | -26.62 | FALSE |
| SRX12813811 | TFs and others | HEXIM1 | Breast | MCF-7 | 10470 | 6113/31605 | -0.18/0.26 | -155.3 | -154.1 | -26.61 | FALSE |
| SRX523396 | TFs and others | APOBEC3B | Breast | MCF-7 | 7715 | 1715/31605 | -0.57/0.26 | -155.2 | -154.1 | -26.61 | FALSE |
| SRX3123810 | TFs and others | ESR1 | Breast | MCF-7 | 17820 | 1693/31605 | -0.57/0.26 | -155.2 | -154.0 | -26.60 | FALSE |
| SRX1758523 | TFs and others | Epitope tags | Breast | MCF-7 | 7718 | 6926/31605 | -0.15/0.26 | -155.1 | -153.9 | -26.59 | FALSE |
| SRX11169863 | TFs and others | ESR1 | Breast | MCF-7 | 11276 | 4073/31605 | -0.28/0.26 | -155.0 | -153.8 | -26.58 | FALSE |
| SRX1758481 | TFs and others | NELFE | Breast | MCF-7 | 6976 | 6181/31605 | -0.18/0.26 | -154.9 | -153.8 | -26.58 | FALSE |
| SRX3229146 | TFs and others | ESR1 | Breast | Breast cancer | 13841 | 4601/31605 | -0.25/0.26 | -154.8 | -153.7 | -26.57 | FALSE |
| SRX4193149 | TFs and others | ESR1 | Breast | MCF-7 | 11295 | 4079/31605 | -0.28/0.26 | -154.6 | -153.5 | -26.55 | FALSE |
| SRX1469953 | TFs and others | ESR1 | Breast | T-47D | 5324 | 846/31605 | -0.91/0.26 | -154.4 | -153.2 | -26.53 | FALSE |
| SRX11867604 | TFs and others | KMT2C | Breast | CAL-51 | 25745 | 10124/31605 | -0.08/0.26 | -154.3 | -153.2 | -26.53 | FALSE |
| SRX1012647 | TFs and others | EP300 | Breast | MCF-7 | 20125 | 3970/31605 | -0.28/0.26 | -154.2 | -153.1 | -26.52 | FALSE |
| SRX17989898 | TFs and others | SAP30 | Breast | MDA-MB-231 | 20015 | 11283/31605 | -0.06/0.26 | -154.2 | -153.0 | -26.51 | FALSE |
| SRX3229156 | TFs and others | ESR1 | Breast | Breast cancer | 15654 | 5870/31605 | -0.19/0.26 | -154.0 | -152.9 | -26.50 | FALSE |
| SRX16364102 | TFs and others | ESR1 | Breast | MCF-7 | 20431 | 3058/31605 | -0.36/0.26 | -154.0 | -152.8 | -26.50 | FALSE |
| SRX4786575 | TFs and others | KDM6A | Breast | MCF-7 | 13032 | 7445/31605 | -0.14/0.26 | -153.8 | -152.6 | -26.48 | FALSE |
| SRX8616997 | TFs and others | TET2 | Breast | MCF-7 | 12225 | 1673/31605 | -0.57/0.26 | -153.7 | -152.6 | -26.48 | FALSE |
| SRX4377554 | TFs and others | BRD2 | Breast | MDA-MB-231 | 18635 | 9877/31605 | -0.09/0.26 | -153.6 | -152.4 | -26.46 | FALSE |
| SRX10024466 | TFs and others | ESR1 | Breast | MCF-7 | 3763 | 371/31605 | -1.50/0.26 | -153.5 | -152.4 | -26.46 | FALSE |
| SRX8080570 | TFs and others | POU2F1 | Breast | Mammary gland ductal carcinoma | 32845 | 10940/31605 | -0.07/0.26 | -153.5 | -152.4 | -26.46 | FALSE |
| SRX747802 | TFs and others | ESR1 | Breast | MCF-7 | 6395 | 778/31605 | -0.96/0.26 | -153.5 | -152.3 | -26.45 | FALSE |
| SRX8616977 | TFs and others | ESR1 | Breast | ZR-75-1 | 9150 | 1407/31605 | -0.65/0.26 | -153.4 | -152.3 | -26.45 | FALSE |
| SRX054575 | TFs and others | Epitope tags | Breast | MCF-7 | 33565 | 11951/31605 | -0.05/0.26 | -153.3 | -152.2 | -26.44 | FALSE |
| SRX5702621 | TFs and others | ESR1 | Breast | T-47D | 17982 | 2556/31605 | -0.41/0.26 | -153.2 | -152.1 | -26.43 | FALSE |
| SRX7902939 | TFs and others | BRD4 | Breast | SUM 159PT | 26080 | 10819/31605 | -0.07/0.26 | -153.1 | -152.0 | -26.42 | FALSE |
| SRX003939 | TFs and others | ESR1 | Breast | MCF-7 | 8066 | 1093/31605 | -0.77/0.26 | -153.1 | -152.0 | -26.42 | FALSE |
| SRX4451216 | TFs and others | ESR1 | Breast | MCF-7 | 17361 | 2399/31605 | -0.44/0.26 | -152.9 | -151.8 | -26.40 | FALSE |
| SRX185912 | TFs and others | FOXM1 | Breast | MDA-MB-231 | 19059 | 7416/31605 | -0.14/0.26 | -152.9 | -151.8 | -26.40 | FALSE |
| SRX5702625 | TFs and others | ESR1 | Breast | Breast cancer cells | 6831 | 1724/31605 | -0.56/0.26 | -152.9 | -151.7 | -26.40 | FALSE |
| SRX2943585 | TFs and others | ESR1 | Breast | MCF-7 | 10477 | 1364/31605 | -0.66/0.26 | -152.8 | -151.7 | -26.40 | FALSE |
| SRX11867605 | TFs and others | KMT2C | Breast | CAL-51 | 24540 | 10108/31605 | -0.08/0.26 | -152.8 | -151.7 | -26.40 | FALSE |
| SRX21459799 | TFs and others | BRD4 | Breast | BT-549 | 22244 | 9914/31605 | -0.08/0.26 | -152.8 | -151.7 | -26.40 | FALSE |
| ERX470435 | TFs and others | NRIP1 | Breast | MCF-7 | 14287 | 1533/31605 | -0.61/0.26 | -152.7 | -151.6 | -26.39 | FALSE |
| SRX9719173 | TFs and others | TET2 | Breast | MCF-7 | 8188 | 940/31605 | -0.85/0.26 | -152.7 | -151.5 | -26.38 | FALSE |
| SRX10468622 | TFs and others | ESR1 | Breast | Breast cancer cells | 26458 | 6220/31605 | -0.17/0.26 | -152.6 | -151.5 | -26.38 | FALSE |
| SRX10365551 | TFs and others | NCOA3 | Breast | MCF-7 | 12028 | 1125/31605 | -0.75/0.26 | -152.6 | -151.5 | -26.37 | FALSE |
| SRX8861815 | TFs and others | ESR1 | Breast | T-47D | 8538 | 1278/31605 | -0.69/0.26 | -152.5 | -151.4 | -26.37 | FALSE |
| SRX21459795 | TFs and others | BRD4 | Breast | BT-549 | 28436 | 9647/31605 | -0.09/0.26 | -152.5 | -151.4 | -26.37 | FALSE |
| SRX1758519 | TFs and others | NELFE | Breast | MCF-7 | 5481 | 5235/31605 | -0.21/0.26 | -152.4 | -151.3 | -26.36 | FALSE |
| SRX103000 | TFs and others | MYC | Breast | MCF-7 | 52498 | 12219/31605 | -0.05/0.26 | -152.2 | -151.1 | -26.34 | FALSE |
| SRX1469952 | TFs and others | ESR1 | Breast | T-47D | 6369 | 977/31605 | -0.82/0.26 | -152.2 | -151.1 | -26.34 | FALSE |
| SRX9419924 | TFs and others | BRD4 | Breast | SK-BR-3 | 36471 | 10504/31605 | -0.07/0.26 | -152.1 | -151.0 | -26.33 | FALSE |
| SRX9932073 | TFs and others | HDAC6 | Breast | MCF-7 | 3624 | 398/31605 | -1.43/0.26 | -152.1 | -151.0 | -26.33 | FALSE |
| SRX5323807 | TFs and others | ESR1 | Breast | T-47D | 4377 | 674/31605 | -1.04/0.26 | -152.0 | -150.9 | -26.32 | FALSE |
| SRX5089461 | TFs and others | ESR1 | Breast | MCF-7 | 12544 | 2203/31605 | -0.46/0.26 | -151.8 | -150.7 | -26.30 | FALSE |
| SRX21459800 | TFs and others | BRD4 | Breast | BT-549 | 22211 | 9612/31605 | -0.09/0.26 | -151.7 | -150.6 | -26.30 | FALSE |
| SRX4377531 | TFs and others | BRD2 | Breast | MDA-MB-231 | 18741 | 9915/31605 | -0.08/0.26 | -151.7 | -150.6 | -26.30 | FALSE |
| SRX1469950 | TFs and others | ESR1 | Breast | MCF-7 | 22305 | 2481/31605 | -0.42/0.26 | -151.7 | -150.6 | -26.30 | FALSE |
| SRX9419933 | TFs and others | MED1 | Breast | SK-BR-3 | 38678 | 10666/31605 | -0.07/0.26 | -151.5 | -150.4 | -26.28 | FALSE |
| SRX5824585 | TFs and others | BRD2 | Breast | SUM 159PT | 9322 | 7037/31605 | -0.15/0.26 | -151.5 | -150.4 | -26.28 | FALSE |
| SRX21459798 | TFs and others | BRD4 | Breast | BT-549 | 23672 | 10558/31605 | -0.07/0.26 | -151.4 | -150.3 | -26.27 | FALSE |
| SRX3091903 | TFs and others | ZFX | Breast | MCF-7 | 33775 | 8904/31605 | -0.10/0.26 | -151.4 | -150.3 | -26.27 | FALSE |
| SRX747778 | TFs and others | ESR1 | Breast | MCF-7 | 13623 | 1279/31605 | -0.69/0.26 | -151.3 | -150.2 | -26.27 | FALSE |
| SRX8861813 | TFs and others | ESR1 | Breast | T-47D | 3752 | 538/31605 | -1.19/0.26 | -151.2 | -150.1 | -26.26 | FALSE |
| SRX23740430 | TFs and others | KDM5A | Breast | SUM 149PT | 17511 | 11311/31605 | -0.06/0.26 | -151.2 | -150.1 | -26.26 | FALSE |
| SRX003940 | TFs and others | ESR1 | Breast | MCF-7 | 3805 | 411/31605 | -1.40/0.26 | -151.1 | -150.0 | -26.24 | FALSE |
| SRX9419923 | TFs and others | BRD4 | Breast | SK-BR-3 | 42101 | 10977/31605 | -0.07/0.26 | -150.9 | -149.8 | -26.23 | FALSE |
| SRX384490 | TFs and others | XBP1 | Breast | T-47D | 14667 | 6854/31605 | -0.15/0.26 | -150.8 | -149.8 | -26.22 | FALSE |
| SRX21459801 | TFs and others | BRD4 | Breast | BT-549 | 21771 | 10539/31605 | -0.07/0.26 | -150.8 | -149.7 | -26.22 | FALSE |
| SRX8537721 | TFs and others | ESR1 | Breast | T-47D | 4229 | 591/31605 | -1.13/0.26 | -150.8 | -149.7 | -26.22 | FALSE |
| SRX21459794 | TFs and others | BRD4 | Breast | BT-549 | 28548 | 10402/31605 | -0.07/0.26 | -150.8 | -149.7 | -26.22 | FALSE |
| SRX5757570 | TFs and others | SMAD3 | Breast | WHIM12 | 20291 | 11502/31605 | -0.06/0.26 | -150.7 | -149.6 | -26.21 | FALSE |
| SRX4377540 | TFs and others | BRD2 | Breast | MDA-MB-231 | 20379 | 10722/31605 | -0.07/0.26 | -150.5 | -149.4 | -26.19 | FALSE |
| SRX5709753 | TFs and others | CTCF | Breast | MCF-7 | 7660 | 6735/31605 | -0.15/0.26 | -150.5 | -149.4 | -26.19 | FALSE |
| SRX5251422 | TFs and others | ESR1 | Breast | MCF-7 | 2768 | 311/31605 | -1.65/0.26 | -150.4 | -149.3 | -26.18 | FALSE |
| SRX23740461 | TFs and others | KDM5A | Breast | SUM 149PT | 11193 | 8256/31605 | -0.11/0.26 | -150.3 | -149.2 | -26.18 | FALSE |
| SRX5757564 | TFs and others | SMAD3 | Breast | SUM 159PT | 20630 | 11197/31605 | -0.06/0.26 | -150.3 | -149.2 | -26.17 | FALSE |
| SRX3229142 | TFs and others | ESR1 | Breast | Breast cancer | 13517 | 4231/31605 | -0.26/0.26 | -150.3 | -149.2 | -26.17 | FALSE |
| SRX2943580 | TFs and others | ESR1 | Breast | MCF-7 | 7905 | 1088/31605 | -0.76/0.26 | -150.1 | -149.0 | -26.16 | FALSE |
| SRX9932055 | TFs and others | STAT5A | Breast | MCF-7 | 10933 | 6478/31605 | -0.16/0.26 | -150.1 | -149.0 | -26.16 | FALSE |
| SRX23740431 | TFs and others | KDM5A | Breast | SUM 149PT | 15556 | 10880/31605 | -0.07/0.26 | -150.1 | -149.0 | -26.16 | FALSE |
| SRX2900152 | TFs and others | KMT2C | Breast | CAL-51 | 13876 | 7742/31605 | -0.13/0.26 | -150.0 | -148.9 | -26.15 | FALSE |
| SRX4115992 | TFs and others | ESR1 | Breast | MCF-7 | 2469 | 330/31605 | -1.59/0.26 | -149.9 | -148.8 | -26.14 | FALSE |
| SRX1161201 | TFs and others | ESR1 | Breast | T-47D | 11751 | 1240/31605 | -0.70/0.26 | -149.8 | -148.7 | -26.13 | FALSE |
| SRX8639779 | TFs and others | ESR1 | Breast | MDA-MB-134-VI | 3045 | 442/31605 | -1.33/0.26 | -149.5 | -148.5 | -26.11 | FALSE |
| SRX3110721 | TFs and others | ESR1 | Breast | MCF-7 | 12231 | 2885/31605 | -0.37/0.26 | -149.5 | -148.4 | -26.11 | FALSE |
| SRX8537716 | TFs and others | ESR1 | Breast | MDA-MB-134-VI | 12366 | 2033/31605 | -0.49/0.26 | -149.4 | -148.4 | -26.10 | FALSE |
| SRX8080559 | TFs and others | Epitope tags | Breast | Mammary gland adenocarcinoma | 23010 | 4827/31605 | -0.23/0.26 | -149.4 | -148.4 | -26.10 | FALSE |
| SRX21459796 | TFs and others | BRD4 | Breast | BT-549 | 23589 | 9154/31605 | -0.09/0.26 | -149.4 | -148.3 | -26.09 | FALSE |
| SRX100584 | TFs and others | ESR1 | Breast | T-47D | 17652 | 1974/31605 | -0.50/0.26 | -149.3 | -148.3 | -26.09 | FALSE |
| SRX968965 | TFs and others | ESR1 | Breast | MCF-7 | 8520 | 1127/31605 | -0.74/0.26 | -149.2 | -148.2 | -26.08 | FALSE |
| SRX3322359 | TFs and others | ZFX | Breast | MCF-7 | 35718 | 8983/31605 | -0.10/0.26 | -149.2 | -148.1 | -26.08 | FALSE |
| SRX21459792 | TFs and others | MYC | Breast | BT-549 | 7739 | 6468/31605 | -0.16/0.26 | -149.2 | -148.1 | -26.08 | FALSE |
| SRX2897209 | TFs and others | ESR1 | Breast | Breast cancer cells | 15615 | 8692/31605 | -0.10/0.26 | -149.1 | -148.0 | -26.07 | FALSE |
| SRX11169862 | TFs and others | ESR1 | Breast | MCF-7 | 6263 | 2059/31605 | -0.48/0.26 | -149.1 | -148.0 | -26.07 | FALSE |
| SRX11120870 | TFs and others | CREBBP | Breast | MCF-7 | 10228 | 3640/31605 | -0.30/0.26 | -149.0 | -148.0 | -26.06 | FALSE |
| SRX9419955 | TFs and others | BRD4 | Breast | SK-BR-3 | 31233 | 10109/31605 | -0.08/0.26 | -148.9 | -147.8 | -26.05 | FALSE |
| SRX2900159 | TFs and others | KDM6A | Breast | CAL-51 | 12120 | 7836/31605 | -0.12/0.26 | -148.7 | -147.7 | -26.04 | FALSE |
| SRX9109114 | TFs and others | AR | Breast | MDA-MB-453 | 49557 | 11577/31605 | -0.05/0.26 | -148.7 | -147.6 | -26.03 | FALSE |
| SRX5089560 | TFs and others | BRD4 | Breast | MCF-7 | 11915 | 6771/31605 | -0.15/0.26 | -148.6 | -147.6 | -26.03 | FALSE |
| SRX4451215 | TFs and others | ESR1 | Breast | MCF-7 | 15057 | 1534/31605 | -0.60/0.26 | -148.5 | -147.5 | -26.02 | FALSE |
| SRX2912059 | TFs and others | ESR1 | Breast | MCF-7 | 15741 | 1144/31605 | -0.73/0.26 | -148.5 | -147.4 | -26.01 | FALSE |
| SRX1510969 | TFs and others | ZNF143 | Breast | MCF-7 | 35264 | 11749/31605 | -0.05/0.26 | -148.4 | -147.3 | -26.01 | FALSE |
| SRX182682 | TFs and others | BRCA1 | Breast | MCF 10A | 6465 | 5630/31605 | -0.19/0.26 | -148.4 | -147.3 | -26.00 | FALSE |
| SRX2194269 | TFs and others | BRD4 | Breast | SUM 159PT | 37549 | 10704/31605 | -0.07/0.26 | -148.3 | -147.3 | -26.00 | FALSE |
| SRX5323870 | TFs and others | STAT3 | Breast | MCF-7 | 22942 | 7835/31605 | -0.12/0.26 | -148.0 | -147.0 | -25.98 | FALSE |
| SRX10468617 | TFs and others | ESR1 | Breast | Breast cancer cells | 18060 | 5069/31605 | -0.21/0.26 | -148.0 | -146.9 | -25.97 | FALSE |
| SRX21459781 | TFs and others | MYC | Breast | BT-549 | 14019 | 7542/31605 | -0.13/0.26 | -147.9 | -146.8 | -25.96 | FALSE |
| SRX10024465 | TFs and others | ESR1 | Breast | MCF-7 | 4007 | 381/31605 | -1.45/0.26 | -147.8 | -146.8 | -25.96 | FALSE |
| SRX11867600 | TFs and others | KMT2C | Breast | CAL-51 | 13038 | 7508/31605 | -0.13/0.26 | -147.8 | -146.8 | -25.96 | FALSE |
| SRX8861816 | TFs and others | ESR1 | Breast | T-47D | 4485 | 763/31605 | -0.95/0.26 | -147.6 | -146.5 | -25.94 | FALSE |
| SRX2585781 | TFs and others | E2F1 | Breast | MDA-MB-231 | 42548 | 11672/31605 | -0.05/0.26 | -147.5 | -146.5 | -25.93 | FALSE |
| SRX8080568 | TFs and others | POU2F1 | Breast | Mammary gland ductal carcinoma | 32405 | 11274/31605 | -0.06/0.26 | -147.4 | -146.4 | -25.92 | FALSE |
| SRX3322358 | TFs and others | ZFX | Breast | MCF-7 | 11638 | 7195/31605 | -0.14/0.26 | -147.4 | -146.4 | -25.92 | FALSE |
| SRX7865936 | TFs and others | BRD4 | Breast | MDA-MB-231 | 27042 | 7512/31605 | -0.13/0.26 | -147.2 | -146.2 | -25.90 | FALSE |
| SRX5323813 | TFs and others | ESR1 | Breast | T-47D | 8338 | 1345/31605 | -0.65/0.26 | -147.2 | -146.1 | -25.90 | FALSE |
| SRX8861819 | TFs and others | ESR1 | Breast | T-47D | 4971 | 863/31605 | -0.87/0.26 | -147.1 | -146.1 | -25.90 | FALSE |
| SRX17989897 | TFs and others | SAP30 | Breast | MDA-MB-231 | 22748 | 11795/31605 | -0.05/0.26 | -147.0 | -146.0 | -25.89 | FALSE |
| SRX4377548 | TFs and others | BRD2 | Breast | HCC1806 | 28649 | 12006/31605 | -0.05/0.26 | -146.9 | -145.9 | -25.88 | FALSE |
| SRX8272752 | TFs and others | ESR2 | Breast | MCF-7 | 14667 | 2145/31605 | -0.46/0.26 | -146.9 | -145.8 | -25.87 | FALSE |
| SRX5089446 | TFs and others | ESR1 | Breast | MCF-7 | 15615 | 2108/31605 | -0.47/0.26 | -146.8 | -145.8 | -25.87 | FALSE |
| SRX8616965 | TFs and others | TET2 | Breast | MCF-7 | 4082 | 495/31605 | -1.23/0.26 | -146.7 | -145.6 | -25.85 | FALSE |
| SRX8537726 | TFs and others | ESR1 | Breast | MDA-MB-134-VI | 5432 | 1228/31605 | -0.69/0.26 | -146.6 | -145.6 | -25.85 | FALSE |
| SRX9109109 | TFs and others | AR | Breast | MDA-MB-453 | 53865 | 10823/31605 | -0.06/0.26 | -146.6 | -145.6 | -25.85 | FALSE |
| SRX4193148 | TFs and others | ESR1 | Breast | MCF-7 | 6255 | 2047/31605 | -0.48/0.26 | -146.6 | -145.6 | -25.85 | FALSE |
| SRX1787675 | TFs and others | KMT2C | Breast | MCF-7 | 29229 | 6467/31605 | -0.16/0.26 | -146.5 | -145.5 | -25.84 | FALSE |
| SRX4377543 | TFs and others | BRD2 | Breast | SUM 159PT | 29614 | 11602/31605 | -0.05/0.26 | -146.5 | -145.4 | -25.84 | FALSE |
| SRX8616986 | TFs and others | ESR1 | Breast | AB555 | 9629 | 1959/31605 | -0.49/0.26 | -146.4 | -145.4 | -25.83 | FALSE |
| SRX747781 | TFs and others | ESR1 | Breast | MCF-7 | 9287 | 954/31605 | -0.81/0.26 | -146.2 | -145.2 | -25.81 | FALSE |
| SRX4451200 | TFs and others | ESR1 | Breast | MCF-7 | 2290 | 200/31605 | -2.08/0.26 | -146.1 | -145.1 | -25.81 | FALSE |
| SRX9932062 | TFs and others | HDAC6 | Breast | MCF-7 | 2623 | 268/31605 | -1.76/0.26 | -146.0 | -145.0 | -25.80 | FALSE |
| SRX1012683 | TFs and others | ESR1 | Breast | MCF-7 | 3520 | 316/31605 | -1.60/0.26 | -145.9 | -144.9 | -25.79 | FALSE |
| SRX3091902 | TFs and others | ZFX | Breast | MCF-7 | 11618 | 7165/31605 | -0.13/0.26 | -145.8 | -144.8 | -25.78 | FALSE |
| SRX5323814 | TFs and others | ESR1 | Breast | T-47D | 6100 | 974/31605 | -0.80/0.26 | -145.8 | -144.8 | -25.78 | FALSE |
| SRX2194286 | TFs and others | BRD4 | Breast | SUM 229PE | 30137 | 10118/31605 | -0.07/0.26 | -145.8 | -144.8 | -25.78 | FALSE |
| SRX2943591 | TFs and others | ESR1 | Breast | MCF-7 | 34938 | 4663/31605 | -0.23/0.26 | -145.8 | -144.8 | -25.78 | FALSE |
| SRX8616985 | TFs and others | ESR1 | Breast | STG195 | 8475 | 1758/31605 | -0.53/0.26 | -145.7 | -144.7 | -25.77 | FALSE |
| SRX13327979 | TFs and others | XRCC6 | Breast | MCF-7 | 22189 | 10165/31605 | -0.07/0.26 | -145.7 | -144.6 | -25.76 | FALSE |
| SRX2194275 | TFs and others | BRD4 | Breast | SUM 159PT | 42578 | 11184/31605 | -0.06/0.26 | -145.7 | -144.6 | -25.76 | FALSE |
| SRX2194273 | TFs and others | BRD4 | Breast | SUM 159PT | 39326 | 11004/31605 | -0.06/0.26 | -145.5 | -144.5 | -25.75 | FALSE |
| SRX10468616 | TFs and others | ESR1 | Breast | Breast cancer cells | 25839 | 6733/31605 | -0.15/0.26 | -145.3 | -144.3 | -25.74 | FALSE |
| SRX13340676 | TFs and others | Epitope tags | Breast | T-47D | 18626 | 6677/31605 | -0.15/0.26 | -145.3 | -144.3 | -25.73 | FALSE |
| SRX1161172 | TFs and others | FOXA1 | Breast | MCF-7 | 40273 | 8907/31605 | -0.09/0.26 | -145.3 | -144.3 | -25.73 | FALSE |
| SRX371473 | TFs and others | ESR1 | Breast | T-47D | 18179 | 2099/31605 | -0.46/0.26 | -145.3 | -144.3 | -25.73 | FALSE |
| SRX194575 | TFs and others | ESR1 | Breast | MCF-7 | 29500 | 6213/31605 | -0.16/0.26 | -145.0 | -144.0 | -25.71 | FALSE |
| SRX23740455 | TFs and others | KDM5A | Breast | SUM 149PT | 11704 | 8598/31605 | -0.10/0.26 | -144.8 | -143.8 | -25.69 | FALSE |
| SRX1521297 | TFs and others | ZMYND8 | Breast | ZR-75-30 | 14531 | 5633/31605 | -0.18/0.26 | -144.8 | -143.8 | -25.69 | FALSE |
| SRX21459785 | TFs and others | MYC | Breast | MCF-7 | 45072 | 12917/31605 | -0.03/0.26 | -144.7 | -143.7 | -25.68 | FALSE |
| SRX8537730 | TFs and others | ESR1 | Breast | PE1B | 22590 | 5568/31605 | -0.19/0.26 | -144.7 | -143.7 | -25.68 | FALSE |
| SRX2194283 | TFs and others | BRD4 | Breast | SUM 159PT | 32633 | 9578/31605 | -0.08/0.26 | -144.5 | -143.5 | -25.66 | FALSE |
| SRX23740439 | TFs and others | KDM5A | Breast | SUM 149PT | 17513 | 11295/31605 | -0.05/0.26 | -144.5 | -143.5 | -25.66 | FALSE |
| SRX16318947 | TFs and others | MYC | Breast | MDA-MB-231 | 10435 | 6030/31605 | -0.17/0.26 | -144.5 | -143.5 | -25.66 | FALSE |
| SRX5089509 | TFs and others | HDAC1 | Breast | MCF-7 | 10546 | 8292/31605 | -0.11/0.26 | -144.4 | -143.4 | -25.65 | FALSE |
| SRX10468615 | TFs and others | ESR1 | Breast | Breast cancer cells | 15237 | 6000/31605 | -0.17/0.26 | -144.3 | -143.3 | -25.65 | FALSE |
| SRX1012689 | TFs and others | ESR1 | Breast | MCF-7 | 3378 | 304/31605 | -1.63/0.26 | -144.3 | -143.3 | -25.64 | FALSE |
| SRX11867603 | TFs and others | KMT2C | Breast | CAL-51 | 12837 | 7368/31605 | -0.13/0.26 | -144.3 | -143.3 | -25.64 | FALSE |
| SRX2194270 | TFs and others | BRD4 | Breast | SUM 159PT | 38027 | 10719/31605 | -0.06/0.26 | -144.1 | -143.1 | -25.63 | FALSE |
| SRX13991438 | TFs and others | WDR77 | Breast | MCF 10A | 7690 | 5679/31605 | -0.18/0.26 | -144.0 | -143.0 | -25.62 | FALSE |
| SRX1161171 | TFs and others | ESR1 | Breast | MCF-7 | 14470 | 1050/31605 | -0.76/0.26 | -143.9 | -142.9 | -25.61 | FALSE |
| SRX5287706 | TFs and others | ESR1 | Breast | MCF-7 | 11611 | 1300/31605 | -0.65/0.26 | -143.9 | -142.9 | -25.61 | FALSE |
| SRX8080563 | TFs and others | Epitope tags | Breast | Mammary gland adenocarcinoma | 20855 | 3847/31605 | -0.27/0.26 | -143.8 | -142.8 | -25.60 | FALSE |
| SRX4663586 | TFs and others | ESR1 | Breast | MCF-7 | 11852 | 849/31605 | -0.87/0.26 | -143.7 | -142.7 | -25.59 | FALSE |
| SRX21459784 | TFs and others | MYC | Breast | MCF-7 | 41225 | 12878/31605 | -0.03/0.26 | -143.6 | -142.6 | -25.58 | FALSE |
| SRX9419962 | TFs and others | MED1 | Breast | SK-BR-3 | 39443 | 10579/31605 | -0.06/0.26 | -143.6 | -142.6 | -25.58 | FALSE |
| SRX5323811 | TFs and others | ESR1 | Breast | T-47D | 3966 | 582/31605 | -1.10/0.26 | -143.5 | -142.5 | -25.57 | FALSE |
| SRX4377547 | TFs and others | BRD2 | Breast | HCC1806 | 29412 | 12046/31605 | -0.04/0.26 | -143.4 | -142.4 | -25.56 | FALSE |
| SRX10775021 | TFs and others | ESR1 | Breast | T-47D | 3836 | 333/31605 | -1.54/0.26 | -143.4 | -142.4 | -25.56 | FALSE |
| SRX100419 | TFs and others | ESR1 | Breast | T-47D | 15312 | 2077/31605 | -0.46/0.26 | -143.3 | -142.3 | -25.55 | FALSE |
| SRX6712596 | TFs and others | NCAPG | Breast | MCF-7 | 11861 | 7313/31605 | -0.13/0.26 | -143.3 | -142.3 | -25.55 | FALSE |
| SRX7902933 | TFs and others | BRD4 | Breast | SUM 159PT | 26805 | 11022/31605 | -0.06/0.26 | -143.1 | -142.1 | -25.53 | FALSE |
| SRX6413750 | TFs and others | ESR1 | Breast | MCF-7 | 12629 | 1782/31605 | -0.52/0.26 | -143.0 | -142.0 | -25.53 | FALSE |
| SRX1224602 | TFs and others | BRD4 | Breast | MCF 10A | 10669 | 6801/31605 | -0.14/0.26 | -142.9 | -141.9 | -25.52 | FALSE |
| SRX6712574 | TFs and others | CTCF | Breast | MCF-7 | 11026 | 7445/31605 | -0.12/0.26 | -142.9 | -141.9 | -25.52 | FALSE |
| SRX18454201 | TFs and others | BRD4 | Breast | MDA-MB-231 | 12825 | 9341/31605 | -0.08/0.26 | -142.6 | -141.6 | -25.49 | FALSE |
| SRX6712570 | TFs and others | CBX3 | Breast | MCF-7 | 40122 | 7061/31605 | -0.13/0.26 | -142.6 | -141.6 | -25.49 | FALSE |
| SRX5089556 | TFs and others | BRD4 | Breast | MCF-7 | 7842 | 5051/31605 | -0.20/0.26 | -142.6 | -141.6 | -25.49 | FALSE |
| SRX673750 | TFs and others | MED1 | Breast | MCF-7 | 12337 | 7956/31605 | -0.11/0.26 | -142.2 | -141.2 | -25.46 | FALSE |
| SRX5534853 | TFs and others | ESR1 | Breast | MCF-7 | 5061 | 604/31605 | -1.07/0.26 | -142.2 | -141.2 | -25.46 | FALSE |
| SRX23740438 | TFs and others | KDM5A | Breast | SUM 149PT | 17768 | 11355/31605 | -0.05/0.26 | -142.1 | -141.1 | -25.44 | FALSE |
| SRX21459783 | TFs and others | MYC | Breast | BT-549 | 21934 | 11267/31605 | -0.05/0.26 | -141.8 | -140.8 | -25.42 | FALSE |
| SRX2900153 | TFs and others | KMT2C | Breast | CAL-51 | 14562 | 7458/31605 | -0.12/0.26 | -141.7 | -140.8 | -25.41 | FALSE |
| SRX4377532 | TFs and others | BRD4 | Breast | MDA-MB-231 | 40430 | 11284/31605 | -0.05/0.26 | -141.7 | -140.7 | -25.40 | FALSE |
| SRX2943589 | TFs and others | ESR1 | Breast | MCF-7 | 28387 | 3507/31605 | -0.29/0.26 | -141.5 | -140.5 | -25.39 | FALSE |
| SRX4377555 | TFs and others | BRD4 | Breast | MDA-MB-231 | 40432 | 11286/31605 | -0.05/0.26 | -141.5 | -140.5 | -25.39 | FALSE |
| SRX2194277 | TFs and others | BRD4 | Breast | SUM 159PT | 41003 | 11156/31605 | -0.05/0.26 | -141.3 | -140.3 | -25.37 | FALSE |
| SRX20282967 | TFs and others | ESR1 | Breast | MCF-7 | 19073 | 2168/31605 | -0.44/0.26 | -141.2 | -140.3 | -25.37 | FALSE |
| SRX21459782 | TFs and others | MYC | Breast | BT-549 | 11170 | 6567/31605 | -0.15/0.26 | -141.2 | -140.3 | -25.37 | FALSE |
| SRX7846778 | TFs and others | ESR1 | Breast | MCF-7 | 8034 | 630/31605 | -1.04/0.26 | -141.2 | -140.2 | -25.37 | FALSE |
| SRX10024473 | TFs and others | ESR1 | Breast | MCF-7 | 3627 | 331/31605 | -1.53/0.26 | -141.1 | -140.1 | -25.35 | FALSE |
| SRX2194231 | TFs and others | BRD4 | Breast | SUM 159PT | 19013 | 7925/31605 | -0.11/0.26 | -141.0 | -140.1 | -25.35 | FALSE |
| SRX3857007 | TFs and others | CDKN1B | Breast | MDA-MB-231 | 20786 | 10424/31605 | -0.06/0.26 | -140.9 | -139.9 | -25.33 | FALSE |
| SRX13991440 | TFs and others | WDR77 | Breast | MCF 10A | 8486 | 6287/31605 | -0.15/0.26 | -140.8 | -139.9 | -25.33 | FALSE |
| SRX9419925 | TFs and others | BRD4 | Breast | SK-BR-3 | 31283 | 9581/31605 | -0.08/0.26 | -140.8 | -139.8 | -25.33 | FALSE |
| SRX8861814 | TFs and others | ESR1 | Breast | T-47D | 12490 | 2422/31605 | -0.40/0.26 | -140.7 | -139.7 | -25.32 | FALSE |
| SRX16028851 | TFs and others | MINA | Breast | MDA-MB-231 | 5222 | 4763/31605 | -0.21/0.26 | -140.7 | -139.7 | -25.32 | FALSE |
| SRX8537727 | TFs and others | ESR1 | Breast | MDA-MB-134-VI | 4791 | 1113/31605 | -0.72/0.26 | -140.4 | -139.5 | -25.29 | FALSE |
| SRX3110718 | TFs and others | ESR1 | Breast | MCF-7 | 7923 | 1617/31605 | -0.55/0.26 | -140.4 | -139.4 | -25.29 | FALSE |
| SRX14255714 | TFs and others | ESR1 | Breast | MCF-7 | 8808 | 801/31605 | -0.89/0.26 | -140.1 | -139.2 | -25.27 | FALSE |
| SRX371472 | TFs and others | ESR1 | Breast | T-47D | 18002 | 2323/31605 | -0.42/0.26 | -140.0 | -139.1 | -25.26 | FALSE |
| SRX10773084 | TFs and others | TCF7 | Breast | MB 157 | 27501 | 10020/31605 | -0.07/0.26 | -139.9 | -139.0 | -25.25 | FALSE |
| SRX5323808 | TFs and others | ESR1 | Breast | T-47D | 5552 | 1021/31605 | -0.76/0.26 | -139.7 | -138.7 | -25.23 | FALSE |
| SRX9719170 | TFs and others | ESR1 | Breast | MCF-7 | 2626 | 313/31605 | -1.57/0.26 | -139.7 | -138.7 | -25.23 | FALSE |
| SRX5709754 | TFs and others | CTCF | Breast | MCF-7 | 7538 | 6560/31605 | -0.14/0.26 | -139.6 | -138.6 | -25.22 | FALSE |
| SRX9932071 | TFs and others | HDAC6 | Breast | MCF-7 | 2637 | 311/31605 | -1.58/0.26 | -139.5 | -138.6 | -25.21 | FALSE |
| SRX3080213 | TFs and others | ESR1 | Breast | MCF-7 | 17990 | 1606/31605 | -0.55/0.26 | -139.5 | -138.5 | -25.21 | FALSE |
| SRX3110720 | TFs and others | ESR1 | Breast | MCF-7 | 25435 | 2800/31605 | -0.35/0.26 | -139.4 | -138.5 | -25.20 | FALSE |
| SRX8537717 | TFs and others | ESR1 | Breast | MDA-MB-134-VI | 17419 | 2793/31605 | -0.36/0.26 | -139.3 | -138.3 | -25.19 | FALSE |
| SRX11647484 | TFs and others | ESR1 | Breast | MCF-7 | 2627 | 231/31605 | -1.87/0.26 | -139.3 | -138.3 | -25.19 | FALSE |
| SRX19843156 | TFs and others | Epitope tags | Breast | MCF-7 | 21378 | 10195/31605 | -0.06/0.26 | -139.2 | -138.2 | -25.18 | FALSE |
| SRX9719168 | TFs and others | ESR1 | Breast | MCF-7 | 17837 | 2259/31605 | -0.42/0.26 | -139.1 | -138.2 | -25.18 | FALSE |
| SRX250091 | TFs and others | MYC | Breast | MDA-MB-453 | 47822 | 12628/31605 | -0.03/0.26 | -139.1 | -138.1 | -25.17 | FALSE |
| SRX7030913 | TFs and others | HEXIM1 | Breast | MCF-7 | 15647 | 7899/31605 | -0.11/0.26 | -139.0 | -138.0 | -25.16 | FALSE |
| SRX8861820 | TFs and others | ESR1 | Breast | T-47D | 7297 | 1283/31605 | -0.64/0.26 | -139.0 | -138.0 | -25.16 | FALSE |
| SRX4377544 | TFs and others | BRD2 | Breast | SUM 159PT | 39080 | 11934/31605 | -0.04/0.26 | -138.8 | -137.9 | -25.15 | FALSE |
| SRX5323866 | TFs and others | STAT3 | Breast | MCF-7 | 18732 | 6994/31605 | -0.13/0.26 | -138.5 | -137.6 | -25.12 | FALSE |
| SRX1012748 | TFs and others | ESR1 | Breast | MCF-7 | 2677 | 342/31605 | -1.48/0.26 | -138.5 | -137.5 | -25.11 | FALSE |
| SRX747782 | TFs and others | ESR1 | Breast | MCF-7 | 11615 | 1251/31605 | -0.65/0.26 | -138.4 | -137.5 | -25.11 | FALSE |
| SRX2194261 | TFs and others | BRD4 | Breast | SUM 159PT | 49107 | 11474/31605 | -0.05/0.26 | -138.3 | -137.4 | -25.10 | FALSE |
| SRX9719169 | TFs and others | ESR1 | Breast | MCF-7 | 18400 | 2469/31605 | -0.39/0.26 | -138.3 | -137.3 | -25.10 | FALSE |
| SRX3123830 | TFs and others | ESR1 | Breast | MCF-7 | 19650 | 1869/31605 | -0.49/0.26 | -138.0 | -137.1 | -25.07 | FALSE |
| SRX5128104 | TFs and others | ESR1 | Breast | ZR-75-1 | 4833 | 557/31605 | -1.11/0.26 | -138.0 | -137.0 | -25.07 | FALSE |
| SRX3541105 | TFs and others | ESR1 | Breast | MCF-7 | 3331 | 277/31605 | -1.67/0.26 | -138.0 | -137.0 | -25.07 | FALSE |
| SRX9932067 | TFs and others | HDAC6 | Breast | MCF-7 | 2968 | 280/31605 | -1.66/0.26 | -137.8 | -136.9 | -25.06 | FALSE |
| SRX265414 | TFs and others | KDM5B | Breast | MCF-7 | 46969 | 12776/31605 | -0.03/0.26 | -137.8 | -136.8 | -25.05 | FALSE |
| SRX21459802 | TFs and others | KAT2A | Breast | BT-549 | 22335 | 7574/31605 | -0.11/0.26 | -137.5 | -136.6 | -25.03 | FALSE |
| SRX21459780 | TFs and others | MYC | Breast | BT-549 | 11375 | 6472/31605 | -0.14/0.26 | -137.3 | -136.4 | -25.01 | FALSE |
| SRX176859 | TFs and others | ESR1 | Breast | MCF-7 | 19151 | 2378/31605 | -0.40/0.26 | -137.2 | -136.3 | -25.00 | FALSE |
| SRX14733327 | TFs and others | ESR1 | Breast | MCF-7 | 21145 | 5147/31605 | -0.19/0.26 | -136.9 | -136.0 | -24.98 | FALSE |
| SRX8639783 | TFs and others | ESR1 | Breast | MDA-MB-134-VI | 8080 | 1373/31605 | -0.61/0.26 | -136.8 | -135.8 | -24.96 | FALSE |
| SRX9719176 | TFs and others | TET2 | Breast | MCF-7 | 4478 | 480/31605 | -1.20/0.26 | -136.7 | -135.7 | -24.95 | FALSE |
| SRX16874142 | TFs and others | ESR1 | Breast | MCF-7 | 3691 | 368/31605 | -1.41/0.26 | -136.6 | -135.7 | -24.94 | FALSE |
| SRX20282966 | TFs and others | ESR1 | Breast | MCF-7 | 20743 | 2512/31605 | -0.38/0.26 | -136.5 | -135.6 | -24.93 | FALSE |
| SRX21459804 | TFs and others | KAT2A | Breast | BT-549 | 19147 | 7220/31605 | -0.12/0.26 | -136.3 | -135.4 | -24.92 | FALSE |
| SRX730430 | TFs and others | NCAPG | Breast | MCF-7 | 2531 | 297/31605 | -1.60/0.26 | -136.2 | -135.3 | -24.91 | FALSE |
| SRX3110722 | TFs and others | ESR1 | Breast | MCF-7 | 14063 | 2238/31605 | -0.42/0.26 | -136.1 | -135.2 | -24.90 | FALSE |
| SRX10971494 | TFs and others | ESR1 | Breast | MCF-7 | 3472 | 1007/31605 | -0.75/0.26 | -136.1 | -135.2 | -24.90 | FALSE |
| SRX8080564 | TFs and others | Epitope tags | Breast | Mammary gland adenocarcinoma | 20725 | 4874/31605 | -0.20/0.26 | -136.0 | -135.1 | -24.89 | FALSE |
| SRX10468620 | TFs and others | ESR1 | Breast | Breast cancer cells | 12438 | 3456/31605 | -0.29/0.26 | -135.9 | -135.0 | -24.88 | FALSE |
| SRX2194282 | TFs and others | BRD4 | Breast | SUM 159PT | 36789 | 10243/31605 | -0.06/0.26 | -135.9 | -134.9 | -24.88 | FALSE |
| SRX5323812 | TFs and others | ESR1 | Breast | T-47D | 3247 | 481/31605 | -1.20/0.26 | -135.8 | -134.9 | -24.87 | FALSE |
| SRX250092 | TFs and others | AR | Breast | MDA-MB-453 | 57402 | 12700/31605 | -0.03/0.26 | -135.8 | -134.8 | -24.87 | FALSE |
| SRX5089445 | TFs and others | ESR1 | Breast | MCF-7 | 18903 | 2509/31605 | -0.38/0.26 | -135.6 | -134.7 | -24.85 | FALSE |
| SRX1758495 | TFs and others | NELFE | Breast | MCF-7 | 5730 | 5136/31605 | -0.19/0.26 | -135.6 | -134.6 | -24.85 | FALSE |
| SRX21459779 | TFs and others | MYC | Breast | BT-549 | 17870 | 11183/31605 | -0.05/0.26 | -135.5 | -134.6 | -24.85 | FALSE |
| SRX5089458 | TFs and others | ESR1 | Breast | MCF-7 | 16279 | 3010/31605 | -0.32/0.26 | -135.1 | -134.2 | -24.81 | FALSE |
| SRX5089459 | TFs and others | ESR1 | Breast | MCF-7 | 16700 | 3098/31605 | -0.32/0.26 | -135.0 | -134.1 | -24.80 | FALSE |
| SRX5323810 | TFs and others | ESR1 | Breast | T-47D | 3683 | 607/31605 | -1.03/0.26 | -134.9 | -134.0 | -24.79 | FALSE |
| SRX4663584 | TFs and others | ESR1 | Breast | MCF-7 | 6875 | 371/31605 | -1.39/0.26 | -134.9 | -134.0 | -24.79 | FALSE |
| SRX10365548 | TFs and others | NCOA3 | Breast | MCF-7 | 14495 | 1030/31605 | -0.73/0.26 | -134.9 | -134.0 | -24.78 | FALSE |
| SRX747806 | TFs and others | ESR1 | Breast | MCF-7 | 5209 | 605/31605 | -1.03/0.26 | -134.8 | -133.9 | -24.78 | FALSE |
| SRX2897201 | TFs and others | ESR1 | Breast | Breast cancer cells | 10495 | 5056/31605 | -0.19/0.26 | -134.8 | -133.9 | -24.78 | FALSE |
| SRX8639782 | TFs and others | ESR1 | Breast | MDA-MB-134-VI | 5537 | 846/31605 | -0.84/0.26 | -134.7 | -133.8 | -24.77 | FALSE |
| SRX969014 | TFs and others | ESR1 | Breast | MCF-7 | 4376 | 770/31605 | -0.89/0.26 | -134.6 | -133.6 | -24.75 | FALSE |
| SRX3857006 | TFs and others | CDKN1B | Breast | MDA-MB-231 | 23100 | 11128/31605 | -0.05/0.26 | -134.5 | -133.6 | -24.75 | FALSE |
| SRX9719172 | TFs and others | ESR1 | Breast | MCF-7 | 2726 | 297/31605 | -1.58/0.26 | -134.4 | -133.5 | -24.74 | FALSE |
| SRX5457525 | TFs and others | CLOCK | Breast | MCF-7 | 7732 | 4760/31605 | -0.20/0.26 | -134.4 | -133.5 | -24.74 | FALSE |
| SRX8616999 | TFs and others | TET2 | Breast | MCF-7 | 16783 | 2209/31605 | -0.42/0.26 | -134.1 | -133.2 | -24.71 | FALSE |
| SRX9419953 | TFs and others | BRD4 | Breast | SK-BR-3 | 22788 | 9151/31605 | -0.08/0.26 | -134.1 | -133.2 | -24.71 | FALSE |
| SRX17989899 | TFs and others | SIN3A | Breast | MDA-MB-231 | 7499 | 6140/31605 | -0.15/0.26 | -134.1 | -133.2 | -24.71 | FALSE |
| SRX190319 | TFs and others | MAX | Breast | MCF-7 | 55544 | 12863/31605 | -0.02/0.26 | -134.1 | -133.2 | -24.71 | FALSE |
| SRX490434 | TFs and others | ESR1 | Breast | MCF-7 | 1940 | 222/31605 | -1.87/0.26 | -133.9 | -133.0 | -24.69 | FALSE |
| SRX190225 | TFs and others | FOXM1 | Breast | MCF-7 | 12614 | 2749/31605 | -0.35/0.26 | -133.8 | -132.9 | -24.68 | FALSE |
| SRX10365552 | TFs and others | NCOA3 | Breast | MCF-7 | 7681 | 638/31605 | -1.00/0.26 | -133.8 | -132.9 | -24.68 | FALSE |
| SRX20830451 | TFs and others | ESR1 | Breast | T-47D | 5146 | 579/31605 | -1.06/0.26 | -133.7 | -132.8 | -24.68 | FALSE |
| SRX673752 | TFs and others | ESR1 | Breast | MCF-7 | 1944 | 277/31605 | -1.64/0.26 | -133.7 | -132.8 | -24.68 | FALSE |
| SRX176857 | TFs and others | ESR1 | Breast | MCF-7 | 21899 | 2399/31605 | -0.39/0.26 | -133.6 | -132.7 | -24.66 | FALSE |
| SRX5457550 | TFs and others | GATA3 | Breast | MCF-7 | 49706 | 8111/31605 | -0.10/0.26 | -133.5 | -132.6 | -24.65 | FALSE |
| SRX3229136 | TFs and others | ESR1 | Breast | Breast cancer | 5296 | 3466/31605 | -0.28/0.26 | -133.4 | -132.5 | -24.65 | FALSE |
| SRX8617003 | TFs and others | TET2 | Breast | MCF-7 | 18719 | 2385/31605 | -0.39/0.26 | -133.3 | -132.4 | -24.64 | FALSE |
| SRX5824580 | TFs and others | BRD4 | Breast | SUM 159PT | 13449 | 7263/31605 | -0.12/0.26 | -133.3 | -132.3 | -24.63 | FALSE |
| SRX4377533 | TFs and others | BRD4 | Breast | MDA-MB-231 | 49870 | 12043/31605 | -0.03/0.26 | -133.2 | -132.3 | -24.63 | FALSE |
| SRX7427510 | TFs and others | ESR1 | Breast | MCF-7 | 23290 | 5994/31605 | -0.15/0.26 | -133.2 | -132.3 | -24.63 | FALSE |
| SRX3229155 | TFs and others | ESR1 | Breast | Breast cancer | 9505 | 4318/31605 | -0.22/0.26 | -133.2 | -132.3 | -24.62 | FALSE |
| SRX16364101 | TFs and others | ESR1 | Breast | MCF-7 | 4196 | 560/31605 | -1.08/0.26 | -133.1 | -132.2 | -24.62 | FALSE |
| SRX4377556 | TFs and others | BRD4 | Breast | MDA-MB-231 | 49827 | 12043/31605 | -0.03/0.26 | -133.1 | -132.1 | -24.61 | FALSE |
| SRX795228 | TFs and others | BRD4 | Breast | SUM 159PT | 13464 | 7260/31605 | -0.11/0.26 | -132.9 | -132.0 | -24.60 | FALSE |
| SRX10468618 | TFs and others | ESR1 | Breast | Breast cancer cells | 15552 | 4189/31605 | -0.23/0.26 | -132.9 | -132.0 | -24.60 | FALSE |
| SRX2900095 | TFs and others | BAP1 | Breast | CAL-51 | 7464 | 5998/31605 | -0.15/0.26 | -132.7 | -131.8 | -24.58 | FALSE |
| SRX21459807 | TFs and others | KAT2A | Breast | BT-549 | 16322 | 6594/31605 | -0.13/0.26 | -132.4 | -131.5 | -24.55 | FALSE |
| SRX10468619 | TFs and others | ESR1 | Breast | Breast cancer cells | 12400 | 3370/31605 | -0.29/0.26 | -132.4 | -131.5 | -24.55 | FALSE |
| SRX1012630 | TFs and others | ESR1 | Breast | MCF-7 | 23310 | 2037/31605 | -0.44/0.26 | -132.1 | -131.2 | -24.52 | FALSE |
| SRX194567 | TFs and others | E2F4 | Breast | MCF-7 | 1428 | 1002/31605 | -0.74/0.26 | -132.1 | -131.2 | -24.52 | FALSE |
| SRX3541099 | TFs and others | ESR1 | Breast | MCF-7 | 2723 | 256/31605 | -1.71/0.26 | -132.0 | -131.1 | -24.52 | FALSE |
| SRX5089559 | TFs and others | BRD4 | Breast | MCF-7 | 8178 | 5368/31605 | -0.17/0.26 | -132.0 | -131.1 | -24.52 | FALSE |
| SRX21459797 | TFs and others | BRD4 | Breast | BT-549 | 21022 | 7218/31605 | -0.11/0.26 | -132.0 | -131.1 | -24.51 | FALSE |
| SRX190281 | TFs and others | HDAC2 | Breast | MCF-7 | 38413 | 9019/31605 | -0.08/0.26 | -131.8 | -130.9 | -24.50 | FALSE |
| SRX2897208 | TFs and others | ESR1 | Breast | Breast cancer cells | 44852 | 12777/31605 | -0.02/0.26 | -131.6 | -130.7 | -24.48 | FALSE |
| SRX9932052 | TFs and others | STAT5A | Breast | MCF-7 | 6744 | 4196/31605 | -0.23/0.26 | -131.6 | -130.7 | -24.48 | FALSE |
| SRX8080555 | TFs and others | Epitope tags | Breast | Mammary gland adenocarcinoma | 4941 | 349/31605 | -1.42/0.26 | -131.5 | -130.6 | -24.47 | FALSE |
| SRX10773108 | TFs and others | YY1 | Breast | MB 157 | 24128 | 10271/31605 | -0.05/0.26 | -131.4 | -130.5 | -24.46 | FALSE |
| SRX176882 | TFs and others | EP300 | Breast | MCF-7 | 14335 | 1202/31605 | -0.65/0.26 | -131.3 | -130.4 | -24.45 | FALSE |
| SRX2194285 | TFs and others | BRD4 | Breast | SUM 159PT | 44330 | 10783/31605 | -0.05/0.26 | -131.2 | -130.3 | -24.44 | FALSE |
| SRX2912055 | TFs and others | ESR1 | Breast | MCF-7 | 28522 | 1943/31605 | -0.45/0.26 | -131.2 | -130.3 | -24.44 | FALSE |
| SRX3323089 | TFs and others | CREB1 | Breast | MCF-7 | 24002 | 7274/31605 | -0.11/0.26 | -131.1 | -130.2 | -24.43 | FALSE |
| SRX176858 | TFs and others | ESR1 | Breast | MCF-7 | 24481 | 2500/31605 | -0.37/0.26 | -131.0 | -130.2 | -24.43 | FALSE |
| SRX8080549 | TFs and others | Epitope tags | Breast | Mammary gland ductal carcinoma | 30877 | 5491/31605 | -0.17/0.26 | -130.9 | -130.0 | -24.41 | FALSE |
| SRX10773107 | TFs and others | YY1 | Breast | MB 157 | 22631 | 9846/31605 | -0.06/0.26 | -130.9 | -130.0 | -24.41 | FALSE |
| SRX5089561 | TFs and others | BRD4 | Breast | MCF-7 | 9685 | 6065/31605 | -0.15/0.26 | -130.9 | -130.0 | -24.41 | FALSE |
| SRX5323875 | TFs and others | ESR1 | Breast | MCF-7 | 19728 | 2799/31605 | -0.34/0.26 | -130.9 | -130.0 | -24.41 | FALSE |
| SRX12008355 | TFs and others | CTCF | Breast | MCF 10A | 65012 | 12603/31605 | -0.02/0.26 | -130.8 | -129.9 | -24.41 | FALSE |
| SRX4377542 | TFs and others | BRD2 | Breast | MDA-MB-231 | 12327 | 8332/31605 | -0.09/0.26 | -130.7 | -129.9 | -24.40 | FALSE |
| SRX1721565 | TFs and others | KDM5A | Breast | T-47D | 5589 | 4140/31605 | -0.23/0.26 | -130.7 | -129.8 | -24.40 | FALSE |
| SRX1733715 | TFs and others | ESR1 | Breast | MCF-7 | 2554 | 601/31605 | -1.02/0.26 | -130.6 | -129.7 | -24.39 | FALSE |
| SRX7427506 | TFs and others | ESR1 | Breast | MCF-7 | 13164 | 2633/31605 | -0.35/0.26 | -130.6 | -129.7 | -24.38 | FALSE |
| SRX5089456 | TFs and others | ESR1 | Breast | MCF-7 | 15717 | 3539/31605 | -0.27/0.26 | -130.5 | -129.6 | -24.37 | FALSE |
| SRX194577 | TFs and others | NR2F2 | Breast | MCF-7 | 33404 | 9456/31605 | -0.07/0.26 | -130.5 | -129.6 | -24.37 | FALSE |
| SRX10921876 | TFs and others | HDAC1 | Breast | MCF-7 | 7128 | 5265/31605 | -0.17/0.26 | -130.4 | -129.5 | -24.36 | FALSE |
| SRX7366778 | TFs and others | ZMYND8 | Breast | MCF-7 | 11934 | 6092/31605 | -0.14/0.26 | -130.2 | -129.4 | -24.35 | FALSE |
| SRX2194279 | TFs and others | BRD4 | Breast | SUM 159PT | 51071 | 11755/31605 | -0.03/0.26 | -130.2 | -129.3 | -24.34 | FALSE |
| SRX11120893 | TFs and others | MAU2 | Breast | MCF-7 | 26397 | 4795/31605 | -0.19/0.26 | -130.1 | -129.2 | -24.34 | FALSE |
| SRX3229121 | TFs and others | ESR1 | Breast | Breast cancer | 6777 | 3287/31605 | -0.29/0.26 | -130.1 | -129.2 | -24.34 | FALSE |
| SRX8861817 | TFs and others | ESR1 | Breast | T-47D | 16708 | 3569/31605 | -0.27/0.26 | -130.1 | -129.2 | -24.34 | FALSE |
| SRX3322305 | TFs and others | CREB1 | Breast | MCF-7 | 20273 | 6976/31605 | -0.12/0.26 | -130.0 | -129.2 | -24.33 | FALSE |
| SRX673746 | TFs and others | EP300 | Breast | MCF-7 | 17418 | 8234/31605 | -0.09/0.26 | -130.0 | -129.1 | -24.32 | FALSE |
| SRX5323809 | TFs and others | ESR1 | Breast | T-47D | 2391 | 296/31605 | -1.56/0.26 | -130.0 | -129.1 | -24.32 | FALSE |
| SRX13340680 | TFs and others | Epitope tags | Breast | T-47D | 21790 | 6371/31605 | -0.14/0.26 | -129.9 | -129.1 | -24.32 | FALSE |
| SRX176856 | TFs and others | ESR1 | Breast | MCF-7 | 23616 | 2020/31605 | -0.44/0.26 | -129.9 | -129.0 | -24.31 | FALSE |
| SRX8537720 | TFs and others | ESR1 | Breast | T-47D | 3408 | 436/31605 | -1.24/0.26 | -129.8 | -129.0 | -24.31 | FALSE |
| SRX8080560 | TFs and others | Epitope tags | Breast | Mammary gland adenocarcinoma | 18045 | 4545/31605 | -0.21/0.26 | -129.7 | -128.8 | -24.30 | FALSE |
| SRX3322533 | TFs and others | ZNF217 | Breast | MCF-7 | 10023 | 4426/31605 | -0.21/0.26 | -129.6 | -128.7 | -24.29 | FALSE |
| SRX3123823 | TFs and others | ESR1 | Breast | MCF-7 | 22898 | 2189/31605 | -0.41/0.26 | -129.6 | -128.7 | -24.29 | FALSE |
| SRX6712575 | TFs and others | CTCF | Breast | MCF-7 | 10907 | 7953/31605 | -0.09/0.26 | -129.4 | -128.5 | -24.27 | FALSE |
| SRX1469951 | TFs and others | ESR1 | Breast | MCF-7 | 28094 | 3006/31605 | -0.31/0.26 | -129.3 | -128.5 | -24.26 | FALSE |
| SRX14101933 | TFs and others | NELFE | Breast | MCF-7 | 11085 | 7659/31605 | -0.10/0.26 | -129.3 | -128.4 | -24.26 | FALSE |
| SRX8861822 | TFs and others | ESR1 | Breast | T-47D | 4204 | 727/31605 | -0.90/0.26 | -129.3 | -128.4 | -24.26 | FALSE |
| SRX14733326 | TFs and others | ESR1 | Breast | MCF-7 | 21063 | 4565/31605 | -0.20/0.26 | -129.2 | -128.4 | -24.26 | FALSE |
| SRX5534854 | TFs and others | ESR1 | Breast | MCF-7 | 3543 | 530/31605 | -1.09/0.26 | -129.2 | -128.3 | -24.25 | FALSE |
| SRX673743 | TFs and others | EP300 | Breast | MCF-7 | 18543 | 9519/31605 | -0.06/0.26 | -129.1 | -128.2 | -24.24 | FALSE |
| SRX13991434 | TFs and others | MAFB | Breast | MCF 10A | 7191 | 6115/31605 | -0.14/0.26 | -129.1 | -128.2 | -24.24 | FALSE |
| SRX5089448 | TFs and others | ESR1 | Breast | MCF-7 | 20246 | 2686/31605 | -0.34/0.26 | -129.1 | -128.2 | -24.24 | FALSE |
| SRX5465793 | TFs and others | ESR1 | Breast | MCF-7 | 6345 | 606/31605 | -1.01/0.26 | -129.0 | -128.1 | -24.23 | FALSE |
| SRX2194263 | TFs and others | BRD4 | Breast | SUM 159PT | 50054 | 11624/31605 | -0.03/0.26 | -129.0 | -128.1 | -24.23 | FALSE |
| SRX2922226 | TFs and others | ESR1 | Breast | MCF-7 | 4160 | 619/31605 | -0.99/0.26 | -129.0 | -128.1 | -24.23 | FALSE |
| SRX673710 | TFs and others | ESR1 | Breast | MCF-7 | 21110 | 2137/31605 | -0.42/0.26 | -128.9 | -128.1 | -24.23 | FALSE |
| SRX261273 | TFs and others | ESR1 | Breast | MCF-7 | 21151 | 2149/31605 | -0.41/0.26 | -128.9 | -128.1 | -24.23 | FALSE |
| SRX1012628 | TFs and others | ESR1 | Breast | MCF-7 | 26090 | 2914/31605 | -0.32/0.26 | -128.7 | -127.8 | -24.21 | FALSE |
| SRX5323888 | TFs and others | STAT3 | Breast | T-47D | 44566 | 11276/31605 | -0.04/0.26 | -128.4 | -127.5 | -24.17 | FALSE |
| SRX4116034 | TFs and others | ESR1 | Breast | Breast cancer cells | 6581 | 3321/31605 | -0.28/0.26 | -128.1 | -127.2 | -24.15 | FALSE |
| SRX10773085 | TFs and others | TCF7 | Breast | MB 157 | 14508 | 7239/31605 | -0.11/0.26 | -128.0 | -127.2 | -24.14 | FALSE |
| SRX20282946 | TFs and others | ESR1 | Breast | MCF-7 | 30096 | 3461/31605 | -0.27/0.26 | -127.9 | -127.0 | -24.13 | FALSE |
| SRX8616964 | TFs and others | TET2 | Breast | MCF-7 | 2092 | 235/31605 | -1.76/0.26 | -127.8 | -126.9 | -24.12 | FALSE |
| SRX1469949 | TFs and others | ESR1 | Breast | MCF-7 | 29494 | 3104/31605 | -0.30/0.26 | -127.7 | -126.9 | -24.11 | FALSE |
| SRX5323876 | TFs and others | ESR1 | Breast | MCF-7 | 23870 | 3774/31605 | -0.25/0.26 | -127.5 | -126.7 | -24.09 | FALSE |
| SRX8080554 | TFs and others | Epitope tags | Breast | Mammary gland adenocarcinoma | 5560 | 437/31605 | -1.22/0.26 | -127.4 | -126.5 | -24.08 | FALSE |
| SRX10773106 | TFs and others | YY1 | Breast | MB 157 | 33962 | 11244/31605 | -0.04/0.26 | -127.4 | -126.5 | -24.08 | FALSE |
| SRX8616987 | TFs and others | TET2 | Breast | STG195 | 2824 | 354/31605 | -1.38/0.26 | -127.4 | -126.5 | -24.08 | FALSE |
| SRX1012631 | TFs and others | ESR1 | Breast | MCF-7 | 23957 | 2398/31605 | -0.37/0.26 | -127.2 | -126.3 | -24.06 | FALSE |
| SRX2194272 | TFs and others | BRD4 | Breast | SUM 159PT | 51811 | 11298/31605 | -0.03/0.26 | -127.1 | -126.3 | -24.05 | FALSE |
| SRX8080569 | TFs and others | POU2F1 | Breast | Mammary gland ductal carcinoma | 18473 | 11211/31605 | -0.04/0.26 | -127.1 | -126.2 | -24.05 | FALSE |
| SRX14733330 | TFs and others | ESR1 | Breast | MCF-7 | 25863 | 5708/31605 | -0.15/0.26 | -127.0 | -126.1 | -24.04 | FALSE |
| SRX330068 | TFs and others | ESR1 | Breast | MCF-7 | 26874 | 2312/31605 | -0.39/0.26 | -127.0 | -126.1 | -24.04 | FALSE |
| SRX330070 | TFs and others | ESR1 | Breast | MCF-7 | 30522 | 2847/31605 | -0.32/0.26 | -126.9 | -126.1 | -24.04 | FALSE |
| SRX3110723 | TFs and others | ESR1 | Breast | MCF-7 | 29155 | 3485/31605 | -0.27/0.26 | -126.9 | -126.1 | -24.03 | FALSE |
| SRX2194264 | TFs and others | BRD4 | Breast | SUM 159PT | 54754 | 11524/31605 | -0.03/0.26 | -126.9 | -126.0 | -24.03 | FALSE |
| SRX9719171 | TFs and others | ESR1 | Breast | MCF-7 | 14994 | 1873/31605 | -0.46/0.26 | -126.7 | -125.9 | -24.02 | FALSE |
| SRX1131571 | TFs and others | BRD4 | Breast | T-47D | 11263 | 4788/31605 | -0.19/0.26 | -126.7 | -125.9 | -24.01 | FALSE |
| SRX5089451 | TFs and others | ESR1 | Breast | MCF-7 | 20499 | 3903/31605 | -0.24/0.26 | -126.4 | -125.5 | -23.98 | FALSE |
| SRX666559 | TFs and others | NRF1 | Breast | T-47D | 10797 | 7243/31605 | -0.11/0.26 | -126.4 | -125.5 | -23.98 | FALSE |
| SRX14733320 | TFs and others | ESR1 | Breast | MCF-7 | 26844 | 5955/31605 | -0.14/0.26 | -126.3 | -125.4 | -23.97 | FALSE |
| SRX8537723 | TFs and others | ESR1 | Breast | MCF-7 | 34757 | 4370/31605 | -0.21/0.26 | -126.3 | -125.4 | -23.97 | FALSE |
| SRX2424330 | TFs and others | HCFC1 | Breast | MCF-7 | 6479 | 4698/31605 | -0.19/0.26 | -126.2 | -125.4 | -23.97 | FALSE |
| SRX128101 | TFs and others | TFAP2C | Breast | MDA-MB-453 | 31863 | 8865/31605 | -0.07/0.26 | -126.1 | -125.3 | -23.96 | FALSE |
| SRX2194262 | TFs and others | BRD4 | Breast | SUM 159PT | 60203 | 11815/31605 | -0.03/0.26 | -126.1 | -125.2 | -23.96 | FALSE |
| SRX5089449 | TFs and others | ESR1 | Breast | MCF-7 | 23782 | 3208/31605 | -0.29/0.26 | -126.0 | -125.2 | -23.95 | FALSE |
| SRX747805 | TFs and others | ESR1 | Breast | MCF-7 | 12474 | 1318/31605 | -0.59/0.26 | -126.0 | -125.2 | -23.95 | FALSE |
| SRX8639781 | TFs and others | ESR1 | Breast | MDA-MB-134-VI | 2173 | 319/31605 | -1.46/0.26 | -126.0 | -125.1 | -23.94 | FALSE |
| SRX1161199 | TFs and others | ESR1 | Breast | T-47D | 10898 | 3734/31605 | -0.25/0.26 | -125.9 | -125.1 | -23.94 | FALSE |
| SRX21459805 | TFs and others | KAT2A | Breast | BT-549 | 15671 | 6204/31605 | -0.13/0.26 | -125.9 | -125.0 | -23.94 | FALSE |
| SRX7282935 | TFs and others | ESR1 | Breast | ZR-75-1 | 5313 | 401/31605 | -1.28/0.26 | -125.9 | -125.0 | -23.93 | FALSE |
| SRX5089455 | TFs and others | ESR1 | Breast | MCF-7 | 8517 | 2678/31605 | -0.34/0.26 | -125.8 | -125.0 | -23.93 | FALSE |
| SRX250093 | TFs and others | FOXA1 | Breast | MDA-MB-453 | 91694 | 13263/31605 | -0.01/0.26 | -125.8 | -124.9 | -23.92 | FALSE |
| SRX10164643 | TFs and others | ESR1 | Breast | MCF-7 | 9742 | 746/31605 | -0.87/0.26 | -125.6 | -124.8 | -23.91 | FALSE |
| SRX8617023 | TFs and others | ESR1 | Breast | ZR-75-1 | 17006 | 2822/31605 | -0.32/0.26 | -125.5 | -124.6 | -23.90 | FALSE |
| SRX20830446 | TFs and others | ESR1 | Breast | T-47D | 4851 | 551/31605 | -1.05/0.26 | -125.4 | -124.6 | -23.89 | FALSE |
| SRX3857001 | TFs and others | CDKN1B | Breast | MDA-MB-231 | 35842 | 12518/31605 | -0.02/0.26 | -125.3 | -124.5 | -23.88 | FALSE |
| SRX9419961 | TFs and others | MED1 | Breast | SK-BR-3 | 28619 | 8759/31605 | -0.07/0.26 | -125.3 | -124.5 | -23.88 | FALSE |
| SRX8080571 | TFs and others | POU2F1 | Breast | Mammary gland ductal carcinoma | 16346 | 10309/31605 | -0.05/0.26 | -125.3 | -124.4 | -23.88 | FALSE |
| SRX5089454 | TFs and others | ESR1 | Breast | MCF-7 | 18548 | 3894/31605 | -0.23/0.26 | -125.2 | -124.4 | -23.87 | FALSE |
| SRX2881009 | TFs and others | ESR1 | Breast | MCF-7 | 2883 | 289/31605 | -1.54/0.26 | -125.1 | -124.3 | -23.86 | FALSE |
| SRX5323892 | TFs and others | STAT3 | Breast | T-47D | 47495 | 11386/31605 | -0.03/0.26 | -125.0 | -124.1 | -23.85 | FALSE |
| SRX8537718 | TFs and others | ESR1 | Breast | SUM 44PE | 36177 | 4045/31605 | -0.23/0.26 | -125.0 | -124.1 | -23.85 | FALSE |
| SRX8639778 | TFs and others | ESR1 | Breast | MDA-MB-134-VI | 2271 | 335/31605 | -1.41/0.26 | -124.8 | -124.0 | -23.84 | FALSE |
| SRX3285553 | TFs and others | ESR1 | Breast | C49R | 4322 | 429/31605 | -1.22/0.26 | -124.8 | -124.0 | -23.83 | FALSE |
| SRX666557 | TFs and others | ARNT | Breast | T-47D | 58883 | 13151/31605 | -0.01/0.26 | -124.8 | -124.0 | -23.83 | FALSE |
| SRX14733321 | TFs and others | ESR1 | Breast | MCF-7 | 24469 | 5567/31605 | -0.15/0.26 | -124.8 | -123.9 | -23.83 | FALSE |
| SRX469396 | TFs and others | ESR1 | Breast | MCF-7 | 8062 | 935/31605 | -0.74/0.26 | -124.7 | -123.9 | -23.82 | FALSE |
| SRX8537719 | TFs and others | ESR1 | Breast | SUM 44PE | 37133 | 4121/31605 | -0.22/0.26 | -124.7 | -123.8 | -23.82 | FALSE |
| SRX9109113 | TFs and others | AR | Breast | MDA-MB-453 | 80522 | 11437/31605 | -0.03/0.26 | -124.6 | -123.8 | -23.81 | FALSE |
| SRX389294 | TFs and others | XBP1 | Breast | T-47D | 10564 | 5096/31605 | -0.17/0.26 | -124.5 | -123.7 | -23.80 | FALSE |
| SRX323399 | TFs and others | ESR1 | Breast | MCF-7 | 18086 | 3021/31605 | -0.30/0.26 | -124.4 | -123.6 | -23.80 | FALSE |
| SRX11867601 | TFs and others | KMT2C | Breast | CAL-51 | 11334 | 6550/31605 | -0.12/0.26 | -124.4 | -123.5 | -23.79 | FALSE |
| SRX14733331 | TFs and others | ESR1 | Breast | MCF-7 | 22470 | 5261/31605 | -0.16/0.26 | -124.3 | -123.4 | -23.78 | FALSE |
| SRX3322265 | TFs and others | ELF1 | Breast | MCF-7 | 8763 | 5431/31605 | -0.16/0.26 | -123.9 | -123.0 | -23.74 | FALSE |
| SRX5323900 | TFs and others | STAT3 | Breast | T-47D | 51363 | 11343/31605 | -0.03/0.26 | -123.8 | -123.0 | -23.73 | FALSE |
| SRX3322646 | TFs and others | NCOA3 | Breast | MCF-7 | 5269 | 411/31605 | -1.24/0.26 | -123.8 | -122.9 | -23.73 | FALSE |
| SRX2194289 | TFs and others | BRD4 | Breast | SUM 229PE | 19881 | 7452/31605 | -0.10/0.26 | -123.7 | -122.9 | -23.73 | FALSE |
| SRX3856999 | TFs and others | JUN | Breast | MDA-MB-231 | 35124 | 12492/31605 | -0.02/0.26 | -123.6 | -122.8 | -23.72 | FALSE |
| SRX2194271 | TFs and others | BRD4 | Breast | SUM 159PT | 53313 | 11293/31605 | -0.03/0.26 | -123.6 | -122.8 | -23.71 | FALSE |
| SRX5323896 | TFs and others | STAT3 | Breast | T-47D | 38954 | 10067/31605 | -0.05/0.26 | -123.5 | -122.7 | -23.71 | FALSE |
| SRX968962 | TFs and others | ESR1 | Breast | MCF-7 | 8902 | 3644/31605 | -0.25/0.26 | -123.4 | -122.6 | -23.70 | FALSE |
| SRX4451213 | TFs and others | ESR1 | Breast | MCF-7 | 52129 | 4863/31605 | -0.18/0.26 | -123.4 | -122.6 | -23.69 | FALSE |
| SRX3080214 | TFs and others | ESR1 | Breast | MCF-7 | 18486 | 1564/31605 | -0.51/0.26 | -123.4 | -122.6 | -23.69 | FALSE |
| SRX21459803 | TFs and others | KAT2A | Breast | BT-549 | 17955 | 5612/31605 | -0.15/0.26 | -123.3 | -122.5 | -23.69 | FALSE |
| SRX3410386 | TFs and others | BRD4 | Breast | MDA-MB-231 | 17844 | 5913/31605 | -0.14/0.26 | -123.3 | -122.4 | -23.68 | FALSE |
| SRX11120901 | TFs and others | CREBBP | Breast | MCF-7 | 16560 | 4524/31605 | -0.20/0.26 | -123.2 | -122.4 | -23.68 | FALSE |
| SRX792933 | TFs and others | ESR1 | Breast | LY2 | 51267 | 5988/31605 | -0.14/0.26 | -123.0 | -122.2 | -23.66 | FALSE |
| SRX10024474 | TFs and others | ESR1 | Breast | MCF-7 | 2943 | 260/31605 | -1.62/0.26 | -123.0 | -122.2 | -23.66 | FALSE |
| SRX4377561 | TFs and others | BRD9 | Breast | MDA-MB-231 | 45569 | 11036/31605 | -0.03/0.26 | -122.9 | -122.0 | -23.64 | FALSE |
| SRX6027740 | TFs and others | ESR1 | Breast | MCF-7 | 7241 | 2513/31605 | -0.35/0.26 | -122.7 | -121.9 | -23.63 | FALSE |
| SRX8861824 | TFs and others | ESR1 | Breast | T-47D | 4828 | 632/31605 | -0.95/0.26 | -122.7 | -121.9 | -23.63 | FALSE |
| SRX4451214 | TFs and others | ESR1 | Breast | MCF-7 | 47453 | 4628/31605 | -0.19/0.26 | -122.7 | -121.8 | -23.62 | FALSE |
| SRX2194281 | TFs and others | BRD4 | Breast | SUM 159PT | 18147 | 6970/31605 | -0.11/0.26 | -122.6 | -121.8 | -23.62 | FALSE |
| SRX265432 | TFs and others | KDM5B | Breast | HCC2157 | 52444 | 11912/31605 | -0.02/0.26 | -122.6 | -121.8 | -23.62 | FALSE |
| SRX1510972 | TFs and others | ZNF143 | Breast | MCF-7 | 11340 | 5773/31605 | -0.14/0.26 | -122.6 | -121.7 | -23.61 | FALSE |
| SRX1012625 | TFs and others | ESR1 | Breast | MCF-7 | 26711 | 2734/31605 | -0.32/0.26 | -122.5 | -121.7 | -23.61 | FALSE |
| SRX20830445 | TFs and others | ESR1 | Breast | T-47D | 4662 | 638/31605 | -0.94/0.26 | -122.5 | -121.7 | -23.61 | FALSE |
| SRX5089507 | TFs and others | HDAC1 | Breast | MCF-7 | 6897 | 5922/31605 | -0.14/0.26 | -122.5 | -121.7 | -23.61 | FALSE |
| SRX5465792 | TFs and others | ESR1 | Breast | MCF-7 | 5792 | 1123/31605 | -0.65/0.26 | -122.5 | -121.7 | -23.61 | FALSE |
| SRX5323874 | TFs and others | ESR1 | Breast | MCF-7 | 25834 | 4043/31605 | -0.22/0.26 | -122.4 | -121.6 | -23.60 | FALSE |
| SRX2585782 | TFs and others | TP53 | Breast | MDA-MB-231 | 54470 | 10604/31605 | -0.04/0.26 | -122.4 | -121.6 | -23.60 | FALSE |
| SRX2194274 | TFs and others | BRD4 | Breast | SUM 159PT | 44254 | 10838/31605 | -0.04/0.26 | -122.4 | -121.6 | -23.60 | FALSE |
| SRX4451196 | TFs and others | ESR1 | Breast | MCF-7 | 6059 | 2287/31605 | -0.38/0.26 | -122.2 | -121.3 | -23.57 | FALSE |
| SRX185907 | TFs and others | FOXM1 | Breast | MCF-7 | 17356 | 2242/31605 | -0.38/0.26 | -122.0 | -121.2 | -23.56 | FALSE |
| SRX1131549 | TFs and others | BRD4 | Breast | HCC1395 | 43672 | 9838/31605 | -0.05/0.26 | -122.0 | -121.2 | -23.56 | FALSE |
| SRX8861818 | TFs and others | ESR1 | Breast | T-47D | 33222 | 8315/31605 | -0.08/0.26 | -121.8 | -121.0 | -23.54 | FALSE |
| SRX8861821 | TFs and others | ESR1 | Breast | T-47D | 11124 | 2201/31605 | -0.39/0.26 | -121.7 | -120.9 | -23.53 | FALSE |
| SRX2943597 | TFs and others | ESR1 | Breast | MCF-7 | 5614 | 744/31605 | -0.85/0.26 | -121.7 | -120.9 | -23.53 | FALSE |
| SRX9419934 | TFs and others | MED1 | Breast | SK-BR-3 | 22228 | 7581/31605 | -0.09/0.26 | -121.6 | -120.8 | -23.52 | FALSE |
| SRX150520 | TFs and others | MYC | Breast | MCF 10A | 47042 | 10122/31605 | -0.04/0.26 | -121.4 | -120.6 | -23.50 | FALSE |
| SRX330069 | TFs and others | ESR1 | Breast | MCF-7 | 34079 | 3212/31605 | -0.28/0.26 | -121.4 | -120.6 | -23.50 | FALSE |
| SRX5287690 | TFs and others | ESR1 | Breast | T-47D | 3029 | 462/31605 | -1.14/0.26 | -121.4 | -120.5 | -23.50 | FALSE |
| SRX026532 | TFs and others | ESR1 | Breast | T-47D | 2388 | 276/31605 | -1.56/0.26 | -121.3 | -120.5 | -23.49 | FALSE |
| SRX4374940 | TFs and others | RBPJ | Breast | HCC1599 | 26639 | 10820/31605 | -0.03/0.26 | -121.2 | -120.4 | -23.49 | FALSE |
| SRX9109110 | TFs and others | AR | Breast | MDA-MB-453 | 81661 | 11330/31605 | -0.03/0.26 | -121.2 | -120.4 | -23.48 | FALSE |
| SRX5287703 | TFs and others | ESR1 | Breast | MCF-7 | 32073 | 3288/31605 | -0.27/0.26 | -121.2 | -120.4 | -23.48 | FALSE |
| SRX1280452 | TFs and others | NRF1 | Breast | HCC1955 | 15554 | 9474/31605 | -0.05/0.26 | -121.1 | -120.3 | -23.47 | FALSE |
| SRX730453 | TFs and others | ESR1 | Breast | MCF-7 | 2924 | 231/31605 | -1.72/0.26 | -121.0 | -120.2 | -23.47 | FALSE |
| SRX5089462 | TFs and others | ESR1 | Breast | MCF-7 | 22686 | 4374/31605 | -0.20/0.26 | -121.0 | -120.2 | -23.46 | FALSE |
| SRX20282959 | TFs and others | ESR1 | Breast | MCF-7 | 22465 | 2344/31605 | -0.37/0.26 | -121.0 | -120.2 | -23.46 | FALSE |
| SRX13340675 | TFs and others | Epitope tags | Breast | T-47D | 16184 | 5837/31605 | -0.14/0.26 | -120.9 | -120.1 | -23.45 | FALSE |
| SRX185911 | TFs and others | FOXM1 | Breast | MDA-MB-231 | 4779 | 2739/31605 | -0.32/0.26 | -120.8 | -120.0 | -23.45 | FALSE |
| SRX9932057 | TFs and others | STAT5A | Breast | MCF-7 | 7583 | 4328/31605 | -0.20/0.26 | -120.7 | -119.9 | -23.43 | FALSE |
| SRX4377560 | TFs and others | BRD9 | Breast | MDA-MB-231 | 37261 | 10075/31605 | -0.04/0.26 | -120.6 | -119.8 | -23.42 | FALSE |
| SRX186807 | TFs and others | ESR1 | Breast | Breast cancer | 2618 | 548/31605 | -1.03/0.26 | -120.5 | -119.7 | -23.41 | FALSE |
| SRX4377535 | TFs and others | BRD4 | Breast | MDA-MB-231 | 30449 | 9789/31605 | -0.05/0.26 | -120.5 | -119.7 | -23.41 | FALSE |
| SRX6757656 | TFs and others | ESR1 | Breast | MCF-7 | 2398 | 200/31605 | -1.86/0.26 | -120.4 | -119.6 | -23.41 | FALSE |
| SRX2020842 | TFs and others | STAT3 | Breast | HCC70 | 65012 | 10762/31605 | -0.03/0.26 | -120.4 | -119.6 | -23.40 | FALSE |
| SRX673749 | TFs and others | MED1 | Breast | MCF-7 | 9266 | 5758/31605 | -0.14/0.26 | -120.3 | -119.5 | -23.39 | FALSE |
| SRX21459806 | TFs and others | KAT2A | Breast | BT-549 | 11297 | 4873/31605 | -0.17/0.26 | -120.2 | -119.4 | -23.38 | FALSE |
| SRX8080567 | TFs and others | POU2F1 | Breast | Mammary gland ductal carcinoma | 13296 | 9012/31605 | -0.06/0.26 | -119.9 | -119.1 | -23.35 | FALSE |
| SRX968963 | TFs and others | ESR1 | Breast | MCF-7 | 27991 | 2737/31605 | -0.32/0.26 | -119.8 | -119.0 | -23.34 | FALSE |
| SRX1280451 | TFs and others | NRF1 | Breast | HCC1954 | 18149 | 10071/31605 | -0.04/0.26 | -119.8 | -119.0 | -23.34 | FALSE |
| SRX10773104 | TFs and others | YY1 | Breast | MB 157 | 23927 | 10106/31605 | -0.04/0.26 | -119.7 | -118.9 | -23.34 | FALSE |
| SRX1012632 | TFs and others | ESR1 | Breast | MCF-7 | 26985 | 2336/31605 | -0.36/0.26 | -119.7 | -118.9 | -23.33 | FALSE |
| SRX1012740 | TFs and others | ESR1 | Breast | MCF-7 | 1869 | 206/31605 | -1.83/0.26 | -119.5 | -118.7 | -23.32 | FALSE |
| SRX371475 | TFs and others | ESR1 | Breast | BT-474 | 31124 | 3564/31605 | -0.24/0.26 | -119.5 | -118.7 | -23.31 | FALSE |
| SRX4451212 | TFs and others | ESR1 | Breast | MCF-7 | 50279 | 4423/31605 | -0.19/0.26 | -119.4 | -118.6 | -23.31 | FALSE |
| SRX4193141 | TFs and others | ESR1 | Breast | MCF-7 | 5530 | 2347/31605 | -0.36/0.26 | -119.4 | -118.6 | -23.30 | FALSE |
| SRX194572 | TFs and others | ESR1 | Breast | MCF-7 | 34645 | 2991/31605 | -0.29/0.26 | -119.4 | -118.6 | -23.30 | FALSE |
| SRX20830443 | TFs and others | ESR1 | Breast | T-47D | 4838 | 713/31605 | -0.86/0.26 | -119.3 | -118.5 | -23.30 | FALSE |
| SRX20830444 | TFs and others | ESR1 | Breast | T-47D | 4568 | 516/31605 | -1.06/0.26 | -119.3 | -118.5 | -23.30 | FALSE |
| SRX1758507 | TFs and others | NELFE | Breast | MCF-7 | 5379 | 4773/31605 | -0.18/0.26 | -119.3 | -118.5 | -23.30 | FALSE |
| SRX2194290 | TFs and others | BRD4 | Breast | SUM 229PE | 23585 | 8353/31605 | -0.07/0.26 | -119.3 | -118.5 | -23.29 | FALSE |
| SRX8080565 | TFs and others | Epitope tags | Breast | Mammary gland adenocarcinoma | 24706 | 2585/31605 | -0.33/0.26 | -119.3 | -118.5 | -23.29 | FALSE |
| SRX4377558 | TFs and others | BRD4 | Breast | MDA-MB-231 | 30415 | 9775/31605 | -0.05/0.26 | -119.3 | -118.5 | -23.29 | FALSE |
| SRX17989900 | TFs and others | SIN3A | Breast | MDA-MB-231 | 6213 | 5258/31605 | -0.16/0.26 | -119.1 | -118.3 | -23.27 | FALSE |
| SRX3990996 | TFs and others | PGR | Breast | T-47D | 40317 | 7536/31605 | -0.09/0.26 | -119.0 | -118.2 | -23.26 | FALSE |
| SRX10773105 | TFs and others | YY1 | Breast | MB 157 | 36566 | 11722/31605 | -0.02/0.26 | -119.0 | -118.2 | -23.26 | FALSE |
| SRX2881001 | TFs and others | ESR1 | Breast | MCF-7 | 26925 | 3670/31605 | -0.24/0.26 | -118.9 | -118.1 | -23.25 | FALSE |
| SRX3856998 | TFs and others | JUN | Breast | MDA-MB-231 | 38823 | 12803/31605 | -0.01/0.26 | -118.9 | -118.1 | -23.25 | FALSE |
| SRX10775024 | TFs and others | ESR1 | Breast | T-47D | 5570 | 926/31605 | -0.72/0.26 | -118.8 | -118.1 | -23.25 | FALSE |
| SRX4786555 | TFs and others | KMT2C | Breast | MCF-7 | 15132 | 4833/31605 | -0.17/0.26 | -118.8 | -118.0 | -23.24 | FALSE |
| SRX11169859 | TFs and others | ESR1 | Breast | MCF-7 | 5556 | 2348/31605 | -0.36/0.26 | -118.6 | -117.8 | -23.22 | FALSE |
| SRX19024876 | TFs and others | ESR1 | Breast | MCF-7 | 2794 | 214/31605 | -1.78/0.26 | -118.4 | -117.6 | -23.21 | FALSE |
| SRX9045669 | TFs and others | ESR1 | Breast | MCF-7 | 3138 | 611/31605 | -0.95/0.26 | -118.4 | -117.6 | -23.20 | FALSE |
| SRX8616982 | TFs and others | TET2 | Breast | ZR-75-1 | 3230 | 316/31605 | -1.42/0.26 | -118.3 | -117.5 | -23.20 | FALSE |
| SRX8537722 | TFs and others | ESR1 | Breast | MCF-7 | 38551 | 4752/31605 | -0.18/0.26 | -118.3 | -117.5 | -23.20 | FALSE |
| SRX371468 | TFs and others | ESR1 | Breast | MCF-7 | 34282 | 2990/31605 | -0.29/0.26 | -118.0 | -117.2 | -23.17 | FALSE |
| SRX5323838 | TFs and others | STAT3 | Breast | T-47D | 30726 | 8985/31605 | -0.06/0.26 | -118.0 | -117.2 | -23.16 | FALSE |
| SRX8080552 | TFs and others | Epitope tags | Breast | Mammary gland ductal carcinoma | 26810 | 3151/31605 | -0.27/0.26 | -117.9 | -117.1 | -23.15 | FALSE |
| SRX2423719 | TFs and others | MAZ | Breast | MCF-7 | 8548 | 5617/31605 | -0.14/0.26 | -117.8 | -117.1 | -23.15 | FALSE |
| SRX5323878 | TFs and others | ESR1 | Breast | MCF-7 | 36751 | 6166/31605 | -0.12/0.26 | -117.8 | -117.0 | -23.15 | FALSE |
| SRX3857000 | TFs and others | CDKN1B | Breast | MDA-MB-231 | 39785 | 12854/31605 | -0.01/0.26 | -117.6 | -116.8 | -23.12 | FALSE |
| SRX1161174 | TFs and others | FOXA1 | Breast | MCF-7 | 43946 | 7036/31605 | -0.10/0.26 | -117.3 | -116.6 | -23.10 | FALSE |
| SRX2194257 | TFs and others | BRD4 | Breast | HCC1806 | 17638 | 6465/31605 | -0.11/0.26 | -117.3 | -116.6 | -23.10 | FALSE |
| SRX8617002 | TFs and others | TET2 | Breast | MCF-7 | 11210 | 1627/31605 | -0.48/0.26 | -117.3 | -116.6 | -23.10 | FALSE |
| SRX10773103 | TFs and others | YY1 | Breast | MB 157 | 25833 | 10415/31605 | -0.03/0.26 | -117.1 | -116.3 | -23.08 | FALSE |
| SRX5089555 | TFs and others | BRD4 | Breast | MCF-7 | 8782 | 5194/31605 | -0.15/0.26 | -117.1 | -116.3 | -23.07 | FALSE |
| SRX5323877 | TFs and others | ESR1 | Breast | MCF-7 | 32247 | 4718/31605 | -0.17/0.26 | -117.0 | -116.2 | -23.07 | FALSE |
| SRX2897220 | TFs and others | ESR1 | Breast | Breast cancer cells | 23170 | 11760/31605 | -0.02/0.26 | -117.0 | -116.2 | -23.07 | FALSE |
| SRX3123829 | TFs and others | ESR1 | Breast | MCF-7 | 29103 | 2912/31605 | -0.29/0.26 | -117.0 | -116.2 | -23.06 | FALSE |
| SRX5534831 | TFs and others | ESR1 | Breast | MCF-7 | 37399 | 5668/31605 | -0.14/0.26 | -116.9 | -116.2 | -23.06 | FALSE |
| SRX9932075 | TFs and others | HDAC6 | Breast | MCF-7 | 1990 | 218/31605 | -1.74/0.26 | -116.8 | -116.0 | -23.05 | FALSE |
| SRX469397 | TFs and others | ESR1 | Breast | MCF-7 | 4501 | 596/31605 | -0.95/0.26 | -116.5 | -115.7 | -23.02 | FALSE |
| SRX1758505 | TFs and others | NELFE | Breast | MCF-7 | 4909 | 4451/31605 | -0.19/0.26 | -116.5 | -115.7 | -23.01 | FALSE |
| SRX3554402 | TFs and others | ESR1 | Breast | MDA-MB-134-VI | 19233 | 2262/31605 | -0.36/0.26 | -116.4 | -115.6 | -23.01 | FALSE |
| SRX1161200 | TFs and others | ESR1 | Breast | T-47D | 16833 | 2401/31605 | -0.35/0.26 | -116.0 | -115.2 | -22.97 | FALSE |
| SRX16364103 | TFs and others | ESR1 | Breast | MCF-7 | 3830 | 573/31605 | -0.97/0.26 | -115.9 | -115.2 | -22.96 | FALSE |
| SRX3856997 | TFs and others | JUN | Breast | MDA-MB-231 | 48032 | 13008/31605 | -0.00/0.26 | -115.9 | -115.1 | -22.96 | FALSE |
| SRX371477 | TFs and others | ESR1 | Breast | MCF-7 | 31860 | 2686/31605 | -0.31/0.26 | -115.7 | -115.0 | -22.94 | FALSE |
| SRX8616988 | TFs and others | TET2 | Breast | AB555 | 3543 | 398/31605 | -1.22/0.26 | -115.6 | -114.8 | -22.93 | FALSE |
| SRX2900158 | TFs and others | KDM6A | Breast | CAL-51 | 8809 | 5666/31605 | -0.13/0.26 | -115.5 | -114.8 | -22.92 | FALSE |
| SRX2897217 | TFs and others | ESR1 | Breast | Breast cancer cells | 9792 | 8350/31605 | -0.07/0.26 | -115.5 | -114.7 | -22.92 | FALSE |
| SRX6853598 | TFs and others | ARNT | Breast | MCF-7 | 10579 | 5307/31605 | -0.15/0.26 | -115.5 | -114.7 | -22.91 | FALSE |
| SRX10164625 | TFs and others | ESR1 | Breast | MCF-7 | 5148 | 413/31605 | -1.19/0.26 | -115.3 | -114.6 | -22.90 | FALSE |
| SRX265413 | TFs and others | KDM5B | Breast | MCF-7 | 44108 | 12387/31605 | -0.01/0.26 | -115.3 | -114.5 | -22.90 | FALSE |
| SRX8080558 | TFs and others | Epitope tags | Breast | Mammary gland adenocarcinoma | 22181 | 1589/31605 | -0.48/0.26 | -115.3 | -114.5 | -22.90 | FALSE |
| SRX747801 | TFs and others | ESR1 | Breast | MCF-7 | 12027 | 2328/31605 | -0.35/0.26 | -115.1 | -114.3 | -22.88 | FALSE |
| SRX12008351 | TFs and others | CTCF | Breast | MCF 10A | 27314 | 13274/31605 | 0.00/0.26 | -114.9 | -114.2 | -22.86 | FALSE |
| SRX265430 | TFs and others | KDM5B | Breast | MDA-MB-231 | 51879 | 12697/31605 | -0.00/0.26 | -114.9 | -114.1 | -22.85 | FALSE |
| SRX5534843 | TFs and others | ESR1 | Breast | MCF-7 | 5642 | 706/31605 | -0.85/0.26 | -114.8 | -114.0 | -22.85 | FALSE |
| SRX3229163 | TFs and others | ESR1 | Breast | Breast cancer | 9215 | 1725/31605 | -0.45/0.26 | -114.8 | -114.0 | -22.84 | FALSE |
| SRX2513521 | TFs and others | ESR1 | Breast | MCF-7 | 27824 | 2726/31605 | -0.30/0.26 | -114.6 | -113.9 | -22.83 | FALSE |
| SRX969013 | TFs and others | ESR1 | Breast | MCF-7 | 2183 | 407/31605 | -1.19/0.26 | -114.6 | -113.8 | -22.82 | FALSE |
| SRX3110719 | TFs and others | ESR1 | Breast | MCF-7 | 4287 | 930/31605 | -0.70/0.26 | -114.5 | -113.8 | -22.82 | FALSE |
| SRX16874138 | TFs and others | ESR1 | Breast | MCF-7 | 2685 | 253/31605 | -1.58/0.26 | -114.5 | -113.8 | -22.82 | FALSE |
| SRX3857005 | TFs and others | CDKN1B | Breast | MDA-MB-231 | 49101 | 13092/31605 | -0.00/0.26 | -114.5 | -113.7 | -22.82 | FALSE |
| SRX3856993 | TFs and others | JUN | Breast | MDA-MB-231 | 46720 | 12898/31605 | -0.00/0.26 | -114.5 | -113.7 | -22.81 | FALSE |
| SRX8537740 | TFs and others | FOXA1 | Breast | SUM 44PE | 102863 | 9397/31605 | -0.05/0.26 | -114.4 | -113.7 | -22.81 | FALSE |
| SRX4374942 | TFs and others | RBPJ | Breast | HCC1599 | 23234 | 11142/31605 | -0.02/0.26 | -114.4 | -113.6 | -22.80 | FALSE |
| SRX10365536 | TFs and others | ESR1 | Breast | MCF-7 | 27453 | 2072/31605 | -0.39/0.26 | -114.4 | -113.6 | -22.80 | FALSE |
| SRX3229189 | TFs and others | PGR | Breast | Breast cancer | 28133 | 6671/31605 | -0.10/0.26 | -114.2 | -113.5 | -22.79 | FALSE |
| SRX20830448 | TFs and others | ESR1 | Breast | T-47D | 4445 | 431/31605 | -1.15/0.26 | -114.2 | -113.4 | -22.79 | FALSE |
| SRX194576 | TFs and others | GREB1 | Breast | MCF-7 | 1946 | 241/31605 | -1.62/0.26 | -114.0 | -113.3 | -22.77 | FALSE |
| SRX7282934 | TFs and others | ESR1 | Breast | ZR-75-1 | 5162 | 346/31605 | -1.31/0.26 | -113.9 | -113.1 | -22.75 | FALSE |
| SRX10164661 | TFs and others | ESR1 | Breast | MCF-7 | 10943 | 879/31605 | -0.73/0.26 | -113.7 | -112.9 | -22.74 | FALSE |
| SRX3229147 | TFs and others | ESR1 | Breast | Breast cancer | 4181 | 940/31605 | -0.69/0.26 | -113.7 | -112.9 | -22.73 | FALSE |
| SRX323398 | TFs and others | ESR1 | Breast | MCF-7 | 16808 | 4286/31605 | -0.19/0.26 | -113.6 | -112.9 | -22.73 | FALSE |
| SRX8861823 | TFs and others | ESR1 | Breast | T-47D | 18960 | 4186/31605 | -0.19/0.26 | -113.5 | -112.8 | -22.72 | FALSE |
| SRX673751 | TFs and others | ESR1 | Breast | MCF-7 | 1355 | 194/31605 | -1.83/0.26 | -113.4 | -112.6 | -22.70 | FALSE |
| SRX10468621 | TFs and others | ESR1 | Breast | Breast cancer cells | 8462 | 2149/31605 | -0.37/0.26 | -113.3 | -112.6 | -22.70 | FALSE |
| SRX8080556 | TFs and others | Epitope tags | Breast | Mammary gland adenocarcinoma | 9463 | 2809/31605 | -0.29/0.26 | -113.2 | -112.5 | -22.69 | FALSE |
| SRX1012640 | TFs and others | EP300 | Breast | MCF-7 | 5932 | 658/31605 | -0.88/0.26 | -113.1 | -112.3 | -22.67 | FALSE |
| SRX9719167 | TFs and others | ESR1 | Breast | MCF-7 | 22313 | 3147/31605 | -0.26/0.26 | -113.0 | -112.2 | -22.66 | FALSE |
| SRX4374941 | TFs and others | RBPJ | Breast | HCC1599 | 25142 | 11720/31605 | -0.01/0.26 | -112.9 | -112.1 | -22.65 | FALSE |
| SRX12008350 | TFs and others | CTCF | Breast | MCF 10A | 30098 | 13285/31605 | 0.00/0.26 | -112.9 | -112.1 | -22.65 | FALSE |
| SRX3856996 | TFs and others | JUN | Breast | MDA-MB-231 | 51114 | 13161/31605 | 0.00/0.26 | -112.9 | -112.1 | -22.65 | FALSE |
| SRX1012633 | TFs and others | ESR1 | Breast | MCF-7 | 26329 | 2532/31605 | -0.32/0.26 | -112.6 | -111.9 | -22.63 | FALSE |
| SRX1531791 | TFs and others | ESR1 | Breast | MCF-7 | 46550 | 4744/31605 | -0.17/0.26 | -112.6 | -111.9 | -22.63 | FALSE |
| SRX7427508 | TFs and others | ESR1 | Breast | MCF-7 | 24325 | 3547/31605 | -0.23/0.26 | -112.6 | -111.8 | -22.62 | FALSE |
| SRX2194232 | TFs and others | BRD4 | Breast | SUM 159PT | 26239 | 8511/31605 | -0.06/0.26 | -112.4 | -111.7 | -22.60 | FALSE |
| SRX11120876 | TFs and others | MAU2 | Breast | MCF-7 | 14093 | 3383/31605 | -0.24/0.26 | -112.3 | -111.6 | -22.59 | FALSE |
| SRX8080543 | TFs and others | Epitope tags | Breast | Mammary gland ductal carcinoma | 8785 | 4410/31605 | -0.18/0.26 | -112.3 | -111.5 | -22.59 | FALSE |
| SRX8639780 | TFs and others | ESR1 | Breast | MDA-MB-134-VI | 3410 | 551/31605 | -0.98/0.26 | -112.0 | -111.3 | -22.57 | FALSE |
| SRX3990998 | TFs and others | PGR | Breast | T-47D | 32760 | 6531/31605 | -0.10/0.26 | -112.0 | -111.3 | -22.57 | FALSE |
| SRX13340679 | TFs and others | Epitope tags | Breast | T-47D | 16000 | 4794/31605 | -0.16/0.26 | -112.0 | -111.3 | -22.56 | FALSE |
| SRX5493763 | TFs and others | ESR1 | Breast | Breast cancer cells | 3347 | 407/31605 | -1.18/0.26 | -112.0 | -111.2 | -22.56 | FALSE |
| SRX265423 | TFs and others | KDM5B | Breast | SUM 185PE | 51998 | 12945/31605 | 0.00/0.26 | -111.9 | -111.1 | -22.55 | FALSE |
| SRX1012629 | TFs and others | ESR1 | Breast | MCF-7 | 30593 | 2872/31605 | -0.28/0.26 | -111.8 | -111.0 | -22.54 | FALSE |
| SRX3990992 | TFs and others | PGR | Breast | T-47D | 26371 | 5778/31605 | -0.12/0.26 | -111.7 | -111.0 | -22.54 | FALSE |
| SRX185908 | TFs and others | FOXM1 | Breast | MCF-7 | 14502 | 2399/31605 | -0.33/0.26 | -111.6 | -110.8 | -22.52 | FALSE |
| SRX194569 | TFs and others | ESR1 | Breast | MCF-7 | 49246 | 5290/31605 | -0.14/0.26 | -111.5 | -110.7 | -22.51 | FALSE |
| SRX3857002 | TFs and others | CDKN1B | Breast | MDA-MB-231 | 48940 | 13092/31605 | 0.00/0.26 | -111.1 | -110.4 | -22.47 | FALSE |
| SRX5323871 | TFs and others | ESR1 | Breast | MCF-7 | 40032 | 6276/31605 | -0.11/0.26 | -111.1 | -110.4 | -22.47 | FALSE |
| SRX2912053 | TFs and others | ESR1 | Breast | MCF-7 | 6978 | 285/31605 | -1.45/0.26 | -111.0 | -110.2 | -22.46 | FALSE |
| SRX1510970 | TFs and others | ZNF143 | Breast | MCF-7 | 9938 | 5043/31605 | -0.15/0.26 | -111.0 | -110.2 | -22.46 | FALSE |
| SRX5323873 | TFs and others | ESR1 | Breast | MCF-7 | 42968 | 6795/31605 | -0.09/0.26 | -110.9 | -110.2 | -22.46 | FALSE |
| SRX19662638 | TFs and others | TAF1 | Breast | T-47D | 5370 | 5114/31605 | -0.15/0.26 | -110.9 | -110.1 | -22.45 | FALSE |
| SRX2194278 | TFs and others | BRD4 | Breast | SUM 159PT | 60081 | 11634/31605 | -0.01/0.26 | -110.8 | -110.1 | -22.44 | FALSE |
| SRX3857003 | TFs and others | CDKN1B | Breast | MDA-MB-231 | 48184 | 13021/31605 | 0.00/0.26 | -110.6 | -109.8 | -22.42 | FALSE |
| SRX3229165 | TFs and others | ESR1 | Breast | Breast cancer | 4455 | 723/31605 | -0.81/0.26 | -110.5 | -109.8 | -22.41 | FALSE |
| SRX1161186 | TFs and others | ESR1 | Breast | ZR-75-1 | 24276 | 2092/31605 | -0.37/0.26 | -110.5 | -109.8 | -22.41 | FALSE |
| SRX8617001 | TFs and others | TET2 | Breast | MCF-7 | 28094 | 4296/31605 | -0.18/0.26 | -110.5 | -109.8 | -22.41 | FALSE |
| SRX8617004 | TFs and others | TET2 | Breast | MCF-7 | 15839 | 2324/31605 | -0.34/0.26 | -110.5 | -109.7 | -22.41 | FALSE |
| SRX10365540 | TFs and others | RAC1 | Breast | MCF-7 | 7079 | 602/31605 | -0.91/0.26 | -110.4 | -109.7 | -22.40 | FALSE |
| SRX3856992 | TFs and others | JUN | Breast | MDA-MB-231 | 51468 | 13163/31605 | 0.01/0.26 | -110.4 | -109.7 | -22.40 | FALSE |
| SRX4663585 | TFs and others | ESR1 | Breast | MCF-7 | 11571 | 812/31605 | -0.75/0.26 | -110.3 | -109.6 | -22.39 | FALSE |
| SRX3857004 | TFs and others | CDKN1B | Breast | MDA-MB-231 | 49035 | 13129/31605 | 0.00/0.26 | -110.3 | -109.6 | -22.39 | FALSE |
| SRX9932059 | TFs and others | STAT5A | Breast | MCF-7 | 5992 | 4171/31605 | -0.19/0.26 | -110.2 | -109.4 | -22.38 | FALSE |
| SRX3285595 | TFs and others | KDM5B | Breast | MCF-7 | 16469 | 8928/31605 | -0.05/0.26 | -110.0 | -109.3 | -22.36 | FALSE |
| SRX3110725 | TFs and others | ESR1 | Breast | MCF-7 | 40915 | 4927/31605 | -0.15/0.26 | -110.0 | -109.3 | -22.36 | FALSE |
| SRX3285594 | TFs and others | KDM5B | Breast | MCF-7 | 19286 | 9786/31605 | -0.03/0.26 | -109.9 | -109.2 | -22.35 | FALSE |
| SRX8716244 | TFs and others | AGO1 | Breast | MCF-7 | 3253 | 233/31605 | -1.62/0.26 | -109.8 | -109.1 | -22.34 | FALSE |
| SRX2585780 | TFs and others | MYC | Breast | MDA-MB-231 | 6679 | 3898/31605 | -0.20/0.26 | -109.7 | -109.0 | -22.33 | FALSE |
| SRX371474 | TFs and others | ESR1 | Breast | BT-474 | 30656 | 3164/31605 | -0.25/0.26 | -109.7 | -109.0 | -22.33 | FALSE |
| SRX10306472 | TFs and others | ESR1 | Breast | T-47D | 3116 | 245/31605 | -1.57/0.26 | -109.6 | -108.9 | -22.32 | FALSE |
| SRX9419960 | TFs and others | MED1 | Breast | SK-BR-3 | 22307 | 7691/31605 | -0.07/0.26 | -109.6 | -108.9 | -22.32 | FALSE |
| SRX5493769 | TFs and others | ESR1 | Breast | Breast cancer cells | 6993 | 933/31605 | -0.68/0.26 | -109.6 | -108.9 | -22.32 | FALSE |
| SRX2944445 | TFs and others | MYC | Breast | MCF 10A | 11743 | 6168/31605 | -0.11/0.26 | -109.6 | -108.8 | -22.31 | FALSE |
| SRX5089558 | TFs and others | BRD4 | Breast | MCF-7 | 5821 | 3976/31605 | -0.20/0.26 | -109.5 | -108.8 | -22.31 | FALSE |
| SRX10971493 | TFs and others | ESR1 | Breast | MCF-7 | 2929 | 849/31605 | -0.73/0.26 | -109.5 | -108.8 | -22.31 | FALSE |
| SRX2007815 | TFs and others | MEN1 | Breast | MCF-7 | 6579 | 3321/31605 | -0.24/0.26 | -109.3 | -108.6 | -22.29 | FALSE |
| SRX190256 | TFs and others | EP300 | Breast | MCF-7 | 15388 | 2138/31605 | -0.36/0.26 | -109.1 | -108.4 | -22.26 | FALSE |
| SRX5128106 | TFs and others | ESR1 | Breast | ZR-75-1 | 4352 | 505/31605 | -1.01/0.26 | -108.8 | -108.1 | -22.24 | FALSE |
| SRX100265 | TFs and others | ESR1 | Breast | H3396 | 1447 | 437/31605 | -1.11/0.26 | -108.7 | -108.0 | -22.22 | FALSE |
| SRX4451195 | TFs and others | ESR1 | Breast | MCF-7 | 5493 | 2003/31605 | -0.38/0.26 | -108.7 | -108.0 | -22.22 | FALSE |
| SRX4786554 | TFs and others | KMT2C | Breast | MCF-7 | 17284 | 5424/31605 | -0.13/0.26 | -108.7 | -108.0 | -22.22 | FALSE |
| SRX265412 | TFs and others | KDM5B | Breast | MCF-7 | 42656 | 11821/31605 | -0.01/0.26 | -108.7 | -107.9 | -22.22 | FALSE |
| SRX3229140 | TFs and others | ESR1 | Breast | Breast cancer | 8743 | 1349/31605 | -0.52/0.26 | -108.6 | -107.9 | -22.22 | FALSE |
| SRX2460066 | TFs and others | ESR1 | Breast | Breast cancer cells | 3129 | 674/31605 | -0.84/0.26 | -108.5 | -107.8 | -22.21 | FALSE |
| SRX9556505 | TFs and others | GATA3 | Breast | MDA-MB-231 | 36351 | 8501/31605 | -0.05/0.26 | -108.5 | -107.8 | -22.21 | FALSE |
| SRX3541107 | TFs and others | ESR1 | Breast | MCF-7 | 2849 | 263/31605 | -1.50/0.26 | -108.3 | -107.5 | -22.18 | FALSE |
| SRX19888696 | TFs and others | NELFE | Breast | BT-549 | 7207 | 5878/31605 | -0.12/0.26 | -108.3 | -107.5 | -22.18 | FALSE |
| SRX1161185 | TFs and others | ESR1 | Breast | ZR-75-1 | 31809 | 2549/31605 | -0.31/0.26 | -108.2 | -107.5 | -22.18 | FALSE |
| SRX8617000 | TFs and others | TET2 | Breast | MCF-7 | 17222 | 2529/31605 | -0.31/0.26 | -108.2 | -107.5 | -22.17 | FALSE |
| SRX371467 | TFs and others | ESR1 | Breast | MCF-7 | 48667 | 5338/31605 | -0.13/0.26 | -108.2 | -107.4 | -22.17 | FALSE |
| SRX2460054 | TFs and others | ESR1 | Breast | Breast cancer cells | 2590 | 570/31605 | -0.93/0.26 | -108.0 | -107.3 | -22.15 | FALSE |
| SRX9419959 | TFs and others | MED1 | Breast | SK-BR-3 | 20954 | 7492/31605 | -0.07/0.26 | -108.0 | -107.2 | -22.15 | FALSE |
| SRX5323890 | TFs and others | STAT3 | Breast | T-47D | 40282 | 10381/31605 | -0.02/0.26 | -107.9 | -107.2 | -22.14 | FALSE |
| SRX5323858 | TFs and others | STAT3 | Breast | MCF-7 | 28921 | 6543/31605 | -0.10/0.26 | -107.8 | -107.1 | -22.13 | FALSE |
| SRX8537741 | TFs and others | FOXA1 | Breast | SUM 44PE | 84368 | 7930/31605 | -0.06/0.26 | -107.8 | -107.1 | -22.13 | FALSE |
| SRX5213646 | TFs and others | BRD4 | Breast | HCC1937 | 10256 | 6981/31605 | -0.08/0.26 | -107.8 | -107.0 | -22.13 | FALSE |
| SRX7846776 | TFs and others | ESR1 | Breast | MCF-7 | 2302 | 233/31605 | -1.60/0.26 | -107.6 | -106.9 | -22.12 | FALSE |
| SRX1758524 | TFs and others | Epitope tags | Breast | MCF-7 | 4146 | 3859/31605 | -0.20/0.26 | -107.6 | -106.9 | -22.11 | FALSE |
| SRX8850935 | TFs and others | Epitope tags | Breast | MCF 10A | 26910 | 8124/31605 | -0.06/0.26 | -107.6 | -106.9 | -22.11 | FALSE |
| SRX2636443 | TFs and others | ZHX2 | Breast | MCF-7 | 8263 | 3951/31605 | -0.20/0.26 | -107.6 | -106.9 | -22.11 | FALSE |
| SRX10775054 | TFs and others | ESR1 | Breast | T-47D | 2944 | 451/31605 | -1.08/0.26 | -107.5 | -106.8 | -22.10 | FALSE |
| SRX2424235 | TFs and others | CTBP1 | Breast | MCF-7 | 15371 | 4869/31605 | -0.15/0.26 | -107.5 | -106.8 | -22.10 | FALSE |
| SRX5089512 | TFs and others | HDAC1 | Breast | MCF-7 | 5048 | 4439/31605 | -0.17/0.26 | -107.3 | -106.6 | -22.08 | FALSE |
| SRX10365533 | TFs and others | ESR1 | Breast | MCF-7 | 28619 | 2888/31605 | -0.27/0.26 | -107.3 | -106.6 | -22.08 | FALSE |
| SRX5323872 | TFs and others | ESR1 | Breast | MCF-7 | 36996 | 5718/31605 | -0.12/0.26 | -107.3 | -106.6 | -22.08 | FALSE |
| SRX1012649 | TFs and others | EP300 | Breast | MCF-7 | 18475 | 2673/31605 | -0.29/0.26 | -107.3 | -106.5 | -22.07 | FALSE |
| SRX190279 | TFs and others | ESR1 | Breast | T-47D | 7183 | 575/31605 | -0.92/0.26 | -107.0 | -106.3 | -22.05 | FALSE |
| SRX673709 | TFs and others | ESR1 | Breast | MCF-7 | 2896 | 241/31605 | -1.56/0.26 | -106.9 | -106.2 | -22.04 | FALSE |
| SRX10164591 | TFs and others | ESR1 | Breast | MCF-7 | 12755 | 978/31605 | -0.65/0.26 | -106.9 | -106.2 | -22.04 | FALSE |
| SRX5089447 | TFs and others | ESR1 | Breast | MCF-7 | 25768 | 3151/31605 | -0.25/0.26 | -106.8 | -106.1 | -22.03 | FALSE |
| SRX20830447 | TFs and others | ESR1 | Breast | T-47D | 4682 | 524/31605 | -0.98/0.26 | -106.6 | -105.9 | -22.00 | FALSE |
| SRX190239 | TFs and others | GATA3 | Breast | MCF-7 | 66005 | 8321/31605 | -0.05/0.26 | -106.5 | -105.8 | -21.99 | FALSE |
| SRX15430987 | TFs and others | FOXA1 | Breast | MDA-MB-453 | 88994 | 9464/31605 | -0.03/0.26 | -106.4 | -105.7 | -21.99 | FALSE |
| SRX8080557 | TFs and others | Epitope tags | Breast | Mammary gland adenocarcinoma | 10273 | 1806/31605 | -0.41/0.26 | -106.4 | -105.7 | -21.98 | FALSE |
| SRX2194234 | TFs and others | BRD4 | Breast | SUM 159PT | 19311 | 7496/31605 | -0.07/0.26 | -106.4 | -105.7 | -21.98 | FALSE |
| SRX4786548 | TFs and others | ESR1 | Breast | MCF-7 | 4321 | 2611/31605 | -0.30/0.26 | -106.3 | -105.6 | -21.98 | FALSE |
| SRX3229138 | TFs and others | ESR1 | Breast | Breast cancer | 3655 | 2472/31605 | -0.31/0.26 | -106.2 | -105.5 | -21.96 | FALSE |
| SRX3322304 | TFs and others | CREB1 | Breast | MCF-7 | 30780 | 7582/31605 | -0.07/0.26 | -106.2 | -105.5 | -21.96 | FALSE |
| SRX1161208 | TFs and others | FOXA1 | Breast | T-47D | 8236 | 6735/31605 | -0.09/0.26 | -106.0 | -105.3 | -21.95 | FALSE |
| SRX9932044 | TFs and others | STAT5A | Breast | MCF-7 | 4398 | 3077/31605 | -0.25/0.26 | -105.7 | -105.0 | -21.91 | FALSE |
| SRX4663587 | TFs and others | ESR1 | Breast | MCF-7 | 10528 | 722/31605 | -0.79/0.26 | -105.7 | -105.0 | -21.91 | FALSE |
| SRX5323929 | TFs and others | ESR1 | Breast | Breast cancer cells | 3627 | 586/31605 | -0.90/0.26 | -105.7 | -105.0 | -21.91 | FALSE |
| SRX026902 | TFs and others | ESR1 | Breast | MCF-7 | 1486 | 209/31605 | -1.68/0.26 | -105.5 | -104.8 | -21.89 | FALSE |
| SRX4374939 | TFs and others | RBPJ | Breast | HCC1599 | 27388 | 11501/31605 | -0.01/0.26 | -105.4 | -104.7 | -21.88 | FALSE |
| SRX3285603 | TFs and others | ESR1 | Breast | FULR | 19415 | 4107/31605 | -0.18/0.26 | -105.4 | -104.7 | -21.88 | FALSE |
| SRX2460046 | TFs and others | ESR1 | Breast | Breast cancer cells | 2997 | 621/31605 | -0.87/0.26 | -105.1 | -104.3 | -21.84 | FALSE |
| SRX20830442 | TFs and others | ESR1 | Breast | T-47D | 4087 | 517/31605 | -0.98/0.26 | -105.0 | -104.3 | -21.84 | FALSE |
| SRX10637291 | TFs and others | KDM1A | Breast | MDA-MB-231 | 17260 | 6356/31605 | -0.10/0.26 | -104.9 | -104.2 | -21.83 | FALSE |
| SRX5323860 | TFs and others | STAT3 | Breast | MCF-7 | 38330 | 7945/31605 | -0.06/0.26 | -104.8 | -104.1 | -21.82 | FALSE |
| SRX3285610 | TFs and others | KDM5B | Breast | MCF-7 | 18615 | 8879/31605 | -0.04/0.26 | -104.7 | -104.0 | -21.81 | FALSE |
| SRX2423865 | TFs and others | ELK1 | Breast | MCF-7 | 5276 | 3939/31605 | -0.19/0.26 | -104.6 | -103.9 | -21.79 | FALSE |
| SRX1280448 | TFs and others | NRF1 | Breast | HMEC | 15233 | 9267/31605 | -0.03/0.26 | -104.5 | -103.8 | -21.79 | FALSE |
| SRX1161183 | TFs and others | ESR1 | Breast | ZR-75-1 | 16223 | 1617/31605 | -0.44/0.26 | -104.4 | -103.7 | -21.77 | FALSE |
| SRX188954 | TFs and others | MYC | Breast | MCF-7 | 91510 | 12781/31605 | 0.01/0.26 | -104.2 | -103.5 | -21.76 | FALSE |
| SRX3554403 | TFs and others | ESR1 | Breast | MDA-MB-134-VI | 27249 | 2963/31605 | -0.26/0.26 | -104.1 | -103.4 | -21.75 | FALSE |
| SRX3285601 | TFs and others | ESR1 | Breast | FULR | 9107 | 1219/31605 | -0.54/0.26 | -104.1 | -103.4 | -21.74 | FALSE |
| SRX21286688 | TFs and others | TAZ | Breast | MCF 10A | 20033 | 6646/31605 | -0.09/0.26 | -104.1 | -103.4 | -21.74 | FALSE |
| SRX5323832 | TFs and others | STAT3 | Breast | T-47D | 19334 | 6662/31605 | -0.09/0.26 | -104.0 | -103.3 | -21.73 | FALSE |
| SRX4451197 | TFs and others | ESR1 | Breast | MCF-7 | 7742 | 3393/31605 | -0.22/0.26 | -103.9 | -103.2 | -21.72 | FALSE |
| SRX9556499 | TFs and others | GATA3 | Breast | MDA-MB-231 | 32798 | 7772/31605 | -0.06/0.26 | -103.9 | -103.2 | -21.72 | FALSE |
| SRX19662637 | TFs and others | TAF1 | Breast | T-47D | 5291 | 4871/31605 | -0.14/0.26 | -103.8 | -103.1 | -21.71 | FALSE |
| SRX10164576 | TFs and others | ESR1 | Breast | MCF-7 | 3541 | 250/31605 | -1.50/0.26 | -103.8 | -103.1 | -21.71 | FALSE |
| SRX14875684 | TFs and others | ESR1 | Breast | MCF-7 | 9358 | 1057/31605 | -0.60/0.26 | -103.7 | -103.0 | -21.70 | FALSE |
| SRX15235714 | TFs and others | PRRX1 | Breast | Hs 578T | 27341 | 8684/31605 | -0.04/0.26 | -103.4 | -102.7 | -21.67 | FALSE |
| SRX9719177 | TFs and others | TET2 | Breast | MCF-7 | 5941 | 583/31605 | -0.89/0.26 | -103.4 | -102.7 | -21.67 | FALSE |
| SRX10365532 | TFs and others | ESR1 | Breast | MCF-7 | 31571 | 2397/31605 | -0.31/0.26 | -103.2 | -102.5 | -21.65 | FALSE |
| SRX2460058 | TFs and others | ESR1 | Breast | Breast cancer cells | 2899 | 694/31605 | -0.80/0.26 | -103.1 | -102.4 | -21.64 | FALSE |
| SRX3322322 | TFs and others | E2F4 | Breast | MCF-7 | 847 | 476/31605 | -1.01/0.26 | -103.1 | -102.4 | -21.64 | FALSE |
| SRX5089495 | TFs and others | HDAC1 | Breast | MCF-7 | 4464 | 3810/31605 | -0.19/0.26 | -103.1 | -102.4 | -21.63 | FALSE |
| SRX371470 | TFs and others | ESR1 | Breast | ZR-75-1 | 56457 | 5272/31605 | -0.13/0.26 | -102.9 | -102.2 | -21.62 | FALSE |
| SRX9932050 | TFs and others | STAT5A | Breast | MCF-7 | 4548 | 3214/31605 | -0.23/0.26 | -102.9 | -102.2 | -21.62 | FALSE |
| SRX2145568 | TFs and others | RUNX2 | Breast | MCF-7 | 27049 | 7835/31605 | -0.06/0.26 | -102.9 | -102.2 | -21.62 | FALSE |
| SRX3856995 | TFs and others | JUN | Breast | MDA-MB-231 | 63467 | 13447/31605 | 0.02/0.26 | -102.8 | -102.1 | -21.61 | FALSE |
| SRX5089499 | TFs and others | HDAC1 | Breast | MCF-7 | 4541 | 3892/31605 | -0.19/0.26 | -102.7 | -102.0 | -21.59 | FALSE |
| SRX190293 | TFs and others | NR2F2 | Breast | MCF-7 | 45188 | 7433/31605 | -0.07/0.26 | -102.6 | -101.9 | -21.58 | FALSE |
| SRX3229154 | TFs and others | ESR1 | Breast | Breast cancer | 13150 | 4347/31605 | -0.16/0.26 | -102.6 | -101.9 | -21.58 | FALSE |
| SRX063917 | TFs and others | ERG | Breast | MCF-7 | 23606 | 11770/31605 | 0.00/0.26 | -102.5 | -101.8 | -21.57 | FALSE |
| SRX2020847 | TFs and others | STAT3 | Breast | MDA-MB-468 | 33530 | 7247/31605 | -0.07/0.26 | -102.4 | -101.7 | -21.56 | FALSE |
| SRX7366777 | TFs and others | ZMYND8 | Breast | MCF-7 | 9927 | 5119/31605 | -0.13/0.26 | -102.3 | -101.6 | -21.56 | FALSE |
| SRX2881000 | TFs and others | ESR1 | Breast | MCF-7 | 35676 | 5004/31605 | -0.14/0.26 | -102.3 | -101.6 | -21.55 | FALSE |
| SRX190300 | TFs and others | EGR1 | Breast | MCF-7 | 11363 | 5925/31605 | -0.10/0.26 | -102.3 | -101.6 | -21.55 | FALSE |
| SRX1280449 | TFs and others | NRF1 | Breast | HMEC | 17187 | 9913/31605 | -0.02/0.26 | -102.2 | -101.5 | -21.54 | FALSE |
| SRX11120888 | TFs and others | EP300 | Breast | MCF-7 | 6208 | 1296/31605 | -0.51/0.26 | -102.0 | -101.3 | -21.52 | FALSE |
| SRX371469 | TFs and others | ESR1 | Breast | MCF-7 | 50796 | 4580/31605 | -0.15/0.26 | -101.9 | -101.2 | -21.51 | FALSE |
| SRX3285580 | TFs and others | KDM5B | Breast | MCF-7 | 14436 | 8203/31605 | -0.05/0.26 | -101.9 | -101.2 | -21.51 | FALSE |
| SRX8616968 | TFs and others | TET2 | Breast | MCF-7 | 1855 | 197/31605 | -1.71/0.26 | -101.8 | -101.1 | -21.49 | FALSE |
| SRX128102 | TFs and others | TFAP2C | Breast | SK-BR-3 | 64898 | 12046/31605 | 0.00/0.26 | -101.8 | -101.1 | -21.49 | FALSE |
| SRX5405696 | TFs and others | TFAP2C | Breast | SK-BR-3 | 64813 | 12040/31605 | 0.00/0.26 | -101.7 | -101.0 | -21.49 | FALSE |
| SRX3856994 | TFs and others | JUN | Breast | MDA-MB-231 | 63023 | 13459/31605 | 0.02/0.26 | -101.6 | -100.9 | -21.48 | FALSE |
| SRX6712564 | TFs and others | BRD4 | Breast | MCF-7 | 37971 | 9499/31605 | -0.03/0.26 | -101.6 | -100.9 | -21.48 | FALSE |
| SRX8616980 | TFs and others | TET2 | Breast | ZR-75-1 | 2461 | 202/31605 | -1.68/0.26 | -101.6 | -100.9 | -21.47 | FALSE |
| SRX1758480 | TFs and others | NELFE | Breast | MCF-7 | 4698 | 4286/31605 | -0.16/0.26 | -101.3 | -100.6 | -21.45 | FALSE |
| SRX5089450 | TFs and others | ESR1 | Breast | MCF-7 | 29878 | 3861/31605 | -0.19/0.26 | -101.1 | -100.5 | -21.43 | FALSE |
| SRX10365558 | TFs and others | ESR1 | Breast | MCF-7 | 37631 | 3416/31605 | -0.21/0.26 | -100.9 | -100.3 | -21.41 | FALSE |
| SRX1012644 | TFs and others | EP300 | Breast | MCF-7 | 23388 | 2863/31605 | -0.26/0.26 | -100.8 | -100.1 | -21.39 | FALSE |
| SRX5323894 | TFs and others | STAT3 | Breast | T-47D | 50903 | 11239/31605 | -0.00/0.26 | -100.7 | -100.0 | -21.38 | FALSE |
| SRX20282938 | TFs and others | ESR1 | Breast | MCF-7 | 35047 | 4173/31605 | -0.17/0.26 | -100.7 | -100.0 | -21.38 | FALSE |
| SRX5323898 | TFs and others | STAT3 | Breast | T-47D | 49124 | 10920/31605 | -0.01/0.26 | -100.6 | -99.9 | -21.37 | FALSE |
| SRX2194287 | TFs and others | BRD4 | Breast | SUM 229PE | 21538 | 7371/31605 | -0.06/0.26 | -100.6 | -99.9 | -21.37 | FALSE |
| SRX7624157 | TFs and others | ESR1 | Breast | MCF-7 | 100966 | 10839/31605 | -0.01/0.26 | -100.4 | -99.8 | -21.35 | FALSE |
| SRX9063419 | TFs and others | JUN | Breast | MCF-7 | 12932 | 6110/31605 | -0.09/0.26 | -100.4 | -99.7 | -21.35 | FALSE |
| SRX9932042 | TFs and others | STAT5A | Breast | MCF-7 | 4267 | 2975/31605 | -0.25/0.26 | -100.4 | -99.7 | -21.35 | FALSE |
| SRX1721564 | TFs and others | KDM5A | Breast | T-47D | 4633 | 3311/31605 | -0.22/0.26 | -100.4 | -99.7 | -21.35 | FALSE |
| SRX2145569 | TFs and others | RUNX2 | Breast | MCF-7 | 27828 | 7793/31605 | -0.05/0.26 | -100.3 | -99.6 | -21.34 | FALSE |
| SRX5794314 | TFs and others | TCF7 | Breast | Breast organoids | 7854 | 6436/31605 | -0.09/0.26 | -100.3 | -99.6 | -21.34 | FALSE |
| SRX2423850 | TFs and others | ZBTB40 | Breast | MCF-7 | 11127 | 4361/31605 | -0.16/0.26 | -100.2 | -99.6 | -21.33 | FALSE |
| SRX2912063 | TFs and others | ESR1 | Breast | MCF-7 | 3815 | 1670/31605 | -0.41/0.26 | -100.1 | -99.4 | -21.32 | FALSE |
| SRX2424122 | TFs and others | GTF2F1 | Breast | MCF-7 | 6105 | 3697/31605 | -0.19/0.26 | -100.1 | -99.4 | -21.31 | FALSE |
| SRX1012648 | TFs and others | EP300 | Breast | MCF-7 | 25574 | 2888/31605 | -0.25/0.26 | -100.0 | -99.3 | -21.31 | FALSE |
| SRX9556497 | TFs and others | GATA3 | Breast | MDA-MB-231 | 41524 | 8856/31605 | -0.03/0.26 | -99.9 | -99.2 | -21.29 | FALSE |
| SRX19843146 | TFs and others | MBD3 | Breast | MCF-7 | 13553 | 6366/31605 | -0.09/0.26 | -99.8 | -99.1 | -21.28 | FALSE |
| SRX1224601 | TFs and others | BRD4 | Breast | MCF 10A | 6564 | 4867/31605 | -0.14/0.26 | -99.8 | -99.1 | -21.28 | FALSE |
| SRX7624156 | TFs and others | ESR1 | Breast | MCF-7 | 71436 | 7868/31605 | -0.05/0.26 | -99.7 | -99.0 | -21.27 | FALSE |
| ERX470436 | TFs and others | NRIP1 | Breast | MCF-7 | 27892 | 4222/31605 | -0.16/0.26 | -99.6 | -99.0 | -21.27 | FALSE |
| SRX100454 | TFs and others | FOXA1 | Breast | T-47D | 76914 | 9843/31605 | -0.02/0.26 | -99.6 | -99.0 | -21.27 | FALSE |
| SRX3583224 | TFs and others | ESR1 | Breast | MCF-7 | 2814 | 182/31605 | -1.76/0.26 | -99.4 | -98.7 | -21.24 | FALSE |
| SRX265420 | TFs and others | KDM5B | Breast | T-47D | 77896 | 13991/31605 | 0.03/0.26 | -99.3 | -98.6 | -21.23 | FALSE |
| SRX643281 | TFs and others | ESR1 | Breast | MCF 10A | 14979 | 5325/31605 | -0.12/0.26 | -99.2 | -98.5 | -21.22 | FALSE |
| SRX968966 | TFs and others | ESR1 | Breast | MCF-7 | 4245 | 1093/31605 | -0.57/0.26 | -99.2 | -98.5 | -21.22 | FALSE |
| SRX6712571 | TFs and others | CBX3 | Breast | MCF-7 | 24848 | 5745/31605 | -0.10/0.26 | -99.2 | -98.5 | -21.22 | FALSE |
| SRX128100 | TFs and others | TFAP2C | Breast | BT-474 | 64472 | 12956/31605 | 0.02/0.26 | -99.2 | -98.5 | -21.22 | FALSE |
| SRX2194256 | TFs and others | BRD4 | Breast | HCC1806 | 9684 | 4232/31605 | -0.16/0.26 | -98.9 | -98.2 | -21.18 | FALSE |
| SRX2513524 | TFs and others | ESR1 | Breast | MCF-7 | 2039 | 339/31605 | -1.22/0.26 | -98.8 | -98.1 | -21.18 | FALSE |
| SRX3541108 | TFs and others | ESR1 | Breast | MCF-7 | 2042 | 171/31605 | -1.82/0.26 | -98.8 | -98.1 | -21.17 | FALSE |
| SRX2460050 | TFs and others | ESR1 | Breast | Breast cancer cells | 2551 | 620/31605 | -0.83/0.26 | -98.6 | -98.0 | -21.16 | FALSE |
| SRX5323834 | TFs and others | STAT3 | Breast | T-47D | 20365 | 6642/31605 | -0.08/0.26 | -98.6 | -97.9 | -21.15 | FALSE |
| SRX4065875 | TFs and others | CTCF | Breast | MCF-7 | 51602 | 13322/31605 | 0.02/0.26 | -98.4 | -97.7 | -21.13 | FALSE |
| SRX9556502 | TFs and others | GATA3 | Breast | MDA-MB-231 | 39898 | 8026/31605 | -0.05/0.26 | -98.3 | -97.6 | -21.12 | FALSE |
| SRX9556501 | TFs and others | GATA3 | Breast | MDA-MB-231 | 36955 | 8130/31605 | -0.04/0.26 | -98.2 | -97.5 | -21.11 | FALSE |
| SRX2513518 | TFs and others | ESR1 | Breast | MCF-7 | 29915 | 3178/31605 | -0.22/0.26 | -98.1 | -97.4 | -21.10 | FALSE |
| SRX2900157 | TFs and others | KDM6A | Breast | CAL-51 | 5481 | 4310/31605 | -0.16/0.26 | -98.1 | -97.4 | -21.10 | FALSE |
| SRX2423718 | TFs and others | MAZ | Breast | MCF-7 | 6756 | 4180/31605 | -0.16/0.26 | -98.1 | -97.4 | -21.10 | FALSE |
| SRX13298925 | TFs and others | ZEB1 | Breast | MCF-7 | 27031 | 4470/31605 | -0.15/0.26 | -98.1 | -97.4 | -21.10 | FALSE |
| SRX5323902 | TFs and others | STAT3 | Breast | T-47D | 46003 | 10503/31605 | -0.01/0.26 | -98.0 | -97.3 | -21.09 | FALSE |
| SRX2578880 | TFs and others | ESR1 | Breast | MDA-MB-231 | 22854 | 9359/31605 | -0.02/0.26 | -97.9 | -97.3 | -21.08 | FALSE |
| SRX5089552 | TFs and others | BRD4 | Breast | MCF-7 | 5497 | 3477/31605 | -0.20/0.26 | -97.9 | -97.2 | -21.08 | FALSE |
| SRX2881002 | TFs and others | ESR1 | Breast | MCF-7 | 38322 | 5133/31605 | -0.12/0.26 | -97.9 | -97.2 | -21.08 | FALSE |
| SRX9932072 | TFs and others | HDAC6 | Breast | MCF-7 | 1383 | 157/31605 | -1.90/0.26 | -97.8 | -97.1 | -21.06 | FALSE |
| SRX371471 | TFs and others | ESR1 | Breast | ZR-75-1 | 67792 | 5443/31605 | -0.11/0.26 | -97.7 | -97.0 | -21.05 | FALSE |
| SRX2513519 | TFs and others | ESR1 | Breast | MCF-7 | 32604 | 3293/31605 | -0.21/0.26 | -97.3 | -96.7 | -21.01 | FALSE |
| SRX1995230 | TFs and others | ESR1 | Breast | MCF-7 | 47785 | 4395/31605 | -0.15/0.26 | -97.3 | -96.6 | -21.01 | FALSE |
| SRX8080547 | TFs and others | Epitope tags | Breast | Mammary gland ductal carcinoma | 10356 | 4103/31605 | -0.16/0.26 | -97.2 | -96.5 | -21.00 | FALSE |
| SRX1012594 | TFs and others | ESR1 | Breast | T-47D | 5150 | 495/31605 | -0.95/0.26 | -97.2 | -96.5 | -21.00 | FALSE |
| SRX23740436 | TFs and others | ZBTB7A | Breast | SUM 149PT | 33892 | 11299/31605 | 0.00/0.26 | -97.1 | -96.4 | -20.99 | FALSE |
| SRX7282936 | TFs and others | ESR1 | Breast | ZR-75-1 | 4857 | 309/31605 | -1.27/0.26 | -97.1 | -96.4 | -20.99 | FALSE |
| SRX2423836 | TFs and others | CHD1 | Breast | MCF-7 | 7445 | 3775/31605 | -0.18/0.26 | -96.9 | -96.2 | -20.97 | FALSE |
| SRX371479 | TFs and others | ESR1 | Breast | MCF-7 | 40522 | 4221/31605 | -0.16/0.26 | -96.8 | -96.2 | -20.96 | FALSE |
| SRX10721429 | TFs and others | Epitope tags | Breast | MCF-7 | 7385 | 536/31605 | -0.90/0.26 | -96.8 | -96.1 | -20.95 | FALSE |
| SRX5323856 | TFs and others | STAT3 | Breast | MCF-7 | 24194 | 5416/31605 | -0.11/0.26 | -96.7 | -96.0 | -20.94 | FALSE |
| SRX3229150 | TFs and others | ESR1 | Breast | Breast cancer | 7634 | 1577/31605 | -0.42/0.26 | -96.6 | -96.0 | -20.94 | FALSE |
| SRX2424710 | TFs and others | PKNOX1 | Breast | MCF-7 | 19210 | 5667/31605 | -0.10/0.26 | -96.6 | -95.9 | -20.94 | FALSE |
| SRX9912220 | TFs and others | FOXA1 | Breast | T-47D | 26846 | 5165/31605 | -0.12/0.26 | -96.6 | -95.9 | -20.94 | FALSE |
| SRX3285599 | TFs and others | ESR1 | Breast | FULR | 10070 | 1522/31605 | -0.43/0.26 | -96.5 | -95.9 | -20.93 | FALSE |
| SRX1161203 | TFs and others | FOXA1 | Breast | T-47D | 47018 | 6018/31605 | -0.09/0.26 | -96.5 | -95.8 | -20.93 | FALSE |
| SRX3229152 | TFs and others | ESR1 | Breast | Breast cancer | 2265 | 362/31605 | -1.15/0.26 | -96.4 | -95.8 | -20.92 | FALSE |
| SRX23740454 | TFs and others | ZBTB7A | Breast | SUM 149PT | 32622 | 10494/31605 | -0.01/0.26 | -96.3 | -95.6 | -20.90 | FALSE |
| SRX5323862 | TFs and others | STAT3 | Breast | MCF-7 | 29493 | 6818/31605 | -0.07/0.26 | -96.3 | -95.6 | -20.90 | FALSE |
| SRX9556506 | TFs and others | GATA3 | Breast | MDA-MB-231 | 38987 | 7942/31605 | -0.05/0.26 | -96.2 | -95.5 | -20.89 | FALSE |
| SRX3321962 | TFs and others | KLF9 | Breast | MCF-7 | 9990 | 6154/31605 | -0.09/0.26 | -96.2 | -95.5 | -20.89 | FALSE |
| SRX265428 | TFs and others | CTCF | Breast | SUM 159PT | 79915 | 13058/31605 | 0.02/0.26 | -96.1 | -95.5 | -20.88 | FALSE |
| SRX3554405 | TFs and others | ESR1 | Breast | MDA-MB-134-VI | 21493 | 2895/31605 | -0.24/0.26 | -96.1 | -95.5 | -20.88 | FALSE |
| SRX2194265 | TFs and others | EP300 | Breast | SUM 159PT | 52280 | 9021/31605 | -0.03/0.26 | -95.9 | -95.2 | -20.86 | FALSE |
| SRX063918 | TFs and others | ERG | Breast | MCF-7 | 23500 | 11779/31605 | 0.01/0.26 | -95.7 | -95.0 | -20.83 | FALSE |
| SRX4377557 | TFs and others | BRD4 | Breast | MDA-MB-231 | 16266 | 6541/31605 | -0.07/0.26 | -95.5 | -94.8 | -20.81 | FALSE |
| SRX9912224 | TFs and others | FOXA1 | Breast | T-47D | 40395 | 7043/31605 | -0.06/0.26 | -95.3 | -94.7 | -20.80 | FALSE |
| SRX6712566 | TFs and others | BRD4 | Breast | MCF-7 | 3922 | 1516/31605 | -0.43/0.26 | -95.3 | -94.6 | -20.79 | FALSE |
| SRX20282953 | TFs and others | ESR1 | Breast | MCF-7 | 35713 | 4269/31605 | -0.15/0.26 | -95.2 | -94.5 | -20.78 | FALSE |
| SRX4377534 | TFs and others | BRD4 | Breast | MDA-MB-231 | 16271 | 6540/31605 | -0.07/0.26 | -95.2 | -94.5 | -20.78 | FALSE |
| SRX19210556 | TFs and others | EZH2 | Breast | SUM 149PT | 10625 | 7734/31605 | -0.05/0.26 | -94.7 | -94.0 | -20.72 | FALSE |
| SRX9932068 | TFs and others | HDAC6 | Breast | MCF-7 | 1530 | 146/31605 | -1.94/0.26 | -94.6 | -94.0 | -20.72 | FALSE |
| SRX185917 | TFs and others | FOXM1 | Breast | MCF-7 | 3167 | 842/31605 | -0.66/0.26 | -94.6 | -93.9 | -20.71 | FALSE |
| SRX15430999 | TFs and others | FOXA1 | Breast | T-47D | 62959 | 7534/31605 | -0.05/0.26 | -94.6 | -93.9 | -20.71 | FALSE |
| SRX3285604 | TFs and others | KDM5B | Breast | FULR | 26262 | 9669/31605 | -0.01/0.26 | -94.4 | -93.7 | -20.69 | FALSE |
| SRX10721428 | TFs and others | Epitope tags | Breast | MCF-7 | 6439 | 461/31605 | -0.98/0.26 | -94.1 | -93.5 | -20.66 | FALSE |
| SRX8080548 | TFs and others | Epitope tags | Breast | Mammary gland ductal carcinoma | 6747 | 1539/31605 | -0.42/0.26 | -94.1 | -93.5 | -20.66 | FALSE |
| SRX10859412 | TFs and others | SMARCA4 | Breast | BT-549 | 42529 | 11208/31605 | 0.01/0.26 | -94.1 | -93.4 | -20.66 | FALSE |
| SRX1012643 | TFs and others | EP300 | Breast | MCF-7 | 21217 | 2173/31605 | -0.31/0.26 | -94.1 | -93.4 | -20.66 | FALSE |
| SRX4451198 | TFs and others | ESR1 | Breast | MCF-7 | 6749 | 2888/31605 | -0.24/0.26 | -94.0 | -93.3 | -20.64 | FALSE |
| SRX6712578 | TFs and others | FOXA1 | Breast | MCF-7 | 35894 | 4441/31605 | -0.14/0.26 | -93.9 | -93.3 | -20.64 | FALSE |
| SRX8617024 | TFs and others | TET2 | Breast | ZR-75-1 | 2637 | 211/31605 | -1.56/0.26 | -93.9 | -93.2 | -20.64 | FALSE |
| SRX10365535 | TFs and others | ESR1 | Breast | MCF-7 | 38786 | 3506/31605 | -0.19/0.26 | -93.7 | -93.0 | -20.61 | FALSE |
| SRX5089511 | TFs and others | HDAC1 | Breast | MCF-7 | 5312 | 4549/31605 | -0.14/0.26 | -93.6 | -93.0 | -20.61 | FALSE |
| SRX13327981 | TFs and others | XRCC6 | Breast | MCF-7 | 9131 | 5338/31605 | -0.11/0.26 | -93.5 | -92.9 | -20.59 | FALSE |
| ERX470433 | TFs and others | NRIP1 | Breast | MCF-7 | 18074 | 3733/31605 | -0.18/0.26 | -93.3 | -92.7 | -20.58 | FALSE |
| SRX9556498 | TFs and others | GATA3 | Breast | MDA-MB-231 | 39609 | 7930/31605 | -0.04/0.26 | -93.3 | -92.7 | -20.58 | FALSE |
| SRX9932064 | TFs and others | HDAC6 | Breast | MCF-7 | 1604 | 147/31605 | -1.92/0.26 | -93.3 | -92.7 | -20.57 | FALSE |
| SRX1012653 | TFs and others | PGR | Breast | MCF-7 | 19027 | 2049/31605 | -0.33/0.26 | -93.3 | -92.6 | -20.57 | FALSE |
| SRX2194233 | TFs and others | BRD4 | Breast | SUM 159PT | 13964 | 6166/31605 | -0.08/0.26 | -93.1 | -92.4 | -20.55 | FALSE |
| SRX4451194 | TFs and others | ESR1 | Breast | MCF-7 | 4335 | 1645/31605 | -0.39/0.26 | -93.1 | -92.4 | -20.55 | FALSE |
| SRX22236306 | TFs and others | GATA4 | Breast | MCF-7 | 1077 | 278/31605 | -1.32/0.26 | -93.1 | -92.4 | -20.54 | FALSE |
| SRX6712567 | TFs and others | BRD4 | Breast | MCF-7 | 4076 | 1645/31605 | -0.39/0.26 | -92.9 | -92.3 | -20.53 | FALSE |
| SRX4065876 | TFs and others | CTCF | Breast | MCF-7 | 58517 | 13420/31605 | 0.03/0.26 | -92.9 | -92.3 | -20.53 | FALSE |
| SRX8873457 | TFs and others | BANP | Breast | HCC1954 | 11187 | 4589/31605 | -0.13/0.26 | -92.8 | -92.2 | -20.52 | FALSE |
| SRX9932076 | TFs and others | HDAC6 | Breast | MCF-7 | 1249 | 150/31605 | -1.89/0.26 | -92.8 | -92.1 | -20.51 | FALSE |
| SRX2513520 | TFs and others | ESR1 | Breast | MCF-7 | 35219 | 3531/31605 | -0.19/0.26 | -92.8 | -92.1 | -20.51 | FALSE |
| SRX2944444 | TFs and others | MYC | Breast | MCF 10A | 8369 | 4382/31605 | -0.14/0.26 | -92.7 | -92.0 | -20.50 | FALSE |
| SRX4374902 | TFs and others | RBPJ | Breast | MB 157 | 11882 | 5974/31605 | -0.08/0.26 | -92.6 | -91.9 | -20.49 | FALSE |
| SRX3323090 | TFs and others | CREB1 | Breast | MCF-7 | 27151 | 6468/31605 | -0.07/0.26 | -92.5 | -91.9 | -20.48 | FALSE |
| SRX371476 | TFs and others | ESR1 | Breast | MCF-7 | 41138 | 3342/31605 | -0.20/0.26 | -92.5 | -91.8 | -20.48 | FALSE |
| SRX330067 | TFs and others | ESR1 | Breast | MCF-7 | 41413 | 3953/31605 | -0.16/0.26 | -92.5 | -91.8 | -20.48 | FALSE |
| SRX11150130 | TFs and others | GATA3 | Breast | Breast cancer cells | 104744 | 9119/31605 | -0.02/0.26 | -92.4 | -91.8 | -20.47 | FALSE |
| SRX9932054 | TFs and others | STAT5A | Breast | MCF-7 | 3806 | 2917/31605 | -0.23/0.26 | -92.3 | -91.7 | -20.46 | FALSE |
| SRX20282969 | TFs and others | AHR | Breast | MCF-7 | 24338 | 3025/31605 | -0.22/0.26 | -92.2 | -91.6 | -20.45 | FALSE |
| SRX8080546 | TFs and others | Epitope tags | Breast | Mammary gland ductal carcinoma | 12051 | 1526/31605 | -0.41/0.26 | -92.0 | -91.4 | -20.43 | FALSE |
| SRX10365539 | TFs and others | RAC1 | Breast | MCF-7 | 6926 | 435/31605 | -1.00/0.26 | -92.0 | -91.3 | -20.42 | FALSE |
| SRX8111167 | TFs and others | GFP | Breast | MCF 10A | 8809 | 5147/31605 | -0.11/0.26 | -92.0 | -91.3 | -20.42 | FALSE |
| SRX673744 | TFs and others | EP300 | Breast | MCF-7 | 5150 | 2162/31605 | -0.31/0.26 | -91.9 | -91.2 | -20.41 | FALSE |
| SRX15670546 | TFs and others | TFE3 | Breast | MDA-MB-453 | 9584 | 5584/31605 | -0.09/0.26 | -91.7 | -91.0 | -20.39 | FALSE |
| SRX2912057 | TFs and others | ESR1 | Breast | MCF-7 | 8000 | 333/31605 | -1.17/0.26 | -91.4 | -90.7 | -20.35 | FALSE |
| SRX1961157 | TFs and others | FOXA1 | Breast | T-47D | 66203 | 7325/31605 | -0.05/0.26 | -91.2 | -90.6 | -20.34 | FALSE |
| SRX5089379 | TFs and others | ARID1A | Breast | MCF-7 | 34415 | 5353/31605 | -0.10/0.26 | -91.1 | -90.5 | -20.33 | FALSE |
| SRX3990988 | TFs and others | PGR | Breast | T-47D | 24696 | 4746/31605 | -0.12/0.26 | -91.1 | -90.5 | -20.32 | FALSE |
| SRX3285563 | TFs and others | KDM5B | Breast | C49R | 20250 | 9416/31605 | -0.01/0.26 | -91.1 | -90.5 | -20.32 | FALSE |
| SRX028633 | TFs and others | TFAP2C | Breast | MCF-7 | 25940 | 6664/31605 | -0.06/0.26 | -91.0 | -90.4 | -20.31 | FALSE |
| SRX1161176 | TFs and others | FOXA1 | Breast | MCF-7 | 46788 | 5541/31605 | -0.09/0.26 | -90.9 | -90.3 | -20.30 | FALSE |
| SRX673730 | TFs and others | MED1 | Breast | MCF-7 | 4193 | 650/31605 | -0.76/0.26 | -90.7 | -90.1 | -20.28 | FALSE |
| SRX1758506 | TFs and others | NELFE | Breast | MCF-7 | 3919 | 3583/31605 | -0.18/0.26 | -90.7 | -90.1 | -20.28 | FALSE |
| SRX3285614 | TFs and others | KDM5B | Breast | C70R | 18719 | 9841/31605 | -0.01/0.26 | -90.7 | -90.0 | -20.27 | FALSE |
| SRX188943 | TFs and others | MYC | Breast | MCF-7 | 98767 | 10497/31605 | 0.00/0.26 | -90.1 | -89.5 | -20.21 | FALSE |
| SRX9932060 | TFs and others | STAT5A | Breast | MCF-7 | 3825 | 2981/31605 | -0.22/0.26 | -90.1 | -89.5 | -20.21 | FALSE |
| SRX3321963 | TFs and others | KLF9 | Breast | MCF-7 | 8458 | 5567/31605 | -0.09/0.26 | -90.0 | -89.4 | -20.20 | FALSE |
| SRX10475704 | TFs and others | OVOL1 | Breast | MCF-7 | 13705 | 4049/31605 | -0.15/0.26 | -89.9 | -89.3 | -20.19 | FALSE |
| SRX186824 | TFs and others | ESR1 | Breast | Breast cancer | 7492 | 1781/31605 | -0.36/0.26 | -89.9 | -89.2 | -20.18 | FALSE |
| SRX176883 | TFs and others | EP300 | Breast | MCF-7 | 27349 | 2861/31605 | -0.23/0.26 | -89.8 | -89.2 | -20.18 | FALSE |
| SRX13298924 | TFs and others | ZEB1 | Breast | MCF-7 | 23224 | 3577/31605 | -0.18/0.26 | -89.8 | -89.1 | -20.17 | FALSE |
| SRX194573 | TFs and others | GREB1 | Breast | MCF-7 | 3620 | 342/31605 | -1.14/0.26 | -89.7 | -89.0 | -20.16 | FALSE |
| SRX095408 | TFs and others | NCOA1 | Breast | LY2 | 11936 | 5321/31605 | -0.10/0.26 | -89.7 | -89.0 | -20.16 | FALSE |
| SRX730452 | TFs and others | ESR1 | Breast | MCF-7 | 2095 | 156/31605 | -1.81/0.26 | -89.5 | -88.9 | -20.14 | FALSE |
| SRX11120900 | TFs and others | CREBBP | Breast | MCF-7 | 14928 | 4316/31605 | -0.14/0.26 | -89.5 | -88.8 | -20.14 | FALSE |
| SRX1156538 | TFs and others | SMARCA4 | Breast | MDA-MB-231 | 75160 | 10600/31605 | 0.00/0.26 | -89.5 | -88.8 | -20.14 | FALSE |
| SRX20282940 | TFs and others | ESR1 | Breast | MCF-7 | 45676 | 5504/31605 | -0.09/0.26 | -89.4 | -88.8 | -20.13 | FALSE |
| SRX10579965 | TFs and others | KDM1A | Breast | MCF-7 | 15134 | 3874/31605 | -0.16/0.26 | -89.4 | -88.8 | -20.13 | FALSE |
| SRX5089504 | TFs and others | HDAC1 | Breast | MCF-7 | 3667 | 3248/31605 | -0.20/0.26 | -89.2 | -88.5 | -20.10 | FALSE |
| SRX3110711 | TFs and others | ESR1 | Breast | MCF-7 | 3258 | 993/31605 | -0.56/0.26 | -89.0 | -88.4 | -20.08 | FALSE |
| SRX5323868 | TFs and others | STAT3 | Breast | MCF-7 | 7976 | 3171/31605 | -0.20/0.26 | -88.9 | -88.2 | -20.07 | FALSE |
| SRX2194266 | TFs and others | EP300 | Breast | SUM 159PT | 62621 | 9351/31605 | -0.01/0.26 | -88.7 | -88.0 | -20.05 | FALSE |
| SRX2423399 | TFs and others | RFX1 | Breast | MCF-7 | 14095 | 5401/31605 | -0.09/0.26 | -88.6 | -88.0 | -20.04 | FALSE |
| SRX8537739 | TFs and others | FOXA1 | Breast | MDA-MB-134-VI | 36546 | 4475/31605 | -0.13/0.26 | -88.6 | -87.9 | -20.04 | FALSE |
| SRX8080573 | TFs and others | CTCF | Breast | Mammary gland ductal carcinoma | 33728 | 10876/31605 | 0.01/0.26 | -88.5 | -87.9 | -20.03 | FALSE |
| SRX10637290 | TFs and others | KDM1A | Breast | MDA-MB-231 | 40051 | 9057/31605 | -0.01/0.26 | -88.4 | -87.8 | -20.02 | FALSE |
| SRX2007813 | TFs and others | MEN1 | Breast | MCF-7 | 4410 | 2654/31605 | -0.24/0.26 | -88.4 | -87.8 | -20.01 | FALSE |
| SRX2900151 | TFs and others | KMT2C | Breast | CAL-51 | 6569 | 3890/31605 | -0.16/0.26 | -88.3 | -87.7 | -20.01 | FALSE |
| SRX3990994 | TFs and others | PGR | Breast | T-47D | 18823 | 4410/31605 | -0.13/0.26 | -88.3 | -87.7 | -20.01 | FALSE |
| SRX1161168 | TFs and others | ESR1 | Breast | MCF-7 | 7777 | 523/31605 | -0.87/0.26 | -88.3 | -87.7 | -20.01 | FALSE |
| SRX3229153 | TFs and others | ESR1 | Breast | Breast cancer | 10041 | 1488/31605 | -0.41/0.26 | -88.3 | -87.6 | -20.00 | FALSE |
| SRX10773082 | TFs and others | TCF7 | Breast | MB 157 | 5979 | 4358/31605 | -0.13/0.26 | -88.2 | -87.6 | -19.99 | FALSE |
| SRX2424597 | TFs and others | ZNF687 | Breast | MCF-7 | 7339 | 3482/31605 | -0.18/0.26 | -88.2 | -87.6 | -19.99 | FALSE |
| SRX10475380 | TFs and others | SPDEF | Breast | MCF-7 | 12177 | 3741/31605 | -0.16/0.26 | -88.2 | -87.6 | -19.99 | FALSE |
| SRX1012596 | TFs and others | ESR1 | Breast | T-47D | 3169 | 337/31605 | -1.14/0.26 | -88.1 | -87.5 | -19.99 | FALSE |
| SRX4374904 | TFs and others | RBPJ | Breast | MB 157 | 9451 | 6235/31605 | -0.07/0.26 | -88.1 | -87.5 | -19.98 | FALSE |
| SRX039137 | TFs and others | FOXA1 | Breast | MCF-7 | 6297 | 613/31605 | -0.78/0.26 | -88.1 | -87.4 | -19.98 | FALSE |
| SRX1995229 | TFs and others | ESR1 | Breast | MCF-7 | 49259 | 5043/31605 | -0.10/0.26 | -88.0 | -87.3 | -19.97 | FALSE |
| SRX5323865 | TFs and others | STAT3 | Breast | MCF-7 | 5525 | 2803/31605 | -0.23/0.26 | -87.9 | -87.2 | -19.95 | FALSE |
| SRX5323822 | TFs and others | STAT3 | Breast | T-47D | 13433 | 4601/31605 | -0.12/0.26 | -87.9 | -87.2 | -19.95 | FALSE |
| SRX4671294 | TFs and others | RXRA | Breast | NAMEC8 | 21386 | 5415/31605 | -0.09/0.26 | -87.8 | -87.2 | -19.95 | FALSE |
| SRX371478 | TFs and others | ESR1 | Breast | MCF-7 | 53795 | 5174/31605 | -0.10/0.26 | -87.7 | -87.1 | -19.94 | FALSE |
| SRX1012642 | TFs and others | EP300 | Breast | MCF-7 | 11573 | 822/31605 | -0.63/0.26 | -87.6 | -87.0 | -19.92 | FALSE |
| SRX055308 | TFs and others | KDM5B | Breast | MCF-7 | 3463 | 3079/31605 | -0.20/0.26 | -87.5 | -86.9 | -19.91 | FALSE |
| SRX10164607 | TFs and others | ESR1 | Breast | MCF-7 | 3422 | 235/31605 | -1.41/0.26 | -87.4 | -86.8 | -19.91 | FALSE |
| SRX185918 | TFs and others | FOXM1 | Breast | MCF-7 | 2767 | 939/31605 | -0.58/0.26 | -87.3 | -86.7 | -19.89 | FALSE |
| SRX3091904 | TFs and others | ZNF711 | Breast | MCF-7 | 4532 | 3792/31605 | -0.16/0.26 | -87.2 | -86.6 | -19.88 | FALSE |
| SRX11150129 | TFs and others | GATA3 | Breast | Breast cancer cells | 92411 | 8488/31605 | -0.02/0.26 | -87.1 | -86.5 | -19.87 | FALSE |
| SRX19210522 | TFs and others | RELA | Breast | SUM 149PT | 13526 | 5865/31605 | -0.08/0.26 | -86.9 | -86.3 | -19.85 | FALSE |
| SRX3229143 | TFs and others | ESR1 | Breast | Breast cancer | 3922 | 404/31605 | -1.01/0.26 | -86.9 | -86.3 | -19.84 | FALSE |
| SRX6853582 | TFs and others | ARNT | Breast | MCF-7 | 5384 | 3419/31605 | -0.18/0.26 | -86.7 | -86.1 | -19.82 | FALSE |
| SRX5287689 | TFs and others | YAP1 | Breast | MCF-7 | 10413 | 1355/31605 | -0.43/0.26 | -86.7 | -86.1 | -19.82 | FALSE |
| SRX16028852 | TFs and others | MINA | Breast | MDA-MB-231 | 3188 | 2810/31605 | -0.22/0.26 | -86.7 | -86.0 | -19.82 | FALSE |
| SRX20282960 | TFs and others | ESR1 | Breast | MCF-7 | 49112 | 4793/31605 | -0.11/0.26 | -86.6 | -86.0 | -19.81 | FALSE |
| SRX16318944 | TFs and others | MYC | Breast | MDA-MB-231 | 5753 | 3117/31605 | -0.20/0.26 | -86.6 | -85.9 | -19.80 | FALSE |
| SRX8616978 | TFs and others | TET2 | Breast | ZR-75-1 | 2756 | 218/31605 | -1.46/0.26 | -86.6 | -85.9 | -19.80 | FALSE |
| SRX20282971 | TFs and others | ESR1 | Breast | MCF-7 | 40838 | 5104/31605 | -0.10/0.26 | -86.5 | -85.9 | -19.79 | FALSE |
| SRX1012638 | TFs and others | EP300 | Breast | MCF-7 | 11064 | 809/31605 | -0.64/0.26 | -86.4 | -85.8 | -19.78 | FALSE |
| SRX10859413 | TFs and others | SMARCC1 | Breast | BT-549 | 42087 | 8507/31605 | -0.02/0.26 | -86.3 | -85.7 | -19.78 | FALSE |
| SRX8111168 | TFs and others | GFP | Breast | MCF 10A | 9393 | 5362/31605 | -0.09/0.26 | -86.2 | -85.6 | -19.77 | FALSE |
| SRX1012741 | TFs and others | ESR1 | Breast | MCF-7 | 2350 | 604/31605 | -0.77/0.26 | -86.2 | -85.6 | -19.76 | FALSE |
| SRX10365556 | TFs and others | ESR1 | Breast | MCF-7 | 4430 | 316/31605 | -1.17/0.26 | -86.2 | -85.5 | -19.76 | FALSE |
| SRX9419950 | TFs and others | FOXA1 | Breast | SK-BR-3 | 66679 | 7870/31605 | -0.03/0.26 | -85.8 | -85.2 | -19.72 | FALSE |
| SRX10773092 | TFs and others | SMC1A | Breast | MB 157 | 74967 | 13878/31605 | 0.04/0.26 | -85.8 | -85.2 | -19.72 | FALSE |
| SRX13298923 | TFs and others | ZEB1 | Breast | MCF-7 | 21835 | 2993/31605 | -0.21/0.26 | -85.6 | -85.0 | -19.69 | FALSE |
| SRX2424598 | TFs and others | ZNF687 | Breast | MCF-7 | 7371 | 3287/31605 | -0.18/0.26 | -85.5 | -84.9 | -19.68 | FALSE |
| SRX2793579 | TFs and others | RB1 | Breast | MCF-7 | 2897 | 1441/31605 | -0.41/0.26 | -85.5 | -84.9 | -19.68 | FALSE |
| SRX5089390 | TFs and others | ARID1A | Breast | MCF-7 | 24466 | 6312/31605 | -0.06/0.26 | -85.4 | -84.7 | -19.66 | FALSE |
| ERX470434 | TFs and others | NRIP1 | Breast | MCF-7 | 7472 | 869/31605 | -0.60/0.26 | -85.2 | -84.6 | -19.65 | FALSE |
| SRX5493758 | TFs and others | FOXA1 | Breast | Breast cancer cells | 41189 | 5678/31605 | -0.08/0.26 | -85.2 | -84.6 | -19.64 | FALSE |
| SRX6777400 | TFs and others | ESR1 | Breast | MCF-7 | 2870 | 409/31605 | -0.99/0.26 | -85.1 | -84.5 | -19.63 | FALSE |
| SRX2423552 | TFs and others | NRF1 | Breast | MCF-7 | 5080 | 3173/31605 | -0.19/0.26 | -85.0 | -84.4 | -19.62 | FALSE |
| SRX3285586 | TFs and others | KDM5B | Breast | TAMR | 17459 | 8421/31605 | -0.02/0.26 | -85.0 | -84.4 | -19.62 | FALSE |
| SRX4786562 | TFs and others | KMT2C | Breast | MCF-7 | 13512 | 4066/31605 | -0.14/0.26 | -85.0 | -84.3 | -19.62 | FALSE |
| SRX185913 | TFs and others | FOXM1 | Breast | MDA-MB-231 | 3146 | 1520/31605 | -0.39/0.26 | -85.0 | -84.3 | -19.62 | FALSE |
| SRX355609 | TFs and others | ESR1 | Breast | MDA-MB-134-VI | 3282 | 200/31605 | -1.52/0.26 | -84.9 | -84.3 | -19.62 | FALSE |
| SRX2912049 | TFs and others | ESR1 | Breast | MCF-7 | 3549 | 1744/31605 | -0.35/0.26 | -84.9 | -84.3 | -19.61 | FALSE |
| SRX11120892 | TFs and others | MAU2 | Breast | MCF-7 | 29149 | 4556/31605 | -0.12/0.26 | -84.9 | -84.3 | -19.61 | FALSE |
| SRX100559 | TFs and others | GATA3 | Breast | T-47D | 64696 | 8007/31605 | -0.03/0.26 | -84.6 | -84.0 | -19.58 | FALSE |
| SRX8873458 | TFs and others | BANP | Breast | HCC1954 | 10538 | 3924/31605 | -0.15/0.26 | -84.6 | -84.0 | -19.58 | FALSE |
| SRX5457181 | TFs and others | ZNF444 | Breast | MCF-7 | 10956 | 4754/31605 | -0.11/0.26 | -84.6 | -84.0 | -19.57 | FALSE |
| SRX1709986 | TFs and others | ESR1 | Breast | T-47D | 2085 | 504/31605 | -0.86/0.26 | -84.6 | -83.9 | -19.57 | FALSE |
| SRX10773083 | TFs and others | TCF7 | Breast | MB 157 | 5204 | 3968/31605 | -0.14/0.26 | -84.5 | -83.9 | -19.57 | FALSE |
| SRX028632 | TFs and others | TFAP2C | Breast | MCF-7 | 22554 | 6198/31605 | -0.06/0.26 | -84.5 | -83.9 | -19.57 | FALSE |
| SRX8850934 | TFs and others | Epitope tags | Breast | MCF 10A | 15482 | 5542/31605 | -0.08/0.26 | -84.4 | -83.8 | -19.55 | FALSE |
| SRX10164667 | TFs and others | ESR1 | Breast | MCF-7 | 7820 | 371/31605 | -1.04/0.26 | -84.4 | -83.8 | -19.55 | FALSE |
| SRX2513517 | TFs and others | ESR1 | Breast | MCF-7 | 37372 | 3860/31605 | -0.15/0.26 | -84.3 | -83.7 | -19.54 | FALSE |
| SRX20830453 | TFs and others | Epitope tags | Breast | T-47D | 18752 | 2842/31605 | -0.21/0.26 | -84.3 | -83.7 | -19.53 | FALSE |
| SRX15430988 | TFs and others | FOXA1 | Breast | MDA-MB-453 | 70494 | 7193/31605 | -0.04/0.26 | -84.2 | -83.6 | -19.53 | FALSE |
| SRX7615734 | TFs and others | AFF4 | Breast | MCF-7 | 3578 | 2555/31605 | -0.24/0.26 | -84.0 | -83.4 | -19.50 | FALSE |
| SRX9322286 | TFs and others | ESR1 | Breast | MCF-7 | 2548 | 230/31605 | -1.39/0.26 | -84.0 | -83.4 | -19.50 | FALSE |
| SRX23740444 | TFs and others | ZBTB7A | Breast | SUM 149PT | 25926 | 9837/31605 | 0.00/0.26 | -83.9 | -83.3 | -19.50 | FALSE |
| SRX3229132 | TFs and others | ESR1 | Breast | Breast cancer | 12811 | 1690/31605 | -0.35/0.26 | -83.9 | -83.3 | -19.50 | FALSE |
| SRX7030931 | TFs and others | NELFA | Breast | MCF-7 | 4885 | 3775/31605 | -0.15/0.26 | -83.8 | -83.2 | -19.49 | FALSE |
| SRX185914 | TFs and others | FOXM1 | Breast | MDA-MB-231 | 4762 | 1953/31605 | -0.31/0.26 | -83.5 | -82.9 | -19.45 | FALSE |
| SRX14101909 | TFs and others | NELFE | Breast | MCF-7 | 7780 | 5344/31605 | -0.09/0.26 | -83.5 | -82.9 | -19.45 | FALSE |
| SRX4374905 | TFs and others | SMC1A | Breast | MB 157 | 43906 | 12732/31605 | 0.03/0.26 | -83.5 | -82.9 | -19.44 | FALSE |
| SRX9556500 | TFs and others | GATA3 | Breast | MDA-MB-231 | 31187 | 6613/31605 | -0.05/0.26 | -83.4 | -82.8 | -19.44 | FALSE |
| SRX1012655 | TFs and others | PGR | Breast | MCF-7 | 31298 | 3267/31605 | -0.18/0.26 | -83.3 | -82.7 | -19.42 | FALSE |
| SRX2194284 | TFs and others | BRD4 | Breast | SUM 159PT | 23539 | 6898/31605 | -0.04/0.26 | -83.3 | -82.7 | -19.42 | FALSE |
| SRX3541095 | TFs and others | ESR1 | Breast | MCF-7 | 1884 | 177/31605 | -1.61/0.26 | -83.3 | -82.7 | -19.42 | FALSE |
| SRX2424711 | TFs and others | PKNOX1 | Breast | MCF-7 | 13367 | 4392/31605 | -0.12/0.26 | -83.2 | -82.6 | -19.41 | FALSE |
| SRX2194235 | TFs and others | MED1 | Breast | SUM 159PT | 9750 | 4318/31605 | -0.12/0.26 | -83.2 | -82.6 | -19.41 | FALSE |
| SRX1961158 | TFs and others | FOXA1 | Breast | T-47D | 60734 | 6905/31605 | -0.04/0.26 | -83.2 | -82.6 | -19.41 | FALSE |
| SRX6750798 | TFs and others | SOX2 | Breast | MDA-MB-231 | 24748 | 7227/31605 | -0.04/0.26 | -83.0 | -82.5 | -19.39 | FALSE |
| SRX2020843 | TFs and others | STAT3 | Breast | HCC70 | 34574 | 5828/31605 | -0.07/0.26 | -83.0 | -82.4 | -19.38 | FALSE |
| SRX10475381 | TFs and others | SPDEF | Breast | MCF-7 | 9449 | 2972/31605 | -0.20/0.26 | -82.9 | -82.3 | -19.37 | FALSE |
| SRX2897211 | TFs and others | ESR1 | Breast | Breast cancer cells | 5356 | 4483/31605 | -0.11/0.26 | -82.7 | -82.1 | -19.35 | FALSE |
| SRX23740448 | TFs and others | ZBTB7A | Breast | SUM 149PT | 23856 | 7974/31605 | -0.02/0.26 | -82.6 | -82.0 | -19.34 | FALSE |
| SRX6712597 | TFs and others | NCAPG | Breast | MCF-7 | 7181 | 4687/31605 | -0.11/0.26 | -82.6 | -82.0 | -19.34 | FALSE |
| SRX9556495 | TFs and others | GATA3 | Breast | MDA-MB-231 | 28158 | 6672/31605 | -0.05/0.26 | -82.5 | -82.0 | -19.33 | FALSE |
| SRX7114561 | TFs and others | Epitope tags | Breast | MCF-7 | 71459 | 10527/31605 | 0.01/0.26 | -82.5 | -81.9 | -19.33 | FALSE |
| SRX20282972 | TFs and others | ESR1 | Breast | MCF-7 | 48156 | 4666/31605 | -0.11/0.26 | -82.4 | -81.8 | -19.32 | FALSE |
| SRX5203293 | TFs and others | ESR1 | Breast | MCF-7 | 1815 | 123/31605 | -1.97/0.26 | -82.3 | -81.7 | -19.30 | FALSE |
| SRX10773093 | TFs and others | SMC1A | Breast | MB 157 | 69775 | 13673/31605 | 0.04/0.26 | -82.3 | -81.7 | -19.30 | FALSE |
| SRX190317 | TFs and others | TCF12 | Breast | MCF-7 | 10799 | 1951/31605 | -0.30/0.26 | -82.2 | -81.6 | -19.29 | FALSE |
| SRX15235713 | TFs and others | PRRX1 | Breast | Hs 578T | 48103 | 8028/31605 | -0.02/0.26 | -82.2 | -81.6 | -19.29 | FALSE |
| SRX20282939 | TFs and others | ESR1 | Breast | MCF-7 | 50140 | 4564/31605 | -0.11/0.26 | -82.2 | -81.6 | -19.29 | FALSE |
| SRX5089550 | TFs and others | BRD4 | Breast | MCF-7 | 4372 | 2742/31605 | -0.22/0.26 | -82.2 | -81.6 | -19.29 | FALSE |
| SRX3229151 | TFs and others | ESR1 | Breast | Breast cancer | 2843 | 595/31605 | -0.76/0.26 | -82.1 | -81.5 | -19.28 | FALSE |
| SRX5251428 | TFs and others | ESR1 | Breast | T-47D | 2155 | 329/31605 | -1.11/0.26 | -82.0 | -81.5 | -19.27 | FALSE |
| SRX20282936 | TFs and others | AHR | Breast | MCF-7 | 15865 | 1678/31605 | -0.35/0.26 | -81.9 | -81.3 | -19.26 | FALSE |
| SRX20282955 | TFs and others | ESR1 | Breast | MCF-7 | 44210 | 4917/31605 | -0.10/0.26 | -81.8 | -81.2 | -19.24 | FALSE |
| SRX15431002 | TFs and others | TFE3 | Breast | T-47D | 25925 | 5106/31605 | -0.09/0.26 | -81.7 | -81.1 | -19.23 | FALSE |
| SRX10773091 | TFs and others | SMC1A | Breast | MB 157 | 61745 | 13036/31605 | 0.04/0.26 | -81.4 | -80.9 | -19.20 | FALSE |
| SRX10773090 | TFs and others | SMC1A | Breast | MB 157 | 71609 | 13908/31605 | 0.05/0.26 | -81.3 | -80.7 | -19.18 | FALSE |
| SRX2900094 | TFs and others | BAP1 | Breast | CAL-51 | 3300 | 2865/31605 | -0.20/0.26 | -80.9 | -80.3 | -19.14 | FALSE |
| SRX10859410 | TFs and others | SMARCA4 | Breast | BT-549 | 60449 | 10718/31605 | 0.02/0.26 | -80.7 | -80.2 | -19.12 | FALSE |
| SRX23740451 | TFs and others | ZBTB7A | Breast | SUM 149PT | 21117 | 8003/31605 | -0.02/0.26 | -80.6 | -80.0 | -19.10 | FALSE |
| SRX1961163 | TFs and others | FOXA1 | Breast | T-47D | 43752 | 5563/31605 | -0.07/0.26 | -80.4 | -79.8 | -19.08 | FALSE |
| SRX3322645 | TFs and others | NCOA3 | Breast | MCF-7 | 3123 | 196/31605 | -1.49/0.26 | -80.4 | -79.8 | -19.08 | FALSE |
| SRX3990984 | TFs and others | PGR | Breast | T-47D | 49802 | 6619/31605 | -0.05/0.26 | -80.4 | -79.8 | -19.08 | FALSE |
| SRX1224603 | TFs and others | BRD4 | Breast | MCF 10A | 5115 | 3734/31605 | -0.14/0.26 | -80.3 | -79.7 | -19.06 | FALSE |
| SRX3322323 | TFs and others | E2F4 | Breast | MCF-7 | 488 | 286/31605 | -1.19/0.26 | -80.1 | -79.6 | -19.05 | FALSE |
| SRX19210539 | TFs and others | EZH2 | Breast | MDA-MB-231 | 11950 | 7803/31605 | -0.02/0.26 | -80.1 | -79.5 | -19.03 | FALSE |
| SRX1473610 | TFs and others | ESRRA | Breast | BT-474 | 32657 | 4575/31605 | -0.10/0.26 | -79.8 | -79.2 | -19.01 | FALSE |
| SRX3321898 | TFs and others | ZNF579 | Breast | MCF-7 | 7377 | 2950/31605 | -0.19/0.26 | -79.8 | -79.2 | -19.00 | FALSE |
| SRX4611696 | TFs and others | ESR1 | Breast | MCF-7 | 56239 | 11516/31605 | 0.03/0.26 | -79.7 | -79.2 | -19.00 | FALSE |
| SRX11120895 | TFs and others | EP300 | Breast | MCF-7 | 7080 | 1103/31605 | -0.48/0.26 | -79.7 | -79.1 | -18.99 | FALSE |
| SRX3080212 | TFs and others | ESR1 | Breast | MCF-7 | 3392 | 233/31605 | -1.34/0.26 | -79.6 | -79.1 | -18.98 | FALSE |
| SRX1156541 | TFs and others | SMARCA4 | Breast | MDA-MB-231 | 58043 | 10396/31605 | 0.02/0.26 | -79.6 | -79.0 | -18.98 | FALSE |
| SRX10773088 | TFs and others | EBF1 | Breast | MB 157 | 83320 | 13430/31605 | 0.05/0.26 | -79.4 | -78.8 | -18.95 | FALSE |
| SRX673747 | TFs and others | MED1 | Breast | MCF-7 | 6012 | 3892/31605 | -0.13/0.26 | -79.4 | -78.8 | -18.95 | FALSE |
| SRX3990986 | TFs and others | PGR | Breast | T-47D | 49586 | 6389/31605 | -0.05/0.26 | -79.3 | -78.7 | -18.94 | FALSE |
| SRX5824550 | TFs and others | BRD7 | Breast | SUM 159PT | 36281 | 10171/31605 | 0.01/0.26 | -79.2 | -78.6 | -18.93 | FALSE |
| SRX8537738 | TFs and others | FOXA1 | Breast | MDA-MB-134-VI | 29525 | 3469/31605 | -0.16/0.26 | -79.1 | -78.5 | -18.92 | FALSE |
| SRX10773089 | TFs and others | EBF1 | Breast | MB 157 | 82756 | 13177/31605 | 0.04/0.26 | -79.0 | -78.4 | -18.90 | FALSE |
| SRX24428391 | TFs and others | Epitope tags | Breast | T-47D | 6273 | 4101/31605 | -0.12/0.26 | -79.0 | -78.4 | -18.90 | FALSE |
| SRX3229172 | TFs and others | AR | Breast | Breast cancer | 9915 | 3354/31605 | -0.16/0.26 | -78.9 | -78.4 | -18.90 | FALSE |
| SRX3229164 | TFs and others | ESR1 | Breast | Breast cancer | 2426 | 704/31605 | -0.66/0.26 | -78.7 | -78.2 | -18.87 | FALSE |
| SRX9322287 | TFs and others | ESR1 | Breast | MCF-7 | 2474 | 205/31605 | -1.43/0.26 | -78.7 | -78.1 | -18.87 | FALSE |
| SRX6712565 | TFs and others | BRD4 | Breast | MCF-7 | 19674 | 6177/31605 | -0.05/0.26 | -78.7 | -78.1 | -18.87 | FALSE |
| SRX10773096 | TFs and others | SMC1A | Breast | MB 157 | 83499 | 14261/31605 | 0.05/0.26 | -78.6 | -78.0 | -18.86 | FALSE |
| SRX3990990 | TFs and others | PGR | Breast | T-47D | 15639 | 3433/31605 | -0.16/0.26 | -78.5 | -77.9 | -18.84 | FALSE |
| SRX6712573 | TFs and others | CBX3 | Breast | MCF-7 | 4883 | 3614/31605 | -0.15/0.26 | -78.5 | -77.9 | -18.84 | FALSE |
| SRX14101924 | TFs and others | NELFE | Breast | MCF-7 | 6314 | 4548/31605 | -0.10/0.26 | -78.4 | -77.9 | -18.84 | FALSE |
| SRX9556503 | TFs and others | GATA3 | Breast | MDA-MB-231 | 37279 | 6961/31605 | -0.03/0.26 | -78.4 | -77.8 | -18.84 | FALSE |
| SRX7427512 | TFs and others | ESR1 | Breast | MCF-7 | 44150 | 5844/31605 | -0.06/0.26 | -78.4 | -77.8 | -18.83 | FALSE |
| SRX3229168 | TFs and others | AR | Breast | Breast cancer | 9225 | 2343/31605 | -0.24/0.26 | -78.2 | -77.6 | -18.81 | FALSE |
| SRX20282954 | TFs and others | ESR1 | Breast | MCF-7 | 47098 | 4705/31605 | -0.10/0.26 | -78.1 | -77.6 | -18.80 | FALSE |
| SRX18191639 | TFs and others | ESR1 | Breast | MCF-7 | 2947 | 333/31605 | -1.06/0.26 | -78.0 | -77.5 | -18.79 | FALSE |
| SRX4792908 | TFs and others | RAD21 | Breast | T-47D | 36555 | 11128/31605 | 0.03/0.26 | -78.0 | -77.4 | -18.78 | FALSE |
| SRX9719178 | TFs and others | TET2 | Breast | MCF-7 | 5149 | 465/31605 | -0.86/0.26 | -77.8 | -77.2 | -18.76 | FALSE |
| SRX5323836 | TFs and others | STAT3 | Breast | T-47D | 12345 | 4784/31605 | -0.09/0.26 | -77.8 | -77.2 | -18.76 | FALSE |
| SRX3322692 | TFs and others | NFIB | Breast | MCF-7 | 14244 | 2991/31605 | -0.18/0.26 | -77.8 | -77.2 | -18.75 | FALSE |
| SRX4023329 | TFs and others | CDK9 | Breast | T-47D | 8352 | 5165/31605 | -0.08/0.26 | -77.7 | -77.2 | -18.75 | FALSE |
| SRX19662640 | TFs and others | SIN3A | Breast | T-47D | 2243 | 1062/31605 | -0.48/0.26 | -77.7 | -77.2 | -18.75 | FALSE |
| SRX673728 | TFs and others | TFAP2C | Breast | MCF-7 | 13833 | 3924/31605 | -0.13/0.26 | -77.7 | -77.1 | -18.75 | FALSE |
| SRX7030926 | TFs and others | KDM4C | Breast | MCF-7 | 4235 | 2803/31605 | -0.20/0.26 | -77.6 | -77.0 | -18.73 | FALSE |
| SRX4284793 | TFs and others | Epitope tags | Breast | T-47D | 2162 | 169/31605 | -1.59/0.26 | -77.5 | -76.9 | -18.72 | FALSE |
| SRX666556 | TFs and others | HIF1A | Breast | T-47D | 108623 | 14750/31605 | 0.06/0.26 | -77.2 | -76.6 | -18.68 | FALSE |
| SRX3554404 | TFs and others | ESR1 | Breast | MDA-MB-134-VI | 1955 | 316/31605 | -1.09/0.26 | -77.1 | -76.6 | -18.68 | FALSE |
| SRX4377563 | TFs and others | BRD9 | Breast | MDA-MB-231 | 25392 | 7113/31605 | -0.03/0.26 | -77.1 | -76.5 | -18.67 | FALSE |
| SRX4017892 | TFs and others | TCF7 | Breast | MCF 10A | 43994 | 9084/31605 | 0.00/0.26 | -77.0 | -76.4 | -18.66 | FALSE |
| SRX10773094 | TFs and others | SMC1A | Breast | MB 157 | 80823 | 14264/31605 | 0.06/0.26 | -76.9 | -76.4 | -18.66 | FALSE |
| SRX3229187 | TFs and others | NR3C1 | Breast | Breast cancer | 4053 | 580/31605 | -0.74/0.26 | -76.9 | -76.3 | -18.65 | FALSE |
| SRX1012658 | TFs and others | PGR | Breast | MCF-7 | 12283 | 1284/31605 | -0.41/0.26 | -76.8 | -76.2 | -18.64 | FALSE |
| SRX13991433 | TFs and others | MAFB | Breast | MCF 10A | 3709 | 3336/31605 | -0.16/0.26 | -76.5 | -76.0 | -18.60 | FALSE |
| SRX9932056 | TFs and others | STAT5A | Breast | MCF-7 | 2862 | 2132/31605 | -0.26/0.26 | -76.5 | -75.9 | -18.60 | FALSE |
| SRX1012654 | TFs and others | PGR | Breast | MCF-7 | 21863 | 2132/31605 | -0.26/0.26 | -76.5 | -75.9 | -18.60 | FALSE |
| SRX20282973 | TFs and others | ESR1 | Breast | MCF-7 | 49243 | 5650/31605 | -0.06/0.26 | -76.4 | -75.8 | -18.59 | FALSE |
| SRX1012595 | TFs and others | ESR1 | Breast | T-47D | 2558 | 273/31605 | -1.19/0.26 | -76.4 | -75.8 | -18.59 | FALSE |
| SRX2578876 | TFs and others | ESR1 | Breast | MDA-MB-231 | 9796 | 5419/31605 | -0.07/0.26 | -76.4 | -75.8 | -18.58 | FALSE |
| SRX13340684 | TFs and others | GATA3 | Breast | T-47D | 28141 | 4741/31605 | -0.09/0.26 | -76.3 | -75.7 | -18.57 | FALSE |
| SRX186804 | TFs and others | ESR1 | Breast | Breast cancer | 9410 | 1441/31605 | -0.37/0.26 | -76.2 | -75.6 | -18.56 | FALSE |
| SRX20282937 | TFs and others | AHR | Breast | MCF-7 | 14476 | 1935/31605 | -0.28/0.26 | -75.9 | -75.4 | -18.53 | FALSE |
| SRX2944446 | TFs and others | MYC | Breast | MCF 10A | 5655 | 3666/31605 | -0.14/0.26 | -75.9 | -75.4 | -18.53 | FALSE |
| SRX10365534 | TFs and others | ESR1 | Breast | MCF-7 | 19793 | 1417/31605 | -0.37/0.26 | -75.7 | -75.1 | -18.50 | FALSE |
| SRX5323816 | TFs and others | STAT3 | Breast | T-47D | 9078 | 3552/31605 | -0.14/0.26 | -75.6 | -75.1 | -18.49 | FALSE |
| SRX11867611 | TFs and others | KMT2C | Breast | CAL-51 | 6879 | 3600/31605 | -0.14/0.26 | -75.6 | -75.0 | -18.49 | FALSE |
| SRX2423551 | TFs and others | NRF1 | Breast | MCF-7 | 4396 | 3037/31605 | -0.17/0.26 | -75.5 | -74.9 | -18.47 | FALSE |
| SRX8617020 | TFs and others | TET2 | Breast | MCF-7 | 1388 | 123/31605 | -1.88/0.26 | -75.5 | -74.9 | -18.47 | FALSE |
| SRX3285600 | TFs and others | ESR1 | Breast | FULR | 7679 | 879/31605 | -0.54/0.26 | -75.4 | -74.9 | -18.47 | FALSE |
| SRX795223 | TFs and others | BRD4 | Breast | SUM 159PT | 7936 | 3486/31605 | -0.14/0.26 | -75.2 | -74.7 | -18.44 | FALSE |
| SRX4931261 | TFs and others | KMT2A | Breast | MCF-7 | 3404 | 2805/31605 | -0.19/0.26 | -75.1 | -74.5 | -18.42 | FALSE |
| SRX15036448 | TFs and others | ACTR5 | Breast | MCF-7 | 3487 | 2764/31605 | -0.19/0.26 | -75.0 | -74.5 | -18.42 | FALSE |
| SRX1161177 | TFs and others | FOXA1 | Breast | MCF-7 | 51793 | 5222/31605 | -0.07/0.26 | -75.0 | -74.4 | -18.41 | FALSE |
| SRX2912067 | TFs and others | ESR1 | Breast | MCF-7 | 1946 | 723/31605 | -0.62/0.26 | -74.8 | -74.2 | -18.38 | FALSE |
| SRX5323864 | TFs and others | STAT3 | Breast | MCF-7 | 8160 | 2627/31605 | -0.20/0.26 | -74.7 | -74.1 | -18.38 | FALSE |
| SRX13867216 | TFs and others | SOX9 | Breast | MCF-7 | 23087 | 4777/31605 | -0.09/0.26 | -74.7 | -74.1 | -18.37 | FALSE |
| SRX5457295 | TFs and others | CLOCK | Breast | MCF-7 | 6615 | 2329/31605 | -0.23/0.26 | -74.5 | -73.9 | -18.35 | FALSE |
| SRX10205599 | TFs and others | YAP1 | Breast | CAL-120 | 25311 | 5943/31605 | -0.05/0.26 | -74.5 | -73.9 | -18.35 | FALSE |
| SRX5493768 | TFs and others | ESR1 | Breast | Breast cancer cells | 1585 | 172/31605 | -1.54/0.26 | -74.5 | -73.9 | -18.35 | FALSE |
| SRX10475705 | TFs and others | OVOL1 | Breast | MCF-7 | 10041 | 2834/31605 | -0.19/0.26 | -74.4 | -73.9 | -18.34 | FALSE |
| SRX13327988 | TFs and others | GATA3 | Breast | MCF-7 | 7986 | 502/31605 | -0.79/0.26 | -74.3 | -73.8 | -18.33 | FALSE |
| SRX6712568 | TFs and others | CREBBP | Breast | MCF-7 | 4606 | 2462/31605 | -0.22/0.26 | -74.3 | -73.7 | -18.32 | FALSE |
| SRX5089510 | TFs and others | HDAC1 | Breast | MCF-7 | 3091 | 2670/31605 | -0.20/0.26 | -74.2 | -73.7 | -18.32 | FALSE |
| SRX5457180 | TFs and others | ZNF444 | Breast | MCF-7 | 9525 | 3566/31605 | -0.14/0.26 | -74.1 | -73.6 | -18.31 | FALSE |
| SRX20830452 | TFs and others | Epitope tags | Breast | T-47D | 15446 | 2243/31605 | -0.24/0.26 | -74.1 | -73.6 | -18.31 | FALSE |
| SRX5089385 | TFs and others | ARID1A | Breast | MCF-7 | 28277 | 3907/31605 | -0.12/0.26 | -74.1 | -73.5 | -18.30 | FALSE |
| SRX2423849 | TFs and others | ZBTB40 | Breast | MCF-7 | 9111 | 3256/31605 | -0.16/0.26 | -74.0 | -73.5 | -18.30 | FALSE |
| SRX15391842 | TFs and others | FOXA1 | Breast | T-47D | 42710 | 5019/31605 | -0.08/0.26 | -73.9 | -73.4 | -18.28 | FALSE |
| SRX6860636 | TFs and others | ESR1 | Breast | T-47D | 2576 | 163/31605 | -1.58/0.26 | -73.8 | -73.3 | -18.27 | FALSE |
| SRX371481 | TFs and others | FOXA1 | Breast | MCF-7 | 50031 | 5356/31605 | -0.06/0.26 | -73.7 | -73.2 | -18.25 | FALSE |
| SRX3285576 | TFs and others | KDM5B | Breast | FULR | 12776 | 6648/31605 | -0.03/0.26 | -73.4 | -72.9 | -18.22 | FALSE |
| SRX6750797 | TFs and others | SOX2 | Breast | MDA-MB-231 | 14317 | 5843/31605 | -0.05/0.26 | -73.4 | -72.8 | -18.21 | FALSE |
| SRX7114558 | TFs and others | Epitope tags | Breast | MCF-7 | 56834 | 8705/31605 | 0.01/0.26 | -73.4 | -72.8 | -18.21 | FALSE |
| SRX250094 | TFs and others | TCF7L2 | Breast | MDA-MB-453 | 3935 | 2828/31605 | -0.18/0.26 | -73.1 | -72.5 | -18.17 | FALSE |
| SRX2912051 | TFs and others | ESR1 | Breast | MCF-7 | 2206 | 932/31605 | -0.51/0.26 | -73.0 | -72.4 | -18.16 | FALSE |
| SRX484476 | TFs and others | ESR1 | Breast | MDA-MB-231 | 5093 | 757/31605 | -0.59/0.26 | -73.0 | -72.4 | -18.16 | FALSE |
| SRX2793578 | TFs and others | RB1 | Breast | MCF-7 | 2728 | 933/31605 | -0.51/0.26 | -72.9 | -72.4 | -18.16 | FALSE |
| SRX176886 | TFs and others | ESR1 | Breast | ZR-75-1 | 2337 | 121/31605 | -1.86/0.26 | -72.9 | -72.3 | -18.15 | FALSE |
| SRX185709 | TFs and others | PGR | Breast | T-47D | 36666 | 5506/31605 | -0.06/0.26 | -72.7 | -72.1 | -18.13 | FALSE |
| SRX673748 | TFs and others | MED1 | Breast | MCF-7 | 3182 | 1450/31605 | -0.35/0.26 | -72.6 | -72.0 | -18.11 | FALSE |
| SRX9419951 | TFs and others | FOXA1 | Breast | SK-BR-3 | 58970 | 6965/31605 | -0.02/0.26 | -72.5 | -72.0 | -18.11 | FALSE |
| SRX11120903 | TFs and others | ESR1 | Breast | MCF-7 | 45119 | 4512/31605 | -0.09/0.26 | -72.5 | -71.9 | -18.10 | FALSE |
| SRX5089534 | TFs and others | ARID2 | Breast | MCF-7 | 11768 | 890/31605 | -0.52/0.26 | -72.4 | -71.8 | -18.09 | FALSE |
| SRX5534852 | TFs and others | EP300 | Breast | MCF-7 | 1545 | 275/31605 | -1.14/0.26 | -72.4 | -71.8 | -18.09 | FALSE |
| SRX2145566 | TFs and others | ESR1 | Breast | MCF-7 | 3808 | 131/31605 | -1.77/0.26 | -72.3 | -71.7 | -18.07 | FALSE |
| SRX4815184 | TFs and others | EN1 | Breast | MCF-7 | 22350 | 3679/31605 | -0.13/0.26 | -72.2 | -71.6 | -18.06 | FALSE |
| SRX194570 | TFs and others | GREB1 | Breast | MCF-7 | 8249 | 915/31605 | -0.51/0.26 | -72.2 | -71.6 | -18.06 | FALSE |
| SRX7540327 | TFs and others | BRD4 | Breast | MCF-7 | 3321 | 2370/31605 | -0.22/0.26 | -71.9 | -71.3 | -18.02 | FALSE |
| SRX9912222 | TFs and others | FOXA1 | Breast | T-47D | 40014 | 5957/31605 | -0.04/0.26 | -71.9 | -71.3 | -18.02 | FALSE |
| SRX1161184 | TFs and others | ESR1 | Breast | ZR-75-1 | 18025 | 1572/31605 | -0.33/0.26 | -71.8 | -71.2 | -18.01 | FALSE |
| SRX14138491 | TFs and others | RBMX | Breast | MCF-7 | 2952 | 2173/31605 | -0.24/0.26 | -71.7 | -71.1 | -18.00 | FALSE |
| SRX2007818 | TFs and others | KMT2B | Breast | MCF-7 | 3117 | 2479/31605 | -0.21/0.26 | -71.7 | -71.1 | -18.00 | FALSE |
| SRX15431000 | TFs and others | FOXA1 | Breast | T-47D | 51929 | 5985/31605 | -0.04/0.26 | -71.6 | -71.0 | -17.98 | FALSE |
| SRX5323820 | TFs and others | STAT3 | Breast | T-47D | 8471 | 3349/31605 | -0.14/0.26 | -71.6 | -71.0 | -17.98 | FALSE |
| SRX3931097 | TFs and others | GRHL2 | Breast | MCF-7 | 22018 | 4726/31605 | -0.08/0.26 | -71.5 | -71.0 | -17.98 | FALSE |
| SRX1156535 | TFs and others | SMARCA4 | Breast | MDA-MB-231 | 50423 | 9448/31605 | 0.02/0.26 | -71.5 | -70.9 | -17.97 | FALSE |
| SRX20282935 | TFs and others | AHR | Breast | MCF-7 | 16538 | 2751/31605 | -0.18/0.26 | -71.4 | -70.9 | -17.96 | FALSE |
| SRX4374901 | TFs and others | RBPJ | Breast | MB 157 | 9111 | 4143/31605 | -0.10/0.26 | -71.4 | -70.8 | -17.96 | FALSE |
| SRX883585 | TFs and others | JUN | Breast | MDA-MB-231 | 37569 | 7185/31605 | -0.02/0.26 | -71.3 | -70.7 | -17.94 | FALSE |
| SRX1012659 | TFs and others | PGR | Breast | MCF-7 | 18079 | 1780/31605 | -0.29/0.26 | -71.2 | -70.7 | -17.94 | FALSE |
| SRX8616966 | TFs and others | TET2 | Breast | MCF-7 | 1321 | 127/31605 | -1.78/0.26 | -71.2 | -70.6 | -17.93 | FALSE |
| SRX1161192 | TFs and others | FOXA1 | Breast | ZR-75-1 | 61023 | 5392/31605 | -0.06/0.26 | -71.2 | -70.6 | -17.93 | FALSE |
| SRX484477 | TFs and others | ESR1 | Breast | MDA-MB-231 | 9777 | 1491/31605 | -0.34/0.26 | -71.1 | -70.6 | -17.92 | FALSE |
| SRX2194259 | TFs and others | BRD4 | Breast | HCC1806 | 11479 | 4299/31605 | -0.09/0.26 | -71.0 | -70.4 | -17.91 | FALSE |
| SRX2578872 | TFs and others | ESR1 | Breast | MDA-MB-231 | 11412 | 3540/31605 | -0.13/0.26 | -71.0 | -70.4 | -17.91 | FALSE |
| SRX9556496 | TFs and others | GATA3 | Breast | MDA-MB-231 | 27618 | 5790/31605 | -0.05/0.26 | -71.0 | -70.4 | -17.90 | FALSE |
| SRX4786541 | TFs and others | ESR1 | Breast | MCF-7 | 1469 | 101/31605 | -2.03/0.26 | -70.9 | -70.4 | -17.90 | FALSE |
| SRX10045337 | TFs and others | ASXL1 | Breast | CAL-51 | 3767 | 2925/31605 | -0.17/0.26 | -70.9 | -70.3 | -17.89 | FALSE |
| SRX1161205 | TFs and others | FOXA1 | Breast | T-47D | 38918 | 4509/31605 | -0.09/0.26 | -70.9 | -70.3 | -17.89 | FALSE |
| SRX6853600 | TFs and others | ARNT | Breast | MCF-7 | 4673 | 2717/31605 | -0.18/0.26 | -70.8 | -70.3 | -17.89 | FALSE |
| SRX730425 | TFs and others | ESR1 | Breast | MCF-7 | 1561 | 100/31605 | -2.03/0.26 | -70.7 | -70.2 | -17.87 | FALSE |
| SRX318858 | TFs and others | ZBTB17 | Breast | MDA-MB-231 | 7317 | 4515/31605 | -0.09/0.26 | -70.6 | -70.1 | -17.86 | FALSE |
| SRX4786568 | TFs and others | TET2 | Breast | MCF-7 | 4086 | 3211/31605 | -0.15/0.26 | -70.6 | -70.1 | -17.86 | FALSE |
| SRX128099 | TFs and others | TFAP2C | Breast | MCF-7 | 42902 | 6655/31605 | -0.03/0.26 | -70.6 | -70.0 | -17.85 | FALSE |
| SRX1012597 | TFs and others | ESR1 | Breast | T-47D | 11740 | 1405/31605 | -0.35/0.26 | -70.5 | -69.9 | -17.84 | FALSE |
| SRX5534832 | TFs and others | ESR1 | Breast | MCF-7 | 42304 | 5577/31605 | -0.05/0.26 | -70.5 | -69.9 | -17.84 | FALSE |
| SRX3285611 | TFs and others | KDM5B | Breast | MCF-7 | 20813 | 8575/31605 | 0.01/0.26 | -70.4 | -69.9 | -17.83 | FALSE |
| SRX10365543 | TFs and others | RAC1 | Breast | MCF-7 | 28423 | 2131/31605 | -0.24/0.26 | -70.4 | -69.9 | -17.83 | FALSE |
| SRX150570 | TFs and others | MYC | Breast | MCF 10A | 36343 | 6028/31605 | -0.04/0.26 | -70.2 | -69.7 | -17.81 | FALSE |
| SRX13340687 | TFs and others | GATA3 | Breast | T-47D | 23042 | 3818/31605 | -0.11/0.26 | -70.2 | -69.6 | -17.80 | FALSE |
| SRX3285557 | TFs and others | ESR1 | Breast | MCF-7 | 2861 | 207/31605 | -1.33/0.26 | -70.2 | -69.6 | -17.80 | FALSE |
| SRX19210543 | TFs and others | EZH2 | Breast | MDA-MB-231 | 10941 | 7593/31605 | -0.01/0.26 | -70.0 | -69.5 | -17.78 | FALSE |
| SRX018833 | TFs and others | TFAP2C | Breast | MCF-7 | 42945 | 6674/31605 | -0.02/0.26 | -69.9 | -69.4 | -17.77 | FALSE |
| SRX3931044 | TFs and others | FOXA1 | Breast | MCF-7 | 102485 | 9423/31605 | 0.02/0.26 | -69.9 | -69.3 | -17.77 | FALSE |
| SRX4367192 | TFs and others | ESR1 | Breast | MCF-7 | 77625 | 8210/31605 | 0.00/0.26 | -69.8 | -69.3 | -17.76 | FALSE |
| SRX4367193 | TFs and others | ESR1 | Breast | MCF-7 | 112434 | 10248/31605 | 0.03/0.26 | -69.7 | -69.2 | -17.74 | FALSE |
| SRX20282961 | TFs and others | ESR1 | Breast | MCF-7 | 51408 | 4971/31605 | -0.07/0.26 | -69.7 | -69.1 | -17.74 | FALSE |
| SRX5089430 | TFs and others | SMARCB1 | Breast | MCF-7 | 16532 | 2129/31605 | -0.24/0.26 | -69.5 | -69.0 | -17.72 | FALSE |
| SRX18191637 | TFs and others | ESR1 | Breast | MCF-7 | 2420 | 223/31605 | -1.26/0.26 | -69.5 | -68.9 | -17.71 | FALSE |
| SRX5172064 | TFs and others | SMARCC1 | Breast | MCF-7 | 8939 | 3096/31605 | -0.15/0.26 | -69.4 | -68.9 | -17.70 | FALSE |
| SRX3931059 | TFs and others | GRHL2 | Breast | MCF-7 | 36260 | 6103/31605 | -0.04/0.26 | -69.4 | -68.8 | -17.70 | FALSE |
| SRX3110713 | TFs and others | ESR1 | Breast | MCF-7 | 2348 | 684/31605 | -0.61/0.26 | -69.3 | -68.8 | -17.70 | FALSE |
| SRX4611709 | TFs and others | ESR1 | Breast | MCF-7 | 56276 | 8794/31605 | 0.01/0.26 | -69.3 | -68.8 | -17.69 | FALSE |
| SRX1709990 | TFs and others | ESR1 | Breast | T-47D | 2711 | 506/31605 | -0.75/0.26 | -69.3 | -68.7 | -17.69 | FALSE |
| SRX18191649 | TFs and others | ESR1 | Breast | MCF-7 | 2824 | 314/31605 | -1.02/0.26 | -69.2 | -68.6 | -17.68 | FALSE |
| SRX100567 | TFs and others | EP300 | Breast | T-47D | 17703 | 3610/31605 | -0.12/0.26 | -69.1 | -68.5 | -17.66 | FALSE |
| SRX14875686 | TFs and others | ESR1 | Breast | MCF-7 | 6864 | 792/31605 | -0.55/0.26 | -68.9 | -68.4 | -17.64 | FALSE |
| SRX10468633 | TFs and others | FOXA1 | Breast | Breast cancer cells | 61729 | 5787/31605 | -0.04/0.26 | -68.9 | -68.4 | -17.64 | FALSE |
| SRX9322283 | TFs and others | ESR1 | Breast | MCF-7 | 2675 | 236/31605 | -1.21/0.26 | -68.9 | -68.3 | -17.63 | FALSE |
| SRX19210521 | TFs and others | RELA | Breast | SUM 149PT | 12075 | 4562/31605 | -0.08/0.26 | -68.8 | -68.3 | -17.63 | FALSE |
| SRX19210517 | TFs and others | EZH2 | Breast | SUM 149PT | 9965 | 7390/31605 | -0.01/0.26 | -68.6 | -68.1 | -17.60 | FALSE |
| SRX19888704 | TFs and others | NELFE | Breast | MCF-7 | 6417 | 4470/31605 | -0.08/0.26 | -68.6 | -68.0 | -17.59 | FALSE |
| SRX1012598 | TFs and others | ESR1 | Breast | T-47D | 12138 | 1422/31605 | -0.34/0.26 | -68.6 | -68.0 | -17.59 | FALSE |
| SRX7030927 | TFs and others | KDM4C | Breast | MCF-7 | 2833 | 1962/31605 | -0.25/0.26 | -68.5 | -68.0 | -17.59 | FALSE |
| SRX2900150 | TFs and others | KMT2C | Breast | CAL-51 | 5479 | 2870/31605 | -0.16/0.26 | -68.4 | -67.8 | -17.57 | FALSE |
| SRX1426074 | TFs and others | PHF2 | Breast | HMLE | 5583 | 4436/31605 | -0.08/0.26 | -68.1 | -67.6 | -17.54 | FALSE |
| SRX1585245 | TFs and others | ESR1 | Breast | LTED cells | 1292 | 165/31605 | -1.49/0.26 | -68.0 | -67.4 | -17.52 | FALSE |
| SRX3285565 | TFs and others | KDM5B | Breast | C70R | 16575 | 7841/31605 | 0.00/0.26 | -68.0 | -67.4 | -17.52 | FALSE |
| SRX1012680 | TFs and others | ESR1 | Breast | MCF-7 | 1905 | 217/31605 | -1.27/0.26 | -68.0 | -67.4 | -17.52 | FALSE |
| SRX9556504 | TFs and others | GATA3 | Breast | MDA-MB-231 | 38773 | 6321/31605 | -0.03/0.26 | -67.8 | -67.3 | -17.50 | FALSE |
| SRX5251419 | TFs and others | ESR1 | Breast | MCF-7 | 1345 | 146/31605 | -1.60/0.26 | -67.8 | -67.2 | -17.49 | FALSE |
| SRX490436 | TFs and others | ESR1 | Breast | MCF-7 | 829 | 106/31605 | -1.91/0.26 | -67.3 | -66.8 | -17.43 | FALSE |
| SRX7846779 | TFs and others | ESR1 | Breast | MCF-7 | 2282 | 143/31605 | -1.61/0.26 | -67.3 | -66.8 | -17.43 | FALSE |
| SRX4611723 | TFs and others | ESR1 | Breast | MCF-7 | 62528 | 9395/31605 | 0.02/0.26 | -67.2 | -66.7 | -17.42 | FALSE |
| SRX176861 | TFs and others | ESR1 | Breast | MCF-7 | 72023 | 6412/31605 | -0.02/0.26 | -67.1 | -66.6 | -17.40 | FALSE |
| SRX4611740 | TFs and others | ESR1 | Breast | MCF-7 | 54060 | 9095/31605 | 0.02/0.26 | -67.1 | -66.6 | -17.40 | FALSE |
| SRX2060922 | TFs and others | TP53 | Breast | MCF-7 | 30548 | 4880/31605 | -0.06/0.26 | -67.0 | -66.5 | -17.39 | FALSE |
| SRX1012678 | TFs and others | ESR1 | Breast | MCF-7 | 2002 | 277/31605 | -1.08/0.26 | -67.0 | -66.5 | -17.39 | FALSE |
| SRX3229148 | TFs and others | ESR1 | Breast | Breast cancer | 4141 | 807/31605 | -0.53/0.26 | -67.0 | -66.5 | -17.39 | FALSE |
| SRX1758525 | TFs and others | Epitope tags | Breast | MCF-7 | 3974 | 2991/31605 | -0.15/0.26 | -66.9 | -66.4 | -17.38 | FALSE |
| SRX3931098 | TFs and others | GRHL2 | Breast | MCF-7 | 20529 | 4261/31605 | -0.09/0.26 | -66.9 | -66.3 | -17.37 | FALSE |
| SRX4786552 | TFs and others | TET2 | Breast | MCF-7 | 3339 | 2640/31605 | -0.18/0.26 | -66.9 | -66.3 | -17.37 | FALSE |
| SRX4671296 | TFs and others | RXRA | Breast | NAMEC8 | 11221 | 2763/31605 | -0.17/0.26 | -66.9 | -66.3 | -17.37 | FALSE |
| SRX7114559 | TFs and others | Epitope tags | Breast | MCF-7 | 71704 | 9216/31605 | 0.02/0.26 | -66.8 | -66.3 | -17.37 | FALSE |
| SRX5534865 | TFs and others | GATA3 | Breast | MCF-7 | 10993 | 5052/31605 | -0.06/0.26 | -66.8 | -66.2 | -17.36 | FALSE |
| SRX11858167 | TFs and others | TFAP2C | Breast | MCF-7 | 12651 | 3218/31605 | -0.14/0.26 | -66.8 | -66.2 | -17.36 | FALSE |
| SRX12008354 | TFs and others | CTCF | Breast | MCF 10A | 64640 | 14730/31605 | 0.07/0.26 | -66.6 | -66.0 | -17.33 | FALSE |
| SRX4213901 | TFs and others | MYBL2 | Breast | MCF 10A | 2497 | 1402/31605 | -0.34/0.26 | -66.5 | -66.0 | -17.33 | FALSE |
| SRX25289343 | TFs and others | GRHL2 | Breast | MCF-7 | 17193 | 3614/31605 | -0.11/0.26 | -66.5 | -66.0 | -17.32 | FALSE |
| SRX3554401 | TFs and others | ESR1 | Breast | MDA-MB-134-VI | 1030 | 115/31605 | -1.81/0.26 | -66.5 | -66.0 | -17.32 | FALSE |
| SRX3931057 | TFs and others | GRHL2 | Breast | MCF-7 | 35387 | 6054/31605 | -0.03/0.26 | -66.5 | -65.9 | -17.32 | FALSE |
| SRX4451199 | TFs and others | ESR1 | Breast | MCF-7 | 3967 | 1799/31605 | -0.27/0.26 | -66.4 | -65.9 | -17.32 | FALSE |
| SRX1660871 | TFs and others | TRIM25 | Breast | Breast cancer cells | 35832 | 11400/31605 | 0.05/0.26 | -66.4 | -65.9 | -17.31 | FALSE |
| SRX9932070 | TFs and others | HDAC6 | Breast | MCF-7 | 878 | 105/31605 | -1.91/0.26 | -66.4 | -65.8 | -17.31 | FALSE |
| SRX18191650 | TFs and others | NR2F2 | Breast | MCF-7 | 10852 | 1284/31605 | -0.36/0.26 | -66.3 | -65.8 | -17.30 | FALSE |
| SRX4792912 | TFs and others | RAD21 | Breast | T-47D | 27165 | 9663/31605 | 0.03/0.26 | -66.3 | -65.8 | -17.30 | FALSE |
| SRX3229157 | TFs and others | ESR1 | Breast | Breast cancer | 1538 | 303/31605 | -1.02/0.26 | -66.2 | -65.7 | -17.28 | FALSE |
| SRX1161187 | TFs and others | FOXA1 | Breast | ZR-75-1 | 69288 | 5908/31605 | -0.03/0.26 | -66.1 | -65.6 | -17.27 | FALSE |
| SRX176860 | TFs and others | ESR1 | Breast | MCF-7 | 77212 | 6264/31605 | -0.02/0.26 | -66.1 | -65.5 | -17.26 | FALSE |
| SRX371483 | TFs and others | FOXA1 | Breast | MCF-7 | 54807 | 5463/31605 | -0.04/0.26 | -66.0 | -65.5 | -17.26 | FALSE |
| SRX17989901 | TFs and others | SIN3B | Breast | MDA-MB-231 | 2026 | 2218/31605 | -0.21/0.26 | -66.0 | -65.5 | -17.26 | FALSE |
| SRX11149846 | TFs and others | GATA3 | Breast | MDA-MB-453 | 103187 | 7415/31605 | -0.00/0.26 | -66.0 | -65.4 | -17.25 | FALSE |
| SRX5089506 | TFs and others | HDAC1 | Breast | MCF-7 | 3032 | 2537/31605 | -0.18/0.26 | -65.9 | -65.4 | -17.25 | FALSE |
| SRX15946407 | TFs and others | GATA3 | Breast | MDA-MB-453 | 103147 | 7432/31605 | -0.00/0.26 | -65.9 | -65.4 | -17.25 | FALSE |
| SRX5089381 | TFs and others | ARID1A | Breast | MCF-7 | 18219 | 2342/31605 | -0.20/0.26 | -65.9 | -65.4 | -17.24 | FALSE |
| SRX186810 | TFs and others | ESR1 | Breast | Breast cancer | 5278 | 934/31605 | -0.47/0.26 | -65.8 | -65.3 | -17.24 | FALSE |
| SRX060810 | TFs and others | GATA3 | Breast | MCF-7 | 20636 | 2011/31605 | -0.24/0.26 | -65.8 | -65.2 | -17.23 | FALSE |
| SRX3229195 | TFs and others | GATA3 | Breast | Breast cancer | 17057 | 2515/31605 | -0.18/0.26 | -65.6 | -65.1 | -17.21 | FALSE |
| SRX396819 | TFs and others | ESR1 | Breast | MCF-7 | 1632 | 101/31605 | -1.94/0.26 | -65.5 | -65.0 | -17.19 | FALSE |
| SRX18191636 | TFs and others | ESR1 | Breast | MCF-7 | 2593 | 262/31605 | -1.10/0.26 | -65.4 | -64.9 | -17.18 | FALSE |
| SRX2871360 | TFs and others | PGR | Breast | T-47D | 51883 | 6067/31605 | -0.03/0.26 | -65.4 | -64.9 | -17.17 | FALSE |
| SRX9419949 | TFs and others | FOXA1 | Breast | SK-BR-3 | 64064 | 6758/31605 | -0.01/0.26 | -65.4 | -64.8 | -17.17 | FALSE |
| SRX2944452 | TFs and others | MYC | Breast | MCF 10A | 4867 | 2908/31605 | -0.15/0.26 | -65.3 | -64.7 | -17.16 | FALSE |
| SRX18191651 | TFs and others | NR2F2 | Breast | MCF-7 | 14772 | 2073/31605 | -0.23/0.26 | -65.2 | -64.7 | -17.16 | FALSE |
| SRX3401272 | TFs and others | TEAD1 | Breast | MCF-7 | 42266 | 5035/31605 | -0.05/0.26 | -64.8 | -64.3 | -17.10 | FALSE |
| SRX130108 | TFs and others | CTBP1 | Breast | MCF-7 | 10448 | 2625/31605 | -0.17/0.26 | -64.7 | -64.2 | -17.09 | FALSE |
| SRX2424236 | TFs and others | CTBP1 | Breast | MCF-7 | 12646 | 3240/31605 | -0.13/0.26 | -64.5 | -64.0 | -17.06 | FALSE |
| SRX3229173 | TFs and others | AR | Breast | Breast cancer | 3437 | 400/31605 | -0.84/0.26 | -64.4 | -63.9 | -17.05 | FALSE |
| SRX13427194 | TFs and others | ESR1 | Breast | MDA-MB-453 | 47446 | 5506/31605 | -0.04/0.26 | -64.4 | -63.9 | -17.04 | FALSE |
| SRX14255715 | TFs and others | ESR1 | Breast | MCF-7 | 1552 | 118/31605 | -1.75/0.26 | -64.3 | -63.8 | -17.03 | FALSE |
| SRX10365544 | TFs and others | RAC1 | Breast | MCF-7 | 5704 | 531/31605 | -0.69/0.26 | -64.3 | -63.8 | -17.03 | FALSE |
| SRX24428393 | TFs and others | Epitope tags | Breast | T-47D | 4052 | 2460/31605 | -0.18/0.26 | -64.3 | -63.7 | -17.02 | FALSE |
| SRX1161206 | TFs and others | FOXA1 | Breast | T-47D | 26202 | 3359/31605 | -0.12/0.26 | -64.2 | -63.7 | -17.02 | FALSE |
| SRX15337043 | TFs and others | Epitope tags | Breast | T-47D | 1622 | 1185/31605 | -0.38/0.26 | -64.2 | -63.6 | -17.01 | FALSE |
| SRX13867217 | TFs and others | SOX9 | Breast | MCF-7 | 20501 | 4138/31605 | -0.08/0.26 | -64.1 | -63.6 | -17.00 | FALSE |
| SRX5493764 | TFs and others | ESR1 | Breast | Breast cancer cells | 1353 | 141/31605 | -1.58/0.26 | -64.1 | -63.5 | -17.00 | FALSE |
| SRX4451209 | TFs and others | ESR1 | Breast | MCF-7 | 4218 | 879/31605 | -0.48/0.26 | -64.0 | -63.5 | -16.99 | FALSE |
| SRX2871361 | TFs and others | PGR | Breast | T-47D | 52572 | 6059/31605 | -0.02/0.26 | -63.9 | -63.4 | -16.98 | FALSE |
| SRX10045338 | TFs and others | ASXL1 | Breast | CAL-51 | 2573 | 2304/31605 | -0.20/0.26 | -63.9 | -63.3 | -16.97 | FALSE |
| SRX4611703 | TFs and others | ESR1 | Breast | MCF-7 | 64266 | 9079/31605 | 0.03/0.26 | -63.8 | -63.2 | -16.95 | FALSE |
| SRX13427190 | TFs and others | ESR1 | Breast | MDA-MB-453 | 64279 | 6446/31605 | -0.02/0.26 | -63.7 | -63.2 | -16.95 | FALSE |
| SRX20282951 | TFs and others | AHR | Breast | MCF-7 | 3828 | 364/31605 | -0.88/0.26 | -63.7 | -63.2 | -16.94 | FALSE |
| SRX2944451 | TFs and others | MYC | Breast | MCF 10A | 4933 | 3031/31605 | -0.14/0.26 | -63.6 | -63.1 | -16.94 | FALSE |
| SRX4815197 | TFs and others | FOXA1 | Breast | MCF-7 | 124879 | 9890/31605 | 0.04/0.26 | -63.6 | -63.1 | -16.94 | FALSE |
| SRX5794313 | TFs and others | TCF7 | Breast | Breast organoids | 8132 | 5152/31605 | -0.05/0.26 | -63.5 | -63.0 | -16.92 | FALSE |
| SRX3990982 | TFs and others | PGR | Breast | T-47D | 30512 | 4258/31605 | -0.08/0.26 | -63.5 | -63.0 | -16.92 | FALSE |
| SRX3554410 | TFs and others | CREB1 | Breast | MDA-MB-134-VI | 4813 | 1666/31605 | -0.28/0.26 | -63.5 | -62.9 | -16.92 | FALSE |
| SRX4611737 | TFs and others | ESR1 | Breast | MCF-7 | 60079 | 8559/31605 | 0.02/0.26 | -63.4 | -62.9 | -16.91 | FALSE |
| SRX5493761 | TFs and others | FOXA1 | Breast | Breast cancer cells | 16723 | 2675/31605 | -0.16/0.26 | -63.3 | -62.8 | -16.89 | FALSE |
| SRX2897203 | TFs and others | ESR1 | Breast | Breast cancer cells | 3360 | 2097/31605 | -0.22/0.26 | -63.2 | -62.7 | -16.88 | FALSE |
| SRX100564 | TFs and others | ESR1 | Breast | T-47D | 3476 | 1041/31605 | -0.41/0.26 | -63.0 | -62.5 | -16.85 | FALSE |
| SRX4611741 | TFs and others | ESR1 | Breast | MCF-7 | 61699 | 9569/31605 | 0.03/0.26 | -63.0 | -62.5 | -16.85 | FALSE |
| SRX9932066 | TFs and others | HDAC6 | Breast | MCF-7 | 943 | 89/31605 | -2.03/0.26 | -62.9 | -62.4 | -16.84 | FALSE |
| SRX4611711 | TFs and others | ESR1 | Breast | MCF-7 | 55292 | 9336/31605 | 0.03/0.26 | -62.9 | -62.4 | -16.84 | FALSE |
| SRX3229144 | TFs and others | ESR1 | Breast | Breast cancer | 2657 | 806/31605 | -0.50/0.26 | -62.9 | -62.4 | -16.84 | FALSE |
| SRX2424121 | TFs and others | GTF2F1 | Breast | MCF-7 | 3716 | 2294/31605 | -0.19/0.26 | -62.9 | -62.4 | -16.83 | FALSE |
| SRX10468635 | TFs and others | FOXA1 | Breast | Breast cancer cells | 61217 | 5927/31605 | -0.02/0.26 | -62.8 | -62.3 | -16.82 | FALSE |
| SRX1161191 | TFs and others | FOXA1 | Breast | ZR-75-1 | 50267 | 4523/31605 | -0.07/0.26 | -62.7 | -62.2 | -16.82 | FALSE |
| SRX1161207 | TFs and others | FOXA1 | Breast | T-47D | 32680 | 3499/31605 | -0.11/0.26 | -62.7 | -62.2 | -16.81 | FALSE |
| SRX6712581 | TFs and others | GATA3 | Breast | MCF-7 | 53159 | 5614/31605 | -0.03/0.26 | -62.6 | -62.1 | -16.80 | FALSE |
| SRX5493767 | TFs and others | ESR1 | Breast | Breast cancer cells | 1252 | 134/31605 | -1.60/0.26 | -62.5 | -62.0 | -16.79 | FALSE |
| SRX7540326 | TFs and others | BRD4 | Breast | MCF-7 | 3143 | 2368/31605 | -0.19/0.26 | -62.5 | -62.0 | -16.78 | FALSE |
| SRX4815198 | TFs and others | FOXA1 | Breast | MCF-7 | 167287 | 11682/31605 | 0.06/0.26 | -62.4 | -61.9 | -16.77 | FALSE |
| SRX4611743 | TFs and others | ESR1 | Breast | MCF-7 | 57581 | 8847/31605 | 0.03/0.26 | -62.4 | -61.9 | -16.77 | FALSE |
| SRX4374903 | TFs and others | RBPJ | Breast | MB 157 | 5612 | 3296/31605 | -0.12/0.26 | -62.4 | -61.8 | -16.76 | FALSE |
| SRX7624146 | TFs and others | NR2F2 | Breast | MCF-7 | 11349 | 1356/31605 | -0.33/0.26 | -62.3 | -61.8 | -16.75 | FALSE |
| SRX2871364 | TFs and others | PGR | Breast | T-47D | 28879 | 3821/31605 | -0.09/0.26 | -62.1 | -61.6 | -16.73 | FALSE |
| SRX4611742 | TFs and others | ESR1 | Breast | MCF-7 | 60589 | 8894/31605 | 0.03/0.26 | -62.0 | -61.5 | -16.72 | FALSE |
| SRX5089391 | TFs and others | ARID1A | Breast | MCF-7 | 24101 | 2942/31605 | -0.14/0.26 | -62.0 | -61.5 | -16.72 | FALSE |
| SRX2897216 | TFs and others | ESR1 | Breast | Breast cancer cells | 6243 | 5314/31605 | -0.04/0.26 | -62.0 | -61.5 | -16.72 | FALSE |
| SRX8083099 | TFs and others | FOXA1 | Breast | CAMA-1 | 66675 | 6045/31605 | -0.02/0.26 | -62.0 | -61.5 | -16.71 | FALSE |
| SRX8850933 | TFs and others | Epitope tags | Breast | MCF 10A | 9068 | 3191/31605 | -0.12/0.26 | -61.9 | -61.4 | -16.70 | FALSE |
| SRX371482 | TFs and others | FOXA1 | Breast | MCF-7 | 68250 | 7113/31605 | 0.00/0.26 | -61.7 | -61.2 | -16.68 | FALSE |
| SRX1531788 | TFs and others | NIPBL | Breast | MCF-7 | 2181 | 139/31605 | -1.56/0.26 | -61.7 | -61.2 | -16.68 | FALSE |
| SRX10365517 | TFs and others | RAC1 | Breast | MCF-7 | 10501 | 1089/31605 | -0.39/0.26 | -61.7 | -61.2 | -16.68 | FALSE |
| SRX4611697 | TFs and others | ESR1 | Breast | MCF-7 | 61700 | 9046/31605 | 0.03/0.26 | -61.7 | -61.2 | -16.67 | FALSE |
| SRX6712579 | TFs and others | FOXA1 | Breast | MCF-7 | 1015 | 88/31605 | -2.02/0.26 | -61.6 | -61.1 | -16.66 | FALSE |
| SRX4611688 | TFs and others | ESR1 | Breast | MCF-7 | 49892 | 8629/31605 | 0.03/0.26 | -61.6 | -61.1 | -16.66 | FALSE |
| SRX1161190 | TFs and others | FOXA1 | Breast | ZR-75-1 | 63682 | 5456/31605 | -0.03/0.26 | -61.6 | -61.1 | -16.66 | FALSE |
| SRX188952 | TFs and others | CTCF | Breast | MCF-7 | 94300 | 12910/31605 | 0.07/0.26 | -61.5 | -61.0 | -16.65 | FALSE |
| SRX7030924 | TFs and others | KDM4C | Breast | MCF-7 | 2748 | 1797/31605 | -0.25/0.26 | -61.5 | -61.0 | -16.65 | FALSE |
| SRX190345 | TFs and others | CEBPB | Breast | MCF-7 | 52521 | 6726/31605 | -0.00/0.26 | -61.5 | -61.0 | -16.65 | FALSE |
| SRX4611701 | TFs and others | ESR1 | Breast | MCF-7 | 53495 | 8306/31605 | 0.02/0.26 | -61.5 | -61.0 | -16.64 | FALSE |
| SRX4815193 | TFs and others | TLE3 | Breast | SUM 149PT | 42663 | 7268/31605 | 0.01/0.26 | -61.5 | -61.0 | -16.64 | FALSE |
| SRX3285598 | TFs and others | KDM5B | Breast | TAMR | 11776 | 5067/31605 | -0.04/0.26 | -61.4 | -60.9 | -16.64 | FALSE |
| SRX4611722 | TFs and others | ESR1 | Breast | MCF-7 | 61825 | 8806/31605 | 0.03/0.26 | -61.4 | -60.9 | -16.64 | FALSE |
| SRX25289337 | TFs and others | GRHL2 | Breast | MCF-7 | 11027 | 2795/31605 | -0.15/0.26 | -61.4 | -60.9 | -16.63 | FALSE |
| SRX968968 | TFs and others | ESR1 | Breast | MCF-7 | 1643 | 307/31605 | -0.96/0.26 | -61.3 | -60.8 | -16.62 | FALSE |
| SRX8111166 | TFs and others | GFP | Breast | MCF 10A | 4156 | 2527/31605 | -0.17/0.26 | -61.3 | -60.8 | -16.62 | FALSE |
| SRX1787653 | TFs and others | FOXA1 | Breast | MCF-7 | 39443 | 3776/31605 | -0.09/0.26 | -61.2 | -60.7 | -16.61 | FALSE |
| SRX4611733 | TFs and others | ESR1 | Breast | MCF-7 | 48621 | 7960/31605 | 0.02/0.26 | -61.2 | -60.7 | -16.60 | FALSE |
| SRX4611694 | TFs and others | ESR1 | Breast | MCF-7 | 60017 | 8926/31605 | 0.03/0.26 | -61.1 | -60.6 | -16.60 | FALSE |
| SRX6777397 | TFs and others | ESR1 | Breast | MCF-7 | 2566 | 272/31605 | -1.03/0.26 | -61.0 | -60.5 | -16.58 | FALSE |
| SRX10468636 | TFs and others | FOXA1 | Breast | Breast cancer cells | 54821 | 5240/31605 | -0.04/0.26 | -61.0 | -60.5 | -16.58 | FALSE |
| SRX1117697 | TFs and others | KDM5C | Breast | ZR-75-30 | 6366 | 2708/31605 | -0.15/0.26 | -61.0 | -60.5 | -16.58 | FALSE |
| SRX4611730 | TFs and others | ESR1 | Breast | MCF-7 | 49587 | 8549/31605 | 0.03/0.26 | -61.0 | -60.5 | -16.58 | FALSE |
| SRX4611706 | TFs and others | ESR1 | Breast | MCF-7 | 62088 | 8675/31605 | 0.03/0.26 | -60.9 | -60.4 | -16.57 | FALSE |
| SRX4611726 | TFs and others | ESR1 | Breast | MCF-7 | 56860 | 8196/31605 | 0.02/0.26 | -60.9 | -60.4 | -16.56 | FALSE |
| SRX1961164 | TFs and others | FOXA1 | Breast | T-47D | 34137 | 4216/31605 | -0.07/0.26 | -60.8 | -60.3 | -16.55 | FALSE |
| SRX3546801 | TFs and others | Epitope tags | Breast | MDA-MB-231 | 5232 | 978/31605 | -0.42/0.26 | -60.5 | -60.0 | -16.50 | FALSE |
| SRX16318945 | TFs and others | MYC | Breast | MDA-MB-231 | 6070 | 2665/31605 | -0.15/0.26 | -60.4 | -59.9 | -16.50 | FALSE |
| SRX18191641 | TFs and others | NR2F2 | Breast | MCF-7 | 14191 | 1967/31605 | -0.22/0.26 | -60.4 | -59.9 | -16.50 | FALSE |
| SRX1012660 | TFs and others | PGR | Breast | MCF-7 | 11938 | 1012/31605 | -0.41/0.26 | -60.4 | -59.9 | -16.49 | FALSE |
| SRX185909 | TFs and others | FOXM1 | Breast | MCF-7 | 1046 | 204/31605 | -1.22/0.26 | -60.4 | -59.9 | -16.49 | FALSE |
| SRX7030925 | TFs and others | KDM4C | Breast | MCF-7 | 2400 | 1839/31605 | -0.24/0.26 | -60.1 | -59.6 | -16.46 | FALSE |
| SRX7865932 | TFs and others | BRD4 | Breast | MDA-MB-231 | 15875 | 3186/31605 | -0.12/0.26 | -60.1 | -59.6 | -16.46 | FALSE |
| SRX1161169 | TFs and others | ESR1 | Breast | MCF-7 | 3399 | 204/31605 | -1.22/0.26 | -60.1 | -59.6 | -16.46 | FALSE |
| SRX4611691 | TFs and others | ESR1 | Breast | MCF-7 | 57352 | 9404/31605 | 0.04/0.26 | -60.1 | -59.6 | -16.45 | FALSE |
| SRX3546797 | TFs and others | Epitope tags | Breast | MDA-MB-231 | 10846 | 1951/31605 | -0.22/0.26 | -60.0 | -59.5 | -16.44 | FALSE |
| SRX3229141 | TFs and others | ESR1 | Breast | Breast cancer | 1918 | 351/31605 | -0.87/0.26 | -60.0 | -59.5 | -16.44 | FALSE |
| SRX2578893 | TFs and others | NCOA1 | Breast | MDA-MB-231 | 5551 | 1022/31605 | -0.40/0.26 | -60.0 | -59.5 | -16.44 | FALSE |
| SRX3285606 | TFs and others | KDM5B | Breast | FULR | 15118 | 4876/31605 | -0.05/0.26 | -59.9 | -59.5 | -16.43 | FALSE |
| SRX3110709 | TFs and others | ESR1 | Breast | MCF-7 | 1126 | 433/31605 | -0.76/0.26 | -59.9 | -59.4 | -16.42 | FALSE |
| SRX4611710 | TFs and others | ESR1 | Breast | MCF-7 | 49968 | 8075/31605 | 0.02/0.26 | -59.8 | -59.3 | -16.42 | FALSE |
| SRX4611731 | TFs and others | ESR1 | Breast | MCF-7 | 56404 | 8266/31605 | 0.02/0.26 | -59.8 | -59.3 | -16.42 | FALSE |
| SRX10468634 | TFs and others | FOXA1 | Breast | Breast cancer cells | 55587 | 5490/31605 | -0.03/0.26 | -59.8 | -59.3 | -16.42 | FALSE |
| SRX7644283 | TFs and others | CPSF3L | Breast | MCF-7 | 3427 | 2739/31605 | -0.15/0.26 | -59.8 | -59.3 | -16.41 | FALSE |
| SRX8083098 | TFs and others | FOXA1 | Breast | CAMA-1 | 60991 | 5503/31605 | -0.03/0.26 | -59.7 | -59.2 | -16.40 | FALSE |
| SRX188955 | TFs and others | CTCF | Breast | MCF-7 | 88614 | 11831/31605 | 0.06/0.26 | -59.6 | -59.2 | -16.39 | FALSE |
| SRX4611695 | TFs and others | ESR1 | Breast | MCF-7 | 61935 | 9587/31605 | 0.04/0.26 | -59.6 | -59.1 | -16.39 | FALSE |
| SRX7644277 | TFs and others | METTL14 | Breast | MCF-7 | 5504 | 2752/31605 | -0.14/0.26 | -59.6 | -59.1 | -16.38 | FALSE |
| SRX312100 | TFs and others | Epitope tags | Breast | MCF-7 | 2457 | 851/31605 | -0.46/0.26 | -59.5 | -59.0 | -16.36 | FALSE |
| SRX13427192 | TFs and others | ESR1 | Breast | MDA-MB-453 | 61174 | 6610/31605 | -0.00/0.26 | -59.5 | -59.0 | -16.36 | FALSE |
| SRX1161189 | TFs and others | FOXA1 | Breast | ZR-75-1 | 62733 | 5615/31605 | -0.02/0.26 | -59.4 | -58.9 | -16.36 | FALSE |
| SRX2871348 | TFs and others | FOXA1 | Breast | T-47D | 28048 | 3754/31605 | -0.09/0.26 | -59.4 | -58.9 | -16.36 | FALSE |
| SRX5432861 | TFs and others | MAX | Breast | MDA-MB-231 | 2901 | 1935/31605 | -0.22/0.26 | -59.3 | -58.8 | -16.34 | FALSE |
| SRX25289339 | TFs and others | GRHL2 | Breast | MCF-7 | 13187 | 3163/31605 | -0.12/0.26 | -59.3 | -58.8 | -16.34 | FALSE |
| SRX1787657 | TFs and others | GRHL2 | Breast | MCF-7 | 31663 | 4302/31605 | -0.06/0.26 | -59.2 | -58.7 | -16.33 | FALSE |
| SRX4611718 | TFs and others | ESR1 | Breast | MCF-7 | 50003 | 8473/31605 | 0.03/0.26 | -59.2 | -58.7 | -16.32 | FALSE |
| SRX10045340 | TFs and others | BAP1 | Breast | CAL-51 | 2198 | 1971/31605 | -0.22/0.26 | -59.2 | -58.7 | -16.32 | FALSE |
| SRX9912218 | TFs and others | FOXA1 | Breast | T-47D | 24982 | 4108/31605 | -0.07/0.26 | -59.1 | -58.7 | -16.32 | FALSE |
| SRX24428395 | TFs and others | Epitope tags | Breast | T-47D | 5843 | 2242/31605 | -0.19/0.26 | -59.1 | -58.6 | -16.31 | FALSE |
| SRX2578881 | TFs and others | ESR1 | Breast | MDA-MB-231 | 17524 | 3838/31605 | -0.08/0.26 | -59.1 | -58.6 | -16.31 | FALSE |
| SRX20282968 | TFs and others | AHR | Breast | MCF-7 | 7729 | 1267/31605 | -0.33/0.26 | -58.9 | -58.4 | -16.29 | FALSE |
| SRX4611724 | TFs and others | ESR1 | Breast | MCF-7 | 57779 | 8741/31605 | 0.03/0.26 | -58.9 | -58.4 | -16.28 | FALSE |
| SRX4611713 | TFs and others | ESR1 | Breast | MCF-7 | 63614 | 9264/31605 | 0.04/0.26 | -58.8 | -58.4 | -16.28 | FALSE |
| SRX3931058 | TFs and others | GRHL2 | Breast | MCF-7 | 32073 | 5344/31605 | -0.03/0.26 | -58.8 | -58.3 | -16.27 | FALSE |
| SRX4611721 | TFs and others | ESR1 | Breast | MCF-7 | 54525 | 7821/31605 | 0.02/0.26 | -58.6 | -58.2 | -16.25 | FALSE |
| SRX2871365 | TFs and others | PGR | Breast | T-47D | 30701 | 3997/31605 | -0.07/0.26 | -58.6 | -58.1 | -16.25 | FALSE |
| SRX190316 | TFs and others | TEAD4 | Breast | MCF-7 | 11863 | 1687/31605 | -0.25/0.26 | -58.6 | -58.1 | -16.24 | FALSE |
| SRX13427191 | TFs and others | ESR1 | Breast | MDA-MB-453 | 64188 | 6263/31605 | -0.01/0.26 | -58.6 | -58.1 | -16.24 | FALSE |
| SRX2578868 | TFs and others | ESR1 | Breast | MDA-MB-231 | 3003 | 1579/31605 | -0.27/0.26 | -58.5 | -58.0 | -16.23 | FALSE |
| SRX3931043 | TFs and others | FOXA1 | Breast | MCF-7 | 53766 | 5644/31605 | -0.02/0.26 | -58.5 | -58.0 | -16.23 | FALSE |
| SRX3285561 | TFs and others | KDM5B | Breast | MCF-7 | 11880 | 4756/31605 | -0.05/0.26 | -58.5 | -58.0 | -16.22 | FALSE |
| SRX100272 | TFs and others | NCOA3 | Breast | H3396 | 1144 | 444/31605 | -0.73/0.26 | -58.4 | -57.9 | -16.21 | FALSE |
| SRX25289342 | TFs and others | GRHL2 | Breast | MCF-7 | 11838 | 2578/31605 | -0.15/0.26 | -58.3 | -57.8 | -16.20 | FALSE |
| SRX1065300 | TFs and others | Epitope tags | Breast | IMEC | 5251 | 2352/31605 | -0.17/0.26 | -58.3 | -57.8 | -16.20 | FALSE |
| SRX484461 | TFs and others | ESR1 | Breast | MDA-MB-231 | 15901 | 2380/31605 | -0.17/0.26 | -58.2 | -57.7 | -16.19 | FALSE |
| SRX8488073 | TFs and others | JUNB | Breast | SUM 229PE | 62440 | 7757/31605 | 0.02/0.26 | -58.2 | -57.7 | -16.18 | FALSE |
| SRX3091905 | TFs and others | ZNF711 | Breast | MCF-7 | 3182 | 2453/31605 | -0.16/0.26 | -58.2 | -57.7 | -16.18 | FALSE |
| SRX9932058 | TFs and others | STAT5A | Breast | MCF-7 | 1840 | 1539/31605 | -0.27/0.26 | -58.2 | -57.7 | -16.18 | FALSE |
| SRX4611705 | TFs and others | ESR1 | Breast | MCF-7 | 56078 | 8279/31605 | 0.03/0.26 | -58.1 | -57.6 | -16.17 | FALSE |
| SRX3931083 | TFs and others | FOXA1 | Breast | MCF-7 | 37899 | 4544/31605 | -0.05/0.26 | -58.0 | -57.6 | -16.16 | FALSE |
| SRX3110740 | TFs and others | ESR1 | Breast | MCF-7 | 56197 | 8278/31605 | 0.03/0.26 | -57.9 | -57.4 | -16.14 | FALSE |
| SRX3410396 | TFs and others | RNF2 | Breast | T-47D | 6084 | 1643/31605 | -0.25/0.26 | -57.8 | -57.4 | -16.14 | FALSE |
| SRX4815191 | TFs and others | TLE3 | Breast | MCF-7 | 53215 | 4913/31605 | -0.04/0.26 | -57.8 | -57.3 | -16.13 | FALSE |
| SRX2871345 | TFs and others | GATA3 | Breast | T-47D | 21974 | 3168/31605 | -0.11/0.26 | -57.7 | -57.3 | -16.12 | FALSE |
| SRX1787680 | TFs and others | KMT2C | Breast | MCF-7 | 14683 | 2201/31605 | -0.18/0.26 | -57.6 | -57.1 | -16.10 | FALSE |
| SRX4611704 | TFs and others | ESR1 | Breast | MCF-7 | 54105 | 8268/31605 | 0.03/0.26 | -57.5 | -57.0 | -16.09 | FALSE |
| SRX8246295 | TFs and others | FGFR1 | Breast | MCF-7 | 3879 | 2814/31605 | -0.13/0.26 | -57.5 | -57.0 | -16.09 | FALSE |
| SRX5203302 | TFs and others | ESR1 | Breast | MCF-7 | 1428 | 84/31605 | -1.99/0.26 | -57.5 | -57.0 | -16.08 | FALSE |
| SRX19024870 | TFs and others | NR3C1 | Breast | MCF-7 | 23697 | 1619/31605 | -0.26/0.26 | -57.5 | -57.0 | -16.08 | FALSE |
| SRX3110730 | TFs and others | ESR1 | Breast | MCF-7 | 59518 | 8666/31605 | 0.03/0.26 | -57.5 | -57.0 | -16.08 | FALSE |
| SRX3110738 | TFs and others | ESR1 | Breast | MCF-7 | 91288 | 10889/31605 | 0.06/0.26 | -57.4 | -57.0 | -16.08 | FALSE |
| SRX490433 | TFs and others | ESR1 | Breast | MCF-7 | 744 | 86/31605 | -1.96/0.26 | -57.3 | -56.9 | -16.06 | FALSE |
| SRX5323818 | TFs and others | STAT3 | Breast | T-47D | 7043 | 2372/31605 | -0.17/0.26 | -57.3 | -56.8 | -16.06 | FALSE |
| SRX2944449 | TFs and others | MYC | Breast | MCF 10A | 3450 | 2335/31605 | -0.17/0.26 | -57.3 | -56.8 | -16.06 | FALSE |
| SRX4663588 | TFs and others | NCOA3 | Breast | MCF-7 | 3885 | 191/31605 | -1.23/0.26 | -57.2 | -56.7 | -16.04 | FALSE |
| SRX3401283 | TFs and others | TEAD1 | Breast | T-47D | 24758 | 4169/31605 | -0.06/0.26 | -57.2 | -56.7 | -16.04 | FALSE |
| SRX7624147 | TFs and others | NR2F2 | Breast | MCF-7 | 9861 | 1172/31605 | -0.34/0.26 | -57.1 | -56.6 | -16.03 | FALSE |
| SRX4611727 | TFs and others | ESR1 | Breast | MCF-7 | 59056 | 8913/31605 | 0.04/0.26 | -57.1 | -56.6 | -16.03 | FALSE |
| SRX4611700 | TFs and others | ESR1 | Breast | MCF-7 | 52438 | 8051/31605 | 0.03/0.26 | -57.0 | -56.5 | -16.02 | FALSE |
| SRX2900156 | TFs and others | KDM6A | Breast | CAL-51 | 2948 | 2165/31605 | -0.19/0.26 | -57.0 | -56.5 | -16.01 | FALSE |
| SRX9322284 | TFs and others | ESR1 | Breast | MCF-7 | 2227 | 179/31605 | -1.28/0.26 | -57.0 | -56.5 | -16.01 | FALSE |
| SRX6896409 | TFs and others | GRHL2 | Breast | T-47D | 22416 | 3863/31605 | -0.07/0.26 | -56.8 | -56.3 | -15.99 | FALSE |
| SRX4611729 | TFs and others | ESR1 | Breast | MCF-7 | 53064 | 8539/31605 | 0.03/0.26 | -56.8 | -56.3 | -15.99 | FALSE |
| SRX4611690 | TFs and others | ESR1 | Breast | MCF-7 | 68058 | 9118/31605 | 0.04/0.26 | -56.8 | -56.3 | -15.99 | FALSE |
| SRX469395 | TFs and others | ESR1 | Breast | MCF-7 | 2635 | 267/31605 | -1.00/0.26 | -56.8 | -56.3 | -15.98 | FALSE |
| SRX4931260 | TFs and others | KMT2A | Breast | MCF-7 | 2378 | 2003/31605 | -0.20/0.26 | -56.8 | -56.3 | -15.98 | FALSE |
| SRX5287705 | TFs and others | YAP1 | Breast | MCF-7 | 2458 | 238/31605 | -1.07/0.26 | -56.8 | -56.3 | -15.98 | FALSE |
| SRX11858168 | TFs and others | TFAP2C | Breast | MCF-7 | 10229 | 2379/31605 | -0.16/0.26 | -56.7 | -56.3 | -15.98 | FALSE |
| SRX5287711 | TFs and others | Biotin | Breast | MCF-7 | 8833 | 2695/31605 | -0.14/0.26 | -56.7 | -56.2 | -15.97 | FALSE |
| SRX4611739 | TFs and others | ESR1 | Breast | MCF-7 | 59817 | 8735/31605 | 0.04/0.26 | -56.7 | -56.2 | -15.97 | FALSE |
| SRX3546798 | TFs and others | Epitope tags | Breast | MDA-MB-231 | 7931 | 1380/31605 | -0.30/0.26 | -56.7 | -56.2 | -15.97 | FALSE |
| SRX15670547 | TFs and others | TFE3 | Breast | MDA-MB-453 | 8202 | 2853/31605 | -0.13/0.26 | -56.6 | -56.1 | -15.95 | FALSE |
| SRX5323829 | TFs and others | FOXA1 | Breast | T-47D | 36184 | 4001/31605 | -0.07/0.26 | -56.6 | -56.1 | -15.95 | FALSE |
| SRX1660877 | TFs and others | TRIM25 | Breast | Breast cancer cells | 15685 | 5312/31605 | -0.03/0.26 | -56.5 | -56.1 | -15.95 | FALSE |
| SRX1012599 | TFs and others | ESR1 | Breast | T-47D | 8580 | 978/31605 | -0.40/0.26 | -56.5 | -56.0 | -15.94 | FALSE |
| SRX5089376 | TFs and others | ARID1A | Breast | MCF-7 | 33724 | 3888/31605 | -0.07/0.26 | -56.4 | -55.9 | -15.93 | FALSE |
| SRX19888705 | TFs and others | NELFE | Breast | MCF-7 | 5654 | 3752/31605 | -0.08/0.26 | -56.3 | -55.8 | -15.91 | FALSE |
| SRX13427193 | TFs and others | ESR1 | Breast | MDA-MB-453 | 65728 | 6942/31605 | 0.01/0.26 | -56.3 | -55.8 | -15.91 | FALSE |
| SRX2020845 | TFs and others | STAT3 | Breast | Breast cancer cells | 7898 | 2974/31605 | -0.12/0.26 | -56.3 | -55.8 | -15.91 | FALSE |
| SRX6860640 | TFs and others | FOXA1 | Breast | T-47D | 38678 | 4563/31605 | -0.05/0.26 | -56.3 | -55.8 | -15.91 | FALSE |
| SRX5172061 | TFs and others | SMARCC1 | Breast | MCF-7 | 10951 | 2487/31605 | -0.15/0.26 | -56.2 | -55.8 | -15.91 | FALSE |
| SRX5287710 | TFs and others | Biotin | Breast | MCF-7 | 10987 | 2895/31605 | -0.12/0.26 | -56.2 | -55.7 | -15.90 | FALSE |
| SRX5089380 | TFs and others | ARID1A | Breast | MCF-7 | 18481 | 2056/31605 | -0.19/0.26 | -56.2 | -55.7 | -15.90 | FALSE |
| SRX7540328 | TFs and others | BRD4 | Breast | MCF-7 | 2732 | 2127/31605 | -0.19/0.26 | -56.1 | -55.7 | -15.89 | FALSE |
| SRX13427195 | TFs and others | ESR1 | Breast | MDA-MB-453 | 54449 | 5992/31605 | -0.01/0.26 | -56.1 | -55.6 | -15.88 | FALSE |
| SRX3541094 | TFs and others | ESR1 | Breast | MCF-7 | 1978 | 90/31605 | -1.89/0.26 | -56.0 | -55.6 | -15.88 | FALSE |
| SRX6712580 | TFs and others | GATA3 | Breast | MCF-7 | 26154 | 2319/31605 | -0.17/0.26 | -56.0 | -55.6 | -15.88 | FALSE |
| SRX10365525 | TFs and others | ESR1 | Breast | MDA-MB-231 | 3850 | 418/31605 | -0.74/0.26 | -56.0 | -55.5 | -15.87 | FALSE |
| SRX2578871 | TFs and others | ESR1 | Breast | MDA-MB-231 | 3435 | 455/31605 | -0.70/0.26 | -56.0 | -55.5 | -15.87 | FALSE |
| SRX3285578 | TFs and others | KDM5B | Breast | MCF-7 | 6819 | 4088/31605 | -0.06/0.26 | -55.9 | -55.5 | -15.86 | FALSE |
| SRX17989902 | TFs and others | SIN3B | Breast | MDA-MB-231 | 1719 | 1887/31605 | -0.21/0.26 | -55.9 | -55.5 | -15.86 | FALSE |
| SRX5323830 | TFs and others | FOXA1 | Breast | T-47D | 37186 | 4277/31605 | -0.06/0.26 | -55.9 | -55.4 | -15.85 | FALSE |
| SRX15430996 | TFs and others | TFE3 | Breast | SK-BR-3 | 61077 | 10106/31605 | 0.05/0.26 | -55.9 | -55.4 | -15.85 | FALSE |
| SRX4611699 | TFs and others | ESR1 | Breast | MCF-7 | 55852 | 8205/31605 | 0.03/0.26 | -55.8 | -55.4 | -15.85 | FALSE |
| SRX3110728 | TFs and others | ESR1 | Breast | MCF-7 | 70878 | 8988/31605 | 0.04/0.26 | -55.8 | -55.3 | -15.84 | FALSE |
| SRX1787656 | TFs and others | GRHL2 | Breast | MCF-7 | 32363 | 4347/31605 | -0.05/0.26 | -55.8 | -55.3 | -15.84 | FALSE |
| SRX11222441 | TFs and others | FOXA1 | Breast | MCF-7 | 40620 | 4825/31605 | -0.04/0.26 | -55.8 | -55.3 | -15.84 | FALSE |
| SRX4611725 | TFs and others | ESR1 | Breast | MCF-7 | 61799 | 8399/31605 | 0.03/0.26 | -55.8 | -55.3 | -15.84 | FALSE |
| SRX6896407 | TFs and others | FOXA1 | Breast | T-47D | 33480 | 4307/31605 | -0.05/0.26 | -55.7 | -55.2 | -15.82 | FALSE |
| SRX4451211 | TFs and others | ESR1 | Breast | MCF-7 | 3442 | 571/31605 | -0.59/0.26 | -55.6 | -55.2 | -15.82 | FALSE |
| SRX883584 | TFs and others | JUN | Breast | MDA-MB-231 | 52349 | 6993/31605 | 0.01/0.26 | -55.6 | -55.1 | -15.82 | FALSE |
| SRX1091828 | TFs and others | CTCFL | Breast | MCF-7 | 17666 | 5944/31605 | -0.01/0.26 | -55.6 | -55.1 | -15.81 | FALSE |
| SRX10859414 | TFs and others | SMARCC1 | Breast | BT-549 | 16560 | 4679/31605 | -0.04/0.26 | -55.5 | -55.0 | -15.80 | FALSE |
| SRX490432 | TFs and others | ESR1 | Breast | MCF-7 | 655 | 79/31605 | -2.02/0.26 | -55.4 | -55.0 | -15.79 | FALSE |
| SRX2460062 | TFs and others | ESR1 | Breast | Breast cancer cells | 1088 | 163/31605 | -1.33/0.26 | -55.4 | -54.9 | -15.78 | FALSE |
| SRX3321935 | TFs and others | GABPA | Breast | MCF-7 | 2565 | 2268/31605 | -0.17/0.26 | -55.2 | -54.8 | -15.76 | FALSE |
| SRX10119367 | TFs and others | WWTR1 | Breast | MCF 10A | 49474 | 6264/31605 | 0.00/0.26 | -55.2 | -54.7 | -15.76 | FALSE |
| SRX673721 | TFs and others | Biotin | Breast | MCF-7 | 3558 | 1461/31605 | -0.27/0.26 | -55.1 | -54.6 | -15.74 | FALSE |
| SRX8488088 | TFs and others | JUNB | Breast | SUM 229PE | 65257 | 8235/31605 | 0.03/0.26 | -55.0 | -54.5 | -15.73 | FALSE |
| SRX3931054 | TFs and others | FOXA1 | Breast | MCF-7 | 50496 | 5328/31605 | -0.02/0.26 | -55.0 | -54.5 | -15.72 | FALSE |
| SRX5089382 | TFs and others | ARID1A | Breast | MCF-7 | 29513 | 3659/31605 | -0.08/0.26 | -55.0 | -54.5 | -15.72 | FALSE |
| SRX4611720 | TFs and others | ESR1 | Breast | MCF-7 | 52933 | 7439/31605 | 0.02/0.26 | -54.9 | -54.5 | -15.72 | FALSE |
| SRX6860635 | TFs and others | FOXA1 | Breast | T-47D | 63430 | 6135/31605 | -0.00/0.26 | -54.8 | -54.3 | -15.70 | FALSE |
| SRX10205607 | TFs and others | YAP1 | Breast | CAL-120 | 20173 | 4135/31605 | -0.06/0.26 | -54.8 | -54.3 | -15.70 | FALSE |
| SRX667411 | TFs and others | Epitope tags | Breast | MCF-7 | 23474 | 8670/31605 | 0.04/0.26 | -54.7 | -54.3 | -15.69 | FALSE |
| SRX3285605 | TFs and others | KDM5B | Breast | FULR | 15720 | 6309/31605 | 0.00/0.26 | -54.5 | -54.1 | -15.66 | FALSE |
| SRX4611698 | TFs and others | ESR1 | Breast | MCF-7 | 56133 | 8118/31605 | 0.03/0.26 | -54.4 | -53.9 | -15.64 | FALSE |
| SRX10045331 | TFs and others | ASXL1 | Breast | CAL-51 | 2574 | 2040/31605 | -0.19/0.26 | -54.3 | -53.9 | -15.63 | FALSE |
| SRX1660875 | TFs and others | TRIM25 | Breast | Breast cancer cells | 97872 | 11235/31605 | 0.07/0.26 | -54.3 | -53.8 | -15.62 | FALSE |
| SRX24151138 | TFs and others | ESR1 | Breast | MCF-7 | 1339 | 67/31605 | -2.19/0.26 | -54.3 | -53.8 | -15.62 | FALSE |
| SRX3110743 | TFs and others | ESR1 | Breast | MCF-7 | 71548 | 8765/31605 | 0.04/0.26 | -54.2 | -53.7 | -15.61 | FALSE |
| SRX23740437 | TFs and others | ZBTB7A | Breast | SUM 149PT | 16799 | 6827/31605 | 0.01/0.26 | -54.1 | -53.6 | -15.59 | FALSE |
| SRX8949280 | TFs and others | NCOA3 | Breast | ZR-75-1 | 54667 | 4233/31605 | -0.05/0.26 | -54.1 | -53.6 | -15.59 | FALSE |
| SRX673718 | TFs and others | Biotin | Breast | MCF-7 | 8010 | 2141/31605 | -0.18/0.26 | -54.0 | -53.6 | -15.58 | FALSE |
| SRX19210523 | TFs and others | RELA | Breast | SUM 149PT | 11332 | 4132/31605 | -0.06/0.26 | -53.9 | -53.5 | -15.57 | FALSE |
| SRX4663591 | TFs and others | NCOA3 | Breast | MCF-7 | 4161 | 220/31605 | -1.09/0.26 | -53.9 | -53.4 | -15.56 | FALSE |
| SRX2194258 | TFs and others | BRD4 | Breast | HCC1806 | 7173 | 2783/31605 | -0.12/0.26 | -53.8 | -53.4 | -15.55 | FALSE |
| SRX8083087 | TFs and others | FGFR1 | Breast | CAMA-1 | 4638 | 3241/31605 | -0.09/0.26 | -53.8 | -53.3 | -15.55 | FALSE |
| SRX15221369 | TFs and others | GRHL2 | Breast | MCF-7 | 17276 | 2940/31605 | -0.11/0.26 | -53.8 | -53.3 | -15.55 | FALSE |
| SRX3110733 | TFs and others | ESR1 | Breast | MCF-7 | 66513 | 8316/31605 | 0.04/0.26 | -53.8 | -53.3 | -15.55 | FALSE |
| SRX3931073 | TFs and others | FOXA1 | Breast | MCF-7 | 37975 | 4487/31605 | -0.04/0.26 | -53.7 | -53.2 | -15.53 | FALSE |
| SRX4611716 | TFs and others | ESR1 | Breast | MCF-7 | 50438 | 7650/31605 | 0.03/0.26 | -53.6 | -53.2 | -15.53 | FALSE |
| SRX3931082 | TFs and others | FOXA1 | Breast | MCF-7 | 32650 | 3936/31605 | -0.06/0.26 | -53.6 | -53.1 | -15.52 | FALSE |
| SRX1012600 | TFs and others | ESR1 | Breast | T-47D | 7439 | 885/31605 | -0.41/0.26 | -53.5 | -53.0 | -15.50 | FALSE |
| SRX8520796 | TFs and others | STAT3 | Breast | MCF-7 | 7138 | 1689/31605 | -0.23/0.26 | -53.4 | -53.0 | -15.50 | FALSE |
| SRX1012602 | TFs and others | ESR1 | Breast | T-47D | 8369 | 1020/31605 | -0.37/0.26 | -53.4 | -52.9 | -15.49 | FALSE |
| SRX20282970 | TFs and others | AHR | Breast | MCF-7 | 5706 | 780/31605 | -0.45/0.26 | -53.3 | -52.8 | -15.48 | FALSE |
| SRX4671295 | TFs and others | RXRA | Breast | NAMEC8 | 21944 | 3606/31605 | -0.07/0.26 | -53.3 | -52.8 | -15.47 | FALSE |
| SRX4611734 | TFs and others | ESR1 | Breast | MCF-7 | 61096 | 8722/31605 | 0.04/0.26 | -53.3 | -52.8 | -15.47 | FALSE |
| SRX3931055 | TFs and others | FOXA1 | Breast | MCF-7 | 46492 | 4952/31605 | -0.03/0.26 | -53.2 | -52.8 | -15.47 | FALSE |
| SRX490437 | TFs and others | ESR1 | Breast | MCF-7 | 658 | 68/31605 | -2.15/0.26 | -53.2 | -52.7 | -15.46 | FALSE |
| SRX3110731 | TFs and others | ESR1 | Breast | MCF-7 | 79526 | 10279/31605 | 0.06/0.26 | -53.2 | -52.7 | -15.46 | FALSE |
| SRX19024868 | TFs and others | NR3C1 | Breast | MCF-7 | 16308 | 1051/31605 | -0.36/0.26 | -53.1 | -52.7 | -15.45 | FALSE |
| SRX490429 | TFs and others | BRD4 | Breast | MCF-7 | 1883 | 1572/31605 | -0.24/0.26 | -53.1 | -52.6 | -15.45 | FALSE |
| SRX10306473 | TFs and others | FOXA1 | Breast | T-47D | 36191 | 3985/31605 | -0.06/0.26 | -53.0 | -52.6 | -15.43 | FALSE |
| SRX4611693 | TFs and others | ESR1 | Breast | MCF-7 | 49422 | 8176/31605 | 0.04/0.26 | -53.0 | -52.5 | -15.43 | FALSE |
| SRX3931081 | TFs and others | FOXA1 | Breast | MCF-7 | 28730 | 3511/31605 | -0.08/0.26 | -53.0 | -52.5 | -15.43 | FALSE |
| SRX6860646 | TFs and others | FOXA1 | Breast | T-47D | 47996 | 5016/31605 | -0.02/0.26 | -53.0 | -52.5 | -15.43 | FALSE |
| SRX15221378 | TFs and others | GRHL2 | Breast | MCF-7 | 17835 | 3328/31605 | -0.09/0.26 | -53.0 | -52.5 | -15.43 | FALSE |
| SRX4611714 | TFs and others | ESR1 | Breast | MCF-7 | 60881 | 8482/31605 | 0.04/0.26 | -53.0 | -52.5 | -15.43 | FALSE |
| SRX18191646 | TFs and others | ESR1 | Breast | MCF-7 | 2801 | 336/31605 | -0.82/0.26 | -53.0 | -52.5 | -15.42 | FALSE |
| SRX8850928 | TFs and others | Epitope tags | Breast | MCF 10A | 5871 | 2211/31605 | -0.16/0.26 | -52.9 | -52.5 | -15.42 | FALSE |
| SRX2423813 | TFs and others | MTA1 | Breast | MCF-7 | 3978 | 1659/31605 | -0.23/0.26 | -52.9 | -52.4 | -15.42 | FALSE |
| SRX2378695 | TFs and others | AHR | Breast | MCF-7 | 23343 | 2980/31605 | -0.11/0.26 | -52.9 | -52.4 | -15.42 | FALSE |
| SRX673712 | TFs and others | Biotin | Breast | MCF-7 | 4133 | 1200/31605 | -0.31/0.26 | -52.9 | -52.4 | -15.41 | FALSE |
| SRX20282957 | TFs and others | AHR | Breast | MCF-7 | 3185 | 237/31605 | -1.03/0.26 | -52.8 | -52.4 | -15.41 | FALSE |
| SRX1161202 | TFs and others | FOXA1 | Breast | T-47D | 30273 | 3580/31605 | -0.07/0.26 | -52.8 | -52.4 | -15.41 | FALSE |
| SRX11120887 | TFs and others | EP300 | Breast | MCF-7 | 7230 | 1407/31605 | -0.27/0.26 | -52.8 | -52.4 | -15.40 | FALSE |
| SRX15221376 | TFs and others | GRHL2 | Breast | MCF-7 | 15107 | 2729/31605 | -0.12/0.26 | -52.7 | -52.2 | -15.38 | FALSE |
| SRX14612991 | TFs and others | FOXA1 | Breast | T-47D | 30807 | 3471/31605 | -0.08/0.26 | -52.6 | -52.2 | -15.38 | FALSE |
| SRX4663590 | TFs and others | NCOA3 | Breast | MCF-7 | 5134 | 306/31605 | -0.87/0.26 | -52.6 | -52.2 | -15.37 | FALSE |
| SRX3722703 | TFs and others | ESR1 | Breast | Breast cancer cells | 63437 | 8918/31605 | 0.05/0.26 | -52.6 | -52.1 | -15.37 | FALSE |
| SRX8803196 | TFs and others | MYC | Breast | MCF-7 | 3324 | 1752/31605 | -0.21/0.26 | -52.5 | -52.1 | -15.36 | FALSE |
| SRX15221364 | TFs and others | GRHL2 | Breast | MCF-7 | 14388 | 2647/31605 | -0.13/0.26 | -52.4 | -52.0 | -15.34 | FALSE |
| SRX3229190 | TFs and others | PGR | Breast | Breast cancer | 12012 | 2128/31605 | -0.17/0.26 | -52.4 | -51.9 | -15.34 | FALSE |
| SRX6860627 | TFs and others | FOXA1 | Breast | T-47D | 40154 | 4338/31605 | -0.04/0.26 | -52.4 | -51.9 | -15.34 | FALSE |
| SRX11149845 | TFs and others | GATA3 | Breast | MDA-MB-453 | 98479 | 6779/31605 | 0.02/0.26 | -52.4 | -51.9 | -15.34 | FALSE |
| SRX15221375 | TFs and others | GRHL2 | Breast | MCF-7 | 16196 | 2908/31605 | -0.11/0.26 | -52.2 | -51.8 | -15.31 | FALSE |
| SRX3447355 | TFs and others | ESR1 | Breast | MCF-7 | 48184 | 5797/31605 | -0.00/0.26 | -52.1 | -51.7 | -15.30 | FALSE |
| SRX4815192 | TFs and others | TLE3 | Breast | MCF-7 | 53138 | 5088/31605 | -0.02/0.26 | -52.1 | -51.7 | -15.30 | FALSE |
| SRX5457549 | TFs and others | GATA3 | Breast | MCF-7 | 25676 | 3226/31605 | -0.09/0.26 | -52.1 | -51.6 | -15.29 | FALSE |
| SRX2871349 | TFs and others | FOXA1 | Breast | T-47D | 25279 | 3290/31605 | -0.09/0.26 | -52.1 | -51.6 | -15.29 | FALSE |
| SRX2194237 | TFs and others | MED1 | Breast | SUM 159PT | 10466 | 3852/31605 | -0.06/0.26 | -52.0 | -51.6 | -15.29 | FALSE |
| SRX3322437 | TFs and others | CTCF | Breast | Breast epithelium | 26451 | 6000/31605 | 0.00/0.26 | -52.0 | -51.6 | -15.29 | FALSE |
| SRX4611692 | TFs and others | ESR1 | Breast | MCF-7 | 52573 | 7863/31605 | 0.03/0.26 | -52.0 | -51.5 | -15.28 | FALSE |
| SRX3447360 | TFs and others | ESR1 | Breast | MCF-7 | 73552 | 8931/31605 | 0.05/0.26 | -51.9 | -51.5 | -15.27 | FALSE |
| SRX10721430 | TFs and others | Epitope tags | Breast | MCF-7 | 10860 | 704/31605 | -0.48/0.26 | -51.9 | -51.5 | -15.27 | FALSE |
| SRX4611689 | TFs and others | ESR1 | Breast | MCF-7 | 54113 | 8034/31605 | 0.04/0.26 | -51.9 | -51.4 | -15.26 | FALSE |
| SRX2912065 | TFs and others | ESR1 | Breast | MCF-7 | 4452 | 2053/31605 | -0.18/0.26 | -51.8 | -51.4 | -15.26 | FALSE |
| SRX3322693 | TFs and others | NFIB | Breast | MCF-7 | 9648 | 1603/31605 | -0.23/0.26 | -51.8 | -51.3 | -15.25 | FALSE |
| SRX8080572 | TFs and others | CTCF | Breast | Mammary gland ductal carcinoma | 33878 | 7462/31605 | 0.03/0.26 | -51.7 | -51.2 | -15.24 | FALSE |
| SRX6860695 | TFs and others | FOXA1 | Breast | T-47D | 42640 | 4707/31605 | -0.03/0.26 | -51.7 | -51.2 | -15.23 | FALSE |
| SRX13340686 | TFs and others | GATA3 | Breast | T-47D | 20368 | 3084/31605 | -0.10/0.26 | -51.6 | -51.2 | -15.22 | FALSE |
| SRX3931094 | TFs and others | FOXA1 | Breast | MCF-7 | 29749 | 3636/31605 | -0.07/0.26 | -51.6 | -51.1 | -15.22 | FALSE |
| SRX25289351 | TFs and others | GRHL2 | Breast | MCF-7 | 11327 | 2459/31605 | -0.14/0.26 | -51.6 | -51.1 | -15.22 | FALSE |
| SRX15221379 | TFs and others | GRHL2 | Breast | MCF-7 | 15836 | 2770/31605 | -0.12/0.26 | -51.6 | -51.1 | -15.22 | FALSE |
| SRX1012619 | TFs and others | PGR | Breast | T-47D | 37187 | 4895/31605 | -0.02/0.26 | -51.6 | -51.1 | -15.22 | FALSE |
| SRX7114560 | TFs and others | Epitope tags | Breast | MCF-7 | 45342 | 6631/31605 | 0.02/0.26 | -51.5 | -51.1 | -15.21 | FALSE |
| SRX4611738 | TFs and others | ESR1 | Breast | MCF-7 | 60084 | 9796/31605 | 0.06/0.26 | -51.5 | -51.1 | -15.21 | FALSE |
| SRX10721431 | TFs and others | Epitope tags | Breast | MCF-7 | 10869 | 736/31605 | -0.46/0.26 | -51.5 | -51.0 | -15.21 | FALSE |
| SRX055310 | TFs and others | KDM5B | Breast | MCF-7 | 1969 | 1735/31605 | -0.21/0.26 | -51.5 | -51.0 | -15.20 | FALSE |
| SRX18191640 | TFs and others | NR2F2 | Breast | MCF-7 | 7363 | 853/31605 | -0.41/0.26 | -51.4 | -51.0 | -15.20 | FALSE |
| SRX5089429 | TFs and others | SMARCB1 | Breast | MCF-7 | 11871 | 1559/31605 | -0.24/0.26 | -51.4 | -51.0 | -15.20 | FALSE |
| SRX10205601 | TFs and others | YAP1 | Breast | CAL-120 | 17104 | 3987/31605 | -0.05/0.26 | -51.4 | -50.9 | -15.19 | FALSE |
| SRX186813 | TFs and others | ESR1 | Breast | Breast cancer | 3178 | 272/31605 | -0.92/0.26 | -51.3 | -50.8 | -15.17 | FALSE |
| SRX647365 | TFs and others | ESR1 | Breast | MCF 10A | 5352 | 2298/31605 | -0.15/0.26 | -51.2 | -50.7 | -15.16 | FALSE |
| SRX9419952 | TFs and others | FOXA1 | Breast | SK-BR-3 | 45280 | 5209/31605 | -0.01/0.26 | -50.7 | -50.2 | -15.08 | FALSE |
| SRX13340681 | TFs and others | GATA3 | Breast | T-47D | 16208 | 2546/31605 | -0.13/0.26 | -50.7 | -50.2 | -15.08 | FALSE |
| SRX5405697 | TFs and others | TFAP2C | Breast | HCC1954 | 18495 | 3419/31605 | -0.08/0.26 | -50.7 | -50.2 | -15.08 | FALSE |
| SRX25289350 | TFs and others | GRHL2 | Breast | MCF-7 | 10923 | 2356/31605 | -0.14/0.26 | -50.7 | -50.2 | -15.08 | FALSE |
| SRX13340683 | TFs and others | GATA3 | Breast | T-47D | 17843 | 2564/31605 | -0.13/0.26 | -50.6 | -50.2 | -15.08 | FALSE |
| SRX3931093 | TFs and others | FOXA1 | Breast | MCF-7 | 28983 | 3569/31605 | -0.07/0.26 | -50.6 | -50.1 | -15.06 | FALSE |
| SRX3931096 | TFs and others | GRHL2 | Breast | MCF-7 | 16036 | 3175/31605 | -0.09/0.26 | -50.5 | -50.1 | -15.06 | FALSE |
| SRX1426078 | TFs and others | PHF2 | Breast | NAMEC8 | 3424 | 2852/31605 | -0.11/0.26 | -50.4 | -49.9 | -15.04 | FALSE |
| SRX1161188 | TFs and others | FOXA1 | Breast | ZR-75-1 | 55673 | 4300/31605 | -0.04/0.26 | -50.3 | -49.9 | -15.03 | FALSE |
| SRX10184539 | TFs and others | FOXA1 | Breast | HCC1954 | 29813 | 4685/31605 | -0.03/0.26 | -50.3 | -49.9 | -15.03 | FALSE |
| SRX15221377 | TFs and others | GRHL2 | Breast | MCF-7 | 15610 | 2805/31605 | -0.11/0.26 | -50.2 | -49.8 | -15.02 | FALSE |
| SRX19024866 | TFs and others | NR3C1 | Breast | MCF-7 | 23419 | 1618/31605 | -0.22/0.26 | -50.1 | -49.7 | -15.00 | FALSE |
| SRX10205600 | TFs and others | YAP1 | Breast | CAL-120 | 17207 | 3846/31605 | -0.05/0.26 | -50.1 | -49.6 | -14.99 | FALSE |
| SRX15221371 | TFs and others | GRHL2 | Breast | MCF-7 | 17584 | 2970/31605 | -0.10/0.26 | -50.0 | -49.6 | -14.98 | FALSE |
| SRX2912047 | TFs and others | ESR1 | Breast | MCF-7 | 1663 | 404/31605 | -0.70/0.26 | -49.9 | -49.5 | -14.97 | FALSE |
| SRX667413 | TFs and others | Epitope tags | Breast | MCF-7 | 41391 | 11510/31605 | 0.08/0.26 | -49.9 | -49.4 | -14.96 | FALSE |
| SRX4611708 | TFs and others | ESR1 | Breast | MCF-7 | 50824 | 7145/31605 | 0.03/0.26 | -49.8 | -49.4 | -14.95 | FALSE |
| SRX3285597 | TFs and others | KDM5B | Breast | TAMR | 7574 | 3575/31605 | -0.07/0.26 | -49.8 | -49.4 | -14.95 | FALSE |
| SRX2636529 | TFs and others | MBD2 | Breast | MCF-7 | 2648 | 988/31605 | -0.35/0.26 | -49.8 | -49.4 | -14.95 | FALSE |
| SRX6853576 | TFs and others | ARNT | Breast | MCF-7 | 1888 | 1437/31605 | -0.25/0.26 | -49.8 | -49.3 | -14.94 | FALSE |
| SRX1116523 | TFs and others | ESR1 | Breast | MCF-7 | 1527 | 1084/31605 | -0.33/0.26 | -49.7 | -49.3 | -14.94 | FALSE |
| SRX1156539 | TFs and others | SMARCA4 | Breast | MDA-MB-231 | 24122 | 6256/31605 | 0.01/0.26 | -49.6 | -49.2 | -14.92 | FALSE |
| SRX055309 | TFs and others | KDM5B | Breast | MCF-7 | 1990 | 1692/31605 | -0.21/0.26 | -49.6 | -49.2 | -14.92 | FALSE |
| SRX9556508 | TFs and others | GATA3 | Breast | MDA-MB-231 | 4392 | 2620/31605 | -0.12/0.26 | -49.6 | -49.1 | -14.92 | FALSE |
| SRX4611735 | TFs and others | ESR1 | Breast | MCF-7 | 57769 | 7702/31605 | 0.04/0.26 | -49.5 | -49.0 | -14.90 | FALSE |
| SRX186821 | TFs and others | ESR1 | Breast | Breast cancer | 2616 | 316/31605 | -0.82/0.26 | -49.5 | -49.0 | -14.90 | FALSE |
| SRX3541106 | TFs and others | ESR1 | Breast | MCF-7 | 1388 | 74/31605 | -1.96/0.26 | -49.4 | -49.0 | -14.89 | FALSE |
| SRX8520792 | TFs and others | STAT3 | Breast | BT-474 | 9451 | 2200/31605 | -0.15/0.26 | -49.3 | -48.8 | -14.87 | FALSE |
| SRX4611717 | TFs and others | ESR1 | Breast | MCF-7 | 56178 | 7976/31605 | 0.04/0.26 | -49.2 | -48.8 | -14.86 | FALSE |
| SRX5323828 | TFs and others | FOXA1 | Breast | T-47D | 30425 | 3741/31605 | -0.06/0.26 | -49.2 | -48.7 | -14.85 | FALSE |
| SRX673756 | TFs and others | FOXA1 | Breast | MCF-7 | 32907 | 3735/31605 | -0.06/0.26 | -49.0 | -48.6 | -14.83 | FALSE |
| SRX9932074 | TFs and others | HDAC6 | Breast | MCF-7 | 659 | 72/31605 | -1.98/0.26 | -49.0 | -48.6 | -14.83 | FALSE |
| SRX1161173 | TFs and others | FOXA1 | Breast | MCF-7 | 46939 | 4181/31605 | -0.04/0.26 | -49.0 | -48.6 | -14.83 | FALSE |
| SRX5251425 | TFs and others | ESR1 | Breast | T-47D | 1507 | 96/31605 | -1.68/0.26 | -49.0 | -48.6 | -14.82 | FALSE |
| SRX1787658 | TFs and others | GRHL2 | Breast | MCF-7 | 36100 | 4127/31605 | -0.04/0.26 | -48.9 | -48.5 | -14.82 | FALSE |
| SRX8850929 | TFs and others | Epitope tags | Breast | MCF 10A | 6278 | 2367/31605 | -0.13/0.26 | -48.9 | -48.5 | -14.81 | FALSE |
| SRX8537744 | TFs and others | FOXA1 | Breast | PE1B | 15171 | 1832/31605 | -0.19/0.26 | -48.9 | -48.5 | -14.81 | FALSE |
| SRX3229159 | TFs and others | ESR1 | Breast | Breast cancer | 1121 | 171/31605 | -1.19/0.26 | -48.7 | -48.3 | -14.78 | FALSE |
| SRX3931095 | TFs and others | FOXA1 | Breast | MCF-7 | 27428 | 3441/31605 | -0.07/0.26 | -48.7 | -48.3 | -14.78 | FALSE |
| SRX5457522 | TFs and others | ZNF574 | Breast | MCF-7 | 3472 | 1523/31605 | -0.23/0.26 | -48.7 | -48.3 | -14.78 | FALSE |
| SRX25289344 | TFs and others | GRHL2 | Breast | MCF-7 | 10785 | 2437/31605 | -0.13/0.26 | -48.7 | -48.2 | -14.78 | FALSE |
| SRX2578897 | TFs and others | NCOA1 | Breast | MDA-MB-231 | 2520 | 615/31605 | -0.51/0.26 | -48.6 | -48.2 | -14.77 | FALSE |
| SRX4611687 | TFs and others | ESR1 | Breast | MCF-7 | 56108 | 7847/31605 | 0.04/0.26 | -48.6 | -48.2 | -14.77 | FALSE |
| SRX25289341 | TFs and others | GRHL2 | Breast | MCF-7 | 10110 | 2175/31605 | -0.15/0.26 | -48.6 | -48.2 | -14.76 | FALSE |
| SRX1161204 | TFs and others | FOXA1 | Breast | T-47D | 26428 | 3303/31605 | -0.07/0.26 | -48.5 | -48.1 | -14.75 | FALSE |
| SRX15221363 | TFs and others | GRHL2 | Breast | MCF-7 | 14945 | 2538/31605 | -0.12/0.26 | -48.5 | -48.0 | -14.74 | FALSE |
| SRX2423398 | TFs and others | RFX1 | Breast | MCF-7 | 17485 | 4367/31605 | -0.03/0.26 | -48.5 | -48.0 | -14.74 | FALSE |
| SRX6860641 | TFs and others | ESR1 | Breast | T-47D | 2145 | 97/31605 | -1.66/0.26 | -48.4 | -47.9 | -14.73 | FALSE |
| SRX3447359 | TFs and others | ESR1 | Breast | MCF-7 | 76816 | 9217/31605 | 0.06/0.26 | -48.2 | -47.8 | -14.71 | FALSE |
| SRX5089422 | TFs and others | SMARCA4 | Breast | MCF-7 | 27277 | 6358/31605 | 0.02/0.26 | -48.2 | -47.8 | -14.71 | FALSE |
| SRX968969 | TFs and others | ESR1 | Breast | MCF-7 | 1650 | 316/31605 | -0.80/0.26 | -48.1 | -47.7 | -14.69 | FALSE |
| SRX1521305 | TFs and others | ZMYND8 | Breast | ZR-75-30 | 6715 | 2467/31605 | -0.12/0.26 | -48.1 | -47.6 | -14.68 | FALSE |
| SRX1131555 | TFs and others | BRD4 | Breast | SUM 1315M02 | 7021 | 2404/31605 | -0.13/0.26 | -48.0 | -47.6 | -14.67 | FALSE |
| SRX4374936 | TFs and others | NOTCH1 | Breast | HCC1599 | 2520 | 1402/31605 | -0.25/0.26 | -47.9 | -47.5 | -14.66 | FALSE |
| SRX8520793 | TFs and others | STAT3 | Breast | HCC1187 | 23012 | 3692/31605 | -0.05/0.26 | -47.9 | -47.4 | -14.65 | FALSE |
| SRX6860690 | TFs and others | FOXA1 | Breast | T-47D | 37228 | 4387/31605 | -0.03/0.26 | -47.8 | -47.4 | -14.65 | FALSE |
| SRX5401866 | TFs and others | PGR | Breast | T-47D | 49742 | 5409/31605 | 0.00/0.26 | -47.8 | -47.3 | -14.64 | FALSE |
| SRX7644285 | TFs and others | CPSF3L | Breast | MCF-7 | 2849 | 2337/31605 | -0.13/0.26 | -47.6 | -47.2 | -14.61 | FALSE |
| SRX10365514 | TFs and others | AR | Breast | MCF-7 | 24208 | 1498/31605 | -0.23/0.26 | -47.6 | -47.1 | -14.60 | FALSE |
| SRX1012742 | TFs and others | ESR1 | Breast | MCF-7 | 1030 | 107/31605 | -1.55/0.26 | -47.6 | -47.1 | -14.60 | FALSE |
| SRX1594205 | TFs and others | CTCF | Breast | MCF-7 | 47701 | 9321/31605 | 0.06/0.26 | -47.5 | -47.1 | -14.60 | FALSE |
| SRX1012601 | TFs and others | ESR1 | Breast | T-47D | 7292 | 816/31605 | -0.40/0.26 | -47.5 | -47.1 | -14.60 | FALSE |
| SRX8716245 | TFs and others | AGO1 | Breast | MCF-7 | 2339 | 77/31605 | -1.87/0.26 | -47.5 | -47.1 | -14.59 | FALSE |
| SRX15221373 | TFs and others | GRHL2 | Breast | MCF-7 | 16569 | 2806/31605 | -0.10/0.26 | -47.5 | -47.0 | -14.58 | FALSE |
| SRX3931072 | TFs and others | FOXA1 | Breast | MCF-7 | 27720 | 3368/31605 | -0.07/0.26 | -47.4 | -47.0 | -14.58 | FALSE |
| SRX11120874 | TFs and others | CTCF | Breast | MCF-7 | 37929 | 7198/31605 | 0.04/0.26 | -47.4 | -47.0 | -14.58 | FALSE |
| SRX4671314 | TFs and others | RARA | Breast | SUM 159PT | 15261 | 3956/31605 | -0.04/0.26 | -47.4 | -47.0 | -14.58 | FALSE |
| SRX4611712 | TFs and others | ESR1 | Breast | MCF-7 | 60946 | 7322/31605 | 0.04/0.26 | -47.2 | -46.8 | -14.55 | FALSE |
| SRX3322720 | TFs and others | TOE1 | Breast | MCF-7 | 5370 | 2370/31605 | -0.13/0.26 | -47.2 | -46.7 | -14.54 | FALSE |
| SRX2578892 | TFs and others | NCOA1 | Breast | MDA-MB-231 | 1836 | 499/31605 | -0.58/0.26 | -47.2 | -46.7 | -14.54 | FALSE |
| SRX523395 | TFs and others | APOBEC3B | Breast | MCF-7 | 1084 | 68/31605 | -2.00/0.26 | -46.9 | -46.5 | -14.50 | FALSE |
| SRX25289336 | TFs and others | GRHL2 | Breast | MCF-7 | 8486 | 2031/31605 | -0.16/0.26 | -46.9 | -46.5 | -14.50 | FALSE |
| SRX2897212 | TFs and others | ESR1 | Breast | Breast cancer cells | 3039 | 2343/31605 | -0.13/0.26 | -46.9 | -46.5 | -14.50 | FALSE |
| SRX1012621 | TFs and others | PGR | Breast | T-47D | 32930 | 3855/31605 | -0.04/0.26 | -46.9 | -46.4 | -14.49 | FALSE |
| SRX1012620 | TFs and others | PGR | Breast | T-47D | 30991 | 3665/31605 | -0.05/0.26 | -46.8 | -46.4 | -14.49 | FALSE |
| SRX2578870 | TFs and others | ESR1 | Breast | MDA-MB-231 | 16236 | 1801/31605 | -0.18/0.26 | -46.8 | -46.4 | -14.48 | FALSE |
| SRX2423837 | TFs and others | CHD1 | Breast | MCF-7 | 3034 | 1757/31605 | -0.19/0.26 | -46.8 | -46.3 | -14.48 | FALSE |
| SRX2944450 | TFs and others | MYC | Breast | MCF 10A | 3215 | 1925/31605 | -0.17/0.26 | -46.6 | -46.2 | -14.46 | FALSE |
| SRX3931074 | TFs and others | FOXA1 | Breast | MCF-7 | 26622 | 3304/31605 | -0.07/0.26 | -46.6 | -46.1 | -14.45 | FALSE |
| SRX5089431 | TFs and others | SMARCB1 | Breast | MCF-7 | 6794 | 790/31605 | -0.40/0.26 | -46.6 | -46.1 | -14.45 | FALSE |
| SRX15221366 | TFs and others | GRHL2 | Breast | MCF-7 | 13448 | 2351/31605 | -0.13/0.26 | -46.5 | -46.0 | -14.43 | FALSE |
| SRX25289340 | TFs and others | GRHL2 | Breast | MCF-7 | 11669 | 2414/31605 | -0.12/0.26 | -46.4 | -46.0 | -14.42 | FALSE |
| SRX8949281 | TFs and others | NCOA3 | Breast | ZR-75-1 | 55286 | 4362/31605 | -0.02/0.26 | -46.4 | -46.0 | -14.42 | FALSE |
| SRX2871344 | TFs and others | GATA3 | Breast | T-47D | 16921 | 2571/31605 | -0.11/0.26 | -46.4 | -45.9 | -14.41 | FALSE |
| SRX10045339 | TFs and others | BAP1 | Breast | CAL-51 | 1520 | 1283/31605 | -0.26/0.26 | -46.3 | -45.9 | -14.40 | FALSE |
| SRX2871346 | TFs and others | FOXA1 | Breast | T-47D | 23701 | 3041/31605 | -0.08/0.26 | -46.3 | -45.9 | -14.40 | FALSE |
| SRX13327987 | TFs and others | GATA3 | Breast | MCF-7 | 7098 | 405/31605 | -0.66/0.26 | -46.2 | -45.8 | -14.39 | FALSE |
| SRX028635 | TFs and others | FOXA1 | Breast | MCF-7 | 34948 | 2874/31605 | -0.09/0.26 | -46.1 | -45.7 | -14.38 | FALSE |
| SRX2578873 | TFs and others | ESR1 | Breast | MDA-MB-231 | 6755 | 1186/31605 | -0.28/0.26 | -46.1 | -45.7 | -14.37 | FALSE |
| SRX3285613 | TFs and others | KDM5B | Breast | C70R | 9503 | 5356/31605 | 0.00/0.26 | -46.1 | -45.7 | -14.37 | FALSE |
| SRX1521303 | TFs and others | EP300 | Breast | ZR-75-30 | 31393 | 4968/31605 | -0.01/0.26 | -46.1 | -45.7 | -14.37 | FALSE |
| SRX5089410 | TFs and others | SMARCA4 | Breast | MCF-7 | 18001 | 3284/31605 | -0.07/0.26 | -46.1 | -45.7 | -14.37 | FALSE |
| SRX15221380 | TFs and others | GRHL2 | Breast | MCF-7 | 14939 | 2558/31605 | -0.11/0.26 | -46.1 | -45.7 | -14.37 | FALSE |
| SRX24351555 | TFs and others | Epitope tags | Breast | MB 157 | 4586 | 2275/31605 | -0.13/0.26 | -46.0 | -45.6 | -14.36 | FALSE |
| SRX6860623 | TFs and others | FOXA1 | Breast | T-47D | 27075 | 3365/31605 | -0.06/0.26 | -45.9 | -45.4 | -14.33 | FALSE |
| SRX9556507 | TFs and others | GATA3 | Breast | MDA-MB-231 | 4470 | 2597/31605 | -0.10/0.26 | -45.8 | -45.4 | -14.33 | FALSE |
| SRX6487055 | TFs and others | FOXA1 | Breast | MCF-7 | 13955 | 1012/31605 | -0.32/0.26 | -45.8 | -45.4 | -14.32 | FALSE |
| SRX185915 | TFs and others | FOXM1 | Breast | MCF-7 | 377 | 94/31605 | -1.64/0.26 | -45.8 | -45.4 | -14.32 | FALSE |
| SRX15221370 | TFs and others | GRHL2 | Breast | MCF-7 | 14907 | 2527/31605 | -0.11/0.26 | -45.7 | -45.2 | -14.30 | FALSE |
| SRX103007 | TFs and others | CTCF | Breast | MCF-7 | 94955 | 11160/31605 | 0.08/0.26 | -45.6 | -45.2 | -14.30 | FALSE |
| SRX7030923 | TFs and others | KDM4B | Breast | MCF-7 | 1222 | 108/31605 | -1.51/0.26 | -45.6 | -45.2 | -14.29 | FALSE |
| SRX150536 | TFs and others | STAT3 | Breast | MCF 10A | 55886 | 6484/31605 | 0.03/0.26 | -45.6 | -45.2 | -14.29 | FALSE |
| SRX1447316 | TFs and others | FOXA1 | Breast | MCF-7 | 34176 | 3641/31605 | -0.05/0.26 | -45.6 | -45.2 | -14.29 | FALSE |
| SRX3322788 | TFs and others | RFX5 | Breast | MCF-7 | 6297 | 2160/31605 | -0.14/0.26 | -45.5 | -45.1 | -14.28 | FALSE |
| SRX10184538 | TFs and others | FOXA1 | Breast | HCC1954 | 27903 | 4368/31605 | -0.02/0.26 | -45.5 | -45.1 | -14.28 | FALSE |
| SRX3322698 | TFs and others | TARDBP | Breast | MCF-7 | 4756 | 1853/31605 | -0.17/0.26 | -45.5 | -45.1 | -14.28 | FALSE |
| SRX150642 | TFs and others | ZNF217 | Breast | MCF-7 | 11368 | 1094/31605 | -0.30/0.26 | -45.5 | -45.1 | -14.28 | FALSE |
| SRX103004 | TFs and others | CTCF | Breast | MCF-7 | 75941 | 10968/31605 | 0.08/0.26 | -45.4 | -44.9 | -14.25 | FALSE |
| SRX1012618 | TFs and others | PGR | Breast | T-47D | 29013 | 3685/31605 | -0.05/0.26 | -45.2 | -44.8 | -14.23 | FALSE |
| SRX667414 | TFs and others | Epitope tags | Breast | MCF-7 | 34079 | 8964/31605 | 0.06/0.26 | -45.1 | -44.7 | -14.21 | FALSE |
| SRX176885 | TFs and others | EP300 | Breast | MCF-7 | 31171 | 3329/31605 | -0.06/0.26 | -45.1 | -44.7 | -14.21 | FALSE |
| SRX19888697 | TFs and others | NELFE | Breast | BT-549 | 3688 | 2718/31605 | -0.09/0.26 | -44.9 | -44.5 | -14.18 | FALSE |
| SRX3011701 | TFs and others | MED12 | Breast | MCF-7 | 1283 | 141/31605 | -1.27/0.26 | -44.9 | -44.5 | -14.17 | FALSE |
| SRX4786563 | TFs and others | KMT2C | Breast | MCF-7 | 4050 | 1983/31605 | -0.15/0.26 | -44.9 | -44.4 | -14.17 | FALSE |
| SRX1161175 | TFs and others | FOXA1 | Breast | MCF-7 | 43494 | 3748/31605 | -0.04/0.26 | -44.8 | -44.4 | -14.16 | FALSE |
| SRX20830449 | TFs and others | Epitope tags | Breast | T-47D | 8144 | 939/31605 | -0.34/0.26 | -44.8 | -44.4 | -14.16 | FALSE |
| SRX13340682 | TFs and others | GATA3 | Breast | T-47D | 13595 | 2198/31605 | -0.13/0.26 | -44.8 | -44.4 | -14.16 | FALSE |
| SRX8716242 | TFs and others | AGO1 | Breast | MCF-7 | 2143 | 71/31605 | -1.90/0.26 | -44.7 | -44.3 | -14.15 | FALSE |
| SRX1787652 | TFs and others | FOXA1 | Breast | MCF-7 | 33639 | 3067/31605 | -0.07/0.26 | -44.7 | -44.3 | -14.15 | FALSE |
| SRX1012622 | TFs and others | PGR | Breast | T-47D | 34370 | 4047/31605 | -0.03/0.26 | -44.6 | -44.2 | -14.13 | FALSE |
| SRX15221365 | TFs and others | GRHL2 | Breast | MCF-7 | 15498 | 2748/31605 | -0.09/0.26 | -44.6 | -44.1 | -14.12 | FALSE |
| SRX3401273 | TFs and others | TRPS1 | Breast | MCF-7 | 27586 | 2358/31605 | -0.12/0.26 | -44.4 | -44.0 | -14.10 | FALSE |
| SRX25289353 | TFs and others | GRHL2 | Breast | MCF-7 | 10727 | 2275/31605 | -0.12/0.26 | -44.4 | -44.0 | -14.09 | FALSE |
| SRX11222437 | TFs and others | FOXA1 | Breast | MCF-7 | 40746 | 4018/31605 | -0.03/0.26 | -44.4 | -44.0 | -14.09 | FALSE |
| SRX5089531 | TFs and others | ARID2 | Breast | MCF-7 | 11897 | 1120/31605 | -0.28/0.26 | -44.3 | -43.9 | -14.09 | FALSE |
| SRX3583212 | TFs and others | CTCF | Breast | MCF-7 | 77040 | 10983/31605 | 0.08/0.26 | -44.3 | -43.9 | -14.08 | FALSE |
| SRX3931032 | TFs and others | FOXA1 | Breast | MCF-7 | 35360 | 3778/31605 | -0.04/0.26 | -44.3 | -43.9 | -14.08 | FALSE |
| SRX2423814 | TFs and others | MTA1 | Breast | MCF-7 | 3671 | 1445/31605 | -0.22/0.26 | -44.3 | -43.9 | -14.08 | FALSE |
| SRX15221372 | TFs and others | GRHL2 | Breast | MCF-7 | 14864 | 2441/31605 | -0.11/0.26 | -44.3 | -43.8 | -14.07 | FALSE |
| SRX11150132 | TFs and others | GATA3 | Breast | Breast cancer cells | 21435 | 2178/31605 | -0.13/0.26 | -44.2 | -43.8 | -14.07 | FALSE |
| SRX4374898 | TFs and others | NOTCH1 | Breast | MB 157 | 2663 | 1277/31605 | -0.25/0.26 | -44.1 | -43.7 | -14.04 | FALSE |
| SRX3554400 | TFs and others | ESR1 | Breast | MDA-MB-134-VI | 1114 | 66/31605 | -1.96/0.26 | -44.1 | -43.6 | -14.04 | FALSE |
| SRX11222442 | TFs and others | FOXA1 | Breast | MCF-7 | 36938 | 4182/31605 | -0.02/0.26 | -44.0 | -43.6 | -14.04 | FALSE |
| SRX1012623 | TFs and others | PGR | Breast | T-47D | 29459 | 3411/31605 | -0.05/0.26 | -44.0 | -43.6 | -14.03 | FALSE |
| SRX100266 | TFs and others | ESR1 | Breast | H3396 | 445 | 215/31605 | -0.97/0.26 | -44.0 | -43.6 | -14.03 | FALSE |
| SRX4611732 | TFs and others | ESR1 | Breast | MCF-7 | 39821 | 8245/31605 | 0.06/0.26 | -44.0 | -43.6 | -14.03 | FALSE |
| SRX2578879 | TFs and others | ESR1 | Breast | MDA-MB-231 | 2564 | 377/31605 | -0.67/0.26 | -44.0 | -43.6 | -14.03 | FALSE |
| SRX8537743 | TFs and others | FOXA1 | Breast | T-47D | 17475 | 2496/31605 | -0.10/0.26 | -44.0 | -43.5 | -14.02 | FALSE |
| SRX5323930 | TFs and others | ESR1 | Breast | Breast cancer cells | 7570 | 668/31605 | -0.44/0.26 | -43.9 | -43.5 | -14.02 | FALSE |
| SRX3931056 | TFs and others | FOXA1 | Breast | MCF-7 | 35979 | 3763/31605 | -0.04/0.26 | -43.6 | -43.2 | -13.97 | FALSE |
| SRX3322593 | TFs and others | CTCF | Breast | Breast epithelium | 26771 | 5680/31605 | 0.02/0.26 | -43.5 | -43.1 | -13.95 | FALSE |
| SRX1156542 | TFs and others | SMARCA4 | Breast | MDA-MB-231 | 17643 | 6094/31605 | 0.03/0.26 | -43.4 | -43.0 | -13.94 | FALSE |
| SRX5089377 | TFs and others | ARID1A | Breast | MCF-7 | 16588 | 1398/31605 | -0.22/0.26 | -43.4 | -43.0 | -13.93 | FALSE |
| SRX1156537 | TFs and others | SMARCA4 | Breast | MDA-MB-231 | 34233 | 6109/31605 | 0.03/0.26 | -43.3 | -42.9 | -13.92 | FALSE |
| SRX6487043 | TFs and others | FOXA1 | Breast | MCF-7 | 13030 | 986/31605 | -0.31/0.26 | -43.2 | -42.8 | -13.90 | FALSE |
| SRX10164649 | TFs and others | ESR1 | Breast | MCF-7 | 2284 | 71/31605 | -1.85/0.26 | -43.1 | -42.7 | -13.88 | FALSE |
| SRX6058755 | TFs and others | CEBPA | Breast | T-47D | 25356 | 3780/31605 | -0.03/0.26 | -43.0 | -42.5 | -13.86 | FALSE |
| SRX5185603 | TFs and others | MED12 | Breast | MCF-7 | 1217 | 79/31605 | -1.74/0.26 | -42.9 | -42.5 | -13.86 | FALSE |
| SRX25289349 | TFs and others | GRHL2 | Breast | MCF-7 | 6975 | 1676/31605 | -0.18/0.26 | -42.9 | -42.5 | -13.86 | FALSE |
| SRX15221374 | TFs and others | GRHL2 | Breast | MCF-7 | 15754 | 2532/31605 | -0.10/0.26 | -42.9 | -42.5 | -13.85 | FALSE |
| SRX5172065 | TFs and others | SMARCA4 | Breast | MCF-7 | 9660 | 2427/31605 | -0.10/0.26 | -42.9 | -42.5 | -13.85 | FALSE |
| SRX8084739 | TFs and others | Epitope tags | Breast | SUM 159PT | 2959 | 1837/31605 | -0.16/0.26 | -42.9 | -42.5 | -13.85 | FALSE |
| SRX18191660 | TFs and others | ESR1 | Breast | MCF-7 | 1374 | 81/31605 | -1.71/0.26 | -42.9 | -42.5 | -13.84 | FALSE |
| SRX2897202 | TFs and others | ESR1 | Breast | Breast cancer cells | 1274 | 300/31605 | -0.77/0.26 | -42.8 | -42.4 | -13.84 | FALSE |
| SRX5534866 | TFs and others | GATA3 | Breast | MCF-7 | 3739 | 3366/31605 | -0.05/0.26 | -42.8 | -42.4 | -13.84 | FALSE |
| SRX4815188 | TFs and others | CTNNB1 | Breast | MCF-7 | 4683 | 2350/31605 | -0.11/0.26 | -42.8 | -42.4 | -13.84 | FALSE |
| SRX20830492 | TFs and others | CTCF | Breast | T-47D | 59449 | 9064/31605 | 0.07/0.26 | -42.8 | -42.3 | -13.83 | FALSE |
| SRX100943 | TFs and others | TCF7L2 | Breast | MCF-7 | 10980 | 1815/31605 | -0.16/0.26 | -42.7 | -42.3 | -13.82 | FALSE |
| SRX1473609 | TFs and others | ESRRA | Breast | BT-474 | 9512 | 1718/31605 | -0.17/0.26 | -42.7 | -42.3 | -13.82 | FALSE |
| SRX3229137 | TFs and others | ESR1 | Breast | Breast cancer | 1472 | 218/31605 | -0.94/0.26 | -42.6 | -42.2 | -13.80 | FALSE |
| SRX15221368 | TFs and others | GRHL2 | Breast | MCF-7 | 13411 | 2266/31605 | -0.12/0.26 | -42.6 | -42.2 | -13.80 | FALSE |
| SRX6831824 | TFs and others | TEAD4 | Breast | MCF 10A | 9625 | 2564/31605 | -0.09/0.26 | -42.5 | -42.1 | -13.79 | FALSE |
| SRX10365515 | TFs and others | AR | Breast | MCF-7 | 5751 | 352/31605 | -0.68/0.26 | -42.4 | -42.0 | -13.77 | FALSE |
| SRX10164631 | TFs and others | ESR1 | Breast | MCF-7 | 2080 | 69/31605 | -1.87/0.26 | -42.3 | -41.9 | -13.76 | FALSE |
| SRX10306480 | TFs and others | RCOR1 | Breast | T-47D | 7978 | 3181/31605 | -0.06/0.26 | -42.3 | -41.8 | -13.74 | FALSE |
| SRX1161198 | TFs and others | ESR1 | Breast | T-47D | 9965 | 1266/31605 | -0.24/0.26 | -42.2 | -41.8 | -13.74 | FALSE |
| SRX3541104 | TFs and others | ESR1 | Breast | MCF-7 | 1570 | 53/31605 | -2.16/0.26 | -42.2 | -41.8 | -13.73 | FALSE |
| SRX5534863 | TFs and others | EP300 | Breast | MCF-7 | 11772 | 3112/31605 | -0.06/0.26 | -42.1 | -41.7 | -13.72 | FALSE |
| SRX4374938 | TFs and others | NOTCH1 | Breast | HCC1599 | 1703 | 1095/31605 | -0.28/0.26 | -42.1 | -41.7 | -13.71 | FALSE |
| SRX4116026 | TFs and others | ESR1 | Breast | Breast cancer cells | 2850 | 757/31605 | -0.38/0.26 | -42.0 | -41.6 | -13.71 | FALSE |
| SRX3931033 | TFs and others | FOXA1 | Breast | MCF-7 | 39534 | 4147/31605 | -0.02/0.26 | -42.0 | -41.6 | -13.70 | FALSE |
| SRX1012614 | TFs and others | EP300 | Breast | T-47D | 8779 | 1778/31605 | -0.16/0.26 | -41.9 | -41.5 | -13.68 | FALSE |
| SRX3322942 | TFs and others | SUZ12 | Breast | MCF-7 | 4334 | 1614/31605 | -0.18/0.26 | -41.8 | -41.4 | -13.67 | FALSE |
| SRX3229192 | TFs and others | PGR | Breast | Breast cancer | 12548 | 1808/31605 | -0.16/0.26 | -41.7 | -41.3 | -13.66 | FALSE |
| SRX3583188 | TFs and others | CTCF | Breast | MCF-7 | 68704 | 10428/31605 | 0.08/0.26 | -41.7 | -41.3 | -13.65 | FALSE |
| SRX2578889 | TFs and others | MED1 | Breast | MDA-MB-231 | 3173 | 1000/31605 | -0.30/0.26 | -41.7 | -41.3 | -13.65 | FALSE |
| SRX6896406 | TFs and others | FOXA1 | Breast | T-47D | 31822 | 3710/31605 | -0.03/0.26 | -41.7 | -41.3 | -13.64 | FALSE |
| SRX4611736 | TFs and others | ESR1 | Breast | MCF-7 | 46664 | 5776/31605 | 0.03/0.26 | -41.6 | -41.2 | -13.64 | FALSE |
| SRX747790 | TFs and others | RELA | Breast | MCF-7 | 1413 | 812/31605 | -0.36/0.26 | -41.6 | -41.2 | -13.64 | FALSE |
| SRX673716 | TFs and others | Biotin | Breast | MCF-7 | 1131 | 104/31605 | -1.46/0.26 | -41.6 | -41.2 | -13.63 | FALSE |
| SRX4374937 | TFs and others | NOTCH1 | Breast | HCC1599 | 1696 | 1085/31605 | -0.27/0.26 | -41.6 | -41.2 | -13.63 | FALSE |
| SRX20830495 | TFs and others | CTCF | Breast | T-47D | 57300 | 9019/31605 | 0.07/0.26 | -41.5 | -41.1 | -13.62 | FALSE |
| SRX3322721 | TFs and others | TOE1 | Breast | MCF-7 | 7508 | 2479/31605 | -0.09/0.26 | -41.5 | -41.0 | -13.61 | FALSE |
| SRX15221367 | TFs and others | GRHL2 | Breast | MCF-7 | 12749 | 2150/31605 | -0.12/0.26 | -41.4 | -41.0 | -13.60 | FALSE |
| SRX1012610 | TFs and others | EP300 | Breast | T-47D | 13918 | 2101/31605 | -0.12/0.26 | -41.4 | -41.0 | -13.59 | FALSE |
| SRX5287700 | TFs and others | MED1 | Breast | MCF-7 | 1015 | 225/31605 | -0.91/0.26 | -41.4 | -41.0 | -13.59 | FALSE |
| SRX3583180 | TFs and others | CTCF | Breast | MCF-7 | 62812 | 8814/31605 | 0.07/0.26 | -41.3 | -40.9 | -13.58 | FALSE |
| SRX190348 | TFs and others | JUND | Breast | MCF-7 | 13323 | 2504/31605 | -0.09/0.26 | -41.2 | -40.8 | -13.57 | FALSE |
| SRX1531793 | TFs and others | NIPBL | Breast | MCF-7 | 3233 | 386/31605 | -0.63/0.26 | -41.2 | -40.8 | -13.56 | FALSE |
| SRX2897218 | TFs and others | ESR1 | Breast | Breast cancer cells | 3901 | 3353/31605 | -0.04/0.26 | -41.2 | -40.8 | -13.56 | FALSE |
| SRX6860651 | TFs and others | FOXA1 | Breast | T-47D | 31815 | 3497/31605 | -0.04/0.26 | -41.2 | -40.8 | -13.56 | FALSE |
| SRX3229135 | TFs and others | NR3C1 | Breast | MCF-7 | 5899 | 424/31605 | -0.59/0.26 | -41.2 | -40.8 | -13.56 | FALSE |
| SRX2880134 | TFs and others | KDM6A | Breast | CAL-51 | 3071 | 1826/31605 | -0.15/0.26 | -41.1 | -40.7 | -13.56 | FALSE |
| SRX2897214 | TFs and others | ESR1 | Breast | Breast cancer cells | 4014 | 3514/31605 | -0.04/0.26 | -41.1 | -40.7 | -13.55 | FALSE |
| SRX3322943 | TFs and others | SUZ12 | Breast | MCF-7 | 3071 | 1115/31605 | -0.26/0.26 | -41.1 | -40.7 | -13.54 | FALSE |
| SRX1787679 | TFs and others | KMT2C | Breast | MCF-7 | 12427 | 1348/31605 | -0.22/0.26 | -41.1 | -40.7 | -13.54 | FALSE |
| SRX7030922 | TFs and others | KDM4B | Breast | MCF-7 | 1595 | 1063/31605 | -0.28/0.26 | -41.0 | -40.6 | -13.54 | FALSE |
| SRX8520799 | TFs and others | STAT3 | Breast | MDA-MB-453 | 8835 | 1845/31605 | -0.15/0.26 | -41.0 | -40.6 | -13.54 | FALSE |
| SRX7262875 | TFs and others | EP300 | Breast | ZR-75-1 | 21883 | 1493/31605 | -0.19/0.26 | -40.9 | -40.5 | -13.52 | FALSE |
| SRX1757996 | TFs and others | MAX | Breast | MDA-MB-468 | 1926 | 1535/31605 | -0.19/0.26 | -40.9 | -40.5 | -13.52 | FALSE |
| SRX10775053 | TFs and others | RNF2 | Breast | T-47D | 1961 | 257/31605 | -0.82/0.26 | -40.8 | -40.4 | -13.49 | FALSE |
| SRX6712569 | TFs and others | CREBBP | Breast | MCF-7 | 3614 | 1528/31605 | -0.19/0.26 | -40.7 | -40.3 | -13.48 | FALSE |
| SRX2578878 | TFs and others | ESR1 | Breast | MDA-MB-231 | 19950 | 2538/31605 | -0.09/0.26 | -40.7 | -40.3 | -13.48 | FALSE |
| SRX673754 | TFs and others | ESR1 | Breast | MCF-7 | 1207 | 115/31605 | -1.35/0.26 | -40.7 | -40.3 | -13.48 | FALSE |
| SRX5493765 | TFs and others | ESR1 | Breast | Breast cancer cells | 840 | 63/31605 | -1.92/0.26 | -40.6 | -40.3 | -13.47 | FALSE |
| SRX8080542 | TFs and others | Epitope tags | Breast | Mammary gland ductal carcinoma | 624 | 86/31605 | -1.60/0.26 | -40.6 | -40.2 | -13.47 | FALSE |
| SRX1585242 | TFs and others | ESR1 | Breast | MCF-7 | 30869 | 3462/31605 | -0.04/0.26 | -40.5 | -40.1 | -13.44 | FALSE |
| SRX3285582 | TFs and others | KDM5B | Breast | MCF-7 | 4990 | 3264/31605 | -0.05/0.26 | -40.5 | -40.1 | -13.44 | FALSE |
| SRX1091827 | TFs and others | CTCFL | Breast | MCF-7 | 12107 | 4700/31605 | 0.00/0.26 | -40.4 | -40.0 | -13.43 | FALSE |
| SRX490430 | TFs and others | BRD4 | Breast | MCF-7 | 1482 | 1155/31605 | -0.25/0.26 | -40.4 | -40.0 | -13.43 | FALSE |
| SRX20282952 | TFs and others | AHR | Breast | MCF-7 | 2120 | 241/31605 | -0.85/0.26 | -40.3 | -39.9 | -13.42 | FALSE |
| SRX3401276 | TFs and others | CTBP2 | Breast | MCF-7 | 7820 | 689/31605 | -0.40/0.26 | -40.3 | -39.9 | -13.41 | FALSE |
| SRX176862 | TFs and others | FOXA1 | Breast | MCF-7 | 73760 | 5913/31605 | 0.03/0.26 | -40.2 | -39.8 | -13.40 | FALSE |
| SRX8520800 | TFs and others | STAT3 | Breast | SUM 159PT | 27775 | 4371/31605 | -0.00/0.26 | -40.2 | -39.8 | -13.39 | FALSE |
| SRX20282963 | TFs and others | AHR | Breast | MCF-7 | 15200 | 1801/31605 | -0.15/0.26 | -40.2 | -39.8 | -13.39 | FALSE |
| SRX4611715 | TFs and others | ESR1 | Breast | MCF-7 | 43715 | 6099/31605 | 0.04/0.26 | -40.1 | -39.7 | -13.38 | FALSE |
| SRX265425 | TFs and others | KDM5B | Breast | SUM 159PT | 13882 | 3509/31605 | -0.03/0.26 | -40.1 | -39.7 | -13.38 | FALSE |
| SRX2745846 | TFs and others | Epitope tags | Breast | MCF 10A | 45453 | 5450/31605 | 0.02/0.26 | -40.1 | -39.7 | -13.38 | FALSE |
| SRX3040148 | TFs and others | STAG2 | Breast | HMEC | 64126 | 9268/31605 | 0.08/0.26 | -40.0 | -39.6 | -13.37 | FALSE |
| SRX1156476 | TFs and others | GATA3 | Breast | MDA-MB-231 | 37598 | 4570/31605 | 0.00/0.26 | -39.9 | -39.5 | -13.35 | FALSE |
| SRX11120873 | TFs and others | CTCF | Breast | MCF-7 | 28607 | 6310/31605 | 0.04/0.26 | -39.9 | -39.5 | -13.34 | FALSE |
| SRX1012613 | TFs and others | EP300 | Breast | T-47D | 9149 | 1737/31605 | -0.15/0.26 | -39.8 | -39.4 | -13.33 | FALSE |
| SRX3322289 | TFs and others | DDX20 | Breast | MCF-7 | 3072 | 1371/31605 | -0.21/0.26 | -39.8 | -39.4 | -13.33 | FALSE |
| SRX1594208 | TFs and others | CTCF | Breast | MCF-7 | 46195 | 8561/31605 | 0.07/0.26 | -39.8 | -39.4 | -13.32 | FALSE |
| SRX3110744 | TFs and others | ESR1 | Breast | MCF-7 | 69529 | 9769/31605 | 0.08/0.26 | -39.7 | -39.4 | -13.32 | FALSE |
| SRX8488074 | TFs and others | JUNB | Breast | SUM 229PE | 48324 | 5538/31605 | 0.03/0.26 | -39.7 | -39.3 | -13.31 | FALSE |
| SRX1156477 | TFs and others | GATA3 | Breast | MDA-MB-231 | 35561 | 4294/31605 | -0.01/0.26 | -39.7 | -39.3 | -13.31 | FALSE |
| SRX371480 | TFs and others | FOXA1 | Breast | MCF-7 | 39135 | 3508/31605 | -0.03/0.26 | -39.7 | -39.3 | -13.30 | FALSE |
| SRX5185605 | TFs and others | MED12 | Breast | MCF-7 | 1210 | 70/31605 | -1.78/0.26 | -39.5 | -39.1 | -13.28 | FALSE |
| SRX265422 | TFs and others | KDM5B | Breast | SUM 185PE | 10410 | 2968/31605 | -0.06/0.26 | -39.5 | -39.1 | -13.28 | FALSE |
| SRX1012639 | TFs and others | EP300 | Breast | MCF-7 | 902 | 64/31605 | -1.87/0.26 | -39.5 | -39.1 | -13.28 | FALSE |
| SRX3229120 | TFs and others | ESR1 | Breast | Breast cancer | 3051 | 674/31605 | -0.40/0.26 | -39.5 | -39.1 | -13.28 | FALSE |
| SRX4374935 | TFs and others | NOTCH1 | Breast | HCC1599 | 1807 | 979/31605 | -0.29/0.26 | -39.4 | -39.0 | -13.27 | FALSE |
| SRX4192708 | TFs and others | Epitope tags | Breast | MDA-MB-231 | 16479 | 8860/31605 | 0.07/0.26 | -39.4 | -39.0 | -13.26 | FALSE |
| SRX10773100 | TFs and others | CTCF | Breast | MB 157 | 34888 | 6566/31605 | 0.05/0.26 | -39.3 | -38.9 | -13.25 | FALSE |
| SRX15430994 | TFs and others | FOXA1 | Breast | SK-BR-3 | 53883 | 4937/31605 | 0.01/0.26 | -39.3 | -38.9 | -13.25 | FALSE |
| SRX099863 | TFs and others | CTCF | Breast | MCF-7 | 51251 | 8827/31605 | 0.07/0.26 | -39.3 | -38.9 | -13.24 | FALSE |
| SRX026529 | TFs and others | ESR1 | Breast | MCF-7 | 986 | 77/31605 | -1.68/0.26 | -39.2 | -38.8 | -13.23 | FALSE |
| SRX3546803 | TFs and others | Epitope tags | Breast | MDA-MB-231 | 1909 | 379/31605 | -0.61/0.26 | -39.2 | -38.8 | -13.22 | FALSE |
| SRX7627555 | TFs and others | ESRRG | Breast | BT-474 | 7823 | 1441/31605 | -0.19/0.26 | -39.2 | -38.8 | -13.22 | FALSE |
| SRX4451210 | TFs and others | ESR1 | Breast | MCF-7 | 2096 | 291/31605 | -0.74/0.26 | -39.2 | -38.8 | -13.22 | FALSE |
| SRX6858250 | TFs and others | HSF1 | Breast | MCF-7 | 24457 | 3871/31605 | -0.02/0.26 | -39.1 | -38.7 | -13.21 | FALSE |
| SRX4815187 | TFs and others | CTNNB1 | Breast | MCF-7 | 4475 | 2113/31605 | -0.11/0.26 | -39.0 | -38.7 | -13.20 | FALSE |
| SRX667410 | TFs and others | Epitope tags | Breast | MCF-7 | 11473 | 5955/31605 | 0.04/0.26 | -38.9 | -38.6 | -13.18 | FALSE |
| SRX6098145 | TFs and others | TRPS1 | Breast | MCF-7 | 18408 | 1787/31605 | -0.14/0.26 | -38.9 | -38.5 | -13.17 | FALSE |
| SRX7121994 | TFs and others | SMARCA4 | Breast | MCF-7 | 14861 | 2900/31605 | -0.06/0.26 | -38.8 | -38.4 | -13.16 | FALSE |
| SRX5202982 | TFs and others | FOXA1 | Breast | MCF-7 | 84315 | 6132/31605 | 0.04/0.26 | -38.8 | -38.4 | -13.15 | FALSE |
| SRX080416 | TFs and others | CTCF | Breast | HMEC | 53716 | 8746/31605 | 0.08/0.26 | -38.7 | -38.3 | -13.14 | FALSE |
| SRX25289352 | TFs and others | GRHL2 | Breast | MCF-7 | 8716 | 1759/31605 | -0.15/0.26 | -38.7 | -38.3 | -13.13 | FALSE |
| SRX24602130 | TFs and others | VGLL1 | Breast | BT-20 | 42344 | 4866/31605 | 0.01/0.26 | -38.6 | -38.2 | -13.12 | FALSE |
| SRX6712594 | TFs and others | MED26 | Breast | MCF-7 | 1622 | 89/31605 | -1.53/0.26 | -38.6 | -38.2 | -13.12 | FALSE |
| SRX1012703 | TFs and others | ESR1 | Breast | T-47D | 1981 | 174/31605 | -1.02/0.26 | -38.5 | -38.1 | -13.10 | FALSE |
| SRX734338 | TFs and others | CHD8 | Breast | T-47D | 2206 | 1342/31605 | -0.20/0.26 | -38.5 | -38.1 | -13.09 | FALSE |
| SRX5794309 | TFs and others | TCF7 | Breast | Breast organoids | 12830 | 5058/31605 | 0.02/0.26 | -38.4 | -38.0 | -13.09 | FALSE |
| SRX15039970 | TFs and others | GATA3 | Breast | MDA-MB-231 | 42023 | 4755/31605 | 0.01/0.26 | -38.4 | -38.0 | -13.08 | FALSE |
| SRX20830460 | TFs and others | CTCF | Breast | T-47D | 49788 | 7832/31605 | 0.07/0.26 | -38.4 | -38.0 | -13.08 | FALSE |
| SRX2424665 | TFs and others | ESRRA | Breast | MCF-7 | 4604 | 896/31605 | -0.30/0.26 | -38.4 | -38.0 | -13.08 | FALSE |
| SRX10971495 | TFs and others | ESR1 | Breast | MCF-7 | 1373 | 337/31605 | -0.66/0.26 | -38.3 | -38.0 | -13.08 | FALSE |
| SRX176864 | TFs and others | GATA3 | Breast | MCF-7 | 55100 | 3271/31605 | -0.04/0.26 | -38.3 | -37.9 | -13.06 | FALSE |
| SRX5089546 | TFs and others | ARID2 | Breast | MCF-7 | 2947 | 202/31605 | -0.92/0.26 | -38.2 | -37.8 | -13.05 | FALSE |
| SRX3800712 | TFs and others | CTCF | Breast | T-47D | 35849 | 6407/31605 | 0.05/0.26 | -38.2 | -37.8 | -13.05 | FALSE |
| SRX10119387 | TFs and others | YAP1 | Breast | MDA-MB-231 | 31392 | 4547/31605 | 0.01/0.26 | -38.2 | -37.8 | -13.05 | FALSE |
| SRX4611702 | TFs and others | ESR1 | Breast | MCF-7 | 59173 | 8619/31605 | 0.08/0.26 | -38.2 | -37.8 | -13.04 | FALSE |
| SRX4374907 | TFs and others | CTCF | Breast | MB 157 | 40409 | 8422/31605 | 0.07/0.26 | -38.2 | -37.8 | -13.04 | FALSE |
| SRX13991447 | TFs and others | LEF1 | Breast | MCF 10A | 1792 | 1632/31605 | -0.16/0.26 | -38.1 | -37.8 | -13.04 | FALSE |
| SRX5323824 | TFs and others | FOXA1 | Breast | T-47D | 22940 | 2726/31605 | -0.06/0.26 | -38.1 | -37.7 | -13.03 | FALSE |
| SRX1660876 | TFs and others | TRIM25 | Breast | Breast cancer cells | 8645 | 3207/31605 | -0.04/0.26 | -38.0 | -37.7 | -13.02 | FALSE |
| SRX3322787 | TFs and others | RFX5 | Breast | MCF-7 | 5397 | 1926/31605 | -0.12/0.26 | -38.0 | -37.7 | -13.02 | FALSE |
| SRX7700193 | TFs and others | TP63 | Breast | MCF10DCIS.com | 22192 | 4116/31605 | -0.00/0.26 | -38.0 | -37.6 | -13.01 | FALSE |
| SRX1660872 | TFs and others | TRIM25 | Breast | Breast cancer cells | 32066 | 12095/31605 | 0.10/0.26 | -37.9 | -37.5 | -13.00 | FALSE |
| SRX3546802 | TFs and others | Epitope tags | Breast | MDA-MB-231 | 1644 | 291/31605 | -0.72/0.26 | -37.9 | -37.5 | -12.99 | FALSE |
| SRX5824596 | TFs and others | BRD4 | Breast | SUM 159PT | 5518 | 1665/31605 | -0.15/0.26 | -37.7 | -37.4 | -12.97 | FALSE |
| SRX4377562 | TFs and others | BRD9 | Breast | MDA-MB-231 | 9578 | 2997/31605 | -0.05/0.26 | -37.7 | -37.4 | -12.97 | FALSE |
| SRX5493760 | TFs and others | FOXA1 | Breast | Breast cancer cells | 13586 | 1924/31605 | -0.12/0.26 | -37.7 | -37.3 | -12.96 | FALSE |
| SRX4611728 | TFs and others | ESR1 | Breast | MCF-7 | 58727 | 8498/31605 | 0.08/0.26 | -37.7 | -37.3 | -12.96 | FALSE |
| SRX3447352 | TFs and others | ESR1 | Breast | MCF-7 | 67584 | 9169/31605 | 0.08/0.26 | -37.6 | -37.3 | -12.95 | FALSE |
| SRX25289338 | TFs and others | GRHL2 | Breast | MCF-7 | 6492 | 1560/31605 | -0.16/0.26 | -37.6 | -37.2 | -12.95 | FALSE |
| SRX5702619 | TFs and others | AR | Breast | T-47D | 26495 | 1920/31605 | -0.12/0.26 | -37.6 | -37.2 | -12.95 | FALSE |
| SRX19662636 | TFs and others | RARA | Breast | T-47D | 1114 | 887/31605 | -0.30/0.26 | -37.6 | -37.2 | -12.94 | FALSE |
| SRX2630262 | TFs and others | ZBTB11 | Breast | MCF-7 | 1594 | 977/31605 | -0.28/0.26 | -37.6 | -37.2 | -12.94 | FALSE |
| SRX15826238 | TFs and others | KMT2D | Breast | MCF-7 | 9168 | 1130/31605 | -0.24/0.26 | -37.5 | -37.1 | -12.93 | FALSE |
| SRX5089516 | TFs and others | SMARCA4 | Breast | MCF-7 | 11343 | 1755/31605 | -0.14/0.26 | -37.5 | -37.1 | -12.92 | FALSE |
| SRX18191647 | TFs and others | ESR1 | Breast | MCF-7 | 1764 | 197/31605 | -0.92/0.26 | -37.4 | -37.0 | -12.91 | FALSE |
| SRX20830462 | TFs and others | CTCF | Breast | T-47D | 47555 | 7196/31605 | 0.06/0.26 | -37.3 | -37.0 | -12.90 | FALSE |
| SRX5089543 | TFs and others | ARID2 | Breast | MCF-7 | 2130 | 147/31605 | -1.11/0.26 | -37.3 | -36.9 | -12.89 | FALSE |
| SRX6860647 | TFs and others | ESR1 | Breast | T-47D | 1615 | 61/31605 | -1.86/0.26 | -37.3 | -36.9 | -12.89 | FALSE |
| SRX20830458 | TFs and others | CTCF | Breast | T-47D | 46402 | 7425/31605 | 0.06/0.26 | -37.2 | -36.8 | -12.88 | FALSE |
| SRX13340677 | TFs and others | Epitope tags | Breast | T-47D | 4784 | 680/31605 | -0.38/0.26 | -37.2 | -36.8 | -12.87 | FALSE |
| SRX20830497 | TFs and others | CTCF | Breast | T-47D | 52442 | 8004/31605 | 0.07/0.26 | -37.2 | -36.8 | -12.87 | FALSE |
| SRX2944448 | TFs and others | MYC | Breast | MCF 10A | 2151 | 1351/31605 | -0.19/0.26 | -37.1 | -36.8 | -12.86 | FALSE |
| SRX15039967 | TFs and others | GATA3 | Breast | MDA-MB-231 | 73304 | 6701/31605 | 0.05/0.26 | -37.1 | -36.8 | -12.86 | FALSE |
| SRX7644315 | TFs and others | EP300 | Breast | MCF-7 | 1042 | 59/31605 | -1.89/0.26 | -37.1 | -36.7 | -12.86 | FALSE |
| SRX5323826 | TFs and others | FOXA1 | Breast | T-47D | 23141 | 2829/31605 | -0.05/0.26 | -37.1 | -36.7 | -12.85 | FALSE |
| SRX10365516 | TFs and others | RAC1 | Breast | MCF-7 | 4034 | 243/31605 | -0.80/0.26 | -37.0 | -36.7 | -12.85 | FALSE |
| SRX233463 | TFs and others | TFAP2A | Breast | MCF-7 | 13670 | 2112/31605 | -0.10/0.26 | -37.0 | -36.6 | -12.84 | FALSE |
| SRX15039969 | TFs and others | GATA3 | Breast | MDA-MB-231 | 53421 | 5636/31605 | 0.04/0.26 | -37.0 | -36.6 | -12.83 | FALSE |
| SRX2897199 | TFs and others | ESR1 | Breast | Breast cancer cells | 1498 | 578/31605 | -0.43/0.26 | -36.9 | -36.6 | -12.83 | FALSE |
| SRX795225 | TFs and others | BRD4 | Breast | SUM 159PT | 5456 | 1632/31605 | -0.15/0.26 | -36.9 | -36.6 | -12.83 | FALSE |
| SRX24428383 | TFs and others | Epitope tags | Breast | T-47D | 3380 | 2034/31605 | -0.11/0.26 | -36.9 | -36.5 | -12.82 | FALSE |
| SRX13991445 | TFs and others | LEF1 | Breast | MCF 10A | 1716 | 1593/31605 | -0.16/0.26 | -36.9 | -36.5 | -12.82 | FALSE |
| SRX1161182 | TFs and others | NR3C1 | Breast | ZR-75-1 | 23542 | 1658/31605 | -0.15/0.26 | -36.8 | -36.4 | -12.81 | FALSE |
| SRX15826235 | TFs and others | KMT2D | Breast | MCF-7 | 10156 | 1245/31605 | -0.21/0.26 | -36.8 | -36.4 | -12.80 | FALSE |
| SRX11222438 | TFs and others | FOXA1 | Breast | MCF-7 | 33678 | 3327/31605 | -0.03/0.26 | -36.7 | -36.4 | -12.79 | FALSE |
| SRX8949279 | TFs and others | NCOA3 | Breast | ZR-75-1 | 65899 | 5783/31605 | 0.04/0.26 | -36.7 | -36.3 | -12.79 | FALSE |
| SRX5203303 | TFs and others | FOXA1 | Breast | MCF-7 | 27821 | 1812/31605 | -0.13/0.26 | -36.7 | -36.3 | -12.79 | FALSE |
| SRX10773098 | TFs and others | CTCF | Breast | MB 157 | 35673 | 6508/31605 | 0.05/0.26 | -36.7 | -36.3 | -12.78 | FALSE |
| SRX1787654 | TFs and others | FOXA1 | Breast | MCF-7 | 26496 | 2356/31605 | -0.08/0.26 | -36.7 | -36.3 | -12.78 | FALSE |
| SRX4023330 | TFs and others | CDK9 | Breast | T-47D | 3588 | 1597/31605 | -0.15/0.26 | -36.6 | -36.2 | -12.76 | FALSE |
| SRX1787677 | TFs and others | KMT2C | Breast | MCF-7 | 10836 | 1081/31605 | -0.24/0.26 | -36.6 | -36.2 | -12.76 | FALSE |
| SRX3110734 | TFs and others | ESR1 | Breast | MCF-7 | 63106 | 8872/31605 | 0.08/0.26 | -36.5 | -36.1 | -12.75 | FALSE |
| SRX6896411 | TFs and others | RNF2 | Breast | T-47D | 2016 | 116/31605 | -1.26/0.26 | -36.5 | -36.1 | -12.74 | FALSE |
| SRX8537736 | TFs and others | FOXA1 | Breast | MCF-7 | 26027 | 2457/31605 | -0.07/0.26 | -36.4 | -36.0 | -12.73 | FALSE |
| SRX7098802 | TFs and others | CTCF | Breast | Mammary epithelial cells | 40422 | 7884/31605 | 0.07/0.26 | -36.4 | -36.0 | -12.73 | FALSE |
| SRX3110741 | TFs and others | ESR1 | Breast | MCF-7 | 54543 | 7467/31605 | 0.07/0.26 | -36.3 | -36.0 | -12.72 | FALSE |
| SRX20830454 | TFs and others | CTCF | Breast | T-47D | 46574 | 7793/31605 | 0.07/0.26 | -36.3 | -35.9 | -12.71 | FALSE |
| SRX747788 | TFs and others | RELA | Breast | MCF-7 | 1058 | 681/31605 | -0.37/0.26 | -36.2 | -35.8 | -12.69 | FALSE |
| SRX3447356 | TFs and others | ESR1 | Breast | MCF-7 | 68384 | 9071/31605 | 0.08/0.26 | -36.2 | -35.8 | -12.69 | FALSE |
| SRX3447354 | TFs and others | ESR1 | Breast | MCF-7 | 64901 | 8652/31605 | 0.08/0.26 | -36.2 | -35.8 | -12.69 | FALSE |
| SRX4564977 | TFs and others | CTCF | Breast | MCF-7 | 33709 | 6049/31605 | 0.05/0.26 | -36.2 | -35.8 | -12.69 | FALSE |
| SRX3583204 | TFs and others | CTCF | Breast | MCF-7 | 52779 | 8372/31605 | 0.08/0.26 | -36.1 | -35.7 | -12.68 | FALSE |
| SRX1594207 | TFs and others | CTCF | Breast | MCF-7 | 43852 | 7934/31605 | 0.07/0.26 | -36.1 | -35.7 | -12.67 | FALSE |
| SRX19662630 | TFs and others | MAX | Breast | T-47D | 2012 | 1178/31605 | -0.22/0.26 | -36.0 | -35.7 | -12.67 | FALSE |
| SRX15039968 | TFs and others | GATA3 | Breast | MDA-MB-231 | 49883 | 5319/31605 | 0.03/0.26 | -36.0 | -35.7 | -12.67 | FALSE |
| SRX3931031 | TFs and others | FOXA1 | Breast | MCF-7 | 31234 | 3227/31605 | -0.03/0.26 | -36.0 | -35.7 | -12.66 | FALSE |
| SRX20830491 | TFs and others | CTCF | Breast | T-47D | 50778 | 8027/31605 | 0.07/0.26 | -36.0 | -35.6 | -12.66 | FALSE |
| SRX103008 | TFs and others | CTCF | Breast | MCF-7 | 84533 | 9365/31605 | 0.09/0.26 | -35.9 | -35.6 | -12.65 | FALSE |
| SRX190347 | TFs and others | REST | Breast | MCF-7 | 14347 | 3533/31605 | -0.02/0.26 | -35.9 | -35.6 | -12.65 | FALSE |
| SRX1012686 | TFs and others | ESR1 | Breast | MCF-7 | 1437 | 94/31605 | -1.41/0.26 | -35.9 | -35.5 | -12.64 | FALSE |
| SRX6896408 | TFs and others | GRHL2 | Breast | T-47D | 14607 | 2445/31605 | -0.07/0.26 | -35.8 | -35.5 | -12.63 | FALSE |
| SRX1531794 | TFs and others | MED1 | Breast | MCF-7 | 2245 | 334/31605 | -0.63/0.26 | -35.8 | -35.4 | -12.62 | FALSE |
| SRX361224 | TFs and others | GATA3 | Breast | T-47D | 12037 | 1608/31605 | -0.15/0.26 | -35.8 | -35.4 | -12.62 | FALSE |
| SRX4374897 | TFs and others | NOTCH1 | Breast | MB 157 | 2526 | 1145/31605 | -0.22/0.26 | -35.8 | -35.4 | -12.61 | FALSE |
| SRX3800709 | TFs and others | CTCF | Breast | T-47D | 52242 | 8452/31605 | 0.08/0.26 | -35.8 | -35.4 | -12.61 | FALSE |
| SRX1447317 | TFs and others | FOXA1 | Breast | MCF-7 | 49020 | 4420/31605 | 0.01/0.26 | -35.7 | -35.4 | -12.61 | FALSE |
| SRX5287687 | TFs and others | TEAD4 | Breast | MCF-7 | 5811 | 1176/31605 | -0.22/0.26 | -35.7 | -35.4 | -12.61 | FALSE |
| SRX11149853 | TFs and others | GATA3 | Breast | MFM-223 | 64762 | 4857/31605 | 0.02/0.26 | -35.7 | -35.4 | -12.61 | FALSE |
| SRX4786537 | TFs and others | ESR1 | Breast | MCF-7 | 1226 | 58/31605 | -1.86/0.26 | -35.7 | -35.3 | -12.60 | FALSE |
| SRX10164596 | TFs and others | ESR1 | Breast | MCF-7 | 2802 | 90/31605 | -1.45/0.26 | -35.7 | -35.3 | -12.60 | FALSE |
| SRX10119369 | TFs and others | WWTR1 | Breast | MCF 10A | 19451 | 3249/31605 | -0.03/0.26 | -35.7 | -35.3 | -12.60 | FALSE |
| SRX3447351 | TFs and others | ESR1 | Breast | MCF-7 | 63663 | 8658/31605 | 0.08/0.26 | -35.6 | -35.2 | -12.58 | FALSE |
| SRX1787655 | TFs and others | FOXA1 | Breast | MCF-7 | 34417 | 2635/31605 | -0.06/0.26 | -35.6 | -35.2 | -12.58 | FALSE |
| SRX667407 | TFs and others | Epitope tags | Breast | MCF-7 | 6025 | 3364/31605 | -0.02/0.26 | -35.5 | -35.2 | -12.57 | FALSE |
| SRX15391841 | TFs and others | FOXA1 | Breast | T-47D | 20717 | 2544/31605 | -0.06/0.26 | -35.5 | -35.2 | -12.57 | FALSE |
| SRX2020841 | TFs and others | STAT3 | Breast | MDA-MB-231 | 12994 | 2458/31605 | -0.07/0.26 | -35.5 | -35.2 | -12.57 | FALSE |
| SRX10773099 | TFs and others | CTCF | Breast | MB 157 | 34539 | 6304/31605 | 0.05/0.26 | -35.5 | -35.1 | -12.57 | FALSE |
| SRX6058757 | TFs and others | CEBPA | Breast | T-47D | 17567 | 2919/31605 | -0.04/0.26 | -35.5 | -35.1 | -12.56 | FALSE |
| SRX8850951 | TFs and others | Epitope tags | Breast | MCF 10A | 4289 | 1610/31605 | -0.15/0.26 | -35.4 | -35.1 | -12.56 | FALSE |
| SRX10971499 | TFs and others | ESR1 | Breast | MCF-7 | 1191 | 234/31605 | -0.80/0.26 | -35.4 | -35.0 | -12.55 | FALSE |
| SRX3800708 | TFs and others | CTCF | Breast | T-47D | 34745 | 6506/31605 | 0.06/0.26 | -35.4 | -35.0 | -12.55 | FALSE |
| SRX4374908 | TFs and others | CTCF | Breast | MB 157 | 34301 | 6559/31605 | 0.06/0.26 | -35.4 | -35.0 | -12.54 | FALSE |
| SRX4611707 | TFs and others | ESR1 | Breast | MCF-7 | 33148 | 6509/31605 | 0.06/0.26 | -35.3 | -35.0 | -12.54 | FALSE |
| SRX5089553 | TFs and others | BRD4 | Breast | MCF-7 | 2084 | 1139/31605 | -0.22/0.26 | -35.3 | -35.0 | -12.54 | FALSE |
| SRX9419946 | TFs and others | MED1 | Breast | BT-474 | 22617 | 3770/31605 | -0.01/0.26 | -35.3 | -35.0 | -12.54 | FALSE |
| SRX5401864 | TFs and others | PGR | Breast | T-47D | 27129 | 3229/31605 | -0.03/0.26 | -35.3 | -34.9 | -12.53 | FALSE |
| SRX5089393 | TFs and others | ARID1A | Breast | MCF-7 | 6297 | 535/31605 | -0.44/0.26 | -35.3 | -34.9 | -12.53 | FALSE |
| SRX19662634 | TFs and others | RARA | Breast | T-47D | 1124 | 934/31605 | -0.27/0.26 | -35.3 | -34.9 | -12.52 | FALSE |
| SRX10365557 | TFs and others | ESR1 | Breast | MCF-7 | 7202 | 370/31605 | -0.58/0.26 | -35.2 | -34.9 | -12.52 | FALSE |
| SRX278610 | TFs and others | MED12 | Breast | MCF-7 | 791 | 36/31605 | -2.42/0.26 | -35.2 | -34.9 | -12.52 | FALSE |
| SRX6853596 | TFs and others | ARNT | Breast | MCF-7 | 2389 | 1077/31605 | -0.23/0.26 | -35.2 | -34.9 | -12.52 | FALSE |
| SRX747783 | TFs and others | RELA | Breast | MCF-7 | 920 | 587/31605 | -0.41/0.26 | -35.2 | -34.8 | -12.51 | FALSE |
| SRX5057632 | TFs and others | GATA3 | Breast | MCF-7 | 44410 | 2558/31605 | -0.06/0.26 | -35.2 | -34.8 | -12.51 | FALSE |
| SRX3923514 | TFs and others | FOXA1 | Breast | MCF-7 | 54789 | 4821/31605 | 0.02/0.26 | -35.2 | -34.8 | -12.51 | FALSE |
| SRX2020846 | TFs and others | STAT3 | Breast | MDA-MB-468 | 11075 | 2184/31605 | -0.09/0.26 | -35.1 | -34.8 | -12.50 | FALSE |
| SRX1787684 | TFs and others | KMT2C | Breast | MCF-7 | 10238 | 1028/31605 | -0.24/0.26 | -35.1 | -34.7 | -12.50 | FALSE |
| SRX1787683 | TFs and others | KMT2C | Breast | MCF-7 | 10144 | 1013/31605 | -0.25/0.26 | -35.1 | -34.7 | -12.49 | FALSE |
| SRX11149852 | TFs and others | GATA3 | Breast | MFM-223 | 60403 | 4489/31605 | 0.02/0.26 | -35.1 | -34.7 | -12.49 | FALSE |
| SRX3401284 | TFs and others | TRPS1 | Breast | T-47D | 7175 | 836/31605 | -0.30/0.26 | -35.0 | -34.7 | -12.48 | FALSE |
| SRX4792905 | TFs and others | CTCF | Breast | T-47D | 36232 | 9370/31605 | 0.09/0.26 | -35.0 | -34.6 | -12.48 | FALSE |
| SRX5457486 | TFs and others | E2F8 | Breast | MCF-7 | 1343 | 420/31605 | -0.52/0.26 | -35.0 | -34.6 | -12.47 | FALSE |
| SRX19888707 | TFs and others | NELFE | Breast | MCF-7 | 3926 | 2090/31605 | -0.09/0.26 | -34.9 | -34.6 | -12.47 | FALSE |
| SRX176884 | TFs and others | EP300 | Breast | MCF-7 | 19393 | 1635/31605 | -0.14/0.26 | -34.9 | -34.6 | -12.46 | FALSE |
| SRX20830496 | TFs and others | CTCF | Breast | T-47D | 46788 | 7162/31605 | 0.07/0.26 | -34.9 | -34.6 | -12.46 | FALSE |
| SRX5794310 | TFs and others | TCF7 | Breast | Breast organoids | 5047 | 2232/31605 | -0.08/0.26 | -34.9 | -34.6 | -12.46 | FALSE |
| SRX150524 | TFs and others | GATA3 | Breast | MCF-7 | 11421 | 981/31605 | -0.25/0.26 | -34.9 | -34.5 | -12.46 | FALSE |
| SRX3322395 | TFs and others | RAD51 | Breast | MCF-7 | 3742 | 1491/31605 | -0.16/0.26 | -34.9 | -34.5 | -12.46 | FALSE |
| SRX3800707 | TFs and others | CTCF | Breast | T-47D | 36066 | 6353/31605 | 0.05/0.26 | -34.9 | -34.5 | -12.46 | FALSE |
| SRX20830457 | TFs and others | CTCF | Breast | T-47D | 52007 | 7823/31605 | 0.07/0.26 | -34.9 | -34.5 | -12.46 | FALSE |
| SRX5203307 | TFs and others | FOXA1 | Breast | MCF-7 | 24904 | 1780/31605 | -0.12/0.26 | -34.9 | -34.5 | -12.45 | FALSE |
| SRX9419938 | TFs and others | BRD4 | Breast | BT-474 | 22402 | 3757/31605 | -0.00/0.26 | -34.8 | -34.4 | -12.44 | FALSE |
| SRX1660873 | TFs and others | TRIM25 | Breast | Breast cancer cells | 36605 | 12477/31605 | 0.11/0.26 | -34.8 | -34.4 | -12.43 | FALSE |
| SRX20830490 | TFs and others | CTCF | Breast | T-47D | 48587 | 7740/31605 | 0.07/0.26 | -34.7 | -34.3 | -12.42 | FALSE |
| SRX099864 | TFs and others | CTCF | Breast | MCF-7 | 57163 | 8876/31605 | 0.09/0.26 | -34.6 | -34.3 | -12.41 | FALSE |
| SRX8520805 | TFs and others | NR3C1 | Breast | MCF-7 | 13886 | 887/31605 | -0.28/0.26 | -34.6 | -34.3 | -12.41 | FALSE |
| SRX1787651 | TFs and others | FOXA1 | Breast | MCF-7 | 32296 | 2600/31605 | -0.06/0.26 | -34.6 | -34.3 | -12.41 | FALSE |
| SRX10119378 | TFs and others | WWTR1 | Breast | MCF 10A | 49035 | 5415/31605 | 0.04/0.26 | -34.6 | -34.3 | -12.41 | FALSE |
| SRX4792902 | TFs and others | CTCF | Breast | T-47D | 33126 | 8295/31605 | 0.08/0.26 | -34.5 | -34.2 | -12.39 | FALSE |
| SRX3447357 | TFs and others | ESR1 | Breast | MCF-7 | 65874 | 8778/31605 | 0.09/0.26 | -34.5 | -34.2 | -12.39 | FALSE |
| SRX20830459 | TFs and others | CTCF | Breast | T-47D | 46893 | 7133/31605 | 0.07/0.26 | -34.5 | -34.2 | -12.39 | FALSE |
| SRX150478 | TFs and others | FOS | Breast | MCF 10A | 110387 | 8163/31605 | 0.08/0.26 | -34.5 | -34.2 | -12.39 | FALSE |
| SRX5323823 | TFs and others | FOXA1 | Breast | T-47D | 17345 | 2173/31605 | -0.09/0.26 | -34.5 | -34.2 | -12.39 | FALSE |
| SRX3401279 | TFs and others | CTBP2 | Breast | MCF-7 | 1688 | 129/31605 | -1.14/0.26 | -34.5 | -34.1 | -12.38 | FALSE |
| SRX9419947 | TFs and others | MED1 | Breast | BT-474 | 21568 | 3669/31605 | -0.01/0.26 | -34.5 | -34.1 | -12.38 | FALSE |
| SRX747789 | TFs and others | RELA | Breast | MCF-7 | 1101 | 567/31605 | -0.41/0.26 | -34.5 | -34.1 | -12.38 | FALSE |
| SRX190247 | TFs and others | RAD21 | Breast | MCF-7 | 57445 | 6692/31605 | 0.06/0.26 | -34.4 | -34.1 | -12.37 | FALSE |
| SRX5089417 | TFs and others | SMARCA4 | Breast | MCF-7 | 15641 | 3138/31605 | -0.03/0.26 | -34.4 | -34.1 | -12.37 | FALSE |
| SRX5172062 | TFs and others | SMARCA4 | Breast | MCF-7 | 15320 | 2056/31605 | -0.09/0.26 | -34.4 | -34.1 | -12.37 | FALSE |
| SRX14101905 | TFs and others | KAT2B | Breast | MCF-7 | 4027 | 1642/31605 | -0.14/0.26 | -34.4 | -34.1 | -12.37 | FALSE |
| SRX190174 | TFs and others | JUND | Breast | T-47D | 10650 | 1549/31605 | -0.15/0.26 | -34.2 | -33.8 | -12.33 | FALSE |
| SRX5089444 | TFs and others | SMARCB1 | Breast | MCF-7 | 16355 | 2184/31605 | -0.08/0.26 | -34.2 | -33.8 | -12.32 | FALSE |
| SRX3800706 | TFs and others | CTCF | Breast | T-47D | 57374 | 8944/31605 | 0.09/0.26 | -34.2 | -33.8 | -12.32 | FALSE |
| SRX2897198 | TFs and others | ESR1 | Breast | Breast cancer cells | 1842 | 1049/31605 | -0.23/0.26 | -34.1 | -33.8 | -12.32 | FALSE |
| SRX20830455 | TFs and others | CTCF | Breast | T-47D | 47261 | 7710/31605 | 0.08/0.26 | -34.1 | -33.7 | -12.31 | FALSE |
| SRX10773101 | TFs and others | CTCF | Breast | MB 157 | 29277 | 5661/31605 | 0.05/0.26 | -34.1 | -33.7 | -12.30 | FALSE |
| SRX3554411 | TFs and others | CREB1 | Breast | MDA-MB-134-VI | 2288 | 734/31605 | -0.33/0.26 | -34.1 | -33.7 | -12.30 | FALSE |
| SRX5323869 | TFs and others | STAT3 | Breast | MCF-7 | 1759 | 775/31605 | -0.31/0.26 | -34.0 | -33.7 | -12.30 | FALSE |
| SRX4547413 | TFs and others | CTCF | Breast | MCF-7 | 21732 | 5587/31605 | 0.04/0.26 | -34.0 | -33.7 | -12.29 | FALSE |
| SRX4611719 | TFs and others | ESR1 | Breast | MCF-7 | 48079 | 5949/31605 | 0.05/0.26 | -34.0 | -33.6 | -12.29 | FALSE |
| SRX4792909 | TFs and others | RAD21 | Breast | T-47D | 10978 | 4308/31605 | 0.02/0.26 | -34.0 | -33.6 | -12.29 | FALSE |
| SRX2871342 | TFs and others | GATA3 | Breast | T-47D | 12745 | 1746/31605 | -0.12/0.26 | -34.0 | -33.6 | -12.28 | FALSE |
| SRX747786 | TFs and others | RELA | Breast | MCF-7 | 1045 | 697/31605 | -0.34/0.26 | -33.9 | -33.6 | -12.28 | FALSE |
| SRX395617 | TFs and others | CTCF | Breast | T-47D | 43686 | 6664/31605 | 0.06/0.26 | -33.9 | -33.5 | -12.27 | FALSE |
| SRX361226 | TFs and others | GATA3 | Breast | MCF-7 | 19042 | 2000/31605 | -0.10/0.26 | -33.8 | -33.5 | -12.26 | FALSE |
| SRX2636200 | TFs and others | RAD21 | Breast | MCF-7 | 52170 | 6051/31605 | 0.05/0.26 | -33.7 | -33.4 | -12.24 | FALSE |
| SRX1594206 | TFs and others | CTCF | Breast | MCF-7 | 38489 | 7471/31605 | 0.07/0.26 | -33.7 | -33.3 | -12.23 | FALSE |
| SRX10365542 | TFs and others | RAC1 | Breast | MCF-7 | 13545 | 1238/31605 | -0.19/0.26 | -33.7 | -33.3 | -12.23 | FALSE |
| SRX3285589 | TFs and others | KDM5B | Breast | C70R | 10538 | 4721/31605 | 0.03/0.26 | -33.6 | -33.3 | -12.22 | FALSE |
| SRX673715 | TFs and others | Biotin | Breast | MCF-7 | 2415 | 613/31605 | -0.38/0.26 | -33.6 | -33.2 | -12.21 | FALSE |
| SRX3110742 | TFs and others | ESR1 | Breast | MCF-7 | 50219 | 6926/31605 | 0.07/0.26 | -33.5 | -33.2 | -12.20 | FALSE |
| SRX4792907 | TFs and others | RAD21 | Breast | T-47D | 20464 | 5675/31605 | 0.05/0.26 | -33.5 | -33.2 | -12.20 | FALSE |
| SRX9419945 | TFs and others | MED1 | Breast | BT-474 | 21207 | 3568/31605 | -0.01/0.26 | -33.5 | -33.2 | -12.20 | FALSE |
| SRX1709984 | TFs and others | ESR1 | Breast | T-47D | 1045 | 371/31605 | -0.56/0.26 | -33.5 | -33.1 | -12.19 | FALSE |
| SRX4374900 | TFs and others | NOTCH1 | Breast | MB 157 | 1821 | 970/31605 | -0.25/0.26 | -33.4 | -33.0 | -12.18 | FALSE |
| SRX3229162 | TFs and others | ESR1 | Breast | Breast cancer | 1140 | 74/31605 | -1.56/0.26 | -33.4 | -33.0 | -12.18 | FALSE |
| SRX4213899 | TFs and others | YAP1 | Breast | MCF 10A | 10531 | 2407/31605 | -0.06/0.26 | -33.4 | -33.0 | -12.17 | FALSE |
| SRX176863 | TFs and others | FOXA1 | Breast | MCF-7 | 80280 | 5879/31605 | 0.05/0.26 | -33.3 | -33.0 | -12.16 | FALSE |
| SRX10119370 | TFs and others | WWTR1 | Breast | MCF 10A | 30911 | 4199/31605 | 0.01/0.26 | -33.3 | -32.9 | -12.16 | FALSE |
| SRX11120894 | TFs and others | EP300 | Breast | MCF-7 | 8430 | 1358/31605 | -0.17/0.26 | -33.3 | -32.9 | -12.15 | FALSE |
| SRX2900154 | TFs and others | SUZ12 | Breast | CAL-51 | 1438 | 1000/31605 | -0.24/0.26 | -33.2 | -32.8 | -12.14 | FALSE |
| SRX18191661 | TFs and others | ESR1 | Breast | MCF-7 | 1033 | 53/31605 | -1.88/0.26 | -33.2 | -32.8 | -12.13 | FALSE |
| SRX15431001 | TFs and others | TFE3 | Breast | T-47D | 11573 | 1533/31605 | -0.14/0.26 | -33.2 | -32.8 | -12.13 | FALSE |
| SRX5089427 | TFs and others | SMARCB1 | Breast | MCF-7 | 9662 | 1391/31605 | -0.16/0.26 | -33.2 | -32.8 | -12.13 | FALSE |
| SRX2871347 | TFs and others | FOXA1 | Breast | T-47D | 16812 | 2092/31605 | -0.08/0.26 | -33.1 | -32.8 | -12.13 | FALSE |
| SRX8520795 | TFs and others | STAT3 | Breast | HCC70 | 13723 | 2606/31605 | -0.05/0.26 | -33.1 | -32.7 | -12.12 | FALSE |
| SRX10365524 | TFs and others | ESR1 | Breast | MDA-MB-231 | 5063 | 466/31605 | -0.46/0.26 | -33.0 | -32.6 | -12.10 | FALSE |
| SRX1116522 | TFs and others | ESR1 | Breast | MCF-7 | 715 | 428/31605 | -0.49/0.26 | -32.9 | -32.6 | -12.09 | FALSE |
| SRX8537737 | TFs and others | FOXA1 | Breast | MCF-7 | 27374 | 2545/31605 | -0.05/0.26 | -32.9 | -32.5 | -12.08 | FALSE |
| SRX20830494 | TFs and others | CTCF | Breast | T-47D | 43872 | 6787/31605 | 0.07/0.26 | -32.9 | -32.5 | -12.08 | FALSE |
| SRX1787676 | TFs and others | KMT2C | Breast | MCF-7 | 13858 | 1470/31605 | -0.15/0.26 | -32.8 | -32.5 | -12.07 | FALSE |
| SRX3322545 | TFs and others | NCOA3 | Breast | MCF-7 | 1825 | 73/31605 | -1.55/0.26 | -32.8 | -32.4 | -12.06 | FALSE |
| SRX13300074 | TFs and others | CTCF | Breast | MCF 10A | 37586 | 5988/31605 | 0.06/0.26 | -32.7 | -32.4 | -12.05 | FALSE |
| SRX4547416 | TFs and others | CTCF | Breast | FULVR | 9041 | 2912/31605 | -0.03/0.26 | -32.7 | -32.3 | -12.05 | FALSE |
| SRX490435 | TFs and others | ESR1 | Breast | MCF-7 | 361 | 38/31605 | -2.25/0.26 | -32.7 | -32.3 | -12.04 | FALSE |
| SRX2194250 | TFs and others | CEBPB | Breast | SUM 159PT | 72512 | 6833/31605 | 0.07/0.26 | -32.7 | -32.3 | -12.04 | FALSE |
| SRX5683084 | TFs and others | ESR1 | Breast | T-47D | 662 | 48/31605 | -1.97/0.26 | -32.6 | -32.3 | -12.04 | FALSE |
| SRX2636442 | TFs and others | ZHX2 | Breast | MCF-7 | 1738 | 997/31605 | -0.23/0.26 | -32.6 | -32.3 | -12.03 | FALSE |
| SRX20830450 | TFs and others | Epitope tags | Breast | T-47D | 5072 | 477/31605 | -0.45/0.26 | -32.6 | -32.3 | -12.03 | FALSE |
| SRX055311 | TFs and others | KDM5B | Breast | MCF-7 | 1330 | 1079/31605 | -0.21/0.26 | -32.6 | -32.2 | -12.03 | FALSE |
| SRX1165109 | TFs and others | Epitope tags | Breast | MCF-7 | 52136 | 6036/31605 | 0.06/0.26 | -32.6 | -32.2 | -12.03 | FALSE |
| SRX5251418 | TFs and others | ESR1 | Breast | MCF-7 | 819 | 142/31605 | -1.04/0.26 | -32.5 | -32.2 | -12.02 | FALSE |
| SRX150476 | TFs and others | FOS | Breast | MCF 10A | 85712 | 7312/31605 | 0.08/0.26 | -32.5 | -32.1 | -12.00 | FALSE |
| SRX20830493 | TFs and others | CTCF | Breast | T-47D | 42546 | 6475/31605 | 0.06/0.26 | -32.5 | -32.1 | -12.00 | FALSE |
| SRX15391846 | TFs and others | ESR1 | Breast | T-47D | 1451 | 51/31605 | -1.90/0.26 | -32.5 | -32.1 | -12.00 | FALSE |
| SRX6828394 | TFs and others | CTCF | Breast | MCF-7 | 61582 | 7768/31605 | 0.08/0.26 | -32.4 | -32.1 | -12.00 | FALSE |
| SRX5089388 | TFs and others | ARID1A | Breast | MCF-7 | 24761 | 2294/31605 | -0.07/0.26 | -32.4 | -32.1 | -12.00 | FALSE |
| SRX2424664 | TFs and others | ESRRA | Breast | MCF-7 | 4274 | 689/31605 | -0.33/0.26 | -32.3 | -32.0 | -11.98 | FALSE |
| SRX1165108 | TFs and others | Epitope tags | Breast | MCF-7 | 55263 | 6410/31605 | 0.06/0.26 | -32.3 | -32.0 | -11.98 | FALSE |
| SRX1787682 | TFs and others | KMT2C | Breast | MCF-7 | 7640 | 764/31605 | -0.30/0.26 | -32.3 | -31.9 | -11.97 | FALSE |
| SRX11374765 | TFs and others | PGR | Breast | T-47D | 34892 | 3226/31605 | -0.01/0.26 | -32.2 | -31.9 | -11.96 | FALSE |
| SRX3110745 | TFs and others | ESR1 | Breast | MCF-7 | 51570 | 7719/31605 | 0.08/0.26 | -32.2 | -31.9 | -11.95 | FALSE |
| SRX1091826 | TFs and others | CTCF | Breast | MCF-7 | 68508 | 8735/31605 | 0.09/0.26 | -32.2 | -31.9 | -11.95 | FALSE |
| SRX5806610 | TFs and others | CTCF | Breast | MCF-7 | 63743 | 8345/31605 | 0.09/0.26 | -32.2 | -31.8 | -11.95 | FALSE |
| SRX13340685 | TFs and others | GATA3 | Breast | T-47D | 9836 | 1406/31605 | -0.15/0.26 | -32.2 | -31.8 | -11.95 | FALSE |
| SRX15430993 | TFs and others | FOXA1 | Breast | SK-BR-3 | 48759 | 4675/31605 | 0.03/0.26 | -32.1 | -31.8 | -11.94 | FALSE |
| SRX5323843 | TFs and others | FOXA1 | Breast | MCF-7 | 33710 | 2725/31605 | -0.04/0.26 | -32.1 | -31.8 | -11.93 | FALSE |
| SRX20830456 | TFs and others | CTCF | Breast | T-47D | 37316 | 5889/31605 | 0.06/0.26 | -32.1 | -31.8 | -11.93 | FALSE |
| SRX18191663 | TFs and others | ESR1 | Breast | MCF-7 | 1421 | 65/31605 | -1.64/0.26 | -32.0 | -31.7 | -11.92 | FALSE |
| SRX2020840 | TFs and others | STAT3 | Breast | MDA-MB-231 | 11894 | 2146/31605 | -0.07/0.26 | -32.0 | -31.7 | -11.92 | FALSE |
| SRX11374766 | TFs and others | PGR | Breast | T-47D | 42591 | 4094/31605 | 0.02/0.26 | -32.0 | -31.7 | -11.92 | FALSE |
| SRX3229134 | TFs and others | AR | Breast | MCF-7 | 1213 | 58/31605 | -1.75/0.26 | -32.0 | -31.7 | -11.92 | FALSE |
| SRX968976 | TFs and others | RELA | Breast | MCF-7 | 6969 | 2015/31605 | -0.08/0.26 | -32.0 | -31.7 | -11.91 | FALSE |
| SRX9419937 | TFs and others | BRD4 | Breast | BT-474 | 21215 | 3610/31605 | 0.00/0.26 | -32.0 | -31.6 | -11.91 | FALSE |
| SRX18013278 | TFs and others | VGLL1 | Breast | FULVR | 26387 | 3118/31605 | -0.02/0.26 | -31.9 | -31.5 | -11.89 | FALSE |
| SRX095407 | TFs and others | NCOA1 | Breast | LY2 | 3350 | 1447/31605 | -0.15/0.26 | -31.9 | -31.5 | -11.89 | FALSE |
| SRX9419944 | TFs and others | MED1 | Breast | BT-474 | 20888 | 3586/31605 | 0.00/0.26 | -31.9 | -31.5 | -11.89 | FALSE |
| SRX8520797 | TFs and others | STAT3 | Breast | MDA-MB-231 | 11908 | 2153/31605 | -0.07/0.26 | -31.8 | -31.5 | -11.88 | FALSE |
| SRX10773102 | TFs and others | CTCF | Breast | MB 157 | 26178 | 5060/31605 | 0.04/0.26 | -31.8 | -31.5 | -11.88 | FALSE |
| SRX3923533 | TFs and others | FOXA1 | Breast | ZR-75-1 | 61838 | 5392/31605 | 0.05/0.26 | -31.8 | -31.5 | -11.88 | FALSE |
| SRX3229160 | TFs and others | ESR1 | Breast | Breast cancer | 854 | 240/31605 | -0.73/0.26 | -31.8 | -31.5 | -11.87 | FALSE |
| SRX6058753 | TFs and others | PGR | Breast | T-47D | 13091 | 1978/31605 | -0.09/0.26 | -31.8 | -31.4 | -11.87 | FALSE |
| SRX107363 | TFs and others | ESR1 | Breast | H3396 | 6910 | 443/31605 | -0.47/0.26 | -31.7 | -31.4 | -11.86 | FALSE |
| SRX3110715 | TFs and others | ESR1 | Breast | MCF-7 | 759 | 130/31605 | -1.08/0.26 | -31.7 | -31.4 | -11.86 | FALSE |
| SRX3110732 | TFs and others | ESR1 | Breast | MCF-7 | 49854 | 6897/31605 | 0.07/0.26 | -31.7 | -31.3 | -11.85 | FALSE |
| SRX11120882 | TFs and others | RAD21 | Breast | MCF-7 | 37589 | 6388/31605 | 0.07/0.26 | -31.7 | -31.3 | -11.85 | FALSE |
| SRX10641820 | TFs and others | TP73 | Breast | SUM 159PT | 13818 | 2955/31605 | -0.02/0.26 | -31.6 | -31.3 | -11.84 | FALSE |
| SRX7644286 | TFs and others | CPSF3L | Breast | MCF-7 | 1811 | 1389/31605 | -0.15/0.26 | -31.6 | -31.3 | -11.84 | FALSE |
| SRX2194247 | TFs and others | CEBPB | Breast | SUM 159PT | 50599 | 5468/31605 | 0.05/0.26 | -31.6 | -31.3 | -11.84 | FALSE |
| SRX19662631 | TFs and others | MAX | Breast | T-47D | 1246 | 1115/31605 | -0.20/0.26 | -31.6 | -31.2 | -11.83 | FALSE |
| SRX1156478 | TFs and others | GATA3 | Breast | MDA-MB-231 | 27225 | 3289/31605 | -0.01/0.26 | -31.6 | -31.2 | -11.83 | FALSE |
| SRX10773097 | TFs and others | CTCF | Breast | MB 157 | 25654 | 5091/31605 | 0.04/0.26 | -31.5 | -31.2 | -11.83 | FALSE |
| SRX13340678 | TFs and others | Epitope tags | Breast | T-47D | 5209 | 678/31605 | -0.33/0.26 | -31.5 | -31.1 | -11.81 | FALSE |
| SRX15039966 | TFs and others | GATA3 | Breast | MDA-MB-231 | 61668 | 5862/31605 | 0.06/0.26 | -31.5 | -31.1 | -11.81 | FALSE |
| SRX5203294 | TFs and others | FOXA1 | Breast | MCF-7 | 23792 | 1587/31605 | -0.12/0.26 | -31.4 | -31.0 | -11.79 | FALSE |
| SRX13300073 | TFs and others | CTCF | Breast | MCF 10A | 37428 | 6073/31605 | 0.06/0.26 | -31.3 | -31.0 | -11.79 | FALSE |
| SRX747785 | TFs and others | RELA | Breast | MCF-7 | 758 | 492/31605 | -0.42/0.26 | -31.2 | -30.9 | -11.77 | FALSE |
| SRX3923519 | TFs and others | FOXA1 | Breast | MCF-7 | 36928 | 3344/31605 | -0.01/0.26 | -31.2 | -30.9 | -11.76 | FALSE |
| SRX14101904 | TFs and others | KAT2B | Breast | MCF-7 | 3721 | 1379/31605 | -0.15/0.26 | -31.1 | -30.8 | -11.75 | FALSE |
| SRX5287695 | TFs and others | YAP1 | Breast | T-47D | 10078 | 1242/31605 | -0.17/0.26 | -31.0 | -30.7 | -11.72 | FALSE |
| SRX8321653 | TFs and others | CDYL2 | Breast | MCF-7 | 30330 | 12097/31605 | 0.12/0.26 | -31.0 | -30.7 | -11.72 | FALSE |
| SRX20282950 | TFs and others | AHR | Breast | MCF-7 | 1611 | 196/31605 | -0.82/0.26 | -31.0 | -30.7 | -11.72 | FALSE |
| SRX4611744 | TFs and others | ESR1 | Breast | MCF-7 | 55614 | 7486/31605 | 0.08/0.26 | -31.0 | -30.7 | -11.72 | FALSE |
| SRX667409 | TFs and others | Epitope tags | Breast | MCF-7 | 11863 | 5441/31605 | 0.05/0.26 | -30.9 | -30.5 | -11.70 | FALSE |
| SRX3800710 | TFs and others | CTCF | Breast | T-47D | 36473 | 5865/31605 | 0.06/0.26 | -30.9 | -30.5 | -11.69 | FALSE |
| SRX10971496 | TFs and others | ESR1 | Breast | MCF-7 | 864 | 216/31605 | -0.76/0.26 | -30.9 | -30.5 | -11.69 | FALSE |
| SRX18013279 | TFs and others | VGLL1 | Breast | FULVR | 26731 | 3090/31605 | -0.01/0.26 | -30.8 | -30.5 | -11.68 | FALSE |
| SRX673717 | TFs and others | Biotin | Breast | MCF-7 | 933 | 76/31605 | -1.46/0.26 | -30.8 | -30.5 | -11.68 | FALSE |
| SRX8537742 | TFs and others | FOXA1 | Breast | T-47D | 13390 | 1904/31605 | -0.09/0.26 | -30.7 | -30.4 | -11.67 | FALSE |
| SRX9419936 | TFs and others | BRD4 | Breast | BT-474 | 22198 | 3764/31605 | 0.01/0.26 | -30.7 | -30.4 | -11.66 | FALSE |
| SRX3800711 | TFs and others | CTCF | Breast | T-47D | 31892 | 5330/31605 | 0.05/0.26 | -30.7 | -30.4 | -11.66 | FALSE |
| SRX3229128 | TFs and others | ESR1 | Breast | Breast cancer | 7455 | 1056/31605 | -0.20/0.26 | -30.7 | -30.3 | -11.66 | FALSE |
| SRX1156479 | TFs and others | GATA3 | Breast | MDA-MB-231 | 28263 | 3422/31605 | 0.00/0.26 | -30.7 | -30.3 | -11.65 | FALSE |
| SRX19210519 | TFs and others | NFKB2 | Breast | SUM 149PT | 3677 | 1901/31605 | -0.09/0.26 | -30.6 | -30.3 | -11.65 | FALSE |
| SRX185910 | TFs and others | FOXM1 | Breast | MCF-7 | 589 | 93/31605 | -1.29/0.26 | -30.6 | -30.3 | -11.65 | FALSE |
| SRX7627556 | TFs and others | ESRRG | Breast | BT-474 | 5078 | 928/31605 | -0.23/0.26 | -30.6 | -30.3 | -11.64 | FALSE |
| SRX10775049 | TFs and others | RNF2 | Breast | T-47D | 1911 | 297/31605 | -0.61/0.26 | -30.6 | -30.2 | -11.64 | FALSE |
| SRX673738 | TFs and others | ESR1 | Breast | MCF-7 | 603 | 55/31605 | -1.75/0.26 | -30.6 | -30.2 | -11.64 | FALSE |
| SRX3923529 | TFs and others | FOXA1 | Breast | ZR-75-1 | 58103 | 5059/31605 | 0.05/0.26 | -30.4 | -30.0 | -11.60 | FALSE |
| SRX7366776 | TFs and others | KDM5C | Breast | MCF-7 | 877 | 95/31605 | -1.27/0.26 | -30.4 | -30.0 | -11.59 | FALSE |
| SRX080338 | TFs and others | CTCF | Breast | HMF | 45453 | 7108/31605 | 0.08/0.26 | -30.3 | -30.0 | -11.59 | FALSE |
| SRX490427 | TFs and others | BRD4 | Breast | MCF-7 | 1699 | 1206/31605 | -0.17/0.26 | -30.3 | -29.9 | -11.58 | FALSE |
| SRX10365521 | TFs and others | ESR1 | Breast | MDA-MB-231 | 755 | 151/31605 | -0.95/0.26 | -30.2 | -29.9 | -11.57 | FALSE |
| SRX395616 | TFs and others | CTCF | Breast | T-47D | 44113 | 6486/31605 | 0.07/0.26 | -30.2 | -29.9 | -11.56 | FALSE |
| SRX3040149 | TFs and others | SMC1A | Breast | HMEC | 51310 | 7711/31605 | 0.09/0.26 | -30.2 | -29.9 | -11.56 | FALSE |
| SRX7088154 | TFs and others | EPAS1 | Breast | MDA-MB-231 | 3016 | 710/31605 | -0.30/0.26 | -30.2 | -29.8 | -11.56 | FALSE |
| SRX3800731 | TFs and others | RAD21 | Breast | T-47D | 11566 | 5223/31605 | 0.05/0.26 | -30.1 | -29.8 | -11.54 | FALSE |
| SRX190224 | TFs and others | FOSL2 | Breast | MCF-7 | 23674 | 3034/31605 | -0.01/0.26 | -30.1 | -29.7 | -11.54 | FALSE |
| SRX7122002 | TFs and others | GRHL1 | Breast | MCF-7 | 7765 | 1176/31605 | -0.18/0.26 | -30.1 | -29.7 | -11.54 | FALSE |
| SRX4714479 | TFs and others | CTCF | Breast | T-47D | 44119 | 6485/31605 | 0.07/0.26 | -30.1 | -29.7 | -11.54 | FALSE |
| SRX11150127 | TFs and others | GATA3 | Breast | ZR-75-1 | 55088 | 3991/31605 | 0.02/0.26 | -30.0 | -29.7 | -11.53 | FALSE |
| SRX2378694 | TFs and others | AHR | Breast | MCF-7 | 5391 | 582/31605 | -0.36/0.26 | -30.0 | -29.7 | -11.53 | FALSE |
| SRX2424762 | TFs and others | CUX1 | Breast | MCF-7 | 6846 | 972/31605 | -0.22/0.26 | -30.0 | -29.7 | -11.52 | FALSE |
| SRX1012612 | TFs and others | EP300 | Breast | T-47D | 13392 | 1665/31605 | -0.11/0.26 | -30.0 | -29.7 | -11.52 | FALSE |
| SRX5457130 | TFs and others | NEUROD1 | Breast | MCF-7 | 4005 | 664/31605 | -0.32/0.26 | -30.0 | -29.6 | -11.52 | FALSE |
| SRX3923511 | TFs and others | FOXA1 | Breast | MCF-7 | 27363 | 2345/31605 | -0.05/0.26 | -30.0 | -29.6 | -11.52 | FALSE |
| SRX5806609 | TFs and others | CTCF | Breast | MCF-7 | 52328 | 7239/31605 | 0.08/0.26 | -30.0 | -29.6 | -11.51 | FALSE |
| SRX2423589 | TFs and others | SREBF1 | Breast | MCF-7 | 3763 | 1002/31605 | -0.21/0.26 | -29.9 | -29.6 | -11.51 | FALSE |
| SRX6860634 | TFs and others | RNF2 | Breast | T-47D | 2022 | 93/31605 | -1.27/0.26 | -29.9 | -29.6 | -11.50 | FALSE |
| SRX1733712 | TFs and others | ESR1 | Breast | MCF-7 | 1106 | 109/31605 | -1.15/0.26 | -29.9 | -29.5 | -11.50 | FALSE |
| SRX18013266 | TFs and others | CTCF | Breast | MCF 10A | 49559 | 8126/31605 | 0.09/0.26 | -29.8 | -29.5 | -11.49 | FALSE |
| SRX14875683 | TFs and others | ESR1 | Breast | MCF-7 | 9014 | 976/31605 | -0.22/0.26 | -29.8 | -29.5 | -11.48 | FALSE |
| SRX469394 | TFs and others | ESR1 | Breast | MCF-7 | 1649 | 160/31605 | -0.91/0.26 | -29.8 | -29.4 | -11.48 | FALSE |
| SRX1012606 | TFs and others | EP300 | Breast | T-47D | 2631 | 172/31605 | -0.87/0.26 | -29.8 | -29.4 | -11.48 | FALSE |
| SRX3923518 | TFs and others | FOXA1 | Breast | MCF-7 | 39112 | 3488/31605 | 0.01/0.26 | -29.7 | -29.4 | -11.47 | FALSE |
| SRX10119375 | TFs and others | YAP1 | Breast | MCF 10A | 33364 | 4014/31605 | 0.02/0.26 | -29.7 | -29.4 | -11.47 | FALSE |
| SRX747787 | TFs and others | RELA | Breast | MCF-7 | 680 | 313/31605 | -0.57/0.26 | -29.6 | -29.3 | -11.44 | FALSE |
| SRX7846777 | TFs and others | ESR1 | Breast | MCF-7 | 1188 | 40/31605 | -2.06/0.26 | -29.6 | -29.3 | -11.44 | FALSE |
| SRX5089517 | TFs and others | SMARCA4 | Breast | MCF-7 | 11070 | 2238/31605 | -0.05/0.26 | -29.6 | -29.2 | -11.44 | FALSE |
| SRX6712592 | TFs and others | MED26 | Breast | MCF-7 | 1281 | 557/31605 | -0.36/0.26 | -29.5 | -29.1 | -11.42 | FALSE |
| SRX5457524 | TFs and others | CLOCK | Breast | MCF-7 | 3628 | 988/31605 | -0.21/0.26 | -29.4 | -29.1 | -11.41 | FALSE |
| SRX5251427 | TFs and others | ESR1 | Breast | T-47D | 1103 | 101/31605 | -1.20/0.26 | -29.4 | -29.1 | -11.41 | FALSE |
| SRX11120872 | TFs and others | AFF4 | Breast | MCF-7 | 2124 | 1257/31605 | -0.16/0.26 | -29.4 | -29.1 | -11.40 | FALSE |
| SRX3321897 | TFs and others | ZNF579 | Breast | MCF-7 | 3685 | 903/31605 | -0.23/0.26 | -29.4 | -29.0 | -11.40 | FALSE |
| SRX6712622 | TFs and others | SMC1A | Breast | MCF-7 | 41859 | 5978/31605 | 0.07/0.26 | -29.3 | -29.0 | -11.39 | FALSE |
| SRX4792911 | TFs and others | RAD21 | Breast | T-47D | 10309 | 4770/31605 | 0.04/0.26 | -29.3 | -29.0 | -11.38 | FALSE |
| SRX484462 | TFs and others | FOXA2 | Breast | MDA-MB-231 | 26304 | 3389/31605 | 0.00/0.26 | -29.3 | -29.0 | -11.38 | FALSE |
| SRX19662639 | TFs and others | SIN3A | Breast | T-47D | 248 | 90/31605 | -1.28/0.26 | -29.2 | -28.9 | -11.37 | FALSE |
| SRX186816 | TFs and others | ESR1 | Breast | Breast cancer | 1114 | 49/31605 | -1.82/0.26 | -29.1 | -28.8 | -11.35 | FALSE |
| SRX2578885 | TFs and others | MED1 | Breast | MDA-MB-231 | 1793 | 500/31605 | -0.39/0.26 | -29.0 | -28.7 | -11.33 | FALSE |
| SRX3322394 | TFs and others | RAD51 | Breast | MCF-7 | 3015 | 1123/31605 | -0.18/0.26 | -29.0 | -28.7 | -11.33 | FALSE |
| SRX1156536 | TFs and others | SMARCA4 | Breast | MDA-MB-231 | 10237 | 3866/31605 | 0.02/0.26 | -29.0 | -28.7 | -11.33 | FALSE |
| SRX199859 | TFs and others | CTCF | Breast | HMEC | 42246 | 6365/31605 | 0.07/0.26 | -29.0 | -28.7 | -11.33 | FALSE |
| SRX199886 | TFs and others | CTCF | Breast | MCF-7 | 53666 | 6934/31605 | 0.08/0.26 | -29.0 | -28.7 | -11.32 | FALSE |
| SRX6853578 | TFs and others | ARNT | Breast | MCF-7 | 844 | 508/31605 | -0.39/0.26 | -29.0 | -28.7 | -11.32 | FALSE |
| SRX3285592 | TFs and others | KDM5B | Breast | FULR | 5445 | 2069/31605 | -0.06/0.26 | -29.0 | -28.6 | -11.32 | FALSE |
| SRX15826236 | TFs and others | KDM6A | Breast | MCF-7 | 22739 | 2008/31605 | -0.07/0.26 | -29.0 | -28.6 | -11.32 | FALSE |
| SRX5089533 | TFs and others | ARID2 | Breast | MCF-7 | 5274 | 393/31605 | -0.48/0.26 | -29.0 | -28.6 | -11.31 | FALSE |
| SRX5090157 | TFs and others | CTCF | Breast | MCF-7 | 55408 | 7215/31605 | 0.08/0.26 | -28.9 | -28.5 | -11.30 | FALSE |
| SRX355611 | TFs and others | ESR1 | Breast | MDA-MB-134-VI | 1983 | 63/31605 | -1.56/0.26 | -28.7 | -28.4 | -11.27 | FALSE |
| SRX20282964 | TFs and others | AHR | Breast | MCF-7 | 10456 | 1249/31605 | -0.15/0.26 | -28.7 | -28.4 | -11.26 | FALSE |
| SRX5089383 | TFs and others | ARID1A | Breast | MCF-7 | 15402 | 1594/31605 | -0.11/0.26 | -28.7 | -28.4 | -11.26 | FALSE |
| SRX3923528 | TFs and others | FOXA1 | Breast | ZR-75-1 | 47618 | 4179/31605 | 0.03/0.26 | -28.7 | -28.3 | -11.26 | FALSE |
| SRX3285584 | TFs and others | KDM5B | Breast | MCF-7 | 3486 | 1902/31605 | -0.08/0.26 | -28.6 | -28.3 | -11.25 | FALSE |
| SRX7122000 | TFs and others | FOXA1 | Breast | MCF-7 | 12760 | 1343/31605 | -0.14/0.26 | -28.6 | -28.3 | -11.24 | FALSE |
| SRX2897219 | TFs and others | ESR1 | Breast | Breast cancer cells | 2274 | 1297/31605 | -0.14/0.26 | -28.6 | -28.3 | -11.24 | FALSE |
| SRX12193400 | TFs and others | ESR1 | Breast | MCF-7 | 716 | 80/31605 | -1.35/0.26 | -28.5 | -28.2 | -11.23 | FALSE |
| SRX11374767 | TFs and others | PGR | Breast | T-47D | 39764 | 3903/31605 | 0.03/0.26 | -28.5 | -28.2 | -11.23 | FALSE |
| SRX5089413 | TFs and others | SMARCA4 | Breast | MCF-7 | 14514 | 3143/31605 | -0.00/0.26 | -28.5 | -28.2 | -11.22 | FALSE |
| SRX5287702 | TFs and others | MED1 | Breast | MCF-7 | 1050 | 458/31605 | -0.42/0.26 | -28.4 | -28.1 | -11.21 | FALSE |
| SRX3923516 | TFs and others | FOXA1 | Breast | MCF-7 | 41918 | 3782/31605 | 0.02/0.26 | -28.4 | -28.1 | -11.20 | FALSE |
| SRX11120884 | TFs and others | RAD21 | Breast | MCF-7 | 31127 | 5326/31605 | 0.06/0.26 | -28.4 | -28.1 | -11.20 | FALSE |
| SRX4192132 | TFs and others | XRCC4 | Breast | MCF 10A | 1520 | 991/31605 | -0.20/0.26 | -28.4 | -28.1 | -11.20 | FALSE |
| SRX5089425 | TFs and others | SMARCA4 | Breast | MCF-7 | 14478 | 2791/31605 | -0.02/0.26 | -28.4 | -28.0 | -11.19 | FALSE |
| SRX5203306 | TFs and others | ESR1 | Breast | MCF-7 | 925 | 35/31605 | -2.17/0.26 | -28.4 | -28.0 | -11.19 | FALSE |
| SRX3546799 | TFs and others | Epitope tags | Breast | MDA-MB-231 | 657 | 126/31605 | -1.02/0.26 | -28.3 | -28.0 | -11.18 | FALSE |
| SRX3923532 | TFs and others | FOXA1 | Breast | ZR-75-1 | 47125 | 4152/31605 | 0.03/0.26 | -28.3 | -27.9 | -11.18 | FALSE |
| SRX5089412 | TFs and others | SMARCA4 | Breast | MCF-7 | 6242 | 1129/31605 | -0.17/0.26 | -28.3 | -27.9 | -11.18 | FALSE |
| SRX19210549 | TFs and others | RELA | Breast | MDA-MB-231 | 5991 | 2208/31605 | -0.05/0.26 | -28.2 | -27.9 | -11.17 | FALSE |
| SRX16646095 | TFs and others | HSF1 | Breast | MCF-7 | 13789 | 2668/31605 | -0.02/0.26 | -28.2 | -27.9 | -11.16 | FALSE |
| SRX4547415 | TFs and others | CTCF | Breast | FULVR | 12252 | 3457/31605 | 0.01/0.26 | -28.1 | -27.8 | -11.15 | FALSE |
| SRX1531789 | TFs and others | MED1 | Breast | MCF-7 | 963 | 89/31605 | -1.26/0.26 | -28.1 | -27.8 | -11.14 | FALSE |
| SRX2423668 | TFs and others | DPF2 | Breast | MCF-7 | 11304 | 1128/31605 | -0.17/0.26 | -28.0 | -27.7 | -11.12 | FALSE |
| SRX176887 | TFs and others | ESR1 | Breast | ZR-75-1 | 1569 | 61/31605 | -1.57/0.26 | -28.0 | -27.7 | -11.12 | FALSE |
| SRX186697 | TFs and others | EZH2 | Breast | HMEC | 5237 | 1514/31605 | 0.62/0.26 | -28.0 | -27.6 | 11.11 | TRUE |
| SRX3722705 | TFs and others | ESR1 | Breast | Breast cancer cells | 31468 | 5228/31605 | 0.06/0.26 | -28.0 | -27.6 | -11.11 | FALSE |
| SRX6712576 | TFs and others | ESR1 | Breast | MCF-7 | 49418 | 7348/31605 | 0.09/0.26 | -27.9 | -27.6 | -11.11 | FALSE |
| SRX9719179 | TFs and others | GATA3 | Breast | MCF-7 | 19062 | 1855/31605 | -0.08/0.26 | -27.9 | -27.6 | -11.11 | FALSE |
| SRX13991448 | TFs and others | LEF1 | Breast | MCF 10A | 1410 | 1271/31605 | -0.14/0.26 | -27.9 | -27.6 | -11.11 | FALSE |
| SRX25289345 | TFs and others | GRHL2 | Breast | MCF-7 | 6057 | 1327/31605 | -0.13/0.26 | -27.8 | -27.5 | -11.09 | FALSE |
| SRX5202981 | TFs and others | FOXA1 | Breast | MCF-7 | 18543 | 1974/31605 | -0.06/0.26 | -27.8 | -27.5 | -11.09 | FALSE |
| SRX3110729 | TFs and others | ESR1 | Breast | MCF-7 | 43881 | 6361/31605 | 0.08/0.26 | -27.8 | -27.5 | -11.09 | FALSE |
| SRX3229180 | TFs and others | FOXA1 | Breast | Breast cancer | 14574 | 1773/31605 | -0.08/0.26 | -27.8 | -27.5 | -11.08 | FALSE |
| SRX2871343 | TFs and others | GATA3 | Breast | T-47D | 9454 | 1363/31605 | -0.13/0.26 | -27.8 | -27.5 | -11.08 | FALSE |
| SRX3722706 | TFs and others | ESR1 | Breast | Breast cancer cells | 34966 | 4856/31605 | 0.05/0.26 | -27.8 | -27.5 | -11.08 | FALSE |
| SRX4792906 | TFs and others | CTCF | Breast | T-47D | 27897 | 7489/31605 | 0.09/0.26 | -27.8 | -27.5 | -11.08 | FALSE |
| SRX5457473 | TFs and others | TRIM22 | Breast | MCF-7 | 18070 | 3221/31605 | 0.01/0.26 | -27.7 | -27.4 | -11.07 | FALSE |
| SRX1787681 | TFs and others | KMT2C | Breast | MCF-7 | 6587 | 633/31605 | -0.31/0.26 | -27.7 | -27.4 | -11.06 | FALSE |
| SRX3321934 | TFs and others | GABPA | Breast | MCF-7 | 1273 | 1168/31605 | -0.16/0.26 | -27.7 | -27.4 | -11.06 | FALSE |
| SRX3923517 | TFs and others | FOXA1 | Breast | MCF-7 | 41374 | 3690/31605 | 0.02/0.26 | -27.7 | -27.4 | -11.06 | FALSE |
| SRX5185601 | TFs and others | CARM1 | Breast | MCF-7 | 1029 | 208/31605 | -0.73/0.26 | -27.7 | -27.4 | -11.06 | FALSE |
| SRX5534848 | TFs and others | ESR1 | Breast | MCF-7 | 628 | 104/31605 | -1.13/0.26 | -27.7 | -27.3 | -11.05 | FALSE |
| SRX4192137 | TFs and others | XRCC4 | Breast | MCF 10A | 1412 | 937/31605 | -0.21/0.26 | -27.7 | -27.3 | -11.05 | FALSE |
| SRX4786553 | TFs and others | TET2 | Breast | MCF-7 | 1481 | 1093/31605 | -0.17/0.26 | -27.6 | -27.3 | -11.04 | FALSE |
| SRX1012607 | TFs and others | EP300 | Breast | T-47D | 2377 | 419/31605 | -0.44/0.26 | -27.6 | -27.3 | -11.04 | FALSE |
| SRX2424431 | TFs and others | RFX1 | Breast | MCF-7 | 9121 | 2521/31605 | -0.03/0.26 | -27.6 | -27.3 | -11.04 | FALSE |
| SRX5203314 | TFs and others | FOXA1 | Breast | MCF-7 | 17213 | 1288/31605 | -0.14/0.26 | -27.6 | -27.2 | -11.03 | FALSE |
| SRX4792901 | TFs and others | CTCF | Breast | T-47D | 29129 | 8255/31605 | 0.10/0.26 | -27.5 | -27.2 | -11.02 | FALSE |
| SRX5090156 | TFs and others | CTCF | Breast | MCF-7 | 40657 | 5358/31605 | 0.06/0.26 | -27.5 | -27.2 | -11.01 | FALSE |
| SRX4714474 | TFs and others | CTCF | Breast | T-47D | 33654 | 5552/31605 | 0.07/0.26 | -27.4 | -27.1 | -11.00 | FALSE |
| SRX361222 | TFs and others | GATA3 | Breast | MCF-7 | 19828 | 1688/31605 | -0.09/0.26 | -27.4 | -27.0 | -10.99 | FALSE |
| SRX10119368 | TFs and others | WWTR1 | Breast | MCF 10A | 34129 | 4107/31605 | 0.04/0.26 | -27.3 | -27.0 | -10.98 | FALSE |
| SRX2897200 | TFs and others | ESR1 | Breast | Breast cancer cells | 1123 | 526/31605 | -0.36/0.26 | -27.3 | -27.0 | -10.98 | FALSE |
| SRX747794 | TFs and others | FOXA1 | Breast | MCF-7 | 17355 | 1788/31605 | -0.08/0.26 | -27.3 | -26.9 | -10.97 | FALSE |
| SRX2423667 | TFs and others | DPF2 | Breast | MCF-7 | 8195 | 919/31605 | -0.21/0.26 | -27.3 | -26.9 | -10.97 | FALSE |
| SRX2897204 | TFs and others | ESR1 | Breast | Breast cancer cells | 783 | 62/31605 | -1.53/0.26 | -27.2 | -26.9 | -10.95 | FALSE |
| SRX3923531 | TFs and others | FOXA1 | Breast | ZR-75-1 | 40663 | 3682/31605 | 0.02/0.26 | -27.1 | -26.8 | -10.94 | FALSE |
| SRX457215 | TFs and others | ESR1 | Breast | MCF-7 | 786 | 37/31605 | -2.05/0.26 | -27.1 | -26.8 | -10.93 | FALSE |
| SRX1012608 | TFs and others | EP300 | Breast | T-47D | 2301 | 273/31605 | -0.59/0.26 | -27.1 | -26.8 | -10.93 | FALSE |
| SRX730438 | TFs and others | NCAPG | Breast | MCF-7 | 2802 | 1035/31605 | -0.18/0.26 | -27.0 | -26.7 | -10.91 | FALSE |
| SRX3075718 | TFs and others | DNMT3A | Breast | MCF-7 | 32925 | 6390/31605 | 0.08/0.26 | -26.9 | -26.6 | -10.90 | FALSE |
| SRX18013281 | TFs and others | TEAD4 | Breast | FULVR | 15009 | 2262/31605 | -0.04/0.26 | -26.9 | -26.6 | -10.89 | FALSE |
| SRX747784 | TFs and others | RELA | Breast | MCF-7 | 652 | 299/31605 | -0.55/0.26 | -26.9 | -26.6 | -10.89 | FALSE |
| SRX18013283 | TFs and others | TEAD4 | Breast | MCF-7 | 5413 | 993/31605 | -0.19/0.26 | -26.9 | -26.6 | -10.89 | FALSE |
| SRX667412 | TFs and others | Epitope tags | Breast | MCF-7 | 18227 | 5919/31605 | 0.07/0.26 | -26.9 | -26.5 | -10.88 | FALSE |
| SRX5089536 | TFs and others | ARID2 | Breast | MCF-7 | 4743 | 359/31605 | -0.48/0.26 | -26.8 | -26.5 | -10.86 | FALSE |
| SRX5323844 | TFs and others | FOXA1 | Breast | MCF-7 | 22969 | 1811/31605 | -0.07/0.26 | -26.7 | -26.4 | -10.86 | FALSE |
| SRX2423588 | TFs and others | SREBF1 | Breast | MCF-7 | 2834 | 885/31605 | -0.21/0.26 | -26.7 | -26.4 | -10.85 | FALSE |
| SRX2424328 | TFs and others | ZNF592 | Breast | MCF-7 | 2355 | 636/31605 | -0.30/0.26 | -26.7 | -26.4 | -10.85 | FALSE |
| SRX3722707 | TFs and others | ESR1 | Breast | Breast cancer cells | 33606 | 5202/31605 | 0.06/0.26 | -26.6 | -26.3 | -10.84 | FALSE |
| SRX10205610 | TFs and others | YAP1 | Breast | CAL-120 | 16096 | 2604/31605 | -0.02/0.26 | -26.6 | -26.3 | -10.83 | FALSE |
| SRX3583196 | TFs and others | CTCF | Breast | MCF-7 | 35226 | 5575/31605 | 0.07/0.26 | -26.5 | -26.2 | -10.82 | FALSE |
| SRX9419935 | TFs and others | BRD4 | Breast | BT-474 | 20758 | 3499/31605 | 0.02/0.26 | -26.5 | -26.2 | -10.82 | FALSE |
| SRX5287688 | TFs and others | YAP1 | Breast | MCF-7 | 8742 | 1129/31605 | -0.16/0.26 | -26.5 | -26.2 | -10.81 | FALSE |
| SRX7644280 | TFs and others | WTAP | Breast | MCF-7 | 772 | 301/31605 | -0.54/0.26 | -26.5 | -26.2 | -10.81 | FALSE |
| SRX1012676 | TFs and others | ESR1 | Breast | T-47D | 1491 | 99/31605 | -1.14/0.26 | -26.5 | -26.2 | -10.80 | FALSE |
| SRX100393 | TFs and others | CTCF | Breast | T-47D | 30019 | 4797/31605 | 0.06/0.26 | -26.4 | -26.1 | -10.80 | FALSE |
| SRX3323022 | TFs and others | ATF7 | Breast | MCF-7 | 10685 | 1757/31605 | -0.07/0.26 | -26.4 | -26.1 | -10.80 | FALSE |
| SRX11150133 | TFs and others | GATA3 | Breast | Breast cancer cells | 13405 | 1366/31605 | -0.12/0.26 | -26.4 | -26.0 | -10.78 | FALSE |
| SRX1091825 | TFs and others | CTCF | Breast | MCF-7 | 66836 | 8086/31605 | 0.10/0.26 | -26.3 | -26.0 | -10.78 | FALSE |
| SRX6712577 | TFs and others | ESR1 | Breast | MCF-7 | 45908 | 6810/31605 | 0.09/0.26 | -26.3 | -26.0 | -10.76 | FALSE |
| SRX5323846 | TFs and others | FOXA1 | Breast | MCF-7 | 26691 | 2052/31605 | -0.05/0.26 | -26.3 | -26.0 | -10.76 | FALSE |
| SRX730444 | TFs and others | EP300 | Breast | MCF-7 | 1831 | 472/31605 | -0.38/0.26 | -26.3 | -25.9 | -10.76 | FALSE |
| SRX4116051 | TFs and others | ESR1 | Breast | Breast cancer cells | 488 | 188/31605 | -0.75/0.26 | -26.2 | -25.9 | -10.75 | FALSE |
| SRX15391845 | TFs and others | ESR1 | Breast | T-47D | 1414 | 35/31605 | -2.07/0.26 | -26.2 | -25.9 | -10.74 | FALSE |
| SRX1012609 | TFs and others | EP300 | Breast | T-47D | 12036 | 1428/31605 | -0.11/0.26 | -26.1 | -25.8 | -10.73 | FALSE |
| SRX6860645 | TFs and others | RNF2 | Breast | T-47D | 1624 | 77/31605 | -1.31/0.26 | -26.1 | -25.8 | -10.73 | FALSE |
| SRX9045668 | TFs and others | ESR1 | Breast | MCF-7 | 867 | 104/31605 | -1.09/0.26 | -26.1 | -25.8 | -10.72 | FALSE |
| SRX3923513 | TFs and others | FOXA1 | Breast | MCF-7 | 23821 | 2070/31605 | -0.05/0.26 | -26.0 | -25.7 | -10.71 | FALSE |
| SRX15216102 | TFs and others | MEN1 | Breast | MCF-7 | 1080 | 315/31605 | -0.52/0.26 | -26.0 | -25.7 | -10.71 | FALSE |
| SRX5089411 | TFs and others | SMARCA4 | Breast | MCF-7 | 7192 | 754/31605 | -0.24/0.26 | -26.0 | -25.7 | -10.70 | FALSE |
| SRX10119380 | TFs and others | STAT3 | Breast | MCF 10A | 11838 | 2345/31605 | -0.03/0.26 | -26.0 | -25.7 | -10.70 | FALSE |
| SRX150479 | TFs and others | STAT3 | Breast | MCF 10A | 48184 | 5012/31605 | 0.06/0.26 | -25.9 | -25.6 | -10.68 | FALSE |
| SRX1758482 | TFs and others | NELFE | Breast | MCF-7 | 1381 | 950/31605 | -0.19/0.26 | -25.9 | -25.6 | -10.68 | FALSE |
| SRX3958389 | TFs and others | WDR5 | Breast | Breast cancer cells | 2261 | 804/31605 | -0.23/0.26 | -25.9 | -25.6 | -10.67 | FALSE |
| SRX6853580 | TFs and others | ARNT | Breast | MCF-7 | 1302 | 576/31605 | -0.31/0.26 | -25.9 | -25.5 | -10.67 | FALSE |
| SRX2578891 | TFs and others | NCOA1 | Breast | MDA-MB-231 | 1986 | 608/31605 | -0.30/0.26 | -25.8 | -25.5 | -10.66 | FALSE |
| SRX13991446 | TFs and others | LEF1 | Breast | MCF 10A | 1204 | 1062/31605 | -0.16/0.26 | -25.8 | -25.5 | -10.65 | FALSE |
| SRX5089418 | TFs and others | SMARCA4 | Breast | MCF-7 | 11468 | 2069/31605 | -0.04/0.26 | -25.8 | -25.4 | -10.65 | FALSE |
| SRX3923512 | TFs and others | FOXA1 | Breast | MCF-7 | 22613 | 1888/31605 | -0.06/0.26 | -25.7 | -25.4 | -10.64 | FALSE |
| SRX150517 | TFs and others | FOS | Breast | MCF 10A | 81051 | 6643/31605 | 0.09/0.26 | -25.7 | -25.4 | -10.63 | FALSE |
| SRX3110739 | TFs and others | ESR1 | Breast | MCF-7 | 40620 | 5917/31605 | 0.08/0.26 | -25.7 | -25.4 | -10.63 | FALSE |
| SRX190190 | TFs and others | CTCF | Breast | MCF-7 | 55399 | 6772/31605 | 0.09/0.26 | -25.7 | -25.4 | -10.63 | FALSE |
| SRX3322311 | TFs and others | ZNF24 | Breast | MCF-7 | 6923 | 1244/31605 | -0.13/0.26 | -25.6 | -25.3 | -10.62 | FALSE |
| SRX1012677 | TFs and others | ESR1 | Breast | T-47D | 1557 | 114/31605 | -1.02/0.26 | -25.6 | -25.3 | -10.62 | FALSE |
| SRX3110735 | TFs and others | ESR1 | Breast | MCF-7 | 43370 | 6438/31605 | 0.09/0.26 | -25.6 | -25.3 | -10.62 | FALSE |
| SRX1161181 | TFs and others | NR3C1 | Breast | ZR-75-1 | 25330 | 1670/31605 | -0.08/0.26 | -25.6 | -25.3 | -10.61 | FALSE |
| SRX5203311 | TFs and others | FOXA1 | Breast | MCF-7 | 18896 | 1278/31605 | -0.12/0.26 | -25.6 | -25.3 | -10.61 | FALSE |
| SRX3541096 | TFs and others | ESR1 | Breast | MCF-7 | 1167 | 27/31605 | -2.36/0.26 | -25.5 | -25.2 | -10.60 | FALSE |
| SRX1758526 | TFs and others | Epitope tags | Breast | MCF-7 | 1995 | 1232/31605 | -0.13/0.26 | -25.4 | -25.1 | -10.58 | FALSE |
| SRX7098805 | TFs and others | CTCF | Breast | Mammary epithelial cells | 42828 | 7807/31605 | 0.10/0.26 | -25.4 | -25.1 | -10.58 | FALSE |
| SRX5323850 | TFs and others | FOXA1 | Breast | MCF-7 | 21147 | 1822/31605 | -0.06/0.26 | -25.4 | -25.1 | -10.57 | FALSE |
| SRX13300072 | TFs and others | CTCF | Breast | MCF 10A | 31940 | 5049/31605 | 0.06/0.26 | -25.4 | -25.1 | -10.57 | FALSE |
| SRX080328 | TFs and others | CTCF | Breast | HMF | 40027 | 5623/31605 | 0.07/0.26 | -25.3 | -25.0 | -10.56 | FALSE |
| SRX3322697 | TFs and others | TARDBP | Breast | MCF-7 | 2914 | 867/31605 | -0.20/0.26 | -25.3 | -25.0 | -10.56 | FALSE |
| SRX5089441 | TFs and others | SMARCB1 | Breast | MCF-7 | 9617 | 1046/31605 | -0.16/0.26 | -25.3 | -24.9 | -10.54 | FALSE |
| SRX7644278 | TFs and others | METTL14 | Breast | MCF-7 | 1645 | 750/31605 | -0.24/0.26 | -25.2 | -24.9 | -10.54 | FALSE |
| SRX067475 | TFs and others | CTCF | Breast | HMEC | 36341 | 5274/31605 | 0.07/0.26 | -25.2 | -24.9 | -10.54 | FALSE |
| SRX15946405 | TFs and others | AR | Breast | MDA-MB-453 | 55685 | 3594/31605 | 0.03/0.26 | -25.2 | -24.9 | -10.52 | FALSE |
| SRX2582039 | TFs and others | KDM4A | Breast | BCSC1 | 36979 | 6983/31605 | 0.09/0.26 | -25.2 | -24.9 | -10.52 | FALSE |
| SRX2578896 | TFs and others | NCOA1 | Breast | MDA-MB-231 | 1065 | 373/31605 | -0.44/0.26 | -25.2 | -24.9 | -10.52 | FALSE |
| SRX11120883 | TFs and others | RAD21 | Breast | MCF-7 | 39455 | 5190/31605 | 0.07/0.26 | -25.1 | -24.8 | -10.52 | FALSE |
| SRX10478468 | TFs and others | MSX2 | Breast | MCF-7 | 2794 | 424/31605 | -0.40/0.26 | -25.1 | -24.8 | -10.51 | FALSE |
| SRX104409 | TFs and others | GATA3 | Breast | MCF-7 | 5685 | 363/31605 | -0.45/0.26 | -25.1 | -24.8 | -10.51 | FALSE |
| SRX199891 | TFs and others | CTCF | Breast | MCF-7 | 44708 | 5770/31605 | 0.08/0.26 | -25.0 | -24.7 | -10.49 | FALSE |
| SRX1012611 | TFs and others | EP300 | Breast | T-47D | 8134 | 1020/31605 | -0.17/0.26 | -25.0 | -24.7 | -10.49 | FALSE |
| SRX201314 | TFs and others | AHR | Breast | MCF-7 | 17588 | 1847/31605 | -0.06/0.26 | -25.0 | -24.7 | -10.49 | FALSE |
| SRX19210540 | TFs and others | RELB | Breast | SUM 149PT | 3253 | 1456/31605 | -0.10/0.26 | -24.9 | -24.6 | -10.47 | FALSE |
| SRX10365541 | TFs and others | RAC1 | Breast | MCF-7 | 31004 | 2166/31605 | -0.03/0.26 | -24.9 | -24.6 | -10.47 | FALSE |
| SRX3229127 | TFs and others | ESR1 | Breast | Breast cancer | 1018 | 37/31605 | -1.95/0.26 | -24.9 | -24.6 | -10.47 | FALSE |
| SRX11149848 | TFs and others | AR | Breast | MDA-MB-453 | 55722 | 3591/31605 | 0.03/0.26 | -24.9 | -24.6 | -10.46 | FALSE |
| SRX5287693 | TFs and others | TEAD4 | Breast | T-47D | 9066 | 1679/31605 | -0.07/0.26 | -24.8 | -24.5 | -10.45 | FALSE |
| SRX1165110 | TFs and others | Epitope tags | Breast | MCF-7 | 49563 | 5949/31605 | 0.08/0.26 | -24.8 | -24.5 | -10.45 | FALSE |
| SRX2636201 | TFs and others | RAD21 | Breast | MCF-7 | 49554 | 5946/31605 | 0.08/0.26 | -24.8 | -24.5 | -10.44 | FALSE |
| SRX6058760 | TFs and others | PGR | Breast | T-47D | 7718 | 1061/31605 | -0.16/0.26 | -24.8 | -24.5 | -10.43 | FALSE |
| SRX10478469 | TFs and others | MSX2 | Breast | MCF-7 | 5926 | 727/31605 | -0.24/0.26 | -24.7 | -24.4 | -10.43 | FALSE |
| SRX180167 | TFs and others | PBX1 | Breast | MCF-7-LTED | 2870 | 1272/31605 | -0.12/0.26 | -24.7 | -24.4 | -10.43 | FALSE |
| SRX15039983 | TFs and others | CHD4 | Breast | T-47D | 4554 | 1632/31605 | -0.08/0.26 | -24.7 | -24.4 | -10.41 | FALSE |
| SRX5089535 | TFs and others | ARID2 | Breast | MCF-7 | 2530 | 172/31605 | -0.76/0.26 | -24.6 | -24.3 | -10.40 | FALSE |
| SRX10365520 | TFs and others | ESR1 | Breast | MDA-MB-231 | 785 | 146/31605 | -0.85/0.26 | -24.6 | -24.3 | -10.40 | FALSE |
| SRX3322087 | TFs and others | FOXA1 | Breast | MCF-7 | 20556 | 1756/31605 | -0.06/0.26 | -24.6 | -24.3 | -10.39 | FALSE |
| SRX3322610 | TFs and others | SMARCA5 | Breast | MCF-7 | 3928 | 903/31605 | -0.19/0.26 | -24.5 | -24.2 | -10.38 | FALSE |
| SRX4417024 | TFs and others | GATA3 | Breast | MCF-7 | 5325 | 473/31605 | -0.36/0.26 | -24.5 | -24.2 | -10.38 | FALSE |
| SRX3800733 | TFs and others | RAD21 | Breast | T-47D | 13507 | 4785/31605 | 0.06/0.26 | -24.5 | -24.2 | -10.38 | FALSE |
| SRX10119388 | TFs and others | YAP1 | Breast | MDA-MB-231 | 30807 | 3618/31605 | 0.03/0.26 | -24.5 | -24.2 | -10.38 | FALSE |
| SRX3322521 | TFs and others | ELF1 | Breast | MCF-7 | 1442 | 1021/31605 | -0.16/0.26 | -24.5 | -24.2 | -10.37 | FALSE |
| SRX7897897 | TFs and others | CTCF | Breast | MDA-MB-231 | 34144 | 4884/31605 | 0.07/0.26 | -24.4 | -24.1 | -10.36 | FALSE |
| SRX2424432 | TFs and others | RFX1 | Breast | MCF-7 | 9380 | 2470/31605 | -0.01/0.26 | -24.4 | -24.1 | -10.36 | FALSE |
| SRX20282958 | TFs and others | AHR | Breast | MCF-7 | 1468 | 126/31605 | -0.93/0.26 | -24.4 | -24.1 | -10.36 | FALSE |
| SRX150630 | TFs and others | STAT3 | Breast | MCF 10A | 47711 | 4954/31605 | 0.07/0.26 | -24.4 | -24.1 | -10.35 | FALSE |
| SRX5089414 | TFs and others | SMARCA4 | Breast | MCF-7 | 11459 | 1818/31605 | -0.06/0.26 | -24.4 | -24.1 | -10.35 | FALSE |
| SRX2423883 | TFs and others | RCOR1 | Breast | MCF-7 | 2502 | 623/31605 | -0.28/0.26 | -24.4 | -24.1 | -10.35 | FALSE |
| SRX1091824 | TFs and others | CTCF | Breast | MCF-7 | 56057 | 7179/31605 | 0.10/0.26 | -24.4 | -24.1 | -10.35 | FALSE |
| SRX268299 | TFs and others | JUN | Breast | BT-549 | 16636 | 2309/31605 | -0.02/0.26 | -24.2 | -23.9 | -10.31 | FALSE |
| SRX2194248 | TFs and others | CEBPB | Breast | SUM 159PT | 53009 | 5083/31605 | 0.07/0.26 | -24.2 | -23.9 | -10.30 | FALSE |
| SRX1156534 | TFs and others | SMARCA4 | Breast | MDA-MB-231 | 9637 | 3324/31605 | 0.03/0.26 | -24.2 | -23.9 | -10.30 | FALSE |
| SRX5203297 | TFs and others | ESR1 | Breast | MCF-7 | 948 | 22/31605 | -2.55/0.26 | -24.1 | -23.8 | -10.29 | FALSE |
| SRX1156540 | TFs and others | SMARCA4 | Breast | MDA-MB-231 | 14362 | 4087/31605 | 0.05/0.26 | -24.0 | -23.7 | -10.28 | FALSE |
| SRX6828395 | TFs and others | CTCF | Breast | MCF-7 | 48682 | 6012/31605 | 0.09/0.26 | -24.0 | -23.7 | -10.27 | FALSE |
| SRX10024494 | TFs and others | FOXA1 | Breast | MCF-7 | 25488 | 2073/31605 | -0.03/0.26 | -24.0 | -23.7 | -10.27 | FALSE |
| SRX5457474 | TFs and others | TRIM22 | Breast | MCF-7 | 4912 | 940/31605 | -0.17/0.26 | -24.0 | -23.7 | -10.26 | FALSE |
| SRX7897898 | TFs and others | CTCF | Breast | MDA-MB-231 | 35119 | 5078/31605 | 0.07/0.26 | -24.0 | -23.7 | -10.26 | FALSE |
| SRX18013282 | TFs and others | TEAD4 | Breast | MCF-7 | 4085 | 732/31605 | -0.23/0.26 | -23.9 | -23.6 | -10.25 | FALSE |
| SRX5203318 | TFs and others | FOXA1 | Breast | MCF-7 | 16507 | 1020/31605 | -0.16/0.26 | -23.9 | -23.6 | -10.25 | FALSE |
| SRX10142198 | TFs and others | CTCF | Breast | Breast luminal cells | 9135 | 2020/31605 | -0.04/0.26 | -23.9 | -23.6 | -10.24 | FALSE |
| SRX5323848 | TFs and others | FOXA1 | Breast | MCF-7 | 16984 | 1443/31605 | -0.09/0.26 | -23.9 | -23.6 | -10.24 | FALSE |
| SRX20504946 | TFs and others | STAT3 | Breast | Breast cancer cells | 3480 | 1065/31605 | -0.15/0.26 | -23.8 | -23.6 | -10.23 | FALSE |
| SRX13327962 | TFs and others | CBX3 | Breast | MCF-7 | 2472 | 720/31605 | -0.23/0.26 | -23.8 | -23.6 | -10.23 | FALSE |
| SRX4547412 | TFs and others | CTCF | Breast | MCF-7 | 7833 | 2479/31605 | -0.01/0.26 | -23.8 | -23.5 | -10.23 | FALSE |
| SRX150477 | TFs and others | FOS | Breast | MCF 10A | 106540 | 7511/31605 | 0.10/0.26 | -23.8 | -23.5 | -10.23 | FALSE |
| SRX10205608 | TFs and others | YAP1 | Breast | CAL-120 | 10337 | 1936/31605 | -0.04/0.26 | -23.8 | -23.5 | -10.22 | FALSE |
| SRX3541103 | TFs and others | ESR1 | Breast | MCF-7 | 573 | 18/31605 | -2.83/0.26 | -23.8 | -23.5 | -10.22 | FALSE |
| SRX2194249 | TFs and others | CEBPB | Breast | SUM 159PT | 62074 | 5814/31605 | 0.08/0.26 | -23.6 | -23.3 | -10.18 | FALSE |
| SRX361228 | TFs and others | GATA3 | Breast | T-47D | 7034 | 976/31605 | -0.16/0.26 | -23.5 | -23.2 | -10.16 | FALSE |
| SRX4564976 | TFs and others | CTCF | Breast | MCF-7 | 37081 | 5581/31605 | 0.08/0.26 | -23.5 | -23.2 | -10.16 | FALSE |
| SRX15826239 | TFs and others | KDM6A | Breast | MCF-7 | 9665 | 883/31605 | -0.18/0.26 | -23.5 | -23.2 | -10.15 | FALSE |
| SRX6413748 | TFs and others | ESR1 | Breast | MCF-7 | 2450 | 490/31605 | -0.33/0.26 | -23.4 | -23.2 | -10.14 | FALSE |
| SRX5089408 | TFs and others | SMARCA4 | Breast | MCF-7 | 9371 | 2007/31605 | -0.03/0.26 | -23.4 | -23.1 | -10.12 | FALSE |
| SRX5089513 | TFs and others | SMARCA4 | Breast | MCF-7 | 11034 | 1638/31605 | -0.07/0.26 | -23.3 | -23.1 | -10.12 | FALSE |
| SRX038562 | TFs and others | CTCF | Breast | HMEC | 34300 | 5121/31605 | 0.07/0.26 | -23.3 | -23.0 | -10.12 | FALSE |
| SRX1012682 | TFs and others | ESR1 | Breast | MCF-7 | 1660 | 69/31605 | -1.30/0.26 | -23.3 | -23.0 | -10.10 | FALSE |
| SRX5251421 | TFs and others | ESR1 | Breast | MCF-7 | 737 | 53/31605 | -1.52/0.26 | -23.3 | -23.0 | -10.10 | FALSE |
| SRX12008347 | TFs and others | CTCF | Breast | MCF 10A | 35719 | 5445/31605 | 0.08/0.26 | -23.3 | -23.0 | -10.10 | FALSE |
| SRX4931257 | TFs and others | KMT2A | Breast | MCF-7 | 1138 | 768/31605 | -0.21/0.26 | -23.2 | -22.9 | -10.09 | FALSE |
| SRX24351553 | TFs and others | Epitope tags | Breast | MB 157 | 2429 | 1091/31605 | -0.14/0.26 | -23.2 | -22.9 | -10.09 | FALSE |
| SRX18191638 | TFs and others | ESR1 | Breast | MCF-7 | 1591 | 117/31605 | -0.94/0.26 | -23.2 | -22.9 | -10.08 | FALSE |
| SRX1161167 | TFs and others | NR3C1 | Breast | MCF-7 | 19874 | 1254/31605 | -0.11/0.26 | -23.2 | -22.9 | -10.08 | FALSE |
| SRX5203317 | TFs and others | ESR1 | Breast | MCF-7 | 901 | 23/31605 | -2.44/0.26 | -23.2 | -22.9 | -10.08 | FALSE |
| SRX5089433 | TFs and others | SMARCB1 | Breast | MCF-7 | 9902 | 1115/31605 | -0.13/0.26 | -23.1 | -22.8 | -10.07 | FALSE |
| SRX484471 | TFs and others | FOXA1 | Breast | MCF-7 | 15856 | 1616/31605 | -0.07/0.26 | -23.1 | -22.8 | -10.06 | FALSE |
| SRX16498663 | TFs and others | CTCF | Breast | MCF 10A | 27661 | 4359/31605 | 0.06/0.26 | -23.1 | -22.8 | -10.06 | FALSE |
| SRX10921875 | TFs and others | EP300 | Breast | MCF-7 | 1794 | 137/31605 | -0.84/0.26 | -23.0 | -22.7 | -10.05 | FALSE |
| SRX5323845 | TFs and others | FOXA1 | Breast | MCF-7 | 27901 | 2192/31605 | -0.02/0.26 | -23.0 | -22.7 | -10.04 | FALSE |
| SRX4671311 | TFs and others | RXRA | Breast | SUM 159PT | 10319 | 2115/31605 | -0.02/0.26 | -23.0 | -22.7 | -10.04 | FALSE |
| SRX5287686 | TFs and others | TEAD4 | Breast | MCF-7 | 5636 | 1163/31605 | -0.12/0.26 | -23.0 | -22.7 | -10.04 | FALSE |
| SRX5683085 | TFs and others | ETV4 | Breast | T-47D | 1944 | 710/31605 | -0.23/0.26 | -23.0 | -22.7 | -10.04 | FALSE |
| SRX5323863 | TFs and others | STAT3 | Breast | MCF-7 | 1008 | 400/31605 | -0.39/0.26 | -23.0 | -22.7 | -10.04 | FALSE |
| SRX7262857 | TFs and others | AR | Breast | Breast cancer cells | 20495 | 1536/31605 | -0.07/0.26 | -23.0 | -22.7 | -10.04 | FALSE |
| SRX18191676 | TFs and others | ESR1 | Breast | MCF-7 | 1281 | 44/31605 | -1.68/0.26 | -23.0 | -22.7 | -10.03 | FALSE |
| SRX968977 | TFs and others | RELA | Breast | MCF-7 | 3181 | 659/31605 | -0.25/0.26 | -23.0 | -22.7 | -10.03 | FALSE |
| SRX5202983 | TFs and others | FOXA1 | Breast | MCF-7 | 74172 | 5082/31605 | 0.08/0.26 | -22.9 | -22.6 | -10.02 | FALSE |
| SRX5323825 | TFs and others | FOXA1 | Breast | T-47D | 14819 | 1735/31605 | -0.05/0.26 | -22.9 | -22.6 | -10.02 | FALSE |
| SRX3923184 | TFs and others | FOSL1 | Breast | BT-549 | 15880 | 2185/31605 | -0.02/0.26 | -22.9 | -22.6 | -10.02 | FALSE |
| SRX16498661 | TFs and others | CTCF | Breast | MCF 10A | 29705 | 4793/31605 | 0.07/0.26 | -22.9 | -22.6 | -10.01 | FALSE |
| SRX5089386 | TFs and others | ARID1A | Breast | MCF-7 | 9296 | 630/31605 | -0.26/0.26 | -22.9 | -22.6 | -10.01 | FALSE |
| SRX5089415 | TFs and others | SMARCA4 | Breast | MCF-7 | 8442 | 2088/31605 | -0.03/0.26 | -22.8 | -22.6 | -10.01 | FALSE |
| SRX4714455 | TFs and others | GTF3C2 | Breast | T-47D | 13309 | 1562/31605 | -0.07/0.26 | -22.8 | -22.5 | -9.99 | FALSE |
| SRX5089532 | TFs and others | ARID2 | Breast | MCF-7 | 2941 | 195/31605 | -0.66/0.26 | -22.8 | -22.5 | -9.99 | FALSE |
| SRX10119372 | TFs and others | TEAD1 | Breast | MCF 10A | 20588 | 2667/31605 | 0.01/0.26 | -22.7 | -22.4 | -9.98 | FALSE |
| SRX3800728 | TFs and others | RAD21 | Breast | T-47D | 11616 | 3687/31605 | 0.04/0.26 | -22.7 | -22.4 | -9.98 | FALSE |
| SRX5323867 | TFs and others | STAT3 | Breast | MCF-7 | 1116 | 556/31605 | -0.29/0.26 | -22.6 | -22.4 | -9.96 | FALSE |
| SRX2423866 | TFs and others | ELK1 | Breast | MCF-7 | 2112 | 999/31605 | -0.15/0.26 | -22.6 | -22.3 | -9.96 | FALSE |
| SRX4213900 | TFs and others | MYBL2 | Breast | MCF 10A | 1778 | 841/31605 | -0.18/0.26 | -22.6 | -22.3 | -9.95 | FALSE |
| SRX4792910 | TFs and others | RAD21 | Breast | T-47D | 8364 | 3306/31605 | 0.03/0.26 | -22.6 | -22.3 | -9.95 | FALSE |
| SRX15929728 | TFs and others | STAG2 | Breast | MCF 10A | 40908 | 6451/31605 | 0.10/0.26 | -22.5 | -22.2 | -9.92 | FALSE |
| SRX3923515 | TFs and others | FOXA1 | Breast | MCF-7 | 35877 | 3168/31605 | 0.03/0.26 | -22.4 | -22.1 | -9.91 | FALSE |
| SRX5203298 | TFs and others | FOXA1 | Breast | MCF-7 | 10914 | 725/31605 | -0.22/0.26 | -22.4 | -22.1 | -9.91 | FALSE |
| SRX2378691 | TFs and others | AHR | Breast | MCF-7 | 4119 | 284/31605 | -0.50/0.26 | -22.4 | -22.1 | -9.90 | FALSE |
| SRX194581 | TFs and others | TLE3 | Breast | MCF-7 | 4455 | 354/31605 | -0.42/0.26 | -22.4 | -22.1 | -9.90 | FALSE |
| SRX593748 | TFs and others | SFPQ | Breast | MCF-7 | 228439 | 13335/31605 | 0.15/0.26 | -22.3 | -22.0 | -9.89 | FALSE |
| SRX7122007 | TFs and others | GRHL1 | Breast | MCF-7 | 3831 | 723/31605 | -0.22/0.26 | -22.3 | -22.0 | -9.89 | FALSE |
| SRX5287692 | TFs and others | TEAD4 | Breast | T-47D | 11470 | 2141/31605 | -0.02/0.26 | -22.3 | -22.0 | -9.88 | FALSE |
| SRX673719 | TFs and others | Biotin | Breast | MCF-7 | 1091 | 117/31605 | -0.91/0.26 | -22.3 | -22.0 | -9.88 | FALSE |
| SRX2020844 | TFs and others | STAT3 | Breast | Breast cancer cells | 6444 | 1607/31605 | -0.06/0.26 | -22.3 | -22.0 | -9.88 | FALSE |
| SRX5089515 | TFs and others | SMARCA4 | Breast | MCF-7 | 14157 | 2652/31605 | 0.01/0.26 | -22.2 | -22.0 | -9.87 | FALSE |
| SRX12008346 | TFs and others | CTCF | Breast | MCF 10A | 35101 | 5326/31605 | 0.08/0.26 | -22.2 | -21.9 | -9.87 | FALSE |
| SRX5251424 | TFs and others | ESR1 | Breast | T-47D | 864 | 105/31605 | -0.98/0.26 | -22.2 | -21.9 | -9.86 | FALSE |
| SRX1165106 | TFs and others | RAD21 | Breast | MCF-7 | 43888 | 4960/31605 | 0.08/0.26 | -22.2 | -21.9 | -9.86 | FALSE |
| SRX028634 | TFs and others | FOXA1 | Breast | MCF-7 | 37937 | 3002/31605 | 0.02/0.26 | -22.1 | -21.9 | -9.85 | FALSE |
| SRX3322637 | TFs and others | FOS | Breast | MCF-7 | 28231 | 3261/31605 | 0.03/0.26 | -22.1 | -21.8 | -9.84 | FALSE |
| SRX647367 | TFs and others | ESR1 | Breast | MCF 10A | 1898 | 744/31605 | -0.21/0.26 | -22.1 | -21.8 | -9.83 | FALSE |
| SRX4417026 | TFs and others | TFAP2C | Breast | MCF-7 | 2167 | 410/31605 | -0.37/0.26 | -22.1 | -21.8 | -9.83 | FALSE |
| SRX6487050 | TFs and others | FOXA1 | Breast | MCF-7 | 10950 | 813/31605 | -0.19/0.26 | -22.1 | -21.8 | -9.83 | FALSE |
| SRX3040153 | TFs and others | SMC1A | Breast | HMEC | 28985 | 4547/31605 | 0.07/0.26 | -22.1 | -21.8 | -9.83 | FALSE |
| SRX039146 | TFs and others | FOXA1 | Breast | T-47D | 20607 | 2138/31605 | -0.02/0.26 | -22.0 | -21.7 | -9.81 | FALSE |
| SRX747798 | TFs and others | FOXA1 | Breast | MCF-7 | 16128 | 1557/31605 | -0.06/0.26 | -22.0 | -21.7 | -9.81 | FALSE |
| SRX8488089 | TFs and others | JUNB | Breast | SUM 229PE | 41167 | 4513/31605 | 0.07/0.26 | -22.0 | -21.7 | -9.81 | FALSE |
| SRX7098804 | TFs and others | CTCF | Breast | Mammary epithelial cells | 41082 | 6485/31605 | 0.10/0.26 | -21.9 | -21.7 | -9.80 | FALSE |
| SRX11120871 | TFs and others | AFF4 | Breast | MCF-7 | 1777 | 1062/31605 | -0.13/0.26 | -21.9 | -21.6 | -9.80 | FALSE |
| SRX4374899 | TFs and others | NOTCH1 | Breast | MB 157 | 1555 | 541/31605 | -0.28/0.26 | -21.9 | -21.6 | -9.79 | FALSE |
| SRX2423882 | TFs and others | RCOR1 | Breast | MCF-7 | 3195 | 551/31605 | -0.28/0.26 | -21.8 | -21.5 | -9.77 | FALSE |
| SRX14746766 | TFs and others | HIF1A | Breast | T-47D | 1231 | 395/31605 | -0.37/0.26 | -21.8 | -21.5 | -9.77 | FALSE |
| SRX3923530 | TFs and others | FOXA1 | Breast | ZR-75-1 | 45811 | 3917/31605 | 0.06/0.26 | -21.8 | -21.5 | -9.76 | FALSE |
| SRX3322208 | TFs and others | CTCF | Breast | Breast epithelium | 9353 | 2020/31605 | -0.02/0.26 | -21.8 | -21.5 | -9.76 | FALSE |
| SRX1012693 | TFs and others | PGR | Breast | MCF-7 | 1549 | 108/31605 | -0.95/0.26 | -21.8 | -21.5 | -9.75 | FALSE |
| SRX5323849 | TFs and others | FOXA1 | Breast | MCF-7 | 17234 | 1570/31605 | -0.06/0.26 | -21.7 | -21.4 | -9.75 | FALSE |
| SRX2020839 | TFs and others | STAT3 | Breast | MDA-MB-157 | 17806 | 2371/31605 | -0.00/0.26 | -21.5 | -21.3 | -9.71 | FALSE |
| SRX673727 | TFs and others | TFAP2C | Breast | MCF-7 | 2669 | 851/31605 | -0.17/0.26 | -21.5 | -21.2 | -9.70 | FALSE |
| SRX1156473 | TFs and others | GATA3 | Breast | MDA-MB-231 | 21279 | 2506/31605 | 0.01/0.26 | -21.4 | -21.1 | -9.68 | FALSE |
| SRX1156521 | TFs and others | Epitope tags | Breast | MDA-MB-231 | 15359 | 2152/31605 | -0.01/0.26 | -21.4 | -21.1 | -9.68 | FALSE |
| SRX1143681 | TFs and others | TP63 | Breast | MCF10DCIS.com | 21815 | 2761/31605 | 0.02/0.26 | -21.4 | -21.1 | -9.67 | FALSE |
| SRX6860652 | TFs and others | ESR1 | Breast | T-47D | 1516 | 33/31605 | -1.90/0.26 | -21.4 | -21.1 | -9.67 | FALSE |
| SRX25289348 | TFs and others | GRHL2 | Breast | MCF-7 | 3845 | 854/31605 | -0.17/0.26 | -21.3 | -21.0 | -9.64 | FALSE |
| SRX5089443 | TFs and others | SMARCB1 | Breast | MCF-7 | 9420 | 916/31605 | -0.15/0.26 | -21.2 | -20.9 | -9.62 | FALSE |
| SRX11867610 | TFs and others | KMT2C | Breast | CAL-51 | 2224 | 974/31605 | -0.14/0.26 | -21.2 | -20.9 | -9.61 | FALSE |
| SRX10119376 | TFs and others | YAP1 | Breast | MCF 10A | 33751 | 3795/31605 | 0.06/0.26 | -21.1 | -20.9 | -9.61 | FALSE |
| SRX14746765 | TFs and others | HIF1A | Breast | T-47D | 1122 | 319/31605 | -0.43/0.26 | -21.1 | -20.9 | -9.61 | FALSE |
| SRX7522861 | TFs and others | CTCF | Breast | MCF10DCIS.com | 32333 | 4309/31605 | 0.07/0.26 | -21.1 | -20.8 | -9.60 | FALSE |
| SRX673720 | TFs and others | Biotin | Breast | MCF-7 | 1620 | 468/31605 | -0.31/0.26 | -21.0 | -20.8 | -9.59 | FALSE |
| SRX6058754 | TFs and others | CEBPA | Breast | T-47D | 8087 | 1546/31605 | -0.06/0.26 | -21.0 | -20.7 | -9.58 | FALSE |
| SRX201315 | TFs and others | ARNT | Breast | MCF-7 | 19884 | 1847/31605 | -0.03/0.26 | -21.0 | -20.7 | -9.58 | FALSE |
| SRX3229171 | TFs and others | AR | Breast | Breast cancer | 1066 | 273/31605 | -0.49/0.26 | -20.9 | -20.7 | -9.56 | FALSE |
| SRX10024493 | TFs and others | FOXA1 | Breast | MCF-7 | 20106 | 1689/31605 | -0.04/0.26 | -20.9 | -20.6 | -9.56 | FALSE |
| SRX10119365 | TFs and others | YAP1 | Breast | MCF 10A | 23847 | 3002/31605 | 0.03/0.26 | -20.9 | -20.6 | -9.55 | FALSE |
| SRX9719184 | TFs and others | GATA3 | Breast | MCF-7 | 18066 | 1576/31605 | -0.05/0.26 | -20.9 | -20.6 | -9.54 | FALSE |
| SRX100258 | TFs and others | CREBBP | Breast | H3396 | 478 | 61/31605 | -1.31/0.26 | -20.9 | -20.6 | -9.54 | FALSE |
| SRX13300071 | TFs and others | CTCF | Breast | MCF 10A | 26725 | 4280/31605 | 0.07/0.26 | -20.9 | -20.6 | -9.54 | FALSE |
| SRX7098803 | TFs and others | CTCF | Breast | Mammary epithelial cells | 29573 | 5519/31605 | 0.09/0.26 | -20.8 | -20.5 | -9.53 | FALSE |
| SRX5493756 | TFs and others | FOXA1 | Breast | Breast cancer cells | 8793 | 1140/31605 | -0.11/0.26 | -20.8 | -20.5 | -9.52 | FALSE |
| SRX5089530 | TFs and others | SMARCA4 | Breast | MCF-7 | 5406 | 1110/31605 | -0.11/0.26 | -20.7 | -20.4 | -9.51 | FALSE |
| SRX3583225 | TFs and others | CTCF | Breast | MCF-7 | 32671 | 5496/31605 | 0.09/0.26 | -20.7 | -20.4 | -9.50 | FALSE |
| SRX1161180 | TFs and others | NR3C1 | Breast | ZR-75-1 | 5587 | 317/31605 | -0.43/0.26 | -20.6 | -20.3 | -9.48 | FALSE |
| SRX3080211 | TFs and others | JUN | Breast | MCF-7 | 13332 | 1434/31605 | -0.07/0.26 | -20.5 | -20.3 | -9.47 | FALSE |
| SRX23740459 | TFs and others | KDM5B | Breast | SUM 149PT | 11452 | 2421/31605 | 0.01/0.26 | -20.5 | -20.2 | -9.46 | FALSE |
| SRX2423923 | TFs and others | MTA3 | Breast | MCF-7 | 1971 | 693/31605 | -0.21/0.26 | -20.5 | -20.2 | -9.46 | FALSE |
| SRX2897206 | TFs and others | ESR1 | Breast | Breast cancer cells | 793 | 159/31605 | -0.71/0.26 | -20.5 | -20.2 | -9.46 | FALSE |
| SRX6712590 | TFs and others | MED1 | Breast | MCF-7 | 855 | 490/31605 | -0.29/0.26 | -20.5 | -20.2 | -9.45 | FALSE |
| SRX15929725 | TFs and others | SMC1A | Breast | MCF 10A | 29782 | 4858/31605 | 0.08/0.26 | -20.4 | -20.2 | -9.44 | FALSE |
| SRX5457487 | TFs and others | E2F8 | Breast | MCF-7 | 1180 | 228/31605 | -0.55/0.26 | -20.4 | -20.1 | -9.44 | FALSE |
| SRX10119382 | TFs and others | STAT3 | Breast | MCF 10A | 11060 | 2111/31605 | -0.01/0.26 | -20.4 | -20.1 | -9.43 | FALSE |
| SRX4792904 | TFs and others | CTCF | Breast | T-47D | 16176 | 4599/31605 | 0.08/0.26 | -20.4 | -20.1 | -9.43 | FALSE |
| SRX2871351 | TFs and others | GATA3 | Breast | T-47D | 5986 | 656/31605 | -0.22/0.26 | -20.3 | -20.1 | -9.42 | FALSE |
| SRX6712621 | TFs and others | SMC1A | Breast | MCF-7 | 18290 | 2560/31605 | 0.02/0.26 | -20.3 | -20.0 | -9.41 | FALSE |
| SRX5203313 | TFs and others | ESR1 | Breast | MCF-7 | 818 | 18/31605 | -2.58/0.26 | -20.2 | -20.0 | -9.39 | FALSE |
| SRX4815190 | TFs and others | CTNNB1 | Breast | SUM 149PT | 3365 | 1266/31605 | -0.08/0.26 | -20.2 | -20.0 | -9.39 | FALSE |
| SRX1161166 | TFs and others | NR3C1 | Breast | MCF-7 | 13553 | 777/31605 | -0.18/0.26 | -20.2 | -19.9 | -9.39 | FALSE |
| SRX2871359 | TFs and others | PGR | Breast | T-47D | 2027 | 543/31605 | -0.26/0.26 | -20.2 | -19.9 | -9.38 | FALSE |
| SRX3285608 | TFs and others | KDM5B | Breast | FULR | 5993 | 1457/31605 | -0.06/0.26 | -20.2 | -19.9 | -9.37 | FALSE |
| SRX14101941 | TFs and others | SNAI2 | Breast | MCF-7 | 8390 | 1950/31605 | -0.02/0.26 | -20.1 | -19.8 | -9.36 | FALSE |
| SRX9719182 | TFs and others | GATA3 | Breast | MCF-7 | 14336 | 1148/31605 | -0.10/0.26 | -20.0 | -19.7 | -9.34 | FALSE |
| SRX22740969 | TFs and others | DVL1 | Breast | MDA-MB-231 | 6186 | 1416/31605 | -0.06/0.26 | -20.0 | -19.7 | -9.33 | FALSE |
| SRX10205609 | TFs and others | YAP1 | Breast | CAL-120 | 12173 | 1977/31605 | -0.01/0.26 | -19.9 | -19.6 | -9.31 | FALSE |
| SRX5089428 | TFs and others | SMARCB1 | Breast | MCF-7 | 3081 | 298/31605 | -0.44/0.26 | -19.9 | -19.6 | -9.31 | FALSE |
| SRX11120875 | TFs and others | MAU2 | Breast | MCF-7 | 11074 | 1022/31605 | -0.12/0.26 | -19.9 | -19.6 | -9.31 | FALSE |
| SRX3322159 | TFs and others | ZBTB7B | Breast | MCF-7 | 3327 | 771/31605 | -0.17/0.26 | -19.8 | -19.6 | -9.29 | FALSE |
| SRX6860639 | TFs and others | RNF2 | Breast | T-47D | 1458 | 34/31605 | -1.78/0.26 | -19.8 | -19.5 | -9.29 | FALSE |
| SRX1161165 | TFs and others | NR3C1 | Breast | MCF-7 | 1667 | 540/31605 | -0.26/0.26 | -19.8 | -19.5 | -9.28 | FALSE |
| SRX176866 | TFs and others | GATA3 | Breast | MCF-7 | 31632 | 2156/31605 | -0.00/0.26 | -19.8 | -19.5 | -9.28 | FALSE |
| SRX6487049 | TFs and others | FOXA1 | Breast | MCF-7 | 5970 | 479/31605 | -0.29/0.26 | -19.8 | -19.5 | -9.28 | FALSE |
| SRX2578895 | TFs and others | NCOA1 | Breast | MDA-MB-231 | 1605 | 513/31605 | -0.27/0.26 | -19.8 | -19.5 | -9.28 | FALSE |
| SRX5089424 | TFs and others | SMARCA4 | Breast | MCF-7 | 12243 | 1671/31605 | -0.04/0.26 | -19.8 | -19.5 | -9.28 | FALSE |
| SRX5287694 | TFs and others | YAP1 | Breast | T-47D | 12120 | 1568/31605 | -0.04/0.26 | -19.7 | -19.5 | -9.27 | FALSE |
| SRX19888708 | TFs and others | NELFE | Breast | MCF-7 | 3082 | 1345/31605 | -0.07/0.26 | -19.7 | -19.4 | -9.26 | FALSE |
| SRX9688335 | TFs and others | ESR1 | Breast | ZR-75-1 | 1103 | 20/31605 | -2.40/0.26 | -19.7 | -19.4 | -9.26 | FALSE |
| SRX4115995 | TFs and others | FOXA1 | Breast | MCF-7 | 16001 | 1400/31605 | -0.06/0.26 | -19.7 | -19.4 | -9.26 | FALSE |
| SRX9719181 | TFs and others | GATA3 | Breast | MCF-7 | 10103 | 936/31605 | -0.13/0.26 | -19.7 | -19.4 | -9.25 | FALSE |
| SRX10365550 | TFs and others | NCOA3 | Breast | MCF-7 | 11869 | 615/31605 | -0.22/0.26 | -19.6 | -19.4 | -9.24 | FALSE |
| SRX19024880 | TFs and others | NR3C1 | Breast | MCF-7 | 22649 | 1481/31605 | -0.05/0.26 | -19.6 | -19.3 | -9.24 | FALSE |
| SRX22740970 | TFs and others | DVL1 | Breast | MDA-MB-231 | 6080 | 1379/31605 | -0.06/0.26 | -19.6 | -19.3 | -9.24 | FALSE |
| SRX3322868 | TFs and others | BMI1 | Breast | MCF-7 | 5771 | 1811/31605 | -0.02/0.26 | -19.5 | -19.3 | -9.22 | FALSE |
| SRX11647485 | TFs and others | ESR1 | Breast | MCF-7 | 416 | 99/31605 | -0.93/0.26 | -19.5 | -19.2 | -9.21 | FALSE |
| SRX18191648 | TFs and others | ESR1 | Breast | MCF-7 | 1703 | 195/31605 | -0.59/0.26 | -19.5 | -19.2 | -9.20 | FALSE |
| SRX9683971 | TFs and others | ESRRA | Breast | MDA-MB-231 | 5792 | 1384/31605 | -0.06/0.26 | -19.4 | -19.2 | -9.20 | FALSE |
| SRX14756932 | TFs and others | DNA-RNA hybrid | Breast | MDA-MB-436 | 6005 | 2750/31605 | 0.03/0.26 | -19.4 | -19.2 | -9.20 | FALSE |
| SRX667405 | TFs and others | Epitope tags | Breast | MCF-7 | 1802 | 1190/31605 | -0.09/0.26 | -19.4 | -19.1 | -9.18 | FALSE |
| SRX2378690 | TFs and others | AHR | Breast | MCF-7 | 3679 | 139/31605 | -0.74/0.26 | -19.4 | -19.1 | -9.18 | FALSE |
| SRX4792903 | TFs and others | CTCF | Breast | T-47D | 17497 | 5151/31605 | 0.09/0.26 | -19.3 | -19.0 | -9.17 | FALSE |
| SRX10119373 | TFs and others | TEAD1 | Breast | MCF 10A | 9395 | 1543/31605 | -0.04/0.26 | -19.3 | -19.0 | -9.16 | FALSE |
| SRX1165105 | TFs and others | RAD21 | Breast | MCF-7 | 43126 | 4975/31605 | 0.09/0.26 | -19.2 | -18.9 | -9.14 | FALSE |
| SRX5203308 | TFs and others | CTCF | Breast | MCF-7 | 23600 | 3142/31605 | 0.05/0.26 | -19.2 | -18.9 | -9.14 | FALSE |
| SRX4451205 | TFs and others | ESR1 | Breast | MCF-7 | 578 | 17/31605 | -2.58/0.26 | -19.1 | -18.8 | -9.12 | FALSE |
| SRX5534872 | TFs and others | JUN | Breast | MCF-7 | 10453 | 2580/31605 | 0.03/0.26 | -19.1 | -18.8 | -9.11 | FALSE |
| SRX3554409 | TFs and others | CREB1 | Breast | MDA-MB-134-VI | 1616 | 501/31605 | -0.27/0.26 | -19.1 | -18.8 | -9.11 | FALSE |
| SRX13845689 | TFs and others | HSF1 | Breast | MDA-MB-231 | 2700 | 1245/31605 | -0.08/0.26 | -19.0 | -18.8 | -9.10 | FALSE |
| SRX10119389 | TFs and others | WWTR1 | Breast | MDA-MB-231 | 19931 | 2585/31605 | 0.03/0.26 | -19.0 | -18.7 | -9.08 | FALSE |
| SRX10365549 | TFs and others | NCOA3 | Breast | MCF-7 | 3041 | 153/31605 | -0.68/0.26 | -19.0 | -18.7 | -9.08 | FALSE |
| SRX5824551 | TFs and others | BRD7 | Breast | SUM 159PT | 9990 | 3036/31605 | 0.04/0.26 | -19.0 | -18.7 | -9.08 | FALSE |
| SRX6712619 | TFs and others | SMC1A | Breast | MCF-7 | 43434 | 5547/31605 | 0.10/0.26 | -18.9 | -18.7 | -9.07 | FALSE |
| SRX673726 | TFs and others | Biotin | Breast | MCF-7 | 2218 | 114/31605 | -0.83/0.26 | -18.9 | -18.6 | -9.06 | FALSE |
| SRX15216103 | TFs and others | MEN1 | Breast | MCF-7 | 929 | 103/31605 | -0.89/0.26 | -18.9 | -18.6 | -9.06 | FALSE |
| SRX5493762 | TFs and others | FOXA1 | Breast | Breast cancer cells | 7343 | 858/31605 | -0.14/0.26 | -18.9 | -18.6 | -9.06 | FALSE |
| SRX4115994 | TFs and others | FOXA1 | Breast | MCF-7 | 10559 | 921/31605 | -0.13/0.26 | -18.8 | -18.5 | -9.04 | FALSE |
| SRX14267235 | TFs and others | MYC | Breast | MCF 10A | 1645 | 733/31605 | -0.17/0.26 | -18.8 | -18.5 | -9.03 | FALSE |
| SRX6058762 | TFs and others | PGR | Breast | T-47D | 5746 | 702/31605 | -0.18/0.26 | -18.8 | -18.5 | -9.03 | FALSE |
| SRX1156522 | TFs and others | Epitope tags | Breast | MDA-MB-231 | 11244 | 1755/31605 | -0.02/0.26 | -18.7 | -18.5 | -9.02 | FALSE |
| SRX3546794 | TFs and others | Epitope tags | Breast | MDA-MB-231 | 625 | 92/31605 | -0.95/0.26 | -18.7 | -18.5 | -9.02 | FALSE |
| SRX469398 | TFs and others | ESR1 | Breast | MCF-7 | 428 | 48/31605 | -1.41/0.26 | -18.7 | -18.4 | -9.01 | FALSE |
| SRX14756935 | TFs and others | DNA-RNA hybrid | Breast | MDA-MB-436 | 3846 | 2575/31605 | 0.03/0.26 | -18.7 | -18.4 | -9.00 | FALSE |
| SRX9719183 | TFs and others | GATA3 | Breast | MCF-7 | 13657 | 1190/31605 | -0.08/0.26 | -18.6 | -18.4 | -9.00 | FALSE |
| SRX19210548 | TFs and others | RELA | Breast | MDA-MB-231 | 3919 | 1472/31605 | -0.05/0.26 | -18.6 | -18.4 | -9.00 | FALSE |
| SRX15430989 | TFs and others | TFE3 | Breast | MDA-MB-453 | 25475 | 1808/31605 | -0.02/0.26 | -18.6 | -18.4 | -9.00 | FALSE |
| SRX5511307 | TFs and others | ESR1 | Breast | MDA-MB-231 | 922 | 94/31605 | -0.93/0.26 | -18.6 | -18.3 | -8.99 | FALSE |
| SRX5323847 | TFs and others | FOXA1 | Breast | MCF-7 | 15056 | 1249/31605 | -0.07/0.26 | -18.5 | -18.3 | -8.98 | FALSE |
| SRX7130729 | TFs and others | CTCF | Breast | MCF 10A | 23558 | 3885/31605 | 0.07/0.26 | -18.5 | -18.3 | -8.97 | FALSE |
| SRX2924018 | TFs and others | TP53 | Breast | MCF-7 | 9757 | 1419/31605 | -0.05/0.26 | -18.5 | -18.3 | -8.97 | FALSE |
| SRX5089528 | TFs and others | SMARCA4 | Breast | MCF-7 | 3160 | 459/31605 | -0.28/0.26 | -18.5 | -18.2 | -8.96 | FALSE |
| SRX7692224 | TFs and others | ARID1B | Breast | MCF-7 | 665 | 46/31605 | -1.44/0.26 | -18.5 | -18.2 | -8.96 | FALSE |
| SRX4096861 | TFs and others | GATAD2A | Breast | HCC3153 | 2516 | 795/31605 | -0.15/0.26 | -18.5 | -18.2 | -8.96 | FALSE |
| SRX3800732 | TFs and others | RAD21 | Breast | T-47D | 6258 | 2565/31605 | 0.03/0.26 | -18.4 | -18.2 | -8.94 | FALSE |
| SRX13991444 | TFs and others | TCF4 | Breast | MCF 10A | 2455 | 952/31605 | -0.12/0.26 | -18.4 | -18.2 | -8.94 | FALSE |
| SRX15216105 | TFs and others | MEN1 | Breast | MCF-7 | 962 | 100/31605 | -0.89/0.26 | -18.4 | -18.1 | -8.94 | FALSE |
| SRX5203310 | TFs and others | ESR1 | Breast | MCF-7 | 784 | 19/31605 | -2.37/0.26 | -18.4 | -18.1 | -8.92 | FALSE |
| SRX10119364 | TFs and others | YAP1 | Breast | MCF 10A | 36861 | 3656/31605 | 0.07/0.26 | -18.3 | -18.1 | -8.92 | FALSE |
| SRX3229186 | TFs and others | NR3C1 | Breast | Breast cancer | 858 | 32/31605 | -1.76/0.26 | -18.3 | -18.1 | -8.92 | FALSE |
| SRX7262858 | TFs and others | AR | Breast | Breast cancer cells | 14460 | 1122/31605 | -0.09/0.26 | -18.3 | -18.1 | -8.92 | FALSE |
| SRX673725 | TFs and others | Biotin | Breast | MCF-7 | 3149 | 189/31605 | -0.58/0.26 | -18.3 | -18.0 | -8.91 | FALSE |
| SRX1531795 | TFs and others | SMC1A | Breast | MCF-7 | 38575 | 4686/31605 | 0.09/0.26 | -18.3 | -18.0 | -8.91 | FALSE |
| SRX6098148 | TFs and others | GATA3 | Breast | MCF-7 | 24339 | 1794/31605 | -0.01/0.26 | -18.3 | -18.0 | -8.91 | FALSE |
| SRX20282962 | TFs and others | AHR | Breast | MCF-7 | 5240 | 595/31605 | -0.21/0.26 | -18.3 | -18.0 | -8.91 | FALSE |
| SRX2578888 | TFs and others | MED1 | Breast | MDA-MB-231 | 853 | 169/31605 | -0.62/0.26 | -18.2 | -18.0 | -8.90 | FALSE |
| SRX10024482 | TFs and others | FOXA1 | Breast | MCF-7 | 15253 | 1351/31605 | -0.05/0.26 | -18.2 | -18.0 | -8.90 | FALSE |
| SRX3734321 | TFs and others | TP53 | Breast | Mammary glands | 12232 | 1946/31605 | -0.00/0.26 | -18.2 | -18.0 | -8.89 | FALSE |
| SRX25289346 | TFs and others | GRHL2 | Breast | MCF-7 | 3716 | 789/31605 | -0.15/0.26 | -18.2 | -17.9 | -8.89 | FALSE |
| SRX10024481 | TFs and others | FOXA1 | Breast | MCF-7 | 19111 | 1640/31605 | -0.03/0.26 | -18.2 | -17.9 | -8.89 | FALSE |
| SRX1585243 | TFs and others | ESR1 | Breast | MCF-7 | 13300 | 1464/31605 | -0.04/0.26 | -18.2 | -17.9 | -8.88 | FALSE |
| SRX7122005 | TFs and others | FOXA1 | Breast | MCF-7 | 6113 | 798/31605 | -0.15/0.26 | -18.2 | -17.9 | -8.87 | FALSE |
| SRX3323023 | TFs and others | ATF7 | Breast | MCF-7 | 5416 | 1092/31605 | -0.09/0.26 | -18.1 | -17.9 | -8.87 | FALSE |
| SRX8537751 | TFs and others | GATA3 | Breast | T-47D | 8008 | 665/31605 | -0.19/0.26 | -18.1 | -17.9 | -8.87 | FALSE |
| SRX5457521 | TFs and others | ZNF574 | Breast | MCF-7 | 4092 | 827/31605 | -0.14/0.26 | -18.1 | -17.9 | -8.87 | FALSE |
| SRX3923510 | TFs and others | FOXA1 | Breast | MCF-7 | 28612 | 2353/31605 | 0.02/0.26 | -18.1 | -17.8 | -8.85 | FALSE |
| SRX3734319 | TFs and others | TP63 | Breast | Mammary glands | 45127 | 4293/31605 | 0.08/0.26 | -18.0 | -17.8 | -8.84 | FALSE |
| SRX323402 | TFs and others | SPDEF | Breast | MCF-7 | 862 | 320/31605 | -0.38/0.26 | -18.0 | -17.7 | -8.83 | FALSE |
| SRX6098144 | TFs and others | TRPS1 | Breast | MCF-7 | 7463 | 602/31605 | -0.21/0.26 | -18.0 | -17.7 | -8.83 | FALSE |
| SRX3410404 | TFs and others | ESR1 | Breast | T-47D | 2382 | 92/31605 | -0.92/0.26 | -18.0 | -17.7 | -8.82 | FALSE |
| SRX060812 | TFs and others | EP300 | Breast | MCF-7 | 748 | 51/31605 | -1.33/0.26 | -17.9 | -17.7 | -8.82 | FALSE |
| SRX16646115 | TFs and others | HSF1 | Breast | MCF-7 | 9143 | 1587/31605 | -0.03/0.26 | -17.9 | -17.6 | -8.81 | FALSE |
| SRX1012705 | TFs and others | ESR1 | Breast | T-47D | 1794 | 78/31605 | -1.02/0.26 | -17.9 | -17.6 | -8.81 | FALSE |
| SRX19210524 | TFs and others | RELB | Breast | SUM 149PT | 2143 | 837/31605 | -0.13/0.26 | -17.9 | -17.6 | -8.80 | FALSE |
| SRX673724 | TFs and others | Biotin | Breast | MCF-7 | 4719 | 540/31605 | -0.23/0.26 | -17.8 | -17.6 | -8.79 | FALSE |
| SRX10142205 | TFs and others | CTCF | Breast | Breast cancer cells | 11732 | 2619/31605 | 0.04/0.26 | -17.8 | -17.6 | -8.79 | FALSE |
| SRX3229167 | TFs and others | AR | Breast | Breast cancer | 1548 | 104/31605 | -0.85/0.26 | -17.8 | -17.5 | -8.78 | FALSE |
| SRX2871353 | TFs and others | GATA3 | Breast | T-47D | 6590 | 709/31605 | -0.17/0.26 | -17.8 | -17.5 | -8.77 | FALSE |
| SRX5089527 | TFs and others | SMARCA4 | Breast | MCF-7 | 6060 | 1987/31605 | 0.00/0.26 | -17.8 | -17.5 | -8.77 | FALSE |
| SRX3229124 | TFs and others | FOXA1 | Breast | Breast cancer | 19573 | 1813/31605 | -0.01/0.26 | -17.8 | -17.5 | -8.77 | FALSE |
| SRX6098146 | TFs and others | GATA3 | Breast | MCF-7 | 26151 | 1264/31605 | -0.06/0.26 | -17.7 | -17.4 | -8.76 | FALSE |
| SRX4192884 | TFs and others | JUN | Breast | MCF 10A | 46128 | 4072/31605 | 0.08/0.26 | -17.6 | -17.4 | -8.74 | FALSE |
| SRX150670 | TFs and others | STAT3 | Breast | MCF 10A | 12710 | 1911/31605 | -0.00/0.26 | -17.6 | -17.4 | -8.74 | FALSE |
| SRX5323859 | TFs and others | STAT3 | Breast | MCF-7 | 1440 | 408/31605 | -0.30/0.26 | -17.6 | -17.3 | -8.73 | FALSE |
| SRX384488 | TFs and others | XBP1 | Breast | MDA-MB-231 | 4857 | 990/31605 | -0.10/0.26 | -17.6 | -17.3 | -8.73 | FALSE |
| SRX3229193 | TFs and others | GATA3 | Breast | Breast cancer | 1366 | 77/31605 | -1.02/0.26 | -17.6 | -17.3 | -8.72 | FALSE |
| SRX9683970 | TFs and others | ESRRA | Breast | MDA-MB-231 | 6461 | 1484/31605 | -0.03/0.26 | -17.5 | -17.2 | -8.70 | FALSE |
| SRX747793 | TFs and others | FOXA1 | Breast | MCF-7 | 11466 | 944/31605 | -0.11/0.26 | -17.4 | -17.2 | -8.69 | FALSE |
| SRX3322160 | TFs and others | ZBTB7B | Breast | MCF-7 | 2282 | 527/31605 | -0.23/0.26 | -17.4 | -17.2 | -8.68 | FALSE |
| SRX10164613 | TFs and others | ESR1 | Breast | MCF-7 | 1802 | 28/31605 | -1.84/0.26 | -17.4 | -17.1 | -8.68 | FALSE |
| SRX3080210 | TFs and others | JUN | Breast | MCF-7 | 10948 | 1086/31605 | -0.08/0.26 | -17.4 | -17.1 | -8.67 | FALSE |
| SRX2897215 | TFs and others | ESR1 | Breast | Breast cancer cells | 1680 | 1400/31605 | -0.04/0.26 | -17.3 | -17.1 | -8.66 | FALSE |
| SRX10119371 | TFs and others | TEAD1 | Breast | MCF 10A | 12599 | 1712/31605 | -0.01/0.26 | -17.3 | -17.1 | -8.66 | FALSE |
| SRX4116036 | TFs and others | FOXA1 | Breast | Breast cancer cells | 19143 | 1809/31605 | -0.01/0.26 | -17.3 | -17.1 | -8.66 | FALSE |
| SRX3040147 | TFs and others | STAG1 | Breast | HMEC | 41356 | 5419/31605 | 0.10/0.26 | -17.3 | -17.1 | -8.66 | FALSE |
| SRX4192888 | TFs and others | STAT3 | Breast | MCF 10A | 32390 | 3476/31605 | 0.07/0.26 | -17.3 | -17.0 | -8.65 | FALSE |
| SRX15826242 | TFs and others | KDM6A | Breast | MDA-MB-231 | 18536 | 3370/31605 | 0.06/0.26 | -17.2 | -17.0 | -8.64 | FALSE |
| SRX10205602 | TFs and others | YAP1 | Breast | CAL-120 | 12223 | 1977/31605 | 0.01/0.26 | -17.2 | -17.0 | -8.63 | FALSE |
| SRX15216104 | TFs and others | MEN1 | Breast | MCF-7 | 937 | 70/31605 | -1.06/0.26 | -17.2 | -16.9 | -8.62 | FALSE |
| SRX9063420 | TFs and others | JUN | Breast | MCF-7 | 9058 | 1936/31605 | 0.00/0.26 | -17.2 | -16.9 | -8.62 | FALSE |
| SRX5534860 | TFs and others | ARID1B | Breast | MCF-7 | 1043 | 630/31605 | -0.18/0.26 | -17.2 | -16.9 | -8.61 | FALSE |
| SRX3229181 | TFs and others | FOXA1 | Breast | Breast cancer | 7696 | 1042/31605 | -0.09/0.26 | -17.1 | -16.9 | -8.61 | FALSE |
| SRX6487051 | TFs and others | FOXA1 | Breast | MCF-7 | 11671 | 883/31605 | -0.12/0.26 | -17.1 | -16.8 | -8.60 | FALSE |
| SRX6487039 | TFs and others | FOXA1 | Breast | MCF-7 | 8129 | 671/31605 | -0.17/0.26 | -17.1 | -16.8 | -8.59 | FALSE |
| SRX5089525 | TFs and others | SMARCA4 | Breast | MCF-7 | 5659 | 1603/31605 | -0.02/0.26 | -17.1 | -16.8 | -8.59 | FALSE |
| SRX2194236 | TFs and others | MED1 | Breast | SUM 159PT | 5590 | 1422/31605 | -0.04/0.26 | -17.0 | -16.8 | -8.58 | FALSE |
| SRX5128105 | TFs and others | ESR1 | Breast | ZR-75-1 | 3189 | 149/31605 | -0.64/0.26 | -17.0 | -16.8 | -8.58 | FALSE |
| SRX730437 | TFs and others | NCAPG | Breast | MCF-7 | 1992 | 928/31605 | -0.11/0.26 | -17.0 | -16.8 | -8.58 | FALSE |
| SRX5203292 | TFs and others | CTCF | Breast | MCF-7 | 20808 | 2813/31605 | 0.05/0.26 | -17.0 | -16.8 | -8.58 | FALSE |
| SRX6853577 | TFs and others | ARNT | Breast | MCF-7 | 859 | 222/31605 | -0.48/0.26 | -17.0 | -16.7 | -8.57 | FALSE |
| SRX8246296 | TFs and others | FGFR1 | Breast | MCF-7 | 1534 | 676/31605 | -0.17/0.26 | -17.0 | -16.7 | -8.57 | FALSE |
| SRX6027747 | TFs and others | FOXA1 | Breast | MCF-7 | 8397 | 771/31605 | -0.14/0.26 | -17.0 | -16.7 | -8.56 | FALSE |
| SRX23740441 | TFs and others | KDM5B | Breast | SUM 149PT | 8459 | 2884/31605 | 0.05/0.26 | -17.0 | -16.7 | -8.56 | FALSE |
| SRX21286682 | TFs and others | TAZ | Breast | MCF 10A | 777 | 506/31605 | -0.23/0.26 | -16.9 | -16.7 | -8.56 | FALSE |
| SRX14875682 | TFs and others | SET | Breast | MCF-7 | 1155 | 38/31605 | -1.52/0.26 | -16.9 | -16.6 | -8.54 | FALSE |
| SRX5089496 | TFs and others | HDAC1 | Breast | MCF-7 | 683 | 261/31605 | -0.42/0.26 | -16.8 | -16.6 | -8.53 | FALSE |
| SRX3322636 | TFs and others | FOS | Breast | MCF-7 | 40951 | 3587/31605 | 0.07/0.26 | -16.8 | -16.5 | -8.52 | FALSE |
| SRX5057631 | TFs and others | GATA3 | Breast | T-47D | 8970 | 933/31605 | -0.10/0.26 | -16.8 | -16.5 | -8.51 | FALSE |
| SRX8520802 | TFs and others | NR3C1 | Breast | HCC1187 | 44505 | 3871/31605 | 0.08/0.26 | -16.8 | -16.5 | -8.51 | FALSE |
| SRX20282943 | TFs and others | AHR | Breast | MCF-7 | 2019 | 183/31605 | -0.55/0.26 | -16.7 | -16.5 | -8.50 | FALSE |
| SRX384491 | TFs and others | XBP1 | Breast | Hs 578T | 4263 | 886/31605 | -0.11/0.26 | -16.7 | -16.5 | -8.50 | FALSE |
| SRX5089420 | TFs and others | SMARCA4 | Breast | MCF-7 | 13975 | 1927/31605 | 0.01/0.26 | -16.7 | -16.5 | -8.50 | FALSE |
| SRX2109219 | TFs and others | MYC | Breast | Mammary epithelial cells | 1774 | 708/31605 | -0.15/0.26 | -16.7 | -16.4 | -8.48 | FALSE |
| SRX673723 | TFs and others | GATA3 | Breast | MCF-7 | 2758 | 281/31605 | -0.39/0.26 | -16.7 | -16.4 | -8.48 | FALSE |
| SRX19210547 | TFs and others | RELA | Breast | MDA-MB-231 | 4287 | 1477/31605 | -0.03/0.26 | -16.6 | -16.4 | -8.47 | FALSE |
| SRX8537752 | TFs and others | GATA3 | Breast | T-47D | 9504 | 811/31605 | -0.13/0.26 | -16.6 | -16.4 | -8.47 | FALSE |
| SRX6487045 | TFs and others | FOXA1 | Breast | MCF-7 | 10522 | 826/31605 | -0.12/0.26 | -16.6 | -16.4 | -8.47 | FALSE |
| SRX16646116 | TFs and others | HSF1 | Breast | MCF-7 | 11062 | 1806/31605 | -0.00/0.26 | -16.6 | -16.3 | -8.47 | FALSE |
| SRX15826241 | TFs and others | KMT2D | Breast | MDA-MB-231 | 17612 | 2466/31605 | 0.04/0.26 | -16.6 | -16.3 | -8.46 | FALSE |
| SRX6487044 | TFs and others | FOXA1 | Breast | MCF-7 | 7888 | 594/31605 | -0.19/0.26 | -16.6 | -16.3 | -8.46 | FALSE |
| SRX18013268 | TFs and others | CTCF | Breast | MCF 10A | 25517 | 3869/31605 | 0.08/0.26 | -16.5 | -16.3 | -8.45 | FALSE |
| SRX11374764 | TFs and others | PGR | Breast | T-47D | 10704 | 935/31605 | -0.10/0.26 | -16.5 | -16.3 | -8.45 | FALSE |
| SRX10119390 | TFs and others | WWTR1 | Breast | MDA-MB-231 | 21164 | 2404/31605 | 0.03/0.26 | -16.5 | -16.3 | -8.44 | FALSE |
| SRX4096891 | TFs and others | TRPS1 | Breast | SUM 159PT | 15771 | 1409/31605 | -0.03/0.26 | -16.5 | -16.2 | -8.43 | FALSE |
| SRX9109111 | TFs and others | AR | Breast | MDA-MB-453 | 10230 | 882/31605 | -0.11/0.26 | -16.4 | -16.2 | -8.43 | FALSE |
| SRX3322869 | TFs and others | BMI1 | Breast | MCF-7 | 4550 | 1507/31605 | -0.02/0.26 | -16.4 | -16.2 | -8.42 | FALSE |
| SRX3923508 | TFs and others | FOXA1 | Breast | MCF-7 | 25651 | 2222/31605 | 0.03/0.26 | -16.4 | -16.2 | -8.42 | FALSE |
| SRX1787678 | TFs and others | KMT2C | Breast | MCF-7 | 4976 | 401/31605 | -0.28/0.26 | -16.4 | -16.2 | -8.42 | FALSE |
| SRX2871350 | TFs and others | GATA3 | Breast | T-47D | 4708 | 478/31605 | -0.24/0.26 | -16.4 | -16.1 | -8.41 | FALSE |
| SRX7122001 | TFs and others | FOXA1 | Breast | MCF-7 | 6584 | 712/31605 | -0.15/0.26 | -16.4 | -16.1 | -8.41 | FALSE |
| SRX143299 | TFs and others | SNAPC1 | Breast | MCF 10A | 1205 | 674/31605 | -0.16/0.26 | -16.3 | -16.0 | -8.38 | FALSE |
| SRX1012688 | TFs and others | ESR1 | Breast | MCF-7 | 565 | 29/31605 | -1.74/0.26 | -16.3 | -16.0 | -8.38 | FALSE |
| SRX5251430 | TFs and others | ESR1 | Breast | T-47D | 1026 | 49/31605 | -1.28/0.26 | -16.2 | -16.0 | -8.37 | FALSE |
| SRX1156523 | TFs and others | Epitope tags | Breast | MDA-MB-231 | 11219 | 1738/31605 | -0.00/0.26 | -16.2 | -16.0 | -8.37 | FALSE |
| SRX883576 | TFs and others | YAP1 | Breast | MDA-MB-231 | 9154 | 1354/31605 | -0.04/0.26 | -16.2 | -16.0 | -8.37 | FALSE |
| SRX3410402 | TFs and others | PCGF2 | Breast | T-47D | 7734 | 1612/31605 | -0.01/0.26 | -16.2 | -16.0 | -8.37 | FALSE |
| SRX5511302 | TFs and others | ESR1 | Breast | MDA-MB-231 | 701 | 101/31605 | -0.81/0.26 | -16.2 | -15.9 | -8.35 | FALSE |
| SRX8850950 | TFs and others | Epitope tags | Breast | MCF 10A | 1346 | 440/31605 | -0.25/0.26 | -16.1 | -15.9 | -8.34 | FALSE |
| SRX3229179 | TFs and others | FOXA1 | Breast | Breast cancer | 7849 | 926/31605 | -0.10/0.26 | -16.1 | -15.9 | -8.34 | FALSE |
| SRX038563 | TFs and others | CTCF | Breast | HMEC | 21885 | 2963/31605 | 0.06/0.26 | -16.1 | -15.8 | -8.32 | FALSE |
| SRX039145 | TFs and others | FOXA1 | Breast | T-47D | 4041 | 304/31605 | -0.36/0.26 | -16.1 | -15.8 | -8.32 | FALSE |
| SRX14101940 | TFs and others | SNAI2 | Breast | MCF-7 | 4027 | 1688/31605 | -0.00/0.26 | -16.1 | -15.8 | -8.32 | FALSE |
| SRX2020848 | TFs and others | STAT3 | Breast | HCC1143 | 13969 | 1872/31605 | 0.01/0.26 | -16.0 | -15.8 | -8.32 | FALSE |
| SRX8083086 | TFs and others | FGFR1 | Breast | CAMA-1 | 1789 | 572/31605 | -0.19/0.26 | -16.0 | -15.8 | -8.31 | FALSE |
| SRX23740440 | TFs and others | KDM5B | Breast | SUM 149PT | 8741 | 3028/31605 | 0.06/0.26 | -16.0 | -15.8 | -8.31 | FALSE |
| SRX4116028 | TFs and others | FOXA1 | Breast | Breast cancer cells | 9422 | 1003/31605 | -0.08/0.26 | -16.0 | -15.7 | -8.30 | FALSE |
| SRX7262874 | TFs and others | EP300 | Breast | ZR-75-1 | 17815 | 1118/31605 | -0.06/0.26 | -16.0 | -15.7 | -8.30 | FALSE |
| SRX3229166 | TFs and others | AR | Breast | Breast cancer | 926 | 77/31605 | -0.96/0.26 | -16.0 | -15.7 | -8.30 | FALSE |
| SRX3229123 | TFs and others | FOXA1 | Breast | Breast cancer | 9744 | 984/31605 | -0.08/0.26 | -16.0 | -15.7 | -8.29 | FALSE |
| SRX20282942 | TFs and others | AHR | Breast | MCF-7 | 1594 | 118/31605 | -0.72/0.26 | -15.9 | -15.7 | -8.29 | FALSE |
| SRX673755 | TFs and others | FOXA1 | Breast | MCF-7 | 9337 | 1127/31605 | -0.06/0.26 | -15.9 | -15.7 | -8.29 | FALSE |
| SRX6487058 | TFs and others | FOXA1 | Breast | MCF-7 | 7662 | 619/31605 | -0.17/0.26 | -15.8 | -15.6 | -8.26 | FALSE |
| SRX3990976 | TFs and others | PGR | Breast | T-47D | 1784 | 432/31605 | -0.25/0.26 | -15.8 | -15.6 | -8.26 | FALSE |
| SRX9697818 | TFs and others | Epitope tags | Breast | MDA-MB-231 | 5024 | 1005/31605 | -0.08/0.26 | -15.8 | -15.6 | -8.25 | FALSE |
| SRX4671310 | TFs and others | RXRA | Breast | SUM 159PT | 7505 | 1233/31605 | -0.05/0.26 | -15.8 | -15.6 | -8.25 | FALSE |
| SRX7130725 | TFs and others | CTCF | Breast | MCF 10A | 16170 | 2973/31605 | 0.06/0.26 | -15.8 | -15.5 | -8.24 | FALSE |
| SRX4284791 | TFs and others | Epitope tags | Breast | T-47D | 936 | 27/31605 | -1.77/0.26 | -15.7 | -15.5 | -8.23 | FALSE |
| SRX1489434 | TFs and others | MBD3 | Breast | MDA-MB-231 | 7561 | 1200/31605 | -0.05/0.26 | -15.7 | -15.4 | -8.21 | FALSE |
| SRX10775045 | TFs and others | RNF2 | Breast | T-47D | 1452 | 178/31605 | -0.53/0.26 | -15.7 | -15.4 | -8.21 | FALSE |
| SRX11867609 | TFs and others | KMT2C | Breast | CAL-51 | 2536 | 782/31605 | -0.12/0.26 | -15.5 | -15.3 | -8.18 | FALSE |
| SRX5359048 | TFs and others | SREBF2 | Breast | HCC70 | 32907 | 10680/31605 | 0.15/0.26 | -15.5 | -15.2 | -8.16 | FALSE |
| SRX3322312 | TFs and others | ZNF24 | Breast | MCF-7 | 4674 | 658/31605 | -0.15/0.26 | -15.4 | -15.2 | -8.15 | FALSE |
| SRX4451204 | TFs and others | ESR1 | Breast | MCF-7 | 549 | 19/31605 | -2.14/0.26 | -15.4 | -15.1 | -8.14 | FALSE |
| SRX15235717 | TFs and others | PRRX1 | Breast | MDA-MB-157 | 5642 | 1145/31605 | -0.05/0.26 | -15.4 | -15.1 | -8.13 | FALSE |
| SRX2871352 | TFs and others | GATA3 | Breast | T-47D | 4119 | 411/31605 | -0.26/0.26 | -15.4 | -15.1 | -8.13 | FALSE |
| SRX10142201 | TFs and others | CTCF | Breast | Breast luminal cells | 3544 | 935/31605 | -0.09/0.26 | -15.4 | -15.1 | -8.13 | FALSE |
| SRX7644279 | TFs and others | WTAP | Breast | MCF-7 | 477 | 148/31605 | -0.60/0.26 | -15.3 | -15.1 | -8.12 | FALSE |
| SRX194580 | TFs and others | RXRA | Breast | MCF-7 | 1788 | 227/31605 | -0.43/0.26 | -15.3 | -15.1 | -8.12 | FALSE |
| SRX747792 | TFs and others | FOXA1 | Breast | MCF-7 | 13869 | 1518/31605 | -0.01/0.26 | -15.3 | -15.1 | -8.12 | FALSE |
| SRX18013280 | TFs and others | TEAD4 | Breast | FULVR | 12570 | 1522/31605 | -0.01/0.26 | -15.3 | -15.1 | -8.12 | FALSE |
| SRX3923525 | TFs and others | FOXA1 | Breast | ZR-75-1 | 29995 | 2399/31605 | 0.04/0.26 | -15.3 | -15.1 | -8.11 | FALSE |
| SRX3583255 | TFs and others | HIF1A | Breast | MDA-MB-231 | 1500 | 294/31605 | -0.35/0.26 | -15.3 | -15.1 | -8.11 | FALSE |
| SRX18013265 | TFs and others | STAG1 | Breast | MCF 10A | 14915 | 2734/31605 | 0.06/0.26 | -15.3 | -15.0 | -8.10 | FALSE |
| SRX7865933 | TFs and others | BRD4 | Breast | MDA-MB-231 | 7729 | 1250/31605 | -0.04/0.26 | -15.3 | -15.0 | -8.10 | FALSE |
| SRX25289347 | TFs and others | GRHL2 | Breast | MCF-7 | 3258 | 633/31605 | -0.16/0.26 | -15.2 | -15.0 | -8.08 | FALSE |
| SRX1156520 | TFs and others | Epitope tags | Breast | MDA-MB-231 | 12558 | 1759/31605 | 0.01/0.26 | -15.2 | -15.0 | -8.08 | FALSE |
| SRX4192880 | TFs and others | FOS | Breast | MCF 10A | 80862 | 5775/31605 | 0.12/0.26 | -15.2 | -14.9 | -8.08 | FALSE |
| SRX4815189 | TFs and others | CTNNB1 | Breast | SUM 149PT | 3340 | 1153/31605 | -0.05/0.26 | -15.1 | -14.8 | -8.05 | FALSE |
| SRX4815194 | TFs and others | TLE3 | Breast | SUM 149PT | 38136 | 4131/31605 | 0.09/0.26 | -15.1 | -14.8 | -8.05 | FALSE |
| SRX667408 | TFs and others | Epitope tags | Breast | MCF-7 | 2962 | 1488/31605 | -0.01/0.26 | -15.1 | -14.8 | -8.05 | FALSE |
| SRX4096865 | TFs and others | GATAD2B | Breast | HCC3153 | 2285 | 491/31605 | -0.21/0.26 | -15.0 | -14.8 | -8.04 | FALSE |
| SRX734308 | TFs and others | MYBL2 | Breast | HMEC | 564 | 57/31605 | -1.11/0.26 | -15.0 | -14.8 | -8.04 | FALSE |
| SRX10119391 | TFs and others | TEAD1 | Breast | MDA-MB-231 | 8065 | 1300/31605 | -0.03/0.26 | -15.0 | -14.8 | -8.03 | FALSE |
| SRX185916 | TFs and others | FOXM1 | Breast | MCF-7 | 207 | 34/31605 | -1.51/0.26 | -15.0 | -14.8 | -8.03 | FALSE |
| SRX3322339 | TFs and others | SMARCE1 | Breast | MCF-7 | 7760 | 691/31605 | -0.14/0.26 | -15.0 | -14.7 | -8.01 | FALSE |
| SRX13327983 | TFs and others | XRCC6 | Breast | MCF-7 | 1589 | 597/31605 | -0.16/0.26 | -14.9 | -14.7 | -8.00 | FALSE |
| SRX12813812 | TFs and others | HEXIM1 | Breast | MCF-7 | 2547 | 321/31605 | -0.32/0.26 | -14.9 | -14.7 | -8.00 | FALSE |
| SRX3322086 | TFs and others | FOXA1 | Breast | MCF-7 | 17087 | 1430/31605 | -0.02/0.26 | -14.9 | -14.6 | -8.00 | FALSE |
| SRX330072 | TFs and others | Epitope tags | Breast | MCF-7 | 2701 | 393/31605 | -0.26/0.26 | -14.9 | -14.6 | -7.99 | FALSE |
| SRX3322288 | TFs and others | DDX20 | Breast | MCF-7 | 1906 | 453/31605 | -0.23/0.26 | -14.8 | -14.6 | -7.98 | FALSE |
| SRX667404 | TFs and others | Epitope tags | Breast | MCF-7 | 1702 | 553/31605 | -0.18/0.26 | -14.8 | -14.6 | -7.98 | FALSE |
| SRX1165107 | TFs and others | RAD21 | Breast | MCF-7 | 35073 | 4129/31605 | 0.10/0.26 | -14.8 | -14.6 | -7.98 | FALSE |
| SRX13327960 | TFs and others | CBX3 | Breast | MCF-7 | 2482 | 634/31605 | -0.15/0.26 | -14.8 | -14.5 | -7.96 | FALSE |
| SRX10119377 | TFs and others | WWTR1 | Breast | MCF 10A | 22220 | 2524/31605 | 0.05/0.26 | -14.7 | -14.5 | -7.95 | FALSE |
| SRX18191677 | TFs and others | ESR1 | Breast | MCF-7 | 1034 | 31/31605 | -1.57/0.26 | -14.7 | -14.5 | -7.94 | FALSE |
| SRX14267236 | TFs and others | MYC | Breast | MCF 10A | 1237 | 547/31605 | -0.18/0.26 | -14.6 | -14.4 | -7.92 | FALSE |
| SRX5534841 | TFs and others | FOXA1 | Breast | MCF-7 | 9681 | 801/31605 | -0.10/0.26 | -14.6 | -14.3 | -7.91 | FALSE |
| SRX176870 | TFs and others | GATA3 | Breast | MCF-7 | 3026 | 124/31605 | -0.65/0.26 | -14.6 | -14.3 | -7.91 | FALSE |
| SRX1757997 | TFs and others | MAX | Breast | MDA-MB-468 | 868 | 523/31605 | -0.19/0.26 | -14.5 | -14.3 | -7.89 | FALSE |
| SRX1709988 | TFs and others | ESR1 | Breast | T-47D | 780 | 47/31605 | -1.22/0.26 | -14.5 | -14.3 | -7.89 | FALSE |
| SRX3229122 | TFs and others | ESR1 | Breast | Breast cancer | 804 | 70/31605 | -0.95/0.26 | -14.5 | -14.3 | -7.88 | FALSE |
| SRX5493749 | TFs and others | AR | Breast | Breast cancer cells | 9086 | 732/31605 | -0.12/0.26 | -14.5 | -14.3 | -7.88 | FALSE |
| SRX2109214 | TFs and others | MYC | Breast | Mammary epithelial cells | 1449 | 541/31605 | -0.18/0.26 | -14.5 | -14.2 | -7.88 | FALSE |
| SRX6058758 | TFs and others | CEBPA | Breast | T-47D | 4480 | 747/31605 | -0.11/0.26 | -14.4 | -14.2 | -7.87 | FALSE |
| SRX6860628 | TFs and others | ESR1 | Breast | T-47D | 1442 | 22/31605 | -1.89/0.26 | -14.4 | -14.2 | -7.87 | FALSE |
| SRX6487056 | TFs and others | FOXA1 | Breast | MCF-7 | 7151 | 570/31605 | -0.17/0.26 | -14.4 | -14.2 | -7.85 | FALSE |
| SRX5203305 | TFs and others | CTCF | Breast | MCF-7 | 18861 | 2430/31605 | 0.05/0.26 | -14.3 | -14.1 | -7.83 | FALSE |
| SRX7262876 | TFs and others | EP300 | Breast | ZR-75-1 | 17083 | 743/31605 | -0.11/0.26 | -14.3 | -14.1 | -7.83 | FALSE |
| SRX5089440 | TFs and others | SMARCB1 | Breast | MCF-7 | 8333 | 832/31605 | -0.09/0.26 | -14.3 | -14.1 | -7.83 | FALSE |
| SRX20830476 | TFs and others | PGR | Breast | T-47D | 829 | 33/31605 | -1.49/0.26 | -14.3 | -14.0 | -7.81 | FALSE |
| SRX5457431 | TFs and others | E4F1 | Breast | MCF-7 | 1839 | 426/31605 | -0.23/0.26 | -14.2 | -14.0 | -7.80 | FALSE |
| SRX1733714 | TFs and others | ESR1 | Breast | MCF-7 | 1037 | 65/31605 | -0.98/0.26 | -14.2 | -13.9 | -7.79 | FALSE |
| SRX747795 | TFs and others | FOXA1 | Breast | MCF-7 | 14440 | 1513/31605 | -0.00/0.26 | -14.2 | -13.9 | -7.79 | FALSE |
| SRX5089529 | TFs and others | SMARCA4 | Breast | MCF-7 | 3148 | 1061/31605 | -0.05/0.26 | -14.2 | -13.9 | -7.79 | FALSE |
| SRX5089436 | TFs and others | SMARCB1 | Breast | MCF-7 | 6243 | 624/31605 | -0.14/0.26 | -14.2 | -13.9 | -7.79 | FALSE |
| SRX4663589 | TFs and others | NCOA3 | Breast | MCF-7 | 26828 | 1637/31605 | 0.01/0.26 | -14.2 | -13.9 | -7.78 | FALSE |
| SRX10142200 | TFs and others | CTCF | Breast | Breast luminal cells | 2923 | 802/31605 | -0.10/0.26 | -14.1 | -13.9 | -7.78 | FALSE |
| SRX457213 | TFs and others | ESR1 | Breast | MCF-7 | 687 | 40/31605 | -1.32/0.26 | -14.1 | -13.9 | -7.78 | FALSE |
| SRX673714 | TFs and others | Biotin | Breast | MCF-7 | 365 | 14/31605 | -2.41/0.26 | -14.1 | -13.9 | -7.78 | FALSE |
| SRX8850949 | TFs and others | Epitope tags | Breast | MCF 10A | 1198 | 347/31605 | -0.28/0.26 | -14.1 | -13.9 | -7.77 | FALSE |
| SRX2578866 | TFs and others | ESR1 | Breast | MDA-MB-231 | 364 | 32/31605 | -1.50/0.26 | -14.1 | -13.9 | -7.77 | FALSE |
| SRX3229177 | TFs and others | FOXA1 | Breast | Breast cancer | 11263 | 972/31605 | -0.06/0.26 | -14.1 | -13.9 | -7.76 | FALSE |
| SRX747780 | TFs and others | ESR1 | Breast | MCF-7 | 853 | 55/31605 | -1.08/0.26 | -14.0 | -13.7 | -7.73 | FALSE |
| SRX3322003 | TFs and others | SNIP1 | Breast | MCF-7 | 1648 | 589/31605 | -0.15/0.26 | -13.9 | -13.7 | -7.72 | FALSE |
| SRX5323928 | TFs and others | STAT3 | Breast | Breast cancer cells | 4931 | 429/31605 | -0.22/0.26 | -13.9 | -13.7 | -7.72 | FALSE |
| SRX3229176 | TFs and others | FOXA1 | Breast | Breast cancer | 3192 | 454/31605 | -0.21/0.26 | -13.9 | -13.7 | -7.72 | FALSE |
| SRX3541109 | TFs and others | ESR1 | Breast | MCF-7 | 668 | 19/31605 | -2.01/0.26 | -13.9 | -13.7 | -7.71 | FALSE |
| SRX6487052 | TFs and others | FOXA1 | Breast | MCF-7 | 8127 | 617/31605 | -0.14/0.26 | -13.9 | -13.6 | -7.70 | FALSE |
| SRX5534844 | TFs and others | ESR1 | Breast | MCF-7 | 326 | 94/31605 | -0.76/0.26 | -13.8 | -13.6 | -7.69 | FALSE |
| SRX036564 | TFs and others | ESR1 | Breast | MCF-7 | 497 | 24/31605 | -1.75/0.26 | -13.8 | -13.6 | -7.69 | FALSE |
| SRX1012684 | TFs and others | ESR1 | Breast | MCF-7 | 1254 | 52/31605 | -1.11/0.26 | -13.8 | -13.6 | -7.68 | FALSE |
| SRX6860624 | TFs and others | ESR1 | Breast | T-47D | 1357 | 17/31605 | -2.13/0.26 | -13.8 | -13.6 | -7.68 | FALSE |
| SRX1531790 | TFs and others | SMC1A | Breast | MCF-7 | 33355 | 3703/31605 | 0.09/0.26 | -13.8 | -13.6 | -7.68 | FALSE |
| SRX3923509 | TFs and others | FOXA1 | Breast | MCF-7 | 23939 | 1939/31605 | 0.03/0.26 | -13.8 | -13.6 | -7.68 | FALSE |
| SRX5089520 | TFs and others | SMARCA4 | Breast | MCF-7 | 3326 | 1259/31605 | -0.02/0.26 | -13.8 | -13.6 | -7.68 | FALSE |
| SRX5089423 | TFs and others | SMARCA4 | Breast | MCF-7 | 9016 | 1255/31605 | -0.02/0.26 | -13.8 | -13.5 | -7.67 | FALSE |
| SRX5089439 | TFs and others | SMARCB1 | Breast | MCF-7 | 9223 | 1514/31605 | 0.00/0.26 | -13.7 | -13.5 | -7.66 | FALSE |
| SRX13254866 | TFs and others | SUZ12 | Breast | MCF-7 | 32299 | 3450/31605 | 0.09/0.26 | -13.7 | -13.5 | -7.65 | FALSE |
| SRX17024138 | TFs and others | PARP1 | Breast | MDA-MB-231 | 1165 | 610/31605 | -0.14/0.26 | -13.7 | -13.4 | -7.64 | FALSE |
| SRX747775 | TFs and others | ESR1 | Breast | MCF-7 | 738 | 28/31605 | -1.59/0.26 | -13.7 | -13.4 | -7.64 | FALSE |
| SRX5287714 | TFs and others | Biotin | Breast | MCF-7 | 567 | 43/31605 | -1.24/0.26 | -13.7 | -13.4 | -7.64 | FALSE |
| SRX3040152 | TFs and others | STAG2 | Breast | HMEC | 21698 | 3269/31605 | 0.08/0.26 | -13.6 | -13.4 | -7.63 | FALSE |
| SRX16646094 | TFs and others | HSF1 | Breast | MCF-7 | 7311 | 1423/31605 | -0.00/0.26 | -13.6 | -13.4 | -7.63 | FALSE |
| SRX10775041 | TFs and others | RNF2 | Breast | T-47D | 1728 | 221/31605 | -0.40/0.26 | -13.6 | -13.4 | -7.62 | FALSE |
| SRX6828396 | TFs and others | RAD21 | Breast | MCF-7 | 43611 | 4737/31605 | 0.11/0.26 | -13.6 | -13.3 | -7.61 | FALSE |
| SRX2424329 | TFs and others | ZNF592 | Breast | MCF-7 | 1956 | 287/31605 | -0.32/0.26 | -13.6 | -13.3 | -7.61 | FALSE |
| SRX194578 | TFs and others | KLF4 | Breast | MCF-7 | 1608 | 504/31605 | -0.18/0.26 | -13.5 | -13.3 | -7.60 | FALSE |
| SRX3322136 | TFs and others | KLF4 | Breast | MCF-7 | 2492 | 691/31605 | -0.11/0.26 | -13.5 | -13.3 | -7.59 | FALSE |
| SRX19888700 | TFs and others | SNAI2 | Breast | BT-549 | 7044 | 2680/31605 | 0.07/0.26 | -13.5 | -13.2 | -7.58 | FALSE |
| SRX186801 | TFs and others | ESR1 | Breast | Breast cancer | 1559 | 44/31605 | -1.21/0.26 | -13.4 | -13.2 | -7.58 | FALSE |
| SRX6712591 | TFs and others | MED1 | Breast | MCF-7 | 991 | 325/31605 | -0.28/0.26 | -13.3 | -13.1 | -7.53 | FALSE |
| SRX5323931 | TFs and others | FOXA1 | Breast | Breast cancer cells | 6183 | 464/31605 | -0.19/0.26 | -13.3 | -13.0 | -7.52 | FALSE |
| SRX5089524 | TFs and others | SMARCA4 | Breast | MCF-7 | 2156 | 470/31605 | -0.19/0.26 | -13.3 | -13.0 | -7.52 | FALSE |
| SRX10142204 | TFs and others | CTCF | Breast | Breast cancer cells | 4389 | 1216/31605 | -0.02/0.26 | -13.2 | -13.0 | -7.52 | FALSE |
| SRX5824549 | TFs and others | BRD7 | Breast | SUM 159PT | 4805 | 2169/31605 | 0.05/0.26 | -13.2 | -13.0 | -7.51 | FALSE |
| SRX16874140 | TFs and others | ESR1 | Breast | MCF-7 | 945 | 19/31605 | -1.95/0.26 | -13.2 | -13.0 | -7.50 | FALSE |
| SRX13327958 | TFs and others | CBX3 | Breast | MCF-7 | 2096 | 411/31605 | -0.22/0.26 | -13.2 | -13.0 | -7.50 | FALSE |
| SRX5089426 | TFs and others | SMARCB1 | Breast | MCF-7 | 2033 | 275/31605 | -0.32/0.26 | -13.2 | -12.9 | -7.49 | FALSE |
| SRX11168160 | TFs and others | BRCA2 | Breast | MCF-7 | 2277 | 192/31605 | -0.44/0.26 | -13.2 | -12.9 | -7.49 | FALSE |
| SRX18013277 | TFs and others | VGLL1 | Breast | FULVR | 27361 | 2158/31605 | 0.05/0.26 | -13.1 | -12.9 | -7.49 | FALSE |
| SRX5089407 | TFs and others | SMARCA4 | Breast | MCF-7 | 7127 | 995/31605 | -0.05/0.26 | -13.1 | -12.9 | -7.48 | FALSE |
| SRX1012718 | TFs and others | PGR | Breast | T-47D | 7946 | 804/31605 | -0.08/0.26 | -13.1 | -12.9 | -7.47 | FALSE |
| SRX4671313 | TFs and others | RARA | Breast | SUM 159PT | 4668 | 813/31605 | -0.08/0.26 | -13.1 | -12.9 | -7.47 | FALSE |
| SRX6098147 | TFs and others | GATA3 | Breast | MCF-7 | 13618 | 865/31605 | -0.07/0.26 | -13.1 | -12.8 | -7.46 | FALSE |
| SRX268298 | TFs and others | FOSL1 | Breast | BT-549 | 18460 | 1686/31605 | 0.02/0.26 | -13.0 | -12.8 | -7.45 | FALSE |
| SRX176868 | TFs and others | GATA3 | Breast | MCF-7 | 1561 | 137/31605 | -0.56/0.26 | -13.0 | -12.8 | -7.45 | FALSE |
| SRX13327990 | TFs and others | GATA3 | Breast | MCF-7 | 5776 | 329/31605 | -0.27/0.26 | -13.0 | -12.8 | -7.45 | FALSE |
| SRX3322665 | TFs and others | NFIB | Breast | MCF-7 | 11110 | 1039/31605 | -0.04/0.26 | -13.0 | -12.8 | -7.44 | FALSE |
| SRX039139 | TFs and others | FOS | Breast | MCF-7 | 3503 | 388/31605 | -0.23/0.26 | -13.0 | -12.8 | -7.44 | FALSE |
| SRX5057620 | TFs and others | GATA3 | Breast | T-47D | 2948 | 339/31605 | -0.26/0.26 | -13.0 | -12.7 | -7.43 | FALSE |
| SRX6828397 | TFs and others | RAD21 | Breast | MCF-7 | 39907 | 4422/31605 | 0.11/0.26 | -12.9 | -12.7 | -7.43 | FALSE |
| SRX2871358 | TFs and others | PGR | Breast | T-47D | 1443 | 353/31605 | -0.25/0.26 | -12.9 | -12.7 | -7.42 | FALSE |
| SRX14756933 | TFs and others | DNA-RNA hybrid | Breast | MDA-MB-436 | 7815 | 2565/31605 | 0.07/0.26 | -12.9 | -12.7 | -7.42 | FALSE |
| SRX10775023 | TFs and others | RNF2 | Breast | T-47D | 1261 | 57/31605 | -1.00/0.26 | -12.9 | -12.7 | -7.42 | FALSE |
| SRX3583256 | TFs and others | HIF1A | Breast | MDA-MB-231 | 1235 | 203/31605 | -0.41/0.26 | -12.9 | -12.7 | -7.41 | FALSE |
| SRX9719180 | TFs and others | GATA3 | Breast | MCF-7 | 9202 | 820/31605 | -0.08/0.26 | -12.9 | -12.7 | -7.40 | FALSE |
| SRX1709977 | TFs and others | PGR | Breast | T-47D | 6195 | 745/31605 | -0.09/0.26 | -12.9 | -12.6 | -7.40 | FALSE |
| SRX14875685 | TFs and others | ESR1 | Breast | MCF-7 | 4049 | 356/31605 | -0.25/0.26 | -12.8 | -12.6 | -7.39 | FALSE |
| SRX13991442 | TFs and others | TCF4 | Breast | MCF 10A | 961 | 497/31605 | -0.17/0.26 | -12.8 | -12.6 | -7.39 | FALSE |
| SRX5089522 | TFs and others | SMARCA4 | Breast | MCF-7 | 2463 | 749/31605 | -0.09/0.26 | -12.8 | -12.6 | -7.38 | FALSE |
| SRX457214 | TFs and others | ESR1 | Breast | MCF-7 | 482 | 15/31605 | -2.19/0.26 | -12.8 | -12.6 | -7.38 | FALSE |
| SRX5702618 | TFs and others | AR | Breast | T-47D | 4866 | 279/31605 | -0.31/0.26 | -12.8 | -12.6 | -7.38 | FALSE |
| SRX1521310 | TFs and others | KDM5C | Breast | ZR-75-30 | 909 | 480/31605 | -0.18/0.26 | -12.8 | -12.5 | -7.37 | FALSE |
| SRX2020838 | TFs and others | STAT3 | Breast | MDA-MB-157 | 12595 | 1447/31605 | 0.01/0.26 | -12.7 | -12.5 | -7.36 | FALSE |
| SRX7121997 | TFs and others | SMARCA4 | Breast | MCF-7 | 3055 | 535/31605 | -0.15/0.26 | -12.7 | -12.5 | -7.35 | FALSE |
| SRX673745 | TFs and others | EP300 | Breast | MCF-7 | 1121 | 324/31605 | -0.27/0.26 | -12.7 | -12.5 | -7.35 | FALSE |
| SRX10024492 | TFs and others | FOXA1 | Breast | MCF-7 | 29801 | 2340/31605 | 0.06/0.26 | -12.7 | -12.5 | -7.34 | FALSE |
| SRX5089419 | TFs and others | SMARCA4 | Breast | MCF-7 | 4578 | 806/31605 | -0.08/0.26 | -12.6 | -12.4 | -7.32 | FALSE |
| SRX1070161 | TFs and others | AR | Breast | MDA-MB-453 | 31006 | 1724/31605 | 0.03/0.26 | -12.6 | -12.4 | -7.32 | FALSE |
| SRX5457544 | TFs and others | ZNF507 | Breast | MCF-7 | 2691 | 648/31605 | -0.11/0.26 | -12.6 | -12.4 | -7.32 | FALSE |
| SRX15232261 | TFs and others | DNA-RNA hybrid | Breast | MDA-MB-436 | 2645 | 1065/31605 | -0.03/0.26 | -12.6 | -12.3 | -7.31 | FALSE |
| SRX3322546 | TFs and others | NCOA3 | Breast | MCF-7 | 2011 | 66/31605 | -0.90/0.26 | -12.6 | -12.3 | -7.31 | FALSE |
| SRX213875 | TFs and others | EP300 | Breast | MCF-7 | 888 | 247/31605 | -0.34/0.26 | -12.5 | -12.3 | -7.30 | FALSE |
| SRX6712620 | TFs and others | SMC1A | Breast | MCF-7 | 35354 | 4410/31605 | 0.11/0.26 | -12.5 | -12.3 | -7.29 | FALSE |
| SRX518249 | TFs and others | AGO1 | Breast | MCF-7 | 495 | 33/31605 | -1.37/0.26 | -12.5 | -12.3 | -7.29 | FALSE |
| SRX4649670 | TFs and others | TP53 | Breast | MCF 10A | 8925 | 1265/31605 | -0.01/0.26 | -12.5 | -12.2 | -7.27 | FALSE |
| SRX4192136 | TFs and others | XRCC4 | Breast | MCF 10A | 1592 | 384/31605 | -0.22/0.26 | -12.4 | -12.2 | -7.27 | FALSE |
| SRX6858248 | TFs and others | HSF1 | Breast | MCF-7 | 1934 | 477/31605 | -0.17/0.26 | -12.4 | -12.2 | -7.27 | FALSE |
| SRX060811 | TFs and others | GATA3 | Breast | MCF-7 | 8220 | 804/31605 | -0.07/0.26 | -12.4 | -12.2 | -7.26 | FALSE |
| SRX7540321 | TFs and others | ESR1 | Breast | MCF-7 | 616 | 25/31605 | -1.61/0.26 | -12.4 | -12.2 | -7.26 | FALSE |
| SRX8520798 | TFs and others | STAT3 | Breast | MDA-MB-361 | 1620 | 415/31605 | -0.20/0.26 | -12.4 | -12.2 | -7.26 | FALSE |
| SRX6487064 | TFs and others | FOXA1 | Breast | MCF-7 | 7869 | 656/31605 | -0.11/0.26 | -12.4 | -12.2 | -7.25 | FALSE |
| SRX5089435 | TFs and others | SMARCB1 | Breast | MCF-7 | 5877 | 603/31605 | -0.12/0.26 | -12.4 | -12.2 | -7.25 | FALSE |
| SRX3321895 | TFs and others | CEBPG | Breast | MCF-7 | 4997 | 750/31605 | -0.08/0.26 | -12.4 | -12.1 | -7.25 | FALSE |
| SRX2424139 | TFs and others | MLLT1 | Breast | MCF-7 | 1928 | 429/31605 | -0.19/0.26 | -12.3 | -12.1 | -7.22 | FALSE |
| SRX3322611 | TFs and others | SMARCA5 | Breast | MCF-7 | 2465 | 301/31605 | -0.28/0.26 | -12.2 | -12.0 | -7.19 | FALSE |
| SRX8537747 | TFs and others | GATA3 | Breast | MDA-MB-134-VI | 1418 | 45/31605 | -1.12/0.26 | -12.2 | -12.0 | -7.19 | FALSE |
| SRX4096886 | TFs and others | TRPS1 | Breast | HCC3153 | 12372 | 1246/31605 | -0.01/0.26 | -12.2 | -11.9 | -7.18 | FALSE |
| SRX2194268 | TFs and others | EP300 | Breast | SUM 159PT | 25317 | 2303/31605 | 0.06/0.26 | -12.1 | -11.9 | -7.16 | FALSE |
| SRX22740968 | TFs and others | DVL1 | Breast | MDA-MB-231 | 4535 | 927/31605 | -0.05/0.26 | -12.1 | -11.9 | -7.16 | FALSE |
| SRX3800730 | TFs and others | RAD21 | Breast | T-47D | 4381 | 1851/31605 | 0.04/0.26 | -12.1 | -11.9 | -7.15 | FALSE |
| SRX24612541 | TFs and others | FOXA1 | Breast | MCF-7 | 11578 | 1186/31605 | -0.01/0.26 | -12.1 | -11.8 | -7.15 | FALSE |
| SRX2378693 | TFs and others | AHRR | Breast | MCF-7 | 2144 | 497/31605 | -0.16/0.26 | -12.1 | -11.8 | -7.15 | FALSE |
| SRX14612993 | TFs and others | ESR1 | Breast | T-47D | 1559 | 38/31605 | -1.23/0.26 | -12.0 | -11.8 | -7.13 | FALSE |
| SRX4096869 | TFs and others | GATAD2B | Breast | HCC3153 | 2273 | 595/31605 | -0.12/0.26 | -12.0 | -11.8 | -7.13 | FALSE |
| SRX14267243 | TFs and others | MYC | Breast | MCF 10A | 663 | 345/31605 | -0.24/0.26 | -12.0 | -11.8 | -7.13 | FALSE |
| SRX3321896 | TFs and others | CEBPG | Breast | MCF-7 | 4299 | 667/31605 | -0.10/0.26 | -12.0 | -11.8 | -7.13 | FALSE |
| SRX5128131 | TFs and others | AR | Breast | ZR-75-1 | 21823 | 1330/31605 | 0.01/0.26 | -11.9 | -11.7 | -7.11 | FALSE |
| SRX4096859 | TFs and others | GATAD2A | Breast | HCC3153 | 1647 | 506/31605 | -0.15/0.26 | -11.9 | -11.7 | -7.11 | FALSE |
| SRX11867606 | TFs and others | KDM6A | Breast | CAL-51 | 2203 | 586/31605 | -0.12/0.26 | -11.9 | -11.7 | -7.11 | FALSE |
| SRX5089394 | TFs and others | ARID1A | Breast | MCF-7 | 3243 | 122/31605 | -0.57/0.26 | -11.9 | -11.7 | -7.10 | FALSE |
| SRX20282941 | TFs and others | AHR | Breast | MCF-7 | 1022 | 75/31605 | -0.79/0.26 | -11.9 | -11.7 | -7.10 | FALSE |
| SRX747796 | TFs and others | FOXA1 | Breast | MCF-7 | 13349 | 1445/31605 | 0.02/0.26 | -11.9 | -11.7 | -7.10 | FALSE |
| SRX3428653 | TFs and others | ESR1 | Breast | MCF-7 | 1916 | 28/31605 | -1.46/0.26 | -11.9 | -11.7 | -7.10 | FALSE |
| SRX2871363 | TFs and others | PGR | Breast | T-47D | 1635 | 328/31605 | -0.25/0.26 | -11.8 | -11.6 | -7.08 | FALSE |
| SRX5287704 | TFs and others | TEAD4 | Breast | MCF-7 | 2070 | 435/31605 | -0.18/0.26 | -11.8 | -11.6 | -7.08 | FALSE |
| SRX5203316 | TFs and others | CTCF | Breast | MCF-7 | 17686 | 2215/31605 | 0.06/0.26 | -11.8 | -11.6 | -7.07 | FALSE |
| SRX3322254 | TFs and others | NFXL1 | Breast | MCF-7 | 1872 | 137/31605 | -0.52/0.26 | -11.7 | -11.5 | -7.05 | FALSE |
| SRX5794312 | TFs and others | TCF7 | Breast | Breast organoids | 6003 | 1291/31605 | 0.00/0.26 | -11.7 | -11.5 | -7.05 | FALSE |
| SRX5057610 | TFs and others | GATA3 | Breast | T-47D | 2028 | 284/31605 | -0.28/0.26 | -11.7 | -11.5 | -7.04 | FALSE |
| SRX20504947 | TFs and others | STAT3 | Breast | Breast cancer cells | 2823 | 554/31605 | -0.13/0.26 | -11.7 | -11.5 | -7.03 | FALSE |
| SRX4192958 | TFs and others | SMC1A | Breast | MCF-7 | 24795 | 3648/31605 | 0.11/0.26 | -11.7 | -11.5 | -7.03 | FALSE |
| SRX10365537 | TFs and others | RAC1 | Breast | MCF-7 | 10800 | 641/31605 | -0.10/0.26 | -11.7 | -11.5 | -7.03 | FALSE |
| SRX5457296 | TFs and others | CLOCK | Breast | MCF-7 | 1788 | 335/31605 | -0.24/0.26 | -11.7 | -11.4 | -7.02 | FALSE |
| SRX5457432 | TFs and others | E4F1 | Breast | MCF-7 | 2001 | 455/31605 | -0.17/0.26 | -11.7 | -11.4 | -7.02 | FALSE |
| SRX3554406 | TFs and others | CREB1 | Breast | MDA-MB-134-VI | 1479 | 558/31605 | -0.13/0.26 | -11.6 | -11.4 | -7.02 | FALSE |
| SRX5203296 | TFs and others | CTCF | Breast | MCF-7 | 13783 | 1796/31605 | 0.04/0.26 | -11.6 | -11.4 | -7.01 | FALSE |
| SRX11858165 | TFs and others | TRIM37 | Breast | MCF-7 | 575 | 32/31605 | -1.33/0.26 | -11.6 | -11.4 | -7.00 | FALSE |
| SRX20830464 | TFs and others | Epitope tags | Breast | T-47D | 1280 | 29/31605 | -1.41/0.26 | -11.6 | -11.4 | -7.00 | FALSE |
| SRX8084743 | TFs and others | Epitope tags | Breast | WHIM12 | 2231 | 384/31605 | -0.20/0.26 | -11.6 | -11.4 | -6.99 | FALSE |
| SRX4213898 | TFs and others | YAP1 | Breast | MCF 10A | 1196 | 415/31605 | -0.18/0.26 | -11.6 | -11.4 | -6.99 | FALSE |
| SRX176872 | TFs and others | GATA3 | Breast | MCF-7 | 822 | 52/31605 | -0.99/0.26 | -11.6 | -11.3 | -6.99 | FALSE |
| SRX8537748 | TFs and others | GATA3 | Breast | MDA-MB-134-VI | 1398 | 63/31605 | -0.87/0.26 | -11.6 | -11.3 | -6.99 | FALSE |
| SRX2578883 | TFs and others | MED1 | Breast | MDA-MB-231 | 1945 | 615/31605 | -0.11/0.26 | -11.5 | -11.3 | -6.98 | FALSE |
| SRX11867608 | TFs and others | KMT2C | Breast | CAL-51 | 1324 | 502/31605 | -0.14/0.26 | -11.5 | -11.3 | -6.96 | FALSE |
| SRX747797 | TFs and others | FOXA1 | Breast | MCF-7 | 8947 | 682/31605 | -0.09/0.26 | -11.4 | -11.2 | -6.94 | FALSE |
| SRX5534859 | TFs and others | ARID1B | Breast | MCF-7 | 988 | 187/31605 | -0.39/0.26 | -11.4 | -11.2 | -6.94 | FALSE |
| SRX2194238 | TFs and others | MED1 | Breast | SUM 159PT | 3063 | 752/31605 | -0.07/0.26 | -11.4 | -11.2 | -6.93 | FALSE |
| SRX3322356 | TFs and others | EP300 | Breast | Breast epithelium | 1236 | 346/31605 | -0.22/0.26 | -11.4 | -11.1 | -6.92 | FALSE |
| SRX1131550 | TFs and others | BRD4 | Breast | HCC1395 | 9763 | 1123/31605 | -0.01/0.26 | -11.3 | -11.1 | -6.92 | FALSE |
| SRX5089437 | TFs and others | SMARCB1 | Breast | MCF-7 | 3325 | 345/31605 | -0.22/0.26 | -11.3 | -11.1 | -6.90 | FALSE |
| SRX19210542 | TFs and others | EZH2 | Breast | MDA-MB-231 | 5616 | 3821/31605 | 0.11/0.26 | -11.3 | -11.1 | -6.90 | FALSE |
| SRX5089387 | TFs and others | ARID1A | Breast | MCF-7 | 1595 | 83/31605 | -0.71/0.26 | -11.3 | -11.1 | -6.90 | FALSE |
| SRX2512919 | TFs and others | FOXA1 | Breast | MCF-7 | 9232 | 614/31605 | -0.10/0.26 | -11.2 | -11.0 | -6.88 | FALSE |
| SRX100273 | TFs and others | CARM1 | Breast | H3396 | 521 | 28/31605 | -1.41/0.26 | -11.2 | -11.0 | -6.88 | FALSE |
| SRX5511312 | TFs and others | ESR1 | Breast | MDA-MB-231 | 798 | 29/31605 | -1.38/0.26 | -11.2 | -11.0 | -6.88 | FALSE |
| SRX8520794 | TFs and others | STAT3 | Breast | HCC1937 | 4513 | 898/31605 | -0.04/0.26 | -11.2 | -11.0 | -6.87 | FALSE |
| SRX13941919 | TFs and others | ARID1A | Breast | MCF-7 | 7777 | 523/31605 | -0.13/0.26 | -11.2 | -10.9 | -6.86 | FALSE |
| SRX3011703 | TFs and others | MED12 | Breast | MCF-7 | 815 | 18/31605 | -1.81/0.26 | -11.1 | -10.9 | -6.85 | FALSE |
| SRX3322945 | TFs and others | GATAD2B | Breast | MCF-7 | 2690 | 269/31605 | -0.28/0.26 | -11.1 | -10.9 | -6.85 | FALSE |
| SRX10773086 | TFs and others | EBF1 | Breast | MB 157 | 988 | 232/31605 | -0.32/0.26 | -11.1 | -10.9 | -6.85 | FALSE |
| SRX647371 | TFs and others | ZBTB17 | Breast | MCF 10A | 1746 | 567/31605 | -0.11/0.26 | -11.1 | -10.9 | -6.84 | FALSE |
| SRX2424191 | TFs and others | NBN | Breast | MCF-7 | 3197 | 417/31605 | -0.17/0.26 | -11.1 | -10.9 | -6.83 | FALSE |
| SRX186827 | TFs and others | ESR1 | Breast | Breast cancer | 1174 | 39/31605 | -1.15/0.26 | -11.1 | -10.9 | -6.83 | FALSE |
| SRX5089549 | TFs and others | ARID2 | Breast | MCF-7 | 1161 | 94/31605 | -0.65/0.26 | -11.0 | -10.8 | -6.82 | FALSE |
| SRX3923522 | TFs and others | FOXA1 | Breast | ZR-75-1 | 26683 | 2103/31605 | 0.06/0.26 | -11.0 | -10.8 | -6.82 | FALSE |
| SRX3322137 | TFs and others | KLF4 | Breast | MCF-7 | 1781 | 419/31605 | -0.17/0.26 | -11.0 | -10.8 | -6.82 | FALSE |
| SRX14267244 | TFs and others | MYC | Breast | MCF 10A | 759 | 383/31605 | -0.19/0.26 | -11.0 | -10.8 | -6.81 | FALSE |
| SRX518264 | TFs and others | AGO1 | Breast | MCF 10A | 173 | 7/31605 | -3.04/0.26 | -11.0 | -10.8 | -6.80 | FALSE |
| SRX4096882 | TFs and others | TRPS1 | Breast | HCC3153 | 9844 | 1095/31605 | -0.01/0.26 | -10.9 | -10.7 | -6.79 | FALSE |
| SRX6860650 | TFs and others | RNF2 | Breast | T-47D | 1448 | 54/31605 | -0.93/0.26 | -10.9 | -10.7 | -6.79 | FALSE |
| SRX5534861 | TFs and others | SMARCA4 | Breast | MCF-7 | 1040 | 609/31605 | -0.10/0.26 | -10.9 | -10.7 | -6.79 | FALSE |
| SRX15430995 | TFs and others | TFE3 | Breast | SK-BR-3 | 9429 | 892/31605 | -0.04/0.26 | -10.9 | -10.7 | -6.78 | FALSE |
| SRX8084745 | TFs and others | Epitope tags | Breast | WHIM12 | 2176 | 473/31605 | -0.14/0.26 | -10.9 | -10.7 | -6.78 | FALSE |
| SRX2900155 | TFs and others | SUZ12 | Breast | CAL-51 | 4735 | 3288/31605 | 0.10/0.26 | -10.9 | -10.7 | -6.77 | FALSE |
| SRX11149855 | TFs and others | AR | Breast | MFM-223 | 17714 | 1166/31605 | 0.00/0.26 | -10.9 | -10.6 | -6.76 | FALSE |
| SRX2578890 | TFs and others | NCOA1 | Breast | MDA-MB-231 | 480 | 252/31605 | -0.29/0.26 | -10.9 | -10.6 | -6.76 | FALSE |
| SRX3541114 | TFs and others | SUMO2 | Breast | MCF-7 | 1642 | 129/31605 | -0.51/0.26 | -10.9 | -10.6 | -6.76 | FALSE |
| SRX10142202 | TFs and others | CTCF | Breast | Breast cancer cell line | 2958 | 905/31605 | -0.03/0.26 | -10.8 | -10.6 | -6.76 | FALSE |
| SRX7644314 | TFs and others | RBMX | Breast | MCF-7 | 367 | 203/31605 | -0.35/0.26 | -10.8 | -10.6 | -6.75 | FALSE |
| SRX3923523 | TFs and others | FOXA1 | Breast | ZR-75-1 | 30767 | 2256/31605 | 0.07/0.26 | -10.8 | -10.6 | -6.75 | FALSE |
| SRX5203299 | TFs and others | CTCF | Breast | MCF-7 | 12945 | 1606/31605 | 0.04/0.26 | -10.8 | -10.6 | -6.75 | FALSE |
| SRX1012702 | TFs and others | ESR1 | Breast | T-47D | 1107 | 46/31605 | -1.02/0.26 | -10.8 | -10.6 | -6.75 | FALSE |
| SRX10119381 | TFs and others | STAT3 | Breast | MCF 10A | 2985 | 756/31605 | -0.06/0.26 | -10.8 | -10.6 | -6.74 | FALSE |
| SRX10579966 | TFs and others | MTA3 | Breast | MCF-7 | 2657 | 533/31605 | -0.12/0.26 | -10.8 | -10.6 | -6.73 | FALSE |
| SRX4096860 | TFs and others | GATAD2A | Breast | HCC3153 | 997 | 268/31605 | -0.27/0.26 | -10.8 | -10.6 | -6.73 | FALSE |
| SRX2423570 | TFs and others | JUN | Breast | MCF-7 | 2126 | 447/31605 | -0.15/0.26 | -10.8 | -10.6 | -6.73 | FALSE |
| SRX13327989 | TFs and others | GATA3 | Breast | MCF-7 | 7452 | 413/31605 | -0.17/0.26 | -10.8 | -10.6 | -6.73 | FALSE |
| SRX969016 | TFs and others | ESR1 | Breast | MCF-7 | 3441 | 867/31605 | -0.04/0.26 | -10.7 | -10.5 | -6.70 | FALSE |
| SRX8617945 | TFs and others | FGFR1 | Breast | CAMA-1 | 2075 | 493/31605 | -0.13/0.26 | -10.7 | -10.5 | -6.70 | FALSE |
| SRX4096858 | TFs and others | GATAD2A | Breast | HCC3153 | 1278 | 351/31605 | -0.20/0.26 | -10.6 | -10.4 | -6.68 | FALSE |
| SRX518256 | TFs and others | AGO1 | Breast | MCF 10A | 503 | 34/31605 | -1.21/0.26 | -10.6 | -10.4 | -6.67 | FALSE |
| SRX3589923 | TFs and others | STAG2 | Breast | MCF 10A | 16947 | 3010/31605 | 0.10/0.26 | -10.6 | -10.4 | -6.66 | FALSE |
| SRX10977443 | TFs and others | ZNF227 | Breast | MCF-7 | 1535 | 151/31605 | -0.44/0.26 | -10.5 | -10.3 | -6.65 | FALSE |
| SRX12788794 | TFs and others | JMJD6 | Breast | MCF-7 | 504 | 143/31605 | -0.46/0.26 | -10.5 | -10.3 | -6.65 | FALSE |
| SRX2578887 | TFs and others | MED1 | Breast | MDA-MB-231 | 2204 | 587/31605 | -0.10/0.26 | -10.5 | -10.3 | -6.65 | FALSE |
| SRX3589919 | TFs and others | STAG2 | Breast | MCF 10A | 11625 | 2273/31605 | 0.08/0.26 | -10.5 | -10.3 | -6.65 | FALSE |
| SRX6098150 | TFs and others | TRPS1 | Breast | MCF-7 | 1676 | 91/31605 | -0.64/0.26 | -10.5 | -10.3 | -6.64 | FALSE |
| SRX6712593 | TFs and others | MED26 | Breast | MCF-7 | 966 | 256/31605 | -0.28/0.26 | -10.5 | -10.3 | -6.64 | FALSE |
| SRX3401274 | TFs and others | YAP1 | Breast | MCF-7 | 7953 | 663/31605 | -0.07/0.26 | -10.5 | -10.3 | -6.63 | FALSE |
| SRX5493757 | TFs and others | FOXA1 | Breast | Breast cancer cells | 5928 | 663/31605 | -0.07/0.26 | -10.4 | -10.2 | -6.62 | FALSE |
| SRX20830465 | TFs and others | Epitope tags | Breast | T-47D | 1183 | 27/31605 | -1.37/0.26 | -10.4 | -10.2 | -6.60 | FALSE |
| SRX5089389 | TFs and others | ARID1A | Breast | MCF-7 | 2723 | 190/31605 | -0.36/0.26 | -10.4 | -10.2 | -6.59 | FALSE |
| SRX194574 | TFs and others | GREB1 | Breast | MCF-7 | 1562 | 174/31605 | -0.38/0.26 | -10.3 | -10.1 | -6.59 | FALSE |
| SRX1012675 | TFs and others | ESR1 | Breast | T-47D | 1414 | 56/31605 | -0.87/0.26 | -10.3 | -10.1 | -6.59 | FALSE |
| SRX3589920 | TFs and others | STAG2 | Breast | MCF 10A | 10045 | 2091/31605 | 0.07/0.26 | -10.3 | -10.1 | -6.56 | FALSE |
| SRX968978 | TFs and others | RELA | Breast | MCF-7 | 2727 | 782/31605 | -0.05/0.26 | -10.3 | -10.1 | -6.56 | FALSE |
| SRX6487041 | TFs and others | FOXA1 | Breast | MCF-7 | 11344 | 921/31605 | -0.02/0.26 | -10.3 | -10.0 | -6.56 | FALSE |
| SRX5457131 | TFs and others | NEUROD1 | Breast | MCF-7 | 1445 | 131/31605 | -0.48/0.26 | -10.2 | -10.0 | -6.55 | FALSE |
| SRX15232266 | TFs and others | DNA-RNA hybrid | Breast | MDA-MB-436 | 1229 | 631/31605 | -0.08/0.26 | -10.2 | -10.0 | -6.54 | FALSE |
| SRX12193406 | TFs and others | ESR1 | Breast | MCF-7 | 615 | 58/31605 | -0.84/0.26 | -10.2 | -10.0 | -6.54 | FALSE |
| SRX1012704 | TFs and others | ESR1 | Breast | T-47D | 1202 | 55/31605 | -0.87/0.26 | -10.2 | -10.0 | -6.54 | FALSE |
| SRX1131572 | TFs and others | BRD4 | Breast | T-47D | 3106 | 270/31605 | -0.25/0.26 | -10.2 | -10.0 | -6.53 | FALSE |
| SRX1076935 | TFs and others | LMTK3 | Breast | MCF-7 | 2284 | 317/31605 | -0.21/0.26 | -10.2 | -10.0 | -6.53 | FALSE |
| SRX10024479 | TFs and others | FOXA1 | Breast | MCF-7 | 13918 | 1300/31605 | 0.02/0.26 | -10.2 | -10.0 | -6.53 | FALSE |
| SRX2881008 | TFs and others | ESR1 | Breast | MCF-7 | 1043 | 16/31605 | -1.83/0.26 | -10.1 | -9.9 | -6.51 | FALSE |
| SRX13254861 | TFs and others | SUZ12 | Breast | MCF-7 | 48791 | 6233/31605 | 0.15/0.26 | -10.1 | -9.9 | -6.50 | FALSE |
| SRX4192134 | TFs and others | PARP1 | Breast | MCF 10A | 676 | 193/31605 | -0.34/0.26 | -10.1 | -9.9 | -6.50 | FALSE |
| SRX7121993 | TFs and others | SMARCC1 | Breast | MCF-7 | 753 | 31/31605 | -1.24/0.26 | -10.1 | -9.9 | -6.50 | FALSE |
| SRX13327967 | TFs and others | CBX3 | Breast | MCF-7 | 2334 | 428/31605 | -0.15/0.26 | -10.1 | -9.9 | -6.50 | FALSE |
| SRX3322944 | TFs and others | GATAD2B | Breast | MCF-7 | 2953 | 321/31605 | -0.21/0.26 | -10.1 | -9.9 | -6.49 | FALSE |
| SRX852985 | TFs and others | RUNX1 | Breast | MCF-7 | 641 | 88/31605 | -0.63/0.26 | -10.0 | -9.8 | -6.48 | FALSE |
| SRX6487069 | TFs and others | FOXA1 | Breast | MCF-7 | 4470 | 321/31605 | -0.21/0.26 | -10.0 | -9.8 | -6.48 | FALSE |
| SRX2423867 | TFs and others | SIX4 | Breast | MCF-7 | 4331 | 324/31605 | -0.21/0.26 | -10.0 | -9.8 | -6.48 | FALSE |
| SRX1012713 | TFs and others | PGR | Breast | T-47D | 6680 | 716/31605 | -0.05/0.26 | -10.0 | -9.8 | -6.47 | FALSE |
| SRX8850931 | TFs and others | Epitope tags | Breast | MCF 10A | 1030 | 189/31605 | -0.35/0.26 | -10.0 | -9.8 | -6.47 | FALSE |
| SRX5574443 | TFs and others | EN1 | Breast | MCF-7 | 2694 | 184/31605 | -0.36/0.26 | -10.0 | -9.8 | -6.47 | FALSE |
| SRX3923527 | TFs and others | FOXA1 | Breast | ZR-75-1 | 22828 | 1619/31605 | 0.05/0.26 | -10.0 | -9.8 | -6.46 | FALSE |
| SRX5089421 | TFs and others | SMARCA4 | Breast | MCF-7 | 6689 | 990/31605 | -0.01/0.26 | -9.9 | -9.7 | -6.44 | FALSE |
| SRX3629601 | TFs and others | ESR1 | Breast | MCF-7 | 162 | 10/31605 | -2.35/0.26 | -9.9 | -9.7 | -6.44 | FALSE |
| SRX4192883 | TFs and others | JUN | Breast | MCF 10A | 17210 | 1579/31605 | 0.05/0.26 | -9.9 | -9.7 | -6.43 | FALSE |
| SRX1012717 | TFs and others | PGR | Breast | T-47D | 5273 | 523/31605 | -0.10/0.26 | -9.9 | -9.7 | -6.43 | FALSE |
| SRX392293 | TFs and others | PRKCQ | Breast | MCF-7 | 1067 | 258/31605 | -0.26/0.26 | -9.9 | -9.7 | -6.42 | FALSE |
| SRX20830473 | TFs and others | PGR | Breast | T-47D | 520 | 15/31605 | -1.87/0.26 | -9.9 | -9.7 | -6.42 | FALSE |
| SRX14101950 | TFs and others | SNAI2 | Breast | MCF-7 | 6605 | 1003/31605 | -0.00/0.26 | -9.9 | -9.7 | -6.42 | FALSE |
| SRX15337038 | TFs and others | Epitope tags | Breast | MCF 10A | 851 | 68/31605 | -0.74/0.26 | -9.8 | -9.6 | -6.41 | FALSE |
| SRX1012706 | TFs and others | ESR1 | Breast | T-47D | 1599 | 52/31605 | -0.88/0.26 | -9.8 | -9.6 | -6.41 | FALSE |
| SRX3923524 | TFs and others | FOXA1 | Breast | ZR-75-1 | 24481 | 1839/31605 | 0.06/0.26 | -9.8 | -9.6 | -6.41 | FALSE |
| SRX15337039 | TFs and others | Epitope tags | Breast | MCF 10A | 838 | 54/31605 | -0.86/0.26 | -9.8 | -9.6 | -6.41 | FALSE |
| SRX19210541 | TFs and others | RELB | Breast | SUM 149PT | 1491 | 514/31605 | -0.11/0.26 | -9.8 | -9.6 | -6.41 | FALSE |
| SRX2423571 | TFs and others | JUN | Breast | MCF-7 | 2527 | 386/31605 | -0.16/0.26 | -9.8 | -9.6 | -6.40 | FALSE |
| SRX13991431 | TFs and others | CTNNB1 | Breast | MCF 10A | 1965 | 422/31605 | -0.14/0.26 | -9.8 | -9.6 | -6.40 | FALSE |
| SRX6487038 | TFs and others | FOXA1 | Breast | MCF-7 | 5014 | 416/31605 | -0.15/0.26 | -9.8 | -9.6 | -6.40 | FALSE |
| SRX6487061 | TFs and others | FOXA1 | Breast | MCF-7 | 2481 | 158/31605 | -0.40/0.26 | -9.8 | -9.6 | -6.39 | FALSE |
| SRX8080574 | TFs and others | CTCF | Breast | Mammary gland ductal carcinoma | 11145 | 1612/31605 | 0.05/0.26 | -9.8 | -9.6 | -6.39 | FALSE |
| SRX10641846 | TFs and others | TP73 | Breast | SUM 159PT | 8220 | 947/31605 | -0.01/0.26 | -9.7 | -9.5 | -6.38 | FALSE |
| SRX7644313 | TFs and others | Epitope tags | Breast | MCF-7 | 1145 | 375/31605 | -0.17/0.26 | -9.7 | -9.5 | -6.36 | FALSE |
| SRX15039965 | TFs and others | GATA3 | Breast | MDA-MB-231 | 8429 | 1049/31605 | 0.00/0.26 | -9.7 | -9.5 | -6.36 | FALSE |
| SRX19888703 | TFs and others | SNAI2 | Breast | BT-549 | 4560 | 1497/31605 | 0.04/0.26 | -9.7 | -9.5 | -6.36 | FALSE |
| SRX2881006 | TFs and others | ESR1 | Breast | MCF-7 | 905 | 14/31605 | -1.92/0.26 | -9.7 | -9.5 | -6.35 | FALSE |
| SRX4096881 | TFs and others | TRPS1 | Breast | HCC3153 | 7316 | 768/31605 | -0.04/0.26 | -9.7 | -9.5 | -6.35 | FALSE |
| SRX20830461 | TFs and others | Epitope tags | Breast | T-47D | 1404 | 56/31605 | -0.83/0.26 | -9.6 | -9.4 | -6.34 | FALSE |
| SRX1131563 | TFs and others | BRD4 | Breast | SUM 149PT | 1842 | 285/31605 | -0.23/0.26 | -9.6 | -9.4 | -6.34 | FALSE |
| SRX3410384 | TFs and others | PCGF2 | Breast | MDA-MB-231 | 6262 | 633/31605 | -0.07/0.26 | -9.6 | -9.4 | -6.34 | FALSE |
| SRX1070162 | TFs and others | AR | Breast | MDA-MB-453 | 33189 | 1612/31605 | 0.05/0.26 | -9.6 | -9.4 | -6.34 | FALSE |
| SRX5323827 | TFs and others | FOXA1 | Breast | T-47D | 4922 | 547/31605 | -0.09/0.26 | -9.6 | -9.4 | -6.32 | FALSE |
| SRX883578 | TFs and others | WWTR1 | Breast | MDA-MB-231 | 10727 | 1220/31605 | 0.02/0.26 | -9.6 | -9.4 | -6.32 | FALSE |
| SRX15826245 | TFs and others | KDM6A | Breast | MDA-MB-231 | 7169 | 1326/31605 | 0.03/0.26 | -9.6 | -9.4 | -6.31 | FALSE |
| SRX5824562 | TFs and others | BRD4 | Breast | SUM 149PT | 1847 | 306/31605 | -0.21/0.26 | -9.5 | -9.3 | -6.31 | FALSE |
| SRX1012714 | TFs and others | PGR | Breast | T-47D | 5102 | 505/31605 | -0.10/0.26 | -9.5 | -9.3 | -6.30 | FALSE |
| SRX4192879 | TFs and others | FOS | Breast | MCF 10A | 60189 | 4515/31605 | 0.13/0.26 | -9.5 | -9.3 | -6.30 | FALSE |
| SRX18191662 | TFs and others | ESR1 | Breast | MCF-7 | 841 | 111/31605 | -0.51/0.26 | -9.5 | -9.3 | -6.30 | FALSE |
| SRX7114562 | TFs and others | Epitope tags | Breast | MCF-7 | 746 | 51/31605 | -0.87/0.26 | -9.5 | -9.3 | -6.29 | FALSE |
| SRX17657147 | TFs and others | FOXC1 | Breast | BT-549 | 7079 | 796/31605 | -0.03/0.26 | -9.5 | -9.3 | -6.29 | FALSE |
| SRX2900093 | TFs and others | BAP1 | Breast | CAL-51 | 1021 | 209/31605 | -0.30/0.26 | -9.5 | -9.3 | -6.29 | FALSE |
| SRX4096862 | TFs and others | GATAD2A | Breast | HCC3153 | 1455 | 362/31605 | -0.17/0.26 | -9.5 | -9.3 | -6.29 | FALSE |
| SRX8520808 | TFs and others | NR3C1 | Breast | MDA-MB-453 | 10861 | 758/31605 | -0.04/0.26 | -9.5 | -9.3 | -6.28 | FALSE |
| SRX6487048 | TFs and others | FOXA1 | Breast | MCF-7 | 9537 | 775/31605 | -0.03/0.26 | -9.5 | -9.3 | -6.27 | FALSE |
| SRX3589924 | TFs and others | STAG2 | Breast | MCF 10A | 14809 | 2644/31605 | 0.10/0.26 | -9.4 | -9.3 | -6.27 | FALSE |
| SRX3322957 | TFs and others | ZNF512B | Breast | MCF-7 | 2572 | 444/31605 | -0.12/0.26 | -9.4 | -9.2 | -6.25 | FALSE |
| SRX186818 | TFs and others | ESR1 | Breast | Breast cancer | 1213 | 26/31605 | -1.31/0.26 | -9.4 | -9.2 | -6.25 | FALSE |
| SRX3229158 | TFs and others | ESR1 | Breast | Breast cancer | 398 | 141/31605 | -0.42/0.26 | -9.4 | -9.2 | -6.25 | FALSE |
| SRX5203301 | TFs and others | CTCF | Breast | MCF-7 | 11491 | 1543/31605 | 0.05/0.26 | -9.4 | -9.2 | -6.24 | FALSE |
| SRX2897213 | TFs and others | ESR1 | Breast | Breast cancer cells | 943 | 741/31605 | -0.04/0.26 | -9.4 | -9.2 | -6.24 | FALSE |
| SRX3229183 | TFs and others | NR3C1 | Breast | Breast cancer | 611 | 183/31605 | -0.34/0.26 | -9.3 | -9.2 | -6.24 | FALSE |
| SRX3583257 | TFs and others | ZMYND8 | Breast | MDA-MB-231 | 7973 | 891/31605 | -0.01/0.26 | -9.3 | -9.1 | -6.23 | FALSE |
| SRX10119374 | TFs and others | TEAD1 | Breast | MCF 10A | 9469 | 1323/31605 | 0.04/0.26 | -9.3 | -9.1 | -6.23 | FALSE |
| SRX1070163 | TFs and others | AR | Breast | MDA-MB-453 | 18269 | 899/31605 | -0.01/0.26 | -9.3 | -9.1 | -6.23 | FALSE |
| SRX10184541 | TFs and others | GATA3 | Breast | HCC1954 | 8214 | 1149/31605 | 0.02/0.26 | -9.3 | -9.1 | -6.23 | FALSE |
| SRX19321943 | TFs and others | RNF2 | Breast | MCF-7 | 2031 | 1072/31605 | 0.50/0.26 | -9.3 | -9.1 | 6.20 | TRUE |
| SRX19210545 | TFs and others | NFKB2 | Breast | MDA-MB-231 | 919 | 215/31605 | -0.29/0.26 | -9.2 | -9.1 | -6.20 | FALSE |
| SRX16646093 | TFs and others | HSF1 | Breast | MCF-7 | 2160 | 506/31605 | -0.10/0.26 | -9.2 | -9.0 | -6.20 | FALSE |
| SRX3322569 | TFs and others | FOSL2 | Breast | MCF-7 | 5000 | 535/31605 | -0.09/0.26 | -9.2 | -9.0 | -6.19 | FALSE |
| SRX6027741 | TFs and others | FOXA1 | Breast | MCF-7 | 4703 | 419/31605 | -0.13/0.26 | -9.2 | -9.0 | -6.19 | FALSE |
| SRX3546796 | TFs and others | Epitope tags | Breast | MDA-MB-231 | 208 | 39/31605 | -1.01/0.26 | -9.2 | -9.0 | -6.18 | FALSE |
| SRX2424057 | TFs and others | FOXK2 | Breast | MCF-7 | 1442 | 333/31605 | -0.18/0.26 | -9.2 | -9.0 | -6.18 | FALSE |
| SRX19024879 | TFs and others | NR3C1 | Breast | MCF-7 | 16140 | 948/31605 | -0.00/0.26 | -9.2 | -9.0 | -6.18 | FALSE |
| SRX176867 | TFs and others | GATA3 | Breast | MCF-7 | 15388 | 1266/31605 | 0.03/0.26 | -9.2 | -9.0 | -6.18 | FALSE |
| SRX2578884 | TFs and others | MED1 | Breast | MDA-MB-231 | 562 | 40/31605 | -0.99/0.26 | -9.2 | -9.0 | -6.17 | FALSE |
| SRX2424763 | TFs and others | CUX1 | Breast | MCF-7 | 4221 | 395/31605 | -0.14/0.26 | -9.2 | -9.0 | -6.17 | FALSE |
| SRX16500147 | TFs and others | TSC22D2 | Breast | MCF-7 | 1879 | 119/31605 | -0.47/0.26 | -9.1 | -8.9 | -6.14 | FALSE |
| SRX1161196 | TFs and others | NR3C1 | Breast | T-47D | 1873 | 75/31605 | -0.65/0.26 | -9.1 | -8.9 | -6.13 | FALSE |
| SRX20830474 | TFs and others | PGR | Breast | T-47D | 582 | 16/31605 | -1.71/0.26 | -9.1 | -8.9 | -6.13 | FALSE |
| SRX5089434 | TFs and others | SMARCB1 | Breast | MCF-7 | 3441 | 372/31605 | -0.15/0.26 | -9.0 | -8.8 | -6.12 | FALSE |
| SRX186830 | TFs and others | ESR1 | Breast | Breast cancer | 740 | 21/31605 | -1.46/0.26 | -9.0 | -8.8 | -6.12 | FALSE |
| SRX13993201 | TFs and others | HIF1A | Breast | MB 157 | 454 | 100/31605 | -0.53/0.26 | -9.0 | -8.8 | -6.12 | FALSE |
| SRX17767564 | TFs and others | Epitope tags | Breast | MCF-7 | 1293 | 119/31605 | -0.46/0.26 | -9.0 | -8.8 | -6.11 | FALSE |
| SRX4671312 | TFs and others | RARA | Breast | SUM 159PT | 3678 | 650/31605 | -0.05/0.26 | -9.0 | -8.8 | -6.11 | FALSE |
| SRX7130716 | TFs and others | CTCF | Breast | MCF 10A | 10706 | 2082/31605 | 0.08/0.26 | -9.0 | -8.8 | -6.11 | FALSE |
| SRX10024480 | TFs and others | FOXA1 | Breast | MCF-7 | 11085 | 1013/31605 | 0.01/0.26 | -9.0 | -8.8 | -6.11 | FALSE |
| SRX4096870 | TFs and others | GATAD2B | Breast | HCC3153 | 1491 | 174/31605 | -0.34/0.26 | -9.0 | -8.8 | -6.11 | FALSE |
| SRX2424192 | TFs and others | NBN | Breast | MCF-7 | 3267 | 351/31605 | -0.16/0.26 | -9.0 | -8.8 | -6.11 | FALSE |
| SRX647372 | TFs and others | ZBTB17 | Breast | MCF 10A | 1190 | 378/31605 | -0.15/0.26 | -9.0 | -8.8 | -6.11 | FALSE |
| SRX4786546 | TFs and others | TET2 | Breast | MCF-7 | 1161 | 349/31605 | -0.16/0.26 | -9.0 | -8.8 | -6.09 | FALSE |
| SRX194571 | TFs and others | GREB1 | Breast | MCF-7 | 2520 | 236/31605 | -0.25/0.26 | -8.9 | -8.8 | -6.09 | FALSE |
| SRX3541116 | TFs and others | SUMO2 | Breast | MCF-7 | 1322 | 69/31605 | -0.68/0.26 | -8.9 | -8.7 | -6.08 | FALSE |
| SRX15929737 | TFs and others | STAG2 | Breast | MCF 10A | 9852 | 2427/31605 | 0.10/0.26 | -8.9 | -8.7 | -6.08 | FALSE |
| SRX16874136 | TFs and others | ESR1 | Breast | MCF-7 | 799 | 15/31605 | -1.76/0.26 | -8.9 | -8.7 | -6.08 | FALSE |
| SRX13255929 | TFs and others | Epitope tags | Breast | Breast cancer cells | 2681 | 836/31605 | -0.01/0.26 | -8.9 | -8.7 | -6.08 | FALSE |
| SRX18454202 | TFs and others | BRD4 | Breast | MDA-MB-231 | 1502 | 359/31605 | -0.16/0.26 | -8.9 | -8.7 | -6.08 | FALSE |
| SRX5534862 | TFs and others | SMARCA4 | Breast | MCF-7 | 874 | 558/31605 | -0.07/0.26 | -8.9 | -8.7 | -6.07 | FALSE |
| SRX19024878 | TFs and others | NR3C1 | Breast | MCF-7 | 10333 | 573/31605 | -0.07/0.26 | -8.9 | -8.7 | -6.07 | FALSE |
| SRX5794311 | TFs and others | TCF7 | Breast | Breast organoids | 8344 | 2042/31605 | 0.08/0.26 | -8.9 | -8.7 | -6.06 | FALSE |
| SRX2424140 | TFs and others | MLLT1 | Breast | MCF-7 | 1712 | 307/31605 | -0.19/0.26 | -8.9 | -8.7 | -6.06 | FALSE |
| SRX10977369 | TFs and others | ZNF331 | Breast | MCF-7 | 1375 | 105/31605 | -0.50/0.26 | -8.9 | -8.7 | -6.06 | FALSE |
| SRX107364 | TFs and others | ESR1 | Breast | H3396 | 4338 | 275/31605 | -0.21/0.26 | -8.9 | -8.7 | -6.05 | FALSE |
| SRX3734314 | TFs and others | TP63 | Breast | Mammary glands | 33648 | 2983/31605 | 0.11/0.26 | -8.8 | -8.7 | -6.05 | FALSE |
| SRX14101920 | TFs and others | KAT2B | Breast | MCF-7 | 2650 | 77/31605 | -0.63/0.26 | -8.8 | -8.6 | -6.05 | FALSE |
| SRX2578917 | TFs and others | EP300 | Breast | MDA-MB-231 | 855 | 45/31605 | -0.90/0.26 | -8.8 | -8.6 | -6.04 | FALSE |
| SRX2924014 | TFs and others | TP53 | Breast | MCF-7 | 4780 | 580/31605 | -0.07/0.26 | -8.8 | -8.6 | -6.04 | FALSE |
| SRX5128109 | TFs and others | AR | Breast | ZR-75-1 | 9749 | 609/31605 | -0.06/0.26 | -8.8 | -8.6 | -6.02 | FALSE |
| SRX5457543 | TFs and others | ZNF507 | Breast | MCF-7 | 1568 | 402/31605 | -0.13/0.26 | -8.7 | -8.5 | -6.01 | FALSE |
| SRX3401278 | TFs and others | HDAC3 | Breast | MCF-7 | 782 | 23/31605 | -1.34/0.26 | -8.6 | -8.4 | -5.96 | FALSE |
| SRX20830469 | TFs and others | PGR | Breast | T-47D | 445 | 11/31605 | -2.05/0.26 | -8.6 | -8.4 | -5.96 | FALSE |
| SRX8084747 | TFs and others | Epitope tags | Breast | SUM 159PT | 1583 | 568/31605 | -0.06/0.26 | -8.6 | -8.4 | -5.95 | FALSE |
| SRX7030917 | TFs and others | TRIM28 | Breast | MCF-7 | 2580 | 370/31605 | -0.14/0.26 | -8.6 | -8.4 | -5.95 | FALSE |
| SRX5457503 | TFs and others | ZNF217 | Breast | MCF-7 | 1555 | 85/31605 | -0.57/0.26 | -8.5 | -8.3 | -5.94 | FALSE |
| SRX7659598 | TFs and others | C17orf49 | Breast | MCF-7 | 862 | 369/31605 | -0.14/0.26 | -8.5 | -8.3 | -5.93 | FALSE |
| SRX8084748 | TFs and others | Epitope tags | Breast | SUM 159PT | 1597 | 434/31605 | -0.11/0.26 | -8.5 | -8.3 | -5.93 | FALSE |
| SRX1070160 | TFs and others | AR | Breast | MDA-MB-453 | 14278 | 732/31605 | -0.03/0.26 | -8.5 | -8.3 | -5.92 | FALSE |
| SRX3285591 | TFs and others | KDM5B | Breast | FULR | 1700 | 673/31605 | -0.04/0.26 | -8.5 | -8.3 | -5.92 | FALSE |
| SRX7122006 | TFs and others | FOXA1 | Breast | MCF-7 | 2627 | 310/31605 | -0.18/0.26 | -8.5 | -8.3 | -5.92 | FALSE |
| SRX10045332 | TFs and others | ASXL1 | Breast | CAL-51 | 1178 | 228/31605 | -0.25/0.26 | -8.5 | -8.3 | -5.92 | FALSE |
| SRX6487042 | TFs and others | FOXA1 | Breast | MCF-7 | 7690 | 633/31605 | -0.05/0.26 | -8.5 | -8.3 | -5.91 | FALSE |
| SRX1339017 | TFs and others | AR | Breast | MDA-MB-453 | 11506 | 676/31605 | -0.04/0.26 | -8.5 | -8.3 | -5.91 | FALSE |
| SRX4192887 | TFs and others | STAT3 | Breast | MCF 10A | 9384 | 1277/31605 | 0.04/0.26 | -8.4 | -8.2 | -5.89 | FALSE |
| SRX3025917 | TFs and others | TP53 | Breast | MCF-7 | 1200 | 14/31605 | -1.76/0.26 | -8.4 | -8.2 | -5.89 | FALSE |
| SRX747791 | TFs and others | FOXA1 | Breast | MCF-7 | 10675 | 887/31605 | 0.00/0.26 | -8.4 | -8.2 | -5.88 | FALSE |
| SRX3322004 | TFs and others | SNIP1 | Breast | MCF-7 | 2016 | 383/31605 | -0.13/0.26 | -8.4 | -8.2 | -5.88 | FALSE |
| SRX3958390 | TFs and others | WDR5 | Breast | Breast cancer cells | 2456 | 265/31605 | -0.21/0.26 | -8.4 | -8.2 | -5.88 | FALSE |
| SRX6487046 | TFs and others | FOXA1 | Breast | MCF-7 | 5028 | 387/31605 | -0.13/0.26 | -8.4 | -8.2 | -5.87 | FALSE |
| SRX19888699 | TFs and others | SNAI2 | Breast | BT-549 | 5985 | 2251/31605 | 0.10/0.26 | -8.4 | -8.2 | -5.87 | FALSE |
| SRX7366775 | TFs and others | KDM5C | Breast | MCF-7 | 826 | 190/31605 | -0.29/0.26 | -8.4 | -8.2 | -5.87 | FALSE |
| SRX2871362 | TFs and others | PGR | Breast | T-47D | 1210 | 187/31605 | -0.29/0.26 | -8.3 | -8.2 | -5.86 | FALSE |
| SRX5493750 | TFs and others | AR | Breast | Breast cancer cells | 671 | 15/31605 | -1.68/0.26 | -8.3 | -8.1 | -5.86 | FALSE |
| SRX6487054 | TFs and others | FOXA1 | Breast | MCF-7 | 9871 | 797/31605 | -0.01/0.26 | -8.3 | -8.1 | -5.86 | FALSE |
| SRX5287707 | TFs and others | TEAD4 | Breast | MCF-7 | 2029 | 403/31605 | -0.12/0.26 | -8.3 | -8.1 | -5.85 | FALSE |
| SRX3322338 | TFs and others | SMARCE1 | Breast | MCF-7 | 5516 | 520/31605 | -0.07/0.26 | -8.3 | -8.1 | -5.84 | FALSE |
| SRX6487037 | TFs and others | FOXA1 | Breast | MCF-7 | 3632 | 272/31605 | -0.20/0.26 | -8.3 | -8.1 | -5.84 | FALSE |
| SRX13991432 | TFs and others | CTNNB1 | Breast | MCF 10A | 977 | 301/31605 | -0.18/0.26 | -8.3 | -8.1 | -5.84 | FALSE |
| SRX1143683 | TFs and others | TP63 | Breast | MCF10DCIS.com | 12172 | 1361/31605 | 0.05/0.26 | -8.3 | -8.1 | -5.84 | FALSE |
| SRX14746768 | TFs and others | EPAS1 | Breast | T-47D | 821 | 45/31605 | -0.86/0.26 | -8.3 | -8.1 | -5.84 | FALSE |
| SRX9688337 | TFs and others | SP1 | Breast | ZR-75-1 | 1285 | 256/31605 | -0.21/0.26 | -8.3 | -8.1 | -5.83 | FALSE |
| SRX1339018 | TFs and others | AR | Breast | MDA-MB-453 | 16838 | 973/31605 | 0.02/0.26 | -8.2 | -8.1 | -5.83 | FALSE |
| SRX5089438 | TFs and others | SMARCB1 | Breast | MCF-7 | 5001 | 441/31605 | -0.10/0.26 | -8.2 | -8.1 | -5.82 | FALSE |
| SRX4671298 | TFs and others | RARA | Breast | NAMEC8 | 1254 | 236/31605 | -0.23/0.26 | -8.2 | -8.0 | -5.82 | FALSE |
| SRX10119366 | TFs and others | YAP1 | Breast | MCF 10A | 13510 | 1574/31605 | 0.07/0.26 | -8.2 | -8.0 | -5.81 | FALSE |
| SRX176865 | TFs and others | GATA3 | Breast | MCF-7 | 4753 | 330/31605 | -0.15/0.26 | -8.2 | -8.0 | -5.81 | FALSE |
| SRX11867607 | TFs and others | KDM6A | Breast | CAL-51 | 1844 | 387/31605 | -0.12/0.26 | -8.2 | -8.0 | -5.80 | FALSE |
| SRX7586662 | TFs and others | SOX4 | Breast | BT-549 | 353 | 151/31605 | -0.35/0.26 | -8.2 | -8.0 | -5.79 | FALSE |
| SRX1161179 | TFs and others | NR3C1 | Breast | ZR-75-1 | 3487 | 164/31605 | -0.32/0.26 | -8.1 | -8.0 | -5.79 | FALSE |
| SRX13991441 | TFs and others | TCF4 | Breast | MCF 10A | 879 | 299/31605 | -0.17/0.26 | -8.1 | -8.0 | -5.79 | FALSE |
| SRX10119363 | TFs and others | YAP1 | Breast | MCF 10A | 23982 | 2256/31605 | 0.10/0.26 | -8.1 | -8.0 | -5.78 | FALSE |
| SRX7644284 | TFs and others | CPSF3L | Breast | MCF-7 | 641 | 179/31605 | -0.30/0.26 | -8.1 | -8.0 | -5.78 | FALSE |
| SRX3011695 | TFs and others | JMJD6 | Breast | MCF-7 | 583 | 36/31605 | -0.98/0.26 | -8.1 | -7.9 | -5.78 | FALSE |
| SRX3229126 | TFs and others | FOXA1 | Breast | Breast cancer | 6906 | 620/31605 | -0.04/0.26 | -8.1 | -7.9 | -5.78 | FALSE |
| SRX10775027 | TFs and others | RNF2 | Breast | T-47D | 1445 | 25/31605 | -1.22/0.26 | -8.1 | -7.9 | -5.76 | FALSE |
| SRX8617942 | TFs and others | FGFR1 | Breast | CAMA-1 | 1927 | 355/31605 | -0.14/0.26 | -8.1 | -7.9 | -5.76 | FALSE |
| SRX5534842 | TFs and others | FOXA1 | Breast | MCF-7 | 14726 | 1135/31605 | 0.04/0.26 | -8.1 | -7.9 | -5.75 | FALSE |
| SRX20282956 | TFs and others | AHR | Breast | MCF-7 | 812 | 71/31605 | -0.62/0.26 | -8.0 | -7.9 | -5.75 | FALSE |
| SRX6858249 | TFs and others | HSF1 | Breast | MCF-7 | 1391 | 291/31605 | -0.18/0.26 | -8.0 | -7.8 | -5.74 | FALSE |
| SRX2924017 | TFs and others | TP53 | Breast | MCF-7 | 3858 | 489/31605 | -0.08/0.26 | -8.0 | -7.8 | -5.72 | FALSE |
| SRX3541112 | TFs and others | SUMO2 | Breast | MCF-7 | 1800 | 142/31605 | -0.36/0.26 | -8.0 | -7.8 | -5.72 | FALSE |
| SRX6487063 | TFs and others | FOXA1 | Breast | MCF-7 | 2832 | 183/31605 | -0.29/0.26 | -8.0 | -7.8 | -5.72 | FALSE |
| SRX18191679 | TFs and others | ESR1 | Breast | MCF-7 | 898 | 16/31605 | -1.57/0.26 | -8.0 | -7.8 | -5.72 | FALSE |
| SRX5534871 | TFs and others | JUN | Breast | MCF-7 | 1213 | 364/31605 | -0.13/0.26 | -7.9 | -7.7 | -5.70 | FALSE |
| SRX4096884 | TFs and others | TRPS1 | Breast | HCC3153 | 7313 | 791/31605 | -0.00/0.26 | -7.9 | -7.7 | -5.70 | FALSE |
| SRX392294 | TFs and others | PRKCQ | Breast | MCF-7 | 766 | 185/31605 | -0.28/0.26 | -7.9 | -7.7 | -5.68 | FALSE |
| SRX19321945 | TFs and others | RNF2 | Breast | MCF-7 | 2314 | 977/31605 | 0.49/0.26 | -7.8 | -7.7 | 5.67 | TRUE |
| SRX883579 | TFs and others | WWTR1 | Breast | MDA-MB-231 | 9239 | 923/31605 | 0.02/0.26 | -7.8 | -7.7 | -5.67 | FALSE |
| SRX13845683 | TFs and others | HSF1 | Breast | MDA-MB-231 | 1091 | 589/31605 | -0.04/0.26 | -7.8 | -7.7 | -5.67 | FALSE |
| SRX13254862 | TFs and others | SUZ12 | Breast | MCF-7 | 45673 | 5479/31605 | 0.16/0.26 | -7.8 | -7.6 | -5.66 | FALSE |
| SRX10775018 | TFs and others | ESR1 | Breast | T-47D | 1312 | 19/31605 | -1.41/0.26 | -7.8 | -7.6 | -5.66 | FALSE |
| SRX1012721 | TFs and others | PGR | Breast | T-47D | 3773 | 417/31605 | -0.10/0.26 | -7.8 | -7.6 | -5.66 | FALSE |
| SRX8617944 | TFs and others | FGFR1 | Breast | CAMA-1 | 1843 | 452/31605 | -0.09/0.26 | -7.8 | -7.6 | -5.66 | FALSE |
| SRX10024491 | TFs and others | FOXA1 | Breast | MCF-7 | 13227 | 1179/31605 | 0.04/0.26 | -7.8 | -7.6 | -5.66 | FALSE |
| SRX19662633 | TFs and others | MYC | Breast | T-47D | 332 | 238/31605 | -0.21/0.26 | -7.8 | -7.6 | -5.66 | FALSE |
| SRX10184540 | TFs and others | GATA3 | Breast | HCC1954 | 6425 | 926/31605 | 0.02/0.26 | -7.8 | -7.6 | -5.66 | FALSE |
| SRX10977370 | TFs and others | ZNF331 | Breast | MCF-7 | 1767 | 108/31605 | -0.44/0.26 | -7.8 | -7.6 | -5.65 | FALSE |
| SRX10641822 | TFs and others | TP63 | Breast | SUM 159PT | 10087 | 1302/31605 | 0.05/0.26 | -7.8 | -7.6 | -5.65 | FALSE |
| SRX4671300 | TFs and others | EP300 | Breast | NAMEC8 | 5071 | 539/31605 | -0.06/0.26 | -7.8 | -7.6 | -5.65 | FALSE |
| SRX457212 | TFs and others | ESR1 | Breast | MCF-7 | 379 | 11/31605 | -1.92/0.26 | -7.8 | -7.6 | -5.64 | FALSE |
| SRX1161197 | TFs and others | NR3C1 | Breast | T-47D | 1844 | 62/31605 | -0.66/0.26 | -7.8 | -7.6 | -5.64 | FALSE |
| SRX3322664 | TFs and others | NFIB | Breast | MCF-7 | 9646 | 833/31605 | 0.01/0.26 | -7.7 | -7.6 | -5.63 | FALSE |
| SRX10775020 | TFs and others | RNF2 | Breast | T-47D | 1447 | 38/31605 | -0.91/0.26 | -7.7 | -7.6 | -5.63 | FALSE |
| SRX3993991 | TFs and others | TRIM24 | Breast | MCF-7 | 437 | 21/31605 | -1.32/0.26 | -7.7 | -7.6 | -5.63 | FALSE |
| SRX2915389 | TFs and others | PGR | Breast | T-47D | 6021 | 591/31605 | -0.04/0.26 | -7.7 | -7.5 | -5.62 | FALSE |
| SRX11374763 | TFs and others | PGR | Breast | T-47D | 4799 | 247/31605 | -0.20/0.26 | -7.7 | -7.5 | -5.62 | FALSE |
| SRX3229175 | TFs and others | FOXA1 | Breast | Breast cancer | 5956 | 584/31605 | -0.04/0.26 | -7.7 | -7.5 | -5.61 | FALSE |
| SRX13327994 | TFs and others | CBX3 | Breast | MCF-7 | 1244 | 99/31605 | -0.47/0.26 | -7.7 | -7.5 | -5.61 | FALSE |
| SRX15039985 | TFs and others | CHD4 | Breast | T-47D | 2472 | 762/31605 | -0.00/0.26 | -7.7 | -7.5 | -5.60 | FALSE |
| SRX4096885 | TFs and others | TRPS1 | Breast | HCC3153 | 8238 | 791/31605 | 0.00/0.26 | -7.7 | -7.5 | -5.60 | FALSE |
| SRX10775046 | TFs and others | ESR1 | Breast | T-47D | 948 | 18/31605 | -1.43/0.26 | -7.7 | -7.5 | -5.59 | FALSE |
| SRX9697820 | TFs and others | Epitope tags | Breast | MDA-MB-231 | 2287 | 398/31605 | -0.10/0.26 | -7.6 | -7.5 | -5.59 | FALSE |
| SRX6858247 | TFs and others | HSF1 | Breast | MCF-7 | 1456 | 306/31605 | -0.15/0.26 | -7.6 | -7.5 | -5.59 | FALSE |
| SRX594728 | TFs and others | PGR | Breast | ZR-75-1 | 2305 | 251/31605 | -0.20/0.26 | -7.6 | -7.4 | -5.58 | FALSE |
| SRX7405964 | TFs and others | ESR1 | Breast | ZR-75-1 | 2298 | 62/31605 | -0.65/0.26 | -7.6 | -7.4 | -5.57 | FALSE |
| SRX2424353 | TFs and others | SP1 | Breast | MCF-7 | 1545 | 309/31605 | -0.15/0.26 | -7.6 | -7.4 | -5.56 | FALSE |
| SRX323403 | TFs and others | SPDEF | Breast | MCF-7 | 561 | 117/31605 | -0.40/0.26 | -7.6 | -7.4 | -5.56 | FALSE |
| SRX1709978 | TFs and others | ESR1 | Breast | T-47D | 724 | 14/31605 | -1.65/0.26 | -7.6 | -7.4 | -5.56 | FALSE |
| SRX5287699 | TFs and others | MED1 | Breast | MCF-7 | 756 | 152/31605 | -0.32/0.26 | -7.6 | -7.4 | -5.56 | FALSE |
| SRX176869 | TFs and others | GATA3 | Breast | MCF-7 | 3314 | 233/31605 | -0.21/0.26 | -7.5 | -7.3 | -5.54 | FALSE |
| SRX19321949 | TFs and others | RNF2 | Breast | MCF-7 | 3559 | 1370/31605 | 0.45/0.26 | -7.5 | -7.3 | 5.52 | TRUE |
| SRX5457179 | TFs and others | MAFK | Breast | MCF-7 | 6244 | 530/31605 | -0.05/0.26 | -7.5 | -7.3 | -5.52 | FALSE |
| SRX10205611 | TFs and others | SMARCA4 | Breast | CAL-120 | 2962 | 569/31605 | -0.04/0.26 | -7.5 | -7.3 | -5.52 | FALSE |
| SRX1489433 | TFs and others | MBD3 | Breast | MDA-MB-231 | 4970 | 637/31605 | -0.02/0.26 | -7.4 | -7.3 | -5.51 | FALSE |
| SRX5287708 | TFs and others | YAP1 | Breast | MCF-7 | 1766 | 152/31605 | -0.32/0.26 | -7.4 | -7.3 | -5.51 | FALSE |
| SRX673713 | TFs and others | Biotin | Breast | MCF-7 | 400 | 15/31605 | -1.57/0.26 | -7.4 | -7.3 | -5.51 | FALSE |
| SRX3800729 | TFs and others | RAD21 | Breast | T-47D | 4100 | 1090/31605 | 0.04/0.26 | -7.4 | -7.2 | -5.50 | FALSE |
| SRX5128108 | TFs and others | AR | Breast | ZR-75-1 | 8127 | 456/31605 | -0.07/0.26 | -7.4 | -7.2 | -5.50 | FALSE |
| SRX10142197 | TFs and others | CTCF | Breast | Mammary epithelial cells | 3883 | 1227/31605 | 0.05/0.26 | -7.4 | -7.2 | -5.50 | FALSE |
| SRX14746767 | TFs and others | EPAS1 | Breast | T-47D | 741 | 34/31605 | -0.95/0.26 | -7.4 | -7.2 | -5.49 | FALSE |
| SRX22997292 | TFs and others | MAFG | Breast | SK-BR-3 | 4790 | 577/31605 | -0.04/0.26 | -7.4 | -7.2 | -5.47 | FALSE |
| SRX5401865 | TFs and others | PGR | Breast | T-47D | 3820 | 141/31605 | -0.33/0.26 | -7.3 | -7.1 | -5.45 | FALSE |
| SRX2424058 | TFs and others | FOXK2 | Breast | MCF-7 | 1919 | 340/31605 | -0.12/0.26 | -7.3 | -7.1 | -5.44 | FALSE |
| SRX3025914 | TFs and others | TP53 | Breast | MCF-7 | 1159 | 16/31605 | -1.48/0.26 | -7.3 | -7.1 | -5.43 | FALSE |
| SRX1044413 | TFs and others | JUN | Breast | MDA-MB-231 | 15083 | 2031/31605 | 0.10/0.26 | -7.3 | -7.1 | -5.43 | FALSE |
| SRX8850930 | TFs and others | Epitope tags | Breast | MCF 10A | 882 | 136/31605 | -0.34/0.26 | -7.2 | -7.1 | -5.43 | FALSE |
| SRX3833324 | TFs and others | KDM1A | Breast | MCF-7 | 753 | 161/31605 | -0.29/0.26 | -7.2 | -7.1 | -5.43 | FALSE |
| SRX4116053 | TFs and others | FOXA1 | Breast | Breast cancer cells | 6635 | 619/31605 | -0.02/0.26 | -7.2 | -7.0 | -5.42 | FALSE |
| SRX10119396 | TFs and others | JUNB | Breast | MDA-MB-231 | 43502 | 3684/31605 | 0.14/0.26 | -7.2 | -7.0 | -5.41 | FALSE |
| SRX176871 | TFs and others | GATA3 | Breast | MCF-7 | 5842 | 283/31605 | -0.16/0.26 | -7.2 | -7.0 | -5.41 | FALSE |
| SRX13327963 | TFs and others | CBX3 | Breast | MCF-7 | 1825 | 248/31605 | -0.18/0.26 | -7.2 | -7.0 | -5.40 | FALSE |
| SRX6487035 | TFs and others | FOXA1 | Breast | MCF-7 | 7433 | 617/31605 | -0.02/0.26 | -7.2 | -7.0 | -5.39 | FALSE |
| SRX3229169 | TFs and others | AR | Breast | Breast cancer | 699 | 16/31605 | -1.47/0.26 | -7.2 | -7.0 | -5.39 | FALSE |
| SRX20830463 | TFs and others | Epitope tags | Breast | T-47D | 1638 | 38/31605 | -0.86/0.26 | -7.1 | -7.0 | -5.39 | FALSE |
| SRX19321944 | TFs and others | RNF2 | Breast | MCF-7 | 2086 | 1100/31605 | 0.46/0.26 | -7.1 | -6.9 | 5.37 | TRUE |
| SRX2880133 | TFs and others | KDM6A | Breast | CAL-51 | 1012 | 231/31605 | -0.20/0.26 | -7.1 | -6.9 | -5.36 | FALSE |
| SRX18191678 | TFs and others | ESR1 | Breast | MCF-7 | 910 | 16/31605 | -1.46/0.26 | -7.0 | -6.9 | -5.34 | FALSE |
| SRX19321947 | TFs and others | RNF2 | Breast | MCF-7 | 1674 | 892/31605 | 0.48/0.26 | -7.0 | -6.9 | 5.34 | TRUE |
| SRX4896695 | TFs and others | RUNX1 | Breast | MCF 10A | 2072 | 388/31605 | -0.09/0.26 | -7.0 | -6.8 | -5.33 | FALSE |
| SRX5401860 | TFs and others | NR3C1 | Breast | T-47D | 16384 | 1348/31605 | 0.07/0.26 | -7.0 | -6.8 | -5.33 | FALSE |
| SRX10773087 | TFs and others | EBF1 | Breast | MB 157 | 1063 | 294/31605 | -0.14/0.26 | -7.0 | -6.8 | -5.32 | FALSE |
| SRX9109112 | TFs and others | AR | Breast | MDA-MB-453 | 9285 | 658/31605 | -0.01/0.26 | -7.0 | -6.8 | -5.31 | FALSE |
| SRX5089539 | TFs and others | ARID2 | Breast | MCF-7 | 1020 | 22/31605 | -1.19/0.26 | -7.0 | -6.8 | -5.31 | FALSE |
| SRX1012709 | TFs and others | ESR1 | Breast | T-47D | 1536 | 37/31605 | -0.86/0.26 | -7.0 | -6.8 | -5.31 | FALSE |
| SRX2947200 | TFs and others | RBPJ | Breast | MDA-MB-231 | 2952 | 300/31605 | -0.14/0.26 | -6.9 | -6.8 | -5.30 | FALSE |
| SRX13327993 | TFs and others | CBX3 | Breast | MCF-7 | 1472 | 153/31605 | -0.29/0.26 | -6.9 | -6.8 | -5.30 | FALSE |
| SRX21286684 | TFs and others | TAZ | Breast | MCF 10A | 1371 | 165/31605 | -0.27/0.26 | -6.9 | -6.7 | -5.29 | FALSE |
| SRX2423941 | TFs and others | ZKSCAN1 | Breast | MCF-7 | 1918 | 236/31605 | -0.19/0.26 | -6.9 | -6.7 | -5.29 | FALSE |
| SRX4717810 | TFs and others | TCEA2 | Breast | HMLE | 1174 | 186/31605 | -0.24/0.26 | -6.9 | -6.7 | -5.29 | FALSE |
| SRX15337042 | TFs and others | Epitope tags | Breast | T-47D | 700 | 25/31605 | -1.10/0.26 | -6.9 | -6.7 | -5.29 | FALSE |
| SRX4060992 | TFs and others | TRPS1 | Breast | T-47D | 1321 | 100/31605 | -0.42/0.26 | -6.9 | -6.7 | -5.29 | FALSE |
| SRX3322823 | TFs and others | PAX8 | Breast | MCF-7 | 2840 | 240/31605 | -0.18/0.26 | -6.9 | -6.7 | -5.28 | FALSE |
| SRX3322568 | TFs and others | FOSL2 | Breast | MCF-7 | 5563 | 634/31605 | -0.01/0.26 | -6.9 | -6.7 | -5.27 | FALSE |
| SRX4896696 | TFs and others | RUNX1 | Breast | MCF 10A | 2148 | 158/31605 | -0.28/0.26 | -6.8 | -6.7 | -5.26 | FALSE |
| SRX1772411 | TFs and others | Epitope tags | Breast | MCF-7 | 815 | 123/31605 | -0.35/0.26 | -6.8 | -6.7 | -5.26 | FALSE |
| SRX15430990 | TFs and others | TFE3 | Breast | MDA-MB-453 | 9377 | 583/31605 | -0.02/0.26 | -6.8 | -6.6 | -5.25 | FALSE |
| SRX14138492 | TFs and others | Epitope tags | Breast | MCF-7 | 1617 | 215/31605 | -0.20/0.26 | -6.8 | -6.6 | -5.25 | FALSE |
| SRX15826244 | TFs and others | KMT2D | Breast | MDA-MB-231 | 8086 | 1079/31605 | 0.05/0.26 | -6.8 | -6.6 | -5.25 | FALSE |
| SRX5057622 | TFs and others | GATA3 | Breast | T-47D | 2729 | 301/31605 | -0.13/0.26 | -6.8 | -6.6 | -5.24 | FALSE |
| SRX2578874 | TFs and others | ESR1 | Breast | MDA-MB-231 | 223 | 34/31605 | -0.90/0.26 | -6.8 | -6.6 | -5.24 | FALSE |
| SRX13254865 | TFs and others | SUZ12 | Breast | MCF-7 | 19227 | 1962/31605 | 0.10/0.26 | -6.8 | -6.6 | -5.24 | FALSE |
| SRX647369 | TFs and others | ZBTB17 | Breast | MCF 10A | 1117 | 329/31605 | -0.11/0.26 | -6.8 | -6.6 | -5.23 | FALSE |
| SRX18013267 | TFs and others | STAG1 | Breast | MCF 10A | 18269 | 2424/31605 | 0.12/0.26 | -6.8 | -6.6 | -5.22 | FALSE |
| SRX12813813 | TFs and others | TRIM28 | Breast | MCF-7 | 5746 | 185/31605 | -0.24/0.26 | -6.8 | -6.6 | -5.22 | FALSE |
| SRX2924013 | TFs and others | TP53 | Breast | MCF-7 | 3391 | 435/31605 | -0.07/0.26 | -6.7 | -6.6 | -5.22 | FALSE |
| SRX107365 | TFs and others | ESR1 | Breast | H3396 | 2502 | 143/31605 | -0.30/0.26 | -6.7 | -6.6 | -5.21 | FALSE |
| SRX1012738 | TFs and others | ESR1 | Breast | MCF-7 | 620 | 39/31605 | -0.81/0.26 | -6.7 | -6.6 | -5.21 | FALSE |
| SRX5824581 | TFs and others | BRD4 | Breast | SUM 159PT | 24851 | 1610/31605 | 0.09/0.26 | -6.7 | -6.5 | -5.21 | FALSE |
| SRX5457502 | TFs and others | ZNF217 | Breast | MCF-7 | 1691 | 65/31605 | -0.57/0.26 | -6.7 | -6.5 | -5.20 | FALSE |
| SRX5534864 | TFs and others | EP300 | Breast | MCF-7 | 639 | 198/31605 | -0.22/0.26 | -6.7 | -6.5 | -5.20 | FALSE |
| SRX5089432 | TFs and others | SMARCB1 | Breast | MCF-7 | 2886 | 275/31605 | -0.14/0.26 | -6.7 | -6.5 | -5.18 | FALSE |
| SRX10365546 | TFs and others | NCOA3 | Breast | MCF-7 | 5015 | 217/31605 | -0.20/0.26 | -6.7 | -6.5 | -5.18 | FALSE |
| SRX8520809 | TFs and others | NR3C1 | Breast | SUM 159PT | 39919 | 3086/31605 | 0.14/0.26 | -6.7 | -6.5 | -5.18 | FALSE |
| SRX5287713 | TFs and others | Biotin | Breast | MCF-7 | 517 | 36/31605 | -0.85/0.26 | -6.6 | -6.5 | -5.17 | FALSE |
| SRX1156472 | TFs and others | GATA3 | Breast | MDA-MB-231 | 9942 | 1281/31605 | 0.07/0.26 | -6.6 | -6.4 | -5.16 | FALSE |
| SRX2871372 | TFs and others | Epitope tags | Breast | T-47D | 1655 | 95/31605 | -0.42/0.26 | -6.6 | -6.4 | -5.15 | FALSE |
| SRX20830471 | TFs and others | PGR | Breast | T-47D | 423 | 9/31605 | -1.94/0.26 | -6.6 | -6.4 | -5.15 | FALSE |
| SRX13991443 | TFs and others | TCF4 | Breast | MCF 10A | 960 | 365/31605 | -0.09/0.26 | -6.6 | -6.4 | -5.15 | FALSE |
| SRX10664188 | TFs and others | JARID2 | Breast | MCF-7 | 6220 | 615/31605 | -0.01/0.26 | -6.6 | -6.4 | -5.15 | FALSE |
| SRX14101919 | TFs and others | KAT2B | Breast | MCF-7 | 2385 | 75/31605 | -0.51/0.26 | -6.6 | -6.4 | -5.14 | FALSE |
| SRX3428652 | TFs and others | ESR1 | Breast | MCF-7 | 705 | 22/31605 | -1.15/0.26 | -6.6 | -6.4 | -5.14 | FALSE |
| SRX5089547 | TFs and others | ARID2 | Breast | MCF-7 | 974 | 23/31605 | -1.12/0.26 | -6.6 | -6.4 | -5.14 | FALSE |
| SRX3541115 | TFs and others | SUMO2 | Breast | MCF-7 | 1628 | 99/31605 | -0.41/0.26 | -6.6 | -6.4 | -5.14 | FALSE |
| SRX4717807 | TFs and others | TCEA2 | Breast | HMLE | 973 | 243/31605 | -0.17/0.26 | -6.6 | -6.4 | -5.14 | FALSE |
| SRX2900092 | TFs and others | BAP1 | Breast | CAL-51 | 644 | 17/31605 | -1.34/0.26 | -6.6 | -6.4 | -5.14 | FALSE |
| SRX3025913 | TFs and others | TP53 | Breast | MCF-7 | 2248 | 196/31605 | -0.21/0.26 | -6.5 | -6.4 | -5.13 | FALSE |
| SRX3322520 | TFs and others | ELF1 | Breast | MCF-7 | 1091 | 89/31605 | -0.44/0.26 | -6.5 | -6.4 | -5.13 | FALSE |
| SRX6487040 | TFs and others | FOXA1 | Breast | MCF-7 | 1778 | 137/31605 | -0.31/0.26 | -6.5 | -6.4 | -5.13 | FALSE |
| SRX7644275 | TFs and others | METTL3 | Breast | MCF-7 | 560 | 100/31605 | -0.40/0.26 | -6.5 | -6.4 | -5.12 | FALSE |
| SRX10365526 | TFs and others | RAC1 | Breast | MDA-MB-231 | 587 | 9/31605 | -1.93/0.26 | -6.5 | -6.3 | -5.12 | FALSE |
| SRX2424605 | TFs and others | NONO | Breast | MCF-7 | 1583 | 256/31605 | -0.15/0.26 | -6.5 | -6.3 | -5.11 | FALSE |
| SRX19210518 | TFs and others | NFKB2 | Breast | SUM 149PT | 2062 | 624/31605 | -0.01/0.26 | -6.5 | -6.3 | -5.11 | FALSE |
| SRX2423924 | TFs and others | MTA3 | Breast | MCF-7 | 1854 | 226/31605 | -0.18/0.26 | -6.5 | -6.3 | -5.11 | FALSE |
| SRX3040151 | TFs and others | STAG1 | Breast | HMEC | 16549 | 1862/31605 | 0.10/0.26 | -6.5 | -6.3 | -5.10 | FALSE |
| SRX13993204 | TFs and others | HIF1A | Breast | MB 157 | 408 | 63/31605 | -0.57/0.26 | -6.5 | -6.3 | -5.10 | FALSE |
| SRX5128130 | TFs and others | AR | Breast | ZR-75-1 | 4257 | 146/31605 | -0.28/0.26 | -6.4 | -6.3 | -5.08 | FALSE |
| SRX2423592 | TFs and others | HDGF | Breast | MCF-7 | 1721 | 161/31605 | -0.26/0.26 | -6.4 | -6.2 | -5.06 | FALSE |
| SRX14101949 | TFs and others | SNAI2 | Breast | MCF-7 | 2155 | 516/31605 | -0.03/0.26 | -6.4 | -6.2 | -5.06 | FALSE |
| SRX2912045 | TFs and others | ESR1 | Breast | MCF-7 | 453 | 77/31605 | -0.48/0.26 | -6.4 | -6.2 | -5.05 | FALSE |
| SRX4284792 | TFs and others | Epitope tags | Breast | T-47D | 1010 | 13/31605 | -1.54/0.26 | -6.4 | -6.2 | -5.05 | FALSE |
| SRX19321946 | TFs and others | RNF2 | Breast | MCF-7 | 1422 | 793/31605 | 0.48/0.26 | -6.3 | -6.2 | 5.04 | TRUE |
| SRX6831825 | TFs and others | TEAD4 | Breast | MCF 10A | 6035 | 655/31605 | 0.00/0.26 | -6.3 | -6.2 | -5.04 | FALSE |
| SRX15670544 | TFs and others | TFE3 | Breast | MDA-MB-453 | 2447 | 237/31605 | -0.16/0.26 | -6.3 | -6.2 | -5.03 | FALSE |
| SRX3285588 | TFs and others | KDM5B | Breast | C70R | 1105 | 766/31605 | 0.02/0.26 | -6.3 | -6.1 | -5.01 | FALSE |
| SRX647370 | TFs and others | ZBTB17 | Breast | MCF 10A | 867 | 301/31605 | -0.11/0.26 | -6.2 | -6.1 | -5.00 | FALSE |
| SRX15232268 | TFs and others | DNA-RNA hybrid | Breast | MDA-MB-436 | 672 | 203/31605 | -0.19/0.26 | -6.2 | -6.1 | -5.00 | FALSE |
| SRX1044411 | TFs and others | JUN | Breast | MDA-MB-231 | 13689 | 1727/31605 | 0.10/0.26 | -6.2 | -6.0 | -4.99 | FALSE |
| SRX5057629 | TFs and others | GATA3 | Breast | T-47D | 1069 | 125/31605 | -0.32/0.26 | -6.2 | -6.0 | -4.98 | FALSE |
| SRX7130727 | TFs and others | CTCF | Breast | MCF 10A | 5557 | 1088/31605 | 0.06/0.26 | -6.2 | -6.0 | -4.98 | FALSE |
| SRX10164581 | TFs and others | ESR1 | Breast | MCF-7 | 1446 | 14/31605 | -1.45/0.26 | -6.2 | -6.0 | -4.98 | FALSE |
| SRX20830475 | TFs and others | PGR | Breast | T-47D | 318 | 6/31605 | -2.35/0.26 | -6.2 | -6.0 | -4.98 | FALSE |
| SRX6487067 | TFs and others | FOXA1 | Breast | MCF-7 | 2151 | 115/31605 | -0.34/0.26 | -6.2 | -6.0 | -4.98 | FALSE |
| SRX19210557 | TFs and others | EZH2 | Breast | SUM 149PT | 480 | 74/31605 | -0.49/0.26 | -6.2 | -6.0 | -4.98 | FALSE |
| SRX13327972 | TFs and others | XRCC6 | Breast | MCF-7 | 806 | 183/31605 | -0.22/0.26 | -6.2 | -6.0 | -4.97 | FALSE |
| SRX10365545 | TFs and others | NCOA3 | Breast | MCF-7 | 813 | 21/31605 | -1.13/0.26 | -6.2 | -6.0 | -4.97 | FALSE |
| SRX10664184 | TFs and others | SUZ12 | Breast | MCF-7 | 7038 | 831/31605 | 0.03/0.26 | -6.2 | -6.0 | -4.97 | FALSE |
| SRX10119395 | TFs and others | JUNB | Breast | MDA-MB-231 | 40241 | 3520/31605 | 0.15/0.26 | -6.2 | -6.0 | -4.97 | FALSE |
| SRX7098806 | TFs and others | CTCF | Breast | Mammary epithelial cells | 640 | 288/31605 | -0.12/0.26 | -6.2 | -6.0 | -4.96 | FALSE |
| SRX13845684 | TFs and others | HSF1 | Breast | MDA-MB-231 | 815 | 442/31605 | -0.05/0.26 | -6.1 | -6.0 | -4.95 | FALSE |
| SRX1012722 | TFs and others | PGR | Breast | T-47D | 2047 | 184/31605 | -0.21/0.26 | -6.1 | -6.0 | -4.95 | FALSE |
| SRX1012708 | TFs and others | ESR1 | Breast | T-47D | 1234 | 43/31605 | -0.71/0.26 | -6.1 | -5.9 | -4.94 | FALSE |
| SRX10365538 | TFs and others | RAC1 | Breast | MCF-7 | 2163 | 140/31605 | -0.28/0.26 | -6.1 | -5.9 | -4.93 | FALSE |
| SRX9528950 | TFs and others | Epitope tags | Breast | MCF-7 | 1095 | 25/31605 | -1.01/0.26 | -6.1 | -5.9 | -4.93 | FALSE |
| SRX4192131 | TFs and others | XRCC4 | Breast | MCF 10A | 1007 | 170/31605 | -0.23/0.26 | -6.1 | -5.9 | -4.92 | FALSE |
| SRX5493755 | TFs and others | FOXA1 | Breast | Breast cancer cells | 3043 | 408/31605 | -0.06/0.26 | -6.1 | -5.9 | -4.92 | FALSE |
| SRX14311066 | TFs and others | Epitope tags | Breast | MDA-MB-231 | 8987 | 2094/31605 | 0.12/0.26 | -6.1 | -5.9 | -4.92 | FALSE |
| SRX1161178 | TFs and others | FOXA1 | Breast | MCF-7 | 489 | 30/31605 | -0.89/0.26 | -6.0 | -5.9 | -4.91 | FALSE |
| SRX042342 | TFs and others | ESR1 | Breast | MCF-7-LTED | 1088 | 20/31605 | -1.15/0.26 | -6.0 | -5.8 | -4.89 | FALSE |
| SRX14756940 | TFs and others | DNA-RNA hybrid | Breast | MDA-MB-436 | 13144 | 4562/31605 | 0.16/0.26 | -6.0 | -5.8 | -4.88 | FALSE |
| SRX17657162 | TFs and others | FOXC1 | Breast | MDA-MB-468 | 7394 | 775/31605 | 0.03/0.26 | -6.0 | -5.8 | -4.88 | FALSE |
| SRX23330420 | TFs and others | HDAC6 | Breast | MDA-MB-231 | 2781 | 1207/31605 | 0.08/0.26 | -6.0 | -5.8 | -4.88 | FALSE |
| SRX7405963 | TFs and others | ESR1 | Breast | ZR-75-1 | 2189 | 61/31605 | -0.54/0.26 | -5.9 | -5.8 | -4.86 | FALSE |
| SRX15039987 | TFs and others | CHD4 | Breast | MDA-MB-231 | 3118 | 374/31605 | -0.07/0.26 | -5.9 | -5.8 | -4.86 | FALSE |
| SRX10859416 | TFs and others | SMARCE1 | Breast | BT-549 | 2764 | 382/31605 | -0.06/0.26 | -5.9 | -5.8 | -4.86 | FALSE |
| SRX16940293 | TFs and others | MAF | Breast | MCF-7 | 1245 | 100/31605 | -0.37/0.26 | -5.9 | -5.8 | -4.86 | FALSE |
| SRX15574110 | TFs and others | CXXC5 | Breast | MCF-7 | 938 | 193/31605 | -0.19/0.26 | -5.9 | -5.8 | -4.86 | FALSE |
| SRX15039988 | TFs and others | CHD4 | Breast | MDA-MB-231 | 2740 | 282/31605 | -0.11/0.26 | -5.9 | -5.7 | -4.85 | FALSE |
| SRX3321936 | TFs and others | HES1 | Breast | MCF-7 | 2812 | 189/31605 | -0.19/0.26 | -5.8 | -5.7 | -4.82 | FALSE |
| SRX6487036 | TFs and others | FOXA1 | Breast | MCF-7 | 3986 | 331/31605 | -0.08/0.26 | -5.8 | -5.7 | -4.81 | FALSE |
| SRX3025916 | TFs and others | TP53 | Breast | MCF-7 | 2549 | 225/31605 | -0.16/0.26 | -5.8 | -5.7 | -4.81 | FALSE |
| SRX5824586 | TFs and others | BRD4 | Breast | SUM 159PT | 2646 | 105/31605 | -0.34/0.26 | -5.8 | -5.6 | -4.80 | FALSE |
| SRX4096864 | TFs and others | GATAD2A | Breast | HCC3153 | 1470 | 298/31605 | -0.10/0.26 | -5.8 | -5.6 | -4.80 | FALSE |
| SRX13327982 | TFs and others | XRCC6 | Breast | MCF-7 | 695 | 208/31605 | -0.17/0.26 | -5.8 | -5.6 | -4.80 | FALSE |
| SRX2924016 | TFs and others | TP53 | Breast | MCF-7 | 1944 | 239/31605 | -0.14/0.26 | -5.8 | -5.6 | -4.79 | FALSE |
| SRX4671309 | TFs and others | RXRA | Breast | SUM 159PT | 1827 | 249/31605 | -0.13/0.26 | -5.7 | -5.6 | -4.77 | FALSE |
| SRX2424354 | TFs and others | SP1 | Breast | MCF-7 | 1620 | 248/31605 | -0.13/0.26 | -5.7 | -5.6 | -4.77 | FALSE |
| SRX6487059 | TFs and others | FOXA1 | Breast | MCF-7 | 5166 | 351/31605 | -0.07/0.26 | -5.7 | -5.6 | -4.77 | FALSE |
| SRX2423478 | TFs and others | ZNF207 | Breast | MCF-7 | 1773 | 208/31605 | -0.17/0.26 | -5.7 | -5.6 | -4.76 | FALSE |
| SRX100264 | TFs and others | CREBBP | Breast | H3396 | 1768 | 134/31605 | -0.27/0.26 | -5.7 | -5.6 | -4.76 | FALSE |
| SRX8520807 | TFs and others | NR3C1 | Breast | MDA-MB-361 | 3921 | 341/31605 | -0.07/0.26 | -5.7 | -5.5 | -4.76 | FALSE |
| SRX2924015 | TFs and others | TP53 | Breast | MCF-7 | 2689 | 313/31605 | -0.09/0.26 | -5.7 | -5.5 | -4.76 | FALSE |
| SRX10119392 | TFs and others | TEAD1 | Breast | MDA-MB-231 | 6061 | 650/31605 | 0.02/0.26 | -5.7 | -5.5 | -4.75 | FALSE |
| SRX384489 | TFs and others | XBP1 | Breast | MDA-MB-231 | 4252 | 398/31605 | -0.05/0.26 | -5.7 | -5.5 | -4.74 | FALSE |
| SRX19636322 | TFs and others | 5-hmC | Breast | HMLE | 58335 | 5965/31605 | 0.18/0.26 | -5.7 | -5.5 | -4.74 | FALSE |
| SRX4096883 | TFs and others | TRPS1 | Breast | HCC3153 | 8073 | 683/31605 | 0.02/0.26 | -5.7 | -5.5 | -4.74 | FALSE |
| SRX7366784 | TFs and others | CDK9 | Breast | MCF-7 | 671 | 122/31605 | -0.29/0.26 | -5.7 | -5.5 | -4.74 | FALSE |
| SRX5534874 | TFs and others | JUN | Breast | MCF-7 | 7819 | 778/31605 | 0.04/0.26 | -5.7 | -5.5 | -4.73 | FALSE |
| SRX107366 | TFs and others | ESR1 | Breast | H3396 | 4256 | 284/31605 | -0.10/0.26 | -5.6 | -5.5 | -4.72 | FALSE |
| SRX5824597 | TFs and others | BRD4 | Breast | SUM 159PT | 11417 | 620/31605 | 0.01/0.26 | -5.6 | -5.5 | -4.72 | FALSE |
| SRX5089537 | TFs and others | ARID2 | Breast | MCF-7 | 777 | 21/31605 | -1.06/0.26 | -5.6 | -5.4 | -4.71 | FALSE |
| SRX968970 | TFs and others | ESR1 | Breast | MCF-7 | 572 | 56/31605 | -0.55/0.26 | -5.6 | -5.4 | -4.70 | FALSE |
| SRX795230 | TFs and others | BRD4 | Breast | SUM 159PT | 2640 | 101/31605 | -0.34/0.26 | -5.6 | -5.4 | -4.70 | FALSE |
| SRX883582 | TFs and others | TEAD4 | Breast | MDA-MB-231 | 12080 | 1205/31605 | 0.08/0.26 | -5.6 | -5.4 | -4.69 | FALSE |
| SRX15235732 | TFs and others | PRRX1 | Breast | UACC-3199 | 778 | 109/31605 | -0.32/0.26 | -5.6 | -5.4 | -4.69 | FALSE |
| SRX883577 | TFs and others | YAP1 | Breast | MDA-MB-231 | 7447 | 723/31605 | 0.03/0.26 | -5.6 | -5.4 | -4.69 | FALSE |
| SRX730427 | TFs and others | NCAPG | Breast | MCF-7 | 539 | 21/31605 | -1.05/0.26 | -5.6 | -5.4 | -4.68 | FALSE |
| SRX6487060 | TFs and others | FOXA1 | Breast | MCF-7 | 3632 | 252/31605 | -0.12/0.26 | -5.5 | -5.4 | -4.67 | FALSE |
| SRX8850932 | TFs and others | Epitope tags | Breast | MCF 10A | 845 | 127/31605 | -0.28/0.26 | -5.5 | -5.4 | -4.67 | FALSE |
| SRX11168159 | TFs and others | BRCA2 | Breast | MCF-7 | 1654 | 107/31605 | -0.32/0.26 | -5.5 | -5.4 | -4.67 | FALSE |
| SRX3401281 | TFs and others | HDAC3 | Breast | MCF-7 | 679 | 16/31605 | -1.24/0.26 | -5.5 | -5.4 | -4.67 | FALSE |
| SRX1528428 | TFs and others | USP7 | Breast | MDA-MB-231 | 1901 | 39/31605 | -0.70/0.26 | -5.5 | -5.3 | -4.66 | FALSE |
| SRX1044412 | TFs and others | JUN | Breast | MDA-MB-231 | 14377 | 1847/31605 | 0.12/0.26 | -5.5 | -5.3 | -4.65 | FALSE |
| SRX6487047 | TFs and others | FOXA1 | Breast | MCF-7 | 7058 | 522/31605 | -0.01/0.26 | -5.5 | -5.3 | -4.64 | FALSE |
| SRX22997304 | TFs and others | MAFG | Breast | Hs 578T | 4400 | 529/31605 | -0.00/0.26 | -5.5 | -5.3 | -4.64 | FALSE |
| SRX13327978 | TFs and others | XRCC6 | Breast | MCF-7 | 707 | 162/31605 | -0.21/0.26 | -5.5 | -5.3 | -4.64 | FALSE |
| SRX6487068 | TFs and others | FOXA1 | Breast | MCF-7 | 1778 | 101/31605 | -0.33/0.26 | -5.4 | -5.3 | -4.63 | FALSE |
| SRX5503023 | TFs and others | E4F1 | Breast | SUM 159PT | 2110 | 178/31605 | -0.19/0.26 | -5.4 | -5.3 | -4.63 | FALSE |
| SRX13327959 | TFs and others | CBX3 | Breast | MCF-7 | 1242 | 126/31605 | -0.27/0.26 | -5.4 | -5.3 | -4.63 | FALSE |
| SRX2900097 | TFs and others | BAP1 | Breast | CAL-51 | 948 | 121/31605 | -0.28/0.26 | -5.4 | -5.3 | -4.62 | FALSE |
| SRX8083101 | TFs and others | FGFR1 | Breast | CAMA-1 | 1210 | 138/31605 | -0.25/0.26 | -5.4 | -5.2 | -4.61 | FALSE |
| SRX8084741 | TFs and others | Epitope tags | Breast | SUM 159PT | 1100 | 240/31605 | -0.12/0.26 | -5.4 | -5.2 | -4.60 | FALSE |
| SRX8716243 | TFs and others | AGO1 | Breast | MCF-7 | 1595 | 16/31605 | -1.21/0.26 | -5.4 | -5.2 | -4.59 | FALSE |
| SRX10119393 | TFs and others | STAT3 | Breast | MDA-MB-231 | 1351 | 293/31605 | -0.09/0.26 | -5.3 | -5.2 | -4.59 | FALSE |
| SRX19210520 | TFs and others | NFKB2 | Breast | SUM 149PT | 1164 | 343/31605 | -0.06/0.26 | -5.3 | -5.2 | -4.58 | FALSE |
| DRX013210 | TFs and others | CTCF | Breast | MCF-7 | 14150 | 1766/31605 | 0.12/0.26 | -5.3 | -5.2 | -4.57 | FALSE |
| SRX6487070 | TFs and others | FOXA1 | Breast | MCF-7 | 2090 | 163/31605 | -0.20/0.26 | -5.3 | -5.1 | -4.56 | FALSE |
| SRX10859403 | TFs and others | ARID2 | Breast | BT-549 | 1640 | 618/31605 | 0.02/0.26 | -5.3 | -5.1 | -4.56 | FALSE |
| SRX11149854 | TFs and others | AR | Breast | MFM-223 | 1455 | 28/31605 | -0.85/0.26 | -5.3 | -5.1 | -4.55 | FALSE |
| SRX19321948 | TFs and others | RNF2 | Breast | MCF-7 | 1719 | 922/31605 | 0.45/0.26 | -5.3 | -5.1 | 4.55 | TRUE |
| SRX4096863 | TFs and others | GATAD2A | Breast | HCC3153 | 1829 | 167/31605 | -0.20/0.26 | -5.3 | -5.1 | -4.55 | FALSE |
| SRX15232262 | TFs and others | DNA-RNA hybrid | Breast | MDA-MB-436 | 871 | 323/31605 | -0.07/0.26 | -5.2 | -5.1 | -4.54 | FALSE |
| SRX3265285 | TFs and others | KDM1A | Breast | MCF-7 | 810 | 93/31605 | -0.35/0.26 | -5.2 | -5.1 | -4.54 | FALSE |
| SRX4096866 | TFs and others | GATAD2B | Breast | HCC3153 | 578 | 138/31605 | -0.24/0.26 | -5.2 | -5.1 | -4.53 | FALSE |
| SRX490428 | TFs and others | BRD4 | Breast | MCF-7 | 235 | 45/31605 | -0.61/0.26 | -5.2 | -5.1 | -4.53 | FALSE |
| SRX10239459 | TFs and others | YTHDC1 | Breast | MCF-7 | 359 | 232/31605 | -0.13/0.26 | -5.2 | -5.1 | -4.52 | FALSE |
| SRX5089540 | TFs and others | ARID2 | Breast | MCF-7 | 1149 | 24/31605 | -0.92/0.26 | -5.2 | -5.0 | -4.51 | FALSE |
| SRX3321942 | TFs and others | CSDE1 | Breast | MCF-7 | 2284 | 379/31605 | -0.04/0.26 | -5.2 | -5.0 | -4.50 | FALSE |
| SRX747779 | TFs and others | ESR1 | Breast | MCF-7 | 557 | 13/31605 | -1.34/0.26 | -5.2 | -5.0 | -4.50 | FALSE |
| SRX15039981 | TFs and others | Epitope tags | Breast | MDA-MB-231 | 3724 | 441/31605 | -0.02/0.26 | -5.2 | -5.0 | -4.49 | FALSE |
| SRX10664192 | TFs and others | MTF2 | Breast | MCF-7 | 3784 | 336/31605 | -0.06/0.26 | -5.2 | -5.0 | -4.49 | FALSE |
| SRX4060991 | TFs and others | TRPS1 | Breast | T-47D | 1057 | 58/31605 | -0.50/0.26 | -5.1 | -5.0 | -4.49 | FALSE |
| SRX5057621 | TFs and others | GATA3 | Breast | T-47D | 663 | 8/31605 | -1.78/0.26 | -5.1 | -5.0 | -4.49 | FALSE |
| SRX2423468 | TFs and others | HSF1 | Breast | MCF-7 | 1988 | 195/31605 | -0.16/0.26 | -5.1 | -5.0 | -4.48 | FALSE |
| SRX7030883 | TFs and others | EHMT2 | Breast | MCF-7 | 2138 | 310/31605 | 0.58/0.26 | -5.1 | -5.0 | 4.48 | TRUE |
| SRX2871371 | TFs and others | Epitope tags | Breast | T-47D | 1644 | 97/31605 | -0.33/0.26 | -5.1 | -5.0 | -4.48 | FALSE |
| SRX2578869 | TFs and others | ESR1 | Breast | MDA-MB-231 | 543 | 42/31605 | -0.63/0.26 | -5.1 | -4.9 | -4.46 | FALSE |
| SRX4096890 | TFs and others | TRPS1 | Breast | SUM 159PT | 11753 | 577/31605 | 0.02/0.26 | -5.1 | -4.9 | -4.46 | FALSE |
| SRX5824587 | TFs and others | BRD4 | Breast | SUM 159PT | 8742 | 399/31605 | -0.03/0.26 | -5.1 | -4.9 | -4.46 | FALSE |
| SRX469393 | TFs and others | ESR1 | Breast | MCF-7 | 490 | 175/31605 | -0.18/0.26 | -5.1 | -4.9 | -4.46 | FALSE |
| SRX7624159 | TFs and others | NR2F2 | Breast | T-47D | 666 | 25/31605 | -0.88/0.26 | -5.1 | -4.9 | -4.45 | FALSE |
| SRX13840523 | TFs and others | 8-Hydroxydeoxyguanosine | Breast | MCF 10A | 119982 | 6814/31605 | 0.19/0.26 | -5.1 | -4.9 | -4.44 | FALSE |
| SRX747776 | TFs and others | ESR1 | Breast | MCF-7 | 860 | 16/31605 | -1.17/0.26 | -5.0 | -4.9 | -4.44 | FALSE |
| SRX647368 | TFs and others | ZBTB17 | Breast | MCF 10A | 908 | 271/31605 | -0.09/0.26 | -5.0 | -4.9 | -4.43 | FALSE |
| SRX17682346 | TFs and others | RELA | Breast | MCF-7 | 2424 | 408/31605 | -0.03/0.26 | -5.0 | -4.9 | -4.43 | FALSE |
| SRX5089501 | TFs and others | HDAC1 | Breast | MCF-7 | 940 | 89/31605 | -0.35/0.26 | -5.0 | -4.9 | -4.42 | FALSE |
| SRX2453453 | TFs and others | TOP2B | Breast | MCF 10A | 936 | 158/31605 | -0.20/0.26 | -5.0 | -4.9 | -4.42 | FALSE |
| SRX15670543 | TFs and others | TFE3 | Breast | MDA-MB-453 | 2081 | 215/31605 | -0.13/0.26 | -5.0 | -4.8 | -4.42 | FALSE |
| SRX19321950 | TFs and others | RNF2 | Breast | MCF-7 | 1818 | 868/31605 | 0.45/0.26 | -5.0 | -4.8 | 4.41 | TRUE |
| SRX6639038 | TFs and others | GTF3C2 | Breast | T-47D | 1579 | 135/31605 | -0.23/0.26 | -5.0 | -4.8 | -4.41 | FALSE |
| SRX10859402 | TFs and others | ARID2 | Breast | BT-549 | 1040 | 420/31605 | -0.02/0.26 | -5.0 | -4.8 | -4.41 | FALSE |
| SRX10664193 | TFs and others | CTR9 | Breast | MCF-7 | 4492 | 149/31605 | -0.21/0.26 | -5.0 | -4.8 | -4.40 | FALSE |
| SRX10664194 | TFs and others | CTR9 | Breast | MCF-7 | 3738 | 142/31605 | -0.22/0.26 | -5.0 | -4.8 | -4.40 | FALSE |
| SRX22997306 | TFs and others | MAFG | Breast | Hs 578T | 5351 | 586/31605 | 0.02/0.26 | -5.0 | -4.8 | -4.39 | FALSE |
| SRX3229185 | TFs and others | NR3C1 | Breast | Breast cancer | 915 | 13/31605 | -1.30/0.26 | -4.9 | -4.8 | -4.39 | FALSE |
| SRX15039982 | TFs and others | Epitope tags | Breast | MDA-MB-231 | 5938 | 726/31605 | 0.05/0.26 | -4.9 | -4.7 | -4.37 | FALSE |
| SRX10205616 | TFs and others | SMARCA4 | Breast | CAL-120 | 1853 | 270/31605 | -0.09/0.26 | -4.9 | -4.7 | -4.36 | FALSE |
| SRX1772417 | TFs and others | NEDD4 | Breast | MCF-7 | 1647 | 61/31605 | -0.46/0.26 | -4.9 | -4.7 | -4.36 | FALSE |
| SRX3546804 | TFs and others | Epitope tags | Breast | MDA-MB-231 | 131 | 14/31605 | -1.24/0.26 | -4.9 | -4.7 | -4.36 | FALSE |
| DRX447878 | TFs and others | HIF1A | Breast | MDA-MB-231 | 494 | 16/31605 | -1.14/0.26 | -4.9 | -4.7 | -4.35 | FALSE |
| SRX17682345 | TFs and others | RELA | Breast | MCF-7 | 2339 | 335/31605 | -0.05/0.26 | -4.9 | -4.7 | -4.35 | FALSE |
| SRX2423660 | TFs and others | ZBTB33 | Breast | MCF-7 | 4530 | 542/31605 | 0.02/0.26 | -4.9 | -4.7 | -4.35 | FALSE |
| SRX8520806 | TFs and others | NR3C1 | Breast | MDA-MB-231 | 15578 | 1383/31605 | 0.11/0.26 | -4.9 | -4.7 | -4.34 | FALSE |
| SRX2007817 | TFs and others | KMT2A | Breast | MCF-7 | 1745 | 29/31605 | -0.78/0.26 | -4.8 | -4.7 | -4.34 | FALSE |
| SRX7586660 | TFs and others | SOX4 | Breast | BT-549 | 230 | 77/31605 | -0.38/0.26 | -4.8 | -4.7 | -4.33 | FALSE |
| SRX5493752 | TFs and others | AR | Breast | Breast cancer cells | 354 | 5/31605 | -2.22/0.26 | -4.8 | -4.7 | -4.33 | FALSE |
| SRX5057619 | TFs and others | GATA3 | Breast | T-47D | 723 | 28/31605 | -0.79/0.26 | -4.8 | -4.7 | -4.33 | FALSE |
| SRX12008357 | TFs and others | CTCF | Breast | MCF 10A | 779 | 7/31605 | -1.84/0.26 | -4.8 | -4.7 | -4.33 | FALSE |
| SRX9534927 | TFs and others | FOS | Breast | Breast cancer cells | 1840 | 324/31605 | -0.05/0.26 | -4.8 | -4.7 | -4.32 | FALSE |
| SRX1709975 | TFs and others | PGR | Breast | T-47D | 1361 | 152/31605 | -0.19/0.26 | -4.8 | -4.7 | -4.32 | FALSE |
| SRX6487062 | TFs and others | FOXA1 | Breast | MCF-7 | 1111 | 54/31605 | -0.50/0.26 | -4.8 | -4.7 | -4.32 | FALSE |
| SRX5057626 | TFs and others | GATA3 | Breast | T-47D | 1148 | 49/31605 | -0.53/0.26 | -4.8 | -4.6 | -4.31 | FALSE |
| SRX1076936 | TFs and others | LMTK3 | Breast | MDA-MB-231 | 753 | 135/31605 | -0.22/0.26 | -4.8 | -4.6 | -4.29 | FALSE |
| SRX5172070 | TFs and others | ESR1 | Breast | MCF-7 | 666 | 12/31605 | -1.32/0.26 | -4.7 | -4.6 | -4.27 | FALSE |
| SRX6487053 | TFs and others | FOXA1 | Breast | MCF-7 | 3064 | 215/31605 | -0.12/0.26 | -4.7 | -4.5 | -4.25 | FALSE |
| SRX186798 | TFs and others | ESR1 | Breast | Breast cancer | 1457 | 23/31605 | -0.88/0.26 | -4.7 | -4.5 | -4.25 | FALSE |
| SRX1339020 | TFs and others | AR | Breast | MDA-MB-453 | 7190 | 395/31605 | -0.02/0.26 | -4.7 | -4.5 | -4.25 | FALSE |
| SRX1131559 | TFs and others | BRD4 | Breast | SUM 149PT | 3179 | 169/31605 | -0.16/0.26 | -4.7 | -4.5 | -4.25 | FALSE |
| SRX7366780 | TFs and others | CDK9 | Breast | MCF-7 | 611 | 132/31605 | -0.22/0.26 | -4.7 | -4.5 | -4.24 | FALSE |
| SRX5089518 | TFs and others | SMARCA4 | Breast | MCF-7 | 996 | 28/31605 | -0.77/0.26 | -4.7 | -4.5 | -4.24 | FALSE |
| SRX8083102 | TFs and others | FGFR1 | Breast | CAMA-1 | 1245 | 153/31605 | -0.18/0.26 | -4.6 | -4.5 | -4.24 | FALSE |
| SRX5824576 | TFs and others | BRD4 | Breast | SUM 149PT | 3169 | 169/31605 | -0.16/0.26 | -4.6 | -4.5 | -4.23 | FALSE |
| SRX9046307 | TFs and others | JUNB | Breast | MCF-7 | 2811 | 607/31605 | 0.04/0.26 | -4.6 | -4.5 | -4.22 | FALSE |
| SRX3862454 | TFs and others | FOXA1 | Breast | T-47D | 1371 | 81/31605 | -0.35/0.26 | -4.6 | -4.5 | -4.22 | FALSE |
| SRX490431 | TFs and others | BRD4 | Breast | MCF-7 | 369 | 22/31605 | -0.90/0.26 | -4.6 | -4.5 | -4.22 | FALSE |
| SRX278609 | TFs and others | NR5A2 | Breast | MCF-7 | 1358 | 88/31605 | -0.32/0.26 | -4.6 | -4.4 | -4.21 | FALSE |
| SRX5493759 | TFs and others | FOXA1 | Breast | Breast cancer cells | 1933 | 283/31605 | -0.06/0.26 | -4.5 | -4.4 | -4.19 | FALSE |
| SRX19888702 | TFs and others | SNAI2 | Breast | BT-549 | 3383 | 914/31605 | 0.08/0.26 | -4.5 | -4.4 | -4.19 | FALSE |
| SRX2424030 | TFs and others | GATAD2B | Breast | MCF-7 | 2217 | 100/31605 | -0.28/0.26 | -4.5 | -4.4 | -4.18 | FALSE |
| SRX2000342 | TFs and others | TET3 | Breast | MCF-12A | 1178 | 43/31605 | -0.56/0.26 | -4.5 | -4.4 | -4.17 | FALSE |
| SRX4192959 | TFs and others | SMC1A | Breast | MCF-7 | 13649 | 2121/31605 | 0.14/0.26 | -4.5 | -4.4 | -4.17 | FALSE |
| SRX8083105 | TFs and others | FGFR1 | Breast | CAMA-1 | 1054 | 71/31605 | -0.38/0.26 | -4.5 | -4.4 | -4.17 | FALSE |
| SRX1339019 | TFs and others | AR | Breast | MDA-MB-453 | 3348 | 187/31605 | -0.13/0.26 | -4.5 | -4.4 | -4.16 | FALSE |
| SRX1012707 | TFs and others | ESR1 | Breast | T-47D | 996 | 24/31605 | -0.83/0.26 | -4.5 | -4.4 | -4.16 | FALSE |
| SRX15929727 | TFs and others | STAG1 | Breast | MCF 10A | 13153 | 1452/31605 | 0.12/0.26 | -4.5 | -4.3 | -4.16 | FALSE |
| SRX7586661 | TFs and others | SOX4 | Breast | BT-549 | 214 | 38/31605 | -0.61/0.26 | -4.5 | -4.3 | -4.16 | FALSE |
| SRX14875681 | TFs and others | SET | Breast | MCF-7 | 1057 | 17/31605 | -1.04/0.26 | -4.5 | -4.3 | -4.15 | FALSE |
| SRX2424031 | TFs and others | GATAD2B | Breast | MCF-7 | 1710 | 69/31605 | -0.38/0.26 | -4.5 | -4.3 | -4.15 | FALSE |
| SRX10142199 | TFs and others | CTCF | Breast | Breast luminal cells | 1576 | 181/31605 | -0.14/0.26 | -4.5 | -4.3 | -4.15 | FALSE |
| SRX3546800 | TFs and others | Epitope tags | Breast | MDA-MB-231 | 128 | 23/31605 | -0.85/0.26 | -4.5 | -4.3 | -4.15 | FALSE |
| SRX3070470 | TFs and others | NR3C1 | Breast | MCF 10A | 578 | 68/31605 | -0.39/0.26 | -4.5 | -4.3 | -4.14 | FALSE |
| SRX3589921 | TFs and others | STAG1 | Breast | MCF 10A | 714 | 17/31605 | -1.03/0.26 | -4.4 | -4.3 | -4.13 | FALSE |
| SRX2897210 | TFs and others | ESR1 | Breast | Breast cancer cells | 689 | 235/31605 | -0.09/0.26 | -4.4 | -4.3 | -4.13 | FALSE |
| SRX19210550 | TFs and others | RELB | Breast | MDA-MB-231 | 819 | 131/31605 | -0.21/0.26 | -4.4 | -4.3 | -4.13 | FALSE |
| SRX10664196 | TFs and others | CTR9 | Breast | MCF-7 | 3460 | 108/31605 | -0.25/0.26 | -4.4 | -4.3 | -4.11 | FALSE |
| DRX447879 | TFs and others | HIF1A | Breast | MDA-MB-231 | 447 | 15/31605 | -1.10/0.26 | -4.4 | -4.3 | -4.11 | FALSE |
| SRX15929730 | TFs and others | STAG2 | Breast | MCF 10A | 2164 | 478/31605 | 0.01/0.26 | -4.4 | -4.3 | -4.11 | FALSE |
| SRX1065301 | TFs and others | Epitope tags | Breast | IMEC | 1340 | 100/31605 | -0.27/0.26 | -4.4 | -4.3 | -4.11 | FALSE |
| SRX3923526 | TFs and others | FOXA1 | Breast | ZR-75-1 | 9597 | 664/31605 | 0.05/0.26 | -4.4 | -4.2 | -4.10 | FALSE |
| SRX15574112 | TFs and others | MTA1 | Breast | MCF-7 | 648 | 24/31605 | -0.82/0.26 | -4.4 | -4.2 | -4.10 | FALSE |
| SRX5457178 | TFs and others | MAFK | Breast | MCF-7 | 4985 | 362/31605 | -0.02/0.26 | -4.4 | -4.2 | -4.10 | FALSE |
| SRX3595673 | TFs and others | BRD3 | Breast | MCF-7 | 611 | 60/31605 | -0.42/0.26 | -4.4 | -4.2 | -4.10 | FALSE |
| SRX4714459 | TFs and others | GTF3C2 | Breast | MCF 10A | 470 | 97/31605 | -0.28/0.26 | -4.4 | -4.2 | -4.10 | FALSE |
| SRX15929738 | TFs and others | STAG2 | Breast | MCF 10A | 3483 | 966/31605 | 0.09/0.26 | -4.4 | -4.2 | -4.10 | FALSE |
| SRX5824590 | TFs and others | BRD4 | Breast | SUM 159PT | 2239 | 53/31605 | -0.47/0.26 | -4.4 | -4.2 | -4.10 | FALSE |
| SRX1012697 | TFs and others | PGR | Breast | MCF-7 | 848 | 65/31605 | -0.39/0.26 | -4.4 | -4.2 | -4.09 | FALSE |
| SRX5493770 | TFs and others | ESR1 | Breast | Breast cancer cells | 415 | 6/31605 | -1.88/0.26 | -4.3 | -4.2 | -4.08 | FALSE |
| SRX4714480 | TFs and others | GTF3C2 | Breast | T-47D | 1617 | 94/31605 | -0.28/0.26 | -4.3 | -4.2 | -4.07 | FALSE |
| SRX5534873 | TFs and others | JUN | Breast | MCF-7 | 1238 | 528/31605 | 0.03/0.26 | -4.3 | -4.2 | -4.07 | FALSE |
| SRX8520804 | TFs and others | NR3C1 | Breast | HCC70 | 6778 | 595/31605 | 0.04/0.26 | -4.3 | -4.2 | -4.07 | FALSE |
| SRX17682348 | TFs and others | RELA | Breast | MCF-7 | 3048 | 457/31605 | 0.01/0.26 | -4.3 | -4.2 | -4.06 | FALSE |
| SRX13867213 | TFs and others | SOX9 | Breast | MCF-7 | 1524 | 103/31605 | -0.26/0.26 | -4.3 | -4.1 | -4.05 | FALSE |
| SRX3229178 | TFs and others | FOXA1 | Breast | Breast cancer | 1469 | 141/31605 | -0.18/0.26 | -4.3 | -4.1 | -4.04 | FALSE |
| SRX1131564 | TFs and others | BRD4 | Breast | SUM 149PT | 2946 | 91/31605 | -0.29/0.26 | -4.3 | -4.1 | -4.04 | FALSE |
| SRX6487065 | TFs and others | FOXA1 | Breast | MCF-7 | 4254 | 282/31605 | -0.05/0.26 | -4.3 | -4.1 | -4.04 | FALSE |
| SRX2423868 | TFs and others | SIX4 | Breast | MCF-7 | 1858 | 77/31605 | -0.33/0.26 | -4.3 | -4.1 | -4.03 | FALSE |
| SRX2897207 | TFs and others | ESR1 | Breast | Breast cancer cells | 1761 | 108/31605 | -0.24/0.26 | -4.2 | -4.1 | -4.03 | FALSE |
| SRX5401863 | TFs and others | PGR | Breast | T-47D | 1962 | 87/31605 | -0.30/0.26 | -4.2 | -4.1 | -4.02 | FALSE |
| SRX13327995 | TFs and others | CBX3 | Breast | MCF-7 | 1719 | 41/31605 | -0.55/0.26 | -4.2 | -4.1 | -4.02 | FALSE |
| SRX3322225 | TFs and others | ZBTB1 | Breast | MCF-7 | 1145 | 38/31605 | -0.58/0.26 | -4.2 | -4.1 | -4.02 | FALSE |
| SRX5635067 | TFs and others | RUNX1 | Breast | MCF 10A | 7924 | 681/31605 | 0.06/0.26 | -4.2 | -4.1 | -4.02 | FALSE |
| SRX5287701 | TFs and others | MED1 | Breast | MCF-7 | 653 | 106/31605 | -0.24/0.26 | -4.2 | -4.1 | -4.01 | FALSE |
| SRX15279659 | TFs and others | ZMYND8 | Breast | MDA-MB-231 | 615 | 208/31605 | -0.10/0.26 | -4.2 | -4.1 | -4.00 | FALSE |
| SRX9688334 | TFs and others | ESR1 | Breast | ZR-75-1 | 239 | 3/31605 | -2.68/0.26 | -4.2 | -4.0 | -3.98 | FALSE |
| SRX3321937 | TFs and others | HES1 | Breast | MCF-7 | 2352 | 124/31605 | -0.20/0.26 | -4.2 | -4.0 | -3.97 | FALSE |
| SRX5057627 | TFs and others | GATA3 | Breast | T-47D | 976 | 72/31605 | -0.34/0.26 | -4.1 | -4.0 | -3.97 | FALSE |
| SRX5323927 | TFs and others | STAT3 | Breast | Breast cancer cells | 1112 | 195/31605 | -0.11/0.26 | -4.1 | -4.0 | -3.96 | FALSE |
| SRX12193404 | TFs and others | ESR1 | Breast | MCF-7 | 439 | 39/31605 | -0.56/0.26 | -4.1 | -4.0 | -3.96 | FALSE |
| SRX2423940 | TFs and others | ZKSCAN1 | Breast | MCF-7 | 2702 | 224/31605 | -0.08/0.26 | -4.1 | -4.0 | -3.95 | FALSE |
| SRX15232269 | TFs and others | DNA-RNA hybrid | Breast | MDA-MB-436 | 565 | 104/31605 | -0.24/0.26 | -4.1 | -3.9 | -3.94 | FALSE |
| SRX1012690 | TFs and others | PGR | Breast | MCF-7 | 865 | 61/31605 | -0.39/0.26 | -4.1 | -3.9 | -3.94 | FALSE |
| SRX323401 | TFs and others | SPDEF | Breast | MCF-7 | 220 | 27/31605 | -0.72/0.26 | -4.1 | -3.9 | -3.94 | FALSE |
| SRX1799015 | TFs and others | STAT1 | Breast | MCF-7 | 230 | 117/31605 | -0.21/0.26 | -4.1 | -3.9 | -3.94 | FALSE |
| SRX15235729 | TFs and others | PRRX1 | Breast | MDA-MB-436 | 2007 | 254/31605 | -0.06/0.26 | -4.1 | -3.9 | -3.93 | FALSE |
| SRX19043740 | TFs and others | HOMEZ | Breast | MCF-7 | 2224 | 190/31605 | -0.11/0.26 | -4.1 | -3.9 | -3.93 | FALSE |
| SRX9046306 | TFs and others | JUNB | Breast | MCF-7 | 1506 | 372/31605 | -0.01/0.26 | -4.1 | -3.9 | -3.92 | FALSE |
| SRX10335185 | TFs and others | Epitope tags | Breast | MDA-MB-231 | 1892 | 178/31605 | -0.12/0.26 | -4.0 | -3.9 | -3.92 | FALSE |
| SRX8520803 | TFs and others | NR3C1 | Breast | HCC1937 | 13819 | 1246/31605 | 0.11/0.26 | -4.0 | -3.9 | -3.92 | FALSE |
| SRX5824567 | TFs and others | BRD4 | Breast | SUM 149PT | 2950 | 91/31605 | -0.27/0.26 | -4.0 | -3.9 | -3.92 | FALSE |
| SRX10775057 | TFs and others | RNF2 | Breast | T-47D | 1087 | 18/31605 | -0.93/0.26 | -4.0 | -3.9 | -3.91 | FALSE |
| SRX5185600 | TFs and others | CARM1 | Breast | MCF-7 | 526 | 118/31605 | -0.21/0.26 | -4.0 | -3.9 | -3.91 | FALSE |
| SRX039138 | TFs and others | FOXA1 | Breast | MCF-7 | 2695 | 223/31605 | -0.08/0.26 | -4.0 | -3.9 | -3.91 | FALSE |
| SRX4096867 | TFs and others | GATAD2B | Breast | HCC3153 | 1077 | 14/31605 | -1.08/0.26 | -4.0 | -3.9 | -3.90 | FALSE |
| SRX20830470 | TFs and others | PGR | Breast | T-47D | 300 | 4/31605 | -2.24/0.26 | -4.0 | -3.9 | -3.90 | FALSE |
| SRX5089542 | TFs and others | ARID2 | Breast | MCF-7 | 869 | 15/31605 | -1.03/0.26 | -4.0 | -3.9 | -3.90 | FALSE |
| SRX4649668 | TFs and others | TP63 | Breast | MCF 10A | 11158 | 1174/31605 | 0.11/0.26 | -4.0 | -3.9 | -3.89 | FALSE |
| SRX2424766 | TFs and others | ZNF592 | Breast | MCF-7 | 952 | 99/31605 | -0.24/0.26 | -4.0 | -3.8 | -3.88 | FALSE |
| SRX4671301 | TFs and others | EP300 | Breast | NAMEC8 | 5906 | 502/31605 | 0.03/0.26 | -3.9 | -3.8 | -3.86 | FALSE |
| SRX176873 | TFs and others | GATA3 | Breast | MCF-7 | 1194 | 64/31605 | -0.36/0.26 | -3.9 | -3.8 | -3.86 | FALSE |
| SRX3410378 | TFs and others | RNF2 | Breast | MDA-MB-231 | 2663 | 378/31605 | 0.00/0.26 | -3.9 | -3.8 | -3.85 | FALSE |
| SRX10142203 | TFs and others | CTCF | Breast | Breast cancer cell line | 1861 | 189/31605 | -0.10/0.26 | -3.9 | -3.8 | -3.83 | FALSE |
| SRX3322958 | TFs and others | ZNF512B | Breast | MCF-7 | 2291 | 200/31605 | -0.09/0.26 | -3.9 | -3.7 | -3.82 | FALSE |
| SRX2423469 | TFs and others | HSF1 | Breast | MCF-7 | 1295 | 69/31605 | -0.33/0.26 | -3.9 | -3.7 | -3.82 | FALSE |
| SRX13327980 | TFs and others | XRCC6 | Breast | MCF-7 | 548 | 12/31605 | -1.15/0.26 | -3.9 | -3.7 | -3.81 | FALSE |
| SRX2453452 | TFs and others | RAD51 | Breast | MCF 10A | 1184 | 9/31605 | -1.37/0.26 | -3.9 | -3.7 | -3.81 | FALSE |
| SRX2020849 | TFs and others | STAT3 | Breast | HCC1143 | 6117 | 674/31605 | 0.07/0.26 | -3.8 | -3.7 | -3.81 | FALSE |
| SRX7030882 | TFs and others | EHMT2 | Breast | MCF-7 | 2513 | 327/31605 | 0.52/0.26 | -3.8 | -3.7 | 3.80 | TRUE |
| SRX20152812 | TFs and others | Epitope tags | Breast | MDA-MB-231 | 4537 | 568/31605 | 0.05/0.26 | -3.8 | -3.7 | -3.79 | FALSE |
| SRX12008361 | TFs and others | CTCF | Breast | MCF 10A | 1786 | 9/31605 | -1.36/0.26 | -3.8 | -3.7 | -3.78 | FALSE |
| SRX9046259 | TFs and others | FOSL2 | Breast | MCF-7 | 1043 | 257/31605 | -0.05/0.26 | -3.8 | -3.7 | -3.78 | FALSE |
| SRX883583 | TFs and others | TEAD4 | Breast | MDA-MB-231 | 7626 | 642/31605 | 0.06/0.26 | -3.8 | -3.7 | -3.78 | FALSE |
| SRX7366779 | TFs and others | CDK9 | Breast | MCF-7 | 657 | 146/31605 | -0.14/0.26 | -3.8 | -3.7 | -3.77 | FALSE |
| SRX3554408 | TFs and others | CREB1 | Breast | MDA-MB-134-VI | 1222 | 102/31605 | -0.22/0.26 | -3.8 | -3.6 | -3.77 | FALSE |
| SRX490426 | TFs and others | BRD4 | Breast | MCF-7 | 434 | 27/31605 | -0.67/0.26 | -3.7 | -3.6 | -3.74 | FALSE |
| SRX3862457 | TFs and others | FOXA1 | Breast | T-47D | 1278 | 80/31605 | -0.28/0.26 | -3.7 | -3.6 | -3.74 | FALSE |
| SRX3070473 | TFs and others | NR3C1 | Breast | MCF 10A | 12664 | 1537/31605 | 0.13/0.26 | -3.7 | -3.6 | -3.74 | FALSE |
| SRX19636323 | TFs and others | 5-hmC | Breast | HMLE | 43783 | 4539/31605 | 0.18/0.26 | -3.7 | -3.6 | -3.74 | FALSE |
| SRX10306483 | TFs and others | KDM1A | Breast | T-47D | 796 | 6/31605 | -1.70/0.26 | -3.7 | -3.6 | -3.73 | FALSE |
| SRX14091668 | TFs and others | MYC | Breast | SUM 159PT | 606 | 6/31605 | -1.70/0.26 | -3.7 | -3.6 | -3.73 | FALSE |
| SRX10775029 | TFs and others | RNF2 | Breast | T-47D | 1438 | 12/31605 | -1.13/0.26 | -3.7 | -3.6 | -3.73 | FALSE |
| SRX7642862 | TFs and others | KMT2A | Breast | Mammary epithelial cells | 169 | 46/31605 | -0.45/0.26 | -3.7 | -3.6 | -3.73 | FALSE |
| SRX1528505 | TFs and others | SRPK1 | Breast | MCF 10A | 471 | 118/31605 | -0.18/0.26 | -3.7 | -3.6 | -3.73 | FALSE |
| SRX2642968 | TFs and others | NELFA | Breast | Breast cells | 243 | 56/31605 | -0.38/0.26 | -3.7 | -3.6 | -3.72 | FALSE |
| SRX24428389 | TFs and others | Epitope tags | Breast | T-47D | 1098 | 94/31605 | -0.24/0.26 | -3.7 | -3.6 | -3.72 | FALSE |
| SRX5401861 | TFs and others | NR3C1 | Breast | T-47D | 3084 | 208/31605 | -0.08/0.26 | -3.7 | -3.6 | -3.72 | FALSE |
| SRX13840522 | TFs and others | 8-Hydroxydeoxyguanosine | Breast | MCF 10A | 61692 | 4171/31605 | 0.18/0.26 | -3.7 | -3.6 | -3.72 | FALSE |
| SRX3229174 | TFs and others | AR | Breast | Breast cancer | 3603 | 163/31605 | -0.12/0.26 | -3.7 | -3.6 | -3.72 | FALSE |
| SRX3589918 | TFs and others | STAG1 | Breast | MCF 10A | 3315 | 478/31605 | 0.04/0.26 | -3.7 | -3.6 | -3.72 | FALSE |
| SRX3589928 | TFs and others | STAG2 | Breast | MCF 10A | 2401 | 802/31605 | 0.09/0.26 | -3.7 | -3.5 | -3.71 | FALSE |
| SRX19210546 | TFs and others | NFKB2 | Breast | MDA-MB-231 | 548 | 34/31605 | -0.56/0.26 | -3.7 | -3.5 | -3.70 | FALSE |
| SRX6831823 | TFs and others | TEAD4 | Breast | MCF 10A | 4267 | 344/31605 | -0.00/0.26 | -3.7 | -3.5 | -3.70 | FALSE |
| SRX1709989 | TFs and others | PGR | Breast | T-47D | 1157 | 118/31605 | -0.18/0.26 | -3.7 | -3.5 | -3.69 | FALSE |
| SRX3322410 | TFs and others | PPP1R10 | Breast | MCF-7 | 2120 | 70/31605 | -0.31/0.26 | -3.6 | -3.5 | -3.69 | FALSE |
| SRX9697819 | TFs and others | Epitope tags | Breast | MDA-MB-231 | 602 | 6/31605 | -1.66/0.26 | -3.6 | -3.5 | -3.67 | FALSE |
| SRX3734320 | TFs and others | TP53 | Breast | Mammary glands | 3828 | 349/31605 | 0.00/0.26 | -3.6 | -3.5 | -3.66 | FALSE |
| SRX5089519 | TFs and others | SMARCA4 | Breast | MCF-7 | 792 | 18/31605 | -0.85/0.26 | -3.6 | -3.5 | -3.66 | FALSE |
| SRX5359049 | TFs and others | SREBF2 | Breast | HCC70 | 21900 | 7551/31605 | 0.20/0.26 | -3.6 | -3.5 | -3.66 | FALSE |
| SRX8083104 | TFs and others | FGFR1 | Breast | CAMA-1 | 1038 | 83/31605 | -0.26/0.26 | -3.6 | -3.5 | -3.66 | FALSE |
| SRX107368 | TFs and others | ESR1 | Breast | H3396 | 7492 | 479/31605 | 0.04/0.26 | -3.6 | -3.5 | -3.66 | FALSE |
| SRX7030879 | TFs and others | CBX5 | Breast | MCF-7 | 1528 | 58/31605 | -0.36/0.26 | -3.6 | -3.5 | -3.66 | FALSE |
| SRX15232267 | TFs and others | DNA-RNA hybrid | Breast | MDA-MB-436 | 915 | 274/31605 | -0.03/0.26 | -3.6 | -3.5 | -3.65 | FALSE |
| SRX5323861 | TFs and others | STAT3 | Breast | MCF-7 | 435 | 65/31605 | -0.32/0.26 | -3.6 | -3.4 | -3.65 | FALSE |
| SRX11473188 | TFs and others | TBX2 | Breast | MCF-7 | 1391 | 99/31605 | -0.21/0.26 | -3.6 | -3.4 | -3.65 | FALSE |
| SRX2897223 | TFs and others | ESR1 | Breast | Breast cancer cells | 1189 | 6/31605 | -1.65/0.26 | -3.6 | -3.4 | -3.64 | FALSE |
| SRX2897225 | TFs and others | ESR1 | Breast | Breast cancer cells | 964 | 6/31605 | -1.65/0.26 | -3.6 | -3.4 | -3.64 | FALSE |
| SRX2897227 | TFs and others | ESR1 | Breast | Breast cancer cells | 944 | 6/31605 | -1.65/0.26 | -3.6 | -3.4 | -3.64 | FALSE |
| SRX4393798 | TFs and others | SIX2 | Breast | MCF-7 | 387 | 9/31605 | -1.30/0.26 | -3.6 | -3.4 | -3.64 | FALSE |
| SRX10775042 | TFs and others | ESR1 | Breast | T-47D | 955 | 12/31605 | -1.09/0.26 | -3.5 | -3.4 | -3.63 | FALSE |
| SRX730439 | TFs and others | HECTD1 | Breast | MCF-7 | 404 | 189/31605 | -0.08/0.26 | -3.5 | -3.4 | -3.63 | FALSE |
| SRX4649671 | TFs and others | TP63 | Breast | MCF 10A | 9806 | 1045/31605 | 0.11/0.26 | -3.5 | -3.4 | -3.62 | FALSE |
| SRX16341658 | TFs and others | KDM1A | Breast | T-47D | 1864 | 198/31605 | -0.07/0.26 | -3.5 | -3.4 | -3.60 | FALSE |
| SRX155749 | TFs and others | HSF1 | Breast | MCF-7 | 526 | 92/31605 | -0.22/0.26 | -3.5 | -3.4 | -3.60 | FALSE |
| SRX155755 | TFs and others | HSF1 | Breast | SK-BR-3 | 885 | 227/31605 | -0.05/0.26 | -3.5 | -3.4 | -3.60 | FALSE |
| SRX730435 | TFs and others | NCAPG2 | Breast | MCF-7 | 835 | 19/31605 | -0.80/0.26 | -3.5 | -3.4 | -3.59 | FALSE |
| SRX2460051 | TFs and others | PGR | Breast | Breast cancer cells | 238 | 18/31605 | -0.83/0.26 | -3.5 | -3.4 | -3.59 | FALSE |
| SRX1100313 | TFs and others | RELA | Breast | HMEC | 3686 | 811/31605 | 0.09/0.26 | -3.5 | -3.4 | -3.59 | FALSE |
| SRX13327974 | TFs and others | CBX3 | Breast | MCF-7 | 819 | 35/31605 | -0.52/0.26 | -3.5 | -3.3 | -3.59 | FALSE |
| SRX4714456 | TFs and others | BDP1 | Breast | T-47D | 1999 | 70/31605 | -0.29/0.26 | -3.5 | -3.3 | -3.58 | FALSE |
| SRX3923185 | TFs and others | DDX5 | Breast | BT-549 | 1534 | 86/31605 | -0.24/0.26 | -3.5 | -3.3 | -3.58 | FALSE |
| SRX7644281 | TFs and others | CPSF3L | Breast | MCF-7 | 307 | 68/31605 | -0.30/0.26 | -3.5 | -3.3 | -3.58 | FALSE |
| SRX6487057 | TFs and others | FOXA1 | Breast | MCF-7 | 1348 | 92/31605 | -0.22/0.26 | -3.5 | -3.3 | -3.57 | FALSE |
| SRX2897235 | TFs and others | ESR1 | Breast | Breast cancer cells | 1386 | 8/31605 | -1.36/0.26 | -3.4 | -3.3 | -3.57 | FALSE |
| SRX21436550 | TFs and others | TOP1 | Breast | MCF-7 | 2536 | 18/31605 | -0.82/0.26 | -3.4 | -3.3 | -3.57 | FALSE |
| SRX9697817 | TFs and others | Epitope tags | Breast | MDA-MB-231 | 620 | 58/31605 | -0.34/0.26 | -3.4 | -3.3 | -3.57 | FALSE |
| SRX15039986 | TFs and others | CHD4 | Breast | T-47D | 897 | 197/31605 | -0.07/0.26 | -3.4 | -3.3 | -3.57 | FALSE |
| SRX12788793 | TFs and others | JMJD6 | Breast | MCF-7 | 324 | 30/31605 | -0.58/0.26 | -3.4 | -3.3 | -3.56 | FALSE |
| SRX2424606 | TFs and others | NONO | Breast | MCF-7 | 1863 | 147/31605 | -0.12/0.26 | -3.4 | -3.3 | -3.56 | FALSE |
| SRX6058770 | TFs and others | TOP2A | Breast | T-47D | 3372 | 31/31605 | -0.56/0.26 | -3.4 | -3.3 | -3.55 | FALSE |
| SRX13327977 | TFs and others | CBX3 | Breast | MCF-7 | 994 | 15/31605 | -0.92/0.26 | -3.4 | -3.3 | -3.55 | FALSE |
| SRX2897233 | TFs and others | ESR1 | Breast | Breast cancer cells | 1648 | 10/31605 | -1.18/0.26 | -3.4 | -3.3 | -3.54 | FALSE |
| SRX10040036 | TFs and others | Epitope tags | Breast | MDA-MB-231 | 1774 | 78/31605 | -0.26/0.26 | -3.4 | -3.3 | -3.54 | FALSE |
| SRX13327999 | TFs and others | Epitope tags | Breast | MCF-7 | 761 | 41/31605 | -0.45/0.26 | -3.4 | -3.3 | -3.53 | FALSE |
| SRX11650839 | TFs and others | SMARCA4 | Breast | CAL-120 | 693 | 6/31605 | -1.59/0.26 | -3.4 | -3.3 | -3.53 | FALSE |
| SRX3541113 | TFs and others | SUMO2 | Breast | MCF-7 | 1643 | 80/31605 | -0.25/0.26 | -3.4 | -3.3 | -3.53 | FALSE |
| SRX6487066 | TFs and others | FOXA1 | Breast | MCF-7 | 2151 | 142/31605 | -0.12/0.26 | -3.4 | -3.2 | -3.53 | FALSE |
| SRX4017881 | TFs and others | TP63 | Breast | Breast organoids | 1149 | 39/31605 | -0.47/0.26 | -3.4 | -3.2 | -3.53 | FALSE |
| SRX13300076 | TFs and others | CTCF | Breast | MCF 10A | 796 | 7/31605 | -1.45/0.26 | -3.4 | -3.2 | -3.52 | FALSE |
| SRX9697815 | TFs and others | Epitope tags | Breast | MDA-MB-231 | 722 | 7/31605 | -1.45/0.26 | -3.4 | -3.2 | -3.52 | FALSE |
| SRX6027752 | TFs and others | NR2F2 | Breast | MCF-7 | 18242 | 3194/31605 | 0.33/0.26 | -3.4 | -3.2 | 3.52 | TRUE |
| SRX495668 | TFs and others | NR3C1 | Breast | MDA-MB-231 | 2970 | 115/31605 | -0.17/0.26 | -3.4 | -3.2 | -3.52 | FALSE |
| SRX3110736 | TFs and others | ESR1 | Breast | MCF-7 | 1284 | 97/31605 | -0.20/0.26 | -3.4 | -3.2 | -3.52 | FALSE |
| SRX19210544 | TFs and others | NFKB2 | Breast | MDA-MB-231 | 820 | 121/31605 | -0.15/0.26 | -3.4 | -3.2 | -3.52 | FALSE |
| SRX10306474 | TFs and others | JUN | Breast | T-47D | 943 | 5/31605 | -1.76/0.26 | -3.4 | -3.2 | -3.52 | FALSE |
| SRX10306484 | TFs and others | JUN | Breast | T-47D | 842 | 5/31605 | -1.76/0.26 | -3.4 | -3.2 | -3.52 | FALSE |
| SRX11650840 | TFs and others | SMARCA4 | Breast | CAL-120 | 641 | 5/31605 | -1.76/0.26 | -3.4 | -3.2 | -3.52 | FALSE |
| SRX14612962 | TFs and others | JUN | Breast | T-47D | 1182 | 5/31605 | -1.76/0.26 | -3.4 | -3.2 | -3.52 | FALSE |
| SRX14612963 | TFs and others | JUN | Breast | T-47D | 1018 | 5/31605 | -1.76/0.26 | -3.4 | -3.2 | -3.52 | FALSE |
| SRX14612964 | TFs and others | JUN | Breast | T-47D | 960 | 5/31605 | -1.76/0.26 | -3.4 | -3.2 | -3.52 | FALSE |
| SRX14612977 | TFs and others | SMARCC1 | Breast | T-47D | 931 | 5/31605 | -1.76/0.26 | -3.4 | -3.2 | -3.52 | FALSE |
| SRX14612978 | TFs and others | SMARCC1 | Breast | T-47D | 967 | 5/31605 | -1.76/0.26 | -3.4 | -3.2 | -3.52 | FALSE |
| SRX14612980 | TFs and others | JUN | Breast | T-47D | 894 | 5/31605 | -1.76/0.26 | -3.4 | -3.2 | -3.52 | FALSE |
| SRX14612984 | TFs and others | JUN | Breast | T-47D | 949 | 5/31605 | -1.76/0.26 | -3.4 | -3.2 | -3.52 | FALSE |
| SRX14612985 | TFs and others | JUN | Breast | T-47D | 1025 | 5/31605 | -1.76/0.26 | -3.4 | -3.2 | -3.52 | FALSE |
| SRX2000339 | TFs and others | TET2 | Breast | MCF-12A | 809 | 5/31605 | -1.76/0.26 | -3.4 | -3.2 | -3.52 | FALSE |
| SRX2000340 | TFs and others | RARB | Breast | MCF-12A | 576 | 5/31605 | -1.76/0.26 | -3.4 | -3.2 | -3.52 | FALSE |
| SRX2000341 | TFs and others | 5-hmC | Breast | MCF-12A | 700 | 5/31605 | -1.76/0.26 | -3.4 | -3.2 | -3.52 | FALSE |
| SRX2000343 | TFs and others | RARB | Breast | MCF-12A | 721 | 5/31605 | -1.76/0.26 | -3.4 | -3.2 | -3.52 | FALSE |
| SRX2000344 | TFs and others | 5-hmC | Breast | MCF-12A | 509 | 5/31605 | -1.76/0.26 | -3.4 | -3.2 | -3.52 | FALSE |
| SRX2897222 | TFs and others | ESR1 | Breast | Breast cancer cells | 1182 | 5/31605 | -1.76/0.26 | -3.4 | -3.2 | -3.52 | FALSE |
| SRX8353668 | TFs and others | LMNA | Breast | MDA-MB-231 | 540 | 5/31605 | -1.76/0.26 | -3.4 | -3.2 | -3.52 | FALSE |
| SRX8353670 | TFs and others | LMNA | Breast | MDA-MB-231 | 574 | 5/31605 | -1.76/0.26 | -3.4 | -3.2 | -3.52 | FALSE |
| SRX8353671 | TFs and others | LMNA | Breast | MDA-MB-231 | 580 | 5/31605 | -1.76/0.26 | -3.4 | -3.2 | -3.52 | FALSE |
| SRX5089541 | TFs and others | ARID2 | Breast | MCF-7 | 836 | 11/31605 | -1.10/0.26 | -3.4 | -3.2 | -3.52 | FALSE |
| SRX2425861 | TFs and others | SMAD1 | Breast | MDA-MB-231 | 1659 | 52/31605 | -0.37/0.26 | -3.4 | -3.2 | -3.52 | FALSE |
| SRX20830489 | TFs and others | YAP1 | Breast | T-47D | 284 | 5/31605 | -1.76/0.26 | -3.4 | -3.2 | -3.52 | FALSE |
| SRX7130722 | TFs and others | TP53 | Breast | MCF 10A | 3146 | 288/31605 | -0.01/0.26 | -3.4 | -3.2 | -3.51 | FALSE |
| SRX13254868 | TFs and others | SUZ12 | Breast | MCF-7 | 24878 | 2565/31605 | 0.17/0.26 | -3.4 | -3.2 | -3.51 | FALSE |
| SRX5824571 | TFs and others | BRD4 | Breast | SUM 149PT | 2356 | 108/31605 | -0.18/0.26 | -3.3 | -3.2 | -3.51 | FALSE |
| SRX2578921 | TFs and others | EP300 | Breast | MDA-MB-231 | 867 | 32/31605 | -0.54/0.26 | -3.3 | -3.2 | -3.49 | FALSE |
| SRX2578914 | TFs and others | EP300 | Breast | MDA-MB-231 | 1066 | 19/31605 | -0.77/0.26 | -3.3 | -3.2 | -3.49 | FALSE |
| SRX7262856 | TFs and others | AR | Breast | Breast cancer cells | 2178 | 19/31605 | -0.77/0.26 | -3.3 | -3.2 | -3.49 | FALSE |
| SRX2897234 | TFs and others | ESR1 | Breast | Breast cancer cells | 1387 | 8/31605 | -1.33/0.26 | -3.3 | -3.2 | -3.49 | FALSE |
| SRX7644276 | TFs and others | METTL3 | Breast | MCF-7 | 556 | 26/31605 | -0.62/0.26 | -3.3 | -3.2 | -3.49 | FALSE |
| SRX4451203 | TFs and others | ESR1 | Breast | MCF-7 | 644 | 7/31605 | -1.43/0.26 | -3.3 | -3.2 | -3.48 | FALSE |
| SRX1012694 | TFs and others | PGR | Breast | MCF-7 | 1473 | 47/31605 | -0.40/0.26 | -3.3 | -3.2 | -3.48 | FALSE |
| SRX100262 | TFs and others | CREBBP | Breast | H3396 | 8652 | 495/31605 | 0.46/0.26 | -3.3 | -3.2 | 3.48 | TRUE |
| SRX10119386 | TFs and others | JUNB | Breast | MCF 10A | 47819 | 3515/31605 | 0.18/0.26 | -3.3 | -3.2 | -3.47 | FALSE |
| SRX11374768 | TFs and others | PGR | Breast | T-47D | 2197 | 29/31605 | -0.57/0.26 | -3.3 | -3.2 | -3.47 | FALSE |
| SRX4714460 | TFs and others | BDP1 | Breast | MCF 10A | 1054 | 52/31605 | -0.36/0.26 | -3.3 | -3.1 | -3.46 | FALSE |
| SRX2578916 | TFs and others | EP300 | Breast | MDA-MB-231 | 1186 | 48/31605 | -0.38/0.26 | -3.3 | -3.1 | -3.46 | FALSE |
| SRX5057616 | TFs and others | Epitope tags | Breast | T-47D | 714 | 27/31605 | -0.60/0.26 | -3.3 | -3.1 | -3.46 | FALSE |
| SRX5493748 | TFs and others | AR | Breast | Breast cancer cells | 1153 | 57/31605 | -0.33/0.26 | -3.3 | -3.1 | -3.46 | FALSE |
| SRX003937 | TFs and others | ESR1 | Breast | MCF-7 | 744 | 39/31605 | -0.45/0.26 | -3.3 | -3.1 | -3.45 | FALSE |
| SRX10399320 | TFs and others | KMT2A | Breast | Breast epithelium | 784 | 20/31605 | -0.73/0.26 | -3.3 | -3.1 | -3.45 | FALSE |
| SRX13327996 | TFs and others | CBX3 | Breast | MCF-7 | 941 | 67/31605 | -0.28/0.26 | -3.3 | -3.1 | -3.45 | FALSE |
| SRX4909431 | TFs and others | HDAC2 | Breast | HCC3153 | 928 | 183/31605 | -0.07/0.26 | -3.2 | -3.1 | -3.45 | FALSE |
| SRX7879084 | TFs and others | Epitope tags | Breast | MDA-MB-231 | 1200 | 16/31605 | -0.85/0.26 | -3.2 | -3.1 | -3.44 | FALSE |
| SRX1212811 | TFs and others | MED12 | Breast | MCF-7 | 946 | 14/31605 | -0.92/0.26 | -3.2 | -3.1 | -3.44 | FALSE |
| SRX6639037 | TFs and others | GTF3C2 | Breast | T-47D | 1615 | 73/31605 | -0.26/0.26 | -3.2 | -3.1 | -3.43 | FALSE |
| SRX18191658 | TFs and others | ESR1 | Breast | MCF-7 | 665 | 59/31605 | -0.32/0.26 | -3.2 | -3.1 | -3.43 | FALSE |
| SRX13941916 | TFs and others | ARID1A | Breast | MCF-7 | 2248 | 137/31605 | -0.12/0.26 | -3.2 | -3.1 | -3.43 | FALSE |
| SRX21436549 | TFs and others | TOP1 | Breast | MCF-7 | 1415 | 11/31605 | -1.07/0.26 | -3.2 | -3.1 | -3.43 | FALSE |
| SRX2585779 | TFs and others | FOSL1 | Breast | MDA-MB-231 | 24993 | 1991/31605 | 0.16/0.26 | -3.2 | -3.1 | -3.42 | FALSE |
| SRX3589926 | TFs and others | STAG1 | Breast | MCF 10A | 7204 | 1010/31605 | 0.12/0.26 | -3.2 | -3.1 | -3.42 | FALSE |
| SRX4815186 | TFs and others | EN1 | Breast | SUM 159PT | 1087 | 7/31605 | -1.40/0.26 | -3.2 | -3.1 | -3.42 | FALSE |
| SRX10142196 | TFs and others | CTCF | Breast | Mammary epithelial cells | 974 | 50/31605 | -0.36/0.26 | -3.2 | -3.1 | -3.42 | FALSE |
| SRX4714452 | TFs and others | BDP1 | Breast | T-47D | 1220 | 91/31605 | -0.20/0.26 | -3.2 | -3.1 | -3.41 | FALSE |
| SRX2897224 | TFs and others | ESR1 | Breast | Breast cancer cells | 273 | 4/31605 | -1.93/0.26 | -3.2 | -3.1 | -3.41 | FALSE |
| SRX6098149 | TFs and others | GATA3 | Breast | MCF-7 | 3254 | 165/31605 | -0.09/0.26 | -3.2 | -3.1 | -3.41 | FALSE |
| SRX4192886 | TFs and others | NFKB1 | Breast | MCF 10A | 17179 | 1732/31605 | 0.15/0.26 | -3.2 | -3.1 | -3.41 | FALSE |
| SRX8023128 | TFs and others | TP53 | Breast | MCF 10A | 2168 | 153/31605 | -0.10/0.26 | -3.2 | -3.1 | -3.41 | FALSE |
| SRX15235728 | TFs and others | PRRX1 | Breast | MDA-MB-436 | 899 | 126/31605 | -0.13/0.26 | -3.2 | -3.1 | -3.41 | FALSE |
| SRX155731 | TFs and others | HSF1 | Breast | MCF 10A | 1808 | 351/31605 | 0.02/0.26 | -3.2 | -3.1 | -3.41 | FALSE |
| SRX10775015 | TFs and others | ESR1 | Breast | T-47D | 1190 | 13/31605 | -0.95/0.26 | -3.2 | -3.1 | -3.41 | FALSE |
| SRX15039990 | TFs and others | CHD4 | Breast | MDA-MB-231 | 1755 | 172/31605 | -0.08/0.26 | -3.2 | -3.1 | -3.40 | FALSE |
| SRX10664187 | TFs and others | JARID2 | Breast | MCF-7 | 1975 | 145/31605 | -0.11/0.26 | -3.2 | -3.0 | -3.40 | FALSE |
| SRX262133 | TFs and others | HSF1 | Breast | MCF-7 | 425 | 116/31605 | -0.15/0.26 | -3.1 | -3.0 | -3.38 | FALSE |
| SRX3993992 | TFs and others | TRIM24 | Breast | MCF-7 | 381 | 14/31605 | -0.90/0.26 | -3.1 | -3.0 | -3.38 | FALSE |
| SRX10306478 | TFs and others | JUN | Breast | T-47D | 941 | 12/31605 | -0.99/0.26 | -3.1 | -3.0 | -3.38 | FALSE |
| SRX107367 | TFs and others | ESR1 | Breast | H3396 | 2026 | 112/31605 | -0.15/0.26 | -3.1 | -3.0 | -3.37 | FALSE |
| SRX10239461 | TFs and others | YTHDC1 | Breast | MCF-7 | 121 | 2/31605 | -2.80/0.26 | -3.1 | -3.0 | -3.37 | FALSE |
| SRX386755 | TFs and others | SMARCC1 | Breast | MCF-7 | 142 | 2/31605 | -2.80/0.26 | -3.1 | -3.0 | -3.37 | FALSE |
| SRX730431 | TFs and others | NCAPG | Breast | MCF-7 | 103 | 2/31605 | -2.80/0.26 | -3.1 | -3.0 | -3.37 | FALSE |
| SRX9534896 | TFs and others | NR4A1 | Breast | MCF 10A | 221 | 2/31605 | -2.80/0.26 | -3.1 | -3.0 | -3.37 | FALSE |
| SRX7121996 | TFs and others | SMARCC1 | Breast | MCF-7 | 266 | 9/31605 | -1.18/0.26 | -3.1 | -3.0 | -3.37 | FALSE |
| SRX7624160 | TFs and others | NR2F2 | Breast | T-47D | 1029 | 11/31605 | -1.04/0.26 | -3.1 | -3.0 | -3.36 | FALSE |
| SRX8537745 | TFs and others | GATA3 | Breast | MCF-7 | 910 | 7/31605 | -1.37/0.26 | -3.1 | -3.0 | -3.36 | FALSE |
| SRX518254 | TFs and others | 5-mC | Breast | MCF-7 | 37622 | 3047/31605 | 0.18/0.26 | -3.1 | -3.0 | -3.36 | FALSE |
| SRX15929729 | TFs and others | STAG1 | Breast | MCF 10A | 16068 | 1794/31605 | 0.15/0.26 | -3.1 | -3.0 | -3.36 | FALSE |
| SRX1012650 | TFs and others | PGR | Breast | MCF-7 | 544 | 50/31605 | -0.35/0.26 | -3.1 | -3.0 | -3.35 | FALSE |
| SRX5057608 | TFs and others | Epitope tags | Breast | T-47D | 662 | 16/31605 | -0.82/0.26 | -3.1 | -3.0 | -3.35 | FALSE |
| SRX2423477 | TFs and others | ZNF207 | Breast | MCF-7 | 1496 | 109/31605 | -0.16/0.26 | -3.1 | -3.0 | -3.35 | FALSE |
| SRX14091666 | TFs and others | MYC | Breast | SUM 159PT | 1105 | 11/31605 | -1.04/0.26 | -3.1 | -3.0 | -3.35 | FALSE |
| SRX7101365 | TFs and others | TEAD1 | Breast | Breast cancer cells | 920 | 7/31605 | -1.36/0.26 | -3.1 | -3.0 | -3.34 | FALSE |
| SRX2900096 | TFs and others | BAP1 | Breast | CAL-51 | 1033 | 20/31605 | -0.70/0.26 | -3.1 | -3.0 | -3.34 | FALSE |
| SRX13327975 | TFs and others | CBX3 | Breast | MCF-7 | 1730 | 30/31605 | -0.53/0.26 | -3.1 | -3.0 | -3.34 | FALSE |
| SRX20830472 | TFs and others | PGR | Breast | T-47D | 377 | 5/31605 | -1.66/0.26 | -3.1 | -3.0 | -3.34 | FALSE |
| SRX5057618 | TFs and others | Epitope tags | Breast | T-47D | 523 | 15/31605 | -0.85/0.26 | -3.1 | -3.0 | -3.34 | FALSE |
| SRX4096868 | TFs and others | GATAD2B | Breast | HCC3153 | 1167 | 14/31605 | -0.89/0.26 | -3.1 | -3.0 | -3.34 | FALSE |
| SRX8520801 | TFs and others | NR3C1 | Breast | BT-474 | 510 | 123/31605 | -0.13/0.26 | -3.1 | -3.0 | -3.34 | FALSE |
| SRX2912061 | TFs and others | ESR1 | Breast | MCF-7 | 205 | 26/31605 | -0.58/0.26 | -3.1 | -2.9 | -3.33 | FALSE |
| SRX10205614 | TFs and others | SMARCA4 | Breast | CAL-120 | 663 | 6/31605 | -1.49/0.26 | -3.1 | -2.9 | -3.33 | FALSE |
| SRX11650841 | TFs and others | SMARCA4 | Breast | CAL-120 | 699 | 6/31605 | -1.49/0.26 | -3.1 | -2.9 | -3.33 | FALSE |
| SRX2588918 | TFs and others | KMT2D | Breast | T-47D | 2144 | 27/31605 | -0.57/0.26 | -3.1 | -2.9 | -3.33 | FALSE |
| SRX1709987 | TFs and others | PGR | Breast | T-47D | 1610 | 174/31605 | -0.07/0.26 | -3.1 | -2.9 | -3.33 | FALSE |
| SRX6058769 | TFs and others | TOP2A | Breast | T-47D | 1706 | 10/31605 | -1.09/0.26 | -3.1 | -2.9 | -3.33 | FALSE |
| SRX5503017 | TFs and others | E4F1 | Breast | SUM 159PT | 2153 | 73/31605 | -0.24/0.26 | -3.1 | -2.9 | -3.32 | FALSE |
| SRX2378692 | TFs and others | AHRR | Breast | MCF-7 | 1013 | 69/31605 | -0.26/0.26 | -3.1 | -2.9 | -3.32 | FALSE |
| SRX1012696 | TFs and others | PGR | Breast | MCF-7 | 861 | 58/31605 | -0.30/0.26 | -3.1 | -2.9 | -3.32 | FALSE |
| SRX21436551 | TFs and others | TOP1 | Breast | MCF-7 | 1731 | 16/31605 | -0.81/0.26 | -3.0 | -2.9 | -3.32 | FALSE |
| SRX3229182 | TFs and others | NR3C1 | Breast | Breast cancer | 815 | 36/31605 | -0.45/0.26 | -3.0 | -2.9 | -3.32 | FALSE |
| SRX647366 | TFs and others | ESR1 | Breast | MCF 10A | 543 | 67/31605 | -0.26/0.26 | -3.0 | -2.9 | -3.32 | FALSE |
| SRX5057613 | TFs and others | GATA3 | Breast | T-47D | 546 | 13/31605 | -0.92/0.26 | -3.0 | -2.9 | -3.31 | FALSE |
| SRX1772415 | TFs and others | NEDD4 | Breast | MCF-7 | 1103 | 64/31605 | -0.27/0.26 | -3.0 | -2.9 | -3.31 | FALSE |
| SRX3862460 | TFs and others | FOXA1 | Breast | T-47D | 885 | 60/31605 | -0.29/0.26 | -3.0 | -2.9 | -3.31 | FALSE |
| SRX3229125 | TFs and others | FOXA1 | Breast | Breast cancer | 571 | 15/31605 | -0.84/0.26 | -3.0 | -2.9 | -3.30 | FALSE |
| DRX013211 | TFs and others | RAD21 | Breast | MCF-7 | 136 | 4/31605 | -1.86/0.26 | -3.0 | -2.9 | -3.30 | FALSE |
| SRX5089544 | TFs and others | ARID2 | Breast | MCF-7 | 1062 | 16/31605 | -0.80/0.26 | -3.0 | -2.9 | -3.30 | FALSE |
| SRX20152813 | TFs and others | Epitope tags | Breast | MDA-MB-231 | 4814 | 604/31605 | 0.08/0.26 | -3.0 | -2.9 | -3.30 | FALSE |
| SRX1117696 | TFs and others | ZMYND8 | Breast | ZR-75-30 | 250 | 52/31605 | -0.33/0.26 | -3.0 | -2.9 | -3.29 | FALSE |
| SRX17024137 | TFs and others | PRMT6 | Breast | MDA-MB-231 | 89 | 67/31605 | -0.26/0.26 | -3.0 | -2.9 | -3.28 | FALSE |
| SRX22997296 | TFs and others | MAFG | Breast | MCF-7 | 1495 | 141/31605 | -0.10/0.26 | -3.0 | -2.9 | -3.28 | FALSE |
| SRX1131558 | TFs and others | BRD4 | Breast | SUM 149PT | 2389 | 108/31605 | -0.15/0.26 | -3.0 | -2.9 | -3.28 | FALSE |
| SRX9688338 | TFs and others | SP1 | Breast | ZR-75-1 | 1235 | 9/31605 | -1.14/0.26 | -3.0 | -2.9 | -3.28 | FALSE |
| SRX730429 | TFs and others | NCAPG | Breast | MCF-7 | 653 | 10/31605 | -1.07/0.26 | -3.0 | -2.9 | -3.28 | FALSE |
| SRX13327997 | TFs and others | Epitope tags | Breast | MCF-7 | 602 | 8/31605 | -1.23/0.26 | -3.0 | -2.9 | -3.27 | FALSE |
| SRX5057628 | TFs and others | GATA3 | Breast | T-47D | 883 | 32/31605 | -0.49/0.26 | -3.0 | -2.9 | -3.27 | FALSE |
| SRX10664191 | TFs and others | MTF2 | Breast | MCF-7 | 1533 | 84/31605 | -0.20/0.26 | -3.0 | -2.9 | -3.27 | FALSE |
| SRX481566 | TFs and others | BRCA1 | Breast | MCF 10A | 1586 | 26/31605 | -0.57/0.26 | -3.0 | -2.8 | -3.27 | FALSE |
| SRX3070474 | TFs and others | NR3C1 | Breast | MCF 10A | 1259 | 152/31605 | -0.08/0.26 | -3.0 | -2.8 | -3.27 | FALSE |
| SRX1012710 | TFs and others | ESR1 | Breast | T-47D | 1125 | 16/31605 | -0.79/0.26 | -3.0 | -2.8 | -3.26 | FALSE |
| SRX7642861 | TFs and others | KMT2A | Breast | Mammary epithelial cells | 179 | 49/31605 | -0.34/0.26 | -3.0 | -2.8 | -3.26 | FALSE |
| SRX734307 | TFs and others | FOXM1 | Breast | HMEC | 574 | 27/31605 | -0.55/0.26 | -3.0 | -2.8 | -3.26 | FALSE |
| SRX24428387 | TFs and others | Epitope tags | Breast | T-47D | 1084 | 121/31605 | -0.12/0.26 | -3.0 | -2.8 | -3.26 | FALSE |
| SRX5172067 | TFs and others | ESR1 | Breast | MCF-7 | 579 | 10/31605 | -1.06/0.26 | -2.9 | -2.8 | -3.25 | FALSE |
| SRX9739056 | TFs and others | NKRF | Breast | MDA-MB-231 | 707 | 9/31605 | -1.13/0.26 | -2.9 | -2.8 | -3.25 | FALSE |
| SRX11473186 | TFs and others | TBX2 | Breast | MCF-7 | 1222 | 55/31605 | -0.31/0.26 | -2.9 | -2.8 | -3.24 | FALSE |
| SRX6058759 | TFs and others | PGR | Breast | T-47D | 2161 | 8/31605 | -1.21/0.26 | -2.9 | -2.8 | -3.24 | FALSE |
| SRX4671297 | TFs and others | RARA | Breast | NAMEC8 | 2153 | 214/31605 | -0.03/0.26 | -2.9 | -2.8 | -3.24 | FALSE |
| SRX5323821 | TFs and others | STAT3 | Breast | T-47D | 795 | 38/31605 | -0.42/0.26 | -2.9 | -2.8 | -3.23 | FALSE |
| SRX11120869 | TFs and others | CREBBP | Breast | MCF-7 | 1208 | 13/31605 | -0.89/0.26 | -2.9 | -2.8 | -3.23 | FALSE |
| SRX12193402 | TFs and others | ESR1 | Breast | MCF-7 | 390 | 19/31605 | -0.69/0.26 | -2.9 | -2.8 | -3.22 | FALSE |
| SRX4017885 | TFs and others | TP63 | Breast | Breast organoids | 1786 | 9/31605 | -1.12/0.26 | -2.9 | -2.8 | -3.21 | FALSE |
| SRX2897226 | TFs and others | ESR1 | Breast | Breast cancer cells | 1264 | 7/31605 | -1.29/0.26 | -2.9 | -2.7 | -3.20 | FALSE |
| SRX2897228 | TFs and others | ESR1 | Breast | Breast cancer cells | 1236 | 7/31605 | -1.29/0.26 | -2.9 | -2.7 | -3.20 | FALSE |
| SRX11110957 | TFs and others | HDAC1 | Breast | MCF-7 | 903 | 15/31605 | -0.80/0.26 | -2.9 | -2.7 | -3.20 | FALSE |
| SRX969015 | TFs and others | ESR1 | Breast | MCF-7 | 1456 | 59/31605 | -0.28/0.26 | -2.8 | -2.7 | -3.19 | FALSE |
| SRX3070471 | TFs and others | NR3C1 | Breast | MCF 10A | 14158 | 1592/31605 | 0.15/0.26 | -2.8 | -2.7 | -3.19 | FALSE |
| SRX5574444 | TFs and others | EN1 | Breast | SUM 149PT | 1686 | 78/31605 | -0.21/0.26 | -2.8 | -2.7 | -3.18 | FALSE |
| SRX10119384 | TFs and others | JUNB | Breast | MCF 10A | 36658 | 2630/31605 | 0.18/0.26 | -2.8 | -2.7 | -3.18 | FALSE |
| SRX13327971 | TFs and others | XRCC6 | Breast | MCF-7 | 850 | 26/31605 | -0.54/0.26 | -2.8 | -2.7 | -3.18 | FALSE |
| SRX3589922 | TFs and others | STAG1 | Breast | MCF 10A | 723 | 33/31605 | -0.45/0.26 | -2.8 | -2.7 | -3.17 | FALSE |
| SRX1012695 | TFs and others | PGR | Breast | MCF-7 | 1386 | 47/31605 | -0.34/0.26 | -2.8 | -2.7 | -3.16 | FALSE |
| SRX3229133 | TFs and others | AR | Breast | Breast cancer | 1719 | 64/31605 | -0.25/0.26 | -2.8 | -2.7 | -3.16 | FALSE |
| SRX7030946 | TFs and others | SUV39H1 | Breast | MCF-7 | 817 | 17/31605 | -0.73/0.26 | -2.8 | -2.7 | -3.16 | FALSE |
| SRX7101366 | TFs and others | TEAD1 | Breast | Breast cancer cells | 852 | 8/31605 | -1.17/0.26 | -2.8 | -2.7 | -3.16 | FALSE |
| SRX3070476 | TFs and others | NR3C1 | Breast | MCF 10A | 1250 | 162/31605 | -0.06/0.26 | -2.8 | -2.7 | -3.15 | FALSE |
| SRX14960538 | TFs and others | EZH2 | Breast | MCF-7 | 371 | 59/31605 | 0.78/0.26 | -2.8 | -2.7 | 3.15 | TRUE |
| SRX1709972 | TFs and others | ESR1 | Breast | T-47D | 706 | 9/31605 | -1.09/0.26 | -2.8 | -2.7 | -3.15 | FALSE |
| SRX3990978 | TFs and others | PGR | Breast | T-47D | 684 | 7/31605 | -1.27/0.26 | -2.8 | -2.7 | -3.15 | FALSE |
| SRX7366781 | TFs and others | CDK9 | Breast | MCF-7 | 741 | 132/31605 | -0.10/0.26 | -2.8 | -2.7 | -3.15 | FALSE |
| SRX6860689 | TFs and others | RNF2 | Breast | T-47D | 985 | 8/31605 | -1.17/0.26 | -2.8 | -2.7 | -3.14 | FALSE |
| SRX5323855 | TFs and others | STAT3 | Breast | MCF-7 | 355 | 47/31605 | -0.33/0.26 | -2.8 | -2.7 | -3.14 | FALSE |
| SRX3589917 | TFs and others | STAG1 | Breast | MCF 10A | 2462 | 325/31605 | 0.03/0.26 | -2.8 | -2.7 | -3.14 | FALSE |
| SRX9046258 | TFs and others | FOSL2 | Breast | MCF-7 | 537 | 115/31605 | -0.12/0.26 | -2.8 | -2.7 | -3.14 | FALSE |
| SRX1709983 | TFs and others | PGR | Breast | T-47D | 867 | 12/31605 | -0.90/0.26 | -2.7 | -2.6 | -3.12 | FALSE |
| SRX10024461 | TFs and others | ESR1 | Breast | MCF-7 | 1475 | 15/31605 | -0.78/0.26 | -2.7 | -2.6 | -3.12 | FALSE |
| SRX2897231 | TFs and others | ESR1 | Breast | Breast cancer cells | 1873 | 7/31605 | -1.25/0.26 | -2.7 | -2.6 | -3.11 | FALSE |
| SRX1161164 | TFs and others | NR3C1 | Breast | MCF-7 | 416 | 11/31605 | -0.95/0.26 | -2.7 | -2.6 | -3.11 | FALSE |
| SRX15235733 | TFs and others | PRRX1 | Breast | UACC-3199 | 854 | 23/31605 | -0.58/0.26 | -2.7 | -2.6 | -3.11 | FALSE |
| SRX4017880 | TFs and others | TP63 | Breast | Breast organoids | 945 | 8/31605 | -1.15/0.26 | -2.7 | -2.6 | -3.11 | FALSE |
| SRX7030920 | TFs and others | KDM4B | Breast | MCF-7 | 860 | 9/31605 | -1.07/0.26 | -2.7 | -2.6 | -3.11 | FALSE |
| SRX8617941 | TFs and others | FGFR1 | Breast | CAMA-1 | 1570 | 115/31605 | -0.12/0.26 | -2.7 | -2.6 | -3.11 | FALSE |
| SRX3110737 | TFs and others | ESR1 | Breast | MCF-7 | 85 | 4/31605 | -1.73/0.26 | -2.7 | -2.6 | -3.11 | FALSE |
| SRX2423661 | TFs and others | ZBTB33 | Breast | MCF-7 | 1953 | 151/31605 | -0.07/0.26 | -2.7 | -2.6 | -3.10 | FALSE |
| SRX3229130 | TFs and others | FOXA1 | Breast | Breast cancer | 653 | 15/31605 | -0.77/0.26 | -2.7 | -2.6 | -3.10 | FALSE |
| SRX1995220 | TFs and others | ESR1 | Breast | MCF-7 | 346 | 7/31605 | -1.25/0.26 | -2.7 | -2.6 | -3.10 | FALSE |
| SRX7366783 | TFs and others | CDK9 | Breast | MCF-7 | 765 | 141/31605 | -0.08/0.26 | -2.7 | -2.6 | -3.10 | FALSE |
| SRX155761 | TFs and others | HSF1 | Breast | BT-20 | 751 | 182/31605 | -0.04/0.26 | -2.7 | -2.6 | -3.10 | FALSE |
| SRX1131553 | TFs and others | BRD4 | Breast | MDA-MB-436 | 2685 | 45/31605 | -0.34/0.26 | -2.7 | -2.6 | -3.09 | FALSE |
| SRX9697816 | TFs and others | Epitope tags | Breast | MDA-MB-231 | 911 | 7/31605 | -1.24/0.26 | -2.7 | -2.6 | -3.09 | FALSE |
| SRX9697822 | TFs and others | Epitope tags | Breast | MDA-MB-231 | 849 | 7/31605 | -1.24/0.26 | -2.7 | -2.6 | -3.09 | FALSE |
| SRX899076 | TFs and others | TP53 | Breast | Breast cancer cells | 713 | 43/31605 | -0.35/0.26 | -2.7 | -2.6 | -3.09 | FALSE |
| SRX19210551 | TFs and others | RELB | Breast | MDA-MB-231 | 755 | 38/31605 | -0.39/0.26 | -2.7 | -2.6 | -3.09 | FALSE |
| SRX730445 | TFs and others | EP300 | Breast | MCF-7 | 1163 | 268/31605 | 0.01/0.26 | -2.7 | -2.6 | -3.08 | FALSE |
| SRX15235737 | TFs and others | PRRX1 | Breast | HDQ-P1 | 729 | 12/31605 | -0.88/0.26 | -2.7 | -2.6 | -3.08 | FALSE |
| SRX3322255 | TFs and others | NFXL1 | Breast | MCF-7 | 1641 | 60/31605 | -0.25/0.26 | -2.7 | -2.6 | -3.07 | FALSE |
| SRX14612969 | TFs and others | FOXA1 | Breast | T-47D | 802 | 7/31605 | -1.23/0.26 | -2.7 | -2.5 | -3.06 | FALSE |
| SRX730441 | TFs and others | EP300 | Breast | MCF-7 | 937 | 623/31605 | 0.10/0.26 | -2.6 | -2.5 | -3.06 | FALSE |
| SRX12019743 | TFs and others | STAT2 | Breast | MCF-7 | 702 | 13/31605 | -0.83/0.26 | -2.6 | -2.5 | -3.06 | FALSE |
| SRX10664195 | TFs and others | CTR9 | Breast | MCF-7 | 3988 | 151/31605 | -0.06/0.26 | -2.6 | -2.5 | -3.05 | FALSE |
| SRX16940291 | TFs and others | MAF | Breast | MCF-7 | 802 | 12/31605 | -0.87/0.26 | -2.6 | -2.5 | -3.05 | FALSE |
| SRX7030880 | TFs and others | EHMT2 | Breast | MCF-7 | 3266 | 505/31605 | 0.43/0.26 | -2.6 | -2.5 | 3.04 | TRUE |
| SRX3070475 | TFs and others | NR3C1 | Breast | MCF 10A | 11752 | 1312/31605 | 0.15/0.26 | -2.6 | -2.5 | -3.03 | FALSE |
| SRX3833328 | TFs and others | KDM1A | Breast | MCF-7 | 250 | 12/31605 | -0.86/0.26 | -2.6 | -2.5 | -3.03 | FALSE |
| SRX22289052 | TFs and others | LMNB1 | Breast | MCF 10A | 1135 | 6/31605 | -1.33/0.26 | -2.6 | -2.5 | -3.03 | FALSE |
| SRX22289063 | TFs and others | LMNB1 | Breast | MCF 10A | 1445 | 6/31605 | -1.33/0.26 | -2.6 | -2.5 | -3.03 | FALSE |
| SRX22289065 | TFs and others | LMNB1 | Breast | MCF 10A | 1239 | 6/31605 | -1.33/0.26 | -2.6 | -2.5 | -3.03 | FALSE |
| SRX9528949 | TFs and others | Epitope tags | Breast | MCF-7 | 1087 | 10/31605 | -0.97/0.26 | -2.6 | -2.5 | -3.02 | FALSE |
| SRX14613001 | TFs and others | SMARCC1 | Breast | T-47D | 1118 | 6/31605 | -1.33/0.26 | -2.6 | -2.5 | -3.02 | FALSE |
| SRX14613002 | TFs and others | SMARCC1 | Breast | T-47D | 1146 | 6/31605 | -1.33/0.26 | -2.6 | -2.5 | -3.02 | FALSE |
| SRX2897229 | TFs and others | ESR1 | Breast | Breast cancer cells | 961 | 6/31605 | -1.33/0.26 | -2.6 | -2.5 | -3.02 | FALSE |
| SRX2897230 | TFs and others | ESR1 | Breast | Breast cancer cells | 1569 | 6/31605 | -1.33/0.26 | -2.6 | -2.5 | -3.02 | FALSE |
| SRX6860622 | TFs and others | RNF2 | Breast | T-47D | 1106 | 6/31605 | -1.33/0.26 | -2.6 | -2.5 | -3.02 | FALSE |
| SRX6860694 | TFs and others | RNF2 | Breast | T-47D | 1115 | 6/31605 | -1.33/0.26 | -2.6 | -2.5 | -3.02 | FALSE |
| SRX14756934 | TFs and others | DNA-RNA hybrid | Breast | MDA-MB-436 | 696 | 115/31605 | -0.11/0.26 | -2.6 | -2.5 | -3.02 | FALSE |
| SRX7030948 | TFs and others | SUV39H1 | Breast | MCF-7 | 735 | 8/31605 | -1.11/0.26 | -2.6 | -2.5 | -3.02 | FALSE |
| SRX7366782 | TFs and others | CDK9 | Breast | MCF-7 | 560 | 97/31605 | -0.14/0.26 | -2.6 | -2.5 | -3.02 | FALSE |
| SRX19210552 | TFs and others | RELB | Breast | MDA-MB-231 | 590 | 52/31605 | -0.28/0.26 | -2.6 | -2.5 | -3.02 | FALSE |
| SRX5089505 | TFs and others | HDAC1 | Breast | MCF-7 | 798 | 13/31605 | -0.81/0.26 | -2.6 | -2.5 | -3.01 | FALSE |
| SRX1772418 | TFs and others | NEDD4 | Breast | MCF-7 | 966 | 11/31605 | -0.90/0.26 | -2.6 | -2.5 | -3.00 | FALSE |
| SRX14612970 | TFs and others | FOXA1 | Breast | T-47D | 921 | 8/31605 | -1.10/0.26 | -2.6 | -2.5 | -3.00 | FALSE |
| SRX1709974 | TFs and others | ESR1 | Breast | T-47D | 458 | 18/31605 | -0.65/0.26 | -2.6 | -2.5 | -3.00 | FALSE |
| SRX2588916 | TFs and others | KMT2D | Breast | T-47D | 2308 | 32/31605 | -0.42/0.26 | -2.6 | -2.5 | -3.00 | FALSE |
| SRX7030921 | TFs and others | KDM4B | Breast | MCF-7 | 644 | 8/31605 | -1.10/0.26 | -2.6 | -2.5 | -2.99 | FALSE |
| SRX7865941 | TFs and others | EN1 | Breast | MDA-MB-231 | 1422 | 11/31605 | -0.90/0.26 | -2.6 | -2.5 | -2.99 | FALSE |
| SRX7865938 | TFs and others | EN1 | Breast | MDA-MB-231 | 1273 | 11/31605 | -0.90/0.26 | -2.6 | -2.5 | -2.99 | FALSE |
| SRX5954737 | TFs and others | FOSL2 | Breast | MDA-MB-231 | 10567 | 974/31605 | 0.13/0.26 | -2.6 | -2.5 | -2.99 | FALSE |
| SRX3589927 | TFs and others | STAG2 | Breast | MCF 10A | 1373 | 521/31605 | 0.09/0.26 | -2.6 | -2.5 | -2.99 | FALSE |
| SRX22289061 | TFs and others | LMNB1 | Breast | MCF 10A | 1418 | 8/31605 | -1.10/0.26 | -2.6 | -2.4 | -2.99 | FALSE |
| SRX5057609 | TFs and others | GATA3 | Breast | T-47D | 628 | 13/31605 | -0.81/0.26 | -2.6 | -2.4 | -2.99 | FALSE |
| SRX5323895 | TFs and others | STAT3 | Breast | T-47D | 1038 | 16/31605 | -0.70/0.26 | -2.6 | -2.4 | -2.99 | FALSE |
| SRX5057625 | TFs and others | GATA3 | Breast | T-47D | 865 | 26/31605 | -0.49/0.26 | -2.5 | -2.4 | -2.98 | FALSE |
| SRX7030881 | TFs and others | EHMT2 | Breast | MCF-7 | 3025 | 503/31605 | 0.43/0.26 | -2.5 | -2.4 | 2.98 | TRUE |
| SRX10775017 | TFs and others | RNF2 | Breast | T-47D | 1234 | 18/31605 | -0.64/0.26 | -2.5 | -2.4 | -2.98 | FALSE |
| SRX1995228 | TFs and others | LMNB1 | Breast | MCF-7 | 558 | 14/31605 | -0.76/0.26 | -2.5 | -2.4 | -2.97 | FALSE |
| SRX4417025 | TFs and others | GATA3 | Breast | MCF-7 | 1368 | 14/31605 | -0.76/0.26 | -2.5 | -2.4 | -2.97 | FALSE |
| SRX13327957 | TFs and others | CBX3 | Breast | MCF-7 | 1348 | 58/31605 | -0.24/0.26 | -2.5 | -2.4 | -2.96 | FALSE |
| SRX5089784 | TFs and others | SATB1 | Breast | MCF 10A | 31201 | 1065/31605 | 0.14/0.26 | -2.5 | -2.4 | -2.96 | FALSE |
| SRX7644287 | TFs and others | CPSF3L | Breast | MCF-7 | 311 | 3/31605 | -1.93/0.26 | -2.5 | -2.4 | -2.96 | FALSE |
| SRX15929735 | TFs and others | STAG1 | Breast | MCF 10A | 4425 | 519/31605 | 0.09/0.26 | -2.5 | -2.4 | -2.96 | FALSE |
| SRX19043739 | TFs and others | HOMEZ | Breast | MCF-7 | 1465 | 105/31605 | -0.11/0.26 | -2.5 | -2.4 | -2.96 | FALSE |
| SRX18895261 | TFs and others | MECP2 | Breast | MCF-7 | 1657 | 16/31605 | -0.69/0.26 | -2.5 | -2.4 | -2.96 | FALSE |
| SRX2460063 | TFs and others | PGR | Breast | Breast cancer cells | 261 | 29/31605 | -0.45/0.26 | -2.5 | -2.4 | -2.96 | FALSE |
| SRX5323817 | TFs and others | STAT3 | Breast | T-47D | 645 | 9/31605 | -1.01/0.26 | -2.5 | -2.4 | -2.95 | FALSE |
| SRX5287709 | TFs and others | Biotin | Breast | MCF-7 | 619 | 15/31605 | -0.72/0.26 | -2.5 | -2.4 | -2.95 | FALSE |
| SRX4116042 | TFs and others | ESR1 | Breast | Breast cancer cells | 216 | 12/31605 | -0.83/0.26 | -2.5 | -2.4 | -2.95 | FALSE |
| SRX10119379 | TFs and others | STAT3 | Breast | MCF 10A | 1011 | 139/31605 | -0.06/0.26 | -2.5 | -2.4 | -2.95 | FALSE |
| SRX7030914 | TFs and others | CBX5 | Breast | MCF-7 | 2073 | 22/31605 | -0.55/0.26 | -2.5 | -2.4 | -2.95 | FALSE |
| SRX9688333 | TFs and others | ESR1 | Breast | ZR-75-1 | 646 | 4/31605 | -1.63/0.26 | -2.5 | -2.4 | -2.94 | FALSE |
| SRX1709971 | TFs and others | PGR | Breast | T-47D | 669 | 10/31605 | -0.94/0.26 | -2.5 | -2.4 | -2.94 | FALSE |
| SRX19024867 | TFs and others | NR3C1 | Breast | MCF-7 | 724 | 9/31605 | -1.00/0.26 | -2.5 | -2.4 | -2.94 | FALSE |
| SRX2423659 | TFs and others | ARID3A | Breast | MCF-7 | 1636 | 31/31605 | -0.42/0.26 | -2.5 | -2.4 | -2.93 | FALSE |
| SRX5057607 | TFs and others | Epitope tags | Breast | T-47D | 529 | 8/31605 | -1.07/0.26 | -2.5 | -2.4 | -2.93 | FALSE |
| SRX155725 | TFs and others | HSF1 | Breast | BPLER | 470 | 108/31605 | -0.11/0.26 | -2.5 | -2.4 | -2.93 | FALSE |
| SRX10239460 | TFs and others | YTHDC1 | Breast | MCF-7 | 152 | 58/31605 | -0.24/0.26 | -2.5 | -2.4 | -2.93 | FALSE |
| SRX1936521 | TFs and others | SMARCA4 | Breast | T-47D | 1053 | 14/31605 | -0.75/0.26 | -2.5 | -2.4 | -2.93 | FALSE |
| SRX2513057 | TFs and others | MEN1 | Breast | MCF 10A | 2347 | 8/31605 | -1.07/0.26 | -2.5 | -2.4 | -2.93 | FALSE |
| SRX14612999 | TFs and others | RCOR1 | Breast | T-47D | 1255 | 7/31605 | -1.16/0.26 | -2.5 | -2.4 | -2.93 | FALSE |
| SRX8353667 | TFs and others | LMNA | Breast | MDA-MB-231 | 592 | 7/31605 | -1.16/0.26 | -2.5 | -2.4 | -2.92 | FALSE |
| SRX4017886 | TFs and others | TP63 | Breast | Breast organoids | 640 | 7/31605 | -1.16/0.26 | -2.5 | -2.4 | -2.92 | FALSE |
| SRX2194267 | TFs and others | EP300 | Breast | SUM 159PT | 17296 | 1252/31605 | 0.15/0.26 | -2.5 | -2.4 | -2.92 | FALSE |
| SRX3080247 | TFs and others | BRD4 | Breast | MDA-MB-231 | 1135 | 9/31605 | -0.99/0.26 | -2.5 | -2.4 | -2.92 | FALSE |
| SRX3322226 | TFs and others | ZBTB1 | Breast | MCF-7 | 1148 | 23/31605 | -0.52/0.26 | -2.5 | -2.4 | -2.92 | FALSE |
| SRX1012615 | TFs and others | PGR | Breast | T-47D | 790 | 7/31605 | -1.16/0.26 | -2.4 | -2.3 | -2.91 | FALSE |
| SRX4896697 | TFs and others | RUNX1 | Breast | MCF 10A | 1193 | 147/31605 | -0.05/0.26 | -2.4 | -2.3 | -2.91 | FALSE |
| SRX5511311 | TFs and others | ESR1 | Breast | MDA-MB-231 | 839 | 4/31605 | -1.61/0.26 | -2.4 | -2.3 | -2.91 | FALSE |
| SRX4017883 | TFs and others | TP63 | Breast | Breast organoids | 1811 | 12/31605 | -0.82/0.26 | -2.4 | -2.3 | -2.91 | FALSE |
| SRX734339 | TFs and others | CHD8 | Breast | T-47D | 39931 | 2073/31605 | 0.34/0.26 | -2.4 | -2.3 | 2.91 | TRUE |
| SRX734340 | TFs and others | CHD8 | Breast | T-47D | 81285 | 3980/31605 | 0.31/0.26 | -2.4 | -2.3 | 2.91 | TRUE |
| SRX2578882 | TFs and others | MED1 | Breast | MDA-MB-231 | 491 | 32/31605 | -0.40/0.26 | -2.4 | -2.3 | -2.90 | FALSE |
| SRX3322529 | TFs and others | MNT | Breast | MCF-7 | 1917 | 11/31605 | -0.86/0.26 | -2.4 | -2.3 | -2.90 | FALSE |
| SRX5493754 | TFs and others | AR | Breast | Breast cancer cells | 433 | 41/31605 | -0.32/0.26 | -2.4 | -2.3 | -2.90 | FALSE |
| SRX16341656 | TFs and others | KDM1A | Breast | T-47D | 1452 | 104/31605 | -0.11/0.26 | -2.4 | -2.3 | -2.90 | FALSE |
| SRX5057612 | TFs and others | GATA3 | Breast | T-47D | 850 | 32/31605 | -0.40/0.26 | -2.4 | -2.3 | -2.89 | FALSE |
| SRX17767565 | TFs and others | Epitope tags | Breast | MCF-7 | 1071 | 82/31605 | -0.15/0.26 | -2.4 | -2.3 | -2.89 | FALSE |
| SRX17657163 | TFs and others | FOXC1 | Breast | MDA-MB-468 | 6451 | 632/31605 | 0.11/0.26 | -2.4 | -2.3 | -2.89 | FALSE |
| SRX14612971 | TFs and others | FOXA1 | Breast | T-47D | 781 | 8/31605 | -1.05/0.26 | -2.4 | -2.3 | -2.89 | FALSE |
| SRX1272813 | TFs and others | ESR1 | Breast | MCF-7 | 29 | 1/31605 | -3.44/0.26 | -2.4 | -2.3 | -2.88 | FALSE |
| SRX4017882 | TFs and others | TP63 | Breast | Breast organoids | 2313 | 8/31605 | -1.05/0.26 | -2.4 | -2.3 | -2.88 | FALSE |
| SRX1012764 | TFs and others | PGR | Breast | MCF-7 | 1094 | 18/31605 | -0.61/0.26 | -2.4 | -2.3 | -2.88 | FALSE |
| SRX042343 | TFs and others | ESR1 | Breast | HCC1428-LTED | 723 | 10/31605 | -0.91/0.26 | -2.4 | -2.3 | -2.88 | FALSE |
| SRX11110959 | TFs and others | HDAC1 | Breast | MCF-7 | 726 | 17/31605 | -0.64/0.26 | -2.4 | -2.3 | -2.88 | FALSE |
| SRX4786571 | TFs and others | TET2 | Breast | MCF-7 | 753 | 4/31605 | -1.59/0.26 | -2.4 | -2.3 | -2.88 | FALSE |
| SRX2880132 | TFs and others | KMT2C | Breast | CAL-51 | 928 | 77/31605 | -0.16/0.26 | -2.4 | -2.3 | -2.87 | FALSE |
| SRX9739052 | TFs and others | NKRF | Breast | MDA-MB-231 | 763 | 8/31605 | -1.05/0.26 | -2.4 | -2.3 | -2.87 | FALSE |
| SRX4909433 | TFs and others | MTA2 | Breast | HCC3153 | 1185 | 78/31605 | -0.16/0.26 | -2.4 | -2.3 | -2.87 | FALSE |
| SRX4714451 | TFs and others | GTF3C2 | Breast | T-47D | 2231 | 78/31605 | -0.16/0.26 | -2.4 | -2.3 | -2.87 | FALSE |
| SRX9739054 | TFs and others | NKRF | Breast | MDA-MB-231 | 223 | 8/31605 | -1.04/0.26 | -2.4 | -2.3 | -2.86 | FALSE |
| SRX9932079 | TFs and others | HMGN2 | Breast | MCF-7 | 2328 | 19/31605 | -0.59/0.26 | -2.4 | -2.3 | -2.86 | FALSE |
| SRX647705 | TFs and others | TP53 | Breast | MDA-MB-468 | 2252 | 21/31605 | -0.54/0.26 | -2.4 | -2.3 | -2.86 | FALSE |
| SRX5503019 | TFs and others | E4F1 | Breast | SUM 159PT | 1876 | 49/31605 | -0.27/0.26 | -2.4 | -2.3 | -2.86 | FALSE |
| SRX1772419 | TFs and others | NEDD4 | Breast | MCF-7 | 1003 | 14/31605 | -0.72/0.26 | -2.4 | -2.3 | -2.85 | FALSE |
| SRX15039977 | TFs and others | Epitope tags | Breast | MDA-MB-231 | 2507 | 246/31605 | 0.02/0.26 | -2.4 | -2.3 | -2.85 | FALSE |
| SRX3428655 | TFs and others | ESR1 | Breast | MCF-7 | 1087 | 17/31605 | -0.63/0.26 | -2.4 | -2.3 | -2.85 | FALSE |
| SRX18191656 | TFs and others | ESR1 | Breast | MCF-7 | 727 | 21/31605 | -0.54/0.26 | -2.4 | -2.3 | -2.85 | FALSE |
| SRX13254863 | TFs and others | SUZ12 | Breast | MCF-7 | 44481 | 4042/31605 | 0.20/0.26 | -2.3 | -2.3 | -2.84 | FALSE |
| SRX8023140 | TFs and others | TP53 | Breast | MCF 10A | 3568 | 299/31605 | 0.04/0.26 | -2.3 | -2.3 | -2.84 | FALSE |
| SRX2578877 | TFs and others | ESR1 | Breast | MDA-MB-231 | 635 | 12/31605 | -0.79/0.26 | -2.3 | -2.3 | -2.84 | FALSE |
| SRX2460067 | TFs and others | PGR | Breast | Breast cancer cells | 396 | 9/31605 | -0.95/0.26 | -2.3 | -2.2 | -2.83 | FALSE |
| SRX4284794 | TFs and others | Epitope tags | Breast | T-47D | 825 | 7/31605 | -1.11/0.26 | -2.3 | -2.2 | -2.83 | FALSE |
| SRX647707 | TFs and others | TP53 | Breast | BT-549 | 1491 | 7/31605 | -1.11/0.26 | -2.3 | -2.2 | -2.83 | FALSE |
| SRX23330421 | TFs and others | HDAC6 | Breast | MDA-MB-231 | 440 | 15/31605 | -0.68/0.26 | -2.3 | -2.2 | -2.83 | FALSE |
| SRX2109209 | TFs and others | MYC | Breast | Mammary epithelial cells | 761 | 72/31605 | -0.17/0.26 | -2.3 | -2.2 | -2.83 | FALSE |
| SRX7522858 | TFs and others | BCL9 | Breast | MCF10DCIS.com | 1701 | 202/31605 | 0.00/0.26 | -2.3 | -2.2 | -2.82 | FALSE |
| SRX039141 | TFs and others | JUN | Breast | MCF-7 | 363 | 25/31605 | -0.47/0.26 | -2.3 | -2.2 | -2.82 | FALSE |
| SRX1012715 | TFs and others | PGR | Breast | T-47D | 1688 | 13/31605 | -0.75/0.26 | -2.3 | -2.2 | -2.82 | FALSE |
| SRX20152808 | TFs and others | Epitope tags | Breast | MDA-MB-231 | 645 | 5/31605 | -1.36/0.26 | -2.3 | -2.2 | -2.81 | FALSE |
| SRX471210 | TFs and others | MBD2 | Breast | MCF-7 | 3554 | 1041/31605 | 0.37/0.26 | -2.3 | -2.2 | 2.81 | TRUE |
| SRX1339016 | TFs and others | AR | Breast | MDA-MB-453 | 407 | 6/31605 | -1.22/0.26 | -2.3 | -2.2 | -2.81 | FALSE |
| SRX5287712 | TFs and others | Biotin | Breast | MCF-7 | 646 | 10/31605 | -0.88/0.26 | -2.3 | -2.2 | -2.81 | FALSE |
| SRX155760 | TFs and others | HSF1 | Breast | BT-20 | 510 | 133/31605 | -0.06/0.26 | -2.3 | -2.2 | -2.81 | FALSE |
| SRX13323812 | TFs and others | GFP | Breast | MDA-MB-231 | 1549 | 22/31605 | -0.51/0.26 | -2.3 | -2.2 | -2.81 | FALSE |
| SRX5185602 | TFs and others | MED12 | Breast | MCF-7 | 676 | 15/31605 | -0.67/0.26 | -2.3 | -2.2 | -2.81 | FALSE |
| SRX1044416 | TFs and others | RELA | Breast | MDA-MB-231 | 1312 | 46/31605 | -0.27/0.26 | -2.3 | -2.2 | -2.80 | FALSE |
| SRX155717 | TFs and others | HSF1 | Breast | BPE | 212 | 3/31605 | -1.81/0.26 | -2.3 | -2.2 | -2.80 | FALSE |
| SRX471215 | TFs and others | MBD2 | Breast | MCF-7 | 195 | 3/31605 | -1.81/0.26 | -2.3 | -2.2 | -2.80 | FALSE |
| SRX518263 | TFs and others | AGO1 | Breast | MCF-7 | 85 | 3/31605 | -1.81/0.26 | -2.3 | -2.2 | -2.80 | FALSE |
| SRX1709973 | TFs and others | PGR | Breast | T-47D | 595 | 7/31605 | -1.10/0.26 | -2.3 | -2.2 | -2.80 | FALSE |
| SRX647706 | TFs and others | TP53 | Breast | HCC70 | 1688 | 12/31605 | -0.78/0.26 | -2.3 | -2.2 | -2.79 | FALSE |
| SRX5089409 | TFs and others | SMARCA4 | Breast | MCF-7 | 1175 | 145/31605 | -0.04/0.26 | -2.3 | -2.2 | -2.79 | FALSE |
| SRX5323901 | TFs and others | STAT3 | Breast | T-47D | 1057 | 36/31605 | -0.34/0.26 | -2.3 | -2.2 | -2.78 | FALSE |
| SRX7657047 | TFs and others | NCOA3 | Breast | MCF 10A | 771 | 4/31605 | -1.52/0.26 | -2.3 | -2.2 | -2.78 | FALSE |
| SRX2935292 | TFs and others | 8-Hydroxydeoxyguanosine | Breast | MCF 10A | 90551 | 5857/31605 | 0.21/0.26 | -2.2 | -2.2 | -2.77 | FALSE |
| SRX14613015 | TFs and others | JUN | Breast | T-47D | 1158 | 10/31605 | -0.87/0.26 | -2.2 | -2.2 | -2.77 | FALSE |
| SRX5493746 | TFs and others | AR | Breast | Breast cancer cells | 715 | 49/31605 | -0.25/0.26 | -2.2 | -2.2 | -2.77 | FALSE |
| SRX2424124 | TFs and others | MTA2 | Breast | MCF-7 | 1230 | 21/31605 | -0.52/0.26 | -2.2 | -2.2 | -2.76 | FALSE |
| SRX16341657 | TFs and others | KDM1A | Breast | T-47D | 1336 | 99/31605 | -0.10/0.26 | -2.2 | -2.2 | -2.76 | FALSE |
| SRX13323813 | TFs and others | GFP | Breast | MDA-MB-231 | 8502 | 278/31605 | 0.04/0.26 | -2.2 | -2.1 | -2.76 | FALSE |
| SRX5503021 | TFs and others | E4F1 | Breast | SUM 159PT | 1931 | 59/31605 | -0.21/0.26 | -2.2 | -2.1 | -2.76 | FALSE |
| SRX1012691 | TFs and others | PGR | Breast | MCF-7 | 1261 | 47/31605 | -0.26/0.26 | -2.2 | -2.1 | -2.76 | FALSE |
| SRX036563 | TFs and others | ESR1 | Breast | MCF-7 | 101 | 3/31605 | -1.79/0.26 | -2.2 | -2.1 | -2.76 | FALSE |
| SRX4192133 | TFs and others | TOP1 | Breast | MCF 10A | 117 | 3/31605 | -1.79/0.26 | -2.2 | -2.1 | -2.76 | FALSE |
| SRX5089538 | TFs and others | ARID2 | Breast | MCF-7 | 1024 | 20/31605 | -0.53/0.26 | -2.2 | -2.1 | -2.75 | FALSE |
| SRX734342 | TFs and others | CHD8 | Breast | T-47D | 70220 | 3431/31605 | 0.32/0.26 | -2.2 | -2.1 | 2.75 | TRUE |
| SRX2060921 | TFs and others | TP53 | Breast | MCF-7 | 1562 | 60/31605 | -0.20/0.26 | -2.2 | -2.1 | -2.75 | FALSE |
| SRX4096887 | TFs and others | TRPS1 | Breast | HCC3153 | 2979 | 183/31605 | -0.00/0.26 | -2.2 | -2.1 | -2.74 | FALSE |
| SRX1709976 | TFs and others | ESR1 | Breast | T-47D | 380 | 5/31605 | -1.31/0.26 | -2.2 | -2.1 | -2.74 | FALSE |
| SRX3862451 | TFs and others | FOXA1 | Breast | T-47D | 837 | 45/31605 | -0.27/0.26 | -2.2 | -2.1 | -2.74 | FALSE |
| SRX15929739 | TFs and others | STAG2 | Breast | MCF 10A | 1318 | 269/31605 | 0.04/0.26 | -2.2 | -2.1 | -2.74 | FALSE |
| SRX2423658 | TFs and others | ARID3A | Breast | MCF-7 | 1667 | 19/31605 | -0.55/0.26 | -2.2 | -2.1 | -2.74 | FALSE |
| SRX5574445 | TFs and others | EN1 | Breast | SUM 159PT | 1191 | 87/31605 | -0.12/0.26 | -2.2 | -2.1 | -2.73 | FALSE |
| SRX2897236 | TFs and others | ESR1 | Breast | Breast cancer cells | 3309 | 13/31605 | -0.71/0.26 | -2.2 | -2.1 | -2.73 | FALSE |
| SRX9528951 | TFs and others | Epitope tags | Breast | MCF-7 | 928 | 6/31605 | -1.17/0.26 | -2.2 | -2.1 | -2.73 | FALSE |
| SRX6098151 | TFs and others | TRPS1 | Breast | MCF-7 | 684 | 6/31605 | -1.17/0.26 | -2.2 | -2.1 | -2.73 | FALSE |
| SRX17657158 | TFs and others | FOXC1 | Breast | MDA-MB-231 | 3427 | 421/31605 | 0.08/0.26 | -2.2 | -2.1 | -2.73 | FALSE |
| SRX8272754 | TFs and others | ESR2 | Breast | MCF-7 | 1369 | 13/31605 | -0.71/0.26 | -2.2 | -2.1 | -2.72 | FALSE |
| SRX155754 | TFs and others | HSF1 | Breast | SK-BR-3 | 648 | 172/31605 | -0.01/0.26 | -2.2 | -2.1 | -2.72 | FALSE |
| SRX18191659 | TFs and others | ESR1 | Breast | MCF-7 | 821 | 28/31605 | -0.40/0.26 | -2.2 | -2.1 | -2.72 | FALSE |
| SRX1212810 | TFs and others | CARM1 | Breast | MCF-7 | 886 | 15/31605 | -0.64/0.26 | -2.2 | -2.1 | -2.72 | FALSE |
| SRX20830487 | TFs and others | YAP1 | Breast | T-47D | 262 | 6/31605 | -1.17/0.26 | -2.2 | -2.1 | -2.72 | FALSE |
| SRX1521315 | TFs and others | KDM5C | Breast | ZR-75-30 | 64 | 4/31605 | -1.48/0.26 | -2.2 | -2.1 | -2.71 | FALSE |
| SRX4786572 | TFs and others | TET2 | Breast | MCF-7 | 794 | 5/31605 | -1.29/0.26 | -2.2 | -2.1 | -2.70 | FALSE |
| SRX12813814 | TFs and others | TRIM28 | Breast | MCF-7 | 4619 | 118/31605 | -0.06/0.26 | -2.2 | -2.1 | -2.70 | FALSE |
| SRX673732 | TFs and others | PRKDC | Breast | MCF-7 | 816 | 26/31605 | -0.42/0.26 | -2.2 | -2.1 | -2.70 | FALSE |
| SRX3229191 | TFs and others | PGR | Breast | Breast cancer | 737 | 7/31605 | -1.05/0.26 | -2.2 | -2.1 | -2.70 | FALSE |
| SRX4561217 | TFs and others | CDC16 | Breast | MCF-7 | 2124 | 14/31605 | -0.67/0.26 | -2.2 | -2.1 | -2.70 | FALSE |
| SRX4561221 | TFs and others | CDC16 | Breast | MCF-7 | 2004 | 12/31605 | -0.74/0.26 | -2.2 | -2.1 | -2.70 | FALSE |
| SRX1131552 | TFs and others | BRD4 | Breast | MDA-MB-436 | 567 | 110/31605 | -0.07/0.26 | -2.2 | -2.1 | -2.70 | FALSE |
| SRX950756 | TFs and others | TOP2B | Breast | MCF-7 | 1115 | 15/31605 | -0.64/0.26 | -2.2 | -2.1 | -2.70 | FALSE |
| SRX2460055 | TFs and others | PGR | Breast | Breast cancer cells | 405 | 45/31605 | -0.26/0.26 | -2.2 | -2.1 | -2.69 | FALSE |
| SRX495669 | TFs and others | NR3C1 | Breast | MDA-MB-231 | 3908 | 207/31605 | 0.02/0.26 | -2.2 | -2.1 | -2.69 | FALSE |
| SRX7692442 | TFs and others | POLR2C | Breast | MCF-7 | 1097 | 8/31605 | -0.96/0.26 | -2.1 | -2.1 | -2.69 | FALSE |
| SRX7692445 | TFs and others | POLR2C | Breast | MCF-7 | 1203 | 8/31605 | -0.96/0.26 | -2.1 | -2.1 | -2.69 | FALSE |
| SRX10664183 | TFs and others | SUZ12 | Breast | MCF-7 | 2103 | 146/31605 | -0.03/0.26 | -2.1 | -2.1 | -2.69 | FALSE |
| SRX7030929 | TFs and others | NELFA | Breast | MCF-7 | 723 | 13/31605 | -0.70/0.26 | -2.1 | -2.1 | -2.69 | FALSE |
| SRX1012712 | TFs and others | PGR | Breast | T-47D | 1040 | 9/31605 | -0.89/0.26 | -2.1 | -2.1 | -2.69 | FALSE |
| SRX1995222 | TFs and others | ESR1 | Breast | MCF-7 | 217 | 3/31605 | -1.73/0.26 | -2.1 | -2.1 | -2.69 | FALSE |
| SRX1995224 | TFs and others | FOXA1 | Breast | MCF-7 | 233 | 3/31605 | -1.73/0.26 | -2.1 | -2.1 | -2.69 | FALSE |
| SRX15039973 | TFs and others | Epitope tags | Breast | MDA-MB-231 | 2040 | 197/31605 | 0.01/0.26 | -2.1 | -2.0 | -2.68 | FALSE |
| SRX5057617 | TFs and others | Epitope tags | Breast | T-47D | 716 | 7/31605 | -1.04/0.26 | -2.1 | -2.0 | -2.68 | FALSE |
| SRX9322279 | TFs and others | ESR1 | Breast | MCF-7 | 687 | 14/31605 | -0.66/0.26 | -2.1 | -2.0 | -2.68 | FALSE |
| SRX5323899 | TFs and others | STAT3 | Breast | T-47D | 868 | 13/31605 | -0.70/0.26 | -2.1 | -2.0 | -2.68 | FALSE |
| SRX8202281 | TFs and others | CDK7 | Breast | MCF-7 | 2332 | 67/31605 | -0.16/0.26 | -2.1 | -2.0 | -2.68 | FALSE |
| SRX14284268 | TFs and others | ARID1A | Breast | HMEC | 656 | 4/31605 | -1.46/0.26 | -2.1 | -2.0 | -2.67 | FALSE |
| SRX2578886 | TFs and others | MED1 | Breast | MDA-MB-231 | 340 | 63/31605 | -0.18/0.26 | -2.1 | -2.0 | -2.67 | FALSE |
| SRX5089508 | TFs and others | HDAC1 | Breast | MCF-7 | 719 | 11/31605 | -0.77/0.26 | -2.1 | -2.0 | -2.67 | FALSE |
| SRX10859415 | TFs and others | SMARCE1 | Breast | BT-549 | 935 | 10/31605 | -0.82/0.26 | -2.1 | -2.0 | -2.66 | FALSE |
| SRX5057623 | TFs and others | GATA3 | Breast | T-47D | 784 | 21/31605 | -0.49/0.26 | -2.1 | -2.0 | -2.65 | FALSE |
| SRX4096857 | TFs and others | GATAD2A | Breast | HCC3153 | 1152 | 55/31605 | -0.20/0.26 | -2.1 | -2.0 | -2.65 | FALSE |
| SRX3428654 | TFs and others | ESR1 | Breast | MCF-7 | 873 | 10/31605 | -0.82/0.26 | -2.1 | -2.0 | -2.65 | FALSE |
| SRX15232272 | TFs and others | DNA-RNA hybrid | Breast | MDA-MB-436 | 540 | 8/31605 | -0.94/0.26 | -2.1 | -2.0 | -2.65 | FALSE |
| SRX5511313 | TFs and others | ESR1 | Breast | MDA-MB-231 | 727 | 5/31605 | -1.26/0.26 | -2.1 | -2.0 | -2.65 | FALSE |
| SRX6058768 | TFs and others | TOP2A | Breast | T-47D | 1823 | 16/31605 | -0.59/0.26 | -2.1 | -2.0 | -2.64 | FALSE |
| SRX9688332 | TFs and others | ESR1 | Breast | ZR-75-1 | 1258 | 11/31605 | -0.76/0.26 | -2.1 | -2.0 | -2.64 | FALSE |
| SRX730432 | TFs and others | NCAPG | Breast | MCF-7 | 588 | 5/31605 | -1.26/0.26 | -2.1 | -2.0 | -2.64 | FALSE |
| SRX730454 | TFs and others | NCAPG | Breast | MCF-7 | 551 | 5/31605 | -1.26/0.26 | -2.1 | -2.0 | -2.64 | FALSE |
| SRX10365527 | TFs and others | RAC1 | Breast | MDA-MB-231 | 844 | 71/31605 | -0.15/0.26 | -2.1 | -2.0 | -2.64 | FALSE |
| SRX5323833 | TFs and others | STAT3 | Breast | T-47D | 667 | 10/31605 | -0.81/0.26 | -2.1 | -2.0 | -2.64 | FALSE |
| SRX2578875 | TFs and others | ESR1 | Breast | MDA-MB-231 | 112 | 8/31605 | -0.94/0.26 | -2.1 | -2.0 | -2.63 | FALSE |
| SRX22289058 | TFs and others | LMNB1 | Breast | MCF 10A | 1179 | 7/31605 | -1.02/0.26 | -2.1 | -2.0 | -2.63 | FALSE |
| SRX026531 | TFs and others | ESR1 | Breast | T-47D | 1092 | 36/31605 | -0.31/0.26 | -2.1 | -2.0 | -2.63 | FALSE |
| SRX1012711 | TFs and others | PGR | Breast | T-47D | 1010 | 8/31605 | -0.93/0.26 | -2.1 | -2.0 | -2.63 | FALSE |
| SRX1012720 | TFs and others | PGR | Breast | T-47D | 1329 | 8/31605 | -0.93/0.26 | -2.1 | -2.0 | -2.63 | FALSE |
| SRX7030947 | TFs and others | SUV39H1 | Breast | MCF-7 | 903 | 8/31605 | -0.93/0.26 | -2.1 | -2.0 | -2.63 | FALSE |
| SRX5128107 | TFs and others | AR | Breast | ZR-75-1 | 2848 | 40/31605 | -0.28/0.26 | -2.1 | -2.0 | -2.63 | FALSE |
| SRX5202475 | TFs and others | CUL4B | Breast | MDA-MB-231 | 498 | 28/31605 | -0.38/0.26 | -2.1 | -2.0 | -2.62 | FALSE |
| SRX14612998 | TFs and others | JUN | Breast | T-47D | 1209 | 8/31605 | -0.93/0.26 | -2.1 | -2.0 | -2.62 | FALSE |
| SRX4815185 | TFs and others | EN1 | Breast | SUM 149PT | 1667 | 8/31605 | -0.93/0.26 | -2.1 | -2.0 | -2.62 | FALSE |
| SRX8023112 | TFs and others | TP53 | Breast | MCF 10A | 1896 | 93/31605 | -0.09/0.26 | -2.1 | -2.0 | -2.62 | FALSE |
| SRX18191657 | TFs and others | ESR1 | Breast | MCF-7 | 857 | 19/31605 | -0.51/0.26 | -2.1 | -2.0 | -2.62 | FALSE |
| SRX3546793 | TFs and others | Epitope tags | Breast | MDA-MB-231 | 150 | 9/31605 | -0.86/0.26 | -2.0 | -2.0 | -2.62 | FALSE |
| SRX13327961 | TFs and others | CBX3 | Breast | MCF-7 | 1572 | 57/31605 | -0.19/0.26 | -2.0 | -2.0 | -2.61 | FALSE |
| SRX4786547 | TFs and others | TET2 | Breast | MCF-7 | 856 | 5/31605 | -1.24/0.26 | -2.0 | -2.0 | -2.61 | FALSE |
| SRX20152809 | TFs and others | Epitope tags | Breast | MDA-MB-231 | 679 | 10/31605 | -0.80/0.26 | -2.0 | -2.0 | -2.60 | FALSE |
| SRX673729 | TFs and others | MED1 | Breast | MCF-7 | 979 | 7/31605 | -1.00/0.26 | -2.0 | -1.9 | -2.60 | FALSE |
| SRX673733 | TFs and others | PRKDC | Breast | MCF-7 | 756 | 7/31605 | -1.00/0.26 | -2.0 | -1.9 | -2.60 | FALSE |
| SRX1709961 | TFs and others | ESR1 | Breast | T-47D | 138 | 13/31605 | -0.67/0.26 | -2.0 | -1.9 | -2.60 | FALSE |
| SRX7897894 | TFs and others | EP300 | Breast | MDA-MB-231 | 6116 | 297/31605 | 0.45/0.26 | -2.0 | -1.9 | 2.60 | TRUE |
| SRX1995225 | TFs and others | FOXA1 | Breast | MCF-7 | 583 | 29/31605 | -0.36/0.26 | -2.0 | -1.9 | -2.60 | FALSE |
| SRX8957553 | TFs and others | YTHDC1 | Breast | MCF-7 | 413 | 25/31605 | -0.41/0.26 | -2.0 | -1.9 | -2.60 | FALSE |
| SRX7865937 | TFs and others | BRD4 | Breast | MDA-MB-231 | 7113 | 191/31605 | 0.01/0.26 | -2.0 | -1.9 | -2.59 | FALSE |
| SRX1466274 | TFs and others | PRMT5 | Breast | MDA-MB-231 | 2430 | 256/31605 | 0.05/0.26 | -2.0 | -1.9 | -2.59 | FALSE |
| SRX11650842 | TFs and others | SMARCA4 | Breast | CAL-120 | 1031 | 14/31605 | -0.63/0.26 | -2.0 | -1.9 | -2.59 | FALSE |
| SRX1012651 | TFs and others | PGR | Breast | MCF-7 | 278 | 8/31605 | -0.92/0.26 | -2.0 | -1.9 | -2.59 | FALSE |
| SRX1542106 | TFs and others | SRPK2 | Breast | MCF 10A | 222 | 4/31605 | -1.40/0.26 | -2.0 | -1.9 | -2.59 | FALSE |
| SRX667403 | TFs and others | Epitope tags | Breast | MCF-7 | 203 | 6/31605 | -1.09/0.26 | -2.0 | -1.9 | -2.58 | FALSE |
| SRX5323891 | TFs and others | STAT3 | Breast | T-47D | 973 | 13/31605 | -0.66/0.26 | -2.0 | -1.9 | -2.58 | FALSE |
| SRX3229194 | TFs and others | GATA3 | Breast | Breast cancer | 299 | 55/31605 | -0.19/0.26 | -2.0 | -1.9 | -2.58 | FALSE |
| SRX10977444 | TFs and others | ZNF227 | Breast | MCF-7 | 1386 | 40/31605 | -0.27/0.26 | -2.0 | -1.9 | -2.58 | FALSE |
| SRX3322779 | TFs and others | LARP7 | Breast | MCF-7 | 1206 | 17/31605 | -0.55/0.26 | -2.0 | -1.9 | -2.58 | FALSE |
| SRX10119385 | TFs and others | JUNB | Breast | MCF 10A | 28484 | 2084/31605 | 0.18/0.26 | -2.0 | -1.9 | -2.57 | FALSE |
| SRX7865931 | TFs and others | BRD4 | Breast | MDA-MB-231 | 1479 | 10/31605 | -0.79/0.26 | -2.0 | -1.9 | -2.57 | FALSE |
| SRX21436548 | TFs and others | TOP1 | Breast | MCF-7 | 1838 | 10/31605 | -0.78/0.26 | -2.0 | -1.9 | -2.57 | FALSE |
| SRX14612997 | TFs and others | JUN | Breast | T-47D | 1197 | 9/31605 | -0.84/0.26 | -2.0 | -1.9 | -2.56 | FALSE |
| SRX1012719 | TFs and others | PGR | Breast | T-47D | 1156 | 9/31605 | -0.84/0.26 | -2.0 | -1.9 | -2.56 | FALSE |
| SRX3010234 | TFs and others | EZH2 | Breast | MCF-7 | 214 | 6/31605 | -1.08/0.26 | -2.0 | -1.9 | -2.56 | FALSE |
| SRX5172068 | TFs and others | ESR1 | Breast | MCF-7 | 726 | 18/31605 | -0.51/0.26 | -2.0 | -1.9 | -2.55 | FALSE |
| SRX246860 | TFs and others | MBD3 | Breast | MCF-7 | 972 | 97/31605 | -0.08/0.26 | -2.0 | -1.9 | -2.55 | FALSE |
| SRX13845695 | TFs and others | HSF2 | Breast | MDA-MB-231 | 689 | 121/31605 | -0.04/0.26 | -2.0 | -1.9 | -2.55 | FALSE |
| SRX10024462 | TFs and others | ESR1 | Breast | MCF-7 | 968 | 5/31605 | -1.21/0.26 | -2.0 | -1.9 | -2.55 | FALSE |
| SRX1995219 | TFs and others | CTCF | Breast | MCF-7 | 271 | 5/31605 | -1.21/0.26 | -2.0 | -1.9 | -2.55 | FALSE |
| SRX155728 | TFs and others | HSF1 | Breast | MCF 10A | 341 | 6/31605 | -1.08/0.26 | -2.0 | -1.9 | -2.55 | FALSE |
| SRX673739 | TFs and others | ESR1 | Breast | MCF-7 | 966 | 15/31605 | -0.59/0.26 | -2.0 | -1.9 | -2.54 | FALSE |
| SRX8023134 | TFs and others | TP53 | Breast | MCF 10A | 1155 | 28/31605 | -0.36/0.26 | -2.0 | -1.9 | -2.54 | FALSE |
| SRX852986 | TFs and others | RUNX1 | Breast | MCF-7 | 1980 | 39/31605 | -0.27/0.26 | -2.0 | -1.9 | -2.54 | FALSE |
| SRX2578894 | TFs and others | NCOA1 | Breast | MDA-MB-231 | 376 | 14/31605 | -0.61/0.26 | -1.9 | -1.9 | -2.54 | FALSE |
| SRX1772412 | TFs and others | Epitope tags | Breast | MCF-7 | 1111 | 49/31605 | -0.21/0.26 | -1.9 | -1.9 | -2.53 | FALSE |
| SRX968972 | TFs and others | RELA | Breast | MCF-7 | 201 | 33/31605 | -0.31/0.26 | -1.9 | -1.9 | -2.53 | FALSE |
| SRX12193403 | TFs and others | ESR1 | Breast | MCF-7 | 303 | 6/31605 | -1.07/0.26 | -1.9 | -1.9 | -2.53 | FALSE |
| SRX23330419 | TFs and others | HDAC6 | Breast | MDA-MB-231 | 484 | 29/31605 | -0.35/0.26 | -1.9 | -1.9 | -2.53 | FALSE |
| SRX1936520 | TFs and others | SMARCA4 | Breast | T-47D | 1359 | 10/31605 | -0.77/0.26 | -1.9 | -1.9 | -2.53 | FALSE |
| SRX20830486 | TFs and others | YAP1 | Breast | T-47D | 275 | 4/31605 | -1.36/0.26 | -1.9 | -1.9 | -2.53 | FALSE |
| SRX1012699 | TFs and others | PGR | Breast | MCF-7 | 605 | 23/31605 | -0.42/0.26 | -1.9 | -1.9 | -2.52 | FALSE |
| SRX7030945 | TFs and others | SUV39H1 | Breast | MCF-7 | 769 | 9/31605 | -0.82/0.26 | -1.9 | -1.9 | -2.52 | FALSE |
| SRX4561219 | TFs and others | CDC16 | Breast | MCF-7 | 1925 | 12/31605 | -0.68/0.26 | -1.9 | -1.9 | -2.52 | FALSE |
| SRX2578918 | TFs and others | EP300 | Breast | MDA-MB-231 | 1106 | 15/31605 | -0.58/0.26 | -1.9 | -1.8 | -2.52 | FALSE |
| SRX2460047 | TFs and others | PGR | Breast | Breast cancer cells | 374 | 42/31605 | -0.24/0.26 | -1.9 | -1.8 | -2.52 | FALSE |
| SRX1012692 | TFs and others | PGR | Breast | MCF-7 | 841 | 17/31605 | -0.53/0.26 | -1.9 | -1.8 | -2.52 | FALSE |
| SRX14756941 | TFs and others | DNA-RNA hybrid | Breast | MDA-MB-436 | 7727 | 2613/31605 | 0.19/0.26 | -1.9 | -1.8 | -2.51 | FALSE |
| SRX12193405 | TFs and others | ESR1 | Breast | MCF-7 | 303 | 5/31605 | -1.18/0.26 | -1.9 | -1.8 | -2.51 | FALSE |
| SRX9605793 | TFs and others | TP53 | Breast | MCF 10A | 817 | 6/31605 | -1.06/0.26 | -1.9 | -1.8 | -2.51 | FALSE |
| SRX13300075 | TFs and others | CTCF | Breast | MCF 10A | 762 | 6/31605 | -1.05/0.26 | -1.9 | -1.8 | -2.51 | FALSE |
| SRX14613000 | TFs and others | KDM1A | Breast | T-47D | 1082 | 11/31605 | -0.71/0.26 | -1.9 | -1.8 | -2.50 | FALSE |
| SRX2460059 | TFs and others | PGR | Breast | Breast cancer cells | 335 | 34/31605 | -0.29/0.26 | -1.9 | -1.8 | -2.50 | FALSE |
| SRX5493753 | TFs and others | AR | Breast | Breast cancer cells | 637 | 44/31605 | -0.23/0.26 | -1.9 | -1.8 | -2.49 | FALSE |
| SRX5323815 | TFs and others | STAT3 | Breast | T-47D | 954 | 10/31605 | -0.75/0.26 | -1.9 | -1.8 | -2.49 | FALSE |
| SRX7879086 | TFs and others | Epitope tags | Breast | MDA-MB-231 | 1877 | 28/31605 | -0.35/0.26 | -1.9 | -1.8 | -2.49 | FALSE |
| SRX7101363 | TFs and others | TEAD1 | Breast | Breast cancer cells | 961 | 15/31605 | -0.57/0.26 | -1.9 | -1.8 | -2.48 | FALSE |
| SRX4017887 | TFs and others | TP63 | Breast | MCF 10A | 3748 | 220/31605 | 0.04/0.26 | -1.9 | -1.8 | -2.48 | FALSE |
| SRX3285558 | TFs and others | ESR1 | Breast | MCF-7 | 2617 | 25/31605 | -0.38/0.26 | -1.9 | -1.8 | -2.48 | FALSE |
| SRX5057614 | TFs and others | GATA3 | Breast | T-47D | 658 | 20/31605 | -0.45/0.26 | -1.9 | -1.8 | -2.48 | FALSE |
| SRX1012701 | TFs and others | PGR | Breast | MCF-7 | 607 | 28/31605 | -0.34/0.26 | -1.9 | -1.8 | -2.47 | FALSE |
| SRX7030878 | TFs and others | CBX5 | Breast | MCF-7 | 2364 | 36/31605 | -0.27/0.26 | -1.9 | -1.8 | -2.47 | FALSE |
| SRX5457173 | TFs and others | ZNF512B | Breast | MCF-7 | 1700 | 69/31605 | -0.12/0.26 | -1.9 | -1.8 | -2.46 | FALSE |
| SRX3862461 | TFs and others | GATA3 | Breast | T-47D | 696 | 27/31605 | -0.35/0.26 | -1.9 | -1.8 | -2.46 | FALSE |
| SRX2453441 | TFs and others | NLRP2 | Breast | MCF 10A | 670 | 31/31605 | -0.31/0.26 | -1.9 | -1.8 | -2.46 | FALSE |
| SRX5057611 | TFs and others | GATA3 | Breast | T-47D | 566 | 18/31605 | -0.49/0.26 | -1.9 | -1.8 | -2.46 | FALSE |
| SRX2662143 | TFs and others | SMARCE1 | Breast | HMLE | 854 | 6/31605 | -1.03/0.26 | -1.8 | -1.8 | -2.45 | FALSE |
| SRX6896410 | TFs and others | RNF2 | Breast | T-47D | 628 | 11/31605 | -0.69/0.26 | -1.8 | -1.8 | -2.45 | FALSE |
| SRX730443 | TFs and others | EP300 | Breast | MCF-7 | 667 | 50/31605 | -0.19/0.26 | -1.8 | -1.8 | -2.45 | FALSE |
| SRX4192882 | TFs and others | JUNB | Breast | MCF 10A | 45335 | 2864/31605 | 0.20/0.26 | -1.8 | -1.8 | -2.45 | FALSE |
| SRX18895266 | TFs and others | MECP2 | Breast | MCF-7 | 1311 | 14/31605 | -0.58/0.26 | -1.8 | -1.8 | -2.44 | FALSE |
| SRX3229131 | TFs and others | FOXA1 | Breast | Breast cancer | 1383 | 95/31605 | -0.07/0.26 | -1.8 | -1.8 | -2.44 | FALSE |
| SRX15232264 | TFs and others | DNA-RNA hybrid | Breast | MDA-MB-436 | 271 | 5/31605 | -1.14/0.26 | -1.8 | -1.8 | -2.44 | FALSE |
| SRX3070469 | TFs and others | NR3C1 | Breast | MCF 10A | 3206 | 364/31605 | 0.09/0.26 | -1.8 | -1.7 | -2.43 | FALSE |
| SRX3010034 | TFs and others | EZH2 | Breast | MCF-7 | 270 | 36/31605 | -0.26/0.26 | -1.8 | -1.7 | -2.42 | FALSE |
| SRX5457172 | TFs and others | ZNF512B | Breast | MCF-7 | 1773 | 95/31605 | -0.06/0.26 | -1.8 | -1.7 | -2.41 | FALSE |
| SRX5057624 | TFs and others | GATA3 | Breast | T-47D | 1818 | 98/31605 | -0.06/0.26 | -1.8 | -1.7 | -2.41 | FALSE |
| SRX2512920 | TFs and others | FOXA1 | Breast | MCF-7 | 2838 | 59/31605 | -0.15/0.26 | -1.8 | -1.7 | -2.41 | FALSE |
| SRX13327991 | TFs and others | CBX3 | Breast | MCF-7 | 1077 | 11/31605 | -0.67/0.26 | -1.8 | -1.7 | -2.41 | FALSE |
| SRX1044414 | TFs and others | RELA | Breast | MDA-MB-231 | 1166 | 34/31605 | -0.27/0.26 | -1.8 | -1.7 | -2.41 | FALSE |
| SRX1772414 | TFs and others | Epitope tags | Breast | MCF-7 | 949 | 31/31605 | -0.30/0.26 | -1.8 | -1.7 | -2.41 | FALSE |
| SRX8537750 | TFs and others | GATA3 | Breast | SUM 44PE | 1866 | 53/31605 | -0.17/0.26 | -1.8 | -1.7 | -2.40 | FALSE |
| SRX3087159 | TFs and others | TP53 | Breast | MCF-7 | 906 | 88/31605 | -0.07/0.26 | -1.8 | -1.7 | -2.40 | FALSE |
| SRX10664180 | TFs and others | SUZ12 | Breast | MCF-7 | 10447 | 1742/31605 | 0.18/0.26 | -1.8 | -1.7 | -2.40 | FALSE |
| SRX730455 | TFs and others | NCAPG | Breast | MCF-7 | 477 | 8/31605 | -0.83/0.26 | -1.8 | -1.7 | -2.39 | FALSE |
| SRX14960540 | TFs and others | EZH2 | Breast | MCF-7 | 281 | 27/31605 | 0.84/0.26 | -1.8 | -1.7 | 2.39 | TRUE |
| SRX15929726 | TFs and others | SMC1A | Breast | MCF 10A | 1539 | 228/31605 | 0.05/0.26 | -1.8 | -1.7 | -2.39 | FALSE |
| SRX3447353 | TFs and others | ESR1 | Breast | MCF-7 | 813 | 6/31605 | -0.99/0.26 | -1.8 | -1.7 | -2.39 | FALSE |
| SRX3070472 | TFs and others | NR3C1 | Breast | MCF 10A | 1163 | 142/31605 | -0.00/0.26 | -1.8 | -1.7 | -2.38 | FALSE |
| SRX9932081 | TFs and others | HMGN2 | Breast | MCF-7 | 1841 | 21/31605 | -0.41/0.26 | -1.8 | -1.7 | -2.38 | FALSE |
| SRX262139 | TFs and others | HSF1 | Breast | MCF-7 | 400 | 59/31605 | -0.14/0.26 | -1.8 | -1.7 | -2.38 | FALSE |
| SRX2940758 | TFs and others | TP53 | Breast | MCF-7 | 418 | 64/31605 | -0.13/0.26 | -1.8 | -1.7 | -2.38 | FALSE |
| SRX3011694 | TFs and others | JMJD6 | Breast | MCF-7 | 471 | 28/31605 | -0.32/0.26 | -1.8 | -1.7 | -2.38 | FALSE |
| SRX3321941 | TFs and others | CSDE1 | Breast | MCF-7 | 1875 | 13/31605 | -0.59/0.26 | -1.8 | -1.7 | -2.38 | FALSE |
| SRX4931256 | TFs and others | KMT2A | Breast | MCF-7 | 638 | 13/31605 | -0.59/0.26 | -1.8 | -1.7 | -2.37 | FALSE |
| SRX1758521 | TFs and others | Epitope tags | Breast | MCF-7 | 1006 | 306/31605 | 0.08/0.26 | -1.7 | -1.7 | -2.37 | FALSE |
| SRX15235718 | TFs and others | PRRX1 | Breast | MDA-MB-157 | 949 | 47/31605 | -0.19/0.26 | -1.7 | -1.7 | -2.37 | FALSE |
| SRX2662142 | TFs and others | SMARCC1 | Breast | HMLE | 477 | 4/31605 | -1.26/0.26 | -1.7 | -1.7 | -2.37 | FALSE |
| SRX16940295 | TFs and others | MAF | Breast | MCF-7 | 763 | 26/31605 | -0.34/0.26 | -1.7 | -1.7 | -2.37 | FALSE |
| SRX2662145 | TFs and others | SMARCC1 | Breast | HMLE | 783 | 6/31605 | -0.98/0.26 | -1.7 | -1.7 | -2.36 | FALSE |
| SRX8023126 | TFs and others | TP53 | Breast | MCF 10A | 1011 | 105/31605 | -0.04/0.26 | -1.7 | -1.7 | -2.36 | FALSE |
| SRX7644282 | TFs and others | CPSF3L | Breast | MCF-7 | 666 | 4/31605 | -1.25/0.26 | -1.7 | -1.7 | -2.35 | FALSE |
| SRX14255719 | TFs and others | YAP1 | Breast | MCF-7 | 157 | 13/31605 | -0.58/0.26 | -1.7 | -1.7 | -2.35 | FALSE |
| SRX15232273 | TFs and others | DNA-RNA hybrid | Breast | MDA-MB-436 | 519 | 7/31605 | -0.88/0.26 | -1.7 | -1.6 | -2.35 | FALSE |
| SRX10562203 | TFs and others | CARM1 | Breast | MDA-MB-231 | 1279 | 136/31605 | -0.00/0.26 | -1.7 | -1.6 | -2.34 | FALSE |
| SRX155748 | TFs and others | HSF1 | Breast | MCF-7 | 278 | 18/31605 | -0.45/0.26 | -1.7 | -1.6 | -2.34 | FALSE |
| SRX3080239 | TFs and others | BRD4 | Breast | MDA-MB-231 | 1331 | 23/31605 | -0.37/0.26 | -1.7 | -1.6 | -2.34 | FALSE |
| SRX5432860 | TFs and others | ARNTL | Breast | MDA-MB-231 | 823 | 39/31605 | -0.22/0.26 | -1.7 | -1.6 | -2.34 | FALSE |
| SRX10119394 | TFs and others | STAT3 | Breast | MDA-MB-231 | 736 | 152/31605 | 0.01/0.26 | -1.7 | -1.6 | -2.34 | FALSE |
| SRX673734 | TFs and others | PRKDC | Breast | MCF-7 | 707 | 22/31605 | -0.38/0.26 | -1.7 | -1.6 | -2.34 | FALSE |
| SRX4096872 | TFs and others | GATAD2B | Breast | HCC3153 | 1034 | 13/31605 | -0.57/0.26 | -1.7 | -1.6 | -2.33 | FALSE |
| SRX1757994 | TFs and others | FOXR2 | Breast | MDA-MB-468 | 710 | 11/31605 | -0.65/0.26 | -1.7 | -1.6 | -2.33 | FALSE |
| SRX2453443 | TFs and others | RAD51 | Breast | MCF 10A | 1240 | 11/31605 | -0.64/0.26 | -1.7 | -1.6 | -2.33 | FALSE |
| SRX10335184 | TFs and others | Epitope tags | Breast | MDA-MB-231 | 1303 | 96/31605 | -0.05/0.26 | -1.7 | -1.6 | -2.33 | FALSE |
| SRX2578920 | TFs and others | EP300 | Breast | MDA-MB-231 | 975 | 19/31605 | -0.43/0.26 | -1.7 | -1.6 | -2.32 | FALSE |
| SRX6058764 | TFs and others | RAD21 | Breast | T-47D | 2573 | 98/31605 | -0.05/0.26 | -1.7 | -1.6 | -2.32 | FALSE |
| SRX1728403 | TFs and others | TP53 | Breast | MCF 10A | 809 | 12/31605 | -0.60/0.26 | -1.7 | -1.6 | -2.32 | FALSE |
| SRX155736 | TFs and others | HSF1 | Breast | ZR-75-1 | 596 | 38/31605 | -0.22/0.26 | -1.7 | -1.6 | -2.31 | FALSE |
| SRX1143685 | TFs and others | TP63 | Breast | MDA-MB-231 | 741 | 12/31605 | -0.60/0.26 | -1.7 | -1.6 | -2.31 | FALSE |
| SRX21286686 | TFs and others | TAZ | Breast | MCF 10A | 6788 | 590/31605 | 0.13/0.26 | -1.7 | -1.6 | -2.31 | FALSE |
| SRX3092925 | TFs and others | EP300 | Breast | MDA-MB-231 | 763 | 18/31605 | -0.44/0.26 | -1.7 | -1.6 | -2.31 | FALSE |
| SRX155770 | TFs and others | HSF1 | Breast | Breast cancer cells | 975 | 225/31605 | 0.06/0.26 | -1.7 | -1.6 | -2.30 | FALSE |
| SRX3589925 | TFs and others | STAG1 | Breast | MCF 10A | 6072 | 856/31605 | 0.15/0.26 | -1.7 | -1.6 | -2.30 | FALSE |
| SRX10164577 | TFs and others | PAD2 | Breast | MCF-7 | 1348 | 7/31605 | -0.86/0.26 | -1.7 | -1.6 | -2.30 | FALSE |
| SRX10164601 | TFs and others | ESR1 | Breast | MCF-7 | 1481 | 7/31605 | -0.86/0.26 | -1.7 | -1.6 | -2.30 | FALSE |
| SRX10164614 | TFs and others | PAD2 | Breast | MCF-7 | 1091 | 7/31605 | -0.86/0.26 | -1.7 | -1.6 | -2.30 | FALSE |
| SRX10164626 | TFs and others | PAD2 | Breast | MCF-7 | 1346 | 7/31605 | -0.86/0.26 | -1.7 | -1.6 | -2.30 | FALSE |
| SRX9932078 | TFs and others | HMGN2 | Breast | MCF-7 | 665 | 7/31605 | -0.86/0.26 | -1.7 | -1.6 | -2.30 | FALSE |
| SRX3229188 | TFs and others | NR3C1 | Breast | Breast cancer | 678 | 5/31605 | -1.06/0.26 | -1.7 | -1.6 | -2.30 | FALSE |
| SRX1709985 | TFs and others | PGR | Breast | T-47D | 802 | 7/31605 | -0.86/0.26 | -1.7 | -1.6 | -2.30 | FALSE |
| SRX5513519 | TFs and others | EZH2 | Breast | MDA-MB-231 | 73 | 7/31605 | -0.86/0.26 | -1.7 | -1.6 | -2.30 | FALSE |
| SRX10306465 | TFs and others | KDM1A | Breast | T-47D | 2193 | 402/31605 | 0.11/0.26 | -1.7 | -1.6 | -2.30 | FALSE |
| SRX5172069 | TFs and others | ESR1 | Breast | MCF-7 | 632 | 8/31605 | -0.79/0.26 | -1.7 | -1.6 | -2.30 | FALSE |
| SRX5172072 | TFs and others | ESR1 | Breast | MCF-7 | 627 | 8/31605 | -0.79/0.26 | -1.7 | -1.6 | -2.30 | FALSE |
| SRX10164637 | TFs and others | ESR1 | Breast | MCF-7 | 1672 | 9/31605 | -0.72/0.26 | -1.7 | -1.6 | -2.29 | FALSE |
| SRX5323893 | TFs and others | STAT3 | Breast | T-47D | 730 | 11/31605 | -0.63/0.26 | -1.7 | -1.6 | -2.29 | FALSE |
| SRX17657148 | TFs and others | FOXC1 | Breast | BT-549 | 778 | 26/31605 | -0.32/0.26 | -1.7 | -1.6 | -2.29 | FALSE |
| SRX968974 | TFs and others | RELA | Breast | MCF-7 | 209 | 17/31605 | -0.45/0.26 | -1.7 | -1.6 | -2.29 | FALSE |
| SRX6058766 | TFs and others | RAD21 | Breast | T-47D | 3327 | 146/31605 | 0.01/0.26 | -1.7 | -1.6 | -2.28 | FALSE |
| SRX22289059 | TFs and others | LMNB1 | Breast | MCF 10A | 897 | 5/31605 | -1.05/0.26 | -1.6 | -1.6 | -2.28 | FALSE |
| SRX1772420 | TFs and others | NEDD4 | Breast | MCF-7 | 1259 | 13/31605 | -0.55/0.26 | -1.6 | -1.6 | -2.28 | FALSE |
| SRX3322528 | TFs and others | MNT | Breast | MCF-7 | 1421 | 6/31605 | -0.94/0.26 | -1.6 | -1.6 | -2.28 | FALSE |
| SRX12193399 | TFs and others | ESR1 | Breast | MCF-7 | 285 | 4/31605 | -1.20/0.26 | -1.6 | -1.6 | -2.28 | FALSE |
| SRX1161193 | TFs and others | FOXA1 | Breast | ZR-75-1 | 858 | 7/31605 | -0.85/0.26 | -1.6 | -1.6 | -2.28 | FALSE |
| SRX10119383 | TFs and others | JUNB | Breast | MCF 10A | 37209 | 2692/31605 | 0.20/0.26 | -1.6 | -1.6 | -2.27 | FALSE |
| SRX026903 | TFs and others | ESR1 | Breast | MCF-7 | 115 | 4/31605 | -1.20/0.26 | -1.6 | -1.6 | -2.27 | FALSE |
| SRX10365523 | TFs and others | RAC1 | Breast | MDA-MB-231 | 852 | 6/31605 | -0.93/0.26 | -1.6 | -1.6 | -2.27 | FALSE |
| SRX2513056 | TFs and others | MEN1 | Breast | T-47D | 2881 | 12/31605 | -0.58/0.26 | -1.6 | -1.6 | -2.27 | FALSE |
| SRX7101364 | TFs and others | TEAD1 | Breast | Breast cancer cells | 709 | 8/31605 | -0.77/0.26 | -1.6 | -1.6 | -2.26 | FALSE |
| SRX5089783 | TFs and others | SATB1 | Breast | MCF 10A | 26254 | 841/31605 | 0.16/0.26 | -1.6 | -1.5 | -2.26 | FALSE |
| SRX6058763 | TFs and others | RAD21 | Breast | T-47D | 3387 | 81/31605 | -0.07/0.26 | -1.6 | -1.5 | -2.26 | FALSE |
| SRX11650843 | TFs and others | SMARCA4 | Breast | CAL-120 | 1377 | 27/31605 | -0.30/0.26 | -1.6 | -1.5 | -2.26 | FALSE |
| SRX5089502 | TFs and others | HDAC1 | Breast | MCF-7 | 944 | 35/31605 | -0.23/0.26 | -1.6 | -1.5 | -2.25 | FALSE |
| SRX18191674 | TFs and others | ESR1 | Breast | MCF-7 | 978 | 9/31605 | -0.71/0.26 | -1.6 | -1.5 | -2.25 | FALSE |
| SRX4192709 | TFs and others | Epitope tags | Breast | MDA-MB-231 | 6780 | 4118/31605 | 0.21/0.26 | -1.6 | -1.5 | -2.25 | FALSE |
| SRX4561220 | TFs and others | ANAPC1 | Breast | MCF-7 | 2065 | 54/31605 | -0.14/0.26 | -1.6 | -1.5 | -2.25 | FALSE |
| SRX5323897 | TFs and others | STAT3 | Breast | T-47D | 905 | 33/31605 | -0.25/0.26 | -1.6 | -1.5 | -2.24 | FALSE |
| SRX5457386 | TFs and others | ZNF8 | Breast | MCF-7 | 1428 | 26/31605 | -0.31/0.26 | -1.6 | -1.5 | -2.24 | FALSE |
| SRX8023122 | TFs and others | TP53 | Breast | MCF 10A | 1817 | 92/31605 | -0.04/0.26 | -1.6 | -1.5 | -2.24 | FALSE |
| SRX9804374 | TFs and others | DEK | Breast | MCF-7 | 503 | 10/31605 | -0.65/0.26 | -1.6 | -1.5 | -2.24 | FALSE |
| SRX285808 | TFs and others | TP53 | Breast | MCF-7 | 696 | 101/31605 | -0.03/0.26 | -1.6 | -1.5 | -2.24 | FALSE |
| SRX9322282 | TFs and others | ESR1 | Breast | MCF-7 | 983 | 49/31605 | -0.15/0.26 | -1.6 | -1.5 | -2.24 | FALSE |
| SRX13840520 | TFs and others | 8-Hydroxydeoxyguanosine | Breast | MCF 10A | 24754 | 2929/31605 | 0.20/0.26 | -1.6 | -1.5 | -2.23 | FALSE |
| SRX2915391 | TFs and others | PGR | Breast | T-47D | 119 | 10/31605 | -0.65/0.26 | -1.6 | -1.5 | -2.23 | FALSE |
| SRX5323857 | TFs and others | STAT3 | Breast | MCF-7 | 303 | 27/31605 | -0.29/0.26 | -1.6 | -1.5 | -2.23 | FALSE |
| SRX10205612 | TFs and others | SMARCA4 | Breast | CAL-120 | 1038 | 10/31605 | -0.65/0.26 | -1.6 | -1.5 | -2.23 | FALSE |
| SRX1671744 | TFs and others | ERBB2 | Breast | SK-BR-3 | 882 | 34/31605 | -0.23/0.26 | -1.6 | -1.5 | -2.23 | FALSE |
| SRX15039979 | TFs and others | Epitope tags | Breast | MDA-MB-231 | 2276 | 181/31605 | 0.04/0.26 | -1.6 | -1.5 | -2.22 | FALSE |
| SRX5323837 | TFs and others | STAT3 | Breast | T-47D | 554 | 8/31605 | -0.75/0.26 | -1.6 | -1.5 | -2.22 | FALSE |
| SRX7522859 | TFs and others | BCL9 | Breast | MCF10DCIS.com | 1681 | 189/31605 | 0.05/0.26 | -1.6 | -1.5 | -2.22 | FALSE |
| SRX13845688 | TFs and others | HSF1 | Breast | MDA-MB-231 | 260 | 101/31605 | -0.03/0.26 | -1.6 | -1.5 | -2.21 | FALSE |
| SRX262134 | TFs and others | HSF1 | Breast | MCF-7 | 232 | 51/31605 | -0.14/0.26 | -1.6 | -1.5 | -2.21 | FALSE |
| SRX2915390 | TFs and others | PGR | Breast | T-47D | 103 | 33/31605 | -0.24/0.26 | -1.6 | -1.5 | -2.21 | FALSE |
| SRX15232263 | TFs and others | DNA-RNA hybrid | Breast | MDA-MB-436 | 207 | 4/31605 | -1.16/0.26 | -1.6 | -1.5 | -2.21 | FALSE |
| SRX9534929 | TFs and others | NR4A1 | Breast | Breast cancer cells | 288 | 14/31605 | -0.50/0.26 | -1.6 | -1.5 | -2.20 | FALSE |
| SRX423002 | TFs and others | CDK2 | Breast | T-47D | 530 | 20/31605 | -0.38/0.26 | -1.6 | -1.5 | -2.20 | FALSE |
| SRX1012700 | TFs and others | PGR | Breast | MCF-7 | 594 | 15/31605 | -0.47/0.26 | -1.6 | -1.5 | -2.20 | FALSE |
| SRX3080248 | TFs and others | BRD4 | Breast | MDA-MB-231 | 1391 | 9/31605 | -0.68/0.26 | -1.6 | -1.5 | -2.20 | FALSE |
| SRX13327976 | TFs and others | CBX3 | Breast | MCF-7 | 1135 | 8/31605 | -0.74/0.26 | -1.6 | -1.5 | -2.20 | FALSE |
| SRX4017884 | TFs and others | TP63 | Breast | Breast organoids | 1771 | 15/31605 | -0.47/0.26 | -1.5 | -1.5 | -2.19 | FALSE |
| SRX12008359 | TFs and others | CTCF | Breast | MCF 10A | 1040 | 14/31605 | -0.49/0.26 | -1.5 | -1.5 | -2.18 | FALSE |
| SRX8886223 | TFs and others | NFKB1 | Breast | MDA-MB-231 | 398 | 6/31605 | -0.89/0.26 | -1.5 | -1.5 | -2.18 | FALSE |
| SRX10421405 | TFs and others | ARID1A | Breast | MCF 10A | 510 | 2/31605 | -1.72/0.26 | -1.5 | -1.5 | -2.18 | FALSE |
| SRX14284263 | TFs and others | ARID1A | Breast | HMEC | 552 | 2/31605 | -1.72/0.26 | -1.5 | -1.5 | -2.18 | FALSE |
| SRX19495893 | TFs and others | PML | Breast | MDA-MB-231 | 582 | 2/31605 | -1.72/0.26 | -1.5 | -1.5 | -2.18 | FALSE |
| SRX2915393 | TFs and others | PGR | Breast | T-47D | 203 | 2/31605 | -1.72/0.26 | -1.5 | -1.5 | -2.18 | FALSE |
| SRX4116033 | TFs and others | ESR1 | Breast | Breast cancer cells | 186 | 2/31605 | -1.72/0.26 | -1.5 | -1.5 | -2.18 | FALSE |
| SRX4116035 | TFs and others | FOXA1 | Breast | Breast cancer cells | 200 | 2/31605 | -1.72/0.26 | -1.5 | -1.5 | -2.18 | FALSE |
| SRX4116050 | TFs and others | ESR1 | Breast | Breast cancer cells | 299 | 2/31605 | -1.72/0.26 | -1.5 | -1.5 | -2.18 | FALSE |
| SRX4116052 | TFs and others | FOXA1 | Breast | Breast cancer cells | 256 | 2/31605 | -1.72/0.26 | -1.5 | -1.5 | -2.18 | FALSE |
| SRX4561230 | TFs and others | Biotin | Breast | MCF-7 | 375 | 2/31605 | -1.72/0.26 | -1.5 | -1.5 | -2.18 | FALSE |
| SRX4561231 | TFs and others | Biotin | Breast | MCF-7 | 413 | 2/31605 | -1.72/0.26 | -1.5 | -1.5 | -2.18 | FALSE |
| SRX7657041 | TFs and others | NCOA3 | Breast | MCF10DCIS.com | 514 | 2/31605 | -1.72/0.26 | -1.5 | -1.5 | -2.18 | FALSE |
| SRX8886222 | TFs and others | NFKB1 | Breast | MDA-MB-231 | 339 | 2/31605 | -1.72/0.26 | -1.5 | -1.5 | -2.18 | FALSE |
| SRX9528952 | TFs and others | Epitope tags | Breast | MCF-7 | 676 | 2/31605 | -1.72/0.26 | -1.5 | -1.5 | -2.18 | FALSE |
| SRX3080244 | TFs and others | BRD4 | Breast | MDA-MB-231 | 1512 | 10/31605 | -0.63/0.26 | -1.5 | -1.5 | -2.18 | FALSE |
| SRX6831820 | TFs and others | NCOA3 | Breast | MCF 10A | 1784 | 33/31605 | -0.23/0.26 | -1.5 | -1.5 | -2.18 | FALSE |
| SRX262138 | TFs and others | HSF1 | Breast | MCF-7 | 362 | 32/31605 | -0.24/0.26 | -1.5 | -1.5 | -2.18 | FALSE |
| SRX1012716 | TFs and others | PGR | Breast | T-47D | 1649 | 10/31605 | -0.62/0.26 | -1.5 | -1.5 | -2.17 | FALSE |
| SRX3322409 | TFs and others | PPP1R10 | Breast | MCF-7 | 2231 | 62/31605 | -0.10/0.26 | -1.5 | -1.5 | -2.16 | FALSE |
| SRX182683 | TFs and others | PALB2 | Breast | MCF 10A | 384 | 84/31605 | -0.05/0.26 | -1.5 | -1.4 | -2.16 | FALSE |
| SRX1758522 | TFs and others | Epitope tags | Breast | MCF-7 | 826 | 100/31605 | 0.53/0.26 | -1.5 | -1.4 | 2.15 | TRUE |
| SRX5323831 | TFs and others | STAT3 | Breast | T-47D | 702 | 7/31605 | -0.79/0.26 | -1.5 | -1.4 | -2.15 | FALSE |
| SRX19580919 | TFs and others | NFE2L2 | Breast | MDA-MB-231 | 1847 | 30/31605 | -0.25/0.26 | -1.5 | -1.4 | -2.15 | FALSE |
| SRX10306462 | TFs and others | KDM1A | Breast | T-47D | 1359 | 66/31605 | -0.08/0.26 | -1.5 | -1.4 | -2.15 | FALSE |
| SRX5513515 | TFs and others | MTA1 | Breast | MDA-MB-231 | 98 | 7/31605 | -0.78/0.26 | -1.5 | -1.4 | -2.15 | FALSE |
| SRX1012616 | TFs and others | PGR | Breast | T-47D | 878 | 7/31605 | -0.78/0.26 | -1.5 | -1.4 | -2.15 | FALSE |
| SRX13845693 | TFs and others | HSF1 | Breast | MDA-MB-231 | 384 | 76/31605 | -0.06/0.26 | -1.5 | -1.4 | -2.14 | FALSE |
| SRX17682347 | TFs and others | RELA | Breast | MCF-7 | 2158 | 253/31605 | 0.08/0.26 | -1.5 | -1.4 | -2.14 | FALSE |
| SRX5493747 | TFs and others | AR | Breast | Breast cancer cells | 675 | 46/31605 | -0.15/0.26 | -1.5 | -1.4 | -2.14 | FALSE |
| SRX3322822 | TFs and others | PAX8 | Breast | MCF-7 | 2594 | 95/31605 | -0.03/0.26 | -1.5 | -1.4 | -2.14 | FALSE |
| SRX8023127 | TFs and others | TP53 | Breast | MCF 10A | 3537 | 152/31605 | 0.03/0.26 | -1.5 | -1.4 | -2.14 | FALSE |
| SRX10024469 | TFs and others | ESR1 | Breast | MCF-7 | 946 | 6/31605 | -0.86/0.26 | -1.5 | -1.4 | -2.14 | FALSE |
| SRX4096871 | TFs and others | GATAD2B | Breast | HCC3153 | 1259 | 15/31605 | -0.45/0.26 | -1.5 | -1.4 | -2.14 | FALSE |
| SRX13254864 | TFs and others | SUZ12 | Breast | MCF-7 | 45589 | 3953/31605 | 0.21/0.26 | -1.5 | -1.4 | -2.14 | FALSE |
| SRX19580916 | TFs and others | NFE2L2 | Breast | MDA-MB-231 | 1499 | 23/31605 | -0.32/0.26 | -1.5 | -1.4 | -2.14 | FALSE |
| SRX1117698 | TFs and others | KDM5C | Breast | ZR-75-30 | 151 | 12/31605 | -0.53/0.26 | -1.5 | -1.4 | -2.14 | FALSE |
| SRX15929736 | TFs and others | STAG1 | Breast | MCF 10A | 886 | 17/31605 | -0.41/0.26 | -1.5 | -1.4 | -2.13 | FALSE |
| SRX8023120 | TFs and others | TP53 | Breast | MCF 10A | 667 | 19/31605 | -0.37/0.26 | -1.5 | -1.4 | -2.13 | FALSE |
| SRX7865930 | TFs and others | BRD4 | Breast | MDA-MB-231 | 1596 | 15/31605 | -0.45/0.26 | -1.5 | -1.4 | -2.13 | FALSE |
| SRX730433 | TFs and others | NCAPG | Breast | MCF-7 | 269 | 10/31605 | -0.61/0.26 | -1.5 | -1.4 | -2.13 | FALSE |
| SRX15039974 | TFs and others | Epitope tags | Breast | MDA-MB-231 | 3476 | 389/31605 | 0.12/0.26 | -1.5 | -1.4 | -2.13 | FALSE |
| SRX7030915 | TFs and others | CBX5 | Breast | MCF-7 | 2707 | 24/31605 | -0.30/0.26 | -1.5 | -1.4 | -2.13 | FALSE |
| SRX10664181 | TFs and others | SUZ12 | Breast | MCF-7 | 10507 | 1229/31605 | 0.18/0.26 | -1.5 | -1.4 | -2.12 | FALSE |
| SRX5824554 | TFs and others | BRD7 | Breast | SUM 159PT | 1004 | 22/31605 | -0.32/0.26 | -1.5 | -1.4 | -2.12 | FALSE |
| SRX9534932 | TFs and others | NR4A1 | Breast | Breast cancer cells | 426 | 34/31605 | -0.21/0.26 | -1.5 | -1.4 | -2.12 | FALSE |
| SRX5457448 | TFs and others | ESRRA | Breast | MCF-7 | 1272 | 23/31605 | -0.31/0.26 | -1.5 | -1.4 | -2.12 | FALSE |
| SRX6058767 | TFs and others | TOP2A | Breast | T-47D | 2951 | 13/31605 | -0.50/0.26 | -1.5 | -1.4 | -2.12 | FALSE |
| SRX8023132 | TFs and others | TP53 | Breast | MCF 10A | 314 | 21/31605 | -0.34/0.26 | -1.5 | -1.4 | -2.12 | FALSE |
| SRX13327984 | TFs and others | XRCC6 | Breast | MCF-7 | 608 | 10/31605 | -0.60/0.26 | -1.5 | -1.4 | -2.11 | FALSE |
| SRX8023139 | TFs and others | TP53 | Breast | MCF 10A | 3378 | 142/31605 | 0.03/0.26 | -1.5 | -1.4 | -2.11 | FALSE |
| SRX16646114 | TFs and others | HSF1 | Breast | MCF-7 | 700 | 64/31605 | -0.08/0.26 | -1.5 | -1.4 | -2.11 | FALSE |
| SRX7642860 | TFs and others | RBM39 | Breast | Mammary epithelial cells | 119 | 3/31605 | -1.30/0.26 | -1.5 | -1.4 | -2.11 | FALSE |
| SRX13845696 | TFs and others | HSF2 | Breast | MDA-MB-231 | 433 | 115/31605 | 0.00/0.26 | -1.5 | -1.4 | -2.10 | FALSE |
| SRX5089378 | TFs and others | ARID1A | Breast | MCF-7 | 1278 | 22/31605 | -0.32/0.26 | -1.4 | -1.4 | -2.10 | FALSE |
| SRX2915392 | TFs and others | PGR | Breast | T-47D | 566 | 5/31605 | -0.95/0.26 | -1.4 | -1.4 | -2.10 | FALSE |
| SRX6860691 | TFs and others | ESR1 | Breast | T-47D | 1145 | 7/31605 | -0.76/0.26 | -1.4 | -1.4 | -2.09 | FALSE |
| SRX6860696 | TFs and others | ESR1 | Breast | T-47D | 1114 | 7/31605 | -0.76/0.26 | -1.4 | -1.4 | -2.09 | FALSE |
| SRX758326 | TFs and others | MBD2 | Breast | HMLE | 3547 | 187/31605 | 0.06/0.26 | -1.4 | -1.4 | -2.09 | FALSE |
| SRX155727 | TFs and others | HSF1 | Breast | MCF 10A | 1023 | 28/31605 | -0.25/0.26 | -1.4 | -1.4 | -2.09 | FALSE |
| SRX734341 | TFs and others | CHD8 | Breast | T-47D | 44832 | 2323/31605 | 0.31/0.26 | -1.4 | -1.4 | 2.09 | TRUE |
| SRX11650838 | TFs and others | SMARCA4 | Breast | CAL-120 | 2073 | 64/31605 | -0.08/0.26 | -1.4 | -1.4 | -2.08 | FALSE |
| SRX2585783 | TFs and others | EZH2 | Breast | MDA-MB-231 | 1007 | 12/31605 | -0.51/0.26 | -1.4 | -1.4 | -2.08 | FALSE |
| SRX5185604 | TFs and others | MED12 | Breast | MCF-7 | 572 | 13/31605 | -0.48/0.26 | -1.4 | -1.4 | -2.08 | FALSE |
| SRX673740 | TFs and others | ESR1 | Breast | MCF-7 | 394 | 12/31605 | -0.51/0.26 | -1.4 | -1.4 | -2.08 | FALSE |
| SRX15039980 | TFs and others | Epitope tags | Breast | MDA-MB-231 | 3433 | 330/31605 | 0.11/0.26 | -1.4 | -1.4 | -2.08 | FALSE |
| SRX329001 | TFs and others | Epitope tags | Breast | T-47D | 2167 | 116/31605 | 0.01/0.26 | -1.4 | -1.4 | -2.07 | FALSE |
| SRX5251414 | TFs and others | ESR1 | Breast | MCF-7 | 647 | 6/31605 | -0.83/0.26 | -1.4 | -1.4 | -2.07 | FALSE |
| SRX100261 | TFs and others | CREBBP | Breast | H3396 | 7930 | 485/31605 | 0.13/0.26 | -1.4 | -1.4 | -2.07 | FALSE |
| SRX10164638 | TFs and others | PAD2 | Breast | MCF-7 | 1155 | 5/31605 | -0.93/0.26 | -1.4 | -1.4 | -2.07 | FALSE |
| SRX5089523 | TFs and others | SMARCA4 | Breast | MCF-7 | 801 | 11/31605 | -0.54/0.26 | -1.4 | -1.4 | -2.07 | FALSE |
| SRX15039975 | TFs and others | Epitope tags | Breast | MDA-MB-231 | 2208 | 183/31605 | 0.06/0.26 | -1.4 | -1.3 | -2.06 | FALSE |
| SRX10040035 | TFs and others | TRMT13 | Breast | MDA-MB-231 | 1961 | 20/31605 | -0.33/0.26 | -1.4 | -1.3 | -2.06 | FALSE |
| SRX18895259 | TFs and others | MECP2 | Breast | MCF-7 | 1345 | 13/31605 | -0.47/0.26 | -1.4 | -1.3 | -2.05 | FALSE |
| SRX2425863 | TFs and others | SMAD1 | Breast | MDA-MB-231 | 2702 | 171/31605 | 0.05/0.26 | -1.4 | -1.3 | -2.05 | FALSE |
| SRX495670 | TFs and others | NR3C1 | Breast | MDA-MB-231 | 2633 | 80/31605 | -0.04/0.26 | -1.4 | -1.3 | -2.05 | FALSE |
| SRX19767355 | TFs and others | BrdU | Breast | MCF-7 | 94 | 1/31605 | -2.37/0.26 | -1.4 | -1.3 | -2.05 | FALSE |
| SRX20830484 | TFs and others | YAP1 | Breast | T-47D | 128 | 1/31605 | -2.37/0.26 | -1.4 | -1.3 | -2.05 | FALSE |
| SRX2915388 | TFs and others | PGR | Breast | T-47D | 149 | 1/31605 | -2.37/0.26 | -1.4 | -1.3 | -2.05 | FALSE |
| SRX9534897 | TFs and others | NR4A1 | Breast | MCF 10A | 328 | 1/31605 | -2.37/0.26 | -1.4 | -1.3 | -2.05 | FALSE |
| SRX10164619 | TFs and others | ESR1 | Breast | MCF-7 | 1481 | 8/31605 | -0.67/0.26 | -1.4 | -1.3 | -2.05 | FALSE |
| SRX3080240 | TFs and others | BRD4 | Breast | MDA-MB-231 | 1267 | 12/31605 | -0.50/0.26 | -1.4 | -1.3 | -2.05 | FALSE |
| SRX15574111 | TFs and others | CUL4B | Breast | MCF-7 | 700 | 8/31605 | -0.67/0.26 | -1.4 | -1.3 | -2.04 | FALSE |
| SRX10239456 | TFs and others | YTHDC1 | Breast | MCF-7 | 177 | 5/31605 | -0.92/0.26 | -1.4 | -1.3 | -2.04 | FALSE |
| SRX5323819 | TFs and others | STAT3 | Breast | T-47D | 686 | 8/31605 | -0.67/0.26 | -1.4 | -1.3 | -2.04 | FALSE |
| SRX5323835 | TFs and others | STAT3 | Breast | T-47D | 633 | 8/31605 | -0.67/0.26 | -1.4 | -1.3 | -2.04 | FALSE |
| SRX10164632 | TFs and others | PAD2 | Breast | MCF-7 | 1028 | 5/31605 | -0.91/0.26 | -1.4 | -1.3 | -2.04 | FALSE |
| SRX4561224 | TFs and others | Biotin | Breast | MCF-7 | 629 | 5/31605 | -0.91/0.26 | -1.4 | -1.3 | -2.04 | FALSE |
| SRX4561226 | TFs and others | Biotin | Breast | MCF-7 | 668 | 5/31605 | -0.91/0.26 | -1.4 | -1.3 | -2.04 | FALSE |
| SRX9932080 | TFs and others | HMGN2 | Breast | MCF-7 | 506 | 5/31605 | -0.91/0.26 | -1.4 | -1.3 | -2.04 | FALSE |
| SRX730440 | TFs and others | HECTD1 | Breast | MCF-7 | 1232 | 10/31605 | -0.57/0.26 | -1.4 | -1.3 | -2.04 | FALSE |
| SRX4284795 | TFs and others | Epitope tags | Breast | T-47D | 745 | 7/31605 | -0.73/0.26 | -1.4 | -1.3 | -2.03 | FALSE |
| SRX495671 | TFs and others | NR3C1 | Breast | MDA-MB-231 | 2141 | 18/31605 | -0.36/0.26 | -1.4 | -1.3 | -2.03 | FALSE |
| SRX3080243 | TFs and others | BRD4 | Breast | MDA-MB-231 | 1057 | 9/31605 | -0.61/0.26 | -1.4 | -1.3 | -2.03 | FALSE |
| SRX10164644 | TFs and others | PAD2 | Breast | MCF-7 | 1465 | 10/31605 | -0.57/0.26 | -1.4 | -1.3 | -2.03 | FALSE |
| SRX3087158 | TFs and others | TP53 | Breast | MCF-7 | 690 | 87/31605 | -0.02/0.26 | -1.4 | -1.3 | -2.03 | FALSE |
| SRX14255718 | TFs and others | YAP1 | Breast | MCF-7 | 119 | 7/31605 | -0.72/0.26 | -1.4 | -1.3 | -2.02 | FALSE |
| SRX12019746 | TFs and others | EZH2 | Breast | MCF-7 | 1116 | 16/31605 | -0.39/0.26 | -1.4 | -1.3 | -2.02 | FALSE |
| SRX6895233 | TFs and others | 8-Hydroxydeoxyguanosine | Breast | MCF 10A | 31090 | 2198/31605 | 0.31/0.26 | -1.4 | -1.3 | 2.02 | TRUE |
| SRX5213648 | TFs and others | BRD4 | Breast | HCC1806 | 1077 | 16/31605 | -0.39/0.26 | -1.4 | -1.3 | -2.02 | FALSE |
| SRX10365522 | TFs and others | RAC1 | Breast | MDA-MB-231 | 1087 | 9/31605 | -0.61/0.26 | -1.4 | -1.3 | -2.02 | FALSE |
| SRX3229129 | TFs and others | FOXA1 | Breast | Breast cancer | 2421 | 93/31605 | -0.01/0.26 | -1.4 | -1.3 | -2.01 | FALSE |
| SRX3541117 | TFs and others | SUMO2 | Breast | MCF-7 | 946 | 24/31605 | -0.27/0.26 | -1.4 | -1.3 | -2.01 | FALSE |
| SRX18895263 | TFs and others | MECP2 | Breast | MCF-7 | 1398 | 12/31605 | -0.49/0.26 | -1.4 | -1.3 | -2.01 | FALSE |
| SRX673737 | TFs and others | ESR1 | Breast | MCF-7 | 468 | 7/31605 | -0.72/0.26 | -1.4 | -1.3 | -2.01 | FALSE |
| SRX2944447 | TFs and others | MYC | Breast | MCF 10A | 773 | 26/31605 | -0.25/0.26 | -1.4 | -1.3 | -2.01 | FALSE |
| SRX4815195 | TFs and others | TLE3 | Breast | SUM 159PT | 20442 | 1363/31605 | 0.19/0.26 | -1.3 | -1.3 | -2.01 | FALSE |
| SRX667406 | TFs and others | Epitope tags | Breast | MCF-7 | 199 | 27/31605 | -0.24/0.26 | -1.3 | -1.3 | -2.01 | FALSE |
| SRX8023138 | TFs and others | TP53 | Breast | MCF 10A | 757 | 20/31605 | -0.32/0.26 | -1.3 | -1.3 | -2.01 | FALSE |
| SRX5493751 | TFs and others | AR | Breast | Breast cancer cells | 386 | 20/31605 | -0.32/0.26 | -1.3 | -1.3 | -2.00 | FALSE |
| ERX371679 | TFs and others | BRD4 | Breast | Mammary epithelial cells | 277 | 11/31605 | -0.52/0.26 | -1.3 | -1.3 | -2.00 | FALSE |
| SRX19580918 | TFs and others | NFE2L2 | Breast | MDA-MB-231 | 1761 | 40/31605 | -0.15/0.26 | -1.3 | -1.3 | -2.00 | FALSE |
| SRX3322059 | TFs and others | COPS2 | Breast | MCF-7 | 2024 | 12/31605 | -0.48/0.26 | -1.3 | -1.3 | -2.00 | FALSE |
| SRX6831821 | TFs and others | NCOA3 | Breast | MCF 10A | 2009 | 43/31605 | -0.13/0.26 | -1.3 | -1.3 | -1.99 | FALSE |
| SRX20830482 | TFs and others | YAP1 | Breast | T-47D | 155 | 1/31605 | -2.30/0.26 | -1.3 | -1.3 | -1.99 | FALSE |
| SRX10164586 | TFs and others | ESR1 | Breast | MCF-7 | 1506 | 8/31605 | -0.65/0.26 | -1.3 | -1.3 | -1.99 | FALSE |
| SRX10164620 | TFs and others | PAD2 | Breast | MCF-7 | 1445 | 8/31605 | -0.65/0.26 | -1.3 | -1.3 | -1.99 | FALSE |
| SRX10164650 | TFs and others | PAD2 | Breast | MCF-7 | 1356 | 8/31605 | -0.65/0.26 | -1.3 | -1.3 | -1.99 | FALSE |
| SRX19495896 | TFs and others | LMNB1 | Breast | MDA-MB-231 | 972 | 8/31605 | -0.64/0.26 | -1.3 | -1.3 | -1.98 | FALSE |
| SRX9739058 | TFs and others | NKRF | Breast | MDA-MB-231 | 282 | 23/31605 | -0.27/0.26 | -1.3 | -1.3 | -1.98 | FALSE |
| SRX4116025 | TFs and others | ESR1 | Breast | Breast cancer cells | 381 | 3/31605 | -1.21/0.26 | -1.3 | -1.3 | -1.98 | FALSE |
| SRX054573 | TFs and others | Epitope tags | Breast | MCF-7 | 5928 | 619/31605 | 0.15/0.26 | -1.3 | -1.3 | -1.97 | FALSE |
| SRX3322780 | TFs and others | LARP7 | Breast | MCF-7 | 1378 | 9/31605 | -0.59/0.26 | -1.3 | -1.3 | -1.97 | FALSE |
| SRX877859 | TFs and others | ZNF165 | Breast | WHIM12 | 1098 | 23/31605 | -0.27/0.26 | -1.3 | -1.3 | -1.97 | FALSE |
| SRX423006 | TFs and others | PARP1 | Breast | T-47D | 2026 | 24/31605 | -0.26/0.26 | -1.3 | -1.3 | -1.96 | FALSE |
| SRX10306475 | TFs and others | KDM1A | Breast | T-47D | 1278 | 193/31605 | 0.07/0.26 | -1.3 | -1.2 | -1.96 | FALSE |
| SRX330071 | TFs and others | Epitope tags | Breast | MCF-7 | 280 | 7/31605 | -0.69/0.26 | -1.3 | -1.2 | -1.96 | FALSE |
| SRX495667 | TFs and others | NR3C1 | Breast | MDA-MB-231 | 2218 | 15/31605 | -0.39/0.26 | -1.3 | -1.2 | -1.96 | FALSE |
| SRX1758488 | TFs and others | Epitope tags | Breast | MCF-7 | 1086 | 141/31605 | 0.47/0.26 | -1.3 | -1.2 | 1.95 | TRUE |
| SRX3265284 | TFs and others | KDM1A | Breast | MCF-7 | 876 | 7/31605 | -0.69/0.26 | -1.3 | -1.2 | -1.95 | FALSE |
| SRX15279660 | TFs and others | ZMYND8 | Breast | MDA-MB-231 | 148 | 4/31605 | -1.00/0.26 | -1.3 | -1.2 | -1.95 | FALSE |
| SRX423003 | TFs and others | CDK2 | Breast | T-47D | 1124 | 23/31605 | -0.27/0.26 | -1.3 | -1.2 | -1.95 | FALSE |
| SRX1012617 | TFs and others | PGR | Breast | T-47D | 989 | 9/31605 | -0.58/0.26 | -1.3 | -1.2 | -1.95 | FALSE |
| SRX518253 | TFs and others | CBX5 | Breast | MCF-7 | 1230 | 45/31605 | -0.12/0.26 | -1.3 | -1.2 | -1.94 | FALSE |
| SRX4909434 | TFs and others | MTA2 | Breast | HCC3153 | 1166 | 47/31605 | -0.11/0.26 | -1.3 | -1.2 | -1.94 | FALSE |
| SRX12019750 | TFs and others | STAT2 | Breast | MCF-7 | 271 | 26/31605 | -0.23/0.26 | -1.3 | -1.2 | -1.94 | FALSE |
| SRX9534931 | TFs and others | NR4A1 | Breast | Breast cancer cells | 226 | 12/31605 | -0.46/0.26 | -1.3 | -1.2 | -1.93 | FALSE |
| SRX13845694 | TFs and others | HSF1 | Breast | MDA-MB-231 | 222 | 83/31605 | -0.02/0.26 | -1.3 | -1.2 | -1.93 | FALSE |
| SRX1012652 | TFs and others | PGR | Breast | MCF-7 | 373 | 15/31605 | -0.38/0.26 | -1.3 | -1.2 | -1.93 | FALSE |
| SRX10664185 | TFs and others | JARID2 | Breast | MCF-7 | 1281 | 68/31605 | -0.04/0.26 | -1.3 | -1.2 | -1.93 | FALSE |
| SRX9688339 | TFs and others | SP1 | Breast | ZR-75-1 | 1011 | 7/31605 | -0.68/0.26 | -1.3 | -1.2 | -1.93 | FALSE |
| SRX4671299 | TFs and others | RARA | Breast | NAMEC8 | 835 | 43/31605 | -0.12/0.26 | -1.3 | -1.2 | -1.93 | FALSE |
| SRX2745728 | TFs and others | ZNF516 | Breast | MCF-7 | 794 | 10/31605 | -0.52/0.26 | -1.3 | -1.2 | -1.92 | FALSE |
| SRX5089384 | TFs and others | ARID1A | Breast | MCF-7 | 1104 | 24/31605 | -0.25/0.26 | -1.3 | -1.2 | -1.92 | FALSE |
| SRX16500148 | TFs and others | TSC22D2 | Breast | MCF-7 | 2993 | 90/31605 | -0.00/0.26 | -1.3 | -1.2 | -1.92 | FALSE |
| SRX8023116 | TFs and others | TP53 | Breast | MCF 10A | 2200 | 221/31605 | 0.09/0.26 | -1.3 | -1.2 | -1.92 | FALSE |
| SRX673722 | TFs and others | GATA3 | Breast | MCF-7 | 1013 | 45/31605 | -0.11/0.26 | -1.3 | -1.2 | -1.92 | FALSE |
| SRX14612972 | TFs and others | FOXA1 | Breast | T-47D | 799 | 13/31605 | -0.42/0.26 | -1.3 | -1.2 | -1.91 | FALSE |
| SRX155715 | TFs and others | HSF1 | Breast | HMEC | 226 | 6/31605 | -0.74/0.26 | -1.2 | -1.2 | -1.91 | FALSE |
| SRX7097954 | TFs and others | FBXO44 | Breast | MDA-MB-231 | 545 | 4/31605 | -0.96/0.26 | -1.2 | -1.2 | -1.90 | FALSE |
| SRX4116027 | TFs and others | FOXA1 | Breast | Breast cancer cells | 381 | 3/31605 | -1.15/0.26 | -1.2 | -1.2 | -1.90 | FALSE |
| SRX5824556 | TFs and others | BRD7 | Breast | SUM 159PT | 1523 | 165/31605 | 0.07/0.26 | -1.2 | -1.2 | -1.90 | FALSE |
| SRX8918908 | TFs and others | Epitope tags | Breast | MDA-MB-231 | 998 | 4/31605 | -0.96/0.26 | -1.2 | -1.2 | -1.90 | FALSE |
| SRX5513513 | TFs and others | PHF20L1 | Breast | MDA-MB-231 | 343 | 8/31605 | -0.60/0.26 | -1.2 | -1.2 | -1.90 | FALSE |
| SRX1131556 | TFs and others | BRD4 | Breast | SUM 1315M02 | 1982 | 36/31605 | -0.15/0.26 | -1.2 | -1.2 | -1.89 | FALSE |
| SRX8023115 | TFs and others | TP53 | Breast | MCF 10A | 2219 | 226/31605 | 0.09/0.26 | -1.2 | -1.2 | -1.89 | FALSE |
| SRX8023133 | TFs and others | TP53 | Breast | MCF 10A | 453 | 22/31605 | -0.26/0.26 | -1.2 | -1.2 | -1.88 | FALSE |
| SRX13327966 | TFs and others | CBX3 | Breast | MCF-7 | 610 | 6/31605 | -0.73/0.26 | -1.2 | -1.2 | -1.88 | FALSE |
| SRX11647486 | TFs and others | ESR1 | Breast | MCF-7 | 752 | 9/31605 | -0.55/0.26 | -1.2 | -1.2 | -1.88 | FALSE |
| SRX3070468 | TFs and others | NR3C1 | Breast | MCF 10A | 223 | 25/31605 | -0.22/0.26 | -1.2 | -1.2 | -1.87 | FALSE |
| SRX2424765 | TFs and others | ZNF592 | Breast | MCF-7 | 916 | 61/31605 | -0.05/0.26 | -1.2 | -1.2 | -1.87 | FALSE |
| SRX730434 | TFs and others | NCAPG | Breast | MCF-7 | 596 | 21/31605 | -0.27/0.26 | -1.2 | -1.2 | -1.87 | FALSE |
| SRX4561216 | TFs and others | ANAPC1 | Breast | MCF-7 | 2088 | 44/31605 | -0.11/0.26 | -1.2 | -1.2 | -1.87 | FALSE |
| SRX1100312 | TFs and others | RELA | Breast | HMEC | 1223 | 75/31605 | -0.02/0.26 | -1.2 | -1.2 | -1.87 | FALSE |
| SRX5457385 | TFs and others | ZNF8 | Breast | MCF-7 | 1365 | 37/31605 | -0.14/0.26 | -1.2 | -1.2 | -1.86 | FALSE |
| SRX143300 | TFs and others | SNAPC4 | Breast | MCF 10A | 1178 | 13/31605 | -0.41/0.26 | -1.2 | -1.2 | -1.86 | FALSE |
| SRX423004 | TFs and others | PARP1 | Breast | T-47D | 1662 | 17/31605 | -0.32/0.26 | -1.2 | -1.2 | -1.86 | FALSE |
| SRX7130718 | TFs and others | TP53 | Breast | MCF 10A | 1382 | 58/31605 | -0.06/0.26 | -1.2 | -1.1 | -1.86 | FALSE |
| SRX593750 | TFs and others | SFPQ | Breast | MCF-7 | 3304 | 99/31605 | 0.02/0.26 | -1.2 | -1.1 | -1.85 | FALSE |
| SRX396572 | TFs and others | RING1 | Breast | Breast cells | 801 | 167/31605 | 0.44/0.26 | -1.2 | -1.1 | 1.85 | TRUE |
| SRX11647483 | TFs and others | ESR1 | Breast | MCF-7 | 674 | 6/31605 | -0.71/0.26 | -1.2 | -1.1 | -1.84 | FALSE |
| SRX10641821 | TFs and others | TP63 | Breast | SUM 159PT | 4043 | 202/31605 | 0.09/0.26 | -1.2 | -1.1 | -1.84 | FALSE |
| SRX3265286 | TFs and others | KDM1A | Breast | MDA-MB-231 | 978 | 15/31605 | -0.35/0.26 | -1.2 | -1.1 | -1.84 | FALSE |
| SRX15235736 | TFs and others | PRRX1 | Breast | HDQ-P1 | 929 | 123/31605 | 0.04/0.26 | -1.2 | -1.1 | -1.84 | FALSE |
| SRX18895255 | TFs and others | Epitope tags | Breast | MCF-7 | 1718 | 15/31605 | -0.35/0.26 | -1.2 | -1.1 | -1.83 | FALSE |
| SRX14613014 | TFs and others | SMARCC1 | Breast | T-47D | 854 | 14/31605 | -0.37/0.26 | -1.2 | -1.1 | -1.83 | FALSE |
| SRX3862455 | TFs and others | GATA3 | Breast | T-47D | 551 | 20/31605 | -0.27/0.26 | -1.2 | -1.1 | -1.83 | FALSE |
| SRX1012698 | TFs and others | PGR | Breast | MCF-7 | 554 | 13/31605 | -0.39/0.26 | -1.2 | -1.1 | -1.82 | FALSE |
| SRX155730 | TFs and others | HSF1 | Breast | MCF 10A | 589 | 66/31605 | -0.03/0.26 | -1.2 | -1.1 | -1.82 | FALSE |
| SRX1757995 | TFs and others | FOXR2 | Breast | MDA-MB-468 | 423 | 8/31605 | -0.57/0.26 | -1.2 | -1.1 | -1.82 | FALSE |
| SRX2424125 | TFs and others | MTA2 | Breast | MCF-7 | 1781 | 21/31605 | -0.25/0.26 | -1.2 | -1.1 | -1.82 | FALSE |
| SRX1758487 | TFs and others | Epitope tags | Breast | MCF-7 | 641 | 67/31605 | 0.54/0.26 | -1.2 | -1.1 | 1.81 | TRUE |
| SRX10775079 | TFs and others | Epitope tags | Breast | HMLE | 870 | 16/31605 | -0.32/0.26 | -1.2 | -1.1 | -1.81 | FALSE |
| SRX6831822 | TFs and others | NCOA3 | Breast | MCF 10A | 1911 | 39/31605 | -0.12/0.26 | -1.2 | -1.1 | -1.81 | FALSE |
| SRX9697821 | TFs and others | Epitope tags | Breast | MDA-MB-231 | 713 | 9/31605 | -0.52/0.26 | -1.2 | -1.1 | -1.81 | FALSE |
| SRX15232271 | TFs and others | DNA-RNA hybrid | Breast | MDA-MB-436 | 297 | 6/31605 | -0.69/0.26 | -1.1 | -1.1 | -1.81 | FALSE |
| SRX17657159 | TFs and others | FOXC1 | Breast | MDA-MB-231 | 706 | 17/31605 | -0.31/0.26 | -1.1 | -1.1 | -1.81 | FALSE |
| SRX18191672 | TFs and others | ESR1 | Breast | MCF-7 | 884 | 7/31605 | -0.62/0.26 | -1.1 | -1.1 | -1.80 | FALSE |
| SRX18191673 | TFs and others | ESR1 | Breast | MCF-7 | 915 | 7/31605 | -0.62/0.26 | -1.1 | -1.1 | -1.80 | FALSE |
| SRX23330424 | TFs and others | HDAC6 | Breast | MDA-MB-231 | 831 | 10/31605 | -0.47/0.26 | -1.1 | -1.1 | -1.80 | FALSE |
| SRX155737 | TFs and others | HSF1 | Breast | ZR-75-1 | 810 | 52/31605 | -0.06/0.26 | -1.1 | -1.1 | -1.80 | FALSE |
| SRX13323811 | TFs and others | GFP | Breast | MDA-MB-231 | 1288 | 15/31605 | -0.34/0.26 | -1.1 | -1.1 | -1.79 | FALSE |
| SRX6058761 | TFs and others | PGR | Breast | T-47D | 1617 | 6/31605 | -0.68/0.26 | -1.1 | -1.1 | -1.79 | FALSE |
| SRX666560 | TFs and others | Epitope tags | Breast | T-47D | 3606 | 24/31605 | -0.21/0.26 | -1.1 | -1.1 | -1.79 | FALSE |
| ERX2242414 | TFs and others | ARID1A | Breast | Mammary epithelial cells | 515 | 3/31605 | -1.07/0.26 | -1.1 | -1.1 | -1.79 | FALSE |
| SRX10164572 | TFs and others | PAD2 | Breast | MCF-7 | 1030 | 3/31605 | -1.07/0.26 | -1.1 | -1.1 | -1.79 | FALSE |
| SRX10164582 | TFs and others | PAD2 | Breast | MCF-7 | 1149 | 3/31605 | -1.07/0.26 | -1.1 | -1.1 | -1.79 | FALSE |
| SRX10164597 | TFs and others | PAD2 | Breast | MCF-7 | 937 | 3/31605 | -1.07/0.26 | -1.1 | -1.1 | -1.79 | FALSE |
| SRX10164608 | TFs and others | PAD2 | Breast | MCF-7 | 961 | 3/31605 | -1.07/0.26 | -1.1 | -1.1 | -1.79 | FALSE |
| SRX10399318 | TFs and others | RBM39 | Breast | Breast epithelium | 930 | 3/31605 | -1.07/0.26 | -1.1 | -1.1 | -1.79 | FALSE |
| SRX10399319 | TFs and others | RBM39 | Breast | Breast epithelium | 794 | 3/31605 | -1.07/0.26 | -1.1 | -1.1 | -1.79 | FALSE |
| SRX10421402 | TFs and others | ARID1A | Breast | MCF 10A | 289 | 3/31605 | -1.07/0.26 | -1.1 | -1.1 | -1.79 | FALSE |
| SRX13327992 | TFs and others | CBX3 | Breast | MCF-7 | 738 | 3/31605 | -1.07/0.26 | -1.1 | -1.1 | -1.79 | FALSE |
| SRX13581735 | TFs and others | MED1 | Breast | HMLE | 794 | 3/31605 | -1.07/0.26 | -1.1 | -1.1 | -1.79 | FALSE |
| SRX14284267 | TFs and others | ARID1A | Breast | HMEC | 788 | 3/31605 | -1.07/0.26 | -1.1 | -1.1 | -1.79 | FALSE |
| SRX19495895 | TFs and others | LMNB1 | Breast | MDA-MB-231 | 538 | 3/31605 | -1.07/0.26 | -1.1 | -1.1 | -1.79 | FALSE |
| SRX19495897 | TFs and others | LMNB1 | Breast | MDA-MB-231 | 515 | 3/31605 | -1.07/0.26 | -1.1 | -1.1 | -1.79 | FALSE |
| SRX19495898 | TFs and others | LMNB1 | Breast | MDA-MB-231 | 995 | 3/31605 | -1.07/0.26 | -1.1 | -1.1 | -1.79 | FALSE |
| SRX19767364 | TFs and others | BrdU | Breast | T-47D | 178 | 3/31605 | -1.07/0.26 | -1.1 | -1.1 | -1.79 | FALSE |
| SRX2897221 | TFs and others | ESR1 | Breast | Breast cancer cells | 651 | 3/31605 | -1.07/0.26 | -1.1 | -1.1 | -1.79 | FALSE |
| SRX3092922 | TFs and others | EP300 | Breast | MDA-MB-231 | 904 | 3/31605 | -1.07/0.26 | -1.1 | -1.1 | -1.79 | FALSE |
| SRX3092926 | TFs and others | EP300 | Breast | MDA-MB-231 | 620 | 3/31605 | -1.07/0.26 | -1.1 | -1.1 | -1.79 | FALSE |
| SRX4561225 | TFs and others | Biotin | Breast | MCF-7 | 579 | 3/31605 | -1.07/0.26 | -1.1 | -1.1 | -1.79 | FALSE |
| SRX4561232 | TFs and others | Biotin | Breast | MCF-7 | 483 | 3/31605 | -1.07/0.26 | -1.1 | -1.1 | -1.79 | FALSE |
| SRX5089786 | TFs and others | SATB1 | Breast | MCF 10A | 1849 | 3/31605 | -1.07/0.26 | -1.1 | -1.1 | -1.79 | FALSE |
| SRX5493766 | TFs and others | ESR1 | Breast | Breast cancer cells | 405 | 3/31605 | -1.07/0.26 | -1.1 | -1.1 | -1.79 | FALSE |
| SRX7657039 | TFs and others | NCOA3 | Breast | MCF10DCIS.com | 605 | 3/31605 | -1.07/0.26 | -1.1 | -1.1 | -1.79 | FALSE |
| SRX7657040 | TFs and others | NCOA3 | Breast | MCF10DCIS.com | 571 | 3/31605 | -1.07/0.26 | -1.1 | -1.1 | -1.79 | FALSE |
| SRX7657042 | TFs and others | NCOA3 | Breast | MCF10DCIS.com | 541 | 3/31605 | -1.07/0.26 | -1.1 | -1.1 | -1.79 | FALSE |
| SRX7657043 | TFs and others | NCOA3 | Breast | MCF10DCIS.com | 434 | 3/31605 | -1.07/0.26 | -1.1 | -1.1 | -1.79 | FALSE |
| SRX7657044 | TFs and others | NCOA3 | Breast | MCF10DCIS.com | 421 | 3/31605 | -1.07/0.26 | -1.1 | -1.1 | -1.79 | FALSE |
| SRX7657048 | TFs and others | NCOA3 | Breast | MCF 10A | 964 | 3/31605 | -1.07/0.26 | -1.1 | -1.1 | -1.79 | FALSE |
| SRX7657049 | TFs and others | NCOA3 | Breast | MCF 10A | 1028 | 3/31605 | -1.07/0.26 | -1.1 | -1.1 | -1.79 | FALSE |
| SRX7657050 | TFs and others | NCOA3 | Breast | MCF 10A | 835 | 3/31605 | -1.07/0.26 | -1.1 | -1.1 | -1.79 | FALSE |
| SRX8353664 | TFs and others | LMNA | Breast | MDA-MB-231 | 413 | 3/31605 | -1.07/0.26 | -1.1 | -1.1 | -1.79 | FALSE |
| SRX8353665 | TFs and others | LMNA | Breast | MDA-MB-231 | 369 | 3/31605 | -1.07/0.26 | -1.1 | -1.1 | -1.79 | FALSE |
| SRX8353666 | TFs and others | LMNA | Breast | MDA-MB-231 | 530 | 3/31605 | -1.07/0.26 | -1.1 | -1.1 | -1.79 | FALSE |
| SRX8353669 | TFs and others | LMNA | Breast | MDA-MB-231 | 410 | 3/31605 | -1.07/0.26 | -1.1 | -1.1 | -1.79 | FALSE |
| SRX8537746 | TFs and others | GATA3 | Breast | MCF-7 | 670 | 3/31605 | -1.07/0.26 | -1.1 | -1.1 | -1.79 | FALSE |
| SRX9605789 | TFs and others | TP53 | Breast | MCF 10A | 471 | 3/31605 | -1.07/0.26 | -1.1 | -1.1 | -1.79 | FALSE |
| SRX3229184 | TFs and others | NR3C1 | Breast | Breast cancer | 545 | 4/31605 | -0.89/0.26 | -1.1 | -1.1 | -1.79 | FALSE |
| SRX155769 | TFs and others | HSF1 | Breast | Breast cancer cells | 454 | 103/31605 | 0.03/0.26 | -1.1 | -1.1 | -1.78 | FALSE |
| SRX1671741 | TFs and others | ERBB2 | Breast | BT-474 | 3112 | 167/31605 | 0.08/0.26 | -1.1 | -1.1 | -1.78 | FALSE |
| SRX5323889 | TFs and others | STAT3 | Breast | T-47D | 820 | 11/31605 | -0.43/0.26 | -1.1 | -1.1 | -1.78 | FALSE |
| SRX18014928 | TFs and others | SMAD3 | Breast | MCF 10A | 4908 | 514/31605 | 0.15/0.26 | -1.1 | -1.1 | -1.78 | FALSE |
| SRX10164587 | TFs and others | PAD2 | Breast | MCF-7 | 1714 | 9/31605 | -0.50/0.26 | -1.1 | -1.1 | -1.78 | FALSE |
| SRX10164602 | TFs and others | PAD2 | Breast | MCF-7 | 1421 | 9/31605 | -0.50/0.26 | -1.1 | -1.1 | -1.78 | FALSE |
| SRX10664182 | TFs and others | SUZ12 | Breast | MCF-7 | 3739 | 403/31605 | 0.37/0.26 | -1.1 | -1.1 | 1.77 | TRUE |
| SRX13327970 | TFs and others | XRCC6 | Breast | MCF-7 | 466 | 8/31605 | -0.54/0.26 | -1.1 | -1.1 | -1.77 | FALSE |
| SRX3862452 | TFs and others | GATA3 | Breast | T-47D | 760 | 24/31605 | -0.21/0.26 | -1.1 | -1.1 | -1.76 | FALSE |
| SRX673731 | TFs and others | PRKDC | Breast | MCF-7 | 876 | 15/31605 | -0.33/0.26 | -1.1 | -1.1 | -1.76 | FALSE |
| SRX1012762 | TFs and others | PGR | Breast | MCF-7 | 891 | 19/31605 | -0.26/0.26 | -1.1 | -1.1 | -1.76 | FALSE |
| SRX425996 | TFs and others | HOXC11 | Breast | LY2 | 156 | 10/31605 | -0.46/0.26 | -1.1 | -1.1 | -1.76 | FALSE |
| SRX8537749 | TFs and others | GATA3 | Breast | SUM 44PE | 770 | 27/31605 | -0.18/0.26 | -1.1 | -1.1 | -1.76 | FALSE |
| SRX155721 | TFs and others | HSF1 | Breast | BPE | 325 | 60/31605 | -0.04/0.26 | -1.1 | -1.1 | -1.76 | FALSE |
| SRX19662629 | TFs and others | KDM1A | Breast | T-47D | 614 | 25/31605 | -0.19/0.26 | -1.1 | -1.1 | -1.75 | FALSE |
| SRX9534930 | TFs and others | NR4A1 | Breast | Breast cancer cells | 480 | 11/31605 | -0.42/0.26 | -1.1 | -1.1 | -1.75 | FALSE |
| SRX14812503 | TFs and others | TP53 | Breast | Mammary epithelial cells | 1014 | 10/31605 | -0.45/0.26 | -1.1 | -1.1 | -1.75 | FALSE |
| SRX19580917 | TFs and others | NFE2L2 | Breast | MDA-MB-231 | 1326 | 19/31605 | -0.26/0.26 | -1.1 | -1.1 | -1.75 | FALSE |
| ERX2242375 | TFs and others | BCL11A | Breast | Breast cells | 621 | 4/31605 | -0.86/0.26 | -1.1 | -1.1 | -1.75 | FALSE |
| SRX822011 | TFs and others | KDM1A | Breast | T-47D | 1587 | 12/31605 | -0.39/0.26 | -1.1 | -1.0 | -1.74 | FALSE |
| SRX1161194 | TFs and others | NR3C1 | Breast | T-47D | 643 | 6/31605 | -0.65/0.26 | -1.1 | -1.0 | -1.74 | FALSE |
| SRX3554407 | TFs and others | CREB1 | Breast | MDA-MB-134-VI | 52 | 3/31605 | -1.03/0.26 | -1.1 | -1.0 | -1.74 | FALSE |
| SRX18014932 | TFs and others | JUNB | Breast | MCF 10A | 11419 | 936/31605 | 0.18/0.26 | -1.1 | -1.0 | -1.73 | FALSE |
| SRX3322058 | TFs and others | COPS2 | Breast | MCF-7 | 1495 | 8/31605 | -0.53/0.26 | -1.1 | -1.0 | -1.73 | FALSE |
| SRX3322112 | TFs and others | NFRKB | Breast | MCF-7 | 1447 | 8/31605 | -0.53/0.26 | -1.1 | -1.0 | -1.73 | FALSE |
| SRX4671317 | TFs and others | EP300 | Breast | SUM 159PT | 8954 | 833/31605 | 0.18/0.26 | -1.1 | -1.0 | -1.72 | FALSE |
| SRX3010035 | TFs and others | EZH2 | Breast | MCF-7 | 1344 | 53/31605 | -0.05/0.26 | -1.1 | -1.0 | -1.72 | FALSE |
| SRX6712595 | TFs and others | MED26 | Breast | MCF-7 | 680 | 7/31605 | -0.57/0.26 | -1.1 | -1.0 | -1.71 | FALSE |
| SRX23076223 | TFs and others | NFKB2 | Breast | MDA-MB-231 | 1056 | 101/31605 | 0.04/0.26 | -1.1 | -1.0 | -1.70 | FALSE |
| SRX10306471 | TFs and others | RCOR1 | Breast | T-47D | 1030 | 15/31605 | -0.31/0.26 | -1.1 | -1.0 | -1.70 | FALSE |
| SRX8023111 | TFs and others | TP53 | Breast | MCF 10A | 1115 | 34/31605 | -0.12/0.26 | -1.0 | -1.0 | -1.70 | FALSE |
| SRX495666 | TFs and others | NR3C1 | Breast | MDA-MB-231 | 2682 | 74/31605 | 0.00/0.26 | -1.0 | -1.0 | -1.70 | FALSE |
| SRX1100314 | TFs and others | RELA | Breast | HMEC | 851 | 20/31605 | -0.23/0.26 | -1.0 | -1.0 | -1.68 | FALSE |
| SRX1012761 | TFs and others | PGR | Breast | MCF-7 | 995 | 26/31605 | -0.17/0.26 | -1.0 | -1.0 | -1.68 | FALSE |
| SRX5401859 | TFs and others | NR3C1 | Breast | T-47D | 1758 | 33/31605 | -0.12/0.26 | -1.0 | -1.0 | -1.68 | FALSE |
| SRX10164655 | TFs and others | ESR1 | Breast | MCF-7 | 1387 | 6/31605 | -0.62/0.26 | -1.0 | -1.0 | -1.68 | FALSE |
| SRX13867212 | TFs and others | SOX9 | Breast | MCF-7 | 1090 | 6/31605 | -0.62/0.26 | -1.0 | -1.0 | -1.68 | FALSE |
| SRX5172071 | TFs and others | ESR1 | Breast | MCF-7 | 1344 | 37/31605 | -0.10/0.26 | -1.0 | -1.0 | -1.67 | FALSE |
| SRX3312986 | TFs and others | PADI2 | Breast | T-47D | 9666 | 298/31605 | 0.13/0.26 | -1.0 | -1.0 | -1.67 | FALSE |
| SRX3080209 | TFs and others | JUN | Breast | MCF-7 | 2641 | 175/31605 | 0.09/0.26 | -1.0 | -1.0 | -1.67 | FALSE |
| SRX3322347 | TFs and others | EP300 | Breast | Breast epithelium | 1342 | 29/31605 | -0.14/0.26 | -1.0 | -1.0 | -1.67 | FALSE |
| SRX3322113 | TFs and others | NFRKB | Breast | MCF-7 | 2195 | 10/31605 | -0.42/0.26 | -1.0 | -1.0 | -1.67 | FALSE |
| SRX425995 | TFs and others | HOXC11 | Breast | LY2 | 176 | 14/31605 | -0.31/0.26 | -1.0 | -1.0 | -1.67 | FALSE |
| ERX371709 | TFs and others | BRD4 | Breast | Mammary epithelial cells | 507 | 28/31605 | -0.15/0.26 | -1.0 | -1.0 | -1.67 | FALSE |
| SRX7657045 | TFs and others | NCOA3 | Breast | MCF 10A | 780 | 6/31605 | -0.61/0.26 | -1.0 | -1.0 | -1.66 | FALSE |
| SRX1757998 | TFs and others | MYC | Breast | MDA-MB-468 | 514 | 6/31605 | -0.61/0.26 | -1.0 | -1.0 | -1.66 | FALSE |
| SRX7030918 | TFs and others | TRIM28 | Breast | MCF-7 | 390 | 19/31605 | -0.23/0.26 | -1.0 | -1.0 | -1.66 | FALSE |
| SRX6895232 | TFs and others | 8-Hydroxydeoxyguanosine | Breast | MCF 10A | 32101 | 2472/31605 | 0.30/0.26 | -1.0 | -1.0 | 1.66 | TRUE |
| SRX2423593 | TFs and others | HDGF | Breast | MCF-7 | 1601 | 43/31605 | -0.07/0.26 | -1.0 | -1.0 | -1.65 | FALSE |
| SRX9605791 | TFs and others | TP53 | Breast | MCF 10A | 893 | 5/31605 | -0.69/0.26 | -1.0 | -1.0 | -1.65 | FALSE |
| SRX1709964 | TFs and others | ESR1 | Breast | T-47D | 105 | 11/31605 | -0.38/0.26 | -1.0 | -1.0 | -1.65 | FALSE |
| SRX18014929 | TFs and others | SMAD3 | Breast | MCF 10A | 4025 | 366/31605 | 0.14/0.26 | -1.0 | -1.0 | -1.65 | FALSE |
| SRX6058765 | TFs and others | RAD21 | Breast | T-47D | 3016 | 182/31605 | 0.10/0.26 | -1.0 | -1.0 | -1.65 | FALSE |
| SRX5057630 | TFs and others | GATA3 | Breast | T-47D | 773 | 22/31605 | -0.19/0.26 | -1.0 | -1.0 | -1.64 | FALSE |
| SRX5457449 | TFs and others | ESRRA | Breast | MCF-7 | 1315 | 16/31605 | -0.27/0.26 | -1.0 | -1.0 | -1.64 | FALSE |
| SRX3040154 | TFs and others | ZMYM2 | Breast | HMEC | 905 | 20/31605 | -0.21/0.26 | -1.0 | -1.0 | -1.64 | FALSE |
| SRX1671742 | TFs and others | ERBB2 | Breast | BT-474 | 13385 | 845/31605 | 0.18/0.26 | -1.0 | -1.0 | -1.63 | FALSE |
| SRX8023121 | TFs and others | TP53 | Breast | MCF 10A | 397 | 33/31605 | -0.11/0.26 | -1.0 | -0.9 | -1.63 | FALSE |
| ERX371683 | TFs and others | BRD4 | Breast | Mammary epithelial cells | 734 | 87/31605 | 0.03/0.26 | -1.0 | -0.9 | -1.63 | FALSE |
| SRX155766 | TFs and others | HSF1 | Breast | Breast cancer cells | 816 | 36/31605 | -0.09/0.26 | -1.0 | -0.9 | -1.62 | FALSE |
| SRX1012763 | TFs and others | PGR | Breast | MCF-7 | 956 | 22/31605 | -0.19/0.26 | -1.0 | -0.9 | -1.62 | FALSE |
| SRX1012751 | TFs and others | PGR | Breast | MCF-7 | 669 | 5/31605 | 1.18/0.26 | -1.0 | -0.9 | 1.62 | TRUE |
| SRX17657149 | TFs and others | FOXC1 | Breast | BT-549 | 1900 | 110/31605 | 0.06/0.26 | -1.0 | -0.9 | -1.62 | FALSE |
| SRX3229170 | TFs and others | AR | Breast | Breast cancer | 1370 | 43/31605 | -0.06/0.26 | -1.0 | -0.9 | -1.61 | FALSE |
| SRX5359052 | TFs and others | RORC | Breast | HCC70 | 24305 | 7751/31605 | 0.23/0.26 | -1.0 | -0.9 | -1.60 | FALSE |
| SRX9688336 | TFs and others | SP1 | Breast | ZR-75-1 | 765 | 6/31605 | -0.58/0.26 | -1.0 | -0.9 | -1.60 | FALSE |
| SRX7522854 | TFs and others | BCL9 | Breast | MCF10DCIS.com | 863 | 75/31605 | 0.02/0.26 | -1.0 | -0.9 | -1.60 | FALSE |
| SRX13581732 | TFs and others | MED1 | Breast | HMLE | 844 | 38/31605 | -0.07/0.26 | -0.9 | -0.9 | -1.57 | FALSE |
| SRX285744 | TFs and others | HOXB7 | Breast | BT-474 | 352 | 16/31605 | -0.25/0.26 | -0.9 | -0.9 | -1.57 | FALSE |
| SRX647704 | TFs and others | TP53 | Breast | MDA-MB-175-VII | 2329 | 19/31605 | -0.20/0.26 | -0.9 | -0.9 | -1.57 | FALSE |
| SRX2578915 | TFs and others | EP300 | Breast | MDA-MB-231 | 652 | 4/31605 | -0.75/0.26 | -0.9 | -0.9 | -1.56 | FALSE |
| SRX3092923 | TFs and others | EP300 | Breast | MDA-MB-231 | 778 | 4/31605 | -0.75/0.26 | -0.9 | -0.9 | -1.56 | FALSE |
| SRX7030916 | TFs and others | TRIM28 | Breast | MCF-7 | 1363 | 39/31605 | -0.06/0.26 | -0.9 | -0.9 | -1.56 | FALSE |
| SRX7130720 | TFs and others | TP53 | Breast | MCF 10A | 760 | 30/31605 | -0.11/0.26 | -0.9 | -0.9 | -1.56 | FALSE |
| SRX10859418 | TFs and others | SS18 | Breast | BT-549 | 2716 | 95/31605 | 0.46/0.26 | -0.9 | -0.9 | 1.56 | TRUE |
| SRX100274 | TFs and others | CARM1 | Breast | H3396 | 323 | 8/31605 | -0.45/0.26 | -0.9 | -0.9 | -1.55 | FALSE |
| SRX822004 | TFs and others | BPTF | Breast | T-47D | 36 | 1/31605 | 2.23/0.26 | -0.9 | -0.9 | 1.55 | TRUE |
| SRX026904 | TFs and others | TRIM24 | Breast | MCF-7 | 62 | 6/31605 | -0.55/0.26 | -0.9 | -0.9 | -1.54 | FALSE |
| SRX7659599 | TFs and others | C17orf49 | Breast | MCF-7 | 480 | 11/31605 | -0.34/0.26 | -0.9 | -0.9 | -1.54 | FALSE |
| SRX23330422 | TFs and others | HDAC6 | Breast | MDA-MB-231 | 562 | 72/31605 | 0.02/0.26 | -0.9 | -0.9 | -1.54 | FALSE |
| SRX1044415 | TFs and others | RELA | Breast | MDA-MB-231 | 1551 | 51/31605 | -0.02/0.26 | -0.9 | -0.9 | -1.54 | FALSE |
| SRX17657164 | TFs and others | FOXC1 | Breast | MDA-MB-468 | 3671 | 307/31605 | 0.14/0.26 | -0.9 | -0.9 | -1.53 | FALSE |
| SRX19024869 | TFs and others | NR3C1 | Breast | MCF-7 | 756 | 7/31605 | -0.49/0.26 | -0.9 | -0.9 | -1.53 | FALSE |
| SRX17682349 | TFs and others | RELA | Breast | MCF-7 | 985 | 7/31605 | -0.48/0.26 | -0.9 | -0.9 | -1.53 | FALSE |
| SRX4671315 | TFs and others | EP300 | Breast | SUM 159PT | 1891 | 109/31605 | 0.07/0.26 | -0.9 | -0.9 | -1.53 | FALSE |
| SRX15039978 | TFs and others | Epitope tags | Breast | MDA-MB-231 | 2286 | 225/31605 | 0.12/0.26 | -0.9 | -0.9 | -1.53 | FALSE |
| SRX822010 | TFs and others | CBX3 | Breast | T-47D | 1351 | 19/31605 | -0.19/0.26 | -0.9 | -0.9 | -1.52 | FALSE |
| SRX13327998 | TFs and others | Epitope tags | Breast | MCF-7 | 824 | 4/31605 | -0.72/0.26 | -0.9 | -0.9 | -1.52 | FALSE |
| SRX5511303 | TFs and others | ESR1 | Breast | MDA-MB-231 | 767 | 5/31605 | -0.62/0.26 | -0.9 | -0.9 | -1.52 | FALSE |
| SRX155767 | TFs and others | HSF1 | Breast | Breast cancer cells | 775 | 32/31605 | -0.09/0.26 | -0.9 | -0.9 | -1.52 | FALSE |
| SRX4116043 | TFs and others | FOXA1 | Breast | Breast cancer cells | 260 | 3/31605 | -0.87/0.26 | -0.9 | -0.9 | -1.52 | FALSE |
| SRX15232270 | TFs and others | DNA-RNA hybrid | Breast | MDA-MB-436 | 278 | 5/31605 | -0.61/0.26 | -0.9 | -0.9 | -1.51 | FALSE |
| SRX3595674 | TFs and others | BRD3 | Breast | MCF-7 | 1134 | 24/31605 | -0.14/0.26 | -0.9 | -0.8 | -1.51 | FALSE |
| SRX17682352 | TFs and others | RELA | Breast | MCF-7 | 748 | 9/31605 | -0.39/0.26 | -0.9 | -0.8 | -1.51 | FALSE |
| SRX4017879 | TFs and others | TP63 | Breast | Breast organoids | 1738 | 19/31605 | -0.19/0.26 | -0.9 | -0.8 | -1.51 | FALSE |
| SRX18014926 | TFs and others | SMAD2 | Breast | MCF 10A | 855 | 7/31605 | -0.46/0.26 | -0.9 | -0.8 | -1.48 | FALSE |
| SRX4417027 | TFs and others | TFAP2C | Breast | MCF-7 | 1085 | 22/31605 | -0.15/0.26 | -0.9 | -0.8 | -1.48 | FALSE |
| SRX2578919 | TFs and others | EP300 | Breast | MDA-MB-231 | 803 | 16/31605 | -0.22/0.26 | -0.9 | -0.8 | -1.48 | FALSE |
| SRX647703 | TFs and others | TP53 | Breast | MCF-7 | 829 | 28/31605 | -0.10/0.26 | -0.9 | -0.8 | -1.48 | FALSE |
| SRX5734998 | TFs and others | GFP | Breast | MCF 10A | 23439 | 690/31605 | 0.18/0.26 | -0.9 | -0.8 | -1.48 | FALSE |
| SRX18191675 | TFs and others | ESR1 | Breast | MCF-7 | 926 | 4/31605 | -0.69/0.26 | -0.9 | -0.8 | -1.48 | FALSE |
| SRX1100309 | TFs and others | RELA | Breast | HMEC | 818 | 37/31605 | -0.05/0.26 | -0.8 | -0.8 | -1.46 | FALSE |
| SRX20152811 | TFs and others | FOXA1 | Breast | MDA-MB-231 | 2920 | 225/31605 | 0.13/0.26 | -0.8 | -0.8 | -1.46 | FALSE |
| SRX8886226 | TFs and others | NFKB1 | Breast | MDA-MB-231 | 552 | 15/31605 | -0.23/0.26 | -0.8 | -0.8 | -1.46 | FALSE |
| SRX19767354 | TFs and others | BrdU | Breast | MCF-7 | 112 | 2/31605 | -1.06/0.26 | -0.8 | -0.8 | -1.46 | FALSE |
| SRX19767356 | TFs and others | BrdU | Breast | MCF-7 | 111 | 2/31605 | -1.06/0.26 | -0.8 | -0.8 | -1.46 | FALSE |
| SRX4393797 | TFs and others | SIX2 | Breast | MCF-7 | 279 | 2/31605 | -1.06/0.26 | -0.8 | -0.8 | -1.46 | FALSE |
| SRX8488022 | TFs and others | Epitope tags | Breast | MDA-MB-231 | 274 | 2/31605 | -1.06/0.26 | -0.8 | -0.8 | -1.46 | FALSE |
| SRX8488024 | TFs and others | Epitope tags | Breast | MDA-MB-231 | 867 | 2/31605 | -1.06/0.26 | -0.8 | -0.8 | -1.46 | FALSE |
| SRX8488025 | TFs and others | Epitope tags | Breast | MDA-MB-231 | 695 | 2/31605 | -1.06/0.26 | -0.8 | -0.8 | -1.46 | FALSE |
| SRX5089545 | TFs and others | ARID2 | Breast | MCF-7 | 677 | 8/31605 | -0.40/0.26 | -0.8 | -0.8 | -1.45 | FALSE |
| SRX2915395 | TFs and others | PGR | Breast | T-47D | 104 | 3/31605 | -0.82/0.26 | -0.8 | -0.8 | -1.45 | FALSE |
| ERX371664 | TFs and others | BRD4 | Breast | Mammary epithelial cells | 257 | 14/31605 | -0.24/0.26 | -0.8 | -0.8 | -1.45 | FALSE |
| SRX5323887 | TFs and others | STAT3 | Breast | T-47D | 862 | 11/31605 | -0.30/0.26 | -0.8 | -0.8 | -1.45 | FALSE |
| SRX15039971 | TFs and others | Epitope tags | Breast | MDA-MB-231 | 443 | 13/31605 | -0.26/0.26 | -0.8 | -0.8 | -1.44 | FALSE |
| SRX968975 | TFs and others | RELA | Breast | MCF-7 | 418 | 77/31605 | 0.05/0.26 | -0.8 | -0.8 | -1.43 | FALSE |
| SRX15039972 | TFs and others | Epitope tags | Breast | MDA-MB-231 | 654 | 38/31605 | -0.04/0.26 | -0.8 | -0.8 | -1.43 | FALSE |
| SRX10164656 | TFs and others | PAD2 | Breast | MCF-7 | 1465 | 11/31605 | -0.30/0.26 | -0.8 | -0.8 | -1.43 | FALSE |
| SRX365497 | TFs and others | PGR | Breast | T-47D | 101 | 12/31605 | -0.27/0.26 | -0.8 | -0.8 | -1.42 | FALSE |
| SRX3629595 | TFs and others | ESR1 | Breast | MCF-7 | 91 | 5/31605 | -0.56/0.26 | -0.8 | -0.8 | -1.42 | FALSE |
| SRX1772413 | TFs and others | Epitope tags | Breast | MCF-7 | 1154 | 19/31605 | -0.16/0.26 | -0.8 | -0.8 | -1.41 | FALSE |
| SRX1012757 | TFs and others | PGR | Breast | MCF-7 | 753 | 8/31605 | -0.38/0.26 | -0.8 | -0.8 | -1.41 | FALSE |
| SRX10164668 | TFs and others | PAD2 | Breast | MCF-7 | 2008 | 10/31605 | -0.31/0.26 | -0.8 | -0.8 | -1.41 | FALSE |
| SRX13581733 | TFs and others | MED1 | Breast | HMLE | 1119 | 11/31605 | -0.29/0.26 | -0.8 | -0.8 | -1.41 | FALSE |
| SRX1070165 | TFs and others | PIAS1 | Breast | MDA-MB-453 | 1023 | 11/31605 | -0.29/0.26 | -0.8 | -0.8 | -1.40 | FALSE |
| SRX3447358 | TFs and others | ESR1 | Breast | MCF-7 | 740 | 7/31605 | -0.42/0.26 | -0.8 | -0.8 | -1.40 | FALSE |
| SRX17834043 | TFs and others | TP63 | Breast | MCF 10A | 940 | 11/31605 | -0.29/0.26 | -0.8 | -0.8 | -1.40 | FALSE |
| SRX10365519 | TFs and others | RAC1 | Breast | MCF-7 | 594 | 18/31605 | -0.17/0.26 | -0.8 | -0.8 | -1.40 | FALSE |
| SRX10664179 | TFs and others | SUZ12 | Breast | MCF-7 | 4258 | 748/31605 | 0.32/0.26 | -0.8 | -0.8 | 1.40 | TRUE |
| SRX4672372 | TFs and others | ARNT | Breast | MCF-7 | 206 | 6/31605 | -0.47/0.26 | -0.8 | -0.8 | -1.39 | FALSE |
| SRX022741 | TFs and others | TDRD3 | Breast | MCF-7 | 1206 | 68/31605 | 0.04/0.26 | -0.8 | -0.7 | -1.38 | FALSE |
| SRX2424527 | TFs and others | YBX1 | Breast | MCF-7 | 4800 | 190/31605 | 0.13/0.26 | -0.8 | -0.7 | -1.38 | FALSE |
| SRX13323810 | TFs and others | GFP | Breast | MDA-MB-231 | 3403 | 185/31605 | 0.13/0.26 | -0.8 | -0.7 | -1.37 | FALSE |
| SRX10205613 | TFs and others | SMARCA4 | Breast | CAL-120 | 803 | 20/31605 | -0.14/0.26 | -0.8 | -0.7 | -1.37 | FALSE |
| SRX6895234 | TFs and others | 8-Hydroxydeoxyguanosine | Breast | MCF 10A | 27142 | 2083/31605 | 0.29/0.26 | -0.8 | -0.7 | 1.37 | TRUE |
| SRX13254867 | TFs and others | SUZ12 | Breast | MCF-7 | 13074 | 1208/31605 | 0.20/0.26 | -0.8 | -0.7 | -1.37 | FALSE |
| SRX13993206 | TFs and others | EBF1 | Breast | MB 157 | 315 | 5/31605 | -0.53/0.26 | -0.8 | -0.7 | -1.36 | FALSE |
| SRX155763 | TFs and others | HSF1 | Breast | BPE | 1253 | 171/31605 | 0.12/0.26 | -0.8 | -0.7 | -1.36 | FALSE |
| SRX3011700 | TFs and others | MED12 | Breast | MCF-7 | 246 | 6/31605 | -0.46/0.26 | -0.8 | -0.7 | -1.36 | FALSE |
| SRX1012754 | TFs and others | PGR | Breast | MCF-7 | 620 | 11/31605 | -0.27/0.26 | -0.8 | -0.7 | -1.36 | FALSE |
| SRX7522855 | TFs and others | BCL9 | Breast | MCF10DCIS.com | 2134 | 156/31605 | 0.12/0.26 | -0.8 | -0.7 | -1.35 | FALSE |
| SRX18895257 | TFs and others | Epitope tags | Breast | MCF-7 | 1690 | 13/31605 | -0.22/0.26 | -0.7 | -0.7 | -1.35 | FALSE |
| SRX7657046 | TFs and others | NCOA3 | Breast | MCF 10A | 909 | 4/31605 | -0.61/0.26 | -0.7 | -0.7 | -1.35 | FALSE |
| SRX15039976 | TFs and others | Epitope tags | Breast | MDA-MB-231 | 3005 | 282/31605 | 0.15/0.26 | -0.7 | -0.7 | -1.35 | FALSE |
| SRX3862458 | TFs and others | GATA3 | Breast | T-47D | 809 | 36/31605 | -0.03/0.26 | -0.7 | -0.7 | -1.34 | FALSE |
| SRX8886230 | TFs and others | NFKB1 | Breast | MDA-MB-231 | 466 | 41/31605 | -0.01/0.26 | -0.7 | -0.7 | -1.34 | FALSE |
| SRX115968 | TFs and others | ESR1 | Breast | MCF-7 | 1090 | 10/31605 | -0.29/0.26 | -0.7 | -0.7 | -1.34 | FALSE |
| SRX6058756 | TFs and others | PGR | Breast | T-47D | 1578 | 5/31605 | -0.51/0.26 | -0.7 | -0.7 | -1.33 | FALSE |
| SRX10164571 | TFs and others | ESR1 | Breast | MCF-7 | 2709 | 15/31605 | -0.18/0.26 | -0.7 | -0.7 | -1.32 | FALSE |
| SRX22289053 | TFs and others | LMNB1 | Breast | MCF 10A | 1008 | 4/31605 | -0.59/0.26 | -0.7 | -0.7 | -1.32 | FALSE |
| SRX22289055 | TFs and others | LMNB1 | Breast | MCF 10A | 1303 | 4/31605 | -0.59/0.26 | -0.7 | -0.7 | -1.32 | FALSE |
| SRX10164592 | TFs and others | PAD2 | Breast | MCF-7 | 1167 | 4/31605 | -0.59/0.26 | -0.7 | -0.7 | -1.32 | FALSE |
| SRX11374760 | TFs and others | PGR | Breast | T-47D | 1114 | 4/31605 | -0.59/0.26 | -0.7 | -0.7 | -1.32 | FALSE |
| SRX11374762 | TFs and others | PGR | Breast | T-47D | 1177 | 4/31605 | -0.59/0.26 | -0.7 | -0.7 | -1.32 | FALSE |
| SRX2582041 | TFs and others | KDM4A | Breast | BCSC1 | 759 | 4/31605 | -0.59/0.26 | -0.7 | -0.7 | -1.32 | FALSE |
| SRX9605785 | TFs and others | TP53 | Breast | MCF 10A | 719 | 4/31605 | -0.59/0.26 | -0.7 | -0.7 | -1.32 | FALSE |
| SRX9605787 | TFs and others | TP53 | Breast | MCF 10A | 834 | 4/31605 | -0.59/0.26 | -0.7 | -0.7 | -1.32 | FALSE |
| SRX1012624 | TFs and others | EP300 | Breast | MCF-7 | 1544 | 268/31605 | 0.15/0.26 | -0.7 | -0.7 | -1.32 | FALSE |
| SRX1012752 | TFs and others | PGR | Breast | MCF-7 | 974 | 8/31605 | 0.85/0.26 | -0.7 | -0.7 | 1.31 | TRUE |
| SRX1995221 | TFs and others | ESR1 | Breast | MCF-7 | 227 | 3/31605 | -0.71/0.26 | -0.7 | -0.7 | -1.31 | FALSE |
| SRX1161195 | TFs and others | NR3C1 | Breast | T-47D | 907 | 12/31605 | -0.23/0.26 | -0.7 | -0.7 | -1.31 | FALSE |
| SRX5089785 | TFs and others | SATB1 | Breast | MCF 10A | 35491 | 1159/31605 | 0.21/0.26 | -0.7 | -0.7 | -1.31 | FALSE |
| SRX155723 | TFs and others | HSF1 | Breast | HMLE | 407 | 9/31605 | -0.30/0.26 | -0.7 | -0.7 | -1.30 | FALSE |
| SRX18895256 | TFs and others | Epitope tags | Breast | MCF-7 | 1631 | 7/31605 | -0.37/0.26 | -0.7 | -0.7 | -1.30 | FALSE |
| SRX1012656 | TFs and others | PGR | Breast | MCF-7 | 885 | 142/31605 | 0.12/0.26 | -0.7 | -0.7 | -1.30 | FALSE |
| SRX1100310 | TFs and others | RELA | Breast | HMEC | 1098 | 53/31605 | 0.03/0.26 | -0.7 | -0.7 | -1.30 | FALSE |
| SRX19662628 | TFs and others | KDM1A | Breast | T-47D | 37 | 1/31605 | -1.39/0.26 | -0.7 | -0.7 | -1.28 | FALSE |
| ERX371686 | TFs and others | BRD4 | Breast | Mammary epithelial cells | 86 | 10/31605 | -0.26/0.26 | -0.7 | -0.7 | -1.27 | FALSE |
| SRX12019752 | TFs and others | STAT2 | Breast | MCF-7 | 272 | 76/31605 | 0.44/0.26 | -0.7 | -0.7 | 1.27 | TRUE |
| SRX11374758 | TFs and others | PGR | Breast | T-47D | 1145 | 5/31605 | -0.47/0.26 | -0.7 | -0.7 | -1.27 | FALSE |
| SRX968973 | TFs and others | RELA | Breast | MCF-7 | 143 | 23/31605 | -0.08/0.26 | -0.7 | -0.7 | -1.27 | FALSE |
| SRX7030919 | TFs and others | TRIM28 | Breast | MCF-7 | 1139 | 36/31605 | -0.01/0.26 | -0.7 | -0.7 | -1.26 | FALSE |
| SRX2897232 | TFs and others | ESR1 | Breast | Breast cancer cells | 5843 | 112/31605 | 0.10/0.26 | -0.7 | -0.6 | -1.25 | FALSE |
| SRX1012737 | TFs and others | ESR1 | Breast | MCF-7 | 587 | 12/31605 | -0.21/0.26 | -0.7 | -0.6 | -1.25 | FALSE |
| SRX8918907 | TFs and others | Epitope tags | Breast | MDA-MB-231 | 491 | 8/31605 | -0.31/0.26 | -0.7 | -0.6 | -1.24 | FALSE |
| SRX11005601 | TFs and others | DNMT3B | Breast | MCF10CA1a | 2303 | 108/31605 | 0.41/0.26 | -0.7 | -0.6 | 1.24 | TRUE |
| SRX4561218 | TFs and others | ANAPC1 | Breast | MCF-7 | 2163 | 25/31605 | -0.06/0.26 | -0.7 | -0.6 | -1.24 | FALSE |
| SRX15929734 | TFs and others | STAG1 | Breast | MCF 10A | 796 | 11/31605 | -0.22/0.26 | -0.7 | -0.6 | -1.24 | FALSE |
| SRX6712589 | TFs and others | MED1 | Breast | MCF-7 | 2777 | 14/31605 | -0.17/0.26 | -0.7 | -0.6 | -1.23 | FALSE |
| SRX10664190 | TFs and others | MTF2 | Breast | MCF-7 | 1670 | 200/31605 | 0.37/0.26 | -0.7 | -0.6 | 1.23 | TRUE |
| SRX3322196 | TFs and others | EP300 | Breast | Breast epithelium | 1571 | 9/31605 | -0.27/0.26 | -0.7 | -0.6 | -1.22 | FALSE |
| SRX9322285 | TFs and others | ESR1 | Breast | MCF-7 | 1328 | 78/31605 | 0.08/0.26 | -0.7 | -0.6 | -1.22 | FALSE |
| SRX2424528 | TFs and others | YBX1 | Breast | MCF-7 | 2970 | 93/31605 | 0.09/0.26 | -0.7 | -0.6 | -1.22 | FALSE |
| SRX6027754 | TFs and others | NR2F2 | Breast | MCF-7 | 30507 | 3962/31605 | 0.28/0.26 | -0.7 | -0.6 | 1.22 | TRUE |
| SRX5088593 | TFs and others | Cas9 | Breast | MDA-MB-231 | 624 | 6/31605 | -0.38/0.26 | -0.6 | -0.6 | -1.22 | FALSE |
| SRX4561215 | TFs and others | TRPS1 | Breast | MCF-7 | 5145 | 146/31605 | 0.13/0.26 | -0.6 | -0.6 | -1.21 | FALSE |
| SRX039142 | TFs and others | JUN | Breast | MCF-7 | 423 | 2/31605 | 1.35/0.26 | -0.6 | -0.6 | 1.21 | TRUE |
| SRX1339015 | TFs and others | AR | Breast | MDA-MB-453 | 536 | 6/31605 | -0.38/0.26 | -0.6 | -0.6 | -1.21 | FALSE |
| SRX14756937 | TFs and others | DNA-RNA hybrid | Breast | MDA-MB-436 | 1709 | 475/31605 | 0.18/0.26 | -0.6 | -0.6 | -1.20 | FALSE |
| SRX3990980 | TFs and others | PGR | Breast | T-47D | 659 | 34/31605 | -0.01/0.26 | -0.6 | -0.6 | -1.20 | FALSE |
| SRX4561229 | TFs and others | Biotin | Breast | MCF-7 | 586 | 44/31605 | 0.02/0.26 | -0.6 | -0.6 | -1.20 | FALSE |
| SRX155719 | TFs and others | HSF1 | Breast | HMEC | 296 | 50/31605 | 0.04/0.26 | -0.6 | -0.6 | -1.20 | FALSE |
| SRX11374759 | TFs and others | PGR | Breast | T-47D | 1039 | 6/31605 | -0.37/0.26 | -0.6 | -0.6 | -1.19 | FALSE |
| SRX17682350 | TFs and others | RELA | Breast | MCF-7 | 788 | 28/31605 | 0.54/0.26 | -0.6 | -0.6 | 1.19 | TRUE |
| SRX13581731 | TFs and others | MED1 | Breast | HMLE | 945 | 16/31605 | -0.13/0.26 | -0.6 | -0.6 | -1.19 | FALSE |
| SRX23330425 | TFs and others | HDAC6 | Breast | MDA-MB-231 | 388 | 54/31605 | 0.05/0.26 | -0.6 | -0.6 | -1.19 | FALSE |
| SRX396573 | TFs and others | RNF2 | Breast | Breast cells | 1355 | 92/31605 | 0.10/0.26 | -0.6 | -0.6 | -1.19 | FALSE |
| SRX17682351 | TFs and others | RELA | Breast | MCF-7 | 815 | 6/31605 | -0.36/0.26 | -0.6 | -0.6 | -1.19 | FALSE |
| SRX100259 | TFs and others | CREBBP | Breast | H3396 | 5746 | 394/31605 | 0.18/0.26 | -0.6 | -0.6 | -1.18 | FALSE |
| SRX11005607 | TFs and others | POU2F1 | Breast | MCF10CA1a | 1077 | 41/31605 | 0.02/0.26 | -0.6 | -0.6 | -1.18 | FALSE |
| SRX155764 | TFs and others | HSF1 | Breast | HMEC | 851 | 99/31605 | 0.10/0.26 | -0.6 | -0.6 | -1.17 | FALSE |
| SRX4649667 | TFs and others | TP53 | Breast | MCF 10A | 1600 | 36/31605 | 0.01/0.26 | -0.6 | -0.6 | -1.17 | FALSE |
| SRX9534928 | TFs and others | NR4A1 | Breast | Breast cancer cells | 148 | 20/31605 | -0.08/0.26 | -0.6 | -0.6 | -1.17 | FALSE |
| SRX423001 | TFs and others | CDK2 | Breast | T-47D | 1415 | 17/31605 | -0.11/0.26 | -0.6 | -0.6 | -1.16 | FALSE |
| ERX371660 | TFs and others | BRD4 | Breast | Mammary epithelial cells | 294 | 2/31605 | -0.80/0.26 | -0.6 | -0.6 | -1.16 | FALSE |
| ERX371693 | TFs and others | BCL11A | Breast | Mammary epithelial cells | 227 | 2/31605 | -0.80/0.26 | -0.6 | -0.6 | -1.16 | FALSE |
| ERX371705 | TFs and others | BRD4 | Breast | Mammary epithelial cells | 389 | 2/31605 | -0.80/0.26 | -0.6 | -0.6 | -1.16 | FALSE |
| SRX19024865 | TFs and others | NR3C1 | Breast | MCF-7 | 681 | 5/31605 | -0.41/0.26 | -0.6 | -0.6 | -1.16 | FALSE |
| SRX3595671 | TFs and others | BRD3 | Breast | MCF-7 | 772 | 23/31605 | -0.05/0.26 | -0.6 | -0.6 | -1.15 | FALSE |
| ERX371694 | TFs and others | BRD4 | Breast | Mammary epithelial cells | 302 | 18/31605 | -0.09/0.26 | -0.6 | -0.6 | -1.15 | FALSE |
| SRX1757999 | TFs and others | MYC | Breast | MDA-MB-468 | 421 | 7/31605 | -0.30/0.26 | -0.6 | -0.6 | -1.15 | FALSE |
| ERX371668 | TFs and others | BRD4 | Breast | Mammary epithelial cells | 57 | 14/31605 | 0.65/0.26 | -0.6 | -0.6 | 1.15 | TRUE |
| SRX5089514 | TFs and others | SMARCA4 | Breast | MCF-7 | 601 | 9/31605 | -0.24/0.26 | -0.6 | -0.6 | -1.15 | FALSE |
| SRX1772416 | TFs and others | NEDD4 | Breast | MCF-7 | 768 | 7/31605 | -0.30/0.26 | -0.6 | -0.6 | -1.15 | FALSE |
| SRX7642859 | TFs and others | RBM39 | Breast | Mammary epithelial cells | 121 | 3/31605 | -0.59/0.26 | -0.6 | -0.6 | -1.15 | FALSE |
| SRX3110747 | TFs and others | ESR1 | Breast | MCF-7 | 245 | 12/31605 | -0.17/0.26 | -0.6 | -0.6 | -1.14 | FALSE |
| SRX10664186 | TFs and others | JARID2 | Breast | MCF-7 | 1942 | 98/31605 | 0.11/0.26 | -0.6 | -0.6 | -1.14 | FALSE |
| SRX4096888 | TFs and others | TRPS1 | Breast | HCC3153 | 4782 | 268/31605 | 0.17/0.26 | -0.6 | -0.6 | -1.13 | FALSE |
| SRX10365518 | TFs and others | RAC1 | Breast | MCF-7 | 773 | 27/31605 | -0.02/0.26 | -0.6 | -0.6 | -1.13 | FALSE |
| SRX016326 | TFs and others | ESR1 | Breast | MCF-7 | 184 | 13/31605 | -0.14/0.26 | -0.6 | -0.6 | -1.12 | FALSE |
| SRX4116041 | TFs and others | ESR1 | Breast | Breast cancer cells | 245 | 2/31605 | -0.76/0.26 | -0.6 | -0.6 | -1.12 | FALSE |
| SRX7522856 | TFs and others | STAT3 | Breast | MCF10DCIS.com | 7743 | 580/31605 | 0.31/0.26 | -0.6 | -0.6 | 1.12 | TRUE |
| SRX1995217 | TFs and others | CTCF | Breast | MCF-7 | 780 | 49/31605 | 0.05/0.26 | -0.6 | -0.6 | -1.12 | FALSE |
| SRX5231785 | TFs and others | MYC | Breast | UACC-812 | 848 | 29/31605 | 0.52/0.26 | -0.6 | -0.6 | 1.11 | TRUE |
| SRX423005 | TFs and others | PARP1 | Breast | T-47D | 1878 | 21/31605 | -0.05/0.26 | -0.6 | -0.5 | -1.10 | FALSE |
| SRX8488023 | TFs and others | Epitope tags | Breast | MDA-MB-231 | 1173 | 10/31605 | -0.19/0.26 | -0.6 | -0.5 | -1.10 | FALSE |
| SRX545943 | TFs and others | MBD2 | Breast | MCF-7 | 1552 | 434/31605 | 0.32/0.26 | -0.6 | -0.5 | 1.09 | TRUE |
| SRX20830488 | TFs and others | YAP1 | Breast | T-47D | 277 | 4/31605 | -0.44/0.26 | -0.6 | -0.5 | -1.09 | FALSE |
| SRX11005611 | TFs and others | POU2F1 | Breast | MCF10CA1a | 942 | 24/31605 | -0.03/0.26 | -0.6 | -0.5 | -1.09 | FALSE |
| SRX5179249 | TFs and others | SIPA1 | Breast | MDA-MB-231 | 912 | 60/31605 | 0.08/0.26 | -0.6 | -0.5 | -1.08 | FALSE |
| SRX396569 | TFs and others | CBX7 | Breast | Breast cells | 1245 | 86/31605 | 0.40/0.26 | -0.6 | -0.5 | 1.08 | TRUE |
| SRX8886229 | TFs and others | NFKB1 | Breast | MDA-MB-231 | 667 | 78/31605 | 0.10/0.26 | -0.5 | -0.5 | -1.07 | FALSE |
| SRX1521304 | TFs and others | EP300 | Breast | ZR-75-30 | 1995 | 79/31605 | 0.41/0.26 | -0.5 | -0.5 | 1.07 | TRUE |
| SRX2924012 | TFs and others | TP53 | Breast | MCF-7 | 711 | 40/31605 | 0.04/0.26 | -0.5 | -0.5 | -1.07 | FALSE |
| SRX13327973 | TFs and others | Epitope tags | Breast | MCF-7 | 1255 | 9/31605 | -0.20/0.26 | -0.5 | -0.5 | -1.07 | FALSE |
| SRX10024470 | TFs and others | ESR1 | Breast | MCF-7 | 894 | 6/31605 | -0.30/0.26 | -0.5 | -0.5 | -1.06 | FALSE |
| SRX5057615 | TFs and others | Epitope tags | Breast | T-47D | 644 | 5/31605 | -0.35/0.26 | -0.5 | -0.5 | -1.06 | FALSE |
| SRX2194240 | TFs and others | EP300 | Breast | SUM 159PT | 1579 | 20/31605 | -0.05/0.26 | -0.5 | -0.5 | -1.06 | FALSE |
| SRX5511308 | TFs and others | ESR1 | Breast | MDA-MB-231 | 795 | 6/31605 | -0.30/0.26 | -0.5 | -0.5 | -1.06 | FALSE |
| SRX11005608 | TFs and others | POU2F1 | Breast | MCF10CA1a | 842 | 22/31605 | -0.03/0.26 | -0.5 | -0.5 | -1.05 | FALSE |
| SRX2578867 | TFs and others | ESR1 | Breast | MDA-MB-231 | 28 | 5/31605 | -0.35/0.26 | -0.5 | -0.5 | -1.05 | FALSE |
| SRX285742 | TFs and others | HOXB7 | Breast | BT-474 | 183 | 13/31605 | -0.12/0.26 | -0.5 | -0.5 | -1.05 | FALSE |
| SRX3595672 | TFs and others | BRD3 | Breast | MCF-7 | 1114 | 16/31605 | -0.08/0.26 | -0.5 | -0.5 | -1.04 | FALSE |
| SRX1709953 | TFs and others | ESR1 | Breast | T-47D | 63 | 30/31605 | 0.01/0.26 | -0.5 | -0.5 | -1.04 | FALSE |
| SRX2453442 | TFs and others | NLRP2 | Breast | MCF 10A | 897 | 54/31605 | 0.07/0.26 | -0.5 | -0.5 | -1.04 | FALSE |
| ERX2242387 | TFs and others | ARID1A | Breast | Mammary epithelial cells | 252 | 1/31605 | -1.08/0.26 | -0.5 | -0.5 | -1.04 | FALSE |
| ERX2242399 | TFs and others | ARID1A | Breast | Mammary epithelial cells | 250 | 1/31605 | -1.08/0.26 | -0.5 | -0.5 | -1.04 | FALSE |
| SRX13845690 | TFs and others | HSF2 | Breast | MDA-MB-231 | 284 | 1/31605 | -1.08/0.26 | -0.5 | -0.5 | -1.04 | FALSE |
| SRX18665936 | TFs and others | ZNF652 | Breast | MCF-7 | 1237 | 1/31605 | -1.08/0.26 | -0.5 | -0.5 | -1.04 | FALSE |
| SRX4561227 | TFs and others | Biotin | Breast | MCF-7 | 389 | 1/31605 | -1.08/0.26 | -0.5 | -0.5 | -1.04 | FALSE |
| SRX2194242 | TFs and others | EP300 | Breast | SUM 159PT | 4941 | 312/31605 | 0.18/0.26 | -0.5 | -0.5 | -1.04 | FALSE |
| SRX10399321 | TFs and others | KMT2A | Breast | Breast epithelium | 791 | 30/31605 | 0.01/0.26 | -0.5 | -0.5 | -1.04 | FALSE |
| SRX4815196 | TFs and others | TLE3 | Breast | SUM 159PT | 17735 | 1008/31605 | 0.21/0.26 | -0.5 | -0.5 | -1.04 | FALSE |
| SRX1012657 | TFs and others | PGR | Breast | MCF-7 | 749 | 118/31605 | 0.13/0.26 | -0.5 | -0.5 | -1.04 | FALSE |
| SRX7130731 | TFs and others | TP53 | Breast | MCF 10A | 1878 | 98/31605 | 0.12/0.26 | -0.5 | -0.5 | -1.03 | FALSE |
| SRX11005612 | TFs and others | POU2F1 | Breast | MCF10CA1a | 1009 | 21/31605 | -0.03/0.26 | -0.5 | -0.5 | -1.03 | FALSE |
| SRX1012646 | TFs and others | EP300 | Breast | MCF-7 | 1585 | 257/31605 | 0.17/0.26 | -0.5 | -0.5 | -1.02 | FALSE |
| SRX9534893 | TFs and others | NR4A1 | Breast | MCF 10A | 245 | 2/31605 | 1.18/0.26 | -0.5 | -0.5 | 1.02 | TRUE |
| SRX13993208 | TFs and others | EBF1 | Breast | MB 157 | 373 | 6/31605 | -0.28/0.26 | -0.5 | -0.5 | -1.02 | FALSE |
| SRX8321655 | TFs and others | CDYL2 | Breast | MCF-7 | 12534 | 5060/31605 | 0.27/0.26 | -0.5 | -0.5 | 1.01 | TRUE |
| SRX4192881 | TFs and others | JUNB | Breast | MCF 10A | 24966 | 1627/31605 | 0.22/0.26 | -0.5 | -0.5 | -1.01 | FALSE |
| SRX1995227 | TFs and others | LMNB1 | Breast | MCF-7 | 373 | 5/31605 | -0.32/0.26 | -0.5 | -0.5 | -1.01 | FALSE |
| SRX758323 | TFs and others | MBD2 | Breast | HMEC | 2541 | 95/31605 | 0.12/0.26 | -0.5 | -0.5 | -1.01 | FALSE |
| SRX16646113 | TFs and others | HSF1 | Breast | MCF-7 | 450 | 3/31605 | -0.49/0.26 | -0.5 | -0.5 | -1.01 | FALSE |
| SRX11005609 | TFs and others | POU2F1 | Breast | MCF10CA1a | 803 | 19/31605 | -0.04/0.26 | -0.5 | -0.5 | -1.01 | FALSE |
| SRX1100311 | TFs and others | RELA | Breast | HMEC | 841 | 49/31605 | 0.07/0.26 | -0.5 | -0.5 | -1.00 | FALSE |
| SRX6712588 | TFs and others | MED1 | Breast | MCF-7 | 4189 | 21/31605 | -0.03/0.26 | -0.5 | -0.5 | -1.00 | FALSE |
| SRX1142763 | TFs and others | JUN | Breast | BT-549 | 2686 | 104/31605 | 0.13/0.26 | -0.5 | -0.5 | -1.00 | FALSE |
| SRX8886228 | TFs and others | NFKB1 | Breast | MDA-MB-231 | 748 | 112/31605 | 0.37/0.26 | -0.5 | -0.5 | 0.99 | TRUE |
| SRX1012645 | TFs and others | EP300 | Breast | MCF-7 | 1025 | 186/31605 | 0.16/0.26 | -0.5 | -0.5 | -0.99 | FALSE |
| SRX5734997 | TFs and others | GFP | Breast | MCF 10A | 15951 | 488/31605 | 0.20/0.26 | -0.5 | -0.5 | -0.99 | FALSE |
| SRX10859401 | TFs and others | ARID1A | Breast | BT-549 | 7119 | 420/31605 | 0.32/0.26 | -0.5 | -0.5 | 0.99 | TRUE |
| SRX1542105 | TFs and others | SRPK1 | Breast | MCF 10A | 967 | 42/31605 | 0.06/0.26 | -0.5 | -0.5 | -0.98 | FALSE |
| SRX17657157 | TFs and others | FOXC1 | Breast | MDA-MB-231 | 1848 | 140/31605 | 0.15/0.26 | -0.5 | -0.5 | -0.98 | FALSE |
| SRX13993203 | TFs and others | EBF1 | Breast | MB 157 | 433 | 59/31605 | 0.09/0.26 | -0.5 | -0.5 | -0.97 | FALSE |
| SRX8886224 | TFs and others | NFKB1 | Breast | MDA-MB-231 | 527 | 22/31605 | -0.01/0.26 | -0.5 | -0.5 | -0.96 | FALSE |
| SRX18895265 | TFs and others | MECP2 | Breast | MCF-7 | 1372 | 11/31605 | -0.12/0.26 | -0.5 | -0.5 | -0.96 | FALSE |
| SRX18014927 | TFs and others | SMAD2 | Breast | MCF 10A | 2103 | 99/31605 | 0.38/0.26 | -0.5 | -0.5 | 0.96 | TRUE |
| SRX518261 | TFs and others | 5-mC | Breast | MCF 10A | 10676 | 861/31605 | 0.21/0.26 | -0.5 | -0.5 | -0.95 | FALSE |
| SRX13340673 | TFs and others | Epitope tags | Breast | T-47D | 54 | 3/31605 | -0.45/0.26 | -0.5 | -0.4 | -0.95 | FALSE |
| SRX1709963 | TFs and others | ESR1 | Breast | T-47D | 85 | 9/31605 | -0.15/0.26 | -0.5 | -0.4 | -0.95 | FALSE |
| SRX100263 | TFs and others | CREBBP | Breast | H3396 | 3078 | 172/31605 | 0.35/0.26 | -0.5 | -0.4 | 0.94 | TRUE |
| SRX13845686 | TFs and others | HSF2 | Breast | MDA-MB-231 | 118 | 27/31605 | 0.03/0.26 | -0.5 | -0.4 | -0.93 | FALSE |
| SRX19767363 | TFs and others | BrdU | Breast | T-47D | 156 | 3/31605 | -0.43/0.26 | -0.4 | -0.4 | -0.92 | FALSE |
| SRX22289056 | TFs and others | LMNB1 | Breast | MCF 10A | 1212 | 7/31605 | -0.18/0.26 | -0.4 | -0.4 | -0.90 | FALSE |
| SRX950754 | TFs and others | TOP2B | Breast | MCF-7 | 449 | 9/31605 | -0.13/0.26 | -0.4 | -0.4 | -0.90 | FALSE |
| SRX312101 | TFs and others | Epitope tags | Breast | MCF-7 | 277 | 2/31605 | 1.06/0.26 | -0.4 | -0.4 | 0.89 | TRUE |
| SRX18014931 | TFs and others | JUN | Breast | MCF 10A | 4056 | 326/31605 | 0.19/0.26 | -0.4 | -0.4 | -0.89 | FALSE |
| ERX371698 | TFs and others | BRD4 | Breast | Mammary epithelial cells | 158 | 27/31605 | 0.04/0.26 | -0.4 | -0.4 | -0.88 | FALSE |
| SRX13845685 | TFs and others | HSF2 | Breast | MDA-MB-231 | 395 | 7/31605 | -0.17/0.26 | -0.4 | -0.4 | -0.87 | FALSE |
| SRX24612539 | TFs and others | FOXA1 | Breast | SK-BR-3 | 1062 | 91/31605 | 0.14/0.26 | -0.4 | -0.4 | -0.87 | FALSE |
| SRX12019745 | TFs and others | STAT2 | Breast | MCF-7 | 773 | 56/31605 | 0.40/0.26 | -0.4 | -0.4 | 0.87 | TRUE |
| ERX371667 | TFs and others | BCL11A | Breast | Mammary epithelial cells | 225 | 6/31605 | -0.20/0.26 | -0.4 | -0.4 | -0.87 | FALSE |
| SRX10664178 | TFs and others | SUZ12 | Breast | MCF-7 | 1379 | 132/31605 | 0.35/0.26 | -0.4 | -0.4 | 0.87 | TRUE |
| SRX11005610 | TFs and others | POU2F1 | Breast | MCF10CA1a | 823 | 20/31605 | 0.01/0.26 | -0.4 | -0.4 | -0.86 | FALSE |
| SRX13993200 | TFs and others | EBF1 | Breast | MB 157 | 434 | 33/31605 | 0.07/0.26 | -0.4 | -0.4 | -0.85 | FALSE |
| SRX4116044 | TFs and others | FOXA1 | Breast | Breast cancer cells | 336 | 11/31605 | -0.07/0.26 | -0.4 | -0.4 | -0.84 | FALSE |
| SRX5824591 | TFs and others | BRD4 | Breast | SUM 159PT | 1361 | 117/31605 | 0.16/0.26 | -0.4 | -0.4 | -0.83 | FALSE |
| SRX10775081 | TFs and others | Epitope tags | Breast | HMLE | 1003 | 12/31605 | 0.56/0.26 | -0.4 | -0.4 | 0.83 | TRUE |
| SRX14812502 | TFs and others | TP53 | Breast | Mammary epithelial cells | 875 | 12/31605 | -0.05/0.26 | -0.4 | -0.4 | -0.83 | FALSE |
| SRX3110726 | TFs and others | ESR1 | Breast | MCF-7 | 833 | 18/31605 | 0.01/0.26 | -0.4 | -0.4 | -0.83 | FALSE |
| SRX1995223 | TFs and others | FOXA1 | Breast | MCF-7 | 774 | 28/31605 | 0.06/0.26 | -0.4 | -0.4 | -0.82 | FALSE |
| SRX9534892 | TFs and others | NR4A1 | Breast | MCF 10A | 274 | 4/31605 | -0.27/0.26 | -0.4 | -0.4 | -0.82 | FALSE |
| SRX14613009 | TFs and others | JUN | Breast | T-47D | 1564 | 45/31605 | 0.10/0.26 | -0.4 | -0.4 | -0.81 | FALSE |
| SRX11005602 | TFs and others | DNMT3B | Breast | MCF10CA1a | 2218 | 108/31605 | 0.35/0.26 | -0.4 | -0.4 | 0.81 | TRUE |
| SRX7423961 | TFs and others | RUVBL2 | Breast | MDA-MB-231 | 2165 | 3/31605 | -0.34/0.26 | -0.4 | -0.4 | -0.80 | FALSE |
| SRX18014933 | TFs and others | JUNB | Breast | MCF 10A | 5894 | 481/31605 | 0.21/0.26 | -0.4 | -0.4 | -0.79 | FALSE |
| SRX673711 | TFs and others | Biotin | Breast | MCF-7 | 603 | 6/31605 | -0.16/0.26 | -0.4 | -0.4 | -0.79 | FALSE |
| SRX9534894 | TFs and others | NR4A1 | Breast | MCF 10A | 227 | 1/31605 | -0.76/0.26 | -0.4 | -0.4 | -0.79 | FALSE |
| SRX9534895 | TFs and others | NR4A1 | Breast | MCF 10A | 252 | 1/31605 | -0.76/0.26 | -0.4 | -0.4 | -0.79 | FALSE |
| SRX14960539 | TFs and others | EZH2 | Breast | MCF-7 | 110 | 6/31605 | 0.67/0.26 | -0.4 | -0.4 | 0.79 | TRUE |
| SRX3110727 | TFs and others | ESR1 | Breast | MCF-7 | 974 | 16/31605 | 0.00/0.26 | -0.4 | -0.4 | -0.79 | FALSE |
| SRX968961 | TFs and others | ESR1 | Breast | MCF-7 | 401 | 62/31605 | 0.13/0.26 | -0.4 | -0.3 | -0.78 | FALSE |
| SRX8886225 | TFs and others | NFKB1 | Breast | MDA-MB-231 | 503 | 12/31605 | -0.03/0.26 | -0.4 | -0.3 | -0.77 | FALSE |
| SRX18014930 | TFs and others | JUN | Breast | MCF 10A | 3398 | 281/31605 | 0.20/0.26 | -0.4 | -0.3 | -0.77 | FALSE |
| SRX3312985 | TFs and others | PADI2 | Breast | T-47D | 1611 | 44/31605 | 0.11/0.26 | -0.3 | -0.3 | -0.76 | FALSE |
| SRX115967 | TFs and others | ESR1 | Breast | MCF-7 | 1372 | 9/31605 | -0.07/0.26 | -0.3 | -0.3 | -0.76 | FALSE |
| SRX10306479 | TFs and others | SMARCC1 | Breast | T-47D | 1023 | 64/31605 | 0.14/0.26 | -0.3 | -0.3 | -0.74 | FALSE |
| SRX8886227 | TFs and others | NFKB1 | Breast | MDA-MB-231 | 782 | 154/31605 | 0.18/0.26 | -0.3 | -0.3 | -0.74 | FALSE |
| SRX3011702 | TFs and others | MED12 | Breast | MCF-7 | 430 | 12/31605 | -0.02/0.26 | -0.3 | -0.3 | -0.73 | FALSE |
| SRX19495894 | TFs and others | PML | Breast | MDA-MB-231 | 303 | 6/31605 | -0.13/0.26 | -0.3 | -0.3 | -0.73 | FALSE |
| SRX1012758 | TFs and others | PGR | Breast | MCF-7 | 684 | 7/31605 | -0.10/0.26 | -0.3 | -0.3 | -0.73 | FALSE |
| SRX10164662 | TFs and others | PAD2 | Breast | MCF-7 | 1105 | 4/31605 | -0.20/0.26 | -0.3 | -0.3 | -0.72 | FALSE |
| SRX14756939 | TFs and others | DNA-RNA hybrid | Breast | MDA-MB-436 | 398 | 57/31605 | 0.13/0.26 | -0.3 | -0.3 | -0.71 | FALSE |
| SRX1995218 | TFs and others | CTCF | Breast | MCF-7 | 1078 | 64/31605 | 0.14/0.26 | -0.3 | -0.3 | -0.70 | FALSE |
| SRX19662632 | TFs and others | MYC | Breast | T-47D | 48 | 14/31605 | 0.02/0.26 | -0.3 | -0.3 | -0.70 | FALSE |
| SRX14960537 | TFs and others | EZH2 | Breast | MCF-7 | 289 | 34/31605 | 0.41/0.26 | -0.3 | -0.3 | 0.70 | TRUE |
| SRX8488026 | TFs and others | Epitope tags | Breast | MDA-MB-231 | 789 | 4/31605 | -0.19/0.26 | -0.3 | -0.3 | -0.69 | FALSE |
| ERX2242404 | TFs and others | CHD8 | Breast | Mammary epithelial cells | 776 | 4/31605 | -0.19/0.26 | -0.3 | -0.3 | -0.69 | FALSE |
| SRX246862 | TFs and others | MBD3 | Breast | MDA-MB-231 | 1017 | 26/31605 | 0.43/0.26 | -0.3 | -0.3 | 0.68 | TRUE |
| SRX1012760 | TFs and others | PGR | Breast | MCF-7 | 205 | 7/31605 | 0.58/0.26 | -0.3 | -0.3 | 0.68 | TRUE |
| ERX371697 | TFs and others | BCL11A | Breast | Mammary epithelial cells | 389 | 14/31605 | 0.02/0.26 | -0.3 | -0.3 | -0.68 | FALSE |
| SRX13581734 | TFs and others | MED1 | Breast | HMLE | 1128 | 41/31605 | 0.12/0.26 | -0.3 | -0.3 | -0.67 | FALSE |
| SRX11005600 | TFs and others | DNMT3B | Breast | MCF10CA1a | 2382 | 115/31605 | 0.33/0.26 | -0.3 | -0.3 | 0.67 | TRUE |
| SRX5089526 | TFs and others | SMARCA4 | Breast | MCF-7 | 726 | 14/31605 | 0.03/0.26 | -0.3 | -0.3 | -0.66 | FALSE |
| SRX060813 | TFs and others | EP300 | Breast | MCF-7 | 599 | 24/31605 | 0.09/0.26 | -0.3 | -0.3 | -0.65 | FALSE |
| SRX2935293 | TFs and others | 8-Hydroxydeoxyguanosine | Breast | MCF 10A | 11809 | 1161/31605 | 0.23/0.26 | -0.3 | -0.3 | -0.65 | FALSE |
| SRX3322541 | TFs and others | EP300 | Breast | Breast epithelium | 1817 | 5/31605 | -0.11/0.26 | -0.3 | -0.3 | -0.64 | FALSE |
| SRX7522851 | TFs and others | STAT3 | Breast | MCF10DCIS.com | 1296 | 87/31605 | 0.17/0.26 | -0.3 | -0.3 | -0.64 | FALSE |
| SRX822012 | TFs and others | KDM1A | Breast | T-47D | 1263 | 27/31605 | 0.10/0.26 | -0.3 | -0.3 | -0.63 | FALSE |
| SRX2947201 | TFs and others | L3MBTL3 | Breast | MDA-MB-231 | 2835 | 118/31605 | 0.18/0.26 | -0.3 | -0.3 | -0.63 | FALSE |
| SRX2194241 | TFs and others | EP300 | Breast | SUM 159PT | 4827 | 193/31605 | 0.20/0.26 | -0.3 | -0.3 | -0.62 | FALSE |
| SRX1709955 | TFs and others | ESR1 | Breast | T-47D | 32 | 4/31605 | -0.14/0.26 | -0.3 | -0.3 | -0.62 | FALSE |
| SRX822009 | TFs and others | CBX3 | Breast | T-47D | 1795 | 19/31605 | 0.07/0.26 | -0.3 | -0.3 | -0.62 | FALSE |
| SRX10664189 | TFs and others | MTF2 | Breast | MCF-7 | 3272 | 199/31605 | 0.31/0.26 | -0.3 | -0.3 | 0.61 | TRUE |
| SRX1142761 | TFs and others | JUN | Breast | BT-549 | 1952 | 61/31605 | 0.16/0.26 | -0.3 | -0.3 | -0.61 | FALSE |
| ERX371663 | TFs and others | BCL11A | Breast | Mammary epithelial cells | 190 | 1/31605 | -0.52/0.26 | -0.3 | -0.3 | -0.60 | FALSE |
| SRX396570 | TFs and others | CBX8 | Breast | Breast cells | 1589 | 101/31605 | 0.33/0.26 | -0.3 | -0.2 | 0.60 | TRUE |
| SRX5527890 | TFs and others | UPF1 | Breast | Breast cancer cells | 1479 | 488/31605 | 0.29/0.26 | -0.3 | -0.2 | 0.59 | TRUE |
| SRX1709954 | TFs and others | ESR1 | Breast | T-47D | 9 | 1/31605 | -0.50/0.26 | -0.3 | -0.2 | -0.59 | FALSE |
| SRX1709956 | TFs and others | ESR1 | Breast | T-47D | 8 | 1/31605 | -0.50/0.26 | -0.3 | -0.2 | -0.59 | FALSE |
| SRX1709960 | TFs and others | ESR1 | Breast | T-47D | 6 | 1/31605 | -0.50/0.26 | -0.3 | -0.2 | -0.59 | FALSE |
| SRX3010235 | TFs and others | EZH2 | Breast | MCF-7 | 401 | 8/31605 | 0.52/0.26 | -0.3 | -0.2 | 0.59 | TRUE |
| ERX371712 | TFs and others | BCL11A | Breast | Mammary epithelial cells | 366 | 16/31605 | 0.07/0.26 | -0.3 | -0.2 | -0.59 | FALSE |
| SRX4561228 | TFs and others | Biotin | Breast | MCF-7 | 586 | 41/31605 | 0.14/0.26 | -0.3 | -0.2 | -0.58 | FALSE |
| SRX1709959 | TFs and others | ESR1 | Breast | T-47D | 48 | 4/31605 | -0.12/0.26 | -0.3 | -0.2 | -0.58 | FALSE |
| SRX5824557 | TFs and others | BRD7 | Breast | SUM 159PT | 1514 | 149/31605 | 0.19/0.26 | -0.2 | -0.2 | -0.58 | FALSE |
| SRX5511306 | TFs and others | ESR1 | Breast | MDA-MB-231 | 715 | 3/31605 | -0.17/0.26 | -0.2 | -0.2 | -0.57 | FALSE |
| SRX2940746 | TFs and others | TP53 | Breast | MCF-7 | 340 | 31/31605 | 0.12/0.26 | -0.2 | -0.2 | -0.57 | FALSE |
| SRX9804375 | TFs and others | DEK | Breast | MCF-7 | 507 | 36/31605 | 0.13/0.26 | -0.2 | -0.2 | -0.57 | FALSE |
| SRX14756938 | TFs and others | DNA-RNA hybrid | Breast | MDA-MB-436 | 414 | 32/31605 | 0.13/0.26 | -0.2 | -0.2 | -0.56 | FALSE |
| SRX2425862 | TFs and others | SMAD3 | Breast | MDA-MB-231 | 5669 | 361/31605 | 0.29/0.26 | -0.2 | -0.2 | 0.55 | TRUE |
| SRX8639784 | TFs and others | ESR1 | Breast | MDA-MB-134-VI | 3031 | 114/31605 | 0.19/0.26 | -0.2 | -0.2 | -0.55 | FALSE |
| SRX285743 | TFs and others | HOXB7 | Breast | BT-474 | 775 | 24/31605 | 0.11/0.26 | -0.2 | -0.2 | -0.55 | FALSE |
| SRX11005599 | TFs and others | DNMT3B | Breast | MCF10CA1a | 1770 | 80/31605 | 0.18/0.26 | -0.2 | -0.2 | -0.55 | FALSE |
| SRX5513520 | TFs and others | MTA1 | Breast | MDA-MB-231 | 84 | 6/31605 | -0.03/0.26 | -0.2 | -0.2 | -0.55 | FALSE |
| SRX17834042 | TFs and others | TP63 | Breast | MCF 10A | 1244 | 65/31605 | 0.17/0.26 | -0.2 | -0.2 | -0.54 | FALSE |
| SRX3536220 | TFs and others | MEPCE | Breast | MDA-MB-231 | 37 | 1/31605 | -0.43/0.26 | -0.2 | -0.2 | -0.53 | FALSE |
| SRX5824555 | TFs and others | BRD7 | Breast | SUM 159PT | 1084 | 15/31605 | 0.08/0.26 | -0.2 | -0.2 | -0.53 | FALSE |
| ERX2242409 | TFs and others | ARID1A | Breast | Mammary epithelial cells | 532 | 4/31605 | -0.08/0.26 | -0.2 | -0.2 | -0.52 | FALSE |
| SRX12771705 | TFs and others | ZMYND8 | Breast | MDA-MB-231 | 104 | 2/31605 | -0.22/0.26 | -0.2 | -0.2 | -0.52 | FALSE |
| SRX1012759 | TFs and others | PGR | Breast | MCF-7 | 625 | 8/31605 | 0.02/0.26 | -0.2 | -0.2 | -0.52 | FALSE |
| SRX2194239 | TFs and others | EP300 | Breast | SUM 159PT | 7666 | 381/31605 | 0.29/0.26 | -0.2 | -0.2 | 0.52 | TRUE |
| ERX371671 | TFs and others | BRD4 | Breast | Mammary epithelial cells | 597 | 45/31605 | 0.16/0.26 | -0.2 | -0.2 | -0.51 | FALSE |
| SRX14613010 | TFs and others | RCOR1 | Breast | T-47D | 975 | 39/31605 | 0.15/0.26 | -0.2 | -0.2 | -0.51 | FALSE |
| SRX6750796 | TFs and others | EZH2 | Breast | MDA-MB-231 | 5844 | 507/31605 | 0.28/0.26 | -0.2 | -0.2 | 0.50 | TRUE |
| SRX2453450 | TFs and others | NLRP2 | Breast | MCF 10A | 553 | 8/31605 | 0.03/0.26 | -0.2 | -0.2 | -0.50 | FALSE |
| SRX4192885 | TFs and others | NFKB1 | Breast | MCF 10A | 2908 | 379/31605 | 0.22/0.26 | -0.2 | -0.2 | -0.49 | FALSE |
| SRX396568 | TFs and others | CBX6 | Breast | Breast cells | 1204 | 117/31605 | 0.20/0.26 | -0.2 | -0.2 | -0.49 | FALSE |
| SRX18895262 | TFs and others | MECP2 | Breast | MCF-7 | 1062 | 6/31605 | -0.00/0.26 | -0.2 | -0.2 | -0.49 | FALSE |
| SRX518260 | TFs and others | CBX5 | Breast | MCF 10A | 204 | 1/31605 | 0.85/0.26 | -0.2 | -0.2 | 0.46 | TRUE |
| SRX10859400 | TFs and others | ARID1A | Breast | BT-549 | 1991 | 76/31605 | 0.19/0.26 | -0.2 | -0.2 | -0.46 | FALSE |
| SRX18895260 | TFs and others | MECP2 | Breast | MCF-7 | 1084 | 7/31605 | 0.03/0.26 | -0.2 | -0.2 | -0.46 | FALSE |
| SRX11374761 | TFs and others | PGR | Breast | T-47D | 1199 | 6/31605 | 0.01/0.26 | -0.2 | -0.2 | -0.46 | FALSE |
| SRX13845691 | TFs and others | HSF2 | Breast | MDA-MB-231 | 83 | 4/31605 | 0.55/0.26 | -0.2 | -0.2 | 0.46 | TRUE |
| SRX14284264 | TFs and others | ARID1A | Breast | HMEC | 575 | 3/31605 | -0.08/0.26 | -0.2 | -0.2 | -0.45 | FALSE |
| SRX17657154 | TFs and others | FOXC1 | Breast | Hs 578T | 1308 | 52/31605 | 0.18/0.26 | -0.2 | -0.2 | -0.44 | FALSE |
| SRX7522853 | TFs and others | BCL9 | Breast | MCF10DCIS.com | 495 | 56/31605 | 0.18/0.26 | -0.2 | -0.2 | -0.44 | FALSE |
| SRX9804376 | TFs and others | DEK | Breast | MCF-7 | 612 | 45/31605 | 0.17/0.26 | -0.2 | -0.2 | -0.44 | FALSE |
| ERX371708 | TFs and others | BCL11A | Breast | Mammary epithelial cells | 228 | 2/31605 | -0.14/0.26 | -0.2 | -0.2 | -0.43 | FALSE |
| SRX1709957 | TFs and others | ESR1 | Breast | T-47D | 18 | 2/31605 | -0.13/0.26 | -0.2 | -0.2 | -0.43 | FALSE |
| SRX5513514 | TFs and others | EZH2 | Breast | MDA-MB-231 | 37 | 5/31605 | 0.02/0.26 | -0.2 | -0.2 | -0.41 | FALSE |
| SRX1012756 | TFs and others | PGR | Breast | MCF-7 | 630 | 8/31605 | 0.07/0.26 | -0.2 | -0.2 | -0.41 | FALSE |
| SRX4671316 | TFs and others | EP300 | Breast | SUM 159PT | 1015 | 46/31605 | 0.18/0.26 | -0.2 | -0.2 | -0.40 | FALSE |
| SRX11005604 | TFs and others | DNMT3B | Breast | MCF10CA1a | 2029 | 91/31605 | 0.20/0.26 | -0.2 | -0.2 | -0.40 | FALSE |
| SRX10664177 | TFs and others | SUZ12 | Breast | MCF-7 | 3148 | 398/31605 | 0.28/0.26 | -0.2 | -0.2 | 0.39 | TRUE |
| SRX17657153 | TFs and others | FOXC1 | Breast | Hs 578T | 4356 | 288/31605 | 0.23/0.26 | -0.2 | -0.1 | -0.38 | FALSE |
| SRX3583258 | TFs and others | ZMYND8 | Breast | MDA-MB-231 | 2875 | 143/31605 | 0.30/0.26 | -0.2 | -0.1 | 0.38 | TRUE |
| SRX1995226 | TFs and others | LMNB1 | Breast | MCF-7 | 239 | 5/31605 | 0.47/0.26 | -0.1 | -0.1 | 0.37 | TRUE |
| SRX11005603 | TFs and others | DNMT3B | Breast | MCF10CA1a | 1821 | 82/31605 | 0.20/0.26 | -0.1 | -0.1 | -0.37 | FALSE |
| SRX471212 | TFs and others | MBD2 | Breast | MCF-7 | 816 | 9/31605 | 0.10/0.26 | -0.1 | -0.1 | -0.36 | FALSE |
| SRX1070164 | TFs and others | PIAS1 | Breast | MDA-MB-453 | 1206 | 15/31605 | 0.14/0.26 | -0.1 | -0.1 | -0.35 | FALSE |
| SRX6750795 | TFs and others | EZH2 | Breast | MDA-MB-231 | 5408 | 547/31605 | 0.27/0.26 | -0.1 | -0.1 | 0.35 | TRUE |
| SRX2425860 | TFs and others | SMAD3 | Breast | MDA-MB-231 | 1583 | 21/31605 | 0.35/0.26 | -0.1 | -0.1 | 0.34 | TRUE |
| SRX822005 | TFs and others | BPTF | Breast | T-47D | 57 | 3/31605 | 0.50/0.26 | -0.1 | -0.1 | 0.32 | TRUE |
| SRX7522852 | TFs and others | STAT3 | Breast | MCF10DCIS.com | 4979 | 357/31605 | 0.28/0.26 | -0.1 | -0.1 | 0.32 | TRUE |
| SRX673735 | TFs and others | PRKDC | Breast | MCF-7 | 389 | 5/31605 | 0.09/0.26 | -0.1 | -0.1 | -0.30 | FALSE |
| SRX1497516 | TFs and others | TRIM28 | Breast | MCF-7 | 1882 | 18/31605 | 0.17/0.26 | -0.1 | -0.1 | -0.29 | FALSE |
| SRX22149143 | TFs and others | KDM2B | Breast | MDA-MB-231 | 1134 | 6/31605 | 0.11/0.26 | -0.1 | -0.1 | -0.29 | FALSE |
| SRX1012753 | TFs and others | PGR | Breast | MCF-7 | 500 | 8/31605 | 0.13/0.26 | -0.1 | -0.1 | -0.28 | FALSE |
| SRX24351556 | TFs and others | Epitope tags | Breast | MB 157 | 635 | 67/31605 | 0.30/0.26 | -0.1 | -0.1 | 0.28 | TRUE |
| SRX10239457 | TFs and others | GFP | Breast | MCF-7 | 126 | 1/31605 | -0.10/0.26 | -0.1 | -0.1 | -0.28 | FALSE |
| SRX18895258 | TFs and others | Epitope tags | Breast | MCF-7 | 1401 | 6/31605 | 0.11/0.26 | -0.1 | -0.1 | -0.28 | FALSE |
| SRX285745 | TFs and others | HOXB7 | Breast | BT-474 | 312 | 11/31605 | 0.15/0.26 | -0.1 | -0.1 | -0.27 | FALSE |
| SRX1671745 | TFs and others | ERBB2 | Breast | SK-BR-3 | 962 | 45/31605 | 0.21/0.26 | -0.1 | -0.1 | -0.26 | FALSE |
| SRX115966 | TFs and others | ESR1 | Breast | MCF-7 | 1444 | 7/31605 | 0.13/0.26 | -0.1 | -0.1 | -0.26 | FALSE |
| SRX7276893 | TFs and others | TOP2B | Breast | MCF-7 | 1773 | 66/31605 | 0.29/0.26 | -0.1 | -0.1 | 0.25 | TRUE |
| SRX4909432 | TFs and others | HDAC2 | Breast | HCC3153 | 279 | 7/31605 | 0.38/0.26 | -0.1 | -0.1 | 0.25 | TRUE |
| SRX7276892 | TFs and others | TOP2B | Breast | MCF-7 | 3593 | 241/31605 | 0.28/0.26 | -0.1 | -0.1 | 0.25 | TRUE |
| SRX1012637 | TFs and others | EP300 | Breast | MCF-7 | 852 | 148/31605 | 0.23/0.26 | -0.1 | -0.1 | -0.23 | FALSE |
| SRX7522850 | TFs and others | STAT3 | Breast | MCF10DCIS.com | 3722 | 284/31605 | 0.27/0.26 | -0.1 | -0.1 | 0.23 | TRUE |
| SRX4671302 | TFs and others | EP300 | Breast | NAMEC8 | 2078 | 102/31605 | 0.23/0.26 | -0.1 | -0.1 | -0.22 | FALSE |
| SRX1012755 | TFs and others | PGR | Breast | MCF-7 | 641 | 9/31605 | 0.16/0.26 | -0.1 | -0.1 | -0.22 | FALSE |
| SRX3110746 | TFs and others | ESR1 | Breast | MCF-7 | 618 | 19/31605 | 0.32/0.26 | -0.1 | -0.1 | 0.22 | TRUE |
| ERX2242405 | TFs and others | BCL11A | Breast | Mammary epithelial cells | 1028 | 6/31605 | 0.14/0.26 | -0.1 | -0.1 | -0.22 | FALSE |
| SRX3070467 | TFs and others | NR3C1 | Breast | MCF 10A | 1582 | 171/31605 | 0.23/0.26 | -0.1 | -0.1 | -0.21 | FALSE |
| SRX5088594 | TFs and others | Cas9 | Breast | MDA-MB-231 | 564 | 6/31605 | 0.36/0.26 | -0.1 | -0.1 | 0.20 | TRUE |
| SRX3629592 | TFs and others | ESR1 | Breast | MCF-7 | 110 | 1/31605 | 0.01/0.26 | -0.1 | -0.1 | -0.19 | FALSE |
| SRX1528506 | TFs and others | SRPK2 | Breast | MCF 10A | 189 | 2/31605 | 0.41/0.26 | -0.1 | -0.1 | 0.17 | TRUE |
| SRX1012641 | TFs and others | EP300 | Breast | MCF-7 | 666 | 127/31605 | 0.24/0.26 | -0.1 | -0.1 | -0.16 | FALSE |
| SRX055312 | TFs and others | KDM5B | Breast | MCF-7 | 357 | 5/31605 | 0.33/0.26 | -0.1 | -0.0 | 0.14 | TRUE |
| SRX5683086 | TFs and others | ESR1 | Breast | T-47D | 3180 | 54/31605 | 0.28/0.26 | -0.0 | -0.0 | 0.14 | TRUE |
| SRX5954736 | TFs and others | FOSL1 | Breast | MDA-MB-231 | 13099 | 901/31605 | 0.25/0.26 | -0.0 | -0.0 | -0.14 | FALSE |
| SRX3110717 | TFs and others | ESR1 | Breast | MCF-7 | 1016 | 40/31605 | 0.28/0.26 | -0.0 | -0.0 | 0.13 | TRUE |
| SRX7522857 | TFs and others | STAT3 | Breast | MCF10DCIS.com | 8291 | 559/31605 | 0.25/0.26 | -0.0 | -0.0 | -0.13 | FALSE |
| ERX371716 | TFs and others | BRD4 | Breast | Mammary epithelial cells | 967 | 92/31605 | 0.24/0.26 | -0.0 | -0.0 | -0.12 | FALSE |
| SRX5359053 | TFs and others | RORC | Breast | HCC70 | 22928 | 7185/31605 | 0.25/0.26 | -0.0 | -0.0 | -0.12 | FALSE |
| ERX371713 | TFs and others | BRD4 | Breast | Mammary epithelial cells | 2172 | 237/31605 | 0.26/0.26 | -0.0 | -0.0 | 0.11 | TRUE |
| SRX10205615 | TFs and others | SMARCA4 | Breast | CAL-120 | 135 | 2/31605 | 0.16/0.26 | -0.0 | -0.0 | -0.11 | FALSE |
| ERX371701 | TFs and others | BRD4 | Breast | Mammary epithelial cells | 953 | 102/31605 | 0.27/0.26 | -0.0 | -0.0 | 0.10 | TRUE |
| SRX17657152 | TFs and others | FOXC1 | Breast | Hs 578T | 2420 | 120/31605 | 0.25/0.26 | -0.0 | -0.0 | -0.08 | FALSE |
| SRX115969 | TFs and others | ESR1 | Breast | MCF-7 | 1567 | 7/31605 | 0.29/0.26 | -0.0 | -0.0 | 0.07 | TRUE |
| SRX1709962 | TFs and others | ESR1 | Breast | T-47D | 18 | 4/31605 | 0.30/0.26 | -0.0 | -0.0 | 0.06 | TRUE |
| SRX13340674 | TFs and others | Epitope tags | Breast | T-47D | 28 | 1/31605 | 0.19/0.26 | -0.0 | -0.0 | -0.05 | FALSE |
| SRX7644288 | TFs and others | CPSF3L | Breast | MCF-7 | 95 | 1/31605 | 0.20/0.26 | -0.0 | -0.0 | -0.05 | FALSE |
| SRX14756936 | TFs and others | DNA-RNA hybrid | Breast | MDA-MB-436 | 609 | 38/31605 | 0.25/0.26 | -0.0 | -0.0 | -0.04 | FALSE |
| SRX20152810 | TFs and others | FOXA1 | Breast | MDA-MB-231 | 2935 | 234/31605 | 0.25/0.26 | -0.0 | -0.0 | -0.03 | FALSE |
| SRX5231783 | TFs and others | MYC | Breast | UACC-812 | 80 | 2/31605 | 0.28/0.26 | -0.0 | -0.0 | 0.03 | TRUE |
| SRX2662146 | TFs and others | SMARCE1 | Breast | HMLE | 228 | 2/31605 | 0.28/0.26 | -0.0 | -0.0 | 0.03 | TRUE |
| SRX2915387 | TFs and others | PGR | Breast | T-47D | 170 | 3/31605 | 0.24/0.26 | -0.0 | -0.0 | -0.02 | FALSE |
| SRX039140 | TFs and others | FOS | Breast | MCF-7 | 323 | 7/31605 | 0.25/0.26 | -0.0 | -0.0 | -0.02 | FALSE |
| SRX386759 | TFs and others | SMARCC1 | Breast | MCF-7 | 206 | 3/31605 | 0.24/0.26 | -0.0 | -0.0 | -0.02 | FALSE |
| ERX371678 | TFs and others | BCL11A | Breast | Mammary epithelial cells | 196 | 1/31605 | 0.24/0.26 | -0.0 | -0.0 | -0.01 | FALSE |
| SRX3536222 | TFs and others | GFP | Breast | MDA-MB-231 | 123 | 1/31605 | 0.24/0.26 | -0.0 | -0.0 | -0.01 | FALSE |
| SRX3629598 | TFs and others | ESR1 | Breast | MCF-7 | 89 | 1/31605 | 0.24/0.26 | -0.0 | -0.0 | -0.01 | FALSE |
| SRX673736 | TFs and others | PRKDC | Breast | MCF-7 | 114 | 1/31605 | 0.24/0.26 | -0.0 | -0.0 | -0.01 | FALSE |
| SRX5511301 | TFs and others | ESR1 | Breast | MDA-MB-231 | 711 | 2/31605 | 0.25/0.26 | -0.0 | -0.0 | -0.01 | FALSE |
| SRX10859417 | TFs and others | SS18 | Breast | BT-549 | 1848 | 27/31605 | 0.25/0.26 | -0.0 | -0.0 | -0.00 | FALSE |
